# DNA barcodes reveal striking arthropod diversity and unveil seasonal patterns of variation in the southern Atlantic Forest

**DOI:** 10.1371/journal.pone.0267390

**Published:** 2022-04-28

**Authors:** Belén Bukowski, Sujeevan Ratnasingham, Priscila E. Hanisch, Paul D. N. Hebert, Kate Perez, Jeremy deWaard, Pablo L. Tubaro, Darío A. Lijtmaer

**Affiliations:** 1 División Ornitología, Museo Argentino de Ciencias Naturales "Bernardino Rivadavia" (MACN-CONICET), Buenos Aires, Argentina; 2 Centre for Biodiversity Genomics, University of Guelph, Guelph, Ontario, Canada; Southeastern Louisiana University, UNITED STATES

## Abstract

The Atlantic Forest harbors 7% of global biodiversity and possesses high levels of endemism, but many of its component taxa remain unstudied. Due to the importance of tropical forests and the urgency to protect them, there is a compelling need to address this knowledge gap. To provide more information on its arthropod fauna, a Malaise trap was deployed for 12 months in a semi-degraded area of the southern Upper Paraná ecoregion of the Atlantic Forest. All specimens were DNA barcoded and the Barcode Index Number (BIN) system was employed to assign each specimen to a species proxy. DNA barcodes were obtained from 75,500 arthropods that included representatives of 8,651 BINs. Nearly 81% of these BINs were first records, highlighting the high rates of endemism and lack of study of arthropods from the Atlantic Forest. Diptera was the most abundant order, followed by Hemiptera, Lepidoptera and Hymenoptera. Diptera was also the most species-rich order, followed by Hymenoptera, Lepidoptera, and Coleoptera, a result consistent with studies in other biogeographic regions. Insects were most abundant in winter and most diverse in autumn and winter. This pattern, however, was caused mainly by the dynamics of dipteran diversity as other orders differed in their seasonal variation. The BIN composition of the insect community varied sharply through the year and also differed between the two consecutive summers included in the sampling period. The study of the 38 commonest BINs showed that seasonal patterns of abundance were not order-specific. Temperature had the strongest impact on seasonal abundance variation. Our results highlight the striking and understudied arthropod diversity of the highly fragmented Atlantic Forest, the predominance of dipterans, and the fact that abundance and richness in this insect community peak in the coolest months. Standardized studies like this generate fast and reliable biodiversity inventories and unveil ecological patterns, thus providing valuable information for conservation programs.

## Introduction

Arthropods dominate terrestrial ecosystems, representing more than half the world’s described biodiversity, far outnumbering other known organisms [1,2], and occupying varied functional niches and microhabitats [3,4]. In addition, they possess four attributes that make them well-suited for environmental monitoring: small body size, high reproductive capacity, acute sensitivity to environmental changes, and ease of sampling [5]. Therefore, they can provide insights into ecosystem integrity, habitat heterogeneity, the development and recovery of forest ecosystems after natural and anthropogenic disturbance, and the degree of forest fragmentation and isolation [4–8]. However, until now, the inclusion of arthropods in broad taxonomic biodiversity inventories has been hampered by the many undescribed species and the difficulty of morphologically discriminating known taxa. In this context, inventories of arthropod diversity are needed to better understand species distributions and ecological patterns, especially in heterogeneous and highly diverse ecosystems.

DNA barcoding employs a short, standardized genomic region–a fragment of the mitochondrial cytochrome *c* oxidase subunit I (COI) gene in the case of animals–for specimen identification and species discovery [9]. The potential of DNA barcoding to rapidly discriminate and objectively differentiate species [10] combined with the capacity of Malaise traps [11] to collect large numbers of flying insects with little effort is enabling assessments of the species composition of terrestrial arthropod communities in diverse ecosystems [12–22]. As a consequence, the Global Malaise Trap Program (www.globalmalaise.org) was launched in 2012 with the goal of documenting and comparing arthropod diversity at sites around the world. This program has so far involved more than 60 institutions that are studying arthropods at selected sites, but whose collective efforts are creating a global dataset on the temporal and spatial patterning of terrestrial arthropod communities.

The Atlantic Forest is one of the most diverse Neotropical habitats, harbouring around 7% of the world’s known flora and fauna [23,24]. It hosts more than 20,000 species of plants, about 1,500 species of terrestrial vertebrates, and an undetermined number of invertebrates (mainly arthropods), many undescribed [24–30]. In addition, and in spite of its cycles of connection and disconnection with other Neotropical forests (Andean Forest, Amazonian Forest; [31–34]), more than 8,000 plants and 550 vertebrates are endemic to the Atlantic Forest, so it hosts almost 5% of all known endemic species [35–37]. Its biota is now at risk because much of the forest has been transformed by agricultural expansion and urbanization [4,38–40], resulting in a highly fragmented landscape that retains only 7–8% of the original forest cover [23,38] and high deforestation rates continue [41]. This combination of high biodiversity, endemism, and loss of habitat led to the inclusion of this region as one of the 25 most important biodiversity hotspots for conservation [35,38].

The Upper Paraná Atlantic Forest, comprising the southwestern section of the Atlantic Forest in northeastern Argentina, eastern Paraguay, and southeastern Brazil, is its largest ecoregion [38] and the most distant from the Atlantic Ocean. It occupied around 470,000 km^2^ before large-scale anthropogenic transformation, but currently occupies less than 8% of this area, mainly due to agricultural expansion westwards in Brazil [38,42]. As a result, the Upper Paraná ecoregion is now a mosaic of primary or near pristine forest, including the largest remnant (10,000 km^2^) of continuous Atlantic Forest [23], surrounded by large blocks of forestry plantations and secondary forest. Both of the latter habitats have much lower ecological value than primary forest due to their far lower plant diversity and simpler structure [4,39,43–47].

Despite considerable biological data on the Atlantic Forest [5,24,48,49], biodiversity assessments are scarce in the Upper Paraná Atlantic Forest. The knowledge of its arthropod diversity is particularly limited ([29,50,51] represent some past investigations in the area). This study begins to address this deficit by DNA barcoding specimens collected through the year-long deployment of a Malaise trap near the southern limits of the Atlantic Forest. The results provide important information on the diversity of arthropods at this site while also revealing patterns of seasonal variation in the abundance and richness of the insect community. In addition, a more detailed analysis of the commonest species examines the influence of the main climatic variables on their abundance patterns.

## Materials and methods

### Study area, collection and environmental conditions

**Fig 1:**
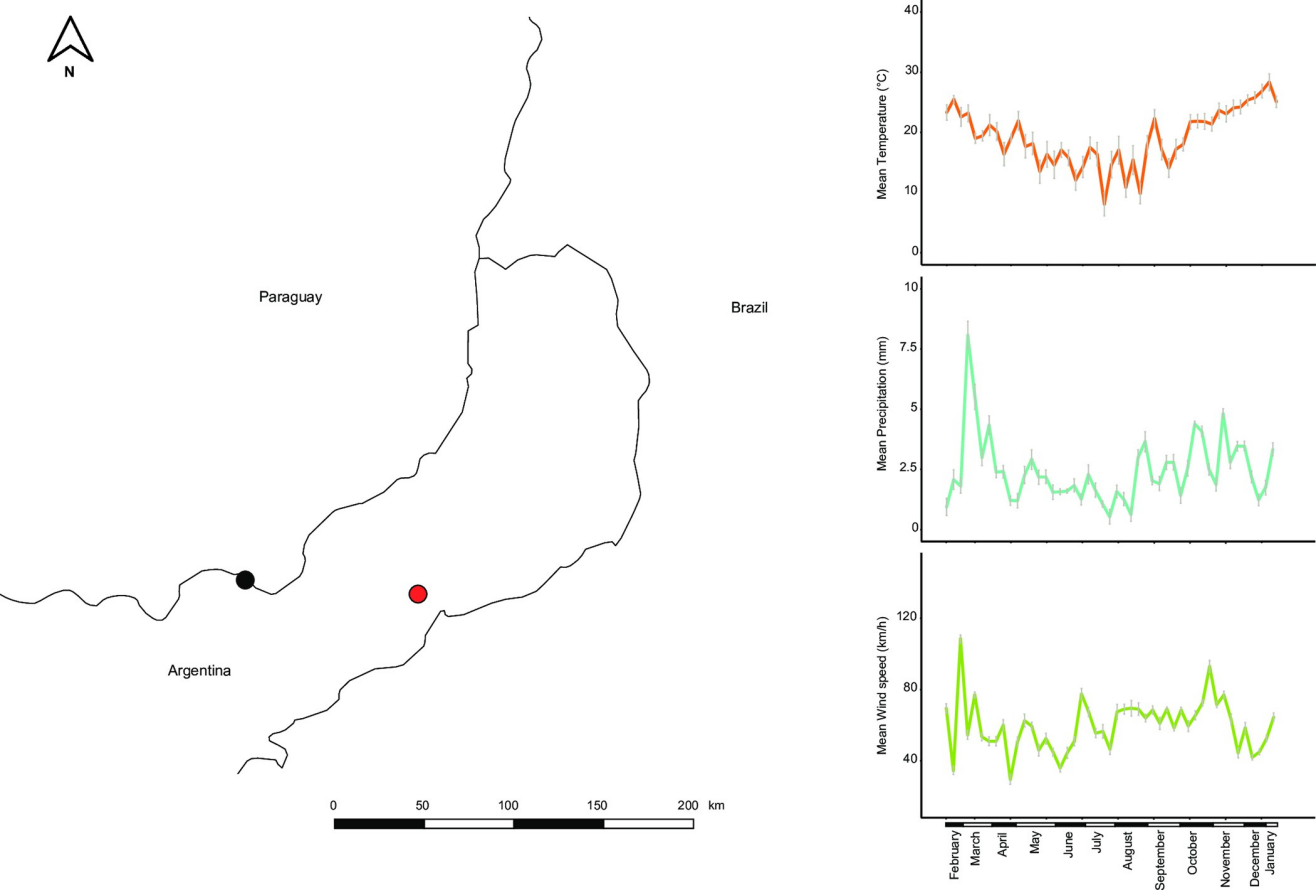
Map of the study area in Misiones province, Argentina. The location of the Centro de Investigaciones Antonia Ramos (CIAR), where the Malaise trap was deployed, is indicated by a red dot. The location of the weather station is indicated by a black dot. The insets to the right show the variation in the climatic variables throughout the sampling period (shown in more detail in S1 Fig).

A Malaise trap was deployed at the Centro de Investigaciones Antonia Ramos (CIAR) in the department of Oberá in southern Misiones province, Argentina (27.44476°S, 54.94032°W, Fig 1). This reserve, which includes around 7 km^2^ of secondary forest that is under a conservation and reforestation program, represents the southern section of a 50 km^2^ patch of semi-degraded forest southwest of the largest, 10,000 km^2^ remnant of continuous pristine forest [37]. As part of the Upper Paraná ecoregion, the climate is subtropical with seasonal variation, both in temperature and rainfall, but without marked wet and dry seasons. Mean daily temperature ranges from 16°C in winter to 26°C in summer while annual rainfall ranges between 1,000–2,200 mm [38].

The trap was deployed from February 2013 to February 2014 (collection permits 135/2012 and 056/2013 from the Ministerio de Ecología y Recursos Naturales Renovables of Misiones province were obtained to deploy this trap and analyze the specimens that it captured). Because this single Malaise trap captured a tiny fraction of the local arthropod population, our research did no harm to populations of the resident species. Sample bottles containing 96% ethanol were changed weekly except for the last two samples which were left for 2 and 3 weeks respectively, so 49 samples were available for analysis. After each catch was harvested, a new bottle was placed on the trap, the ethanol was refreshed in the collected specimens, and samples were held at -20°C in the Museo Argentino de Ciencias Naturales “Bernardino Rivadavia” (MACN) in Buenos Aires until their dispatch to the Centre for Biodiversity Genomics (CBG) at the University of Guelph, Canada.

Meteorological conditions were obtained from the closest weather station with complete climatic data in www.wunderground.com for the entire sampling period. This was the IPOSADAS7 station (27.362°S, 55.903°W), which is also located within the Upper Paraná ecoregion in Posadas city, 95 km west of the trap and at the same elevation. Mean temperature, mean precipitation per hour, and mean wind speed, all averaged by week, were used as climatic variables. Their values for the study period are shown in S1 Fig. The lowest temperatures and precipitation occurred from May to mid-September (i.e., the last month of autumn and through winter), with the coldest week in mid-July having a mean temperature of 8°C. Conversely, the highest temperatures were recorded from mid-November to the end of February (i.e., the last month of spring and the first two months of summer) and the rainiest weeks occurred in March (i.e., the last month of summer).

### Specimen sorting and DNA barcoding

Individuals from each sample were sorted and DNA barcoded following a standard protocol [18]. All arthropods were barcoded excepting a few morphospecies in the order Collembola and the family Cicadellidae (Hemiptera) that were very abundant (50–300 individuals) in many samples.

Specimens were partitioned by size and assigned to an order. Specimens that were too large to fit into a well in a 96-well plate were pinned or placed in a Matrix storage tube and a single leg was removed for DNA extraction. Smaller specimens were placed directly in 96-well microplates and each specimen was recovered after DNA extraction and stored individually in 96-well plates in 96% ethanol [52].

DNA extraction, PCR, and unidirectional Sanger sequencing were conducted at the Canadian Centre for DNA Barcoding (CCDB) following the standard protocol to recover the DNA barcode region [18]. A standard primer pair, C_LepFolF (cocktail of LCO1490 and LepF1) and C_LepFolR (cocktail of LepR1 and HCO2198) [53,54], was used to amplify the 658 bp barcode region of the cytochrome *c* oxidase subunit I (COI) for all arthropods excepting Hemiptera, where the primer pair LepF2_t1 and LepR1 [18,54] was used. All specimen and sequence data were uploaded to the Barcode of Life Data Systems (BOLD, www.boldsystems.org; [55]) into the container project GMTPQ, where all associated data are publicly available in projects corresponding to each weekly sample. These records have also been incorporated to the data sets DS-GMTPQ1 (doi: dx.doi.org/10.5883/DS-GMTPQ1) and DS-GMTPQ2 (doi: dx.doi.org/10.5883/DS-GMTPQ2) and sequences were deposited in GenBank (accession numbers OM543547– OM552185, OM552279–OM561078, OM575152–OM584192, OM585633–OM595350, OM595416–OM604736, OM604775–OM614586, OM704996–OM714488).

At least a family-level taxonomic assignment was made for each specimen by comparing its COI sequence with DNA barcodes already on BOLD (i.e., "reverse taxonomy"). To implement this, we employed the Barcode Index Numbers (BINs), which are automatically generated by BOLD [56], as our operational taxonomic units (OTUs). BINs are a good proxy for species, as it has been shown for several taxonomic groups, and are essential for large-scale biodiversity assessments, especially when many taxa are undescribed [12–14,16–19,21,29,56–60]. After it is uploaded to BOLD, each COI sequence is assigned to a BIN based on the Refined Single Linkage (RESL) algorithm. If the sequence shows congruence to a known BIN it is assigned to it. Otherwise, it founds a new BIN. In brief, RESL first delimits initial OTUs based on single linkage clustering with a 2.2% threshold of maximum divergence allowed within a cluster (a threshold empirically defined based on the analysis of various datasets [56]). After this step, those OTUs with sequence variation among their members are subjected to Markov clustering, a graph analytical approach, to define if a split within the OTU is justified (due to the presence of internal partitions), a task performed through random walks in the section of the graph surrounding each OTU (see [56] for a more detailed explanation).

Specimens representing new BINs were bidirectionally sequenced to ensure their sequence records were in full compliance with the DNA barcode standard [61]. These new BINs were also examined morphologically to identify them to the best possible taxonomic level and up to three representatives of each BIN were photographed. Finally, all voucher specimens and residual DNA extracts were deposited in the scientific collections at MACN.

### Data analysis

Because the DNA barcode library is constantly growing and new sequences can split or merge BINs, all data from GMTPQ were downloaded on August 26, 2016, and all analyses that included comparisons with the data in BOLD were performed at that time. The taxonomic assignments of BINs were validated by constructing a Neighbor-Joining (NJ) tree that included one representative of each BIN. This was implemented on BOLD [56] using the COI nucleotide data, the Kimura-2-Parameter distance model, and the pairwise deletion option for handling sites containing missing data. The NJ tree (S2 Fig) was inspected for unusual placement of taxa; erroneous taxonomic assignments were subsequently corrected by direct examination of the voucher specimen or its image [19].

The entire data set was analyzed to ascertain the number of specimens and BINs collected over the year-long deployment of the trap. However, because the last two samples had longer duration (2–3 weeks), further analyses focused on the first 47 weeks where collections were weekly. These analyses included all specimens assigned to a BIN and that were identified to at least an ordinal level. We first analyzed the total abundance and richness (BIN count) of the most common orders (i.e., those represented by at least 1% of the total number of collected specimens). The proportion of singleton BINs (i.e., those BINs represented by one individual in the entire sample) in each order was also calculated. To test for significant differences in the proportion of singleton BINs among orders, we used the Marascuilo procedure [62], which enables simultaneous testing of the differences between all pairs of proportions when there are multiple proportion data. To assess the completeness of sampling, the lognormal abundance model was employed to estimate the total BIN richness for the most abundant orders using the vegan package [63] in R software [64]. Species numbers were estimated using the Preston fit and the veiledspec functions from the vegan package [63,65]. As another measure of sampling completeness, BIN accumulation curves were computed for the most common orders using the nonparametric species richness estimator Chao 1 [66,67] in EstimateS 9.1.0 software [68]. In addition, curves were computed as the mean of 1000 randomized species accumulation curves without replacement, and were constructed and extrapolated to twice the actual sample size [17,67].

To explore the variation in abundance and richness of insects through the year, we examined time series distributions for the most common orders. For a more detailed analysis, we then characterized the temporal turnover in BIN composition (in this case by considering all orders in the sample) by calculating the pairwise dissimilarity between all bottles collected using the vegdist function in the vegan package in R. This function computes dissimilarity indices and we employed the Bray-Curtis index. We then performed a Pearson correlation between the dissimilarity index and the temporal distance between sample pairs (using the cor.test function in R) and also used a matrix-based heat map to color code dissimilarity values and visualize community temporal variation.

To better understand the dynamics of the insect community, seasonal variation in specimen counts was assessed for a subset of the most abundant BINs (those present in most weeks, those with the most specimens over the year, with further selection to maximize the number of orders represented). This was done by performing a k-means cluster analysis which aggregated species with similar patterns of seasonal abundance by minimizing the variance within each cluster [69]. This analysis was implemented by calculating the area under the abundance curve for each BIN every five weeks (barring the last segment that spanned seven weeks) using the R-package NbClust [70] based on the algorithm by Hartigan and Wong [69]. To select the optimal number of clusters we used the NbClust function and the distance metric was set to euclidean. To perform k-means clustering, we used the kmeans function with 10 as the number of iterations and the Hartigan-Wong algorithm.

Finally, we performed a Canonical Correspondence Analysis (CCA) to examine the relationship between the abundance patterns of these BINs and three environmental variables (temperature, precipitation, wind speed) to relate shifts in community composition to variation in these parameters [71,72]. The CCA was implemented using the cca function from the vegan package [63] in R.

## Results

Barcode sequences were recovered from 89.8% of the arthropods (67,892/75,589) collected by the year-long deployment of the Malaise trap and 64,330 of these sequences met the quality requirements for a BIN assignment. This recovery success mirrors values in prior studies employing similar protocols [12–14,16–18,21]. Among the 8,651 BINs that were present, 80.7% were first records on BOLD.

**Fig 2:**
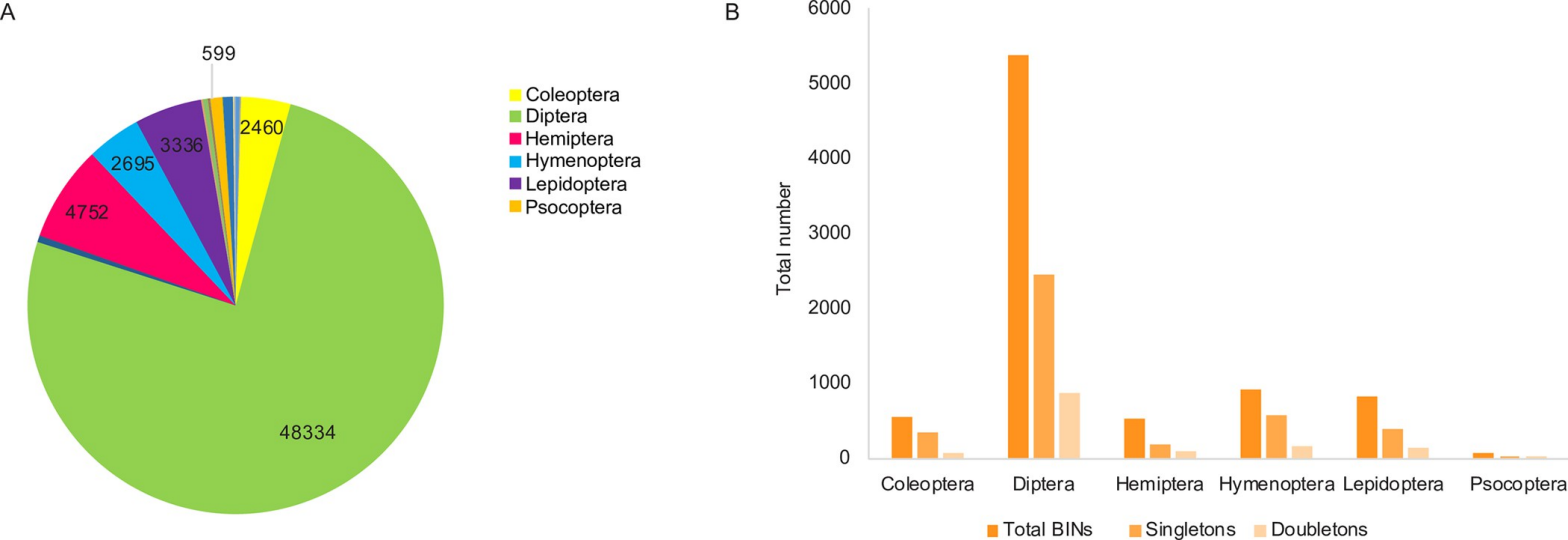
Abundance and richness of the most common orders. (A) Total abundance of each order, detailing the values of the six most common orders; (B) number of total BINs, singletons and doubletons for the six most common orders.

Because the last two samples had longer duration (2–3 weeks), further analyses focused on the first 47 bottles that were collected weekly. They included 63,891 specimens with representatives of 8,581 BINs belonging to 28 orders of arthropods. Diptera comprised 75.7% of these specimens (Fig 2A) with Hemiptera (7.4%), Lepidoptera (5.2%), Hymenoptera (4.2%), Coleoptera (3.9%), and Psocoptera (1%) the only orders comprising at least 1% of the catch. Among the dipteran families, the Sciaridae and Cecidomyiidae were most abundant, each representing about 1/3 of all flies (S3A Fig), while the Cicadellidae was the most abundant (84%) family of hemipterans (S3B Fig). Diptera was also the most BIN-rich order, comprising 2/3 of all BINs (5,382 BINs), followed by Hymenoptera (11%, 910 BINs), Lepidoptera (10%, 824 BINs), Coleoptera (6%, 543 BINs), and Hemiptera (6%, 536 BINs; Fig 2B). Among dipterans, the most BIN-rich family was Cecidomyiidae (53%) (S3C Fig), while among Hymenoptera the richest insect family was Braconidae (34%), followed by Ichneumonidae (27%) (S3D Fig). Within dipterans, Cecidomyiidae had the most richness (53% of BINs) and was also one of the most abundant families (more than 30% of Diptera), whereas Sciaridae was the most abundant family (34% of collected flies) but possessed far fewer BINs (7% of BINs). The remaining BINs belonged to 22 orders including six of Arachnida (Araneae, Mesostigmata, Opiliones, Pseudoscorpiones, Sarcoptiformes, Trombidiformes), 13 of Insecta (Archaeognatha, Blattodea, Embioptera, Ephemeroptera, Mantodea, Neuroptera, Odonata, Orthoptera, Phasmatodea, Plecoptera, Strepsiptera, Thysanoptera, Trichoptera), and three of Collembola (Entomobryomorpha, Poduromorpha, Symphypleona). Collectively, these 22 orders comprised just 2.7% of the specimens and 3.6% of the BINs.

As nearly 50% of the BINs (4,146) were represented by a single specimen, and another 20% by only two individuals (Fig 2B), many species await collection. Hymenoptera and Coleoptera had a significantly higher proportion of singletons than the other orders based on the Marascuilo procedure (S1 Table), suggesting a lower fraction of the total BINs in these orders were collected. Consistent with the high percentage of singletons, presuming a lognormal abundance distribution just 2/3 of the Malaise-trappable insects at the site were captured. Specifically, the current BIN counts represent about 60% of the dipteran species at the site, 70% of the Hymenoptera, 74–79% of the Hemiptera and Lepidoptera, and 54–57% of the Coleoptera and Psocoptera (S4 Fig). In accordance with these results, the BIN accumulation curves indicate that the asymptote was not reached for any order, and that it would not have been achieved even if twice as many insects were analyzed from each order (S5 Fig). The asymptotic species richness estimator Chao 1 suggested that the current sample collected 80% of the hemipteran BINs, 73–74% of the BINs of Diptera, Lepidoptera and Psocoptera, and 66% of those of Hymenoptera and Coleoptera.

**Fig 3:**
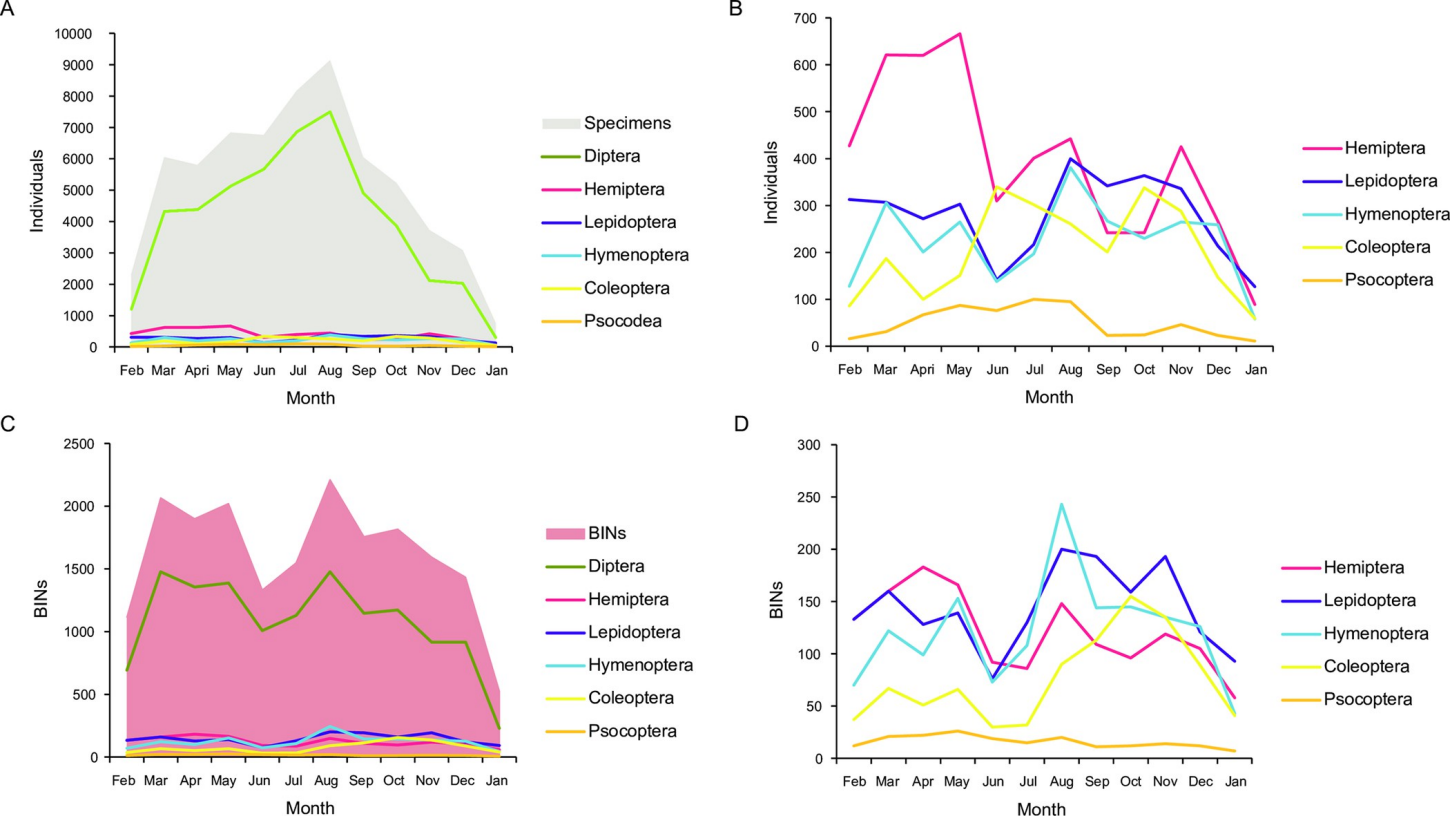
Abundance and richness variation in the most common orders between February 2013 and January 2014. (A) Total number of specimens and number of specimens for each of the most abundant orders throughout the year; (B) details on the number of specimens per order excluding Diptera; (C) total BINs and BINs for each of the most abundant orders throughout the year; (D) details of the BIN counts for the orders excluding Diptera.

Considered overall, the abundance of the six most common orders was lowest in spring and summer, rose in fall, and peaked in winter (Fig 3A). However, this pattern was mainly driven by the Diptera as the abundance trajectories for the other orders was variable (hemipterans peaked in autumn while the abundance of other orders was relatively stable throughout the year; Fig 3B).

BIN richness was also highest in August, but the overall number of BINs was similar in autumn and winter (Fig 3C). As with abundance, this pattern was mainly driven by Diptera. Hymenoptera and Hemiptera presented a pattern consistent with that of Diptera, but other orders showed different trends as the richness of Lepidoptera and Coleoptera peaked in late winter and spring while the richness of Psocoptera was stable through the year (Fig 3D).

**Fig 4:**
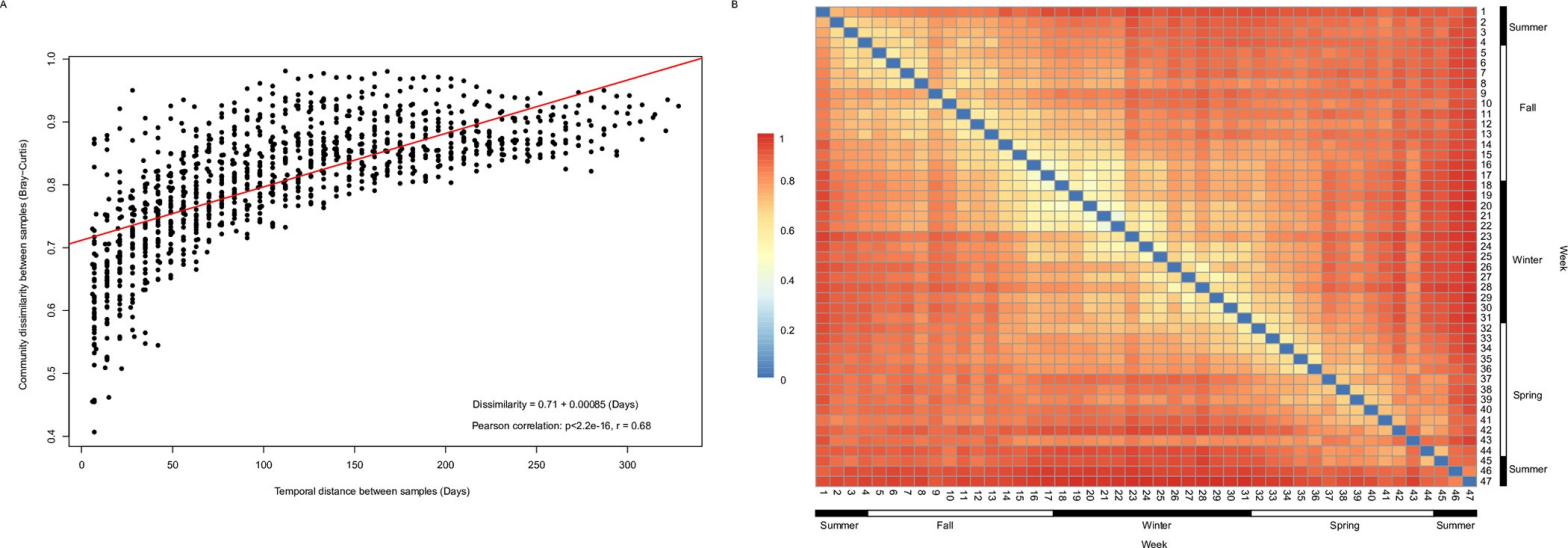
Analysis of the insect community composition throughout the year using the Bray-Curtis dissimilarity index. (A) Temporal decay curve for the insect community showing more dissimilarity as the samples compared are more distant in time. (B) Heat map of pairwise comparisons of the insect community. Hot colors represent high dissimilarity between samples and cold colors represent low dissimilarity between samples. The blue diagonal represents the dissimilarity of each sample compared to itself.

To quantify variation in the insect community through the year (temporal turnover of BINs), we calculated pairwise dissimilarity between all samples. This analysis showed that community composition varied sharply throughout the year as the Bray-Curtis dissimilarity index averaged 0.81 (range = 0.40–0.98). In fact, even samples collected a week apart typically showed high dissimilarity in their BINs (mean Bray-Curtis dissimilarity index for all pairs of consecutive weeks = 0.63 ± 0.10), and dissimilarity rose as the samples compared were more distant in time (Pearson correlation; p < 0.001; Fig 4). Surprisingly, samples from February 2013 and January 2014 were among the most dissimilar (Bray-Curtis dissimilarity index = 0.91 ± 0.02) although both were made in mid-summer. Only 4–7% of the BINs from February 2013 were recaptured in January 2014, suggesting marked variation among consecutive years in composition of the insect community.

As is typical for insect communities [73], most BINs were too uncommon to allow statistical analysis of their abundance shifts on a weekly basis (S6 Fig). As a result, we only assessed the seasonal dynamics of the 38 most abundant BINs based on joint consideration of their specimen count and their presence in the weekly samples. These 38 BINs included representatives from six families of Diptera (Cecidomyiidae, Ceratopogonidae, Chironomidae, Phoridae, Sciaridae, Tachinidae), four of Lepidoptera (Bucculatricidae, Gelechiidae Geometridae, Tineidae), and one each of Hemiptera (Cicadellidae), Hymenoptera (Formicidae), Coleoptera (Curculionidae), and Psocoptera (Caeciliusidae) (S2 Table). Only two of these BINs (ACC4180 and AAZ4402) could be identified to a genus; both were ants belonging to *Pheidole* [29].

**Fig 5:**
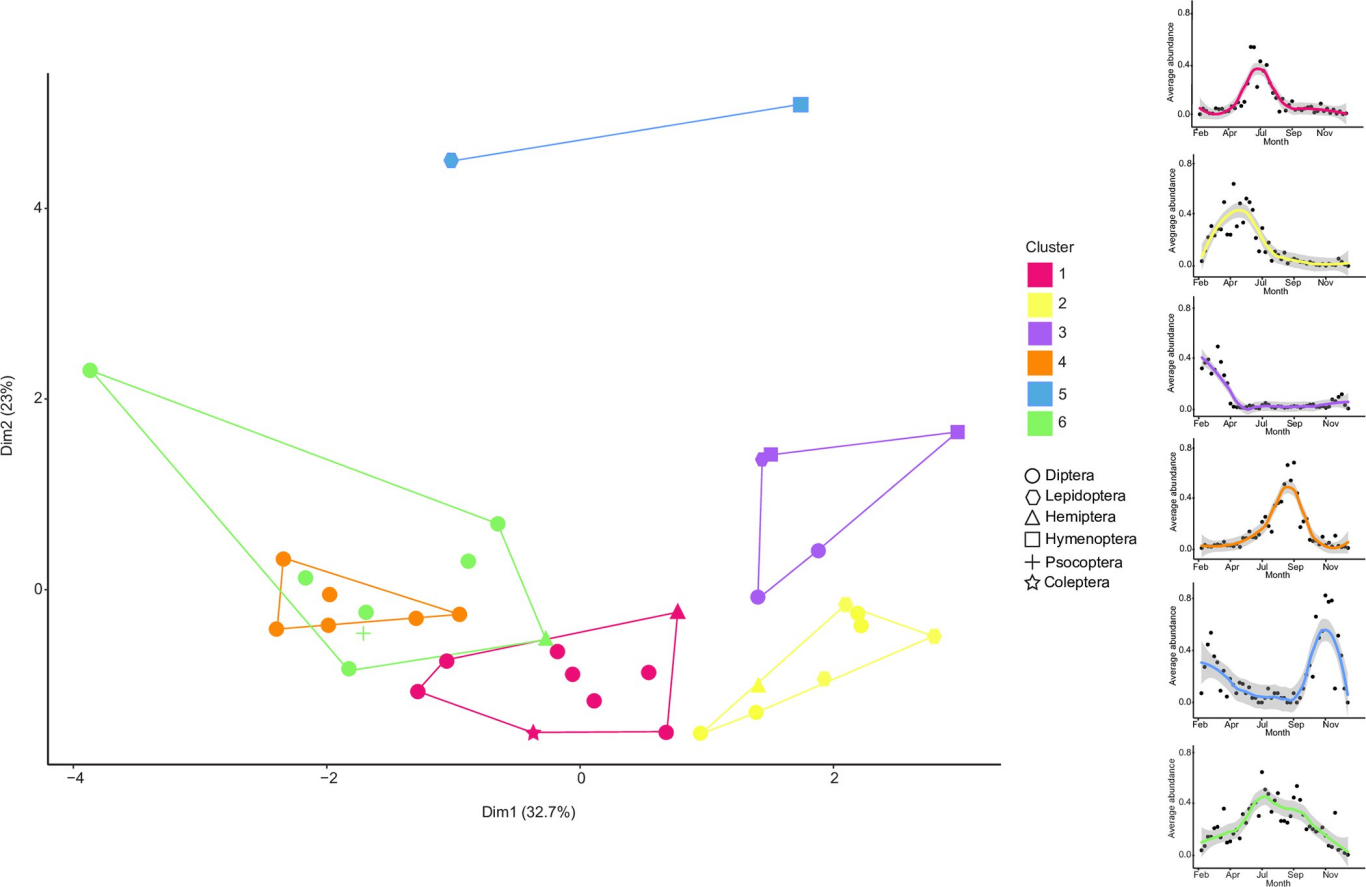
Cluster plot following a k-means analysis using the algorithm of Hartigan and Wong [69]. The 38 most common BINs were grouped into six clusters based on their abundance distributions over the year. The insets to the right show the abundance distribution pattern of each cluster throughout the year (see S7 Fig for more detail).

A k-means analysis grouped these 38 BINs into six clusters (Fig 5) based on their differing patterns of abundance through the year: cluster 1 peaked in early winter (late June to early July), cluster 2 during autumn (end of April through May), cluster 3 in late summer (February and March), cluster 4 in late winter (late July to September), cluster 5 in early summer (December), while cluster 6 had a bimodal abundance distribution (S7 Fig). Five of these six clusters included BINs from different orders. Consistent with the general patterns of abundance described above, the clusters (1, 4) that peaked in winter were dominated by dipterans.

**Fig 6:**
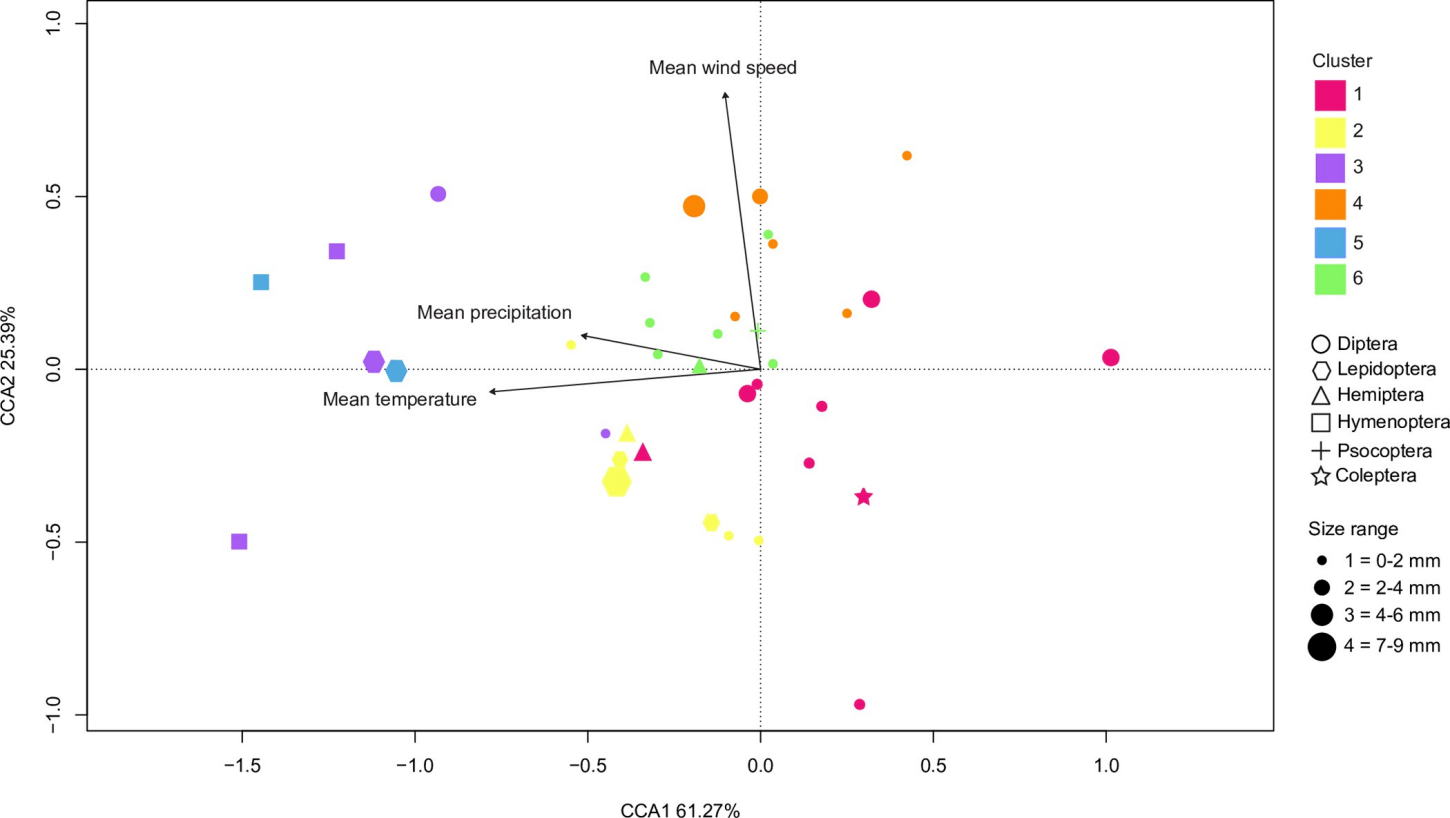
Canonical Correspondence Analysis (CCA) of the abundance distribution of BINs and the environmental variables. Arrows indicate the direction and magnitude of the effect of each environmental variable on the 38 most common BINs of the insect community.

To further investigate which environmental variable (temperature, precipitation, wind speed; S1 Fig) had the greatest impact on abundance distributions of the insect community, we performed a Canonical Correspondence Analysis (CCA). Its first two axes explained 86.5% of the variation in abundance. Based on an analysis of variance (ANOVA) with 999 permutations, two environmental variables, mean temperature and mean wind speed, were found to have a significant effect in the CCA (mean temperature: F = 4.69, *p* = 0.001; mean wind speed: F = 2.02, *p* = 0.025; mean precipitation: F = 1.35, *p* = 0.16). As expected from this result, mean temperature and mean wind speed had longer vectors in the ordination plot than mean precipitation (Fig 6), indicating their stronger influence on the patterning of seasonal BIN abundance. These results are consistent with the clusters identified by the k-means analysis: BINs on the left of the plot tend to peak in the summer (those in clusters 3 and 5), BINs in the center of the plot belong to cluster 2 (peak in autumn) or 6 (bimodal distribution with an extended period of abundance) while BINs on the right tend to have higher abundance in the winter (clusters 1 and 4).

The interpretation of the relationship between BIN abundance in the trap and mean wind speed was not straightforward. First, mean wind speed did not show a clear pattern of annual variation in contrast to precipitation and temperature (see S1 Fig). In addition, the vertical distribution of the BINs in the ordination plot was not clearly correlated with the k-means clusters or to the shared patterns of seasonal variation in abundance among BINs. In fact, the BINs for which abundance appears to be positively correlated with wind speed (i.e., those in the upper part of the ordination plot) belong to different clusters (mainly clusters 3 and 4, but also clusters 5 and 6) and did not always show peak abundance in months with the highest wind speeds.

## Discussion

The year-long deployment of a Malaise trap in the Upper Paraná ecoregion of the Atlantic Forest collected more than 75,000 specimens belonging to over 8,650 BINs. As such, this trap collected more specimens and BINs than any of the other 60 traps so far deployed through the Global Malaise Trap Program [74]. Although the number of BINs is unlikely to correspond to the same number of species, the strong correlation between BIN and species counts documented in several animal groups [56] indicates that the insect fauna of the southern Atlantic Forest is exceptionally diverse. Moreover, more than 6,900 of these BINs were new to BOLD, so this trap also added the greatest number of new BINs within the Global Malaise Trap Program, making clear that the insects of the region are severely understudied.

The high biodiversity at the study site is remarkable if one considers its location. Specifically, the trap was located at the southern extreme of the Atlantic Forest in a small forest fragment 200 km distant from the major (10,000 km^2^) intact patch of the Upper Paraná ecoregion. Moreover, it was secondary forest logged intensively for the last 50 years and with nearby agricultural activity; site restoration was only initiated a decade ago [37,75,76]. Although the insect community in old-growth Atlantic Forest might be more diverse, mixed habitats and forest borders can show increased diversity (e.g. [77,78]). As a result, additional large-scale biodiversity assessments in the region are needed to better understand the high insect richness in this area in the broader context of the entire Atlantic Forest.

Despite the high BIN count, insect richness at this site was clearly much higher. First, projections based on the lognormal species abundance model suggest that a third of the insect species likely to be collected with a Malaise trap await capture (13,143 estimated BINs versus 8,481 collected) and BIN accumulation curves were not asymptotic reflecting the fact that nearly 50% of BINs were represented by a single specimen. Second, previous analyses have shown low species overlap between Malaise traps deployed in close proximity (e.g., [14,17,22]), indicating that the number of species awaiting detection is certain to be much higher than any estimate based on extrapolation from a single trap. Third, the striking seasonal turnover in diversity and the low BIN overlap between the samples of two consecutive summers suggest that many new BINs would be captured with a longer deployment. Finally, many insect species are not collected by Malaise traps because of their morphological traits (e.g., apterous) or flight behavior.

Similar to previous diversity assessments, many of which used Malaise traps coupled with DNA barcoding, Diptera was the most diverse order (over 60% of the BINs), followed by Hymenoptera, and then Lepidoptera. Previous work examined temperate and arctic sites in the northern hemisphere (Canada, [12,18,22]; Germany, [13,20]; Greenland, [14]; Sweden, [79]), the Saharo-Arabian region [16], and tropical rainforests in Central America (Honduras, [17]; Costa Rica, [21]), but the present study in a subtropical forest reinforces evidence that Diptera are the most species-rich insect order rather than Coleoptera which were long held to occupy this position [2,80,81].

Even though dipterans were the most common and diverse order throughout the year, their abundance and richness varied seasonally, peaking in the colder months (highest abundance in winter, when mean daily temperature was 16°C, and highest richness in autumn and winter; see Fig 3). As a result, this was the time of year with the highest abundance and diversity of insects. This contrasts with higher latitudes where, as expected, insect abundance is far lower in the winter (e.g., [13,82,83]). Although temperature is well known to affect insect development (e.g. [84]) and activity (e.g. [85]), prior studies that have analyzed seasonal variation in insect abundance in tropical or subtropical habitats, particularly in the Neotropics, have identified rainfall as the main variable determining abundance. These studies usually examined sites with marked seasonal variation in rainfall and have shown that abundances tend to peak in the wet season or in the transition from the dry to wet season [86–88]. The few analyses in tropical settings without marked seasonal rainfall have also shown that the main factor influencing abundance was rainfall, even when annual changes are subtle [88]. By contrast, insect abundance and richness in the southern Atlantic Forest appear related to temperature as there are no clear changes in rainfall through the year (see S6 Fig) and abundance peaked in the colder months (Fig 3). The role of temperature as the main factor influencing abundance was confirmed by the Canonical Correspondence Analysis (CCA) of the most abundant BINs (see below). Whether this pattern of higher abundance and richness in winter is common to other subtropical forests in the Neotropics awaits study.

Dipterans drove the general trend of seasonal abundance and diversity, but the remaining orders differed in their patterns of abundance. Hemipterans were commonest in the autumn while the other orders did not show a clear trend, suggesting an aseasonal pattern that is common in tropical forests worldwide [88]. In terms of diversity, Hymenoptera was richest in winter, whereas the diversity of Lepidoptera and Coleoptera peaked in late winter and spring. This variation in the patterns of abundance and richness among groups coincides with previous studies and suggests that different orders are differentially affected by climatic factors (e.g. [88–90]).

Consistent with the different pattern among orders, BIN turnover was high. Similarity was highest among samples collected one or two weeks apart, but even in this case BIN similarity rarely exceeded 50%. Moreover, the extremely low similarity between the samples collected in February 2013 and January 2014 (mid-summer of each year) with only 5.7% of shared BINs and a Bray-Curtis dissimilarity index of 0.91 suggests high variation between consecutive years (see Fig 4). This result accords with previous studies in tropical regions that found high inter-annual variation in the insect community, including supra-annual cycles of abundance [17,88].

Study of the commonest BINs showed that their seasonal pattern of abundance was not order-specific. Instead, the species belonging to each order peaked at different times of the year and were therefore distributed among the different clusters in the k-means analysis. For instance, the BINs of Sciaridae were distributed among 5 of the 6 patterns identified in the analysis. This seasonal variability among the most common species in each order, and in some cases even family, could represent a case of temporal niche partitioning in the insect community as previously shown for Coleoptera [91], Hymenoptera [92], petrels [93], rodents [94], and plants [95].

Consistent with the lack of a dry season in the Atlantic Forest and the effect of temperature on the abundance of insects, detailed analysis of the most common species confirmed that temperature is the variable that most influences their seasonal patterns (see Fig 6). However, its influence was complex, impacting different species in different ways and generating the dispersion of seasonal peaks of abundance that existed among BINs, even those in the same family. This indicates that even though cooler months were those with the highest abundance and diversity of insects, particularly dipterans, individual species are differentially affected by temperature. Further study of the most common BINs could shed light on the details and mechanisms determining the differences among species.

## Final remarks

The very high diversity of the insect community revealed at a site near the southern boundary of the Atlantic Forest by deploying just a single year-long Malaise trap shows the value of combining large-scale collection made by Malaise traps with the fast, reliable identification and discrimination of species enabled by DNA barcoding. In particular, the use of the BIN system to automatically assign specimens to operational taxonomic units (OTUs) makes it possible to rapidly assess biodiversity patterns that would otherwise be intractable, including the study of seasonal variation, the comparison of diversity among areas or different years, the evaluation of levels of endemism, and the generation of information on species distributions required to select areas for protection. Moreover, the high proportion of taxa new to BOLD highlights the need for similar analysis in other understudied hyperdiverse environments such as Neotropical forests.

Identifying specimens to the species level within the time frame of large-scale studies such as this one relies on the adoption of "reverse taxonomy" which uses the COI sequences to identify organisms by comparing their sequences with those in the DNA barcode library on BOLD (e.g., [13]). The morphological identification of over 8,500 species is not feasible because the taxonomic specialists needed to carry out the work are unavailable and the demand on their time for such a massive identification program would be extreme. Moreover, the fact that more than 80% of the current BINs were new to the database precluded efforts to examine matters such as the concordance between BINs and taxonomy or the proportion of native vs. invasive species in this area. A clear example of the challenges imposed by the lack of previous records in BOLD for many of the species encountered in this study is indicated by the fact that none of the 38 commonest BINs had been collected before by the iBOL project, and only two could be confidently identified to a genus using the DNA barcode database (see S2 Table). A collaborative effort with taxonomic experts to provide morphology-based identifications for the most common BINs and those with particularly interesting attributes could be an effective strategy for more detailed study of the relationship between their seasonal or geographic abundance variation and morphology, ecology, and evolutionary history.

Finally, expanded studies, both in time and space, are essential to better understand insect diversity in the Atlantic Forest and to clarify patterns detected in this analysis. This is especially relevant given the variation in catch among different traps deployed at a site, the evidence for inter-annual variation in the insect community detected in this study, and the large proportion of BINs represented by a single specimen. An increase in funding to perform such large-scale studies is crucial to better understand biodiversity, not only in the Atlantic Forest but in all hyperdiverse environments.

## Supporting information

S1 Fig Climate data from the Posadas Weather Station. Temperatures were averaged by week, precipitation was the cumulative amount by week and wind speed was summed by week. For each climatic variable, the standard deviation of the mean value of each week is represented with gray bars.
(PDF)

S2 Fig BOLD Taxon ID Tree. Neighbor-Joining tree based on the COI sequence for one representative of each BIN generated using the Kimura-2-Parameter distance model.
(PDF)

S3 Fig Abundance and BIN richness at the family level. Relative abundance of the families within the two most abundant orders: (A) Diptera and (B) Hemiptera. Relative richness of the families of the most BIN-rich orders: (C) Diptera and (D) Hymenoptera.
(PDF)

S4 Fig Lognormal abundance curves for the six most abundant orders. BIN _exp_ = expected BIN count, BIN _obs_ = observed BIN count. All statistical analyses were conducted using R and the vegan package. Estimations of species numbers were based on the Preston fit function.
(PDF)

S5 Fig BIN accumulation curves for the six most abundant orders. The solid line represents the specimen-based rarefaction curve while the dashed line segment extrapolates the curve to double the observed sample size. Color shading indicates the 95% confidence interval. Note the different scales on the axes.
(PDF)

S6 Fig Total number of specimens barcoded for each BIN (abundance of BINs) plotted against the number of weeks in which that BIN was captured (week count). (PDF)

S7 Fig Six clusters of BINs generated with the k-means analysis based on the annual abundance distribution of the 38 most common BINs. The average BIN abundance is shown in the first graph of each cluster (color-coded as in Figs. 5 and 6), and then the abundance distribution of each BIN.
(PDF)

S1 Table Marascuilo procedure for multiple proportion comparison. The pair order compared, the difference of the proportions of the orders compared, the critical value, the Chi-square statistic, and the p-value are informed.
(DOCX)

S2 Table The 38 most abundant BINs detected in the trap during the sampled period. Their taxonomic details, the total number of individuals of each BIN, and the number of weeks in which it was collected are reported.
(DOCX)
