# Supplementary figures and images for: DNA barcodes reveal striking arthropod diversity and unveil seasonal patterns of variation in the southern Atlantic Forest
Source: PLoS One. 2022 Apr 28;17(4):e0267390. doi: 10.1371/journal.pone.0267390 (PMC9049551; doi:10.1371/journal.pone.0267390)

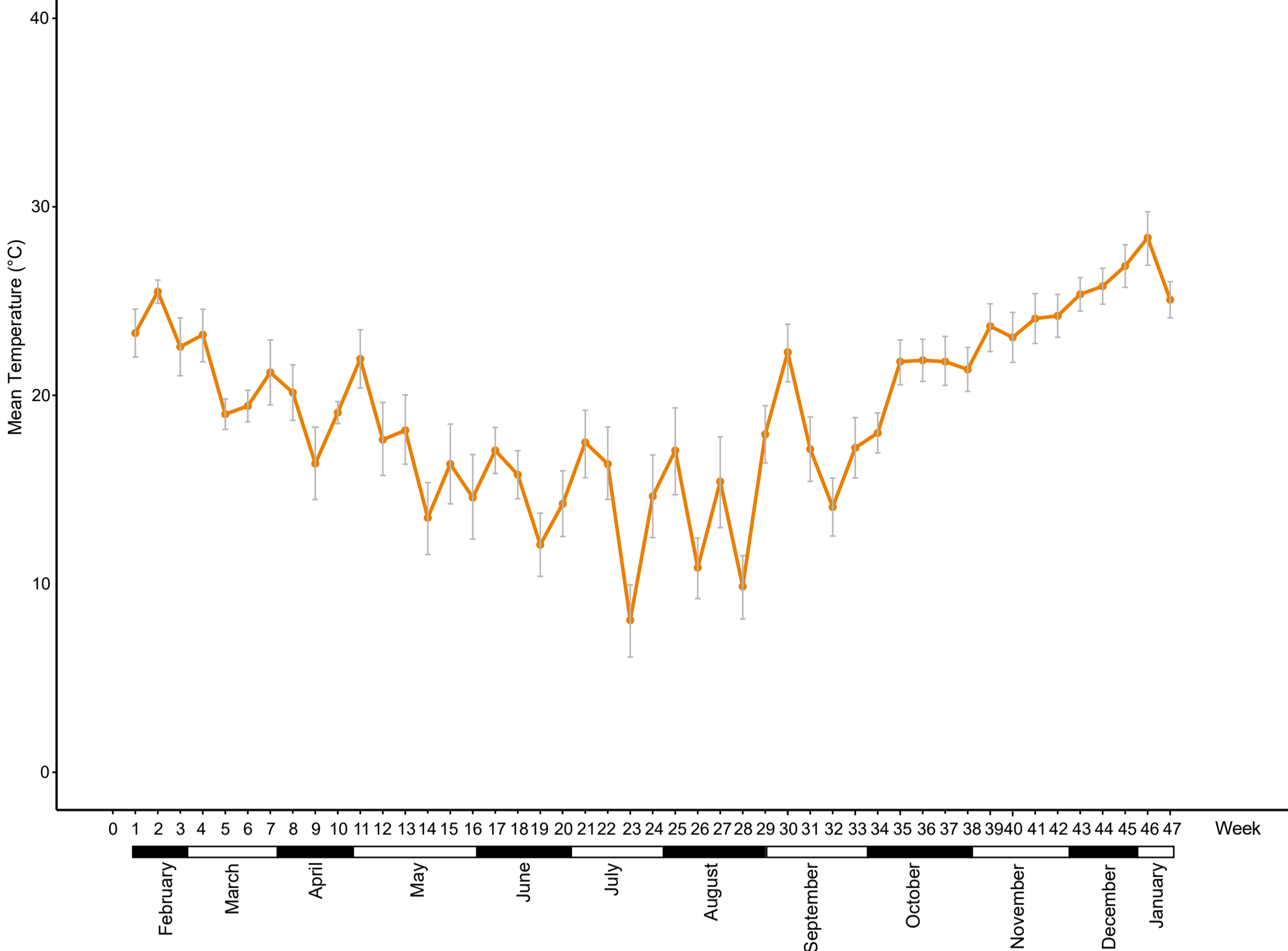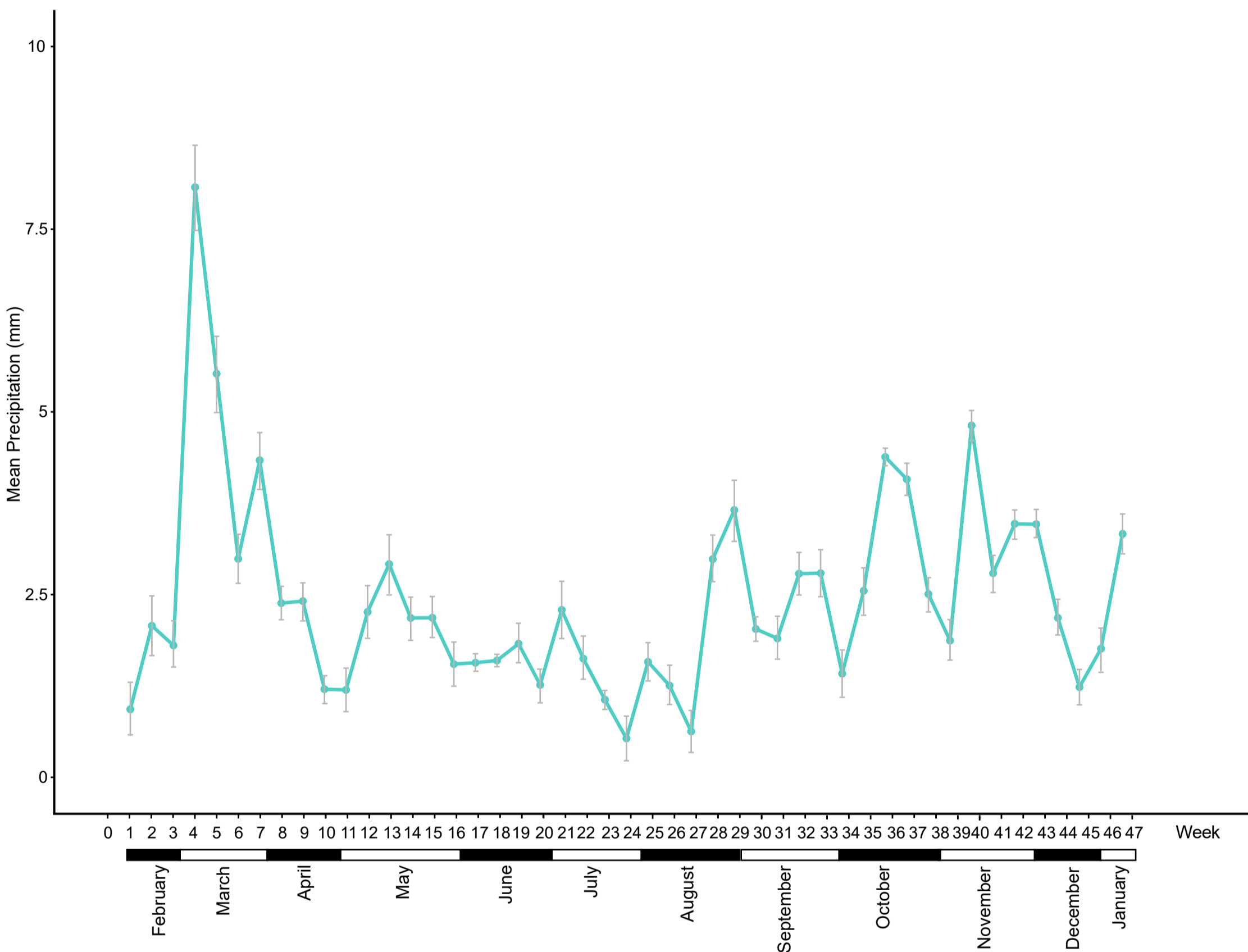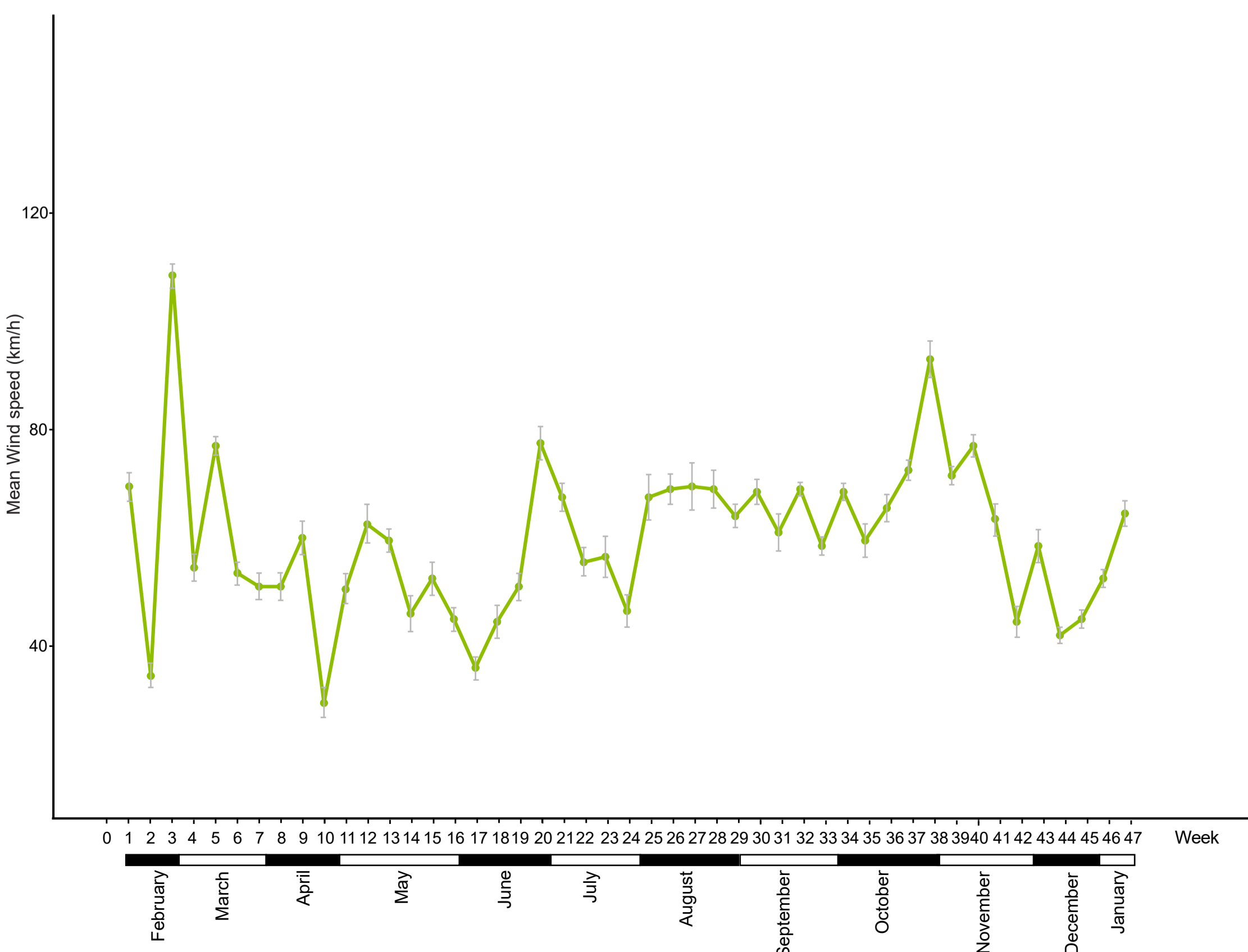

S1 Fig.

Supplement: S1 Fig — Temperatures were averaged by week, precipitation was the cumulative amount by week and wind speed was summed by week. For each climatic variable, the standard deviation of the mean value of each week is represented with gray bars. (PDF) [file pone.0267390.s001.pdf]

A

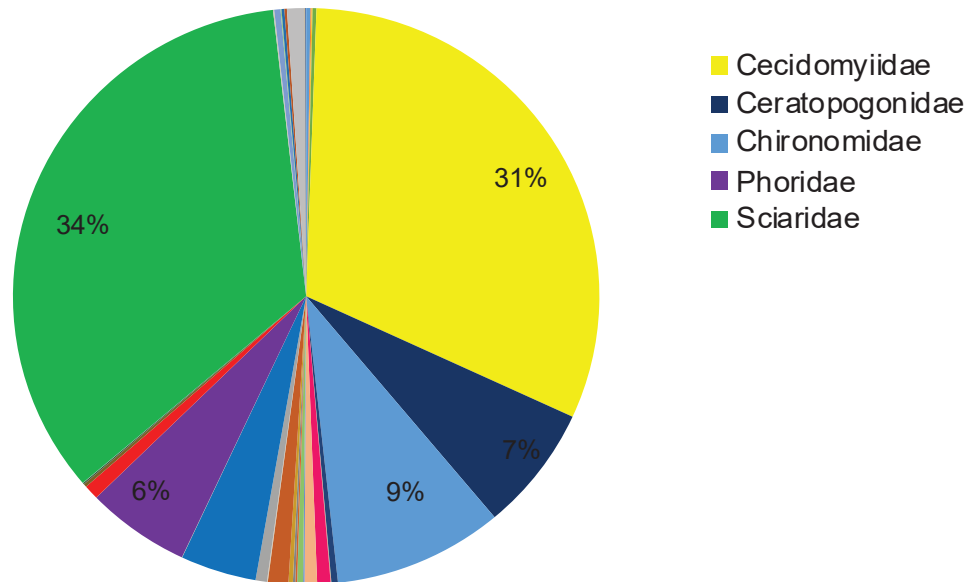

B

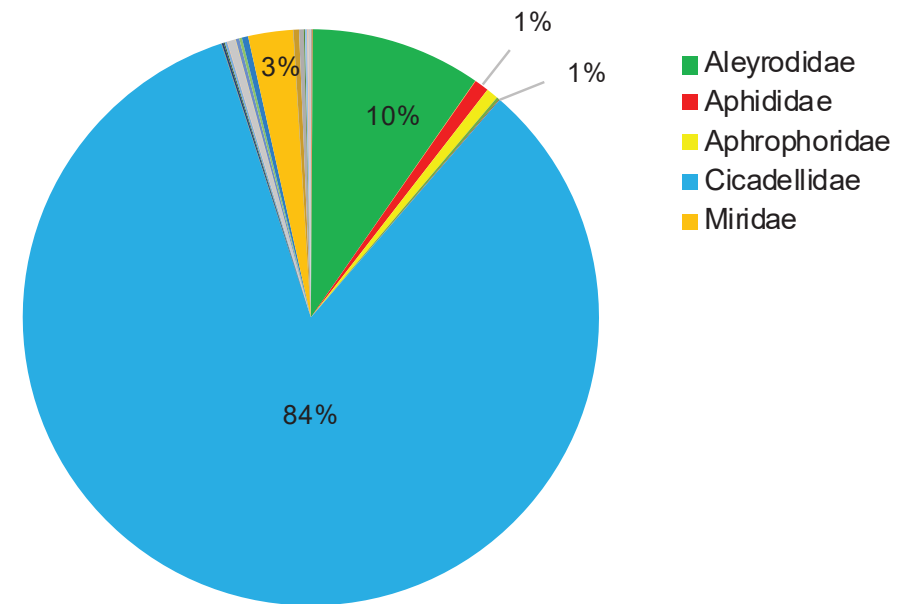

C

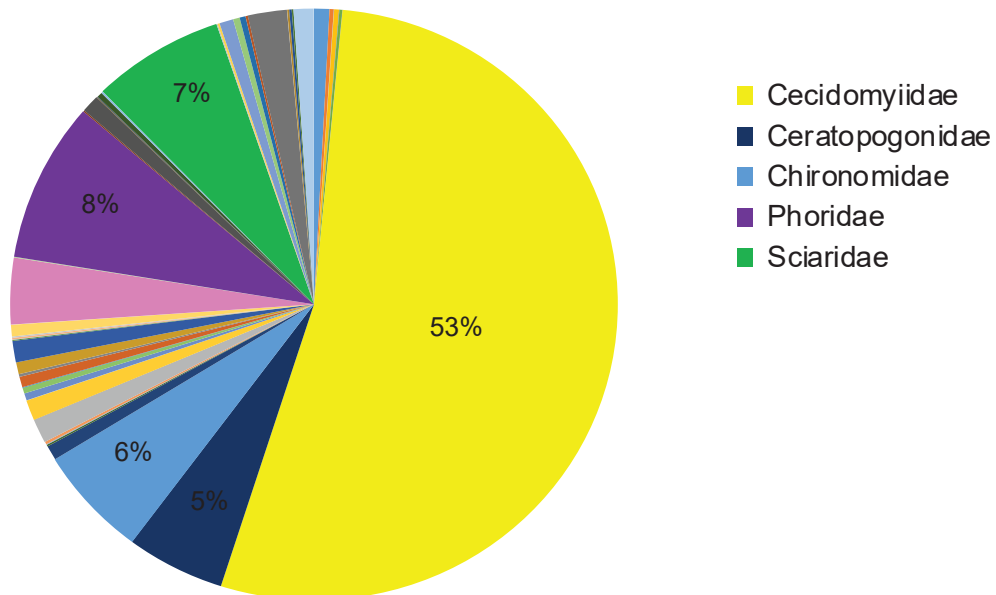

D

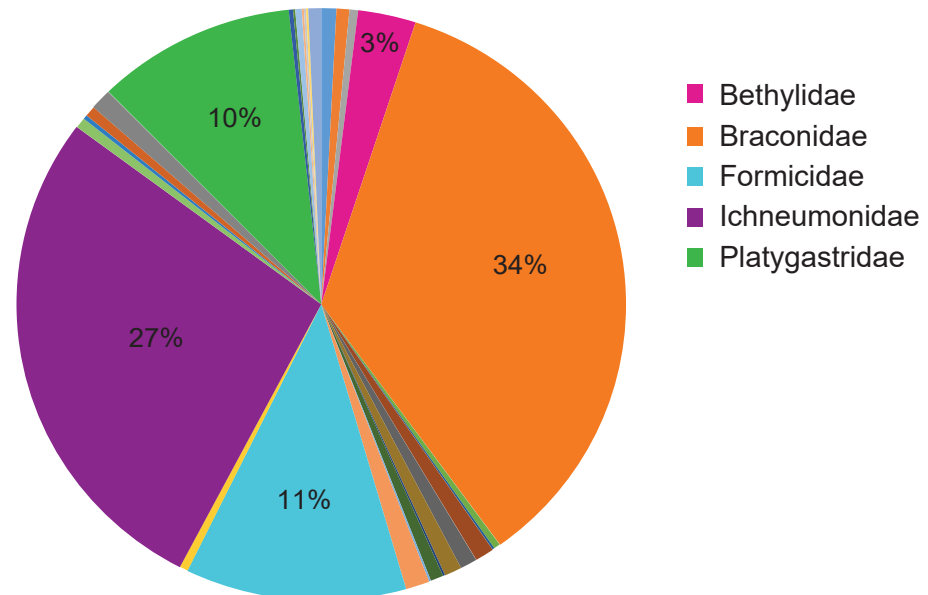

Supplement: S3 Fig — Relative abundance of the families within the two most abundant orders: (A) Diptera and (B) Hemiptera. Relative richness of the families of the most BIN-rich orders: (C) Diptera and (D) Hymenoptera. (PDF) [file pone.0267390.s003.pdf]

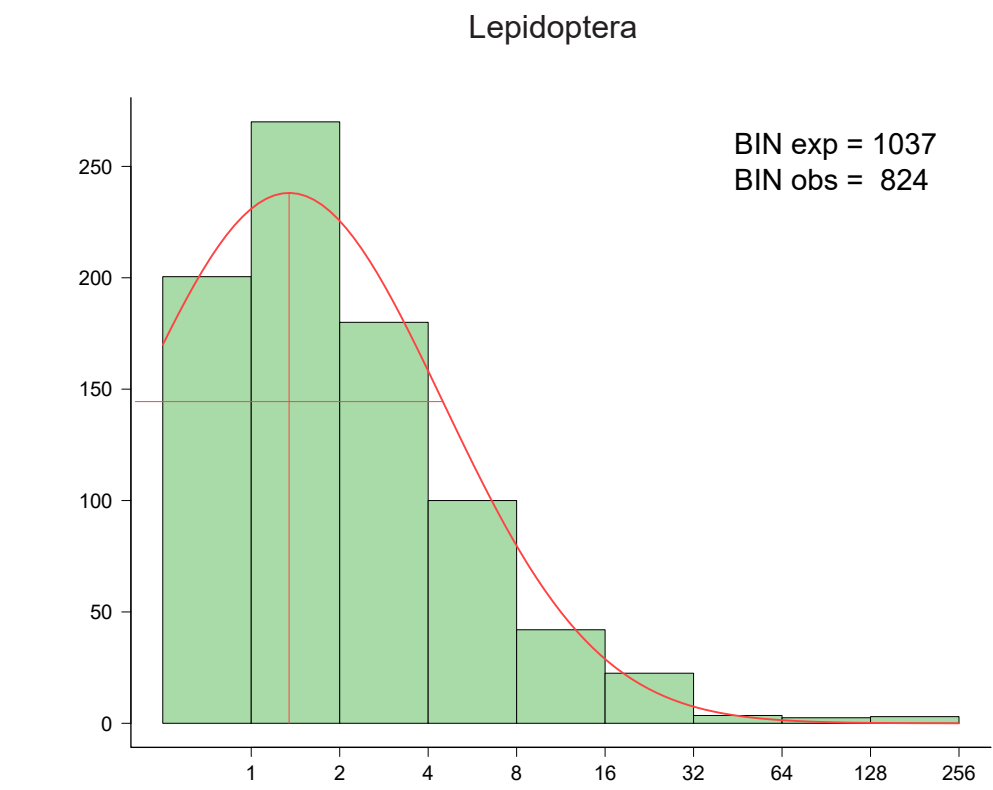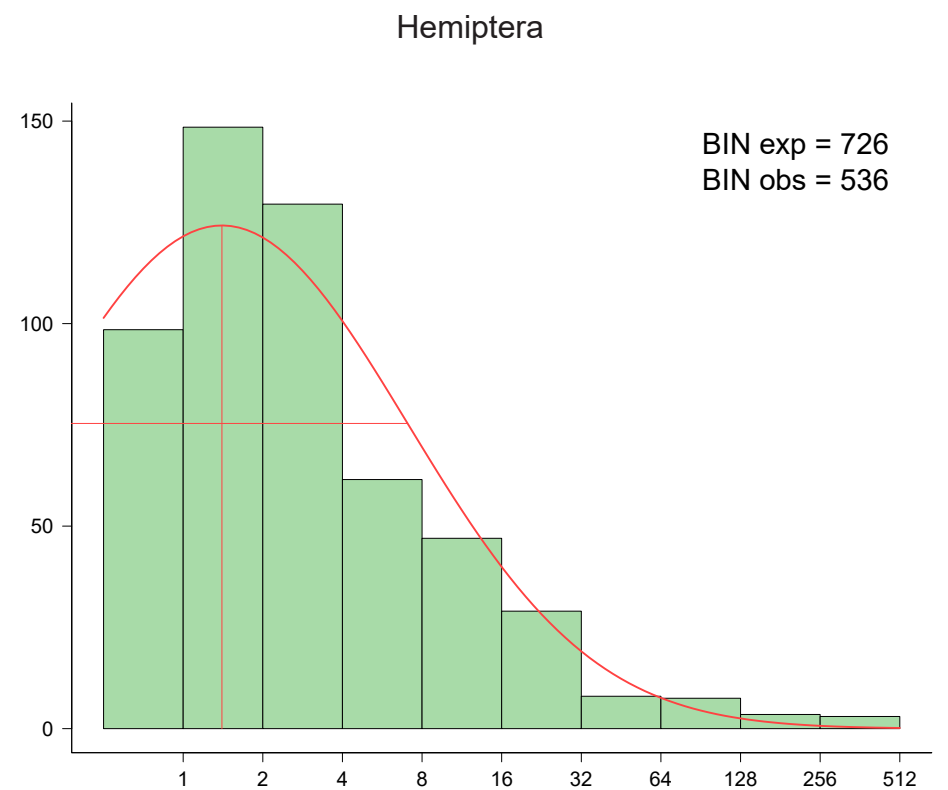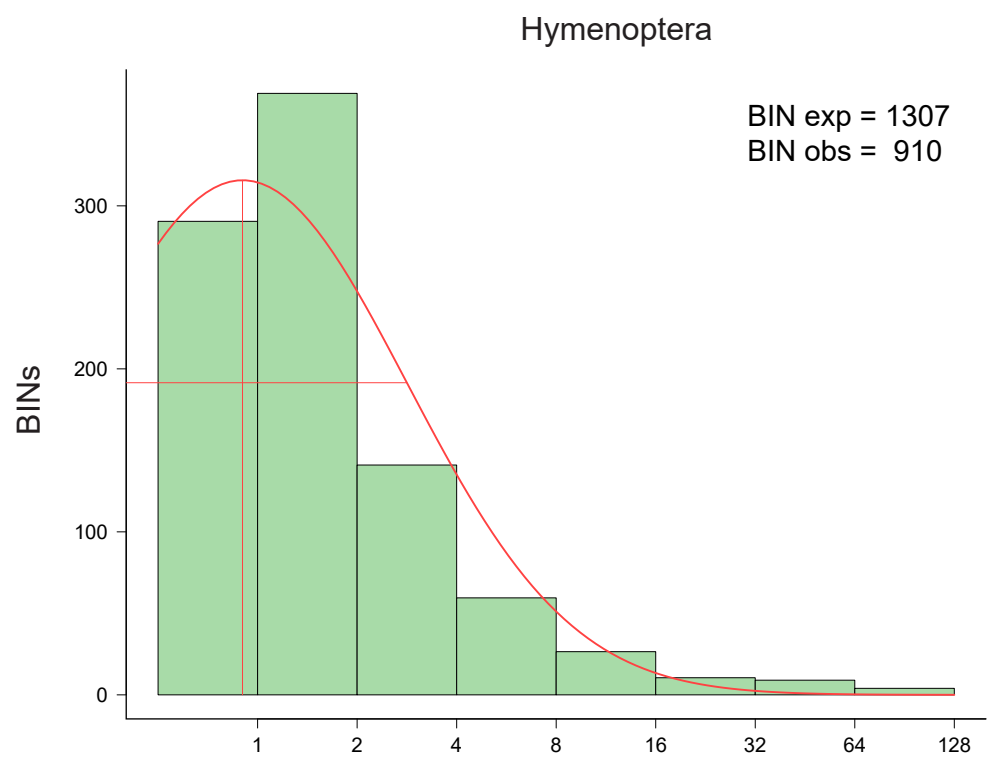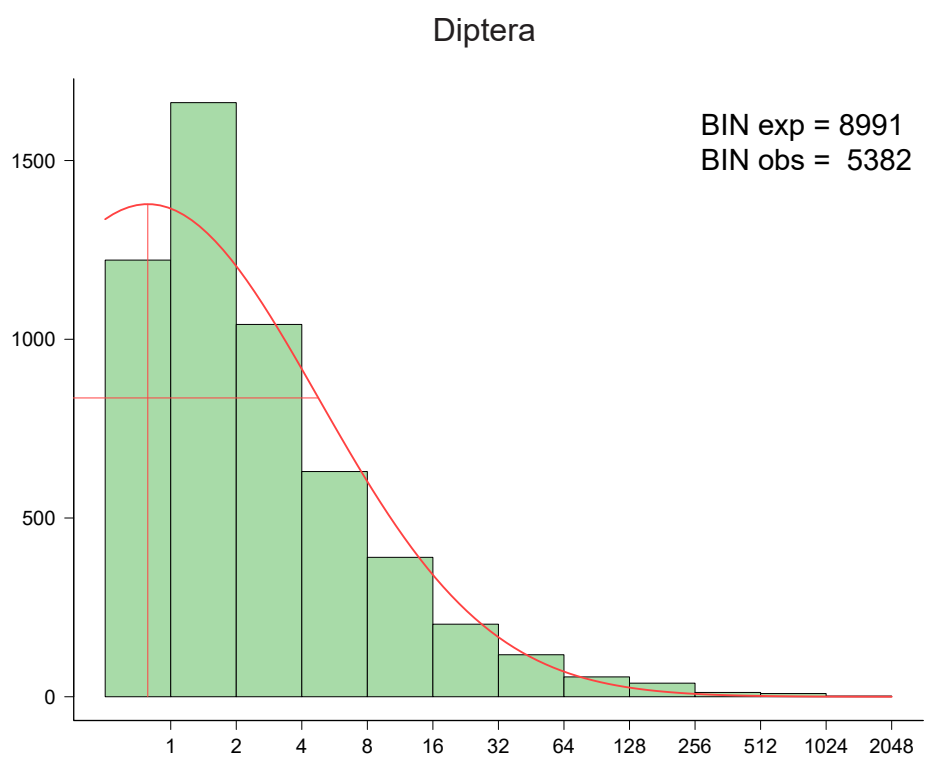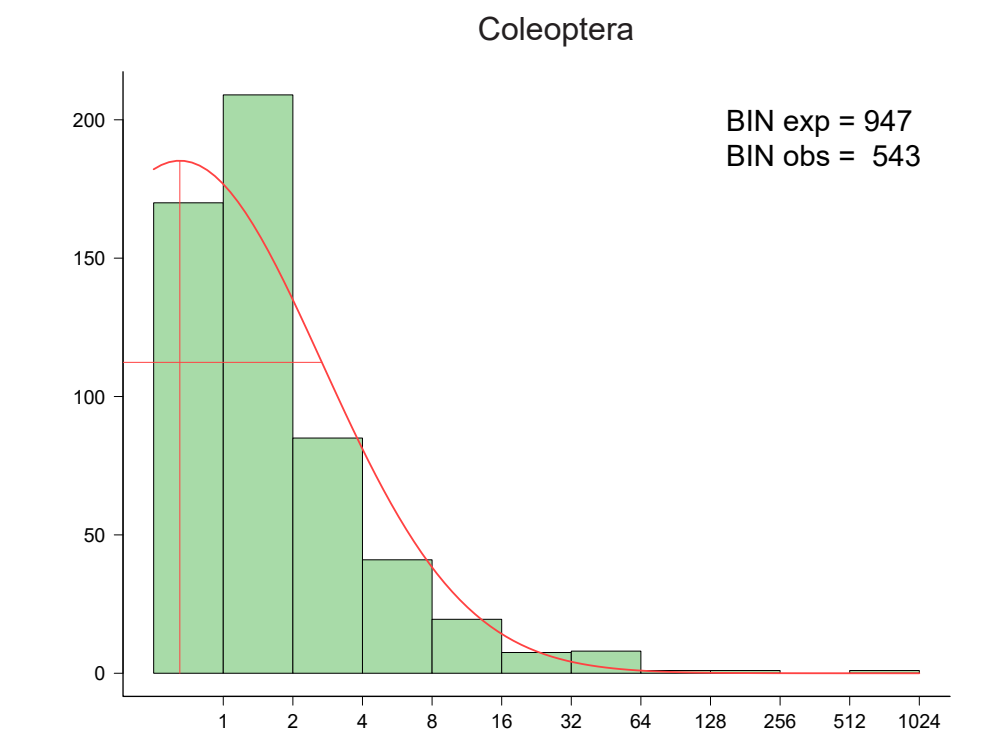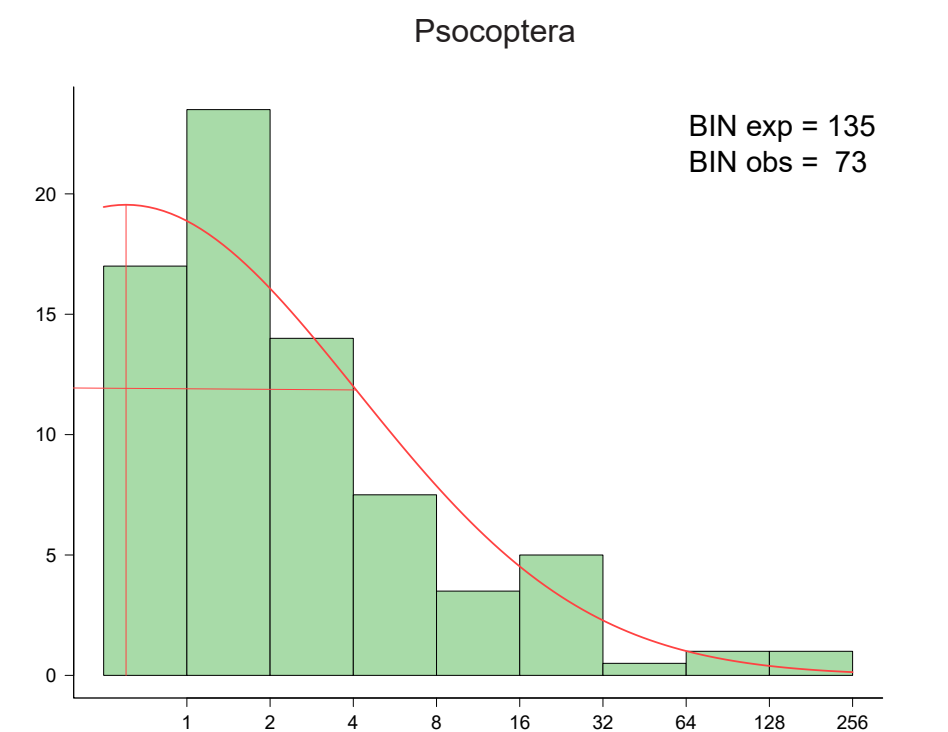

Abundance intervals

Supplement: S4 Fig — BIN exp = expected BIN count, BIN obs = observed BIN count. All statistical analyses were conducted using R and the vegan package. Estimations of species numbers were based on the Preston fit function. (PDF) [file pone.0267390.s004.pdf]

**Diptera**

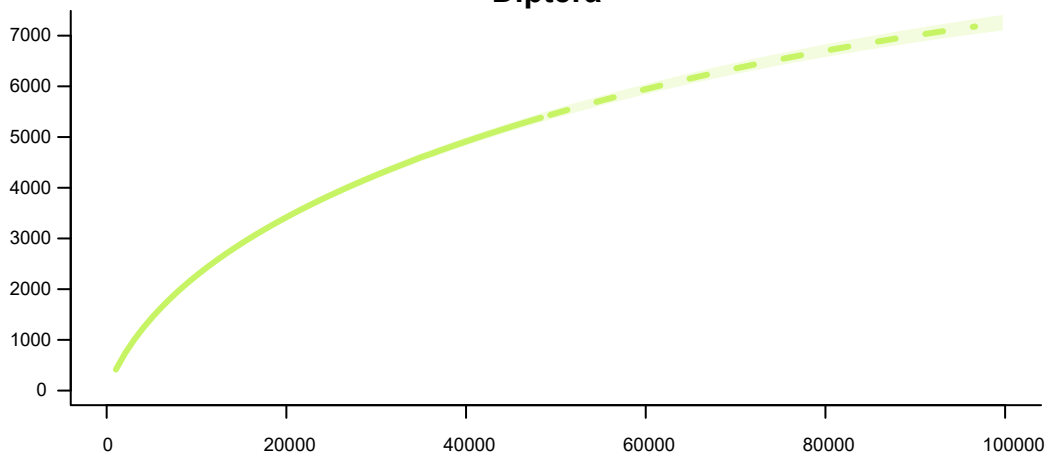

**Hemiptera**

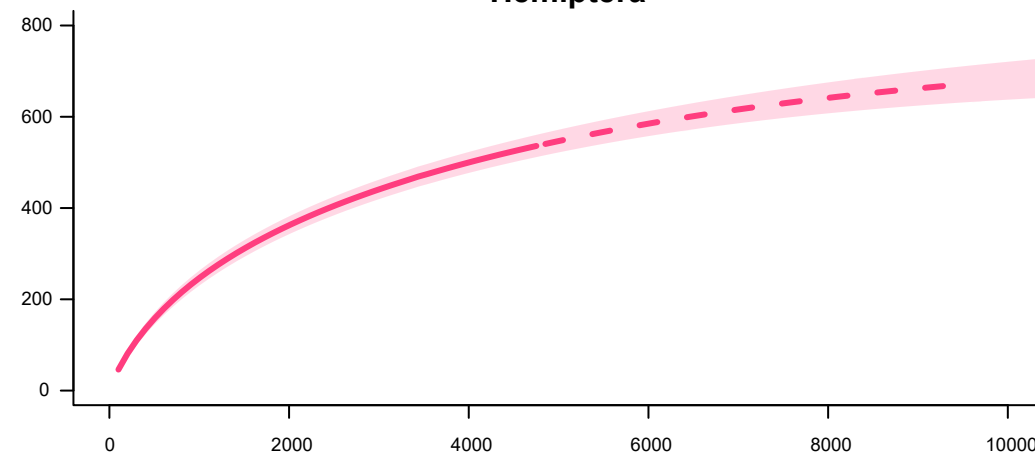

**Lepidoptera**

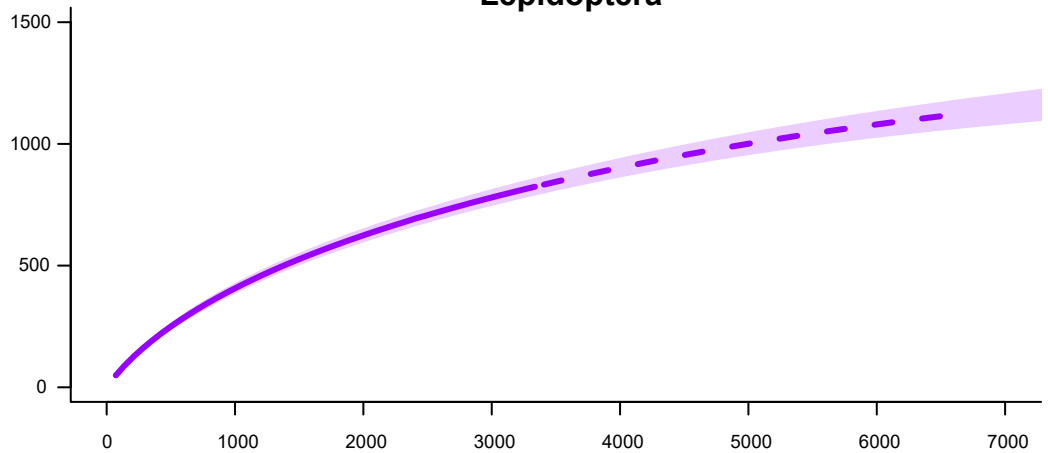

**Hymenoptera**

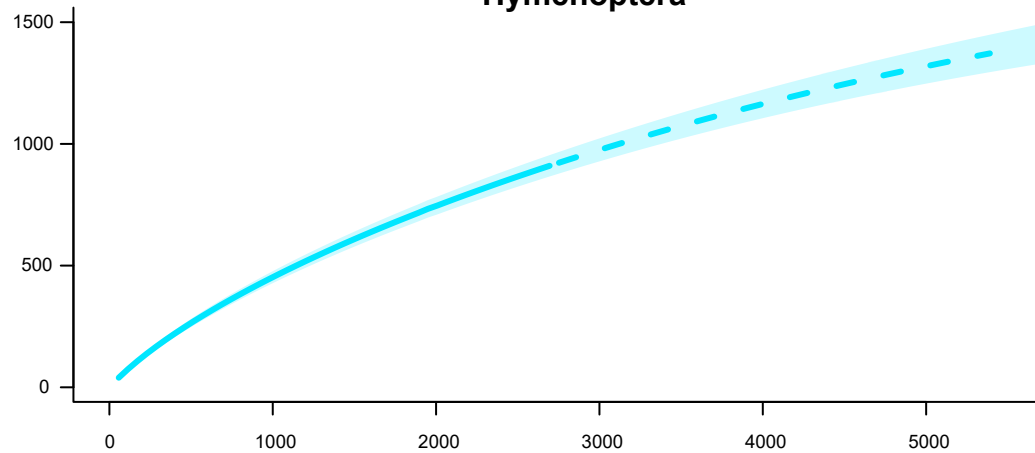

**Coleoptera**

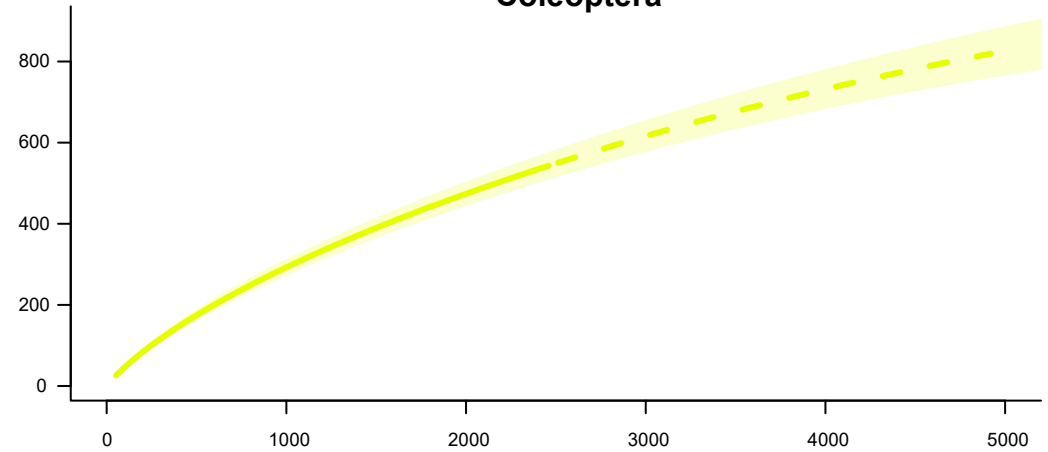

**Psocoptera**

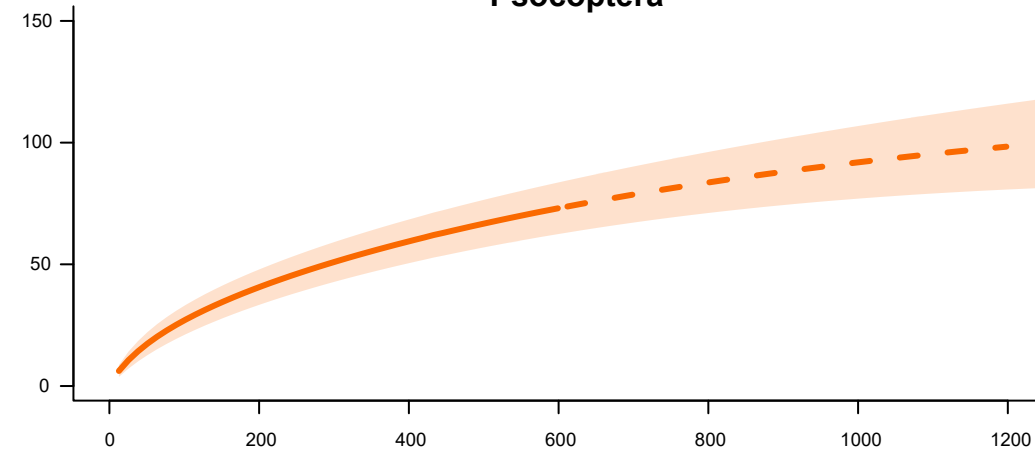

Specimens

Supplement: S5 Fig — The solid line represents the specimen-based rarefaction curve while the dashed line segment extrapolates the curve to double the observed sample size. Color shading indicates the 95% confidence interval. Note the different scales on the axes. (PDF) [file pone.0267390.s005.pdf]

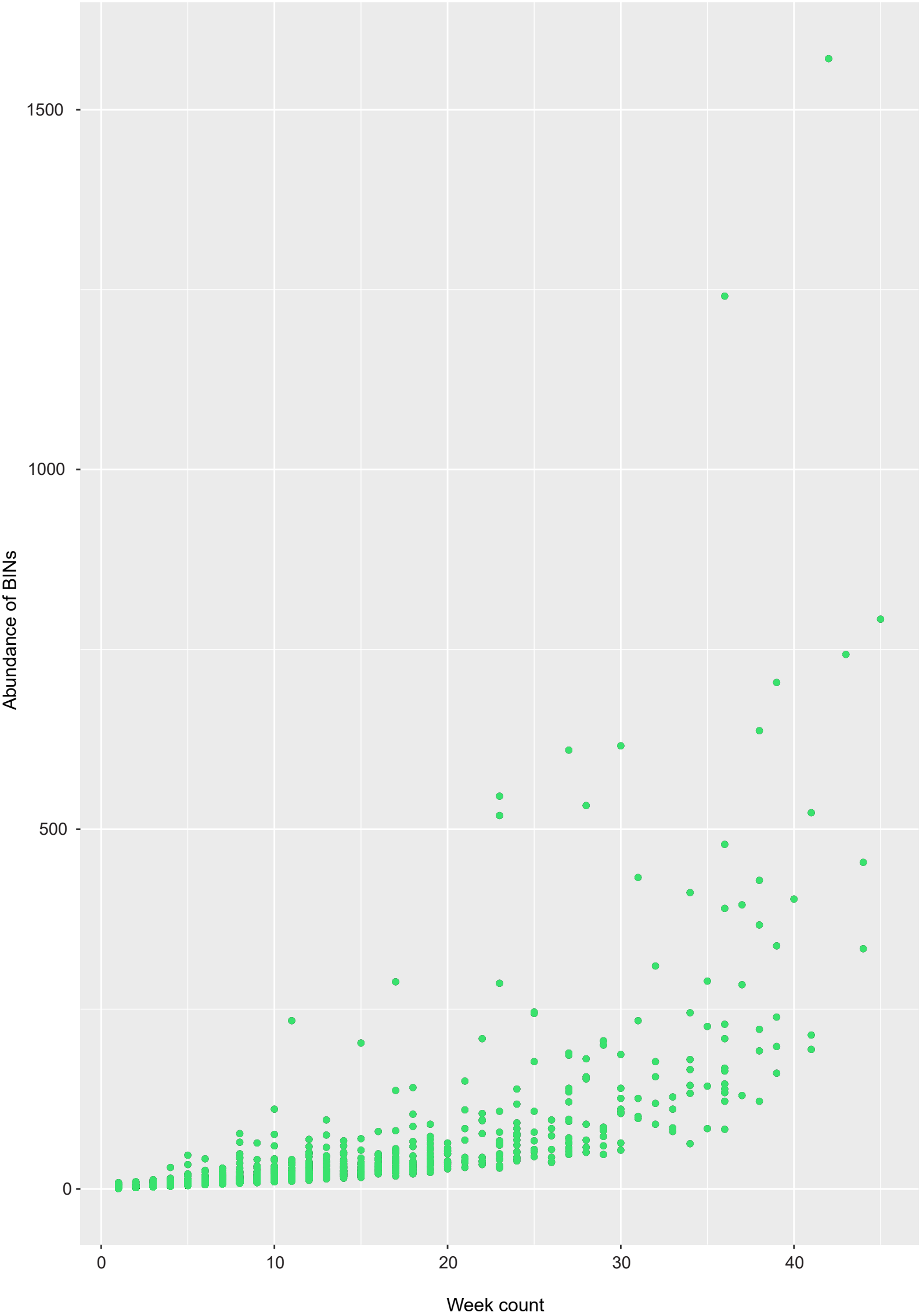

S6 Fig.

Supplement: S6 Fig — (PDF) [file pone.0267390.s006.pdf]

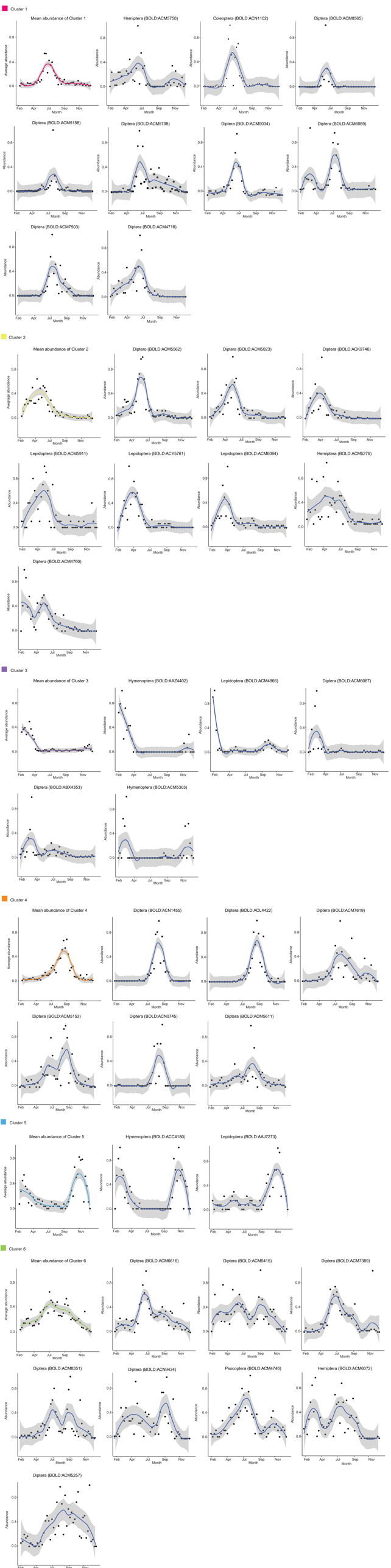

Supplement: S7 Fig — The average BIN abundance is shown in the first graph of each cluster (color-coded as in Figs. 5 and 6), and then the abundance distribution of each BIN. (PDF) [file pone.0267390.s007.pdf]
