# Supplementary material for: DNA barcodes reveal striking arthropod diversity and unveil seasonal patterns of variation in the southern Atlantic Forest
Source: PLoS One. 2022 Apr 28;17(4):e0267390. doi: 10.1371/journal.pone.0267390 (PMC9049551; doi:10.1371/journal.pone.0267390)
Supplement: S2 Fig — Neighbor-Joining tree based on the COI sequence for one representative of each BIN generated using the Kimura-2-Parameter distance model. (PDF) [file pone.0267390.s002.pdf]

BOLD TaxonID Tree

Title	:	SEARCH: Process ids(8661 ids) [SEARCH7]
Date	:	22-September-2016
Data Type	:	Nucleotide
Distance Model	:	Kimura 2 Parameter
Marker	:	COI-5P
Codon Positions	:	1st, 2nd, 3rd
Labels	:	ProcessID, Order, Family, Subfamily, BIN uri
Filters	:	Length > 200
Colorization	:	tax_order
Attachment	:	Photographs & Spreadsheet

Sequence Count	:	8660
Species count	:	211
Genus count	:	414
Family count	:	262
Unidentified	:	8442

BIN Count	:	8660

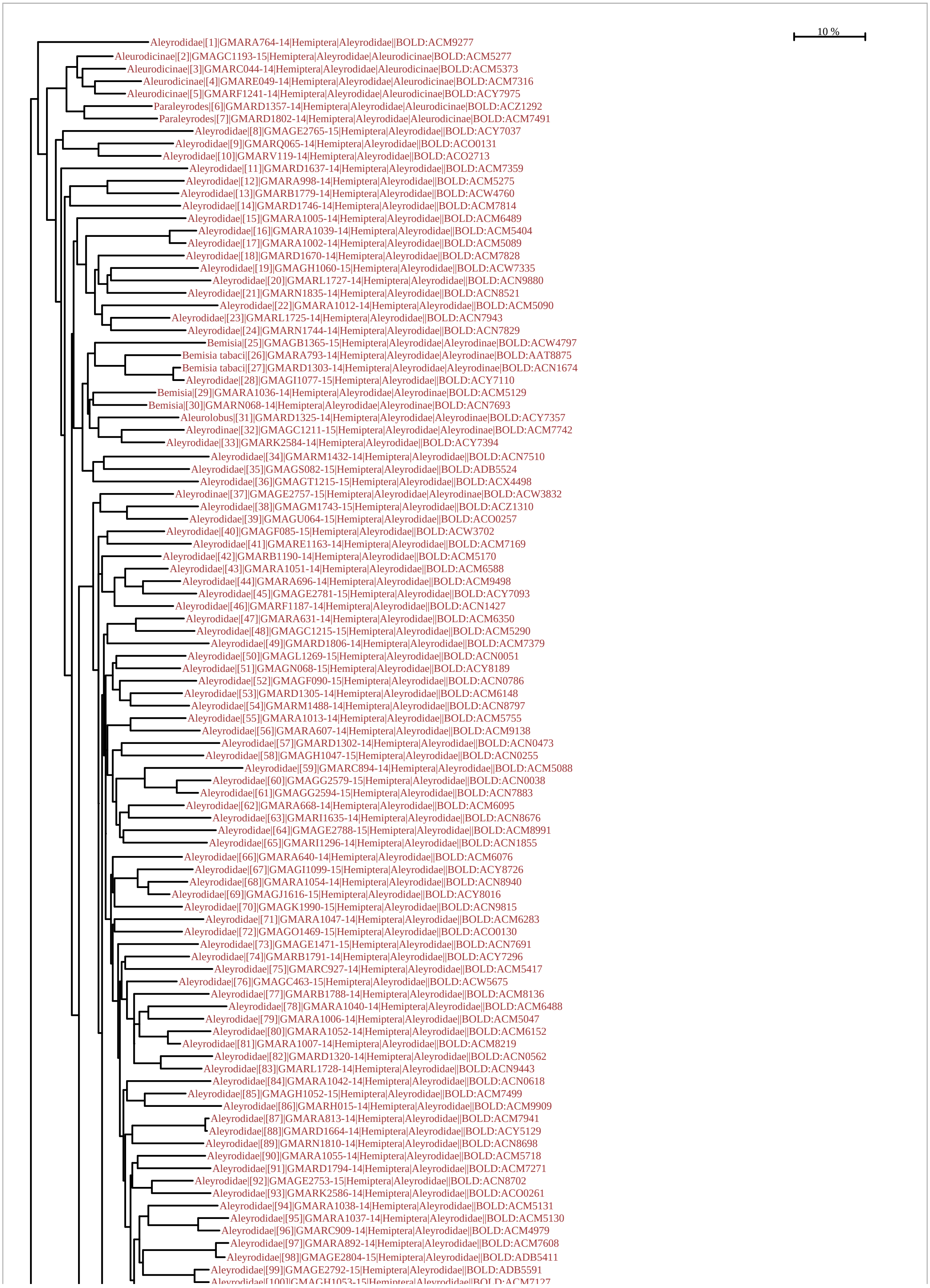
10 %
Aleyrodidae|[1]|GMARA764-14|Hemiptera|Aleyrodidae||BOLD:ACM9277
Aleurodicinae|[2]|GMAGC1193-15|Hemiptera|Aleyrodidae|Aleurodicinae|BOLD:ACM5277
Aleurodicinae|[3]|GMARC044-14|Hemiptera|Aleyrodidae|Aleurodicinae|BOLD:ACM5373
Aleurodicinae|[4]|GMARE049-14|Hemiptera|Aleyrodidae|Aleurodicinae|BOLD:ACM7316
Aleurodicinae|[5]|GMARF1241-14|Hemiptera|Aleyrodidae|Aleurodicinae|BOLD:ACY7975
Paraleyrodes|[6]|GMARD1357-14|Hemiptera|Aleyrodidae|Aleurodicinae|BOLD:ACZ1292
Paraleyrodes|[7]|GMARD1802-14|Hemiptera|Aleyrodidae|Aleurodicinae|BOLD:ACM7491
Aleyrodidae|[8]|GMAGE2765-15|Hemiptera|Aleyrodidae||BOLD:ACY7037
Aleyrodidae|[9]|GMARQ065-14|Hemiptera|Aleyrodidae||BOLD:ACO0131
Aleyrodidae|[10]|GMARV119-14|Hemiptera|Aleyrodidae||BOLD:ACO2713
Aleyrodidae|[11]|GMARD1637-14|Hemiptera|Aleyrodidae||BOLD:ACM7359
Aleyrodidae|[12]|GMARA998-14|Hemiptera|Aleyrodidae||BOLD:ACM5275
Aleyrodidae|[13]|GMARB1779-14|Hemiptera|Aleyrodidae||BOLD:ACW4760
Aleyrodidae|[14]|GMARD1746-14|Hemiptera|Aleyrodidae||BOLD:ACM7814
Aleyrodidae|[15]|GMARA1005-14|Hemiptera|Aleyrodidae||BOLD:ACM6489
Aleyrodidae|[16]|GMARA1039-14|Hemiptera|Aleyrodidae||BOLD:ACM5404
Aleyrodidae|[17]|GMARA1002-14|Hemiptera|Aleyrodidae||BOLD:ACM5089
Aleyrodidae|[18]|GMARD1670-14|Hemiptera|Aleyrodidae||BOLD:ACM7828
Aleyrodidae|[19]|GMAGH1060-15|Hemiptera|Aleyrodidae||BOLD:ACW7335
Aleyrodidae|[20]|GMARL1727-14|Hemiptera|Aleyrodidae||BOLD:ACN9880
Aleyrodidae|[21]|GMARN1835-14|Hemiptera|Aleyrodidae||BOLD:ACN8521
Aleyrodidae|[22]|GMARA1012-14|Hemiptera|Aleyrodidae||BOLD:ACM5090
Aleyrodidae|[23]|GMARL1725-14|Hemiptera|Aleyrodidae||BOLD:ACN7943
Aleyrodidae|[24]|GMARN1744-14|Hemiptera|Aleyrodidae||BOLD:ACN7829
Bemisia|[25]|GMAGB1365-15|Hemiptera|Aleyrodidae|Aleyrodinae|BOLD:ACW4797
Bemisia tabaci|[26]|GMARA793-14|Hemiptera|Aleyrodidae|Aleyrodinae|BOLD:AAT8875
Bemisia tabaci|[27]|GMARD1303-14|Hemiptera|Aleyrodidae|Aleyrodinae|BOLD:ACN1674
Aleyrodidae|[28]|GMAGI1077-15|Hemiptera|Aleyrodidae||BOLD:ACY7110
Bemisia|[29]|GMARA1036-14|Hemiptera|Aleyrodidae|Aleyrodinae|BOLD:ACM5129
Bemisia|[30]|GMARN068-14|Hemiptera|Aleyrodidae|Aleyrodinae|BOLD:ACN7693
Aleurolobus|[31]|GMARD1325-14|Hemiptera|Aleyrodidae|Aleyrodinae|BOLD:ACY7357
Aleyrodinae|[32]|GMAGC1211-15|Hemiptera|Aleyrodidae|Aleyrodinae|BOLD:ACM7742
Aleyrodidae|[33]|GMARK2584-14|Hemiptera|Aleyrodidae||BOLD:ACY7394
Aleyrodidae|[34]|GMARM1432-14|Hemiptera|Aleyrodidae||BOLD:ACN7510
Aleyrodidae|[35]|GMAGS082-15|Hemiptera|Aleyrodidae||BOLD:ADB5524
Aleyrodidae|[36]|GMAGT1215-15|Hemiptera|Aleyrodidae||BOLD:ACX4498
Aleyrodinae|[37]|GMAGE2757-15|Hemiptera|Aleyrodidae|Aleyrodinae|BOLD:ACW3832
Aleyrodidae|[38]|GMAGM1743-15|Hemiptera|Aleyrodidae||BOLD:ACZ1310
Aleyrodidae|[39]|GMAGU064-15|Hemiptera|Aleyrodidae||BOLD:ACO0257
Aleyrodidae|[40]|GMAGF085-15|Hemiptera|Aleyrodidae||BOLD:ACW3702
Aleyrodidae|[41]|GMARE1163-14|Hemiptera|Aleyrodidae||BOLD:ACM7169
Aleyrodidae|[42]|GMARB1190-14|Hemiptera|Aleyrodidae||BOLD:ACM5170
Aleyrodidae|[43]|GMARA1051-14|Hemiptera|Aleyrodidae||BOLD:ACM6588
Aleyrodidae|[44]|GMARA696-14|Hemiptera|Aleyrodidae||BOLD:ACM9498
Aleyrodidae|[45]|GMAGE2781-15|Hemiptera|Aleyrodidae||BOLD:ACY7093
Aleyrodidae|[46]|GMARF1187-14|Hemiptera|Aleyrodidae||BOLD:ACN1427
Aleyrodidae|[47]|GMARA631-14|Hemiptera|Aleyrodidae||BOLD:ACM6350
Aleyrodidae|[48]|GMAGC1215-15|Hemiptera|Aleyrodidae||BOLD:ACM5290
Aleyrodidae|[49]|GMARD1806-14|Hemiptera|Aleyrodidae||BOLD:ACM7379
Aleyrodidae|[50]|GMAGL1269-15|Hemiptera|Aleyrodidae||BOLD:ACN0051
Aleyrodidae|[51]|GMAGN068-15|Hemiptera|Aleyrodidae||BOLD:ACY8189
Aleyrodidae|[52]|GMAGF090-15|Hemiptera|Aleyrodidae||BOLD:ACN0786
Aleyrodidae|[53]|GMARD1305-14|Hemiptera|Aleyrodidae||BOLD:ACM6148
Aleyrodidae|[54]|GMARM1488-14|Hemiptera|Aleyrodidae||BOLD:ACN8797
Aleyrodidae|[55]|GMARA1013-14|Hemiptera|Aleyrodidae||BOLD:ACM5755
Aleyrodidae|[56]|GMARA607-14|Hemiptera|Aleyrodidae||BOLD:ACM9138
Aleyrodidae|[57]|GMARD1302-14|Hemiptera|Aleyrodidae||BOLD:ACN0473
Aleyrodidae|[58]|GMAGH1047-15|Hemiptera|Aleyrodidae||BOLD:ACN0255
Aleyrodidae|[59]|GMARC894-14|Hemiptera|Aleyrodidae||BOLD:ACM5088
Aleyrodidae|[60]|GMAGG2579-15|Hemiptera|Aleyrodidae||BOLD:ACN0038
Aleyrodidae|[61]|GMAGG2594-15|Hemiptera|Aleyrodidae||BOLD:ACN7883
Aleyrodidae|[62]|GMARA668-14|Hemiptera|Aleyrodidae||BOLD:ACM6095
Aleyrodidae|[63]|GMARI1635-14|Hemiptera|Aleyrodidae||BOLD:ACN8676
Aleyrodidae|[64]|GMAGE2788-15|Hemiptera|Aleyrodidae||BOLD:ACM8991
Aleyrodidae|[65]|GMARI1296-14|Hemiptera|Aleyrodidae||BOLD:ACN1855
Aleyrodidae|[66]|GMARA640-14|Hemiptera|Aleyrodidae||BOLD:ACM6076
Aleyrodidae|[67]|GMAGI1099-15|Hemiptera|Aleyrodidae||BOLD:ACY8726
Aleyrodidae|[68]|GMARA1054-14|Hemiptera|Aleyrodidae||BOLD:ACN8940
Aleyrodidae|[69]|GMAGJ1616-15|Hemiptera|Aleyrodidae||BOLD:ACY8016
Aleyrodidae|[70]|GMAGK1990-15|Hemiptera|Aleyrodidae||BOLD:ACN9815
Aleyrodidae|[71]|GMARA1047-14|Hemiptera|Aleyrodidae||BOLD:ACM6283
Aleyrodidae|[72]|GMAGO1469-15|Hemiptera|Aleyrodidae||BOLD:ACO0130
Aleyrodidae|[73]|GMAGE1471-15|Hemiptera|Aleyrodidae||BOLD:ACN7691
Aleyrodidae|[74]|GMARB1791-14|Hemiptera|Aleyrodidae||BOLD:ACY7296
Aleyrodidae|[75]|GMARC927-14|Hemiptera|Aleyrodidae||BOLD:ACM5417
Aleyrodidae|[76]|GMAGC463-15|Hemiptera|Aleyrodidae||BOLD:ACW5675
Aleyrodidae|[77]|GMARB1788-14|Hemiptera|Aleyrodidae||BOLD:ACM8136
Aleyrodidae|[78]|GMARA1040-14|Hemiptera|Aleyrodidae||BOLD:ACM6488
Aleyrodidae|[79]|GMARA1006-14|Hemiptera|Aleyrodidae||BOLD:ACM5047
Aleyrodidae|[80]|GMARA1052-14|Hemiptera|Aleyrodidae||BOLD:ACM6152
Aleyrodidae|[81]|GMARA1007-14|Hemiptera|Aleyrodidae||BOLD:ACM8219
Aleyrodidae|[82]|GMARD1320-14|Hemiptera|Aleyrodidae||BOLD:ACN0562
Aleyrodidae|[83]|GMARL1728-14|Hemiptera|Aleyrodidae||BOLD:ACN9443
Aleyrodidae|[84]|GMARA1042-14|Hemiptera|Aleyrodidae||BOLD:ACN0618
Aleyrodidae|[85]|GMAGH1052-15|Hemiptera|Aleyrodidae||BOLD:ACM7499
Aleyrodidae|[86]|GMARH015-14|Hemiptera|Aleyrodidae||BOLD:ACM9909
Aleyrodidae|[87]|GMARA813-14|Hemiptera|Aleyrodidae||BOLD:ACM7941
Aleyrodidae|[88]|GMARD1664-14|Hemiptera|Aleyrodidae||BOLD:ACY5129
Aleyrodidae|[89]|GMARN1810-14|Hemiptera|Aleyrodidae||BOLD:ACN8698
Aleyrodidae|[90]|GMARA1055-14|Hemiptera|Aleyrodidae||BOLD:ACM5718
Aleyrodidae|[91]|GMARD1794-14|Hemiptera|Aleyrodidae||BOLD:ACM7271
Aleyrodidae|[92]|GMAGE2753-15|Hemiptera|Aleyrodidae||BOLD:ACN8702
Aleyrodidae|[93]|GMARK2586-14|Hemiptera|Aleyrodidae||BOLD:ACO0261
Aleyrodidae|[94]|GMARA1038-14|Hemiptera|Aleyrodidae||BOLD:ACM5131
Aleyrodidae|[95]|GMARA1037-14|Hemiptera|Aleyrodidae||BOLD:ACM5130
Aleyrodidae|[96]|GMARC909-14|Hemiptera|Aleyrodidae||BOLD:ACM4979
Aleyrodidae|[97]|GMARA892-14|Hemiptera|Aleyrodidae||BOLD:ACM7608
Aleyrodidae|[98]|GMAGE2804-15|Hemiptera|Aleyrodidae||BOLD:ADB5411
Aleyrodidae|[99]|GMAGE2792-15|Hemiptera|Aleyrodidae||BOLD:ADB5591
Aleyrodidae|[100]|GMAGH1053-15|Hemiptera|Aleyrodidae||BOLD:ACM7127

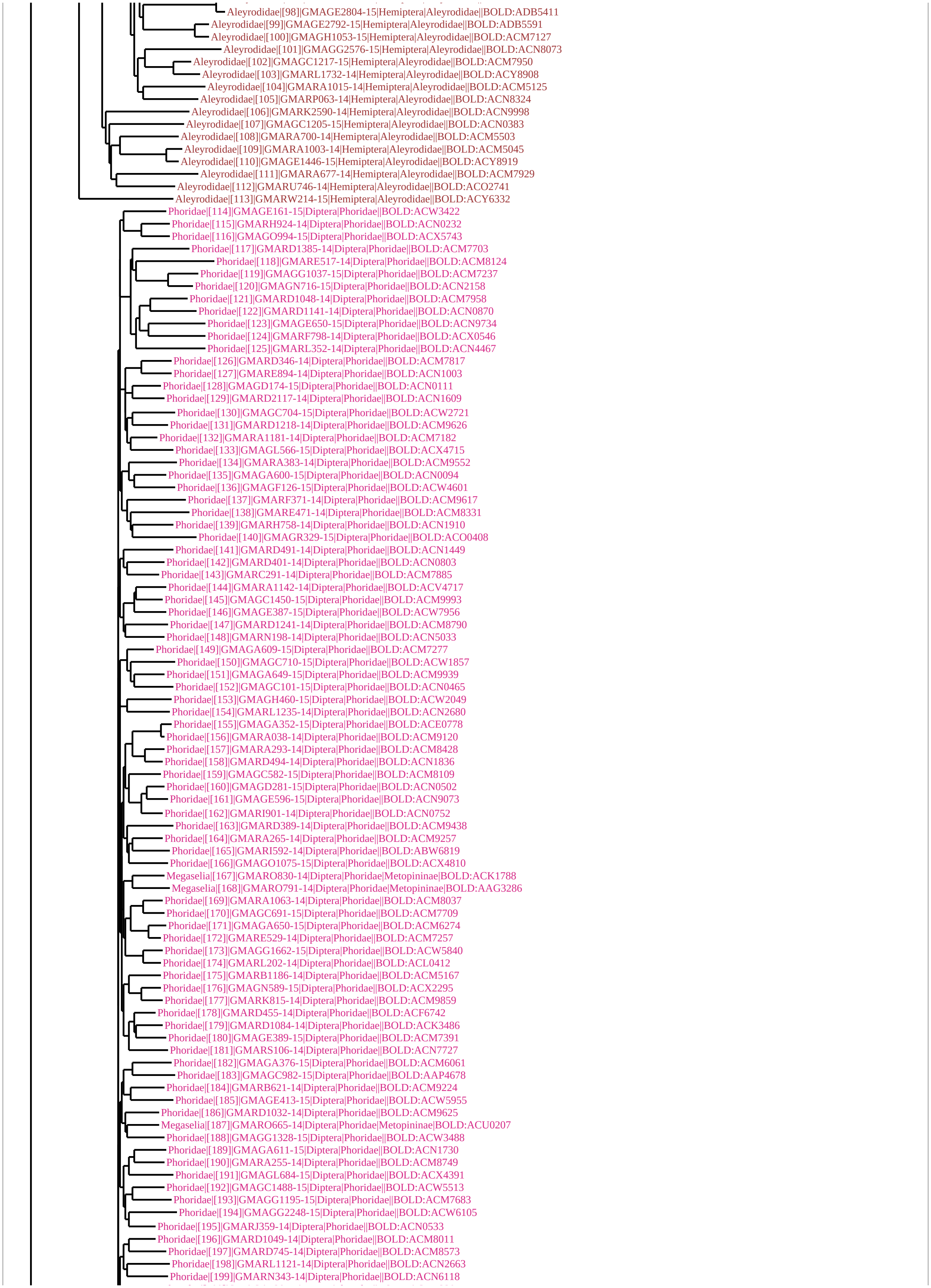
Aleyrodidae|[98]|GMAGE2804-15|Hemiptera|Aleyrodidae||BOLD:ADB5411
Aleyrodidae|[99]|GMAGE2792-15|Hemiptera|Aleyrodidae||BOLD:ADB5591
Aleyrodidae|[100]|GMAGH1053-15|Hemiptera|Aleyrodidae||BOLD:ACM7127
Aleyrodidae|[101]|GMAGG2576-15|Hemiptera|Aleyrodidae||BOLD:ACN8073
Aleyrodidae|[102]|GMAGC1217-15|Hemiptera|Aleyrodidae||BOLD:ACM7950
Aleyrodidae|[103]|GMARL1732-14|Hemiptera|Aleyrodidae||BOLD:ACY8908
Aleyrodidae|[104]|GMARA1015-14|Hemiptera|Aleyrodidae||BOLD:ACM5125
Aleyrodidae|[105]|GMARP063-14|Hemiptera|Aleyrodidae||BOLD:ACN8324
Aleyrodidae|[106]|GMARK2590-14|Hemiptera|Aleyrodidae||BOLD:ACN9998
Aleyrodidae|[107]|GMAGC1205-15|Hemiptera|Aleyrodidae||BOLD:ACN0383
Aleyrodidae|[108]|GMARA700-14|Hemiptera|Aleyrodidae||BOLD:ACM5503
Aleyrodidae|[109]|GMARA1003-14|Hemiptera|Aleyrodidae||BOLD:ACM5045
Aleyrodidae|[110]|GMAGE1446-15|Hemiptera|Aleyrodidae||BOLD:ACY8919
Aleyrodidae|[111]|GMARA677-14|Hemiptera|Aleyrodidae||BOLD:ACM7929
Aleyrodidae|[112]|GMARU746-14|Hemiptera|Aleyrodidae||BOLD:ACO2741
Aleyrodidae|[113]|GMARW214-15|Hemiptera|Aleyrodidae||BOLD:ACY6332
Phoridae|[114]|GMAGE161-15|Diptera|Phoridae||BOLD:ACW3422
Phoridae|[115]|GMARH924-14|Diptera|Phoridae||BOLD:ACN0232
Phoridae|[116]|GMAGO994-15|Diptera|Phoridae||BOLD:ACX5743
Phoridae|[117]|GMARD1385-14|Diptera|Phoridae||BOLD:ACM7703
Phoridae|[118]|GMARE517-14|Diptera|Phoridae||BOLD:ACM8124
Phoridae|[119]|GMAGG1037-15|Diptera|Phoridae||BOLD:ACM7237
Phoridae|[120]|GMAGN716-15|Diptera|Phoridae||BOLD:ACN2158
Phoridae|[121]|GMARD1048-14|Diptera|Phoridae||BOLD:ACM7958
Phoridae|[122]|GMARD1141-14|Diptera|Phoridae||BOLD:ACN0870
Phoridae|[123]|GMAGE650-15|Diptera|Phoridae||BOLD:ACN9734
Phoridae|[124]|GMARF798-14|Diptera|Phoridae||BOLD:ACX0546
Phoridae|[125]|GMARL352-14|Diptera|Phoridae||BOLD:ACN4467
Phoridae|[126]|GMARD346-14|Diptera|Phoridae||BOLD:ACM7817
Phoridae|[127]|GMARE894-14|Diptera|Phoridae||BOLD:ACN1003
Phoridae|[128]|GMAGD174-15|Diptera|Phoridae||BOLD:ACN0111
Phoridae|[129]|GMARD2117-14|Diptera|Phoridae||BOLD:ACN1609
Phoridae|[130]|GMAGC704-15|Diptera|Phoridae||BOLD:ACW2721
Phoridae|[131]|GMARD1218-14|Diptera|Phoridae||BOLD:ACM9626
Phoridae|[132]|GMARA1181-14|Diptera|Phoridae||BOLD:ACM7182
Phoridae|[133]|GMAGL566-15|Diptera|Phoridae||BOLD:ACX4715
Phoridae|[134]|GMARA383-14|Diptera|Phoridae||BOLD:ACM9552
Phoridae|[135]|GMAGA600-15|Diptera|Phoridae||BOLD:ACN0094
Phoridae|[136]|GMAGF126-15|Diptera|Phoridae||BOLD:ACW4601
Phoridae|[137]|GMARF371-14|Diptera|Phoridae||BOLD:ACM9617
Phoridae|[138]|GMARE471-14|Diptera|Phoridae||BOLD:ACM8331
Phoridae|[139]|GMARH758-14|Diptera|Phoridae||BOLD:ACN1910
Phoridae|[140]|GMAGR329-15|Diptera|Phoridae||BOLD:ACO0408
Phoridae|[141]|GMARD491-14|Diptera|Phoridae||BOLD:ACN1449
Phoridae|[142]|GMARD401-14|Diptera|Phoridae||BOLD:ACN0803
Phoridae|[143]|GMARC291-14|Diptera|Phoridae||BOLD:ACM7885
Phoridae|[144]|GMARA1142-14|Diptera|Phoridae||BOLD:ACV4717
Phoridae|[145]|GMAGC1450-15|Diptera|Phoridae||BOLD:ACM9993
Phoridae|[146]|GMAGE387-15|Diptera|Phoridae||BOLD:ACW7956
Phoridae|[147]|GMARD1241-14|Diptera|Phoridae||BOLD:ACM8790
Phoridae|[148]|GMARN198-14|Diptera|Phoridae||BOLD:ACN5033
Phoridae|[149]|GMAGA609-15|Diptera|Phoridae||BOLD:ACM7277
Phoridae|[150]|GMAGC710-15|Diptera|Phoridae||BOLD:ACW1857
Phoridae|[151]|GMAGA649-15|Diptera|Phoridae||BOLD:ACM9939
Phoridae|[152]|GMAGC101-15|Diptera|Phoridae||BOLD:ACN0465
Phoridae|[153]|GMAGH460-15|Diptera|Phoridae||BOLD:ACW2049
Phoridae|[154]|GMARL1235-14|Diptera|Phoridae||BOLD:ACN2680
Phoridae|[155]|GMAGA352-15|Diptera|Phoridae||BOLD:ACE0778
Phoridae|[156]|GMARA038-14|Diptera|Phoridae||BOLD:ACM9120
Phoridae|[157]|GMARA293-14|Diptera|Phoridae||BOLD:ACM8428
Phoridae|[158]|GMARD494-14|Diptera|Phoridae||BOLD:ACN1836
Phoridae|[159]|GMAGC582-15|Diptera|Phoridae||BOLD:ACM8109
Phoridae|[160]|GMAGD281-15|Diptera|Phoridae||BOLD:ACN0502
Phoridae|[161]|GMAGE596-15|Diptera|Phoridae||BOLD:ACN9073
Phoridae|[162]|GMARI901-14|Diptera|Phoridae||BOLD:ACN0752
Phoridae|[163]|GMARD389-14|Diptera|Phoridae||BOLD:ACM9438
Phoridae|[164]|GMARA265-14|Diptera|Phoridae||BOLD:ACM9257
Phoridae|[165]|GMARI592-14|Diptera|Phoridae||BOLD:ABW6819
Phoridae|[166]|GMAGO1075-15|Diptera|Phoridae||BOLD:ACX4810
Megaselia|[167]|GMARO830-14|Diptera|Phoridae|Metopininae|BOLD:ACK1788
Megaselia|[168]|GMARO791-14|Diptera|Phoridae|Metopininae|BOLD:AAG3286
Phoridae|[169]|GMARA1063-14|Diptera|Phoridae||BOLD:ACM8037
Phoridae|[170]|GMAGC691-15|Diptera|Phoridae||BOLD:ACM7709
Phoridae|[171]|GMAGA650-15|Diptera|Phoridae||BOLD:ACM6274
Phoridae|[172]|GMARE529-14|Diptera|Phoridae||BOLD:ACM7257
Phoridae|[173]|GMAGG1662-15|Diptera|Phoridae||BOLD:ACW5840
Phoridae|[174]|GMARL202-14|Diptera|Phoridae||BOLD:ACL0412
Phoridae|[175]|GMARB1186-14|Diptera|Phoridae||BOLD:ACM5167
Phoridae|[176]|GMAGN589-15|Diptera|Phoridae||BOLD:ACX2295
Phoridae|[177]|GMARK815-14|Diptera|Phoridae||BOLD:ACM9859
Phoridae|[178]|GMARD455-14|Diptera|Phoridae||BOLD:ACF6742
Phoridae|[179]|GMARD1084-14|Diptera|Phoridae||BOLD:ACK3486
Phoridae|[180]|GMAGE389-15|Diptera|Phoridae||BOLD:ACM7391
Phoridae|[181]|GMARS106-14|Diptera|Phoridae||BOLD:ACN7727
Phoridae|[182]|GMAGA376-15|Diptera|Phoridae||BOLD:ACM6061
Phoridae|[183]|GMAGC982-15|Diptera|Phoridae||BOLD:AAP4678
Phoridae|[184]|GMARB621-14|Diptera|Phoridae||BOLD:ACM9224
Phoridae|[185]|GMAGE413-15|Diptera|Phoridae||BOLD:ACW5955
Phoridae|[186]|GMARD1032-14|Diptera|Phoridae||BOLD:ACM9625
Megaselia|[187]|GMARO665-14|Diptera|Phoridae|Metopininae|BOLD:ACU0207
Phoridae|[188]|GMAGG1328-15|Diptera|Phoridae||BOLD:ACW3488
Phoridae|[189]|GMAGA611-15|Diptera|Phoridae||BOLD:ACN1730
Phoridae|[190]|GMARA255-14|Diptera|Phoridae||BOLD:ACM8749
Phoridae|[191]|GMAGL684-15|Diptera|Phoridae||BOLD:ACX4391
Phoridae|[192]|GMAGC1488-15|Diptera|Phoridae||BOLD:ACW5513
Phoridae|[193]|GMAGG1195-15|Diptera|Phoridae||BOLD:ACM7683
Phoridae|[194]|GMAGG2248-15|Diptera|Phoridae||BOLD:ACW6105
Phoridae|[195]|GMARJ359-14|Diptera|Phoridae||BOLD:ACN0533
Phoridae|[196]|GMARD1049-14|Diptera|Phoridae||BOLD:ACM8011
Phoridae|[197]|GMARD745-14|Diptera|Phoridae||BOLD:ACM8573
Phoridae|[198]|GMARL1121-14|Diptera|Phoridae||BOLD:ACN2663
Phoridae|[199]|GMARN343-14|Diptera|Phoridae||BOLD:ACN6118

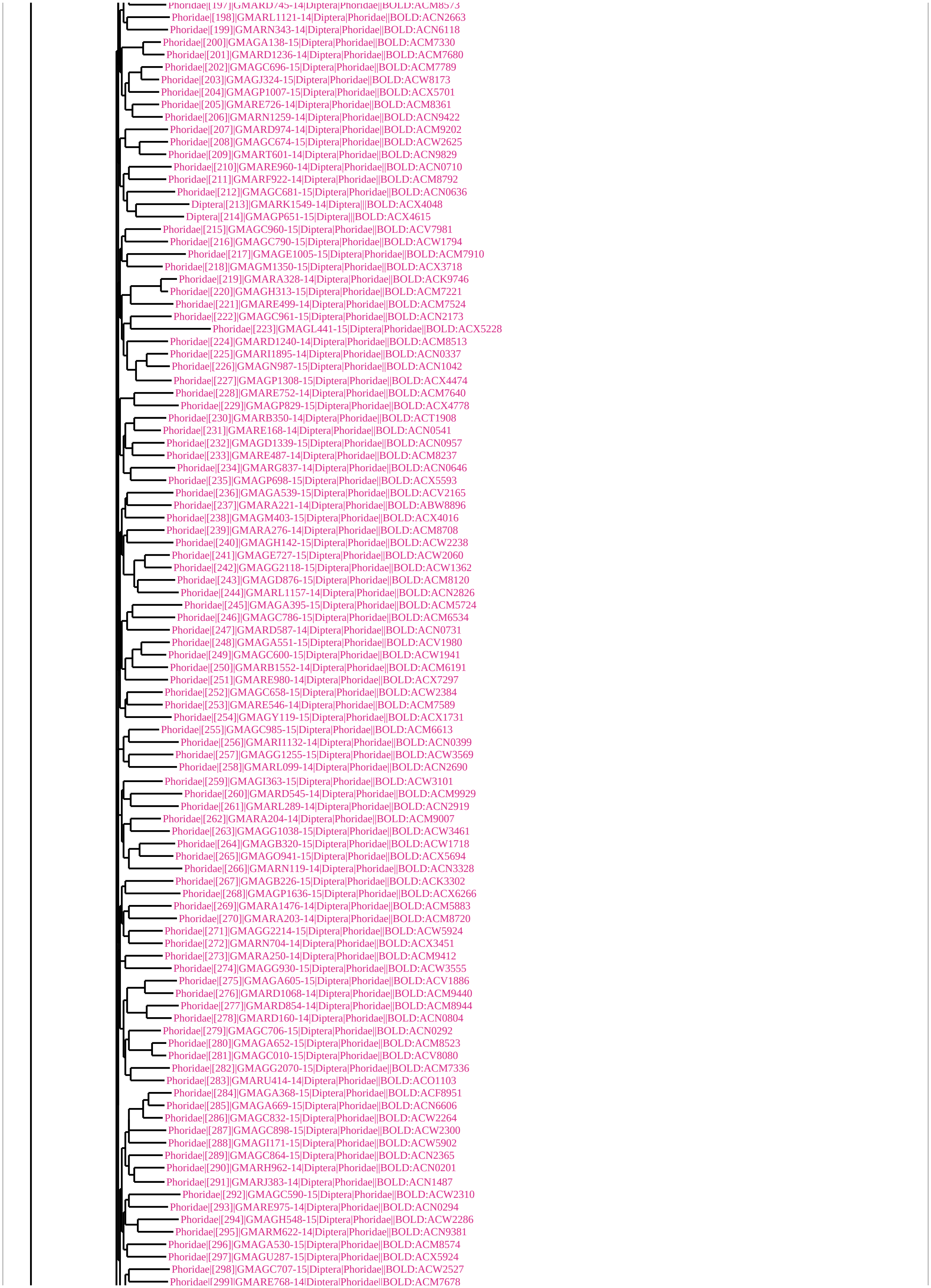
Phoridae|[197]|GMARD745-14|Diptera|Phoridae||BOLD:ACM8573
Phoridae|[198]|GMARL1121-14|Diptera|Phoridae||BOLD:ACN2663
Phoridae|[199]|GMARN343-14|Diptera|Phoridae||BOLD:ACN6118
Phoridae|[200]|GMAGA138-15|Diptera|Phoridae||BOLD:ACM7330
Phoridae|[201]|GMARD1236-14|Diptera|Phoridae||BOLD:ACM7680
Phoridae|[202]|GMAGC696-15|Diptera|Phoridae||BOLD:ACM7789
Phoridae|[203]|GMAGJ324-15|Diptera|Phoridae||BOLD:ACW8173
Phoridae|[204]|GMAGP1007-15|Diptera|Phoridae||BOLD:ACX5701
Phoridae|[205]|GMARE726-14|Diptera|Phoridae||BOLD:ACM8361
Phoridae|[206]|GMARN1259-14|Diptera|Phoridae||BOLD:ACN9422
Phoridae|[207]|GMARD974-14|Diptera|Phoridae||BOLD:ACM9202
Phoridae|[208]|GMAGC674-15|Diptera|Phoridae||BOLD:ACW2625
Phoridae|[209]|GMART601-14|Diptera|Phoridae||BOLD:ACN9829
Phoridae|[210]|GMARE960-14|Diptera|Phoridae||BOLD:ACN0710
Phoridae|[211]|GMARF922-14|Diptera|Phoridae||BOLD:ACM8792
Phoridae|[212]|GMAGC681-15|Diptera|Phoridae||BOLD:ACN0636
Diptera|[213]|GMARK1549-14|Diptera|||BOLD:ACX4048
Diptera|[214]|GMAGP651-15|Diptera|||BOLD:ACX4615
Phoridae|[215]|GMAGC960-15|Diptera|Phoridae||BOLD:ACV7981
Phoridae|[216]|GMAGC790-15|Diptera|Phoridae||BOLD:ACW1794
Phoridae|[217]|GMAGE1005-15|Diptera|Phoridae||BOLD:ACM7910
Phoridae|[218]|GMAGM1350-15|Diptera|Phoridae||BOLD:ACX3718
Phoridae|[219]|GMARA328-14|Diptera|Phoridae||BOLD:ACK9746
Phoridae|[220]|GMAGH313-15|Diptera|Phoridae||BOLD:ACM7221
Phoridae|[221]|GMARE499-14|Diptera|Phoridae||BOLD:ACM7524
Phoridae|[222]|GMAGC961-15|Diptera|Phoridae||BOLD:ACN2173
Phoridae|[223]|GMAGL441-15|Diptera|Phoridae||BOLD:ACX5228
Phoridae|[224]|GMARD1240-14|Diptera|Phoridae||BOLD:ACM8513
Phoridae|[225]|GMARI1895-14|Diptera|Phoridae||BOLD:ACN0337
Phoridae|[226]|GMAGN987-15|Diptera|Phoridae||BOLD:ACN1042
Phoridae|[227]|GMAGP1308-15|Diptera|Phoridae||BOLD:ACX4474
Phoridae|[228]|GMARE752-14|Diptera|Phoridae||BOLD:ACM7640
Phoridae|[229]|GMAGP829-15|Diptera|Phoridae||BOLD:ACX4778
Phoridae|[230]|GMARB350-14|Diptera|Phoridae||BOLD:ACT1908
Phoridae|[231]|GMARE168-14|Diptera|Phoridae||BOLD:ACN0541
Phoridae|[232]|GMAGD1339-15|Diptera|Phoridae||BOLD:ACN0957
Phoridae|[233]|GMARE487-14|Diptera|Phoridae||BOLD:ACM8237
Phoridae|[234]|GMARG837-14|Diptera|Phoridae||BOLD:ACN0646
Phoridae|[235]|GMAGP698-15|Diptera|Phoridae||BOLD:ACX5593
Phoridae|[236]|GMAGA539-15|Diptera|Phoridae||BOLD:ACV2165
Phoridae|[237]|GMARA221-14|Diptera|Phoridae||BOLD:ABW8896
Phoridae|[238]|GMAGM403-15|Diptera|Phoridae||BOLD:ACX4016
Phoridae|[239]|GMARA276-14|Diptera|Phoridae||BOLD:ACM8708
Phoridae|[240]|GMAGH142-15|Diptera|Phoridae||BOLD:ACW2238
Phoridae|[241]|GMAGE727-15|Diptera|Phoridae||BOLD:ACW2060
Phoridae|[242]|GMAGG2118-15|Diptera|Phoridae||BOLD:ACW1362
Phoridae|[243]|GMAGD876-15|Diptera|Phoridae||BOLD:ACM8120
Phoridae|[244]|GMARL1157-14|Diptera|Phoridae||BOLD:ACN2826
Phoridae|[245]|GMAGA395-15|Diptera|Phoridae||BOLD:ACM5724
Phoridae|[246]|GMAGC786-15|Diptera|Phoridae||BOLD:ACM6534
Phoridae|[247]|GMARD587-14|Diptera|Phoridae||BOLD:ACN0731
Phoridae|[248]|GMAGA551-15|Diptera|Phoridae||BOLD:ACV1980
Phoridae|[249]|GMAGC600-15|Diptera|Phoridae||BOLD:ACW1941
Phoridae|[250]|GMARB1552-14|Diptera|Phoridae||BOLD:ACM6191
Phoridae|[251]|GMARE980-14|Diptera|Phoridae||BOLD:ACX7297
Phoridae|[252]|GMAGC658-15|Diptera|Phoridae||BOLD:ACW2384
Phoridae|[253]|GMARE546-14|Diptera|Phoridae||BOLD:ACM7589
Phoridae|[254]|GMAGY119-15|Diptera|Phoridae||BOLD:ACX1731
Phoridae|[255]|GMAGC985-15|Diptera|Phoridae||BOLD:ACM6613
Phoridae|[256]|GMARI1132-14|Diptera|Phoridae||BOLD:ACN0399
Phoridae|[257]|GMAGG1255-15|Diptera|Phoridae||BOLD:ACW3569
Phoridae|[258]|GMARL099-14|Diptera|Phoridae||BOLD:ACN2690
Phoridae|[259]|GMAGI363-15|Diptera|Phoridae||BOLD:ACW3101
Phoridae|[260]|GMARD545-14|Diptera|Phoridae||BOLD:ACM9929
Phoridae|[261]|GMARL289-14|Diptera|Phoridae||BOLD:ACN2919
Phoridae|[262]|GMARA204-14|Diptera|Phoridae||BOLD:ACM9007
Phoridae|[263]|GMAGG1038-15|Diptera|Phoridae||BOLD:ACW3461
Phoridae|[264]|GMAGB320-15|Diptera|Phoridae||BOLD:ACW1718
Phoridae|[265]|GMAGO941-15|Diptera|Phoridae||BOLD:ACX5694
Phoridae|[266]|GMARN119-14|Diptera|Phoridae||BOLD:ACN3328
Phoridae|[267]|GMAGB226-15|Diptera|Phoridae||BOLD:ACK3302
Phoridae|[268]|GMAGP1636-15|Diptera|Phoridae||BOLD:ACX6266
Phoridae|[269]|GMARA1476-14|Diptera|Phoridae||BOLD:ACM5883
Phoridae|[270]|GMARA203-14|Diptera|Phoridae||BOLD:ACM8720
Phoridae|[271]|GMAGG2214-15|Diptera|Phoridae||BOLD:ACW5924
Phoridae|[272]|GMARN704-14|Diptera|Phoridae||BOLD:ACX3451
Phoridae|[273]|GMARA250-14|Diptera|Phoridae||BOLD:ACM9412
Phoridae|[274]|GMAGG930-15|Diptera|Phoridae||BOLD:ACW3555
Phoridae|[275]|GMAGA605-15|Diptera|Phoridae||BOLD:ACV1886
Phoridae|[276]|GMARD1068-14|Diptera|Phoridae||BOLD:ACM9440
Phoridae|[277]|GMARD854-14|Diptera|Phoridae||BOLD:ACM8944
Phoridae|[278]|GMARD160-14|Diptera|Phoridae||BOLD:ACN0804
Phoridae|[279]|GMAGC706-15|Diptera|Phoridae||BOLD:ACN0292
Phoridae|[280]|GMAGA652-15|Diptera|Phoridae||BOLD:ACM8523
Phoridae|[281]|GMAGC010-15|Diptera|Phoridae||BOLD:ACV8080
Phoridae|[282]|GMAGG2070-15|Diptera|Phoridae||BOLD:ACM7336
Phoridae|[283]|GMARU414-14|Diptera|Phoridae||BOLD:ACO1103
Phoridae|[284]|GMAGA368-15|Diptera|Phoridae||BOLD:ACF8951
Phoridae|[285]|GMAGA669-15|Diptera|Phoridae||BOLD:ACN6606
Phoridae|[286]|GMAGC832-15|Diptera|Phoridae||BOLD:ACW2264
Phoridae|[287]|GMAGC898-15|Diptera|Phoridae||BOLD:ACW2300
Phoridae|[288]|GMAGI171-15|Diptera|Phoridae||BOLD:ACW5902
Phoridae|[289]|GMAGC864-15|Diptera|Phoridae||BOLD:ACN2365
Phoridae|[290]|GMARH962-14|Diptera|Phoridae||BOLD:ACN0201
Phoridae|[291]|GMARJ383-14|Diptera|Phoridae||BOLD:ACN1487
Phoridae|[292]|GMAGC590-15|Diptera|Phoridae||BOLD:ACW2310
Phoridae|[293]|GMARE975-14|Diptera|Phoridae||BOLD:ACN0294
Phoridae|[294]|GMAGH548-15|Diptera|Phoridae||BOLD:ACW2286
Phoridae|[295]|GMARM622-14|Diptera|Phoridae||BOLD:ACN9381
Phoridae|[296]|GMAGA530-15|Diptera|Phoridae||BOLD:ACM8574
Phoridae|[297]|GMAGU287-15|Diptera|Phoridae||BOLD:ACX5924
Phoridae|[298]|GMAGC707-15|Diptera|Phoridae||BOLD:ACW2527
Phoridae|[299]|GMARE768-14|Diptera|Phoridae||BOLD:ACM7678

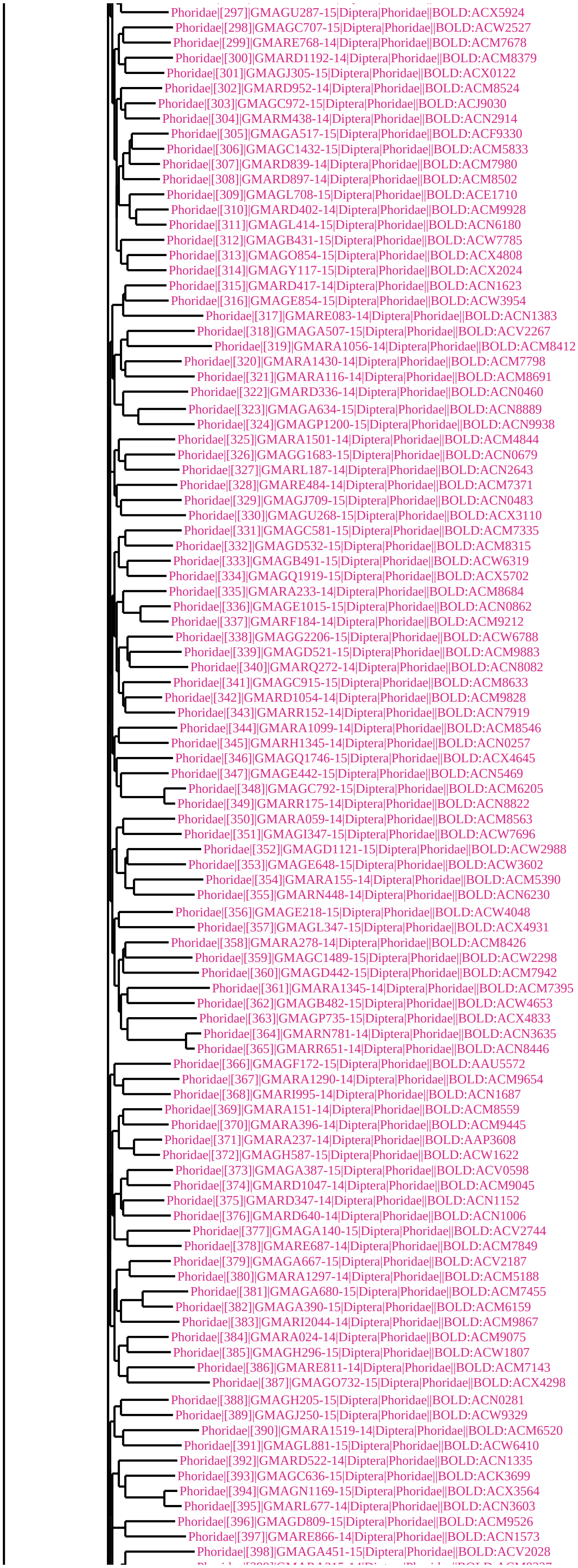
Phoridae|[297]|GMAGU287-15|Diptera|Phoridae||BOLD:ACX5924
Phoridae|[298]|GMAGC707-15|Diptera|Phoridae||BOLD:ACW2527
Phoridae|[299]|GMARE768-14|Diptera|Phoridae||BOLD:ACM7678
Phoridae|[300]|GMARD1192-14|Diptera|Phoridae||BOLD:ACM8379
Phoridae|[301]|GMAGJ305-15|Diptera|Phoridae||BOLD:ACX0122
Phoridae|[302]|GMARD952-14|Diptera|Phoridae||BOLD:ACM8524
Phoridae|[303]|GMAGC972-15|Diptera|Phoridae||BOLD:ACJ9030
Phoridae|[304]|GMARM438-14|Diptera|Phoridae||BOLD:ACN2914
Phoridae|[305]|GMAGA517-15|Diptera|Phoridae||BOLD:ACF9330
Phoridae|[306]|GMAGC1432-15|Diptera|Phoridae||BOLD:ACM5833
Phoridae|[307]|GMARD839-14|Diptera|Phoridae||BOLD:ACM7980
Phoridae|[308]|GMARD897-14|Diptera|Phoridae||BOLD:ACM8502
Phoridae|[309]|GMAGL708-15|Diptera|Phoridae||BOLD:ACE1710
Phoridae|[310]|GMARD402-14|Diptera|Phoridae||BOLD:ACM9928
Phoridae|[311]|GMAGL414-15|Diptera|Phoridae||BOLD:ACN6180
Phoridae|[312]|GMAGB431-15|Diptera|Phoridae||BOLD:ACW7785
Phoridae|[313]|GMAGO854-15|Diptera|Phoridae||BOLD:ACX4808
Phoridae|[314]|GMAGY117-15|Diptera|Phoridae||BOLD:ACX2024
Phoridae|[315]|GMARD417-14|Diptera|Phoridae||BOLD:ACN1623
Phoridae|[316]|GMAGE854-15|Diptera|Phoridae||BOLD:ACW3954
Phoridae|[317]|GMARE083-14|Diptera|Phoridae||BOLD:ACN1383
Phoridae|[318]|GMAGA507-15|Diptera|Phoridae||BOLD:ACV2267
Phoridae|[319]|GMARA1056-14|Diptera|Phoridae||BOLD:ACM8412
Phoridae|[320]|GMARA1430-14|Diptera|Phoridae||BOLD:ACM7798
Phoridae|[321]|GMARA116-14|Diptera|Phoridae||BOLD:ACM8691
Phoridae|[322]|GMARD336-14|Diptera|Phoridae||BOLD:ACN0460
Phoridae|[323]|GMAGA634-15|Diptera|Phoridae||BOLD:ACN8889
Phoridae|[324]|GMAGP1200-15|Diptera|Phoridae||BOLD:ACN9938
Phoridae|[325]|GMARA1501-14|Diptera|Phoridae||BOLD:ACM4844
Phoridae|[326]|GMAGG1683-15|Diptera|Phoridae||BOLD:ACN0679
Phoridae|[327]|GMARL187-14|Diptera|Phoridae||BOLD:ACN2643
Phoridae|[328]|GMARE484-14|Diptera|Phoridae||BOLD:ACM7371
Phoridae|[329]|GMAGJ709-15|Diptera|Phoridae||BOLD:ACN0483
Phoridae|[330]|GMAGU268-15|Diptera|Phoridae||BOLD:ACX3110
Phoridae|[331]|GMAGC581-15|Diptera|Phoridae||BOLD:ACM7335
Phoridae|[332]|GMAGD532-15|Diptera|Phoridae||BOLD:ACM8315
Phoridae|[333]|GMAGB491-15|Diptera|Phoridae||BOLD:ACW6319
Phoridae|[334]|GMAGQ1919-15|Diptera|Phoridae||BOLD:ACX5702
Phoridae|[335]|GMARA233-14|Diptera|Phoridae||BOLD:ACM8684
Phoridae|[336]|GMAGE1015-15|Diptera|Phoridae||BOLD:ACN0862
Phoridae|[337]|GMARF184-14|Diptera|Phoridae||BOLD:ACM9212
Phoridae|[338]|GMAGG2206-15|Diptera|Phoridae||BOLD:ACW6788
Phoridae|[339]|GMAGD521-15|Diptera|Phoridae||BOLD:ACM9883
Phoridae|[340]|GMARQ272-14|Diptera|Phoridae||BOLD:ACN8082
Phoridae|[341]|GMAGC915-15|Diptera|Phoridae||BOLD:ACM8633
Phoridae|[342]|GMARD1054-14|Diptera|Phoridae||BOLD:ACM9828
Phoridae|[343]|GMARR152-14|Diptera|Phoridae||BOLD:ACN7919
Phoridae|[344]|GMARA1099-14|Diptera|Phoridae||BOLD:ACM8546
Phoridae|[345]|GMARH1345-14|Diptera|Phoridae||BOLD:ACN0257
Phoridae|[346]|GMAGQ1746-15|Diptera|Phoridae||BOLD:ACX4645
Phoridae|[347]|GMAGE442-15|Diptera|Phoridae||BOLD:ACN5469
Phoridae|[348]|GMAGC792-15|Diptera|Phoridae||BOLD:ACM6205
Phoridae|[349]|GMARR175-14|Diptera|Phoridae||BOLD:ACN8822
Phoridae|[350]|GMARA059-14|Diptera|Phoridae||BOLD:ACM8563
Phoridae|[351]|GMAGI347-15|Diptera|Phoridae||BOLD:ACW7696
Phoridae|[352]|GMAGD1121-15|Diptera|Phoridae||BOLD:ACW2988
Phoridae|[353]|GMAGE648-15|Diptera|Phoridae||BOLD:ACW3602
Phoridae|[354]|GMARA155-14|Diptera|Phoridae||BOLD:ACM5390
Phoridae|[355]|GMARN448-14|Diptera|Phoridae||BOLD:ACN6230
Phoridae|[356]|GMAGE218-15|Diptera|Phoridae||BOLD:ACW4048
Phoridae|[357]|GMAGL347-15|Diptera|Phoridae||BOLD:ACX4931
Phoridae|[358]|GMARA278-14|Diptera|Phoridae||BOLD:ACM8426
Phoridae|[359]|GMAGC1489-15|Diptera|Phoridae||BOLD:ACW2298
Phoridae|[360]|GMAGD442-15|Diptera|Phoridae||BOLD:ACM7942
Phoridae|[361]|GMARA1345-14|Diptera|Phoridae||BOLD:ACM7395
Phoridae|[362]|GMAGB482-15|Diptera|Phoridae||BOLD:ACW4653
Phoridae|[363]|GMAGP735-15|Diptera|Phoridae||BOLD:ACX4833
Phoridae|[364]|GMARN781-14|Diptera|Phoridae||BOLD:ACN3635
Phoridae|[365]|GMARR651-14|Diptera|Phoridae||BOLD:ACN8446
Phoridae|[366]|GMAGF172-15|Diptera|Phoridae||BOLD:AAU5572
Phoridae|[367]|GMARA1290-14|Diptera|Phoridae||BOLD:ACM9654
Phoridae|[368]|GMARI995-14|Diptera|Phoridae||BOLD:ACN1687
Phoridae|[369]|GMARA151-14|Diptera|Phoridae||BOLD:ACM8559
Phoridae|[370]|GMARA396-14|Diptera|Phoridae||BOLD:ACM9445
Phoridae|[371]|GMARA237-14|Diptera|Phoridae||BOLD:AAP3608
Phoridae|[372]|GMAGH587-15|Diptera|Phoridae||BOLD:ACW1622
Phoridae|[373]|GMAGA387-15|Diptera|Phoridae||BOLD:ACV0598
Phoridae|[374]|GMARD1047-14|Diptera|Phoridae||BOLD:ACM9045
Phoridae|[375]|GMARD347-14|Diptera|Phoridae||BOLD:ACN1152
Phoridae|[376]|GMARD640-14|Diptera|Phoridae||BOLD:ACN1006
Phoridae|[377]|GMAGA140-15|Diptera|Phoridae||BOLD:ACV2744
Phoridae|[378]|GMARE687-14|Diptera|Phoridae||BOLD:ACM7849
Phoridae|[379]|GMAGA667-15|Diptera|Phoridae||BOLD:ACV2187
Phoridae|[380]|GMARA1297-14|Diptera|Phoridae||BOLD:ACM5188
Phoridae|[381]|GMAGA680-15|Diptera|Phoridae||BOLD:ACM7455
Phoridae|[382]|GMAGA390-15|Diptera|Phoridae||BOLD:ACM6159
Phoridae|[383]|GMARI2044-14|Diptera|Phoridae||BOLD:ACM9867
Phoridae|[384]|GMARA024-14|Diptera|Phoridae||BOLD:ACM9075
Phoridae|[385]|GMAGH296-15|Diptera|Phoridae||BOLD:ACW1807
Phoridae|[386]|GMARE811-14|Diptera|Phoridae||BOLD:ACM7143
Phoridae|[387]|GMAGO732-15|Diptera|Phoridae||BOLD:ACX4298
Phoridae|[388]|GMAGH205-15|Diptera|Phoridae||BOLD:ACN0281
Phoridae|[389]|GMAGJ250-15|Diptera|Phoridae||BOLD:ACW9329
Phoridae|[390]|GMARA1519-14|Diptera|Phoridae||BOLD:ACM6520
Phoridae|[391]|GMAGL881-15|Diptera|Phoridae||BOLD:ACW6410
Phoridae|[392]|GMARD522-14|Diptera|Phoridae||BOLD:ACN1335
Phoridae|[393]|GMAGC636-15|Diptera|Phoridae||BOLD:ACK3699
Phoridae|[394]|GMAGN1169-15|Diptera|Phoridae||BOLD:ACX3564
Phoridae|[395]|GMARL677-14|Diptera|Phoridae||BOLD:ACN3603
Phoridae|[396]|GMAGD809-15|Diptera|Phoridae||BOLD:ACM9526
Phoridae|[397]|GMARE866-14|Diptera|Phoridae||BOLD:ACN1573
Phoridae|[398]|GMAGA451-15|Diptera|Phoridae||BOLD:ACV2028

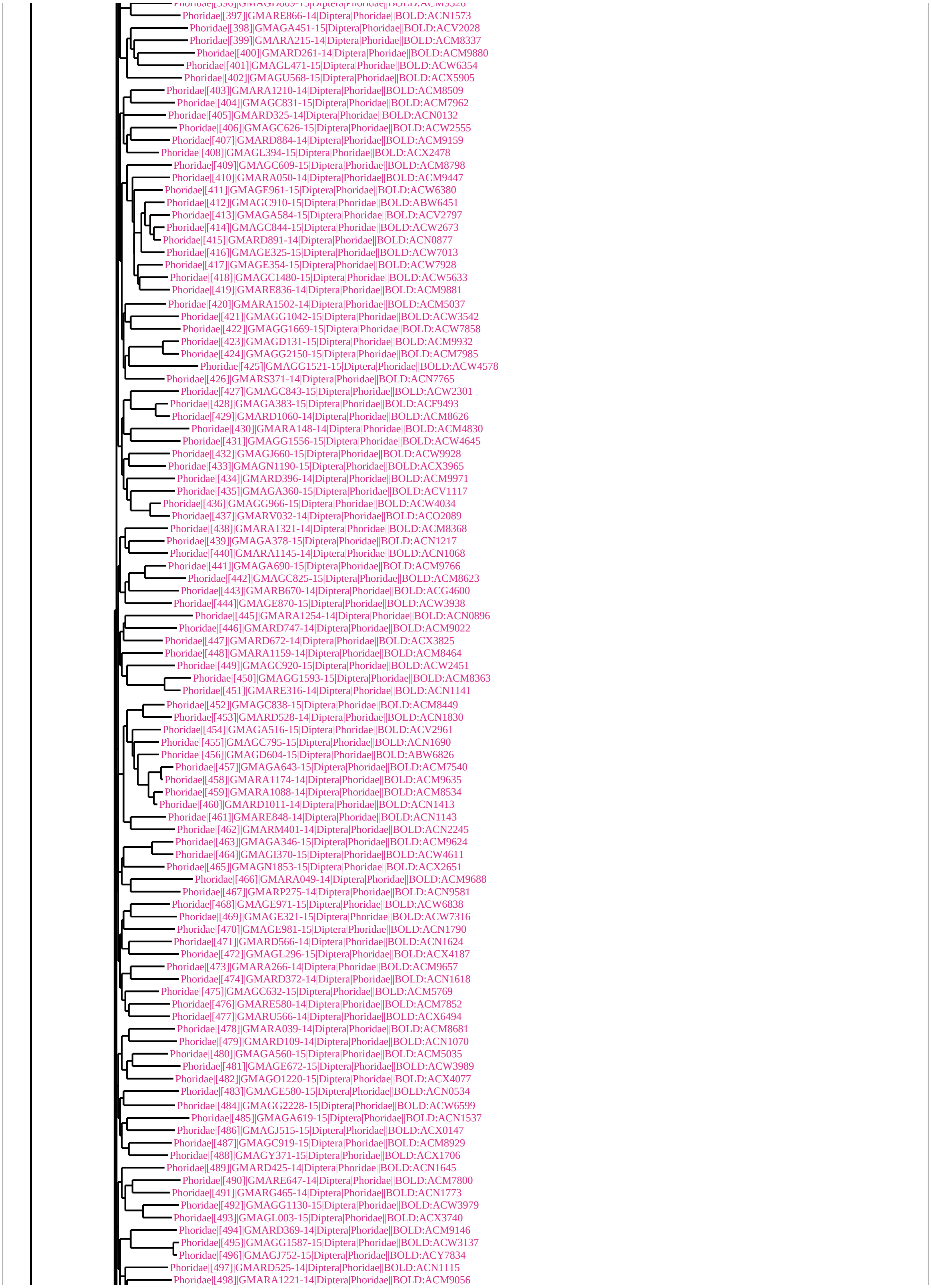
Phoridae|[397]|GMARE866-14|Diptera|Phoridae||BOLD:ACN1573
Phoridae|[398]|GMAGA451-15|Diptera|Phoridae||BOLD:ACV2028
Phoridae|[399]|GMARA215-14|Diptera|Phoridae||BOLD:ACM8337
Phoridae|[400]|GMARD261-14|Diptera|Phoridae||BOLD:ACM9880
Phoridae|[401]|GMAGL471-15|Diptera|Phoridae||BOLD:ACW6354
Phoridae|[402]|GMAGU568-15|Diptera|Phoridae||BOLD:ACX5905
Phoridae|[403]|GMARA1210-14|Diptera|Phoridae||BOLD:ACM8509
Phoridae|[404]|GMAGC831-15|Diptera|Phoridae||BOLD:ACM7962
Phoridae|[405]|GMARD325-14|Diptera|Phoridae||BOLD:ACN0132
Phoridae|[406]|GMAGC626-15|Diptera|Phoridae||BOLD:ACW2555
Phoridae|[407]|GMARD884-14|Diptera|Phoridae||BOLD:ACM9159
Phoridae|[408]|GMAGL394-15|Diptera|Phoridae||BOLD:ACX2478
Phoridae|[409]|GMAGC609-15|Diptera|Phoridae||BOLD:ACM8798
Phoridae|[410]|GMARA050-14|Diptera|Phoridae||BOLD:ACM9447
Phoridae|[411]|GMAGE961-15|Diptera|Phoridae||BOLD:ACW6380
Phoridae|[412]|GMAGC910-15|Diptera|Phoridae||BOLD:ABW6451
Phoridae|[413]|GMAGA584-15|Diptera|Phoridae||BOLD:ACV2797
Phoridae|[414]|GMAGC844-15|Diptera|Phoridae||BOLD:ACW2673
Phoridae|[415]|GMARD891-14|Diptera|Phoridae||BOLD:ACN0877
Phoridae|[416]|GMAGE325-15|Diptera|Phoridae||BOLD:ACW7013
Phoridae|[417]|GMAGE354-15|Diptera|Phoridae||BOLD:ACW7928
Phoridae|[418]|GMAGC1480-15|Diptera|Phoridae||BOLD:ACW5633
Phoridae|[419]|GMARE836-14|Diptera|Phoridae||BOLD:ACM9881
Phoridae|[420]|GMARA1502-14|Diptera|Phoridae||BOLD:ACM5037
Phoridae|[421]|GMAGG1042-15|Diptera|Phoridae||BOLD:ACW3542
Phoridae|[422]|GMAGG1669-15|Diptera|Phoridae||BOLD:ACW7858
Phoridae|[423]|GMAGD131-15|Diptera|Phoridae||BOLD:ACM9932
Phoridae|[424]|GMAGG2150-15|Diptera|Phoridae||BOLD:ACM7985
Phoridae|[425]|GMAGG1521-15|Diptera|Phoridae||BOLD:ACW4578
Phoridae|[426]|GMARS371-14|Diptera|Phoridae||BOLD:ACN7765
Phoridae|[427]|GMAGC843-15|Diptera|Phoridae||BOLD:ACW2301
Phoridae|[428]|GMAGA383-15|Diptera|Phoridae||BOLD:ACF9493
Phoridae|[429]|GMARD1060-14|Diptera|Phoridae||BOLD:ACM8626
Phoridae|[430]|GMARA148-14|Diptera|Phoridae||BOLD:ACM4830
Phoridae|[431]|GMAGG1556-15|Diptera|Phoridae||BOLD:ACW4645
Phoridae|[432]|GMAGJ660-15|Diptera|Phoridae||BOLD:ACW9928
Phoridae|[433]|GMAGN1190-15|Diptera|Phoridae||BOLD:ACX3965
Phoridae|[434]|GMARD396-14|Diptera|Phoridae||BOLD:ACM9971
Phoridae|[435]|GMAGA360-15|Diptera|Phoridae||BOLD:ACV1117
Phoridae|[436]|GMAGG966-15|Diptera|Phoridae||BOLD:ACW4034
Phoridae|[437]|GMARV032-14|Diptera|Phoridae||BOLD:ACO2089
Phoridae|[438]|GMARA1321-14|Diptera|Phoridae||BOLD:ACM8368
Phoridae|[439]|GMAGA378-15|Diptera|Phoridae||BOLD:ACN1217
Phoridae|[440]|GMARA1145-14|Diptera|Phoridae||BOLD:ACN1068
Phoridae|[441]|GMAGA690-15|Diptera|Phoridae||BOLD:ACM9766
Phoridae|[442]|GMAGC825-15|Diptera|Phoridae||BOLD:ACM8623
Phoridae|[443]|GMARB670-14|Diptera|Phoridae||BOLD:ACG4600
Phoridae|[444]|GMAGE870-15|Diptera|Phoridae||BOLD:ACW3938
Phoridae|[445]|GMARA1254-14|Diptera|Phoridae||BOLD:ACN0896
Phoridae|[446]|GMARD747-14|Diptera|Phoridae||BOLD:ACM9022
Phoridae|[447]|GMARD672-14|Diptera|Phoridae||BOLD:ACX3825
Phoridae|[448]|GMARA1159-14|Diptera|Phoridae||BOLD:ACM8464
Phoridae|[449]|GMAGC920-15|Diptera|Phoridae||BOLD:ACW2451
Phoridae|[450]|GMAGG1593-15|Diptera|Phoridae||BOLD:ACM8363
Phoridae|[451]|GMARE316-14|Diptera|Phoridae||BOLD:ACN1141
Phoridae|[452]|GMAGC838-15|Diptera|Phoridae||BOLD:ACM8449
Phoridae|[453]|GMARD528-14|Diptera|Phoridae||BOLD:ACN1830
Phoridae|[454]|GMAGA516-15|Diptera|Phoridae||BOLD:ACV2961
Phoridae|[455]|GMAGC795-15|Diptera|Phoridae||BOLD:ACN1690
Phoridae|[456]|GMAGD604-15|Diptera|Phoridae||BOLD:ABW6826
Phoridae|[457]|GMAGA643-15|Diptera|Phoridae||BOLD:ACM7540
Phoridae|[458]|GMARA1174-14|Diptera|Phoridae||BOLD:ACM9635
Phoridae|[459]|GMARA1088-14|Diptera|Phoridae||BOLD:ACM8534
Phoridae|[460]|GMARD1011-14|Diptera|Phoridae||BOLD:ACN1413
Phoridae|[461]|GMARE848-14|Diptera|Phoridae||BOLD:ACN1143
Phoridae|[462]|GMARM401-14|Diptera|Phoridae||BOLD:ACN2245
Phoridae|[463]|GMAGA346-15|Diptera|Phoridae||BOLD:ACM9624
Phoridae|[464]|GMAGI370-15|Diptera|Phoridae||BOLD:ACW4611
Phoridae|[465]|GMAGN1853-15|Diptera|Phoridae||BOLD:ACX2651
Phoridae|[466]|GMARA049-14|Diptera|Phoridae||BOLD:ACM9688
Phoridae|[467]|GMARP275-14|Diptera|Phoridae||BOLD:ACN9581
Phoridae|[468]|GMAGE971-15|Diptera|Phoridae||BOLD:ACW6838
Phoridae|[469]|GMAGE321-15|Diptera|Phoridae||BOLD:ACW7316
Phoridae|[470]|GMAGE981-15|Diptera|Phoridae||BOLD:ACN1790
Phoridae|[471]|GMARD566-14|Diptera|Phoridae||BOLD:ACN1624
Phoridae|[472]|GMAGL296-15|Diptera|Phoridae||BOLD:ACX4187
Phoridae|[473]|GMARA266-14|Diptera|Phoridae||BOLD:ACM9657
Phoridae|[474]|GMARD372-14|Diptera|Phoridae||BOLD:ACN1618
Phoridae|[475]|GMAGC632-15|Diptera|Phoridae||BOLD:ACM5769
Phoridae|[476]|GMARE580-14|Diptera|Phoridae||BOLD:ACM7852
Phoridae|[477]|GMARU566-14|Diptera|Phoridae||BOLD:ACX6494
Phoridae|[478]|GMARA039-14|Diptera|Phoridae||BOLD:ACM8681
Phoridae|[479]|GMARD109-14|Diptera|Phoridae||BOLD:ACN1070
Phoridae|[480]|GMAGA560-15|Diptera|Phoridae||BOLD:ACM5035
Phoridae|[481]|GMAGE672-15|Diptera|Phoridae||BOLD:ACW3989
Phoridae|[482]|GMAGO1220-15|Diptera|Phoridae||BOLD:ACX4077
Phoridae|[483]|GMAGE580-15|Diptera|Phoridae||BOLD:ACN0534
Phoridae|[484]|GMAGG2228-15|Diptera|Phoridae||BOLD:ACW6599
Phoridae|[485]|GMAGA619-15|Diptera|Phoridae||BOLD:ACN1537
Phoridae|[486]|GMAGJ515-15|Diptera|Phoridae||BOLD:ACX0147
Phoridae|[487]|GMAGC919-15|Diptera|Phoridae||BOLD:ACM8929
Phoridae|[488]|GMAGY371-15|Diptera|Phoridae||BOLD:ACX1706
Phoridae|[489]|GMARD425-14|Diptera|Phoridae||BOLD:ACN1645
Phoridae|[490]|GMARE647-14|Diptera|Phoridae||BOLD:ACM7800
Phoridae|[491]|GMARG465-14|Diptera|Phoridae||BOLD:ACN1773
Phoridae|[492]|GMAGG1130-15|Diptera|Phoridae||BOLD:ACW3979
Phoridae|[493]|GMAGL003-15|Diptera|Phoridae||BOLD:ACX3740
Phoridae|[494]|GMARD369-14|Diptera|Phoridae||BOLD:ACM9146
Phoridae|[495]|GMAGG1587-15|Diptera|Phoridae||BOLD:ACW3137
Phoridae|[496]|GMAGJ752-15|Diptera|Phoridae||BOLD:ACY7834
Phoridae|[497]|GMARD525-14|Diptera|Phoridae||BOLD:ACN1115
Phoridae|[498]|GMARA1221-14|Diptera|Phoridae||BOLD:ACM9056

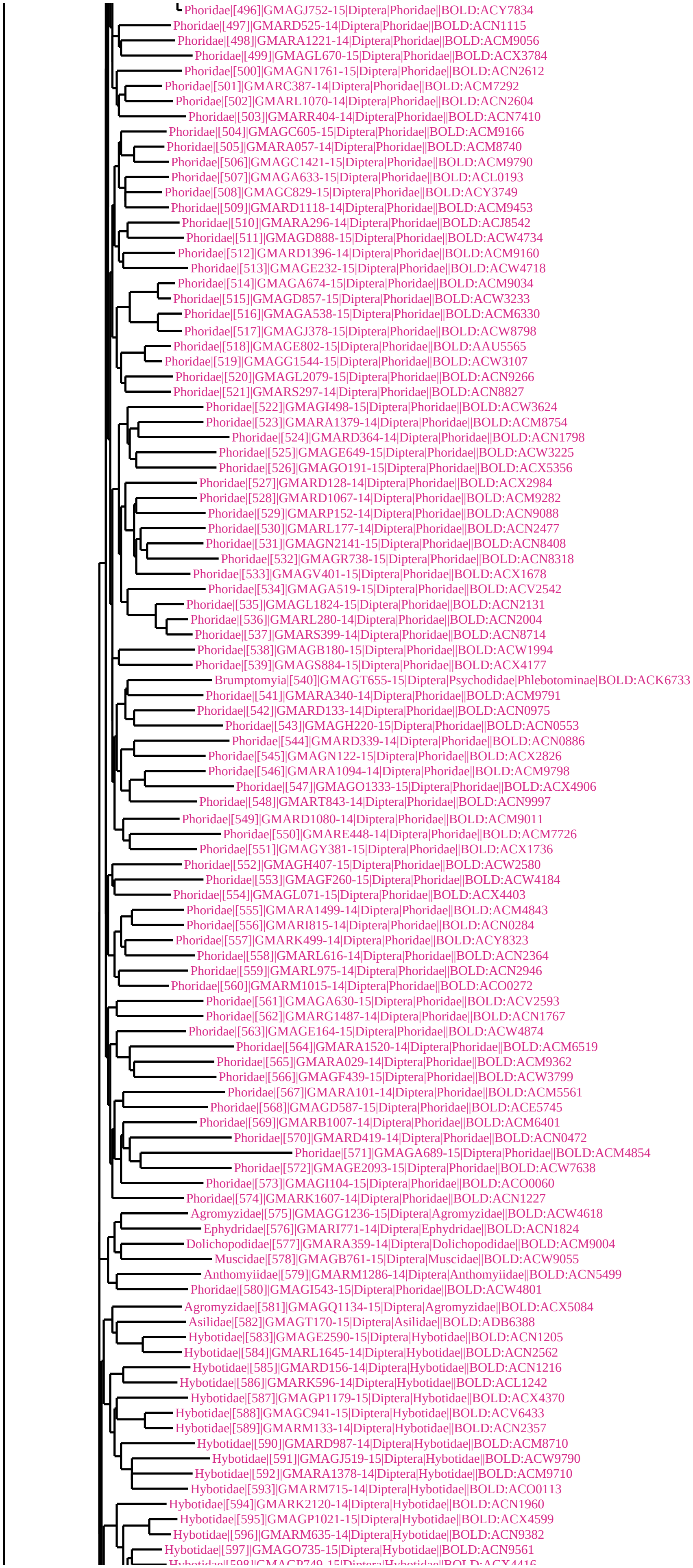
Phoridae|[496]|GMAGJ752-15|Diptera|Phoridae||BOLD:ACY7834
Phoridae|[497]|GMARD525-14|Diptera|Phoridae||BOLD:ACN1115
Phoridae|[498]|GMARA1221-14|Diptera|Phoridae||BOLD:ACM9056
Phoridae|[499]|GMAGL670-15|Diptera|Phoridae||BOLD:ACX3784
Phoridae|[500]|GMAGN1761-15|Diptera|Phoridae||BOLD:ACN2612
Phoridae|[501]|GMARC387-14|Diptera|Phoridae||BOLD:ACM7292
Phoridae|[502]|GMARL1070-14|Diptera|Phoridae||BOLD:ACN2604
Phoridae|[503]|GMARR404-14|Diptera|Phoridae||BOLD:ACN7410
Phoridae|[504]|GMAGC605-15|Diptera|Phoridae||BOLD:ACM9166
Phoridae|[505]|GMARA057-14|Diptera|Phoridae||BOLD:ACM8740
Phoridae|[506]|GMAGC1421-15|Diptera|Phoridae||BOLD:ACM9790
Phoridae|[507]|GMAGA633-15|Diptera|Phoridae||BOLD:ACL0193
Phoridae|[508]|GMAGC829-15|Diptera|Phoridae||BOLD:ACY3749
Phoridae|[509]|GMARD1118-14|Diptera|Phoridae||BOLD:ACM9453
Phoridae|[510]|GMARA296-14|Diptera|Phoridae||BOLD:ACJ8542
Phoridae|[511]|GMAGD888-15|Diptera|Phoridae||BOLD:ACW4734
Phoridae|[512]|GMARD1396-14|Diptera|Phoridae||BOLD:ACM9160
Phoridae|[513]|GMAGE232-15|Diptera|Phoridae||BOLD:ACW4718
Phoridae|[514]|GMAGA674-15|Diptera|Phoridae||BOLD:ACM9034
Phoridae|[515]|GMAGD857-15|Diptera|Phoridae||BOLD:ACW3233
Phoridae|[516]|GMAGA538-15|Diptera|Phoridae||BOLD:ACM6330
Phoridae|[517]|GMAGJ378-15|Diptera|Phoridae||BOLD:ACW8798
Phoridae|[518]|GMAGE802-15|Diptera|Phoridae||BOLD:AAU5565
Phoridae|[519]|GMAGG1544-15|Diptera|Phoridae||BOLD:ACW3107
Phoridae|[520]|GMAGL2079-15|Diptera|Phoridae||BOLD:ACN9266
Phoridae|[521]|GMARS297-14|Diptera|Phoridae||BOLD:ACN8827
Phoridae|[522]|GMAGI498-15|Diptera|Phoridae||BOLD:ACW3624
Phoridae|[523]|GMARA1379-14|Diptera|Phoridae||BOLD:ACM8754
Phoridae|[524]|GMARD364-14|Diptera|Phoridae||BOLD:ACN1798
Phoridae|[525]|GMAGE649-15|Diptera|Phoridae||BOLD:ACW3225
Phoridae|[526]|GMAGO191-15|Diptera|Phoridae||BOLD:ACX5356
Phoridae|[527]|GMARD128-14|Diptera|Phoridae||BOLD:ACX2984
Phoridae|[528]|GMARD1067-14|Diptera|Phoridae||BOLD:ACM9282
Phoridae|[529]|GMARP152-14|Diptera|Phoridae||BOLD:ACN9088
Phoridae|[530]|GMARL177-14|Diptera|Phoridae||BOLD:ACN2477
Phoridae|[531]|GMAGN2141-15|Diptera|Phoridae||BOLD:ACN8408
Phoridae|[532]|GMAGR738-15|Diptera|Phoridae||BOLD:ACN8318
Phoridae|[533]|GMAGV401-15|Diptera|Phoridae||BOLD:ACX1678
Phoridae|[534]|GMAGA519-15|Diptera|Phoridae||BOLD:ACV2542
Phoridae|[535]|GMAGL1824-15|Diptera|Phoridae||BOLD:ACN2131
Phoridae|[536]|GMARL280-14|Diptera|Phoridae||BOLD:ACN2004
Phoridae|[537]|GMARS399-14|Diptera|Phoridae||BOLD:ACN8714
Phoridae|[538]|GMAGB180-15|Diptera|Phoridae||BOLD:ACW1994
Phoridae|[539]|GMAGS884-15|Diptera|Phoridae||BOLD:ACX4177
Brumptomyia|[540]|GMAGT655-15|Diptera|Psychodidae|Phlebotominae|BOLD:ACK6733
Phoridae|[541]|GMARA340-14|Diptera|Phoridae||BOLD:ACM9791
Phoridae|[542]|GMARD133-14|Diptera|Phoridae||BOLD:ACN0975
Phoridae|[543]|GMAGH220-15|Diptera|Phoridae||BOLD:ACN0553
Phoridae|[544]|GMARD339-14|Diptera|Phoridae||BOLD:ACN0886
Phoridae|[545]|GMAGN122-15|Diptera|Phoridae||BOLD:ACX2826
Phoridae|[546]|GMARA1094-14|Diptera|Phoridae||BOLD:ACM9798
Phoridae|[547]|GMAGO1333-15|Diptera|Phoridae||BOLD:ACX4906
Phoridae|[548]|GMART843-14|Diptera|Phoridae||BOLD:ACN9997
Phoridae|[549]|GMARD1080-14|Diptera|Phoridae||BOLD:ACM9011
Phoridae|[550]|GMARE448-14|Diptera|Phoridae||BOLD:ACM7726
Phoridae|[551]|GMAGY381-15|Diptera|Phoridae||BOLD:ACX1736
Phoridae|[552]|GMAGH407-15|Diptera|Phoridae||BOLD:ACW2580
Phoridae|[553]|GMAGF260-15|Diptera|Phoridae||BOLD:ACW4184
Phoridae|[554]|GMAGL071-15|Diptera|Phoridae||BOLD:ACX4403
Phoridae|[555]|GMARA1499-14|Diptera|Phoridae||BOLD:ACM4843
Phoridae|[556]|GMARI815-14|Diptera|Phoridae||BOLD:ACN0284
Phoridae|[557]|GMARK499-14|Diptera|Phoridae||BOLD:ACY8323
Phoridae|[558]|GMARL616-14|Diptera|Phoridae||BOLD:ACN2364
Phoridae|[559]|GMARL975-14|Diptera|Phoridae||BOLD:ACN2946
Phoridae|[560]|GMARM1015-14|Diptera|Phoridae||BOLD:ACO0272
Phoridae|[561]|GMAGA630-15|Diptera|Phoridae||BOLD:ACV2593
Phoridae|[562]|GMARG1487-14|Diptera|Phoridae||BOLD:ACN1767
Phoridae|[563]|GMAGE164-15|Diptera|Phoridae||BOLD:ACW4874
Phoridae|[564]|GMARA1520-14|Diptera|Phoridae||BOLD:ACM6519
Phoridae|[565]|GMARA029-14|Diptera|Phoridae||BOLD:ACM9362
Phoridae|[566]|GMAGF439-15|Diptera|Phoridae||BOLD:ACW3799
Phoridae|[567]|GMARA101-14|Diptera|Phoridae||BOLD:ACM5561
Phoridae|[568]|GMAGD587-15|Diptera|Phoridae||BOLD:ACE5745
Phoridae|[569]|GMARB1007-14|Diptera|Phoridae||BOLD:ACM6401
Phoridae|[570]|GMARD419-14|Diptera|Phoridae||BOLD:ACN0472
Phoridae|[571]|GMAGA689-15|Diptera|Phoridae||BOLD:ACM4854
Phoridae|[572]|GMAGE2093-15|Diptera|Phoridae||BOLD:ACW7638
Phoridae|[573]|GMAGI104-15|Diptera|Phoridae||BOLD:ACO0060
Phoridae|[574]|GMARK1607-14|Diptera|Phoridae||BOLD:ACN1227
Agromyzidae|[575]|GMAGG1236-15|Diptera|Agromyzidae||BOLD:ACW4618
Ephydridae|[576]|GMARI771-14|Diptera|Ephydridae||BOLD:ACN1824
Dolichopodidae|[577]|GMARA359-14|Diptera|Dolichopodidae||BOLD:ACM9004
Muscidae|[578]|GMAGB761-15|Diptera|Muscidae||BOLD:ACW9055
Anthomyiidae|[579]|GMARM1286-14|Diptera|Anthomyiidae||BOLD:ACN5499
Phoridae|[580]|GMAGI543-15|Diptera|Phoridae||BOLD:ACW4801
Agromyzidae|[581]|GMAGQ1134-15|Diptera|Agromyzidae||BOLD:ACX5084
Asilidae|[582]|GMAGT170-15|Diptera|Asilidae||BOLD:ADB6388
Hybotidae|[583]|GMAGE2590-15|Diptera|Hybotidae||BOLD:ACN1205
Hybotidae|[584]|GMARL1645-14|Diptera|Hybotidae||BOLD:ACN2562
Hybotidae|[585]|GMARD156-14|Diptera|Hybotidae||BOLD:ACN1216
Hybotidae|[586]|GMARK596-14|Diptera|Hybotidae||BOLD:ACL1242
Hybotidae|[587]|GMAGP1179-15|Diptera|Hybotidae||BOLD:ACX4370
Hybotidae|[588]|GMAGC941-15|Diptera|Hybotidae||BOLD:ACV6433
Hybotidae|[589]|GMARM133-14|Diptera|Hybotidae||BOLD:ACN2357
Hybotidae|[590]|GMARD987-14|Diptera|Hybotidae||BOLD:ACM8710
Hybotidae|[591]|GMAGJ519-15|Diptera|Hybotidae||BOLD:ACW9790
Hybotidae|[592]|GMARA1378-14|Diptera|Hybotidae||BOLD:ACM9710
Hybotidae|[593]|GMARM715-14|Diptera|Hybotidae||BOLD:ACO0113
Hybotidae|[594]|GMARK2120-14|Diptera|Hybotidae||BOLD:ACN1960
Hybotidae|[595]|GMAGP1021-15|Diptera|Hybotidae||BOLD:ACX4599
Hybotidae|[596]|GMARM635-14|Diptera|Hybotidae||BOLD:ACN9382
Hybotidae|[597]|GMAGO735-15|Diptera|Hybotidae||BOLD:ACN9561

Hybotidae|[595]|GMAGI1021-15|Diptera|Hybotidae||BOLD:ACX4359
Hybotidae|[596]|GMARM635-14|Diptera|Hybotidae||BOLD:ACN9382
Hybotidae|[597]|GMAGO735-15|Diptera|Hybotidae||BOLD:ACN9561
Hybotidae|[598]|GMAGP749-15|Diptera|Hybotidae||BOLD:ACX4416
Hybotidae|[599]|GMARN533-14|Diptera|Hybotidae||BOLD:ACW6097
Limoniidae|[600]|GMARE248-14|Diptera|Limoniidae||BOLD:ACN1564
Limoniidae|[601]|GMAGJ554-15|Diptera|Limoniidae||BOLD:ACW8354
Rhipidia|[602]|GMAGE259-15|Diptera|Limoniidae|Limoniinae|BOLD:AAN4385
Limoniidae|[603]|GMARG1284-14|Diptera|Limoniidae||BOLD:ACN0613
Limoniidae|[604]|GMAGL204-15|Diptera|Limoniidae||BOLD:ACN1018
Limoniidae|[605]|GMARK1619-14|Diptera|Limoniidae||BOLD:ACN1395
Limoniidae|[606]|GMAGN528-15|Diptera|Limoniidae||BOLD:ACX4892
Limoniidae|[607]|GMARL332-14|Diptera|Limoniidae||BOLD:ACN1972
Limoniidae|[608]|GMARK713-14|Diptera|Limoniidae||BOLD:ACN0335
Limoniidae|[609]|GMARP243-14|Diptera|Limoniidae||BOLD:ACN9845
Anopheles|[610]|GMAGK230-15|Diptera|Culicidae|Anophelinae|BOLD:ACE5103
Culicidae|[611]|GMARD709-14|Diptera|Culicidae||BOLD:ACN1211
Culicidae|[612]|GMARF723-14|Diptera|Culicidae||BOLD:ACM8680
Culex spinosus|[613]|GMAGN582-15|Diptera|Culicidae|Culicinae|BOLD:ACQ7571
Culex|[614]|GMARG430-14|Diptera|Culicidae|Culicinae|BOLD:AAF1735
Culex|[615]|GMARP486-14|Diptera|Culicidae|Culicinae|BOLD:AAN3636
Culicidae|[616]|GMAGM434-15|Diptera|Culicidae||BOLD:ACX3516
Ceratopogonidae|[617]|GMARG1550-14|Diptera|Ceratopogonidae||BOLD:ACN1907
Ceratopogonidae|[618]|GMARK1177-14|Diptera|Ceratopogonidae||BOLD:ACN0302
Ceratopogonidae|[619]|GMAGN1928-15|Diptera|Ceratopogonidae||BOLD:ACX3082
Ceratopogonidae|[620]|GMARK2127-14|Diptera|Ceratopogonidae||BOLD:ACN0177
Diptera|[621]|GMARN747-14|Diptera|||BOLD:ACW6519
Diptera|[622]|GMAGU527-15|Diptera|||BOLD:ACX5110
Carabidae|[623]|GMAGA783-15|Coleoptera|Carabidae||BOLD:ACV2747
Carabidae|[624]|GMAGC1286-15|Coleoptera|Carabidae||BOLD:ACO3205
Carabidae|[625]|GMAGK138-15|Coleoptera|Carabidae||BOLD:ACW5985
Carabidae|[626]|GMARM1302-14|Coleoptera|Carabidae||BOLD:ACN5150
Carabidae|[627]|GMARB066-14|Coleoptera|Carabidae||BOLD:ACM5732
Carabidae|[628]|GMAGQ613-15|Coleoptera|Carabidae||BOLD:ACN7383
Carabidae|[629]|GMAGQ674-15|Coleoptera|Carabidae||BOLD:ACX6061
Carabidae|[630]|GMARC968-14|Coleoptera|Carabidae||BOLD:ACM5517
Carabidae|[631]|GMARN2038-14|Coleoptera|Carabidae||BOLD:ACO0805
Carabidae|[632]|GMAGR038-15|Coleoptera|Carabidae||BOLD:ACO1020
Carabidae|[633]|GMARO1110-14|Coleoptera|Carabidae||BOLD:ACO3134
Carabidae|[634]|GMARO028-14|Coleoptera|Carabidae||BOLD:ACO1084
Carabidae|[635]|GMARM1539-14|Coleoptera|Carabidae||BOLD:ACN3224
Carabidae|[636]|GMARQ120-14|Coleoptera|Carabidae||BOLD:ACN9809
Carabidae|[637]|GMARR914-14|Coleoptera|Carabidae||BOLD:ACO0557
Cicindelinae|[638]|GMART1709-14|Coleoptera|Carabidae|Cicindelinae|BOLD:ABA0800
Carabidae|[639]|GMAGB751-15|Coleoptera|Carabidae||BOLD:ACW9002
Carabidae|[640]|GMARR1463-14|Coleoptera|Carabidae||BOLD:ACO0068
Dytiscidae|[641]|GMAGE1634-15|Coleoptera|Dytiscidae||BOLD:ACW2289
Carabidae|[642]|GMARN2035-14|Coleoptera|Carabidae||BOLD:ACO1150
Carabidae|[643]|GMAGZ067-15|Coleoptera|Carabidae||BOLD:ACX4224
Scirtidae|[644]|GMAGI1169-15|Coleoptera|Scirtidae||BOLD:ACW7011
Omolabus|[645]|GMAGH936-15|Coleoptera|Attelabidae|Attelabinae|BOLD:ACI0932
Omolabus|[646]|GMAGN363-15|Coleoptera|Attelabidae|Attelabinae|BOLD:ACI1653
Scirtidae|[647]|GMAGQ642-15|Coleoptera|Scirtidae||BOLD:ACN8762
Scirtidae|[648]|GMAGT1027-15|Coleoptera|Scirtidae||BOLD:ACX4309
Agromyzidae|[649]|GMAGH368-15|Diptera|Agromyzidae||BOLD:ACM9281
Agromyzidae|[650]|GMAGL230-15|Diptera|Agromyzidae||BOLD:ACX5731
Agromyzidae|[651]|GMAGC939-15|Diptera|Agromyzidae||BOLD:ACW0078
Agromyzidae|[652]|GMARK2171-14|Diptera|Agromyzidae||BOLD:ACN1034
Agromyzidae|[653]|GMAGH453-15|Diptera|Agromyzidae||BOLD:ACW2418
Agromyzidae|[654]|GMARL667-14|Diptera|Agromyzidae||BOLD:ACN6815
Agromyzidae|[655]|GMARK083-14|Diptera|Agromyzidae||BOLD:ACN0206
Agromyzidae|[656]|GMARF908-14|Diptera|Agromyzidae||BOLD:ACM8501
Agromyzidae|[657]|GMARL733-14|Diptera|Agromyzidae||BOLD:ACN2202
Agromyzidae|[658]|GMAGO538-15|Diptera|Agromyzidae||BOLD:ACX4250
Agromyzidae|[659]|GMARK1644-14|Diptera|Agromyzidae||BOLD:ACX3799
Agromyzidae|[660]|GMAGO766-15|Diptera|Agromyzidae||BOLD:ACX4230
Agromyzidae|[661]|GMAGP1166-15|Diptera|Agromyzidae||BOLD:ACX6006
Agromyzidae|[662]|GMARL883-14|Diptera|Agromyzidae||BOLD:ACN2744
Agromyzidae|[663]|GMAGN535-15|Diptera|Agromyzidae||BOLD:ACX5626
Agromyzidae|[664]|GMAGP351-15|Diptera|Agromyzidae||BOLD:ACX4375
Agromyzidae|[665]|GMARM425-14|Diptera|Agromyzidae||BOLD:AAO7887
Agromyzidae|[666]|GMARK1992-14|Diptera|Agromyzidae||BOLD:ACN0826
Agromyzidae|[667]|GMAGO368-15|Diptera|Agromyzidae||BOLD:ACX4821
Agromyzidae|[668]|GMAGP1002-15|Diptera|Agromyzidae||BOLD:ACX5759
Agromyzidae|[669]|GMAGH155-15|Diptera|Agromyzidae||BOLD:ACN4751
Agromyzidae|[670]|GMARK1759-14|Diptera|Agromyzidae||BOLD:ACN0105
Agromyzidae|[671]|GMARO196-14|Diptera|Agromyzidae||BOLD:ACN6234
Agromyzidae|[672]|GMAGI508-15|Diptera|Agromyzidae||BOLD:ACN0388
Agromyzidae|[673]|GMAGP895-15|Diptera|Agromyzidae||BOLD:ACX5554
Agromyzidae|[674]|GMAGL238-15|Diptera|Agromyzidae||BOLD:ACX3753
Agromyzidae|[675]|GMARL947-14|Diptera|Agromyzidae||BOLD:ACN2392
Agromyzidae|[676]|GMARN1388-14|Diptera|Agromyzidae||BOLD:ACN9557
Agromyzidae|[677]|GMARA206-14|Diptera|Agromyzidae||BOLD:ACM8823
Lauxaniidae|[678]|GMARL142-14|Diptera|Lauxaniidae||BOLD:ACN2316
Limoniidae|[679]|GMARO020-14|Diptera|Limoniidae||BOLD:ACO0832
Limoniidae|[680]|GMAGH008-15|Diptera|Limoniidae||BOLD:ACX5553
Limoniidae|[681]|GMAGT800-15|Diptera|Limoniidae||BOLD:ACY6575
Tachinidae|[682]|GMAGV081-15|Diptera|Tachinidae||BOLD:ACO2081
Sphaeroceridae|[683]|GMARS163-14|Diptera|Sphaeroceridae||BOLD:ACN8783
Ulidiidae|[684]|GMAGN1118-15|Diptera|Ulidiidae||BOLD:ACX3874
Diptera|[685]|GMAGE064-15|Diptera|||BOLD:ACX5904
Sarcophagidae|[686]|GMAGK024-15|Diptera|Sarcophagidae||BOLD:ACN6278
Sarcophagidae|[687]|GMARR1406-14|Diptera|Sarcophagidae||BOLD:ACO0000
Sarcophagidae|[688]|GMAGD117-15|Diptera|Sarcophagidae||BOLD:ACW8389
Sarcophagidae|[689]|GMARR1407-14|Diptera|Sarcophagidae||BOLD:ACO0561
Sarcophagidae|[690]|GMARS918-14|Diptera|Sarcophagidae||BOLD:ACO2076
Sarcophagidae|[691]|GMAGA194-15|Diptera|Sarcophagidae||BOLD:ACW2963
Sarcophagidae|[692]|GMAGJ1556-15|Diptera|Sarcophagidae||BOLD:ACY6043
Sarcophagidae|[693]|GMARR1411-14|Diptera|Sarcophagidae||BOLD:ACN9261
Sarcophagidae|[694]|GMAGP386-15|Diptera|Sarcophagidae||BOLD:AAA2287
Tricharaea|[695]|GMAGW104-15|Diptera|Sarcophagidae|Sarcophaginae|BOLD:ACY5042
Sarcophagidae|[696]|GMARS912-14|Diptera|Sarcophagidae||BOLD:ACO1721
Sarcophagidae|[697]|GMARK028-14|Diptera|Sarcophagidae||BOLD:ACN5550

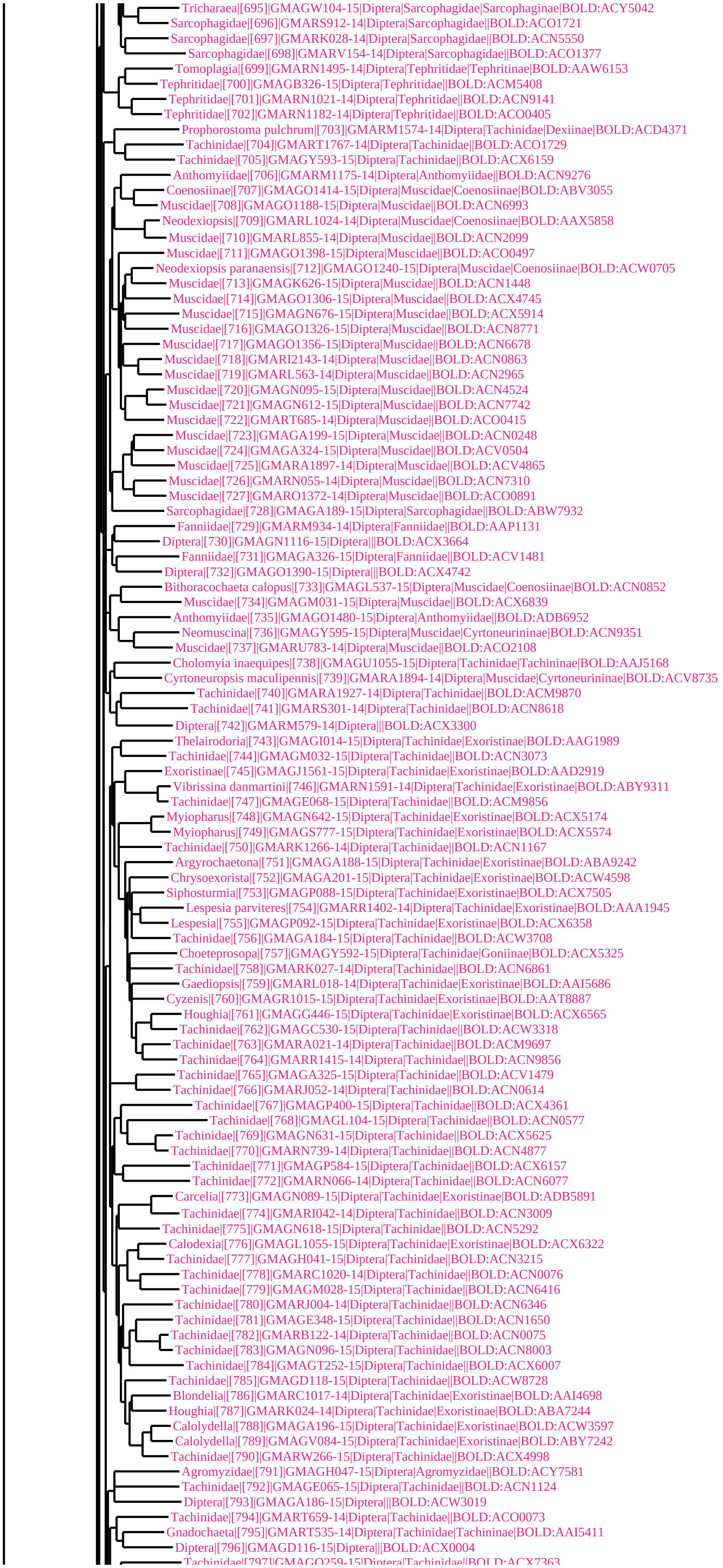
Tricharaea|[695]|GMAGW104-15|Diptera|Sarcophagidae|Sarcophaginae|BOLD:ACY5042
Sarcophagidae|[696]|GMARS912-14|Diptera|Sarcophagidae||BOLD:ACO1721
Sarcophagidae|[697]|GMARK028-14|Diptera|Sarcophagidae||BOLD:ACN5550
Sarcophagidae|[698]|GMARV154-14|Diptera|Sarcophagidae||BOLD:ACO1377
Tomoplagia|[699]|GMARN1495-14|Diptera|Tephritidae|Tephritinae|BOLD:AAW6153
Tephritidae|[700]|GMAGB326-15|Diptera|Tephritidae||BOLD:ACM5408
Tephritidae|[701]|GMARN1021-14|Diptera|Tephritidae||BOLD:ACN9141
Tephritidae|[702]|GMARN1182-14|Diptera|Tephritidae||BOLD:ACO0405
Prophorostoma pulchrum|[703]|GMARM1574-14|Diptera|Tachinidae|Dexiinae|BOLD:ACD4371
Tachinidae|[704]|GMART1767-14|Diptera|Tachinidae||BOLD:ACO1729
Tachinidae|[705]|GMAGY593-15|Diptera|Tachinidae||BOLD:ACX6159
Anthomyiidae|[706]|GMARM1175-14|Diptera|Anthomyiidae||BOLD:ACN9276
Coenosiinae|[707]|GMAGO1414-15|Diptera|Muscidae|Coenosiinae|BOLD:ABV3055
Muscidae|[708]|GMAGO1188-15|Diptera|Muscidae||BOLD:ACN6993
Neodexiopsis|[709]|GMARL1024-14|Diptera|Muscidae|Coenosiinae|BOLD:AAX5858
Muscidae|[710]|GMARL855-14|Diptera|Muscidae||BOLD:ACN2099
Muscidae|[711]|GMAGO1398-15|Diptera|Muscidae||BOLD:ACO0497
Neodexiopsis paranaensis|[712]|GMAGO1240-15|Diptera|Muscidae|Coenosiinae|BOLD:ACW0705
Muscidae|[713]|GMAGK626-15|Diptera|Muscidae||BOLD:ACN1448
Muscidae|[714]|GMAGO1306-15|Diptera|Muscidae||BOLD:ACX4745
Muscidae|[715]|GMAGN676-15|Diptera|Muscidae||BOLD:ACX5914
Muscidae|[716]|GMAGO1326-15|Diptera|Muscidae||BOLD:ACN8771
Muscidae|[717]|GMAGO1356-15|Diptera|Muscidae||BOLD:ACN6678
Muscidae|[718]|GMARI2143-14|Diptera|Muscidae||BOLD:ACN0863
Muscidae|[719]|GMARL563-14|Diptera|Muscidae||BOLD:ACN2965
Muscidae|[720]|GMAGN095-15|Diptera|Muscidae||BOLD:ACN4524
Muscidae|[721]|GMAGN612-15|Diptera|Muscidae||BOLD:ACN7742
Muscidae|[722]|GMART685-14|Diptera|Muscidae||BOLD:ACO0415
Muscidae|[723]|GMAGA199-15|Diptera|Muscidae||BOLD:ACN0248
Muscidae|[724]|GMAGA324-15|Diptera|Muscidae||BOLD:ACV0504
Muscidae|[725]|GMARA1897-14|Diptera|Muscidae||BOLD:ACV4865
Muscidae|[726]|GMARN055-14|Diptera|Muscidae||BOLD:ACN7310
Muscidae|[727]|GMARO1372-14|Diptera|Muscidae||BOLD:ACO0891
Sarcophagidae|[728]|GMAGA189-15|Diptera|Sarcophagidae||BOLD:ABW7932
Fanniidae|[729]|GMARM934-14|Diptera|Fanniidae||BOLD:AAP1131
Diptera|[730]|GMAGN1116-15|Diptera|||BOLD:ACX3664
Fanniidae|[731]|GMAGA326-15|Diptera|Fanniidae||BOLD:ACV1481
Diptera|[732]|GMAGO1390-15|Diptera|||BOLD:ACX4742
Bithoracochaeta calopus|[733]|GMAGL537-15|Diptera|Muscidae|Coenosiinae|BOLD:ACN0852
Muscidae|[734]|GMAGM031-15|Diptera|Muscidae||BOLD:ACX6839
Anthomyiidae|[735]|GMAGO1480-15|Diptera|Anthomyiidae||BOLD:ADB6952
Neomuscina|[736]|GMAGY595-15|Diptera|Muscidae|Cyrtoneurininae|BOLD:ACN9351
Muscidae|[737]|GMARU783-14|Diptera|Muscidae||BOLD:ACO2108
Cholomyia inaequipes|[738]|GMAGU1055-15|Diptera|Tachinidae|Tachininae|BOLD:AAJ5168
Cyrtoneuropsis maculipennis|[739]|GMARA1894-14|Diptera|Muscidae|Cyrtoneurininae|BOLD:ACV8735
Tachinidae|[740]|GMARA1927-14|Diptera|Tachinidae||BOLD:ACM9870
Tachinidae|[741]|GMARS301-14|Diptera|Tachinidae||BOLD:ACN8618
Diptera|[742]|GMARM579-14|Diptera|||BOLD:ACX3300
Thelairodoria|[743]|GMAGI014-15|Diptera|Tachinidae|Exoristinae|BOLD:AAG1989
Tachinidae|[744]|GMAGM032-15|Diptera|Tachinidae||BOLD:ACN3073
Exoristinae|[745]|GMAGJ1561-15|Diptera|Tachinidae|Exoristinae|BOLD:AAD2919
Vibrissina danmartini|[746]|GMARN1591-14|Diptera|Tachinidae|Exoristinae|BOLD:ABY9311
Tachinidae|[747]|GMAGE068-15|Diptera|Tachinidae||BOLD:ACM9856
Myiopharus|[748]|GMAGN642-15|Diptera|Tachinidae|Exoristinae|BOLD:ACX5174
Myiopharus|[749]|GMAGS777-15|Diptera|Tachinidae|Exoristinae|BOLD:ACX5574
Tachinidae|[750]|GMARK1266-14|Diptera|Tachinidae||BOLD:ACN1167
Argyrochaetona|[751]|GMAGA188-15|Diptera|Tachinidae|Exoristinae|BOLD:ABA9242
Chrysoexorista|[752]|GMAGA201-15|Diptera|Tachinidae|Exoristinae|BOLD:ACW4598
Siphosturmia|[753]|GMAGP088-15|Diptera|Tachinidae|Exoristinae|BOLD:ACX7505
Lespesia parviteres|[754]|GMARR1402-14|Diptera|Tachinidae|Exoristinae|BOLD:AAA1945
Lespesia|[755]|GMAGP092-15|Diptera|Tachinidae|Exoristinae|BOLD:ACX6358
Tachinidae|[756]|GMAGA184-15|Diptera|Tachinidae||BOLD:ACW3708
Choeteprosopa|[757]|GMAGY592-15|Diptera|Tachinidae|Goniinae|BOLD:ACX5325
Tachinidae|[758]|GMARK027-14|Diptera|Tachinidae||BOLD:ACN6861
Gaediopsis|[759]|GMARL018-14|Diptera|Tachinidae|Exoristinae|BOLD:AAI5686
Cyzenis|[760]|GMAGR1015-15|Diptera|Tachinidae|Exoristinae|BOLD:AAT8887
Houghia|[761]|GMAGG446-15|Diptera|Tachinidae|Exoristinae|BOLD:ACX6565
Tachinidae|[762]|GMAGC530-15|Diptera|Tachinidae||BOLD:ACW3318
Tachinidae|[763]|GMARA021-14|Diptera|Tachinidae||BOLD:ACM9697
Tachinidae|[764]|GMARR1415-14|Diptera|Tachinidae||BOLD:ACN9856
Tachinidae|[765]|GMAGA325-15|Diptera|Tachinidae||BOLD:ACV1479
Tachinidae|[766]|GMARJ052-14|Diptera|Tachinidae||BOLD:ACN0614
Tachinidae|[767]|GMAGP400-15|Diptera|Tachinidae||BOLD:ACX4361
Tachinidae|[768]|GMAGL104-15|Diptera|Tachinidae||BOLD:ACN0577
Tachinidae|[769]|GMAGN631-15|Diptera|Tachinidae||BOLD:ACX5625
Tachinidae|[770]|GMARN739-14|Diptera|Tachinidae||BOLD:ACN4877
Tachinidae|[771]|GMAGP584-15|Diptera|Tachinidae||BOLD:ACX6157
Tachinidae|[772]|GMARN066-14|Diptera|Tachinidae||BOLD:ACN6077
Carcelia|[773]|GMAGN089-15|Diptera|Tachinidae|Exoristinae|BOLD:ADB5891
Tachinidae|[774]|GMARI042-14|Diptera|Tachinidae||BOLD:ACN3009
Tachinidae|[775]|GMAGN618-15|Diptera|Tachinidae||BOLD:ACN5292
Calodexia|[776]|GMAGL1055-15|Diptera|Tachinidae|Exoristinae|BOLD:ACX6322
Tachinidae|[777]|GMAGH041-15|Diptera|Tachinidae||BOLD:ACN3215
Tachinidae|[778]|GMARC1020-14|Diptera|Tachinidae||BOLD:ACN0076
Tachinidae|[779]|GMAGM028-15|Diptera|Tachinidae||BOLD:ACN6416
Tachinidae|[780]|GMARJ004-14|Diptera|Tachinidae||BOLD:ACN6346
Tachinidae|[781]|GMAGE348-15|Diptera|Tachinidae||BOLD:ACN1650
Tachinidae|[782]|GMARB122-14|Diptera|Tachinidae||BOLD:ACN0075
Tachinidae|[783]|GMAGN096-15|Diptera|Tachinidae||BOLD:ACN8003
Tachinidae|[784]|GMAGT252-15|Diptera|Tachinidae||BOLD:ACX6007
Tachinidae|[785]|GMAGD118-15|Diptera|Tachinidae||BOLD:ACW8728
Blondelia|[786]|GMARC1017-14|Diptera|Tachinidae|Exoristinae|BOLD:AAI4698
Houghia|[787]|GMARK024-14|Diptera|Tachinidae|Exoristinae|BOLD:ABA7244
Calolydella|[788]|GMAGA196-15|Diptera|Tachinidae|Exoristinae|BOLD:ACW3597
Calolydella|[789]|GMAGV084-15|Diptera|Tachinidae|Exoristinae|BOLD:ABY7242
Tachinidae|[790]|GMARW266-15|Diptera|Tachinidae||BOLD:ACX4998
Agromyzidae|[791]|GMAGH047-15|Diptera|Agromyzidae||BOLD:ACY7581
Tachinidae|[792]|GMAGE065-15|Diptera|Tachinidae||BOLD:ACN1124
Diptera|[793]|GMAGA186-15|Diptera|||BOLD:ACW3019
Tachinidae|[794]|GMART659-14|Diptera|Tachinidae||BOLD:ACO0073
Gnadochaeta|[795]|GMART535-14|Diptera|Tachinidae|Tachininae|BOLD:AAI5411
Diptera|[796]|GMAGD116-15|Diptera|||BOLD:ACX0004
Tachinidae|[797]|GMAGO259-15|Diptera|Tachinidae||BOLD:ACX7363

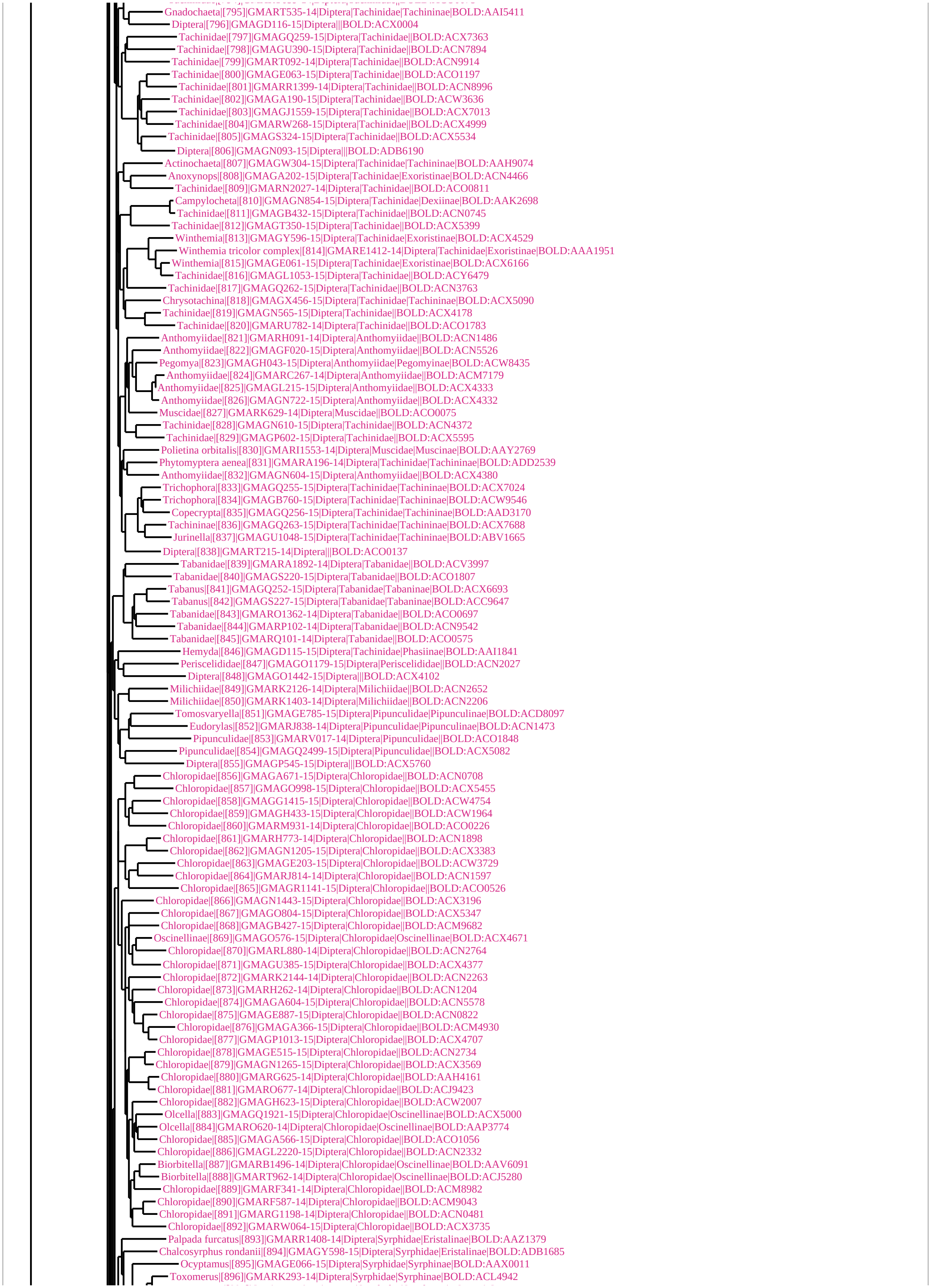
Gnadochaeta|[795]|GMART535-14|Diptera|Tachinidae|Tachininae|BOLD:AAI5411
Diptera|[796]|GMAGD116-15|Diptera|||BOLD:ACX0004
Tachinidae|[797]|GMAGQ259-15|Diptera|Tachinidae||BOLD:ACX7363
Tachinidae|[798]|GMAGU390-15|Diptera|Tachinidae||BOLD:ACN7894
Tachinidae|[799]|GMART092-14|Diptera|Tachinidae||BOLD:ACN9914
Tachinidae|[800]|GMAGE063-15|Diptera|Tachinidae||BOLD:ACO1197
Tachinidae|[801]|GMARR1399-14|Diptera|Tachinidae||BOLD:ACN8996
Tachinidae|[802]|GMAGA190-15|Diptera|Tachinidae||BOLD:ACW3636
Tachinidae|[803]|GMAGJ1559-15|Diptera|Tachinidae||BOLD:ACX7013
Tachinidae|[804]|GMARW268-15|Diptera|Tachinidae||BOLD:ACX4999
Tachinidae|[805]|GMAGS324-15|Diptera|Tachinidae||BOLD:ACX5534
Diptera|[806]|GMAGN093-15|Diptera|||BOLD:ADB6190
Actinochaeta|[807]|GMAGW304-15|Diptera|Tachinidae|Tachininae|BOLD:AAH9074
Anoxynops|[808]|GMAGA202-15|Diptera|Tachinidae|Exoristinae|BOLD:ACN4466
Tachinidae|[809]|GMARN2027-14|Diptera|Tachinidae||BOLD:ACO0811
Campylocheta|[810]|GMAGN854-15|Diptera|Tachinidae|Dexiinae|BOLD:AAK2698
Tachinidae|[811]|GMAGB432-15|Diptera|Tachinidae||BOLD:ACN0745
Tachinidae|[812]|GMAGT350-15|Diptera|Tachinidae||BOLD:ACX5399
Winthemia|[813]|GMAGY596-15|Diptera|Tachinidae|Exoristinae|BOLD:ACX4529
Winthemia tricolor complex|[814]|GMARE1412-14|Diptera|Tachinidae|Exoristinae|BOLD:AAA1951
Winthemia|[815]|GMAGE061-15|Diptera|Tachinidae|Exoristinae|BOLD:ACX6166
Tachinidae|[816]|GMAGL1053-15|Diptera|Tachinidae||BOLD:ACY6479
Tachinidae|[817]|GMAGQ262-15|Diptera|Tachinidae||BOLD:ACN3763
Chrysotachina|[818]|GMAGX456-15|Diptera|Tachinidae|Tachininae|BOLD:ACX5090
Tachinidae|[819]|GMAGN565-15|Diptera|Tachinidae||BOLD:ACX4178
Tachinidae|[820]|GMARU782-14|Diptera|Tachinidae||BOLD:ACO1783
Anthomyiidae|[821]|GMARH091-14|Diptera|Anthomyiidae||BOLD:ACN1486
Anthomyiidae|[822]|GMAGF020-15|Diptera|Anthomyiidae||BOLD:ACN5526
Pegomya|[823]|GMAGH043-15|Diptera|Anthomyiidae|Pegomyinae|BOLD:ACW8435
Anthomyiidae|[824]|GMARC267-14|Diptera|Anthomyiidae||BOLD:ACM7179
Anthomyiidae|[825]|GMAGL215-15|Diptera|Anthomyiidae||BOLD:ACX4333
Anthomyiidae|[826]|GMAGN722-15|Diptera|Anthomyiidae||BOLD:ACX4332
Muscidae|[827]|GMARK629-14|Diptera|Muscidae||BOLD:ACO0075
Tachinidae|[828]|GMAGN610-15|Diptera|Tachinidae||BOLD:ACN4372
Tachinidae|[829]|GMAGP602-15|Diptera|Tachinidae||BOLD:ACX5595
Polietina orbitalis|[830]|GMARI1553-14|Diptera|Muscidae|Muscinae|BOLD:AAY2769
Phytomyptera aenea|[831]|GMARA196-14|Diptera|Tachinidae|Tachininae|BOLD:ADD2539
Anthomyiidae|[832]|GMAGN604-15|Diptera|Anthomyiidae||BOLD:ACX4380
Trichophora|[833]|GMAGQ255-15|Diptera|Tachinidae|Tachininae|BOLD:ACX7024
Trichophora|[834]|GMAGB760-15|Diptera|Tachinidae|Tachininae|BOLD:ACW9546
Copecrypta|[835]|GMAGQ256-15|Diptera|Tachinidae|Tachininae|BOLD:AAD3170
Tachininae|[836]|GMAGQ263-15|Diptera|Tachinidae|Tachininae|BOLD:ACX7688
Jurinella|[837]|GMAGU1048-15|Diptera|Tachinidae|Tachininae|BOLD:ABV1665
Diptera|[838]|GMART215-14|Diptera|||BOLD:ACO0137
Tabanidae|[839]|GMARA1892-14|Diptera|Tabanidae||BOLD:ACV3997
Tabanidae|[840]|GMAGS220-15|Diptera|Tabanidae||BOLD:ACO1807
Tabanus|[841]|GMAGQ252-15|Diptera|Tabanidae|Tabaninae|BOLD:ACX6693
Tabanus|[842]|GMAGS227-15|Diptera|Tabanidae|Tabaninae|BOLD:ACC9647
Tabanidae|[843]|GMARO1362-14|Diptera|Tabanidae||BOLD:ACO0697
Tabanidae|[844]|GMARP102-14|Diptera|Tabanidae||BOLD:ACN9542
Tabanidae|[845]|GMARQ101-14|Diptera|Tabanidae||BOLD:ACO0575
Hemyda|[846]|GMAGD115-15|Diptera|Tachinidae|Phasiinae|BOLD:AAI1841
Periscelididae|[847]|GMAGO1179-15|Diptera|Periscelididae||BOLD:ACN2027
Diptera|[848]|GMAGO1442-15|Diptera|||BOLD:ACX4102
Milichiidae|[849]|GMARK2126-14|Diptera|Milichiidae||BOLD:ACN2652
Milichiidae|[850]|GMARK1403-14|Diptera|Milichiidae||BOLD:ACN2206
Tomosvaryella|[851]|GMAGE785-15|Diptera|Pipunculidae|Pipunculinae|BOLD:ACD8097
Eudorylas|[852]|GMARJ838-14|Diptera|Pipunculidae|Pipunculinae|BOLD:ACN1473
Pipunculidae|[853]|GMARV017-14|Diptera|Pipunculidae||BOLD:ACO1848
Pipunculidae|[854]|GMAGQ2499-15|Diptera|Pipunculidae||BOLD:ACX5082
Diptera|[855]|GMAGP545-15|Diptera|||BOLD:ACX5760
Chloropidae|[856]|GMAGA671-15|Diptera|Chloropidae||BOLD:ACN0708
Chloropidae|[857]|GMAGO998-15|Diptera|Chloropidae||BOLD:ACX5455
Chloropidae|[858]|GMAGG1415-15|Diptera|Chloropidae||BOLD:ACW4754
Chloropidae|[859]|GMAGH433-15|Diptera|Chloropidae||BOLD:ACW1964
Chloropidae|[860]|GMARM931-14|Diptera|Chloropidae||BOLD:ACO0226
Chloropidae|[861]|GMARH773-14|Diptera|Chloropidae||BOLD:ACN1898
Chloropidae|[862]|GMAGN1205-15|Diptera|Chloropidae||BOLD:ACX3383
Chloropidae|[863]|GMAGE203-15|Diptera|Chloropidae||BOLD:ACW3729
Chloropidae|[864]|GMARJ814-14|Diptera|Chloropidae||BOLD:ACN1597
Chloropidae|[865]|GMAGR1141-15|Diptera|Chloropidae||BOLD:ACO0526
Chloropidae|[866]|GMAGN1443-15|Diptera|Chloropidae||BOLD:ACX3196
Chloropidae|[867]|GMAGO804-15|Diptera|Chloropidae||BOLD:ACX5347
Chloropidae|[868]|GMAGB427-15|Diptera|Chloropidae||BOLD:ACM9682
Oscinellinae|[869]|GMAGO576-15|Diptera|Chloropidae|Oscinellinae|BOLD:ACX4671
Chloropidae|[870]|GMARL880-14|Diptera|Chloropidae||BOLD:ACN2764
Chloropidae|[871]|GMAGU385-15|Diptera|Chloropidae||BOLD:ACX4377
Chloropidae|[872]|GMARK2144-14|Diptera|Chloropidae||BOLD:ACN2263
Chloropidae|[873]|GMARH262-14|Diptera|Chloropidae||BOLD:ACN1204
Chloropidae|[874]|GMAGA604-15|Diptera|Chloropidae||BOLD:ACN5578
Chloropidae|[875]|GMAGE887-15|Diptera|Chloropidae||BOLD:ACN0822
Chloropidae|[876]|GMAGA366-15|Diptera|Chloropidae||BOLD:ACM4930
Chloropidae|[877]|GMAGP1013-15|Diptera|Chloropidae||BOLD:ACX4707
Chloropidae|[878]|GMAGE515-15|Diptera|Chloropidae||BOLD:ACN2734
Chloropidae|[879]|GMAGN1265-15|Diptera|Chloropidae||BOLD:ACX3569
Chloropidae|[880]|GMARG625-14|Diptera|Chloropidae||BOLD:AAH4161
Chloropidae|[881]|GMARO677-14|Diptera|Chloropidae||BOLD:ACJ9423
Chloropidae|[882]|GMAGH623-15|Diptera|Chloropidae||BOLD:ACW2007
Olcella|[883]|GMAGQ1921-15|Diptera|Chloropidae|Oscinellinae|BOLD:ACX5000
Olcella|[884]|GMARO620-14|Diptera|Chloropidae|Oscinellinae|BOLD:AAP3774
Chloropidae|[885]|GMAGA566-15|Diptera|Chloropidae||BOLD:ACO1056
Chloropidae|[886]|GMAGL2220-15|Diptera|Chloropidae||BOLD:ACN2332
Biorbitella|[887]|GMARB1496-14|Diptera|Chloropidae|Oscinellinae|BOLD:AAV6091
Biorbitella|[888]|GMART962-14|Diptera|Chloropidae|Oscinellinae|BOLD:ACJ5280
Chloropidae|[889]|GMARF341-14|Diptera|Chloropidae||BOLD:ACM8982
Chloropidae|[890]|GMARF587-14|Diptera|Chloropidae||BOLD:ACM9043
Chloropidae|[891]|GMARG1198-14|Diptera|Chloropidae||BOLD:ACN0481
Chloropidae|[892]|GMARW064-15|Diptera|Chloropidae||BOLD:ACX3735
Palpada furcatus|[893]|GMARR1408-14|Diptera|Syrphidae|Eristalinae|BOLD:AAZ1379
Chalcosyrphus rondanii|[894]|GMAGY598-15|Diptera|Syrphidae|Eristalinae|BOLD:ADB1685
Ocyptamus|[895]|GMAGE066-15|Diptera|Syrphidae|Syrphinae|BOLD:AAX0011
Toxomerus|[896]|GMARK293-14|Diptera|Syrphidae|Syrphinae|BOLD:ACL4942

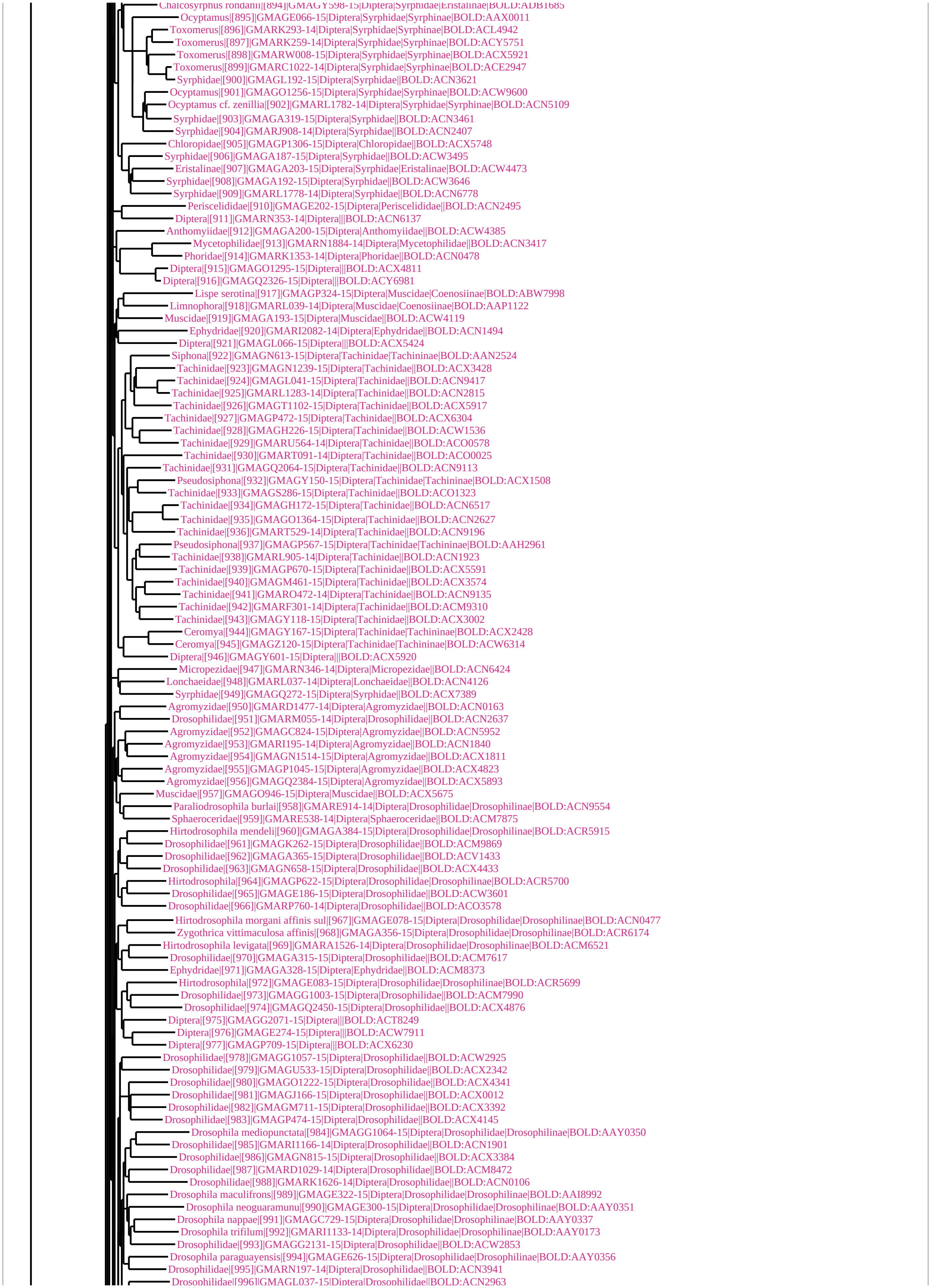
Chalcosyrphus rondanii|[894]|GMAGY598-15|Diptera|Syrphidae|Eristalinae|BOLD:ADB1685
Ocyptamus|[895]|GMAGE066-15|Diptera|Syrphidae|Syrphinae|BOLD:AAX0011
Toxomerus|[896]|GMARK293-14|Diptera|Syrphidae|Syrphinae|BOLD:ACL4942
Toxomerus|[897]|GMARK259-14|Diptera|Syrphidae|Syrphinae|BOLD:ACY5751
Toxomerus|[898]|GMARW008-15|Diptera|Syrphidae|Syrphinae|BOLD:ACX5921
Toxomerus|[899]|GMARC1022-14|Diptera|Syrphidae|Syrphinae|BOLD:ACE2947
Syrphidae|[900]|GMAGL192-15|Diptera|Syrphidae||BOLD:ACN3621
Ocyptamus|[901]|GMAGO1256-15|Diptera|Syrphidae|Syrphinae|BOLD:ACW9600
Ocyptamus cf. zenillia|[902]|GMARL1782-14|Diptera|Syrphidae|Syrphinae|BOLD:ACN5109
Syrphidae|[903]|GMAGA319-15|Diptera|Syrphidae||BOLD:ACN3461
Syrphidae|[904]|GMARJ908-14|Diptera|Syrphidae||BOLD:ACN2407
Chloropidae|[905]|GMAGP1306-15|Diptera|Chloropidae||BOLD:ACX5748
Syrphidae|[906]|GMAGA187-15|Diptera|Syrphidae||BOLD:ACW3495
Eristalinae|[907]|GMAGA203-15|Diptera|Syrphidae|Eristalinae|BOLD:ACW4473
Syrphidae|[908]|GMAGA192-15|Diptera|Syrphidae||BOLD:ACW3646
Syrphidae|[909]|GMARL1778-14|Diptera|Syrphidae||BOLD:ACN6778
Periscelididae|[910]|GMAGE202-15|Diptera|Periscelididae||BOLD:ACN2495
Diptera|[911]|GMARN353-14|Diptera|||BOLD:ACN6137
Anthomyiidae|[912]|GMAGA200-15|Diptera|Anthomyiidae||BOLD:ACW4385
Mycetophilidae|[913]|GMARN1884-14|Diptera|Mycetophilidae||BOLD:ACN3417
Phoridae|[914]|GMARK1353-14|Diptera|Phoridae||BOLD:ACN0478
Diptera|[915]|GMAGO1295-15|Diptera|||BOLD:ACX4811
Diptera|[916]|GMAGQ2326-15|Diptera|||BOLD:ACY6981
Lispe serotina|[917]|GMAGP324-15|Diptera|Muscidae|Coenosiinae|BOLD:ABW7998
Limnophora|[918]|GMARL039-14|Diptera|Muscidae|Coenosiinae|BOLD:AAP1122
Muscidae|[919]|GMAGA193-15|Diptera|Muscidae||BOLD:ACW4119
Ephydridae|[920]|GMARI2082-14|Diptera|Ephydridae||BOLD:ACN1494
Diptera|[921]|GMAGL066-15|Diptera|||BOLD:ACX5424
Siphona|[922]|GMAGN613-15|Diptera|Tachinidae|Tachininae|BOLD:AAN2524
Tachinidae|[923]|GMAGN1239-15|Diptera|Tachinidae||BOLD:ACX3428
Tachinidae|[924]|GMAGL041-15|Diptera|Tachinidae||BOLD:ACN9417
Tachinidae|[925]|GMARL1283-14|Diptera|Tachinidae||BOLD:ACN2815
Tachinidae|[926]|GMAGT1102-15|Diptera|Tachinidae||BOLD:ACX5917
Tachinidae|[927]|GMAGP472-15|Diptera|Tachinidae||BOLD:ACX6304
Tachinidae|[928]|GMAGH226-15|Diptera|Tachinidae||BOLD:ACW1536
Tachinidae|[929]|GMARU564-14|Diptera|Tachinidae||BOLD:ACO0578
Tachinidae|[930]|GMART091-14|Diptera|Tachinidae||BOLD:ACO0025
Tachinidae|[931]|GMAGQ2064-15|Diptera|Tachinidae||BOLD:ACN9113
Pseudosiphona|[932]|GMAGY150-15|Diptera|Tachinidae|Tachininae|BOLD:ACX1508
Tachinidae|[933]|GMAGS286-15|Diptera|Tachinidae||BOLD:ACO1323
Tachinidae|[934]|GMAGH172-15|Diptera|Tachinidae||BOLD:ACN6517
Tachinidae|[935]|GMAGO1364-15|Diptera|Tachinidae||BOLD:ACN2627
Tachinidae|[936]|GMART529-14|Diptera|Tachinidae||BOLD:ACN9196
Pseudosiphona|[937]|GMAGP567-15|Diptera|Tachinidae|Tachininae|BOLD:AAH2961
Tachinidae|[938]|GMARL905-14|Diptera|Tachinidae||BOLD:ACN1923
Tachinidae|[939]|GMAGP670-15|Diptera|Tachinidae||BOLD:ACX5591
Tachinidae|[940]|GMAGM461-15|Diptera|Tachinidae||BOLD:ACX3574
Tachinidae|[941]|GMARO472-14|Diptera|Tachinidae||BOLD:ACN9135
Tachinidae|[942]|GMARF301-14|Diptera|Tachinidae||BOLD:ACM9310
Tachinidae|[943]|GMAGY118-15|Diptera|Tachinidae||BOLD:ACX3002
Ceromya|[944]|GMAGY167-15|Diptera|Tachinidae|Tachininae|BOLD:ACX2428
Ceromya|[945]|GMAGZ120-15|Diptera|Tachinidae|Tachininae|BOLD:ACW6314
Diptera|[946]|GMAGY601-15|Diptera|||BOLD:ACX5920
Micropezidae|[947]|GMARN346-14|Diptera|Micropezidae||BOLD:ACN6424
Lonchaeidae|[948]|GMARL037-14|Diptera|Lonchaeidae||BOLD:ACN4126
Syrphidae|[949]|GMAGQ272-15|Diptera|Syrphidae||BOLD:ACX7389
Agromyzidae|[950]|GMARD1477-14|Diptera|Agromyzidae||BOLD:ACN0163
Drosophilidae|[951]|GMARM055-14|Diptera|Drosophilidae||BOLD:ACN2637
Agromyzidae|[952]|GMAGC824-15|Diptera|Agromyzidae||BOLD:ACN5952
Agromyzidae|[953]|GMARI195-14|Diptera|Agromyzidae||BOLD:ACN1840
Agromyzidae|[954]|GMAGN1514-15|Diptera|Agromyzidae||BOLD:ACX1811
Agromyzidae|[955]|GMAGP1045-15|Diptera|Agromyzidae||BOLD:ACX4823
Agromyzidae|[956]|GMAGQ2384-15|Diptera|Agromyzidae||BOLD:ACX5893
Muscidae|[957]|GMAGO946-15|Diptera|Muscidae||BOLD:ACX5675
Paraliodrosophila burlai|[958]|GMARE914-14|Diptera|Drosophilidae|Drosophilinae|BOLD:ACN9554
Sphaeroceridae|[959]|GMARE538-14|Diptera|Sphaeroceridae||BOLD:ACM7875
Hirtodrosophila mendeli|[960]|GMAGA384-15|Diptera|Drosophilidae|Drosophilinae|BOLD:ACR5915
Drosophilidae|[961]|GMAGK262-15|Diptera|Drosophilidae||BOLD:ACM9869
Drosophilidae|[962]|GMAGA365-15|Diptera|Drosophilidae||BOLD:ACV1433
Drosophilidae|[963]|GMAGN658-15|Diptera|Drosophilidae||BOLD:ACX4433
Hirtodrosophila|[964]|GMAGP622-15|Diptera|Drosophilidae|Drosophilinae|BOLD:ACR5700
Drosophilidae|[965]|GMAGE186-15|Diptera|Drosophilidae||BOLD:ACW3601
Drosophilidae|[966]|GMARP760-14|Diptera|Drosophilidae||BOLD:ACO3578
Hirtodrosophila morgani affinis sul|[967]|GMAGE078-15|Diptera|Drosophilidae|Drosophilinae|BOLD:ACN0477
Zygothrica vittimaculosa affinis|[968]|GMAGA356-15|Diptera|Drosophilidae|Drosophilinae|BOLD:ACR6174
Hirtodrosophila levigata|[969]|GMARA1526-14|Diptera|Drosophilidae|Drosophilinae|BOLD:ACM6521
Drosophilidae|[970]|GMAGA315-15|Diptera|Drosophilidae||BOLD:ACM7617
Ephydridae|[971]|GMAGA328-15|Diptera|Ephydridae||BOLD:ACM8373
Hirtodrosophila|[972]|GMAGE083-15|Diptera|Drosophilidae|Drosophilinae|BOLD:ACR5699
Drosophilidae|[973]|GMAGG1003-15|Diptera|Drosophilidae||BOLD:ACM7990
Drosophilidae|[974]|GMAGQ2450-15|Diptera|Drosophilidae||BOLD:ACX4876
Diptera|[975]|GMAGG2071-15|Diptera|||BOLD:ACT8249
Diptera|[976]|GMAGE274-15|Diptera|||BOLD:ACW7911
Diptera|[977]|GMAGP709-15|Diptera|||BOLD:ACX6230
Drosophilidae|[978]|GMAGG1057-15|Diptera|Drosophilidae||BOLD:ACW2925
Drosophilidae|[979]|GMAGU533-15|Diptera|Drosophilidae||BOLD:ACX2342
Drosophilidae|[980]|GMAGO1222-15|Diptera|Drosophilidae||BOLD:ACX4341
Drosophilidae|[981]|GMAGJ166-15|Diptera|Drosophilidae||BOLD:ACX0012
Drosophilidae|[982]|GMAGM711-15|Diptera|Drosophilidae||BOLD:ACX3392
Drosophilidae|[983]|GMAGP474-15|Diptera|Drosophilidae||BOLD:ACX4145
Drosophila mediopunctata|[984]|GMAGG1064-15|Diptera|Drosophilidae|Drosophilinae|BOLD:AAY0350
Drosophilidae|[985]|GMARI1166-14|Diptera|Drosophilidae||BOLD:ACN1901
Drosophilidae|[986]|GMAGN815-15|Diptera|Drosophilidae||BOLD:ACX3384
Drosophilidae|[987]|GMARD1029-14|Diptera|Drosophilidae||BOLD:ACM8472
Drosophilidae|[988]|GMARK1626-14|Diptera|Drosophilidae||BOLD:ACN0106
Drosophila maculifrons|[989]|GMAGE322-15|Diptera|Drosophilidae|Drosophilinae|BOLD:AAI8992
Drosophila neoguaramunu|[990]|GMAGE300-15|Diptera|Drosophilidae|Drosophilinae|BOLD:AAY0351
Drosophila nappae|[991]|GMAGC729-15|Diptera|Drosophilidae|Drosophilinae|BOLD:AAY0337
Drosophila trifilum|[992]|GMARI1133-14|Diptera|Drosophilidae|Drosophilinae|BOLD:AAY0173
Drosophilidae|[993]|GMAGG2131-15|Diptera|Drosophilidae||BOLD:ACW2853
Drosophila paraguayensis|[994]|GMAGE626-15|Diptera|Drosophilidae|Drosophilinae|BOLD:AAY0356
Drosophilidae|[995]|GMARN197-14|Diptera|Drosophilidae||BOLD:ACN3941
Drosophilidae|[996]|GMAGL037-15|Diptera|Drosophilidae||BOLD:ACN2963

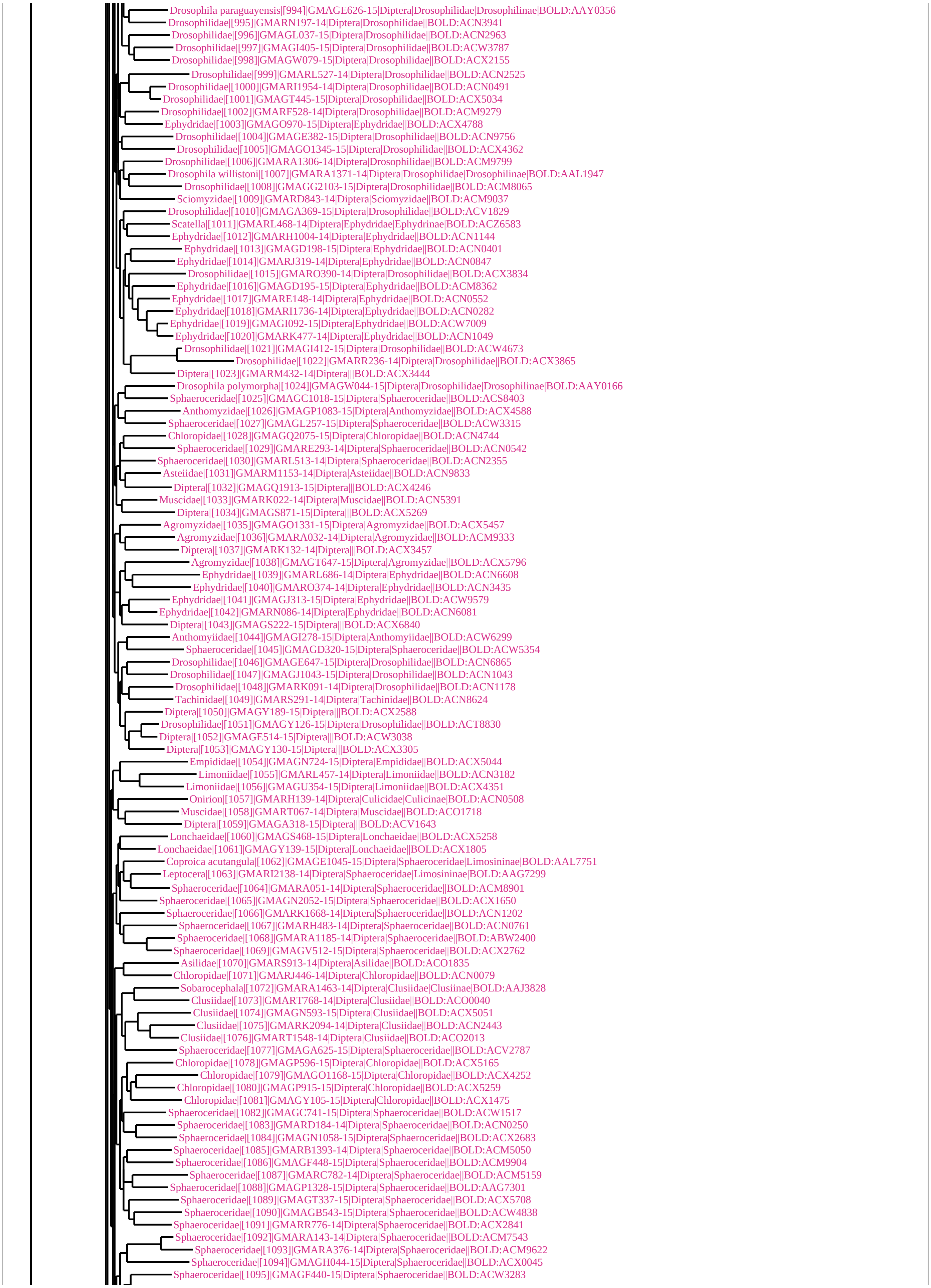
Drosophila paraguayensis|[994]|GMAGE626-15|Diptera|Drosophilidae|Drosophilinae|BOLD:AAY0356
Drosophilidae|[995]|GMARN197-14|Diptera|Drosophilidae||BOLD:ACN3941
Drosophilidae|[996]|GMAGL037-15|Diptera|Drosophilidae||BOLD:ACN2963
Drosophilidae|[997]|GMAGI405-15|Diptera|Drosophilidae||BOLD:ACW3787
Drosophilidae|[998]|GMAGW079-15|Diptera|Drosophilidae||BOLD:ACX2155
Drosophilidae|[999]|GMARL527-14|Diptera|Drosophilidae||BOLD:ACN2525
Drosophilidae|[1000]|GMARI1954-14|Diptera|Drosophilidae||BOLD:ACN0491
Drosophilidae|[1001]|GMAGT445-15|Diptera|Drosophilidae||BOLD:ACX5034
Drosophilidae|[1002]|GMARF528-14|Diptera|Drosophilidae||BOLD:ACM9279
Ephydridae|[1003]|GMAGO970-15|Diptera|Ephydridae||BOLD:ACX4788
Drosophilidae|[1004]|GMAGE382-15|Diptera|Drosophilidae||BOLD:ACN9756
Drosophilidae|[1005]|GMAGO1345-15|Diptera|Drosophilidae||BOLD:ACX4362
Drosophilidae|[1006]|GMARA1306-14|Diptera|Drosophilidae||BOLD:ACM9799
Drosophila willistoni|[1007]|GMARA1371-14|Diptera|Drosophilidae|Drosophilinae|BOLD:AAL1947
Drosophilidae|[1008]|GMAGG2103-15|Diptera|Drosophilidae||BOLD:ACM8065
Sciomyzidae|[1009]|GMARD843-14|Diptera|Sciomyzidae||BOLD:ACM9037
Drosophilidae|[1010]|GMAGA369-15|Diptera|Drosophilidae||BOLD:ACV1829
Scatella|[1011]|GMARL468-14|Diptera|Ephydridae|Ephydrinae|BOLD:ACZ6583
Ephydridae|[1012]|GMARH1004-14|Diptera|Ephydridae||BOLD:ACN1144
Ephydridae|[1013]|GMAGD198-15|Diptera|Ephydridae||BOLD:ACN0401
Ephydridae|[1014]|GMARJ319-14|Diptera|Ephydridae||BOLD:ACN0847
Drosophilidae|[1015]|GMARO390-14|Diptera|Drosophilidae||BOLD:ACX3834
Ephydridae|[1016]|GMAGD195-15|Diptera|Ephydridae||BOLD:ACM8362
Ephydridae|[1017]|GMARE148-14|Diptera|Ephydridae||BOLD:ACN0552
Ephydridae|[1018]|GMARI1736-14|Diptera|Ephydridae||BOLD:ACN0282
Ephydridae|[1019]|GMAGI092-15|Diptera|Ephydridae||BOLD:ACW7009
Ephydridae|[1020]|GMARK477-14|Diptera|Ephydridae||BOLD:ACN1049
Drosophilidae|[1021]|GMAGI412-15|Diptera|Drosophilidae||BOLD:ACW4673
Drosophilidae|[1022]|GMARR236-14|Diptera|Drosophilidae||BOLD:ACX3865
Diptera|[1023]|GMARM432-14|Diptera|||BOLD:ACX3444
Drosophila polymorpha|[1024]|GMAGW044-15|Diptera|Drosophilidae|Drosophilinae|BOLD:AAY0166
Sphaeroceridae|[1025]|GMAGC1018-15|Diptera|Sphaeroceridae||BOLD:ACS8403
Anthomyzidae|[1026]|GMAGP1083-15|Diptera|Anthomyzidae||BOLD:ACX4588
Sphaeroceridae|[1027]|GMAGL257-15|Diptera|Sphaeroceridae||BOLD:ACW3315
Chloropidae|[1028]|GMAGQ2075-15|Diptera|Chloropidae||BOLD:ACN4744
Sphaeroceridae|[1029]|GMARE293-14|Diptera|Sphaeroceridae||BOLD:ACN0542
Sphaeroceridae|[1030]|GMARL513-14|Diptera|Sphaeroceridae||BOLD:ACN2355
Asteiidae|[1031]|GMARM1153-14|Diptera|Asteiidae||BOLD:ACN9833
Diptera|[1032]|GMAGQ1913-15|Diptera|||BOLD:ACX4246
Muscidae|[1033]|GMARK022-14|Diptera|Muscidae||BOLD:ACN5391
Diptera|[1034]|GMAGS871-15|Diptera|||BOLD:ACX5269
Agromyzidae|[1035]|GMAGO1331-15|Diptera|Agromyzidae||BOLD:ACX5457
Agromyzidae|[1036]|GMARA032-14|Diptera|Agromyzidae||BOLD:ACM9333
Diptera|[1037]|GMARK132-14|Diptera|||BOLD:ACX3457
Agromyzidae|[1038]|GMAGT647-15|Diptera|Agromyzidae||BOLD:ACX5796
Ephydridae|[1039]|GMARL686-14|Diptera|Ephydridae||BOLD:ACN6608
Ephydridae|[1040]|GMARO374-14|Diptera|Ephydridae||BOLD:ACN3435
Ephydridae|[1041]|GMAGJ313-15|Diptera|Ephydridae||BOLD:ACW9579
Ephydridae|[1042]|GMARN086-14|Diptera|Ephydridae||BOLD:ACN6081
Diptera|[1043]|GMAGS222-15|Diptera|||BOLD:ACX6840
Anthomyiidae|[1044]|GMAGI278-15|Diptera|Anthomyiidae||BOLD:ACW6299
Sphaeroceridae|[1045]|GMAGD320-15|Diptera|Sphaeroceridae||BOLD:ACW5354
Drosophilidae|[1046]|GMAGE647-15|Diptera|Drosophilidae||BOLD:ACN6865
Drosophilidae|[1047]|GMAGJ1043-15|Diptera|Drosophilidae||BOLD:ACN1043
Drosophilidae|[1048]|GMARK091-14|Diptera|Drosophilidae||BOLD:ACN1178
Tachinidae|[1049]|GMARS291-14|Diptera|Tachinidae||BOLD:ACN8624
Diptera|[1050]|GMAGY189-15|Diptera|||BOLD:ACX2588
Drosophilidae|[1051]|GMAGY126-15|Diptera|Drosophilidae||BOLD:ACT8830
Diptera|[1052]|GMAGE514-15|Diptera|||BOLD:ACW3038
Diptera|[1053]|GMAGY130-15|Diptera|||BOLD:ACX3305
Empididae|[1054]|GMAGN724-15|Diptera|Empididae||BOLD:ACX5044
Limoniidae|[1055]|GMARL457-14|Diptera|Limoniidae||BOLD:ACN3182
Limoniidae|[1056]|GMAGU354-15|Diptera|Limoniidae||BOLD:ACX4351
Onirion|[1057]|GMARH139-14|Diptera|Culicidae|Culicinae|BOLD:ACN0508
Muscidae|[1058]|GMART067-14|Diptera|Muscidae||BOLD:ACO1718
Diptera|[1059]|GMAGA318-15|Diptera|||BOLD:ACV1643
Lonchaeidae|[1060]|GMAGS468-15|Diptera|Lonchaeidae||BOLD:ACX5258
Lonchaeidae|[1061]|GMAGY139-15|Diptera|Lonchaeidae||BOLD:ACX1805
Coproica acutangula|[1062]|GMAGE1045-15|Diptera|Sphaeroceridae|Limosininae|BOLD:AAL7751
Leptocera|[1063]|GMARI2138-14|Diptera|Sphaeroceridae|Limosininae|BOLD:AAG7299
Sphaeroceridae|[1064]|GMARA051-14|Diptera|Sphaeroceridae||BOLD:ACM8901
Sphaeroceridae|[1065]|GMAGN2052-15|Diptera|Sphaeroceridae||BOLD:ACX1650
Sphaeroceridae|[1066]|GMARK1668-14|Diptera|Sphaeroceridae||BOLD:ACN1202
Sphaeroceridae|[1067]|GMARH483-14|Diptera|Sphaeroceridae||BOLD:ACN0761
Sphaeroceridae|[1068]|GMARA1185-14|Diptera|Sphaeroceridae||BOLD:ABW2400
Sphaeroceridae|[1069]|GMAGV512-15|Diptera|Sphaeroceridae||BOLD:ACX2762
Asilidae|[1070]|GMARS913-14|Diptera|Asilidae||BOLD:ACO1835
Chloropidae|[1071]|GMARJ446-14|Diptera|Chloropidae||BOLD:ACN0079
Sobarocephala|[1072]|GMARA1463-14|Diptera|Clusiidae|Clusiinae|BOLD:AAJ3828
Clusiidae|[1073]|GMART768-14|Diptera|Clusiidae||BOLD:ACO0040
Clusiidae|[1074]|GMAGN593-15|Diptera|Clusiidae||BOLD:ACX5051
Clusiidae|[1075]|GMARK2094-14|Diptera|Clusiidae||BOLD:ACN2443
Clusiidae|[1076]|GMART1548-14|Diptera|Clusiidae||BOLD:ACO2013
Sphaeroceridae|[1077]|GMAGA625-15|Diptera|Sphaeroceridae||BOLD:ACV2787
Chloropidae|[1078]|GMAGP596-15|Diptera|Chloropidae||BOLD:ACX5165
Chloropidae|[1079]|GMAGO1168-15|Diptera|Chloropidae||BOLD:ACX4252
Chloropidae|[1080]|GMAGP915-15|Diptera|Chloropidae||BOLD:ACX5259
Chloropidae|[1081]|GMAGY105-15|Diptera|Chloropidae||BOLD:ACX1475
Sphaeroceridae|[1082]|GMAGC741-15|Diptera|Sphaeroceridae||BOLD:ACW1517
Sphaeroceridae|[1083]|GMARD184-14|Diptera|Sphaeroceridae||BOLD:ACN0250
Sphaeroceridae|[1084]|GMAGN1058-15|Diptera|Sphaeroceridae||BOLD:ACX2683
Sphaeroceridae|[1085]|GMARB1393-14|Diptera|Sphaeroceridae||BOLD:ACM5050
Sphaeroceridae|[1086]|GMAGF448-15|Diptera|Sphaeroceridae||BOLD:ACM9904
Sphaeroceridae|[1087]|GMARC782-14|Diptera|Sphaeroceridae||BOLD:ACM5159
Sphaeroceridae|[1088]|GMAGP1328-15|Diptera|Sphaeroceridae||BOLD:AAG7301
Sphaeroceridae|[1089]|GMAGT337-15|Diptera|Sphaeroceridae||BOLD:ACX5708
Sphaeroceridae|[1090]|GMAGB543-15|Diptera|Sphaeroceridae||BOLD:ACW4838
Sphaeroceridae|[1091]|GMARR776-14|Diptera|Sphaeroceridae||BOLD:ACX2841
Sphaeroceridae|[1092]|GMARA143-14|Diptera|Sphaeroceridae||BOLD:ACM7543
Sphaeroceridae|[1093]|GMARA376-14|Diptera|Sphaeroceridae||BOLD:ACM9622
Sphaeroceridae|[1094]|GMAGH044-15|Diptera|Sphaeroceridae||BOLD:ACX0045
Sphaeroceridae|[1095]|GMAGF440-15|Diptera|Sphaeroceridae||BOLD:ACW3283

Sphaeroceridae|[1093]|GMARA376-14|Diptera|Sphaeroceridae||BOLD:ACM9622
Sphaeroceridae|[1094]|GMAGH044-15|Diptera|Sphaeroceridae||BOLD:ACX0045
Sphaeroceridae|[1095]|GMAGF440-15|Diptera|Sphaeroceridae||BOLD:ACW3283
Sphaeroceridae|[1096]|GMARE709-14|Diptera|Sphaeroceridae||BOLD:ACM7122
Sphaeroceridae|[1097]|GMAGM925-15|Diptera|Sphaeroceridae||BOLD:ACX3703
Ulidiidae|[1098]|GMAGQ2415-15|Diptera|Ulidiidae||BOLD:ACX5982
Lauxaniidae|[1099]|GMAGO1107-15|Diptera|Lauxaniidae||BOLD:ACX4134
Lauxaniidae|[1100]|GMAGO1136-15|Diptera|Lauxaniidae||BOLD:ACN2450
Lauxaniidae|[1101]|GMARL533-14|Diptera|Lauxaniidae||BOLD:ACN2742
Lauxaniidae|[1102]|GMAGH229-15|Diptera|Lauxaniidae||BOLD:ACX6492
Lauxaniidae|[1103]|GMARK2076-14|Diptera|Lauxaniidae||BOLD:ACN2844
Lauxaniidae|[1104]|GMAGQ2393-15|Diptera|Lauxaniidae||BOLD:ACX4902
Lauxaniidae|[1105]|GMARL1315-14|Diptera|Lauxaniidae||BOLD:ACN2056
Richardiidae|[1106]|GMARM848-14|Diptera|Richardiidae||BOLD:ACN6896
Lauxaniidae|[1107]|GMAGO1070-15|Diptera|Lauxaniidae||BOLD:ACN3712
Sphaeroceridae|[1108]|GMAGP999-15|Diptera|Sphaeroceridae||BOLD:ACN9901
Diptera|[1109]|GMAGD285-15|Diptera|||BOLD:ACW5793
Diptera|[1110]|GMARN994-14|Diptera|||BOLD:ACX2388
Lauxaniidae|[1111]|GMARA1322-14|Diptera|Lauxaniidae||BOLD:ACM5182
Lauxaniidae|[1112]|GMAGL201-15|Diptera|Lauxaniidae||BOLD:ACX2421
Lauxaniidae|[1113]|GMAGP433-15|Diptera|Lauxaniidae||BOLD:ACX2405
Lauxaniidae|[1114]|GMARN721-14|Diptera|Lauxaniidae||BOLD:ACN7335
Lauxaniidae|[1115]|GMARK1746-14|Diptera|Lauxaniidae||BOLD:ACN0653
Lauxaniidae|[1116]|GMARW050-15|Diptera|Lauxaniidae||BOLD:ACX3754
Lauxaniidae|[1117]|GMAGR867-15|Diptera|Lauxaniidae||BOLD:ACX4702
Diptera|[1118]|GMAGO1314-15|Diptera|||BOLD:ACX6258
Lauxaniidae|[1119]|GMAGC618-15|Diptera|Lauxaniidae||BOLD:ACN0921
Lauxaniidae|[1120]|GMARK1313-14|Diptera|Lauxaniidae||BOLD:ACN2350
Lauxaniidae|[1121]|GMARB724-14|Diptera|Lauxaniidae||BOLD:ACM9028
Lauxaniidae|[1122]|GMARK089-14|Diptera|Lauxaniidae||BOLD:ACX3806
Lauxaniidae|[1123]|GMAGO1283-15|Diptera|Lauxaniidae||BOLD:ACX4784
Lauxaniidae|[1124]|GMARJ382-14|Diptera|Lauxaniidae||BOLD:ACN0641
Lauxaniidae|[1125]|GMAGP410-15|Diptera|Lauxaniidae||BOLD:ACX5492
Lauxaniidae|[1126]|GMAGK175-15|Diptera|Lauxaniidae||BOLD:ACN0688
Lauxaniidae|[1127]|GMAGA339-15|Diptera|Lauxaniidae||BOLD:ACV1224
Lauxaniidae|[1128]|GMAGJ267-15|Diptera|Lauxaniidae||BOLD:ACN1902
Lauxaniidae|[1129]|GMAGM351-15|Diptera|Lauxaniidae||BOLD:ACX3596
Lauxaniidae|[1130]|GMAGO1435-15|Diptera|Lauxaniidae||BOLD:ACX5537
Lauxaniidae|[1131]|GMAGO1147-15|Diptera|Lauxaniidae||BOLD:ACN9470
Lauxaniidae|[1132]|GMAGO1289-15|Diptera|Lauxaniidae||BOLD:ACX2281
Lauxaniidae|[1133]|GMAGP315-15|Diptera|Lauxaniidae||BOLD:ABX8216
Cecidomyiidae|[1134]|GMAGI750-15|Diptera|Cecidomyiidae||BOLD:ACY8257
Lauxaniidae|[1135]|GMAGY190-15|Diptera|Lauxaniidae||BOLD:ACW3139
Lauxaniidae|[1136]|GMAGC627-15|Diptera|Lauxaniidae||BOLD:ACW1372
Lauxaniidae|[1137]|GMAGJ139-15|Diptera|Lauxaniidae||BOLD:ACN3869
Lauxaniidae|[1138]|GMARF576-14|Diptera|Lauxaniidae||BOLD:ACM9020
Lauxaniidae|[1139]|GMARJ1117-14|Diptera|Lauxaniidae||BOLD:ACN0269
Diptera|[1140]|GMARD334-14|Diptera|||BOLD:ACX1557
Diptera|[1141]|GMARL1297-14|Diptera|||BOLD:ACX1190
Diptera|[1142]|GMAGY253-15|Diptera|||BOLD:ACX1713
Drosophilidae|[1143]|GMAGG2181-15|Diptera|Drosophilidae||BOLD:ACN1352
Drosophilidae|[1144]|GMARE431-14|Diptera|Drosophilidae||BOLD:ACN1516
Drosophilidae|[1145]|GMAGE829-15|Diptera|Drosophilidae||BOLD:ACM7801
Drosophilidae|[1146]|GMAGI103-15|Diptera|Drosophilidae||BOLD:ACN1584
Taeniaptera|[1147]|GMARB119-14|Diptera|Micropezidae|Taeniapterinae|BOLD:ACL5274
Drosophilidae|[1148]|GMARD269-14|Diptera|Drosophilidae||BOLD:ACN1691
Micropezidae|[1149]|GMAGA197-15|Diptera|Micropezidae||BOLD:ACN1088
Micropezidae|[1150]|GMARL1773-14|Diptera|Micropezidae||BOLD:ACN6468
Micropezidae|[1151]|GMAGW125-15|Diptera|Micropezidae||BOLD:ACO1916
Sarcophagidae|[1152]|GMART070-14|Diptera|Sarcophagidae||BOLD:ACO1627
Scarabaeidae|[1153]|GMAGX434-15|Coleoptera|Scarabaeidae||BOLD:ACX4913
Sciomyzidae|[1154]|GMAGB762-15|Diptera|Sciomyzidae||BOLD:ACN4743
Sciomyzidae|[1155]|GMAGG445-15|Diptera|Sciomyzidae||BOLD:ACN5598
Sphaeroceridae|[1156]|GMAGA563-15|Diptera|Sphaeroceridae||BOLD:ACM5038
Diptera|[1157]|GMAGE1737-15|Diptera|||BOLD:ACW1914
Ulidiidae|[1158]|GMARK533-14|Diptera|Ulidiidae||BOLD:ACN5034
Ulidiidae|[1159]|GMAGO1355-15|Diptera|Ulidiidae||BOLD:ACX4981
Diptera|[1160]|GMAGO1131-15|Diptera|||BOLD:ACX5075
Orthoptera|[1161]|GMAGM1892-15|Orthoptera|||BOLD:ACO1866
Orthoptera|[1162]|GMARM1307-14|Orthoptera|||BOLD:ACY7533
Orthoptera|[1163]|GMARM1589-14|Orthoptera|||BOLD:ACN6917
Orthoptera|[1164]|GMAGR287-15|Orthoptera|||BOLD:ACX7173
Gryllidae|[1165]|GMAGL1147-15|Orthoptera|Gryllidae||BOLD:ACX6866
Orthoptera|[1166]|GMAGU1064-15|Orthoptera|||BOLD:AAU6102
Tettigoniidae|[1167]|GMAGB001-15|Orthoptera|Tettigoniidae||BOLD:ACN9343
Tettigoniidae|[1168]|GMAGK006-15|Orthoptera|Tettigoniidae||BOLD:ACX6847
Orthoptera|[1169]|GMARL1422-14|Orthoptera|||BOLD:ACN3973
Orthoptera|[1170]|GMARR1380-14|Orthoptera|||BOLD:ACN8591
Orthoptera|[1171]|GMARS616-14|Orthoptera|||BOLD:ACO2115
Dolichopodidae|[1172]|GMART1206-14|Diptera|Dolichopodidae||BOLD:ACO0643
Plecoptera|[1173]|GMARP1275-14|Plecoptera|||BOLD:ACO0081
Dinotoperlinae|[1174]|GMAGV1008-15|Plecoptera|Gripopterygidae|Dinotoperlinae|BOLD:ACX6807
Gripopterygidae|[1175]|GMARF1353-14|Plecoptera|Gripopterygidae||BOLD:ACM9854
Plecoptera|[1176]|GMARR939-14|Plecoptera|||BOLD:ACN9920
Plecoptera|[1177]|GMAGK004-15|Plecoptera|||BOLD:ACN1319
Plecoptera|[1178]|GMARQ035-14|Plecoptera|||BOLD:ACN9629
Plecoptera|[1179]|GMAGS235-15|Plecoptera|||BOLD:ACN9919
Plecoptera|[1180]|GMARS615-14|Plecoptera|||BOLD:ACO1657
Limoniidae|[1181]|GMAGH592-15|Diptera|Limoniidae||BOLD:ACN7246
Limoniidae|[1182]|GMAGC759-15|Diptera|Limoniidae||BOLD:ACM9163
Limoniidae|[1183]|GMAGD169-15|Diptera|Limoniidae||BOLD:ACN0637
Limoniidae|[1184]|GMARO803-14|Diptera|Limoniidae||BOLD:ACN9703
Geranomyia|[1185]|GMARH923-14|Diptera|Limoniidae|Limoniinae|BOLD:ABW0556
Limoniidae|[1186]|GMARG1096-14|Diptera|Limoniidae||BOLD:ACN0347
Limoniidae|[1187]|GMARG1397-14|Diptera|Limoniidae||BOLD:ACN0667
Limoniidae|[1188]|GMAGG1162-15|Diptera|Limoniidae||BOLD:ABV1777
Limoniidae|[1189]|GMAGK239-15|Diptera|Limoniidae||BOLD:ACN1729
Limoniidae|[1190]|GMARQ291-14|Diptera|Limoniidae||BOLD:ACN8700
Limoniidae|[1191]|GMAGG998-15|Diptera|Limoniidae||BOLD:ACM8306
Limoniidae|[1192]|GMARG1061-14|Diptera|Limoniidae||BOLD:ACN0573
Limoniidae|[1193]|GMARA1464-14|Diptera|Limoniidae||BOLD:ACM4900
Limoniidae|[1194]|GMAGF106-15|Diptera|Limoniidae||BOLD:ADD3212
Limoniidae|[1195]|GMARA198-14|Diptera|Limoniidae||BOLD:ACM9360

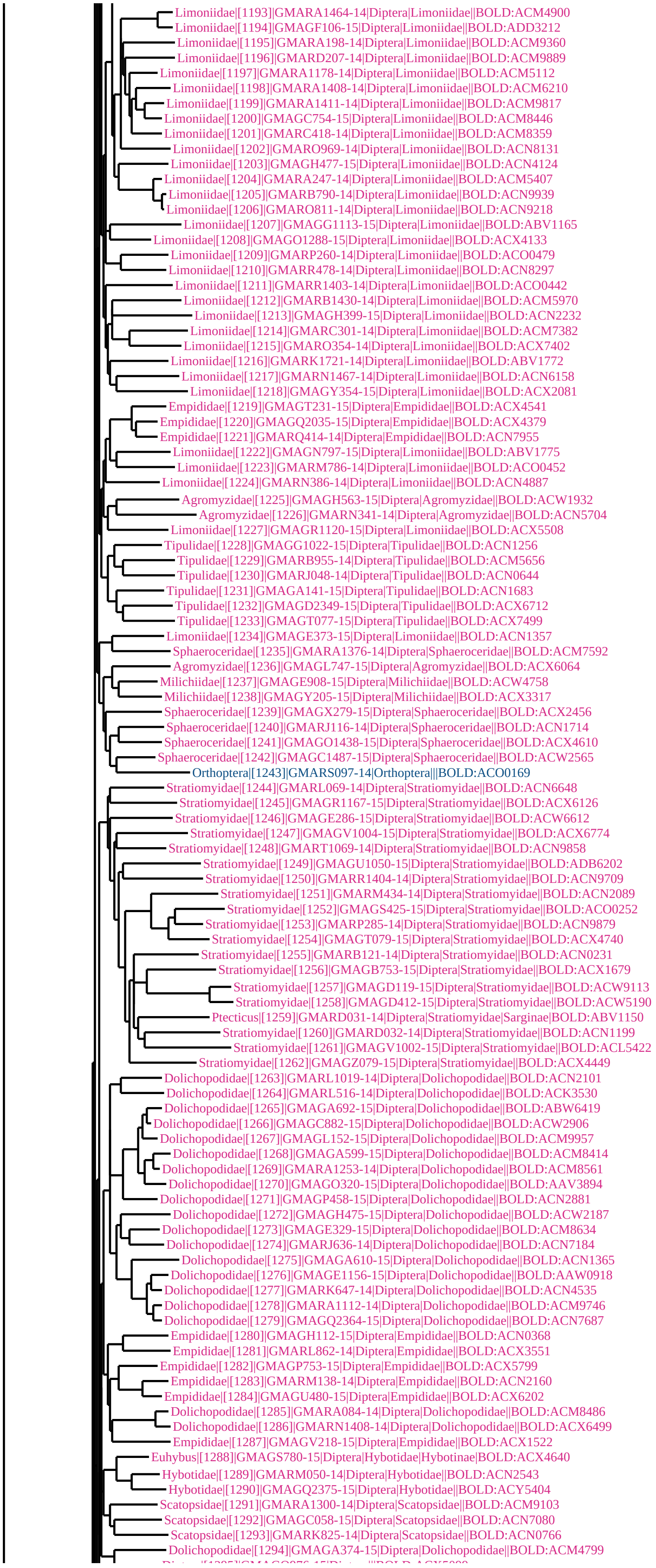
Limoniidae|[1193]|GMARA1464-14|Diptera|Limoniidae||BOLD:ACM4900
Limoniidae|[1194]|GMAGF106-15|Diptera|Limoniidae||BOLD:ADD3212
Limoniidae|[1195]|GMARA198-14|Diptera|Limoniidae||BOLD:ACM9360
Limoniidae|[1196]|GMARD207-14|Diptera|Limoniidae||BOLD:ACM9889
Limoniidae|[1197]|GMARA1178-14|Diptera|Limoniidae||BOLD:ACM5112
Limoniidae|[1198]|GMARA1408-14|Diptera|Limoniidae||BOLD:ACM6210
Limoniidae|[1199]|GMARA1411-14|Diptera|Limoniidae||BOLD:ACM9817
Limoniidae|[1200]|GMAGC754-15|Diptera|Limoniidae||BOLD:ACM8446
Limoniidae|[1201]|GMARC418-14|Diptera|Limoniidae||BOLD:ACM8359
Limoniidae|[1202]|GMARO969-14|Diptera|Limoniidae||BOLD:ACN8131
Limoniidae|[1203]|GMAGH477-15|Diptera|Limoniidae||BOLD:ACN4124
Limoniidae|[1204]|GMARA247-14|Diptera|Limoniidae||BOLD:ACM5407
Limoniidae|[1205]|GMARB790-14|Diptera|Limoniidae||BOLD:ACN9939
Limoniidae|[1206]|GMARO811-14|Diptera|Limoniidae||BOLD:ACN9218
Limoniidae|[1207]|GMAGG1113-15|Diptera|Limoniidae||BOLD:ABV1165
Limoniidae|[1208]|GMAGO1288-15|Diptera|Limoniidae||BOLD:ACX4133
Limoniidae|[1209]|GMARP260-14|Diptera|Limoniidae||BOLD:ACO0479
Limoniidae|[1210]|GMARR478-14|Diptera|Limoniidae||BOLD:ACN8297
Limoniidae|[1211]|GMARR1403-14|Diptera|Limoniidae||BOLD:ACO0442
Limoniidae|[1212]|GMARB1430-14|Diptera|Limoniidae||BOLD:ACM5970
Limoniidae|[1213]|GMAGH399-15|Diptera|Limoniidae||BOLD:ACN2232
Limoniidae|[1214]|GMARC301-14|Diptera|Limoniidae||BOLD:ACM7382
Limoniidae|[1215]|GMARO354-14|Diptera|Limoniidae||BOLD:ACX7402
Limoniidae|[1216]|GMARK1721-14|Diptera|Limoniidae||BOLD:ABV1772
Limoniidae|[1217]|GMARN1467-14|Diptera|Limoniidae||BOLD:ACN6158
Limoniidae|[1218]|GMAGY354-15|Diptera|Limoniidae||BOLD:ACX2081
Empididae|[1219]|GMAGT231-15|Diptera|Empididae||BOLD:ACX4541
Empididae|[1220]|GMAGQ2035-15|Diptera|Empididae||BOLD:ACX4379
Empididae|[1221]|GMARQ414-14|Diptera|Empididae||BOLD:ACN7955
Limoniidae|[1222]|GMAGN797-15|Diptera|Limoniidae||BOLD:ABV1775
Limoniidae|[1223]|GMARM786-14|Diptera|Limoniidae||BOLD:ACO0452
Limoniidae|[1224]|GMARN386-14|Diptera|Limoniidae||BOLD:ACN4887
Agromyzidae|[1225]|GMAGH563-15|Diptera|Agromyzidae||BOLD:ACW1932
Agromyzidae|[1226]|GMARN341-14|Diptera|Agromyzidae||BOLD:ACN5704
Limoniidae|[1227]|GMAGR1120-15|Diptera|Limoniidae||BOLD:ACX5508
Tipulidae|[1228]|GMAGG1022-15|Diptera|Tipulidae||BOLD:ACN1256
Tipulidae|[1229]|GMARB955-14|Diptera|Tipulidae||BOLD:ACM5656
Tipulidae|[1230]|GMARJ048-14|Diptera|Tipulidae||BOLD:ACN0644
Tipulidae|[1231]|GMAGA141-15|Diptera|Tipulidae||BOLD:ACN1683
Tipulidae|[1232]|GMAGD2349-15|Diptera|Tipulidae||BOLD:ACX6712
Tipulidae|[1233]|GMAGT077-15|Diptera|Tipulidae||BOLD:ACX7499
Limoniidae|[1234]|GMAGE373-15|Diptera|Limoniidae||BOLD:ACN1357
Sphaeroceridae|[1235]|GMARA1376-14|Diptera|Sphaeroceridae||BOLD:ACM7592
Agromyzidae|[1236]|GMAGL747-15|Diptera|Agromyzidae||BOLD:ACX6064
Milichiidae|[1237]|GMAGE908-15|Diptera|Milichiidae||BOLD:ACW4758
Milichiidae|[1238]|GMAGY205-15|Diptera|Milichiidae||BOLD:ACX3317
Sphaeroceridae|[1239]|GMAGX279-15|Diptera|Sphaeroceridae||BOLD:ACX2456
Sphaeroceridae|[1240]|GMARJ116-14|Diptera|Sphaeroceridae||BOLD:ACN1714
Sphaeroceridae|[1241]|GMAGO1438-15|Diptera|Sphaeroceridae||BOLD:ACX4610
Sphaeroceridae|[1242]|GMAGC1487-15|Diptera|Sphaeroceridae||BOLD:ACW2565
Orthoptera|[1243]|GMARS097-14|Orthoptera|||BOLD:ACO0169
Stratiomyidae|[1244]|GMARL069-14|Diptera|Stratiomyidae||BOLD:ACN6648
Stratiomyidae|[1245]|GMAGR1167-15|Diptera|Stratiomyidae||BOLD:ACX6126
Stratiomyidae|[1246]|GMAGE286-15|Diptera|Stratiomyidae||BOLD:ACW6612
Stratiomyidae|[1247]|GMAGV1004-15|Diptera|Stratiomyidae||BOLD:ACX6774
Stratiomyidae|[1248]|GMART1069-14|Diptera|Stratiomyidae||BOLD:ACN9858
Stratiomyidae|[1249]|GMAGU1050-15|Diptera|Stratiomyidae||BOLD:ADB6202
Stratiomyidae|[1250]|GMARR1404-14|Diptera|Stratiomyidae||BOLD:ACN9709
Stratiomyidae|[1251]|GMARM434-14|Diptera|Stratiomyidae||BOLD:ACN2089
Stratiomyidae|[1252]|GMAGS425-15|Diptera|Stratiomyidae||BOLD:ACO0252
Stratiomyidae|[1253]|GMARP285-14|Diptera|Stratiomyidae||BOLD:ACN9879
Stratiomyidae|[1254]|GMAGT079-15|Diptera|Stratiomyidae||BOLD:ACX4740
Stratiomyidae|[1255]|GMARB121-14|Diptera|Stratiomyidae||BOLD:ACN0231
Stratiomyidae|[1256]|GMAGB753-15|Diptera|Stratiomyidae||BOLD:ACX1679
Stratiomyidae|[1257]|GMAGD119-15|Diptera|Stratiomyidae||BOLD:ACW9113
Stratiomyidae|[1258]|GMAGD412-15|Diptera|Stratiomyidae||BOLD:ACW5190
Ptecticus|[1259]|GMARD031-14|Diptera|Stratiomyidae|Sarginae|BOLD:ABV1150
Stratiomyidae|[1260]|GMARD032-14|Diptera|Stratiomyidae||BOLD:ACN1199
Stratiomyidae|[1261]|GMAGV1002-15|Diptera|Stratiomyidae||BOLD:ACL5422
Stratiomyidae|[1262]|GMAGZ079-15|Diptera|Stratiomyidae||BOLD:ACX4449
Dolichopodidae|[1263]|GMARL1019-14|Diptera|Dolichopodidae||BOLD:ACN2101
Dolichopodidae|[1264]|GMARL516-14|Diptera|Dolichopodidae||BOLD:ACK3530
Dolichopodidae|[1265]|GMAGA692-15|Diptera|Dolichopodidae||BOLD:ABW6419
Dolichopodidae|[1266]|GMAGC882-15|Diptera|Dolichopodidae||BOLD:ACW2906
Dolichopodidae|[1267]|GMAGL152-15|Diptera|Dolichopodidae||BOLD:ACM9957
Dolichopodidae|[1268]|GMAGA599-15|Diptera|Dolichopodidae||BOLD:ACM8414
Dolichopodidae|[1269]|GMARA1253-14|Diptera|Dolichopodidae||BOLD:ACM8561
Dolichopodidae|[1270]|GMAGO320-15|Diptera|Dolichopodidae||BOLD:AAV3894
Dolichopodidae|[1271]|GMAGP458-15|Diptera|Dolichopodidae||BOLD:ACN2881
Dolichopodidae|[1272]|GMAGH475-15|Diptera|Dolichopodidae||BOLD:ACW2187
Dolichopodidae|[1273]|GMAGE329-15|Diptera|Dolichopodidae||BOLD:ACM8634
Dolichopodidae|[1274]|GMARJ636-14|Diptera|Dolichopodidae||BOLD:ACN7184
Dolichopodidae|[1275]|GMAGA610-15|Diptera|Dolichopodidae||BOLD:ACN1365
Dolichopodidae|[1276]|GMAGE1156-15|Diptera|Dolichopodidae||BOLD:AAW0918
Dolichopodidae|[1277]|GMARK647-14|Diptera|Dolichopodidae||BOLD:ACN4535
Dolichopodidae|[1278]|GMARA1112-14|Diptera|Dolichopodidae||BOLD:ACM9746
Dolichopodidae|[1279]|GMAGQ2364-15|Diptera|Dolichopodidae||BOLD:ACN7687
Empididae|[1280]|GMAGH112-15|Diptera|Empididae||BOLD:ACN0368
Empididae|[1281]|GMARL862-14|Diptera|Empididae||BOLD:ACX3551
Empididae|[1282]|GMAGP753-15|Diptera|Empididae||BOLD:ACX5799
Empididae|[1283]|GMARM138-14|Diptera|Empididae||BOLD:ACN2160
Empididae|[1284]|GMAGU480-15|Diptera|Empididae||BOLD:ACX6202
Dolichopodidae|[1285]|GMARA084-14|Diptera|Dolichopodidae||BOLD:ACM8486
Dolichopodidae|[1286]|GMARN1408-14|Diptera|Dolichopodidae||BOLD:ACX6499
Empididae|[1287]|GMAGV218-15|Diptera|Empididae||BOLD:ACX1522
Euhybus|[1288]|GMAGS780-15|Diptera|Hybotidae|Hybotinae|BOLD:ACX4640
Hybotidae|[1289]|GMARM050-14|Diptera|Hybotidae||BOLD:ACN2543
Hybotidae|[1290]|GMAGQ2375-15|Diptera|Hybotidae||BOLD:ACY5404
Scatopsidae|[1291]|GMARA1300-14|Diptera|Scatopsidae||BOLD:ACM9103
Scatopsidae|[1292]|GMAGC058-15|Diptera|Scatopsidae||BOLD:ACN7080
Scatopsidae|[1293]|GMARK825-14|Diptera|Scatopsidae||BOLD:ACN0766
Dolichopodidae|[1294]|GMAGA374-15|Diptera|Dolichopodidae||BOLD:ACM4799

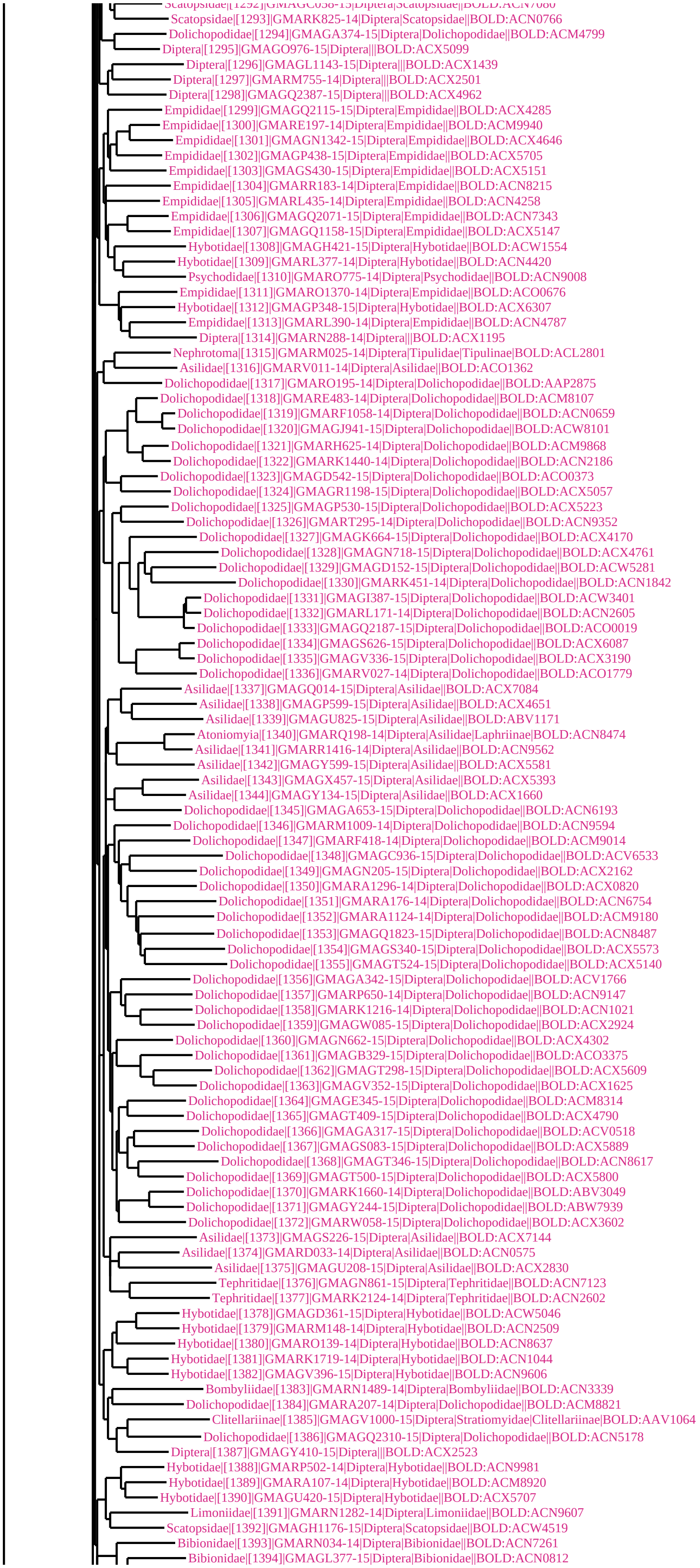
Scatopsidae|[1292]|GMAGC038-15|Diptera|Scatopsidae||BOLD:ACN7080
Scatopsidae|[1293]|GMARK825-14|Diptera|Scatopsidae||BOLD:ACN0766
Dolichopodidae|[1294]|GMAGA374-15|Diptera|Dolichopodidae||BOLD:ACM4799
Diptera|[1295]|GMAGO976-15|Diptera|||BOLD:ACX5099
Diptera|[1296]|GMAGL1143-15|Diptera|||BOLD:ACX1439
Diptera|[1297]|GMARM755-14|Diptera|||BOLD:ACX2501
Diptera|[1298]|GMAGQ2387-15|Diptera|||BOLD:ACX4962
Empididae|[1299]|GMAGQ2115-15|Diptera|Empididae||BOLD:ACX4285
Empididae|[1300]|GMARE197-14|Diptera|Empididae||BOLD:ACM9940
Empididae|[1301]|GMAGN1342-15|Diptera|Empididae||BOLD:ACX4646
Empididae|[1302]|GMAGP438-15|Diptera|Empididae||BOLD:ACX5705
Empididae|[1303]|GMAGS430-15|Diptera|Empididae||BOLD:ACX5151
Empididae|[1304]|GMARR183-14|Diptera|Empididae||BOLD:ACN8215
Empididae|[1305]|GMARL435-14|Diptera|Empididae||BOLD:ACN4258
Empididae|[1306]|GMAGQ2071-15|Diptera|Empididae||BOLD:ACN7343
Empididae|[1307]|GMAGQ1158-15|Diptera|Empididae||BOLD:ACX5147
Hybotidae|[1308]|GMAGH421-15|Diptera|Hybotidae||BOLD:ACW1554
Hybotidae|[1309]|GMARL377-14|Diptera|Hybotidae||BOLD:ACN4420
Psychodidae|[1310]|GMARO775-14|Diptera|Psychodidae||BOLD:ACN9008
Empididae|[1311]|GMARO1370-14|Diptera|Empididae||BOLD:ACO0676
Hybotidae|[1312]|GMAGP348-15|Diptera|Hybotidae||BOLD:ACX6307
Empididae|[1313]|GMARL390-14|Diptera|Empididae||BOLD:ACN4787
Diptera|[1314]|GMARN288-14|Diptera|||BOLD:ACX1195
Nephrotoma|[1315]|GMARM025-14|Diptera|Tipulidae|Tipulinae|BOLD:ACL2801
Asilidae|[1316]|GMARV011-14|Diptera|Asilidae||BOLD:ACO1362
Dolichopodidae|[1317]|GMARO195-14|Diptera|Dolichopodidae||BOLD:AAP2875
Dolichopodidae|[1318]|GMARE483-14|Diptera|Dolichopodidae||BOLD:ACM8107
Dolichopodidae|[1319]|GMARF1058-14|Diptera|Dolichopodidae||BOLD:ACN0659
Dolichopodidae|[1320]|GMAGJ941-15|Diptera|Dolichopodidae||BOLD:ACW8101
Dolichopodidae|[1321]|GMARH625-14|Diptera|Dolichopodidae||BOLD:ACM9868
Dolichopodidae|[1322]|GMARK1440-14|Diptera|Dolichopodidae||BOLD:ACN2186
Dolichopodidae|[1323]|GMAGD542-15|Diptera|Dolichopodidae||BOLD:ACO0373
Dolichopodidae|[1324]|GMAGR1198-15|Diptera|Dolichopodidae||BOLD:ACX5057
Dolichopodidae|[1325]|GMAGP530-15|Diptera|Dolichopodidae||BOLD:ACX5223
Dolichopodidae|[1326]|GMART295-14|Diptera|Dolichopodidae||BOLD:ACN9352
Dolichopodidae|[1327]|GMAGK664-15|Diptera|Dolichopodidae||BOLD:ACX4170
Dolichopodidae|[1328]|GMAGN718-15|Diptera|Dolichopodidae||BOLD:ACX4761
Dolichopodidae|[1329]|GMAGD152-15|Diptera|Dolichopodidae||BOLD:ACW5281
Dolichopodidae|[1330]|GMARK451-14|Diptera|Dolichopodidae||BOLD:ACN1842
Dolichopodidae|[1331]|GMAGI387-15|Diptera|Dolichopodidae||BOLD:ACW3401
Dolichopodidae|[1332]|GMARL171-14|Diptera|Dolichopodidae||BOLD:ACN2605
Dolichopodidae|[1333]|GMAGQ2187-15|Diptera|Dolichopodidae||BOLD:ACO0019
Dolichopodidae|[1334]|GMAGS626-15|Diptera|Dolichopodidae||BOLD:ACX6087
Dolichopodidae|[1335]|GMAGV336-15|Diptera|Dolichopodidae||BOLD:ACX3190
Dolichopodidae|[1336]|GMARV027-14|Diptera|Dolichopodidae||BOLD:ACO1779
Asilidae|[1337]|GMAGQ014-15|Diptera|Asilidae||BOLD:ACX7084
Asilidae|[1338]|GMAGP599-15|Diptera|Asilidae||BOLD:ACX4651
Asilidae|[1339]|GMAGU825-15|Diptera|Asilidae||BOLD:ABV1171
Atoniomyia|[1340]|GMARQ198-14|Diptera|Asilidae|Laphriinae|BOLD:ACN8474
Asilidae|[1341]|GMARR1416-14|Diptera|Asilidae||BOLD:ACN9562
Asilidae|[1342]|GMAGY599-15|Diptera|Asilidae||BOLD:ACX5581
Asilidae|[1343]|GMAGX457-15|Diptera|Asilidae||BOLD:ACX5393
Asilidae|[1344]|GMAGY134-15|Diptera|Asilidae||BOLD:ACX1660
Dolichopodidae|[1345]|GMAGA653-15|Diptera|Dolichopodidae||BOLD:ACN6193
Dolichopodidae|[1346]|GMARM1009-14|Diptera|Dolichopodidae||BOLD:ACN9594
Dolichopodidae|[1347]|GMARF418-14|Diptera|Dolichopodidae||BOLD:ACM9014
Dolichopodidae|[1348]|GMAGC936-15|Diptera|Dolichopodidae||BOLD:ACV6533
Dolichopodidae|[1349]|GMAGN205-15|Diptera|Dolichopodidae||BOLD:ACX2162
Dolichopodidae|[1350]|GMARA1296-14|Diptera|Dolichopodidae||BOLD:ACX0820
Dolichopodidae|[1351]|GMARA176-14|Diptera|Dolichopodidae||BOLD:ACN6754
Dolichopodidae|[1352]|GMARA1124-14|Diptera|Dolichopodidae||BOLD:ACM9180
Dolichopodidae|[1353]|GMAGQ1823-15|Diptera|Dolichopodidae||BOLD:ACN8487
Dolichopodidae|[1354]|GMAGS340-15|Diptera|Dolichopodidae||BOLD:ACX5573
Dolichopodidae|[1355]|GMAGT524-15|Diptera|Dolichopodidae||BOLD:ACX5140
Dolichopodidae|[1356]|GMAGA342-15|Diptera|Dolichopodidae||BOLD:ACV1766
Dolichopodidae|[1357]|GMARP650-14|Diptera|Dolichopodidae||BOLD:ACN9147
Dolichopodidae|[1358]|GMARK1216-14|Diptera|Dolichopodidae||BOLD:ACN1021
Dolichopodidae|[1359]|GMAGW085-15|Diptera|Dolichopodidae||BOLD:ACX2924
Dolichopodidae|[1360]|GMAGN662-15|Diptera|Dolichopodidae||BOLD:ACX4302
Dolichopodidae|[1361]|GMAGB329-15|Diptera|Dolichopodidae||BOLD:ACO3375
Dolichopodidae|[1362]|GMAGT298-15|Diptera|Dolichopodidae||BOLD:ACX5609
Dolichopodidae|[1363]|GMAGV352-15|Diptera|Dolichopodidae||BOLD:ACX1625
Dolichopodidae|[1364]|GMAGE345-15|Diptera|Dolichopodidae||BOLD:ACM8314
Dolichopodidae|[1365]|GMAGT409-15|Diptera|Dolichopodidae||BOLD:ACX4790
Dolichopodidae|[1366]|GMAGA317-15|Diptera|Dolichopodidae||BOLD:ACV0518
Dolichopodidae|[1367]|GMAGS083-15|Diptera|Dolichopodidae||BOLD:ACX5889
Dolichopodidae|[1368]|GMAGT346-15|Diptera|Dolichopodidae||BOLD:ACN8617
Dolichopodidae|[1369]|GMAGT500-15|Diptera|Dolichopodidae||BOLD:ACX5800
Dolichopodidae|[1370]|GMARK1660-14|Diptera|Dolichopodidae||BOLD:ABV3049
Dolichopodidae|[1371]|GMAGY244-15|Diptera|Dolichopodidae||BOLD:ABW7939
Dolichopodidae|[1372]|GMARW058-15|Diptera|Dolichopodidae||BOLD:ACX3602
Asilidae|[1373]|GMAGS226-15|Diptera|Asilidae||BOLD:ACX7144
Asilidae|[1374]|GMARD033-14|Diptera|Asilidae||BOLD:ACN0575
Asilidae|[1375]|GMAGU208-15|Diptera|Asilidae||BOLD:ACX2830
Tephritidae|[1376]|GMAGN861-15|Diptera|Tephritidae||BOLD:ACN7123
Tephritidae|[1377]|GMARK2124-14|Diptera|Tephritidae||BOLD:ACN2602
Hybotidae|[1378]|GMAGD361-15|Diptera|Hybotidae||BOLD:ACW5046
Hybotidae|[1379]|GMARM148-14|Diptera|Hybotidae||BOLD:ACN2509
Hybotidae|[1380]|GMARO139-14|Diptera|Hybotidae||BOLD:ACN8637
Hybotidae|[1381]|GMARK1719-14|Diptera|Hybotidae||BOLD:ACN1044
Hybotidae|[1382]|GMAGV396-15|Diptera|Hybotidae||BOLD:ACN9606
Bombyliidae|[1383]|GMARN1489-14|Diptera|Bombyliidae||BOLD:ACN3339
Dolichopodidae|[1384]|GMARA207-14|Diptera|Dolichopodidae||BOLD:ACM8821
Clitellariinae|[1385]|GMAGV1000-15|Diptera|Stratiomyidae|Clitellariinae|BOLD:AAV1064
Dolichopodidae|[1386]|GMAGQ2310-15|Diptera|Dolichopodidae||BOLD:ACN5178
Diptera|[1387]|GMAGY410-15|Diptera|||BOLD:ACX2523
Hybotidae|[1388]|GMARP502-14|Diptera|Hybotidae||BOLD:ACN9981
Hybotidae|[1389]|GMARA107-14|Diptera|Hybotidae||BOLD:ACM8920
Hybotidae|[1390]|GMAGU420-15|Diptera|Hybotidae||BOLD:ACX5707
Limoniidae|[1391]|GMARN1282-14|Diptera|Limoniidae||BOLD:ACN9607
Scatopsidae|[1392]|GMAGH1176-15|Diptera|Scatopsidae||BOLD:ACW4519
Bibionidae|[1393]|GMARN034-14|Diptera|Bibionidae||BOLD:ACN7261
Bibionidae|[1394]|GMAGL377-15|Diptera|Bibionidae||BOLD:ACN0812

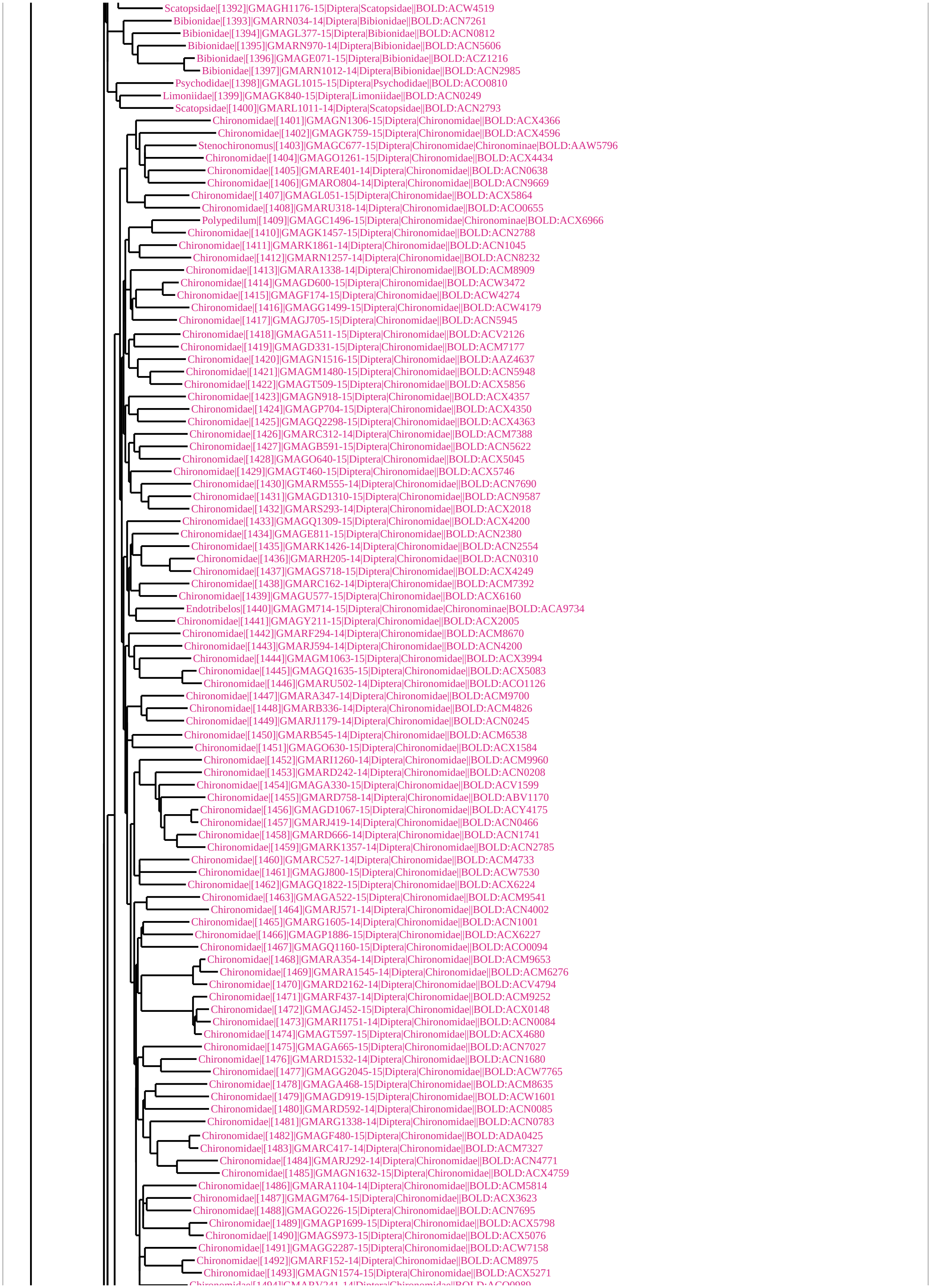
Scatopsidae|[1392]|GMAGH1176-15|Diptera|Scatopsidae||BOLD:ACW4519
Bibionidae|[1393]|GMARN034-14|Diptera|Bibionidae||BOLD:ACN7261
Bibionidae|[1394]|GMAGL377-15|Diptera|Bibionidae||BOLD:ACN0812
Bibionidae|[1395]|GMARN970-14|Diptera|Bibionidae||BOLD:ACN5606
Bibionidae|[1396]|GMAGE071-15|Diptera|Bibionidae||BOLD:ACZ1216
Bibionidae|[1397]|GMARN1012-14|Diptera|Bibionidae||BOLD:ACN2985
Psychodidae|[1398]|GMAGL1015-15|Diptera|Psychodidae||BOLD:ACO0810
Limoniidae|[1399]|GMAGK840-15|Diptera|Limoniidae||BOLD:ACN0249
Scatopsidae|[1400]|GMARL1011-14|Diptera|Scatopsidae||BOLD:ACN2793
Chironomidae|[1401]|GMAGN1306-15|Diptera|Chironomidae||BOLD:ACX4366
Chironomidae|[1402]|GMAGK759-15|Diptera|Chironomidae||BOLD:ACX4596
Stenochironomus|[1403]|GMAGC677-15|Diptera|Chironomidae|Chironominae|BOLD:AAW5796
Chironomidae|[1404]|GMAGO1261-15|Diptera|Chironomidae||BOLD:ACX4434
Chironomidae|[1405]|GMARE401-14|Diptera|Chironomidae||BOLD:ACN0638
Chironomidae|[1406]|GMARO804-14|Diptera|Chironomidae||BOLD:ACN9669
Chironomidae|[1407]|GMAGL051-15|Diptera|Chironomidae||BOLD:ACX5864
Chironomidae|[1408]|GMARU318-14|Diptera|Chironomidae||BOLD:ACO0655
Polypedilum|[1409]|GMAGC1496-15|Diptera|Chironomidae|Chironominae|BOLD:ACX6966
Chironomidae|[1410]|GMAGK1457-15|Diptera|Chironomidae||BOLD:ACN2788
Chironomidae|[1411]|GMARK1861-14|Diptera|Chironomidae||BOLD:ACN1045
Chironomidae|[1412]|GMARN1257-14|Diptera|Chironomidae||BOLD:ACN8232
Chironomidae|[1413]|GMARA1338-14|Diptera|Chironomidae||BOLD:ACM8909
Chironomidae|[1414]|GMAGD600-15|Diptera|Chironomidae||BOLD:ACW3472
Chironomidae|[1415]|GMAGF174-15|Diptera|Chironomidae||BOLD:ACW4274
Chironomidae|[1416]|GMAGG1499-15|Diptera|Chironomidae||BOLD:ACW4179
Chironomidae|[1417]|GMAGJ705-15|Diptera|Chironomidae||BOLD:ACN5945
Chironomidae|[1418]|GMAGA511-15|Diptera|Chironomidae||BOLD:ACV2126
Chironomidae|[1419]|GMAGD331-15|Diptera|Chironomidae||BOLD:ACM7177
Chironomidae|[1420]|GMAGN1516-15|Diptera|Chironomidae||BOLD:AAZ4637
Chironomidae|[1421]|GMAGM1480-15|Diptera|Chironomidae||BOLD:ACN5948
Chironomidae|[1422]|GMAGT509-15|Diptera|Chironomidae||BOLD:ACX5856
Chironomidae|[1423]|GMAGN918-15|Diptera|Chironomidae||BOLD:ACX4357
Chironomidae|[1424]|GMAGP704-15|Diptera|Chironomidae||BOLD:ACX4350
Chironomidae|[1425]|GMAGQ2298-15|Diptera|Chironomidae||BOLD:ACX4363
Chironomidae|[1426]|GMARC312-14|Diptera|Chironomidae||BOLD:ACM7388
Chironomidae|[1427]|GMAGB591-15|Diptera|Chironomidae||BOLD:ACN5622
Chironomidae|[1428]|GMAGO640-15|Diptera|Chironomidae||BOLD:ACX5045
Chironomidae|[1429]|GMAGT460-15|Diptera|Chironomidae||BOLD:ACX5746
Chironomidae|[1430]|GMARM555-14|Diptera|Chironomidae||BOLD:ACN7690
Chironomidae|[1431]|GMAGD1310-15|Diptera|Chironomidae||BOLD:ACN9587
Chironomidae|[1432]|GMARS293-14|Diptera|Chironomidae||BOLD:ACX2018
Chironomidae|[1433]|GMAGQ1309-15|Diptera|Chironomidae||BOLD:ACX4200
Chironomidae|[1434]|GMAGE811-15|Diptera|Chironomidae||BOLD:ACN2380
Chironomidae|[1435]|GMARK1426-14|Diptera|Chironomidae||BOLD:ACN2554
Chironomidae|[1436]|GMARH205-14|Diptera|Chironomidae||BOLD:ACN0310
Chironomidae|[1437]|GMAGS718-15|Diptera|Chironomidae||BOLD:ACX4249
Chironomidae|[1438]|GMARC162-14|Diptera|Chironomidae||BOLD:ACM7392
Chironomidae|[1439]|GMAGU577-15|Diptera|Chironomidae||BOLD:ACX6160
Endotribelos|[1440]|GMAGM714-15|Diptera|Chironomidae|Chironominae|BOLD:ACA9734
Chironomidae|[1441]|GMAGY211-15|Diptera|Chironomidae||BOLD:ACX2005
Chironomidae|[1442]|GMARF294-14|Diptera|Chironomidae||BOLD:ACM8670
Chironomidae|[1443]|GMARJ594-14|Diptera|Chironomidae||BOLD:ACN4200
Chironomidae|[1444]|GMAGM1063-15|Diptera|Chironomidae||BOLD:ACX3994
Chironomidae|[1445]|GMAGQ1635-15|Diptera|Chironomidae||BOLD:ACX5083
Chironomidae|[1446]|GMARU502-14|Diptera|Chironomidae||BOLD:ACO1126
Chironomidae|[1447]|GMARA347-14|Diptera|Chironomidae||BOLD:ACM9700
Chironomidae|[1448]|GMARB336-14|Diptera|Chironomidae||BOLD:ACM4826
Chironomidae|[1449]|GMARJ1179-14|Diptera|Chironomidae||BOLD:ACN0245
Chironomidae|[1450]|GMARB545-14|Diptera|Chironomidae||BOLD:ACM6538
Chironomidae|[1451]|GMAGO630-15|Diptera|Chironomidae||BOLD:ACX1584
Chironomidae|[1452]|GMARI1260-14|Diptera|Chironomidae||BOLD:ACM9960
Chironomidae|[1453]|GMARD242-14|Diptera|Chironomidae||BOLD:ACN0208
Chironomidae|[1454]|GMAGA330-15|Diptera|Chironomidae||BOLD:ACV1599
Chironomidae|[1455]|GMARD758-14|Diptera|Chironomidae||BOLD:ABV1170
Chironomidae|[1456]|GMAGD1067-15|Diptera|Chironomidae||BOLD:ACY4175
Chironomidae|[1457]|GMARJ419-14|Diptera|Chironomidae||BOLD:ACN0466
Chironomidae|[1458]|GMARD666-14|Diptera|Chironomidae||BOLD:ACN1741
Chironomidae|[1459]|GMARK1357-14|Diptera|Chironomidae||BOLD:ACN2785
Chironomidae|[1460]|GMARC527-14|Diptera|Chironomidae||BOLD:ACM4733
Chironomidae|[1461]|GMAGJ800-15|Diptera|Chironomidae||BOLD:ACW7530
Chironomidae|[1462]|GMAGQ1822-15|Diptera|Chironomidae||BOLD:ACX6224
Chironomidae|[1463]|GMAGA522-15|Diptera|Chironomidae||BOLD:ACM9541
Chironomidae|[1464]|GMARJ571-14|Diptera|Chironomidae||BOLD:ACN4002
Chironomidae|[1465]|GMARG1605-14|Diptera|Chironomidae||BOLD:ACN1001
Chironomidae|[1466]|GMAGP1886-15|Diptera|Chironomidae||BOLD:ACX6227
Chironomidae|[1467]|GMAGQ1160-15|Diptera|Chironomidae||BOLD:ACO0094
Chironomidae|[1468]|GMARA354-14|Diptera|Chironomidae||BOLD:ACM9653
Chironomidae|[1469]|GMARA1545-14|Diptera|Chironomidae||BOLD:ACM6276
Chironomidae|[1470]|GMARD2162-14|Diptera|Chironomidae||BOLD:ACV4794
Chironomidae|[1471]|GMARF437-14|Diptera|Chironomidae||BOLD:ACM9252
Chironomidae|[1472]|GMAGJ452-15|Diptera|Chironomidae||BOLD:ACX0148
Chironomidae|[1473]|GMARI1751-14|Diptera|Chironomidae||BOLD:ACN0084
Chironomidae|[1474]|GMAGT597-15|Diptera|Chironomidae||BOLD:ACX4680
Chironomidae|[1475]|GMAGA665-15|Diptera|Chironomidae||BOLD:ACN7027
Chironomidae|[1476]|GMARD1532-14|Diptera|Chironomidae||BOLD:ACN1680
Chironomidae|[1477]|GMAGG2045-15|Diptera|Chironomidae||BOLD:ACW7765
Chironomidae|[1478]|GMAGA468-15|Diptera|Chironomidae||BOLD:ACM8635
Chironomidae|[1479]|GMAGD919-15|Diptera|Chironomidae||BOLD:ACW1601
Chironomidae|[1480]|GMARD592-14|Diptera|Chironomidae||BOLD:ACN0085
Chironomidae|[1481]|GMARG1338-14|Diptera|Chironomidae||BOLD:ACN0783
Chironomidae|[1482]|GMAGF480-15|Diptera|Chironomidae||BOLD:ADA0425
Chironomidae|[1483]|GMARC417-14|Diptera|Chironomidae||BOLD:ACM7327
Chironomidae|[1484]|GMARJ292-14|Diptera|Chironomidae||BOLD:ACN4771
Chironomidae|[1485]|GMAGN1632-15|Diptera|Chironomidae||BOLD:ACX4759
Chironomidae|[1486]|GMARA1104-14|Diptera|Chironomidae||BOLD:ACM5814
Chironomidae|[1487]|GMAGM764-15|Diptera|Chironomidae||BOLD:ACX3623
Chironomidae|[1488]|GMAGO226-15|Diptera|Chironomidae||BOLD:ACN7695
Chironomidae|[1489]|GMAGP1699-15|Diptera|Chironomidae||BOLD:ACX5798
Chironomidae|[1490]|GMAGS973-15|Diptera|Chironomidae||BOLD:ACX5076
Chironomidae|[1491]|GMAGG2287-15|Diptera|Chironomidae||BOLD:ACW7158
Chironomidae|[1492]|GMARF152-14|Diptera|Chironomidae||BOLD:ACM8975
Chironomidae|[1493]|GMAGN1574-15|Diptera|Chironomidae||BOLD:ACX5271

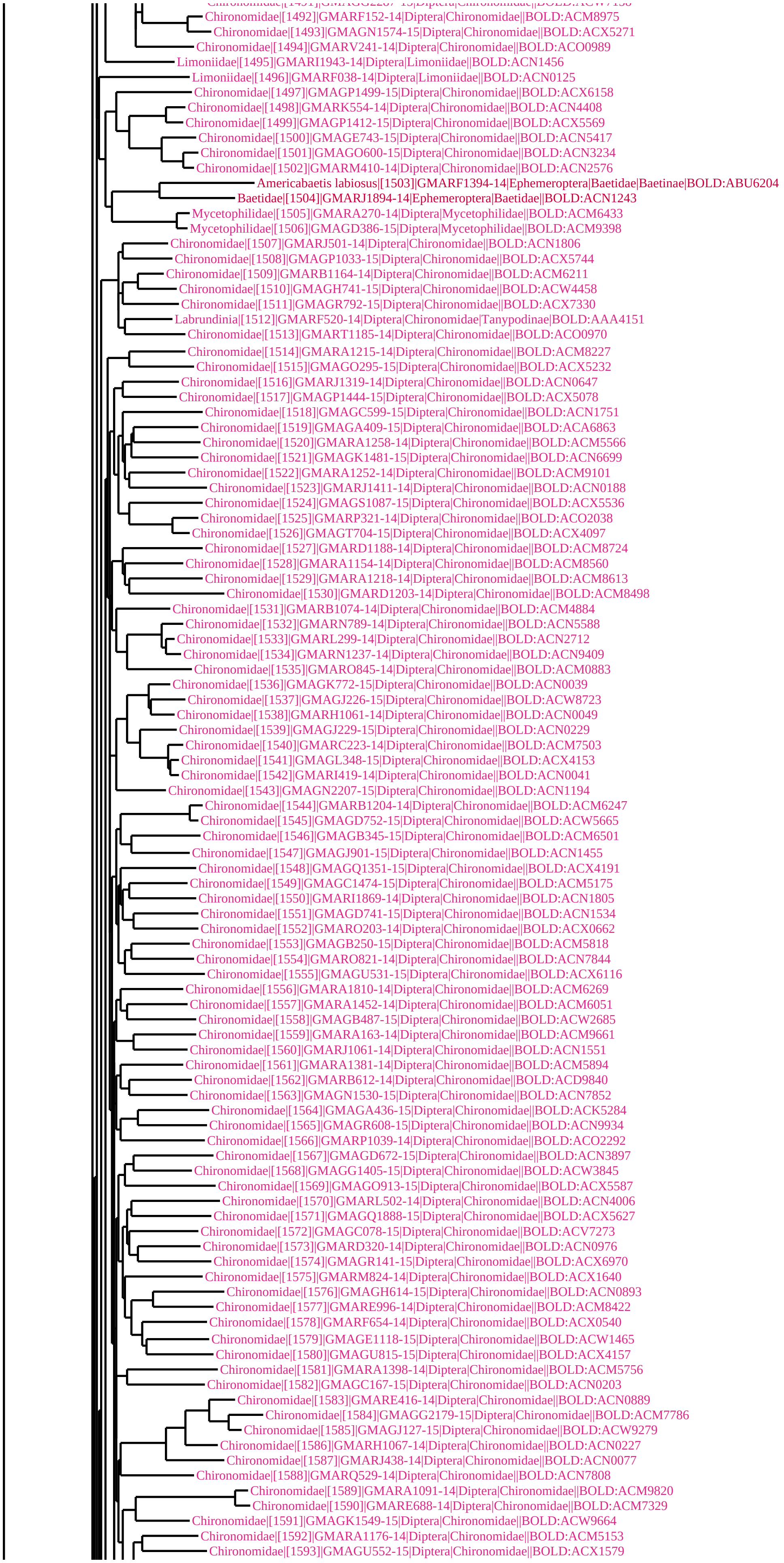
Chironomidae|[1492]|GMARF152-14|Diptera|Chironomidae||BOLD:ACM8975
Chironomidae|[1493]|GMAGN1574-15|Diptera|Chironomidae||BOLD:ACX5271
Chironomidae|[1494]|GMARV241-14|Diptera|Chironomidae||BOLD:ACO0989
Limoniidae|[1495]|GMARI1943-14|Diptera|Limoniidae||BOLD:ACN1456
Limoniidae|[1496]|GMARF038-14|Diptera|Limoniidae||BOLD:ACN0125
Chironomidae|[1497]|GMAGP1499-15|Diptera|Chironomidae||BOLD:ACX6158
Chironomidae|[1498]|GMARK554-14|Diptera|Chironomidae||BOLD:ACN4408
Chironomidae|[1499]|GMAGP1412-15|Diptera|Chironomidae||BOLD:ACX5569
Chironomidae|[1500]|GMAGE743-15|Diptera|Chironomidae||BOLD:ACN5417
Chironomidae|[1501]|GMAGO600-15|Diptera|Chironomidae||BOLD:ACN3234
Chironomidae|[1502]|GMARM410-14|Diptera|Chironomidae||BOLD:ACN2576
Americabaetis labiosus|[1503]|GMARF1394-14|Ephemeroptera|Baetidae|Baetinae|BOLD:ABU6204
Baetidae|[1504]|GMARJ1894-14|Ephemeroptera|Baetidae||BOLD:ACN1243
Mycetophilidae|[1505]|GMARA270-14|Diptera|Mycetophilidae||BOLD:ACM6433
Mycetophilidae|[1506]|GMAGD386-15|Diptera|Mycetophilidae||BOLD:ACM9398
Chironomidae|[1507]|GMARJ501-14|Diptera|Chironomidae||BOLD:ACN1806
Chironomidae|[1508]|GMAGP1033-15|Diptera|Chironomidae||BOLD:ACX5744
Chironomidae|[1509]|GMARB1164-14|Diptera|Chironomidae||BOLD:ACM6211
Chironomidae|[1510]|GMAGH741-15|Diptera|Chironomidae||BOLD:ACW4458
Chironomidae|[1511]|GMAGR792-15|Diptera|Chironomidae||BOLD:ACX7330
Labrundinia|[1512]|GMARF520-14|Diptera|Chironomidae|Tanypodinae|BOLD:AAA4151
Chironomidae|[1513]|GMART1185-14|Diptera|Chironomidae||BOLD:ACO0970
Chironomidae|[1514]|GMARA1215-14|Diptera|Chironomidae||BOLD:ACM8227
Chironomidae|[1515]|GMAGO295-15|Diptera|Chironomidae||BOLD:ACX5232
Chironomidae|[1516]|GMARJ1319-14|Diptera|Chironomidae||BOLD:ACN0647
Chironomidae|[1517]|GMAGP1444-15|Diptera|Chironomidae||BOLD:ACX5078
Chironomidae|[1518]|GMAGC599-15|Diptera|Chironomidae||BOLD:ACN1751
Chironomidae|[1519]|GMAGA409-15|Diptera|Chironomidae||BOLD:ACA6863
Chironomidae|[1520]|GMARA1258-14|Diptera|Chironomidae||BOLD:ACM5566
Chironomidae|[1521]|GMAGK1481-15|Diptera|Chironomidae||BOLD:ACN6699
Chironomidae|[1522]|GMARA1252-14|Diptera|Chironomidae||BOLD:ACM9101
Chironomidae|[1523]|GMARJ1411-14|Diptera|Chironomidae||BOLD:ACN0188
Chironomidae|[1524]|GMAGS1087-15|Diptera|Chironomidae||BOLD:ACX5536
Chironomidae|[1525]|GMARP321-14|Diptera|Chironomidae||BOLD:ACO2038
Chironomidae|[1526]|GMAGT704-15|Diptera|Chironomidae||BOLD:ACX4097
Chironomidae|[1527]|GMARD1188-14|Diptera|Chironomidae||BOLD:ACM8724
Chironomidae|[1528]|GMARA1154-14|Diptera|Chironomidae||BOLD:ACM8560
Chironomidae|[1529]|GMARA1218-14|Diptera|Chironomidae||BOLD:ACM8613
Chironomidae|[1530]|GMARD1203-14|Diptera|Chironomidae||BOLD:ACM8498
Chironomidae|[1531]|GMARB1074-14|Diptera|Chironomidae||BOLD:ACM4884
Chironomidae|[1532]|GMARN789-14|Diptera|Chironomidae||BOLD:ACN5588
Chironomidae|[1533]|GMARL299-14|Diptera|Chironomidae||BOLD:ACN2712
Chironomidae|[1534]|GMARN1237-14|Diptera|Chironomidae||BOLD:ACN9409
Chironomidae|[1535]|GMARO845-14|Diptera|Chironomidae||BOLD:ACM0883
Chironomidae|[1536]|GMAGK772-15|Diptera|Chironomidae||BOLD:ACN0039
Chironomidae|[1537]|GMAGJ226-15|Diptera|Chironomidae||BOLD:ACW8723
Chironomidae|[1538]|GMARH1061-14|Diptera|Chironomidae||BOLD:ACN0049
Chironomidae|[1539]|GMAGJ229-15|Diptera|Chironomidae||BOLD:ACN0229
Chironomidae|[1540]|GMARC223-14|Diptera|Chironomidae||BOLD:ACM7503
Chironomidae|[1541]|GMAGL348-15|Diptera|Chironomidae||BOLD:ACX4153
Chironomidae|[1542]|GMARI419-14|Diptera|Chironomidae||BOLD:ACN0041
Chironomidae|[1543]|GMAGN2207-15|Diptera|Chironomidae||BOLD:ACN1194
Chironomidae|[1544]|GMARB1204-14|Diptera|Chironomidae||BOLD:ACM6247
Chironomidae|[1545]|GMAGD752-15|Diptera|Chironomidae||BOLD:ACW5665
Chironomidae|[1546]|GMAGB345-15|Diptera|Chironomidae||BOLD:ACM6501
Chironomidae|[1547]|GMAGJ901-15|Diptera|Chironomidae||BOLD:ACN1455
Chironomidae|[1548]|GMAGQ1351-15|Diptera|Chironomidae||BOLD:ACX4191
Chironomidae|[1549]|GMAGC1474-15|Diptera|Chironomidae||BOLD:ACM5175
Chironomidae|[1550]|GMARI1869-14|Diptera|Chironomidae||BOLD:ACN1805
Chironomidae|[1551]|GMAGD741-15|Diptera|Chironomidae||BOLD:ACN1534
Chironomidae|[1552]|GMARO203-14|Diptera|Chironomidae||BOLD:ACX0662
Chironomidae|[1553]|GMAGB250-15|Diptera|Chironomidae||BOLD:ACM5818
Chironomidae|[1554]|GMARO821-14|Diptera|Chironomidae||BOLD:ACN7844
Chironomidae|[1555]|GMAGU531-15|Diptera|Chironomidae||BOLD:ACX6116
Chironomidae|[1556]|GMARA1810-14|Diptera|Chironomidae||BOLD:ACM6269
Chironomidae|[1557]|GMARA1452-14|Diptera|Chironomidae||BOLD:ACM6051
Chironomidae|[1558]|GMAGB487-15|Diptera|Chironomidae||BOLD:ACW2685
Chironomidae|[1559]|GMARA163-14|Diptera|Chironomidae||BOLD:ACM9661
Chironomidae|[1560]|GMARJ1061-14|Diptera|Chironomidae||BOLD:ACN1551
Chironomidae|[1561]|GMARA1381-14|Diptera|Chironomidae||BOLD:ACM5894
Chironomidae|[1562]|GMARB612-14|Diptera|Chironomidae||BOLD:ACD9840
Chironomidae|[1563]|GMAGN1530-15|Diptera|Chironomidae||BOLD:ACN7852
Chironomidae|[1564]|GMAGA436-15|Diptera|Chironomidae||BOLD:ACK5284
Chironomidae|[1565]|GMAGR608-15|Diptera|Chironomidae||BOLD:ACN9934
Chironomidae|[1566]|GMARP1039-14|Diptera|Chironomidae||BOLD:ACO2292
Chironomidae|[1567]|GMAGD672-15|Diptera|Chironomidae||BOLD:ACN3897
Chironomidae|[1568]|GMAGG1405-15|Diptera|Chironomidae||BOLD:ACW3845
Chironomidae|[1569]|GMAGO913-15|Diptera|Chironomidae||BOLD:ACX5587
Chironomidae|[1570]|GMARL502-14|Diptera|Chironomidae||BOLD:ACN4006
Chironomidae|[1571]|GMAGQ1888-15|Diptera|Chironomidae||BOLD:ACX5627
Chironomidae|[1572]|GMAGC078-15|Diptera|Chironomidae||BOLD:ACV7273
Chironomidae|[1573]|GMARD320-14|Diptera|Chironomidae||BOLD:ACN0976
Chironomidae|[1574]|GMAGR141-15|Diptera|Chironomidae||BOLD:ACX6970
Chironomidae|[1575]|GMARM824-14|Diptera|Chironomidae||BOLD:ACX1640
Chironomidae|[1576]|GMAGH614-15|Diptera|Chironomidae||BOLD:ACN0893
Chironomidae|[1577]|GMARE996-14|Diptera|Chironomidae||BOLD:ACM8422
Chironomidae|[1578]|GMARF654-14|Diptera|Chironomidae||BOLD:ACX0540
Chironomidae|[1579]|GMAGE1118-15|Diptera|Chironomidae||BOLD:ACW1465
Chironomidae|[1580]|GMAGU815-15|Diptera|Chironomidae||BOLD:ACX4157
Chironomidae|[1581]|GMARA1398-14|Diptera|Chironomidae||BOLD:ACM5756
Chironomidae|[1582]|GMAGC167-15|Diptera|Chironomidae||BOLD:ACN0203
Chironomidae|[1583]|GMARE416-14|Diptera|Chironomidae||BOLD:ACN0889
Chironomidae|[1584]|GMAGG2179-15|Diptera|Chironomidae||BOLD:ACM7786
Chironomidae|[1585]|GMAGJ127-15|Diptera|Chironomidae||BOLD:ACW9279
Chironomidae|[1586]|GMARH1067-14|Diptera|Chironomidae||BOLD:ACN0227
Chironomidae|[1587]|GMARJ438-14|Diptera|Chironomidae||BOLD:ACN0077
Chironomidae|[1588]|GMARQ529-14|Diptera|Chironomidae||BOLD:ACN7808
Chironomidae|[1589]|GMARA1091-14|Diptera|Chironomidae||BOLD:ACM9820
Chironomidae|[1590]|GMARE688-14|Diptera|Chironomidae||BOLD:ACM7329
Chironomidae|[1591]|GMAGK1549-15|Diptera|Chironomidae||BOLD:ACW9664
Chironomidae|[1592]|GMARA1176-14|Diptera|Chironomidae||BOLD:ACM5153
Chironomidae|[1593]|GMAGU552-15|Diptera|Chironomidae||BOLD:ACX1579

Chironomidae|[1591]|GMAGK1549-15|Diptera|Chironomidae||BOLD:ACW9664
Chironomidae|[1592]|GMARA1176-14|Diptera|Chironomidae||BOLD:ACM5153
Chironomidae|[1593]|GMAGU552-15|Diptera|Chironomidae||BOLD:ACX1579
Chironomidae|[1594]|GMAGE1689-15|Diptera|Chironomidae||BOLD:ACW2656
Chironomidae|[1595]|GMARB1563-14|Diptera|Chironomidae||BOLD:ACM4911
Chironomidae|[1596]|GMAGE2125-15|Diptera|Chironomidae||BOLD:ACN8461
Chironomidae|[1597]|GMAGH725-15|Diptera|Chironomidae||BOLD:ACN9648
Chironomidae|[1598]|GMARB1304-14|Diptera|Chironomidae||BOLD:ACM4974
Chironomidae|[1599]|GMAGR619-15|Diptera|Chironomidae||BOLD:ACN3568
Chironomidae|[1600]|GMAGT714-15|Diptera|Chironomidae||BOLD:ACX5041
Chironomidae|[1601]|GMARA1347-14|Diptera|Chironomidae||BOLD:ACM5237
Chironomidae|[1602]|GMART1139-14|Diptera|Chironomidae||BOLD:ACN9850
Chironomidae|[1603]|GMAGE1017-15|Diptera|Chironomidae||BOLD:ACM8853
Chironomidae|[1604]|GMARM502-14|Diptera|Chironomidae||BOLD:ACN2645
Chironomidae|[1605]|GMARB1311-14|Diptera|Chironomidae||BOLD:ACW3704
Chironomidae|[1606]|GMAGA535-15|Diptera|Chironomidae||BOLD:ACM4819
Chironomidae|[1607]|GMAGN2090-15|Diptera|Chironomidae||BOLD:ACO1136
Chironomidae|[1608]|GMARL651-14|Diptera|Chironomidae||BOLD:ACN7213
Chironomidae|[1609]|GMAGD909-15|Diptera|Chironomidae||BOLD:ACW3394
Chironomidae|[1610]|GMARO375-14|Diptera|Chironomidae||BOLD:ACN6103
Chironomidae|[1611]|GMARD1516-14|Diptera|Chironomidae||BOLD:ACN2091
Chironomidae|[1612]|GMAGB643-15|Diptera|Chironomidae||BOLD:ACO0215
Chironomidae|[1613]|GMARC722-14|Diptera|Chironomidae||BOLD:ACM4840
Chironomidae|[1614]|GMARA028-14|Diptera|Chironomidae||BOLD:ACM5474
Chironomidae|[1615]|GMARN585-14|Diptera|Chironomidae||BOLD:ACX6888
Chironomidae|[1616]|GMARA300-14|Diptera|Chironomidae||BOLD:ACM5571
Chironomidae|[1617]|GMAGI138-15|Diptera|Chironomidae||BOLD:ACM8565
Chironomidae|[1618]|GMAGS371-15|Diptera|Chironomidae||BOLD:ACX4709
Chironomidae|[1619]|GMARA209-14|Diptera|Chironomidae||BOLD:ACM6208
Chironomidae|[1620]|GMARO464-14|Diptera|Chironomidae||BOLD:ACX3750
Chironomidae|[1621]|GMAGR398-15|Diptera|Chironomidae||BOLD:ACX7356
Chironomidae|[1622]|GMARB1087-14|Diptera|Chironomidae||BOLD:ACM5329
Chironomidae|[1623]|GMAGD905-15|Diptera|Chironomidae||BOLD:ACW4418
Chironomidae|[1624]|GMAGF540-15|Diptera|Chironomidae||BOLD:ACW3479
Chironomidae|[1625]|GMARO691-14|Diptera|Chironomidae||BOLD:ACN9071
Chironomidae|[1626]|GMAGC174-15|Diptera|Chironomidae||BOLD:ACW4120
Chironomidae|[1627]|GMAGS704-15|Diptera|Chironomidae||BOLD:ACX6169
Chironomidae|[1628]|GMARB1464-14|Diptera|Chironomidae||BOLD:ACM6055
Chironomidae|[1629]|GMARQ548-14|Diptera|Chironomidae||BOLD:ACW3121
Chironomidae|[1630]|GMARN376-14|Diptera|Chironomidae||BOLD:ACN3414
Chironomidae|[1631]|GMARA1444-14|Diptera|Chironomidae||BOLD:ACM9916
Chironomidae|[1632]|GMAGD1192-15|Diptera|Chironomidae||BOLD:ACW3124
Chironomidae|[1633]|GMARI1524-14|Diptera|Chironomidae||BOLD:ACW4687
Chironomidae|[1634]|GMARQ154-14|Diptera|Chironomidae||BOLD:ACW4430
Chironomidae|[1635]|GMARA1244-14|Diptera|Chironomidae||BOLD:ACM4928
Chironomidae|[1636]|GMARA1064-14|Diptera|Chironomidae||BOLD:ACN1673
Chironomidae|[1637]|GMARJ429-14|Diptera|Chironomidae||BOLD:ACN0608
Chironomidae|[1638]|GMARL1249-14|Diptera|Chironomidae||BOLD:ACN2184
Chironomidae|[1639]|GMAGJ1721-15|Diptera|Chironomidae||BOLD:ACN9873
Chironomidae|[1640]|GMAGS976-15|Diptera|Chironomidae||BOLD:ACX4648
Chironomidae|[1641]|GMAGC1471-15|Diptera|Chironomidae||BOLD:ACO0120
Chironomidae|[1642]|GMAGA595-15|Diptera|Chironomidae||BOLD:ACM6268
Chironomidae|[1643]|GMARH323-14|Diptera|Chironomidae||BOLD:ACN0035
Chironomidae|[1644]|GMAGE1016-15|Diptera|Chironomidae||BOLD:ACW6036
Chironomidae|[1645]|GMARD1069-14|Diptera|Chironomidae||BOLD:ACM8624
Chironomidae|[1646]|GMAGL2685-15|Diptera|Chironomidae||BOLD:ACW8330
Chironomidae|[1647]|GMART1628-14|Diptera|Chironomidae||BOLD:ACN9344
Chironomidae|[1648]|GMARB1350-14|Diptera|Chironomidae||BOLD:ACM6130
Chironomidae|[1649]|GMARJ1544-14|Diptera|Chironomidae||BOLD:ACN0270
Chironomidae|[1650]|GMARA1201-14|Diptera|Chironomidae||BOLD:ACM4757
Chironomidae|[1651]|GMAGB596-15|Diptera|Chironomidae||BOLD:ACM8696
Chironomidae|[1652]|GMARP784-14|Diptera|Chironomidae||BOLD:ACO3466
Chironomidae|[1653]|GMAGE1738-15|Diptera|Chironomidae||BOLD:ACW2333
Chironomidae|[1654]|GMAGA417-15|Diptera|Chironomidae||BOLD:ACM5892
Chironomidae|[1655]|GMARB546-14|Diptera|Chironomidae||BOLD:ACM6537
Chironomidae|[1656]|GMAGD1224-15|Diptera|Chironomidae||BOLD:ACW3357
Chironomidae|[1657]|GMAGD2198-15|Diptera|Chironomidae||BOLD:ACM7294
Chironomidae|[1658]|GMAGH236-15|Diptera|Chironomidae||BOLD:ACW2029
Chironomidae|[1659]|GMAGI866-15|Diptera|Chironomidae||BOLD:ACW6798
Chironomidae|[1660]|GMARI304-14|Diptera|Chironomidae||BOLD:ACN0199
Chironomidae|[1661]|GMARM1049-14|Diptera|Chironomidae||BOLD:ACN9677
Chironomidae|[1662]|GMAGP1012-15|Diptera|Chironomidae||BOLD:ACN8479
Chironomidae|[1663]|GMARN267-14|Diptera|Chironomidae||BOLD:ACN3384
Chironomidae|[1664]|GMAGI230-15|Diptera|Chironomidae||BOLD:ACN1476
Chironomidae|[1665]|GMARA1173-14|Diptera|Chironomidae||BOLD:ACM8483
Chironomidae|[1666]|GMARA1257-14|Diptera|Chironomidae||BOLD:ACM9270
Chironomidae|[1667]|GMAGD2001-15|Diptera|Chironomidae||BOLD:ACM8475
Chironomidae|[1668]|GMARC072-14|Diptera|Chironomidae||BOLD:ACM5564
Chironomidae|[1669]|GMAGK1324-15|Diptera|Chironomidae||BOLD:ACW8071
Chironomidae|[1670]|GMARA1287-14|Diptera|Chironomidae||BOLD:ACM9332
Chironomidae|[1671]|GMARB699-14|Diptera|Chironomidae||BOLD:ACN1685
Chironomidae|[1672]|GMARI1004-14|Diptera|Chironomidae||BOLD:ACN1718
Chironomidae|[1673]|GMARG1565-14|Diptera|Chironomidae||BOLD:ACN0228
Chironomidae|[1674]|GMAGC858-15|Diptera|Chironomidae||BOLD:ACM8274
Chironomidae|[1675]|GMARI511-14|Diptera|Chironomidae||BOLD:ACN1684
Chironomidae|[1676]|GMAGM1168-15|Diptera|Chironomidae||BOLD:ACN9842
Chironomidae|[1677]|GMAGN1740-15|Diptera|Chironomidae||BOLD:ACX4338
Chironomidae|[1678]|GMARA1389-14|Diptera|Chironomidae||BOLD:ACM9550
Cricotopus|[1679]|GMARJ777-14|Diptera|Chironomidae|Orthocladiinae|BOLD:AAW4513
Chironomidae|[1680]|GMAGB386-15|Diptera|Chironomidae||BOLD:ACN2653
Chironomidae|[1681]|GMARH1042-14|Diptera|Chironomidae||BOLD:ACN0168
Chironomidae|[1682]|GMAGN1597-15|Diptera|Chironomidae||BOLD:ACX4741
Chironomidae|[1683]|GMAGF301-15|Diptera|Chironomidae||BOLD:ACW4450
Chironomidae|[1684]|GMAGP700-15|Diptera|Chironomidae||BOLD:ACX4688
Chironomidae|[1685]|GMARA1330-14|Diptera|Chironomidae||BOLD:ACM4761
Chironomidae|[1686]|GMARA103-14|Diptera|Chironomidae||BOLD:ACM6443
Chironomidae|[1687]|GMARO763-14|Diptera|Chironomidae||BOLD:ACM1566
Thienemanniella|[1688]|GMARK1349-14|Diptera|Chironomidae|Orthocladiinae|BOLD:AAW4714
Chironomidae|[1689]|GMARB1092-14|Diptera|Chironomidae||BOLD:ACM4888
Chironomidae|[1690]|GMAGD2207-15|Diptera|Chironomidae||BOLD:ACW2467
Chironomidae|[1691]|GMARA1777-14|Diptera|Chironomidae||BOLD:ACM5267
Thienemanniella|[1692]|GMARA253-14|Diptera|Chironomidae|Orthocladiinae|BOLD:AAD5820
Thienemanniella|[1693]|GMARD699-14|Diptera|Chironomidae|Orthocladiinae|BOLD:AAD5821

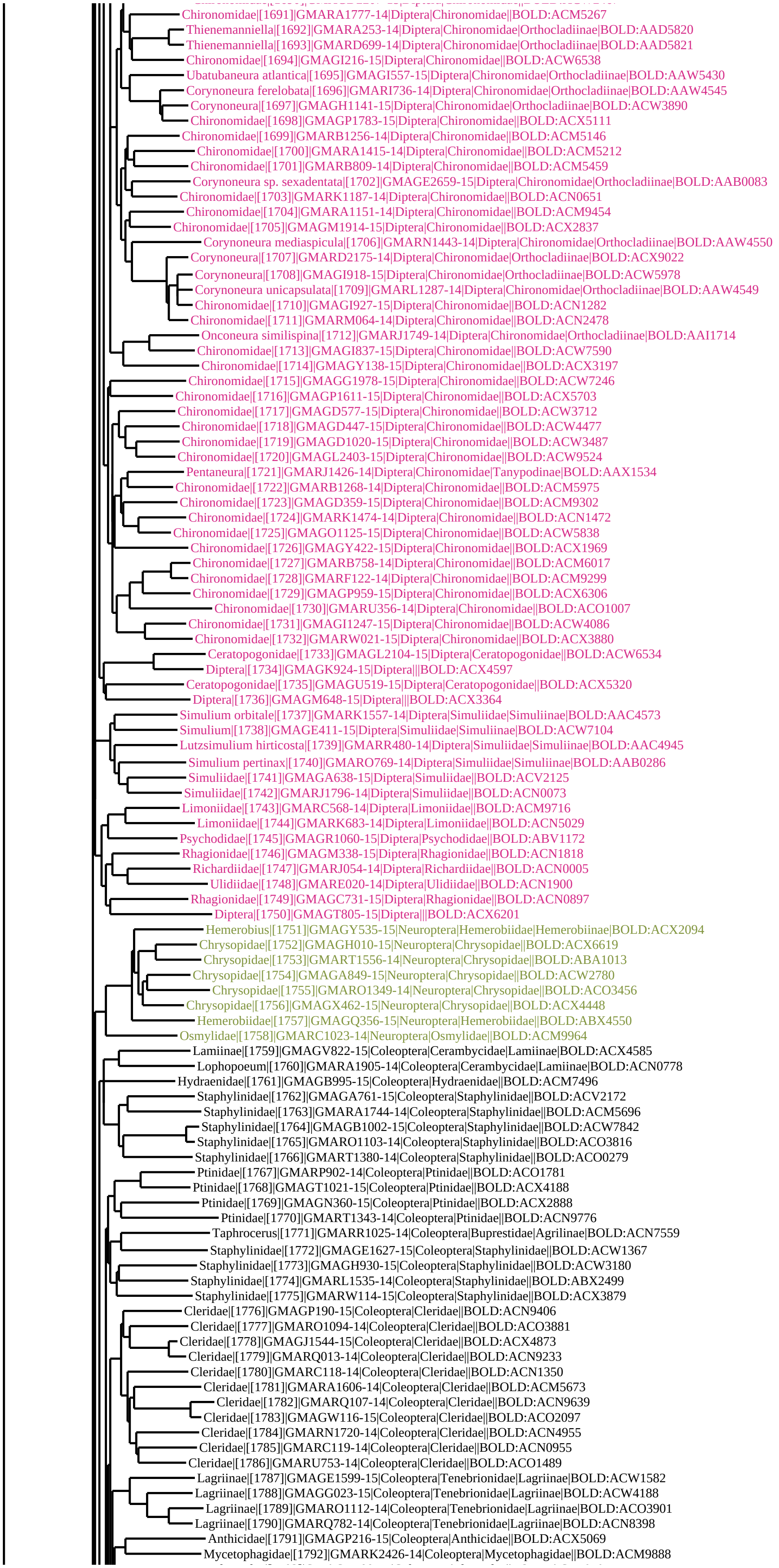
Chironomidae|[1691]|GMARA1777-14|Diptera|Chironomidae||BOLD:ACM5267
Thienemanniella|[1692]|GMARA253-14|Diptera|Chironomidae|Orthocladiinae|BOLD:AAD5820
Thienemanniella|[1693]|GMARD699-14|Diptera|Chironomidae|Orthocladiinae|BOLD:AAD5821
Chironomidae|[1694]|GMAGI216-15|Diptera|Chironomidae||BOLD:ACW6538
Ubatubaneura atlantica|[1695]|GMAGI557-15|Diptera|Chironomidae|Orthocladiinae|BOLD:AAW5430
Corynoneura ferelobata|[1696]|GMARI736-14|Diptera|Chironomidae|Orthocladiinae|BOLD:AAW4545
Corynoneura|[1697]|GMAGH1141-15|Diptera|Chironomidae|Orthocladiinae|BOLD:ACW3890
Chironomidae|[1698]|GMAGP1783-15|Diptera|Chironomidae||BOLD:ACX5111
Chironomidae|[1699]|GMARB1256-14|Diptera|Chironomidae||BOLD:ACM5146
Chironomidae|[1700]|GMARA1415-14|Diptera|Chironomidae||BOLD:ACM5212
Chironomidae|[1701]|GMARB809-14|Diptera|Chironomidae||BOLD:ACM5459
Corynoneura sp. sexadentata|[1702]|GMAGE2659-15|Diptera|Chironomidae|Orthocladiinae|BOLD:AAB0083
Chironomidae|[1703]|GMARK1187-14|Diptera|Chironomidae||BOLD:ACN0651
Chironomidae|[1704]|GMARA1151-14|Diptera|Chironomidae||BOLD:ACM9454
Chironomidae|[1705]|GMAGM1914-15|Diptera|Chironomidae||BOLD:ACX2837
Corynoneura mediaspicula|[1706]|GMARN1443-14|Diptera|Chironomidae|Orthocladiinae|BOLD:AAW4550
Corynoneura|[1707]|GMARD2175-14|Diptera|Chironomidae|Orthocladiinae|BOLD:ACX9022
Corynoneura|[1708]|GMAGI918-15|Diptera|Chironomidae|Orthocladiinae|BOLD:ACW5978
Corynoneura unicapsulata|[1709]|GMARL1287-14|Diptera|Chironomidae|Orthocladiinae|BOLD:AAW4549
Chironomidae|[1710]|GMAGI927-15|Diptera|Chironomidae||BOLD:ACN1282
Chironomidae|[1711]|GMARM064-14|Diptera|Chironomidae||BOLD:ACN2478
Onconeura similispina|[1712]|GMARJ1749-14|Diptera|Chironomidae|Orthocladiinae|BOLD:AAI1714
Chironomidae|[1713]|GMAGI837-15|Diptera|Chironomidae||BOLD:ACW7590
Chironomidae|[1714]|GMAGY138-15|Diptera|Chironomidae||BOLD:ACX3197
Chironomidae|[1715]|GMAGG1978-15|Diptera|Chironomidae||BOLD:ACW7246
Chironomidae|[1716]|GMAGP1611-15|Diptera|Chironomidae||BOLD:ACX5703
Chironomidae|[1717]|GMAGD577-15|Diptera|Chironomidae||BOLD:ACW3712
Chironomidae|[1718]|GMAGD447-15|Diptera|Chironomidae||BOLD:ACW4477
Chironomidae|[1719]|GMAGD1020-15|Diptera|Chironomidae||BOLD:ACW3487
Chironomidae|[1720]|GMAGL2403-15|Diptera|Chironomidae||BOLD:ACW9524
Pentaneura|[1721]|GMARJ1426-14|Diptera|Chironomidae|Tanypodinae|BOLD:AAX1534
Chironomidae|[1722]|GMARB1268-14|Diptera|Chironomidae||BOLD:ACM5975
Chironomidae|[1723]|GMAGD359-15|Diptera|Chironomidae||BOLD:ACM9302
Chironomidae|[1724]|GMARK1474-14|Diptera|Chironomidae||BOLD:ACN1472
Chironomidae|[1725]|GMAGO1125-15|Diptera|Chironomidae||BOLD:ACW5838
Chironomidae|[1726]|GMAGY422-15|Diptera|Chironomidae||BOLD:ACX1969
Chironomidae|[1727]|GMARB758-14|Diptera|Chironomidae||BOLD:ACM6017
Chironomidae|[1728]|GMARF122-14|Diptera|Chironomidae||BOLD:ACM9299
Chironomidae|[1729]|GMAGP959-15|Diptera|Chironomidae||BOLD:ACX6306
Chironomidae|[1730]|GMARU356-14|Diptera|Chironomidae||BOLD:ACO1007
Chironomidae|[1731]|GMAGI1247-15|Diptera|Chironomidae||BOLD:ACW4086
Chironomidae|[1732]|GMARW021-15|Diptera|Chironomidae||BOLD:ACX3880
Ceratopogonidae|[1733]|GMAGL2104-15|Diptera|Ceratopogonidae||BOLD:ACW6534
Diptera|[1734]|GMAGK924-15|Diptera|||BOLD:ACX4597
Ceratopogonidae|[1735]|GMAGU519-15|Diptera|Ceratopogonidae||BOLD:ACX5320
Diptera|[1736]|GMAGM648-15|Diptera|||BOLD:ACX3364
Simulium orbitale|[1737]|GMARK1557-14|Diptera|Simuliidae|Simuliinae|BOLD:AAC4573
Simulium|[1738]|GMAGE411-15|Diptera|Simuliidae|Simuliinae|BOLD:ACW7104
Lutzsimulium hirticosta|[1739]|GMARR480-14|Diptera|Simuliidae|Simuliinae|BOLD:AAC4945
Simulium pertinax|[1740]|GMARO769-14|Diptera|Simuliidae|Simuliinae|BOLD:AAB0286
Simuliidae|[1741]|GMAGA638-15|Diptera|Simuliidae||BOLD:ACV2125
Simuliidae|[1742]|GMARJ1796-14|Diptera|Simuliidae||BOLD:ACN0073
Limoniidae|[1743]|GMARC568-14|Diptera|Limoniidae||BOLD:ACM9716
Limoniidae|[1744]|GMARK683-14|Diptera|Limoniidae||BOLD:ACN5029
Psychodidae|[1745]|GMAGR1060-15|Diptera|Psychodidae||BOLD:ABV1172
Rhagionidae|[1746]|GMAGM338-15|Diptera|Rhagionidae||BOLD:ACN1818
Richardiidae|[1747]|GMARJ054-14|Diptera|Richardiidae||BOLD:ACN0005
Ulidiidae|[1748]|GMARE020-14|Diptera|Ulidiidae||BOLD:ACN1900
Rhagionidae|[1749]|GMAGC731-15|Diptera|Rhagionidae||BOLD:ACN0897
Diptera|[1750]|GMAGT805-15|Diptera|||BOLD:ACX6201
Hemerobius|[1751]|GMAGY535-15|Neuroptera|Hemerobiidae|Hemerobiinae|BOLD:ACX2094
Chrysopidae|[1752]|GMAGH010-15|Neuroptera|Chrysopidae||BOLD:ACX6619
Chrysopidae|[1753]|GMART1556-14|Neuroptera|Chrysopidae||BOLD:ABA1013
Chrysopidae|[1754]|GMAGA849-15|Neuroptera|Chrysopidae||BOLD:ACW2780
Chrysopidae|[1755]|GMARO1349-14|Neuroptera|Chrysopidae||BOLD:ACO3456
Chrysopidae|[1756]|GMAGX462-15|Neuroptera|Chrysopidae||BOLD:ACX4448
Hemerobiidae|[1757]|GMAGQ356-15|Neuroptera|Hemerobiidae||BOLD:ABX4550
Osmylidae|[1758]|GMARC1023-14|Neuroptera|Osmylidae||BOLD:ACM9964
Lamiinae|[1759]|GMAGV822-15|Coleoptera|Cerambycidae|Lamiinae|BOLD:ACX4585
Lophopoeum|[1760]|GMARA1905-14|Coleoptera|Cerambycidae|Lamiinae|BOLD:ACN0778
Hydraenidae|[1761]|GMAGB995-15|Coleoptera|Hydraenidae||BOLD:ACM7496
Staphylinidae|[1762]|GMAGA761-15|Coleoptera|Staphylinidae||BOLD:ACV2172
Staphylinidae|[1763]|GMARA1744-14|Coleoptera|Staphylinidae||BOLD:ACM5696
Staphylinidae|[1764]|GMAGB1002-15|Coleoptera|Staphylinidae||BOLD:ACW7842
Staphylinidae|[1765]|GMARO1103-14|Coleoptera|Staphylinidae||BOLD:ACO3816
Staphylinidae|[1766]|GMART1380-14|Coleoptera|Staphylinidae||BOLD:ACO0279
Ptinidae|[1767]|GMARP902-14|Coleoptera|Ptinidae||BOLD:ACO1781
Ptinidae|[1768]|GMAGT1021-15|Coleoptera|Ptinidae||BOLD:ACX4188
Ptinidae|[1769]|GMAGN360-15|Coleoptera|Ptinidae||BOLD:ACX2888
Ptinidae|[1770]|GMART1343-14|Coleoptera|Ptinidae||BOLD:ACN9776
Taphrocerus|[1771]|GMARR1025-14|Coleoptera|Buprestidae|Agrilinae|BOLD:ACN7559
Staphylinidae|[1772]|GMAGE1627-15|Coleoptera|Staphylinidae||BOLD:ACW1367
Staphylinidae|[1773]|GMAGH930-15|Coleoptera|Staphylinidae||BOLD:ACW3180
Staphylinidae|[1774]|GMARL1535-14|Coleoptera|Staphylinidae||BOLD:ABX2499
Staphylinidae|[1775]|GMARW114-15|Coleoptera|Staphylinidae||BOLD:ACX3879
Cleridae|[1776]|GMAGP190-15|Coleoptera|Cleridae||BOLD:ACN9406
Cleridae|[1777]|GMARO1094-14|Coleoptera|Cleridae||BOLD:ACO3881
Cleridae|[1778]|GMAGJ1544-15|Coleoptera|Cleridae||BOLD:ACX4873
Cleridae|[1779]|GMARQ013-14|Coleoptera|Cleridae||BOLD:ACN9233
Cleridae|[1780]|GMARC118-14|Coleoptera|Cleridae||BOLD:ACN1350
Cleridae|[1781]|GMARA1606-14|Coleoptera|Cleridae||BOLD:ACM5673
Cleridae|[1782]|GMARQ107-14|Coleoptera|Cleridae||BOLD:ACN9639
Cleridae|[1783]|GMAGW116-15|Coleoptera|Cleridae||BOLD:ACO2097
Cleridae|[1784]|GMARN1720-14|Coleoptera|Cleridae||BOLD:ACN4955
Cleridae|[1785]|GMARC119-14|Coleoptera|Cleridae||BOLD:ACN0955
Cleridae|[1786]|GMARU753-14|Coleoptera|Cleridae||BOLD:ACO1489
Lagriinae|[1787]|GMAGE1599-15|Coleoptera|Tenebrionidae|Lagriinae|BOLD:ACW1582
Lagriinae|[1788]|GMAGG023-15|Coleoptera|Tenebrionidae|Lagriinae|BOLD:ACW4188
Lagriinae|[1789]|GMARO1112-14|Coleoptera|Tenebrionidae|Lagriinae|BOLD:ACO3901
Lagriinae|[1790]|GMARQ782-14|Coleoptera|Tenebrionidae|Lagriinae|BOLD:ACN8398
Anthicidae|[1791]|GMAGP216-15|Coleoptera|Anthicidae||BOLD:ACX5069
Mycetophagidae|[1792]|GMARK2426-14|Coleoptera|Mycetophagidae||BOLD:ACM9888

Lagriinae|[1790]|GMARQ782-14|Coleoptera|Tenebrionidae|Lagriinae|BOLD:ACN8398
Anthicidae|[1791]|GMAGP216-15|Coleoptera|Anthicidae||BOLD:ACX5069
Mycetophagidae|[1792]|GMARK2426-14|Coleoptera|Mycetophagidae||BOLD:ACM9888
Elateridae|[1793]|GMAGV799-15|Coleoptera|Elateridae||BOLD:ACX5050
Elateridae|[1794]|GMAGA766-15|Coleoptera|Elateridae||BOLD:ACN8602
Agrypninae|[1795]|GMAGB744-15|Coleoptera|Elateridae|Agrypninae|BOLD:ABV0859
Elateridae|[1796]|GMARJ002-14|Coleoptera|Elateridae||BOLD:ACN5698
Elateridae|[1797]|GMARR102-14|Coleoptera|Elateridae||BOLD:ACN8980
Elateridae|[1798]|GMAGW118-15|Coleoptera|Elateridae||BOLD:ACO1983
Elateridae|[1799]|GMARN001-14|Coleoptera|Elateridae||BOLD:ACO1149
Elateridae|[1800]|GMAGR223-15|Coleoptera|Elateridae||BOLD:ACO0980
Elateridae|[1801]|GMAGQ275-15|Coleoptera|Elateridae||BOLD:ACX7147
Elateridae|[1802]|GMAGU635-15|Coleoptera|Elateridae||BOLD:ACX6180
Elateridae|[1803]|GMAGT1247-15|Coleoptera|Elateridae||BOLD:ACX7214
Elateridae|[1804]|GMAGS1214-15|Coleoptera|Elateridae||BOLD:ACO0520
Elateridae|[1805]|GMARQ009-14|Coleoptera|Elateridae||BOLD:ACN9485
Elateridae|[1806]|GMARS556-14|Coleoptera|Elateridae||BOLD:ACN8646
Elateridae|[1807]|GMAGX126-15|Coleoptera|Elateridae||BOLD:ACX2545
Tenebrionidae|[1808]|GMARV134-14|Coleoptera|Tenebrionidae||BOLD:ACO1655
Tenebrionidae|[1809]|GMARV135-14|Coleoptera|Tenebrionidae||BOLD:ACO2185
Cerambycinae|[1810]|GMAGX432-15|Coleoptera|Cerambycidae|Cerambycinae|BOLD:ACX6027
Cis|[1811]|GMAGD1712-15|Coleoptera|Ciidae|Ciinae|BOLD:ACW4902
Compsocerus violaceus|[1812]|GMAGS188-15|Coleoptera|Cerambycidae|Cerambycinae|BOLD:AAI9184
Cerambycinae|[1813]|GMARM1551-14|Coleoptera|Cerambycidae|Cerambycinae|BOLD:ACN4188
Cerambycinae|[1814]|GMARP1231-14|Coleoptera|Cerambycidae|Cerambycinae|BOLD:ACN8988
Cerambycinae|[1815]|GMARO1410-14|Coleoptera|Cerambycidae|Cerambycinae|BOLD:ACN9341
Megacyllene|[1816]|GMAGU123-15|Coleoptera|Cerambycidae|Cerambycinae|BOLD:ACX7571
Cerambycinae|[1817]|GMARP1236-14|Coleoptera|Cerambycidae|Cerambycinae|BOLD:ACN9120
Chlorida|[1818]|GMARR097-14|Coleoptera|Cerambycidae|Cerambycinae|BOLD:ACN9132
Cerambycinae|[1819]|GMAGF002-15|Coleoptera|Cerambycidae|Cerambycinae|BOLD:ACN3889
Cerambycidae|[1820]|GMAGT227-15|Coleoptera|Cerambycidae||BOLD:ACN9397
Cerambycinae|[1821]|GMARM1530-14|Coleoptera|Cerambycidae|Cerambycinae|BOLD:ACN4654
Cerambycinae|[1822]|GMAGU829-15|Coleoptera|Cerambycidae|Cerambycinae|BOLD:ACX7047
Cerambycidae|[1823]|GMAGV985-15|Coleoptera|Cerambycidae||BOLD:ACO1428
Elateridae|[1824]|GMARR990-14|Coleoptera|Elateridae||BOLD:ACN7620
Elateridae|[1825]|GMARS612-14|Coleoptera|Elateridae||BOLD:ACO1771
Elateridae|[1826]|GMAGV972-15|Coleoptera|Elateridae||BOLD:ACX4142
Elateridae|[1827]|GMARP1242-14|Coleoptera|Elateridae||BOLD:ACO0468
Elateridae|[1828]|GMARQ789-14|Coleoptera|Elateridae||BOLD:ACN9618
Elateridae|[1829]|GMART1723-14|Coleoptera|Elateridae||BOLD:ACO2243
Elateridae|[1830]|GMAGY088-15|Coleoptera|Elateridae||BOLD:ACX5519
Elateridae|[1831]|GMARV128-14|Coleoptera|Elateridae||BOLD:ACO2306
Meloidae|[1832]|GMARP1244-14|Coleoptera|Meloidae||BOLD:ACN9329
Scarabaeidae|[1833]|GMARS925-14|Coleoptera|Scarabaeidae||BOLD:ACO2231
Tenebrionidae|[1834]|GMAGB749-15|Coleoptera|Tenebrionidae||BOLD:ACX0052
Tenebrionidae|[1835]|GMARP1243-14|Coleoptera|Tenebrionidae||BOLD:ACX7153
Tenebrionidae|[1836]|GMARO1383-14|Coleoptera|Tenebrionidae||BOLD:ACO1019
Tenebrionidae|[1837]|GMAGA211-15|Coleoptera|Tenebrionidae||BOLD:ACW2905
Tenebrionidae|[1838]|GMARM1529-14|Coleoptera|Tenebrionidae||BOLD:ACN6564
Tenebrionidae|[1839]|GMARV130-14|Coleoptera|Tenebrionidae||BOLD:ACO2084
Mycteridae|[1840]|GMAGP189-15|Coleoptera|Mycteridae||BOLD:ACX5561
Mycteridae|[1841]|GMAGQ292-15|Coleoptera|Mycteridae||BOLD:ACX5187
Coleoptera|[1842]|GMAGR207-15|Coleoptera|||BOLD:ACN8950
Anthribidae|[1843]|GMARQ674-14|Coleoptera|Anthribidae||BOLD:ACN7633
Anthribidae|[1844]|GMART064-14|Coleoptera|Anthribidae||BOLD:ACO2215
Anthribidae|[1845]|GMART1704-14|Coleoptera|Anthribidae||BOLD:ACO1551
Anthribidae|[1846]|GMAGB747-15|Coleoptera|Anthribidae||BOLD:ACN1008
Anthribidae|[1847]|GMARE1406-14|Coleoptera|Anthribidae||BOLD:ACN4590
Anthribidae|[1848]|GMAGY089-15|Coleoptera|Anthribidae||BOLD:ACX5401
Brentidae|[1849]|GMARE1274-14|Coleoptera|Brentidae||BOLD:ACM7577
Brentidae|[1850]|GMARF1316-14|Coleoptera|Brentidae||BOLD:ACN0973
Brentidae|[1851]|GMAGK085-15|Coleoptera|Brentidae||BOLD:ACW5990
Brentidae|[1852]|GMAGF639-15|Coleoptera|Brentidae||BOLD:ACM7794
Brentidae|[1853]|GMARE1279-14|Coleoptera|Brentidae||BOLD:ACW8693
Brentidae|[1854]|GMAGI1181-15|Coleoptera|Brentidae||BOLD:ACW6777
Brentidae|[1855]|GMARK2420-14|Coleoptera|Brentidae||BOLD:ACN0736
Entiminae|[1856]|GMARK2381-14|Coleoptera|Curculionidae|Entiminae|BOLD:ABW4882
Promecops|[1857]|GMARP968-14|Coleoptera|Curculionidae|Entiminae|BOLD:ABW2078
Curculionidae|[1858]|GMAGA212-15|Coleoptera|Curculionidae||BOLD:ACW3956
Curculionidae|[1859]|GMARO1096-14|Coleoptera|Curculionidae||BOLD:ACO3210
Mimographus ocellatus|[1860]|GMARN2045-14|Coleoptera|Curculionidae|Entiminae|BOLD:ABW2003
Curculionidae|[1861]|GMARR106-14|Coleoptera|Curculionidae||BOLD:ACO0270
Curculionidae|[1862]|GMARM1536-14|Coleoptera|Curculionidae||BOLD:ACN6464
Baridinae|[1863]|GMARN1704-14|Coleoptera|Curculionidae|Baridinae|BOLD:ABW2396
Curculionidae|[1864]|GMARF1400-14|Coleoptera|Curculionidae||BOLD:ACN1263
Curculionidae|[1865]|GMARK2380-14|Coleoptera|Curculionidae||BOLD:ACN1397
Curculionidae|[1866]|GMARL1553-14|Coleoptera|Curculionidae||BOLD:ACN2896
Listronotus bonariensis|[1867]|GMAGN310-15|Coleoptera|Curculionidae|Cyclominae|BOLD:ACI2251
Curculionidae|[1868]|GMARA1605-14|Coleoptera|Curculionidae||BOLD:ACM5526
Curculionidae|[1869]|GMARW262-15|Coleoptera|Curculionidae||BOLD:ACX6292
Curculionidae|[1870]|GMAGT057-15|Coleoptera|Curculionidae||BOLD:ACX6472
Curculionidae|[1871]|GMARM1340-14|Coleoptera|Curculionidae||BOLD:ACN2161
Curculionidae|[1872]|GMAGN330-15|Coleoptera|Curculionidae||BOLD:ACN0566
Curculionidae|[1873]|GMARK2459-14|Coleoptera|Curculionidae||BOLD:ACN2816
Curculionidae|[1874]|GMARL1585-14|Coleoptera|Curculionidae||BOLD:ACN2098
Curculionidae|[1875]|GMARO1132-14|Coleoptera|Curculionidae||BOLD:ACN8803
Curculionidae|[1876]|GMAGX117-15|Coleoptera|Curculionidae||BOLD:ACX1437
Curculionidae|[1877]|GMAGM1824-15|Coleoptera|Curculionidae||BOLD:ACX3894
Scolytinae|[1878]|GMAGE1647-15|Coleoptera|Curculionidae|Scolytinae|BOLD:ACN1102
Scolytinae|[1879]|GMAGH948-15|Coleoptera|Curculionidae|Scolytinae|BOLD:ACN1214
Scolytinae|[1880]|GMAGE1616-15|Coleoptera|Curculionidae|Scolytinae|BOLD:ACW2503
Scolytinae|[1881]|GMARB080-14|Coleoptera|Curculionidae|Scolytinae|BOLD:ACM5389
Scolytinae|[1882]|GMAGK132-15|Coleoptera|Curculionidae|Scolytinae|BOLD:ACW5942
Scolytinae|[1883]|GMARF1334-14|Coleoptera|Curculionidae|Scolytinae|BOLD:ACN1822
Scolytinae|[1884]|GMARL1416-14|Coleoptera|Curculionidae|Scolytinae|BOLD:ACN4031
Scolytinae|[1885]|GMAGC1291-15|Coleoptera|Curculionidae|Scolytinae|BOLD:ACV7919
Scolytinae|[1886]|GMAGN315-15|Coleoptera|Curculionidae|Scolytinae|BOLD:ACX2394
Scolytinae|[1887]|GMAGJ1395-15|Coleoptera|Curculionidae|Scolytinae|BOLD:ACW9291
Scolytinae|[1888]|GMAGQ671-15|Coleoptera|Curculionidae|Scolytinae|BOLD:ACX4641
Scolytinae|[1889]|GMAGQ289-15|Coleoptera|Curculionidae|Scolytinae|BOLD:ACX5618
Scolytinae|[1890]|GMARO1113-14|Coleoptera|Curculionidae|Scolytinae|BOLD:ACO3817
Scolytinae|[1891]|GMAGV955-15|Coleoptera|Curculionidae|Scolytinae|BOLD:ACO0236
Curculionidae|[1892]|GMAGT1057-15|Coleoptera|Curculionidae||BOLD:ACX4881

Scolytinae|[1890]|GMARO1113-14|Coleoptera|Curculionidae|Scolytinae|BOLD:ACO3817
Scolytinae|[1891]|GMAGV955-15|Coleoptera|Curculionidae|Scolytinae|BOLD:ACO0236
Curculionidae|[1892]|GMAGT1057-15|Coleoptera|Curculionidae||BOLD:ACX4881
Curculionidae|[1893]|GMARJ1855-14|Coleoptera|Curculionidae||BOLD:ACN1580
Curculionidae|[1894]|GMAGO152-15|Coleoptera|Curculionidae||BOLD:ACX4107
Curculionidae|[1895]|GMARD055-14|Coleoptera|Curculionidae||BOLD:ACN1462
Curculionidae|[1896]|GMAGQ118-15|Coleoptera|Curculionidae||BOLD:ACX7706
Curculionidae|[1897]|GMAGS1208-15|Coleoptera|Curculionidae||BOLD:ACX6107
Curculionidae|[1898]|GMAGH005-15|Coleoptera|Curculionidae||BOLD:ACX7247
Curculionidae|[1899]|GMARF107-14|Coleoptera|Curculionidae||BOLD:ACO1887
Curculionidae|[1900]|GMAGT1061-15|Coleoptera|Curculionidae||BOLD:ACX5766
Conotrachelus|[1901]|GMAGQ637-15|Coleoptera|Curculionidae|Molytinae|BOLD:ABW4646
Curculionidae|[1902]|GMAGP221-15|Coleoptera|Curculionidae||BOLD:ACX5459
Curculionidae|[1903]|GMAGG020-15|Coleoptera|Curculionidae||BOLD:ACW3815
Curculionidae|[1904]|GMARS535-14|Coleoptera|Curculionidae||BOLD:ACN8084
Curculionidae|[1905]|GMARU685-14|Coleoptera|Curculionidae||BOLD:ACO1141
Molytinae|[1906]|GMARQ106-14|Coleoptera|Curculionidae|Molytinae|BOLD:ABW5049
Curculionidae|[1907]|GMARK052-14|Coleoptera|Curculionidae||BOLD:ACN6315
Curculionidae|[1908]|GMARW257-15|Coleoptera|Curculionidae||BOLD:ACX4506
Curculionidae|[1909]|GMAGS194-15|Coleoptera|Curculionidae||BOLD:ACT4237
Curculionidae|[1910]|GMARR093-14|Coleoptera|Curculionidae||BOLD:ACN9859
Curculionidae|[1911]|GMAGW114-15|Coleoptera|Curculionidae||BOLD:ACX6093
Curculionidae|[1912]|GMARP015-14|Coleoptera|Curculionidae||BOLD:ACO1202
Curculionidae|[1913]|GMARO1128-14|Coleoptera|Curculionidae||BOLD:ACO3670
Curculionidae|[1914]|GMARW120-15|Coleoptera|Curculionidae||BOLD:ACX3615
Chrysomelidae|[1915]|GMARS571-14|Coleoptera|Chrysomelidae||BOLD:ACN8443
Laemophloeidae|[1916]|GMARM1344-14|Coleoptera|Laemophloeidae||BOLD:ACN2470
Phalacridae|[1917]|GMAGP239-15|Coleoptera|Phalacridae||BOLD:ACX6083
Phalacridae|[1918]|GMAGB144-15|Coleoptera|Phalacridae||BOLD:ACW4877
Phalacridae|[1919]|GMAGP197-15|Coleoptera|Phalacridae||BOLD:ACX5490
Phalacridae|[1920]|GMARQ679-14|Coleoptera|Phalacridae||BOLD:ACN8362
Phalacridae|[1921]|GMARI1350-14|Coleoptera|Phalacridae||BOLD:ACN1408
Phalacridae|[1922]|GMAGH939-15|Coleoptera|Phalacridae||BOLD:ACW3339
Phalacridae|[1923]|GMARF1401-14|Coleoptera|Phalacridae||BOLD:ACN1440
Phalacridae|[1924]|GMAGV804-15|Coleoptera|Phalacridae||BOLD:ACX4803
Phalacridae|[1925]|GMART1344-14|Coleoptera|Phalacridae||BOLD:ACN9840
Stylogaster|[1926]|GMARF207-14|Diptera|Conopidae|Stylogastrinae|BOLD:ACN2492
Stylogaster|[1927]|GMAGK683-15|Diptera|Conopidae|Stylogastrinae|BOLD:ACN6038
Coccinellidae|[1928]|GMARR1026-14|Coleoptera|Coccinellidae||BOLD:ACN7806
Corylophidae|[1929]|GMAGN348-15|Coleoptera|Corylophidae||BOLD:ACN7856
Leiodidae|[1930]|GMAGQ625-15|Coleoptera|Leiodidae||BOLD:ACX5131
Chrysomelidae|[1931]|GMARI037-14|Coleoptera|Chrysomelidae||BOLD:ACN5339
Chrysomelidae|[1932]|GMARK057-14|Coleoptera|Chrysomelidae||BOLD:ACN6126
Chrysomelidae|[1933]|GMARM1550-14|Coleoptera|Chrysomelidae||BOLD:ACE1459
Chrysomelidae|[1934]|GMAGN319-15|Coleoptera|Chrysomelidae||BOLD:ACX3302
Chrysomelidae|[1935]|GMAGB987-15|Coleoptera|Chrysomelidae||BOLD:ACM5516
Chrysomelidae|[1936]|GMARM1348-14|Coleoptera|Chrysomelidae||BOLD:ACN2136
Cantharidae|[1937]|GMAGX441-15|Coleoptera|Cantharidae||BOLD:ABV0518
Lampyridae|[1938]|GMARR101-14|Coleoptera|Lampyridae||BOLD:ACO0287
Lampyridae|[1939]|GMAGY086-15|Coleoptera|Lampyridae||BOLD:ACX4143
Anthribidae|[1940]|GMARM1356-14|Coleoptera|Anthribidae||BOLD:ACN2451
Cantharidae|[1941]|GMARR912-14|Coleoptera|Cantharidae||BOLD:ACN9887
Cantharidae|[1942]|GMAGV047-15|Coleoptera|Cantharidae||BOLD:ACX5721
Cantharidae|[1943]|GMARM1546-14|Coleoptera|Cantharidae||BOLD:ACN4613
Cantharidae|[1944]|GMARS091-14|Coleoptera|Cantharidae||BOLD:ABW2642
Cantharidae|[1945]|GMAGF003-15|Coleoptera|Cantharidae||BOLD:ACN0419
Cantharidae|[1946]|GMARA1908-14|Coleoptera|Cantharidae||BOLD:ACN1794
Cantharidae|[1947]|GMARS572-14|Coleoptera|Cantharidae||BOLD:ACN7912
Lampyridae|[1948]|GMARP1230-14|Coleoptera|Lampyridae||BOLD:ACO0164
Lampyridae|[1949]|GMARQ797-14|Coleoptera|Lampyridae||BOLD:ACN9174
Lampyridae|[1950]|GMARR913-14|Coleoptera|Lampyridae||BOLD:ACN7801
Lycidae|[1951]|GMARF104-14|Coleoptera|Lycidae||BOLD:ACO0210
Lycidae|[1952]|GMAGT048-15|Coleoptera|Lycidae||BOLD:ACX7119
Lycidae|[1953]|GMAGD076-15|Coleoptera|Lycidae||BOLD:ABW8706
Lycidae|[1954]|GMARO1377-14|Coleoptera|Lycidae||BOLD:ACO1195
Lycidae|[1955]|GMAGU830-15|Coleoptera|Lycidae||BOLD:ACX6727
Lycidae|[1956]|GMARD057-14|Coleoptera|Lycidae||BOLD:ABW4926
Lycidae|[1957]|GMAGR040-15|Coleoptera|Lycidae||BOLD:ACX6378
Lycidae|[1958]|GMAGS207-15|Coleoptera|Lycidae||BOLD:ACX6813
Lycidae|[1959]|GMAGG447-15|Coleoptera|Lycidae||BOLD:ACX7107
Lycidae|[1960]|GMAGV980-15|Coleoptera|Lycidae||BOLD:ACX6950
Mordellinae|[1961]|GMARB028-14|Coleoptera|Mordellidae|Mordellinae|BOLD:ACN8085
Mordellinae|[1962]|GMARR997-14|Coleoptera|Mordellidae|Mordellinae|BOLD:ACN7456
Mordellinae|[1963]|GMARS552-14|Coleoptera|Mordellidae|Mordellinae|BOLD:ACN7728
Mordellinae|[1964]|GMARS533-14|Coleoptera|Mordellidae|Mordellinae|BOLD:ACN7454
Mordellinae|[1965]|GMART1347-14|Coleoptera|Mordellidae|Mordellinae|BOLD:ACN9118
Mordellinae|[1966]|GMARS549-14|Coleoptera|Mordellidae|Mordellinae|BOLD:ACN7490
Mordellinae|[1967]|GMARB035-14|Coleoptera|Mordellidae|Mordellinae|BOLD:ACN0798
Mordellinae|[1968]|GMAGD1690-15|Coleoptera|Mordellidae|Mordellinae|BOLD:ACY6672
Mordellinae|[1969]|GMARR1046-14|Coleoptera|Mordellidae|Mordellinae|BOLD:ACX2884
Mordellinae|[1970]|GMAGV803-15|Coleoptera|Mordellidae|Mordellinae|BOLD:ACX5562
Mordellinae|[1971]|GMAGY323-15|Coleoptera|Mordellidae|Mordellinae|BOLD:ACX1543
Mordellinae|[1972]|GMARN1680-14|Coleoptera|Mordellidae|Mordellinae|BOLD:ACN3370
Mordellinae|[1973]|GMAGF626-15|Coleoptera|Mordellidae|Mordellinae|BOLD:ACO0710
Mordellinae|[1974]|GMARR929-14|Coleoptera|Mordellidae|Mordellinae|BOLD:ACN9865
Mordellinae|[1975]|GMAGX118-15|Coleoptera|Mordellidae|Mordellinae|BOLD:ACX2963
Mordellinae|[1976]|GMAGU636-15|Coleoptera|Mordellidae|Mordellinae|BOLD:ACN8852
Mordellinae|[1977]|GMAGX123-15|Coleoptera|Mordellidae|Mordellinae|BOLD:ACW9568
Mordellidae|[1978]|GMAGU629-15|Coleoptera|Mordellidae||BOLD:ACX5341
Mordellidae|[1979]|GMAGS1225-15|Coleoptera|Mordellidae||BOLD:ACX5551
Mordellidae|[1980]|GMAGU644-15|Coleoptera|Mordellidae||BOLD:ACX5963
Mordellinae|[1981]|GMARR1019-14|Coleoptera|Mordellidae|Mordellinae|BOLD:ACN8570
Mordellinae|[1982]|GMARO1127-14|Coleoptera|Mordellidae|Mordellinae|BOLD:ACO3478
Mordellinae|[1983]|GMARR1013-14|Coleoptera|Mordellidae|Mordellinae|BOLD:ACN7697
Mordellinae|[1984]|GMAGS1237-15|Coleoptera|Mordellidae|Mordellinae|BOLD:ACX4113
Mordellinae|[1985]|GMAGT1070-15|Coleoptera|Mordellidae|Mordellinae|BOLD:ACX4907
Mordellinae|[1986]|GMAGV771-15|Coleoptera|Mordellidae|Mordellinae|BOLD:ACX5662
Mordellinae|[1987]|GMAGV808-15|Coleoptera|Mordellidae|Mordellinae|BOLD:ACN9504
Mordellinae|[1988]|GMAGE1605-15|Coleoptera|Mordellidae|Mordellinae|BOLD:ACN9696
Mordellinae|[1989]|GMAGP228-15|Coleoptera|Mordellidae|Mordellinae|BOLD:ACX1135
Mordellinae|[1990]|GMAGQ621-15|Coleoptera|Mordellidae|Mordellinae|BOLD:ACX1176
Mordellistena|[1991]|GMAGT1050-15|Coleoptera|Mordellidae|Mordellinae|BOLD:ACO0342
[illegible]

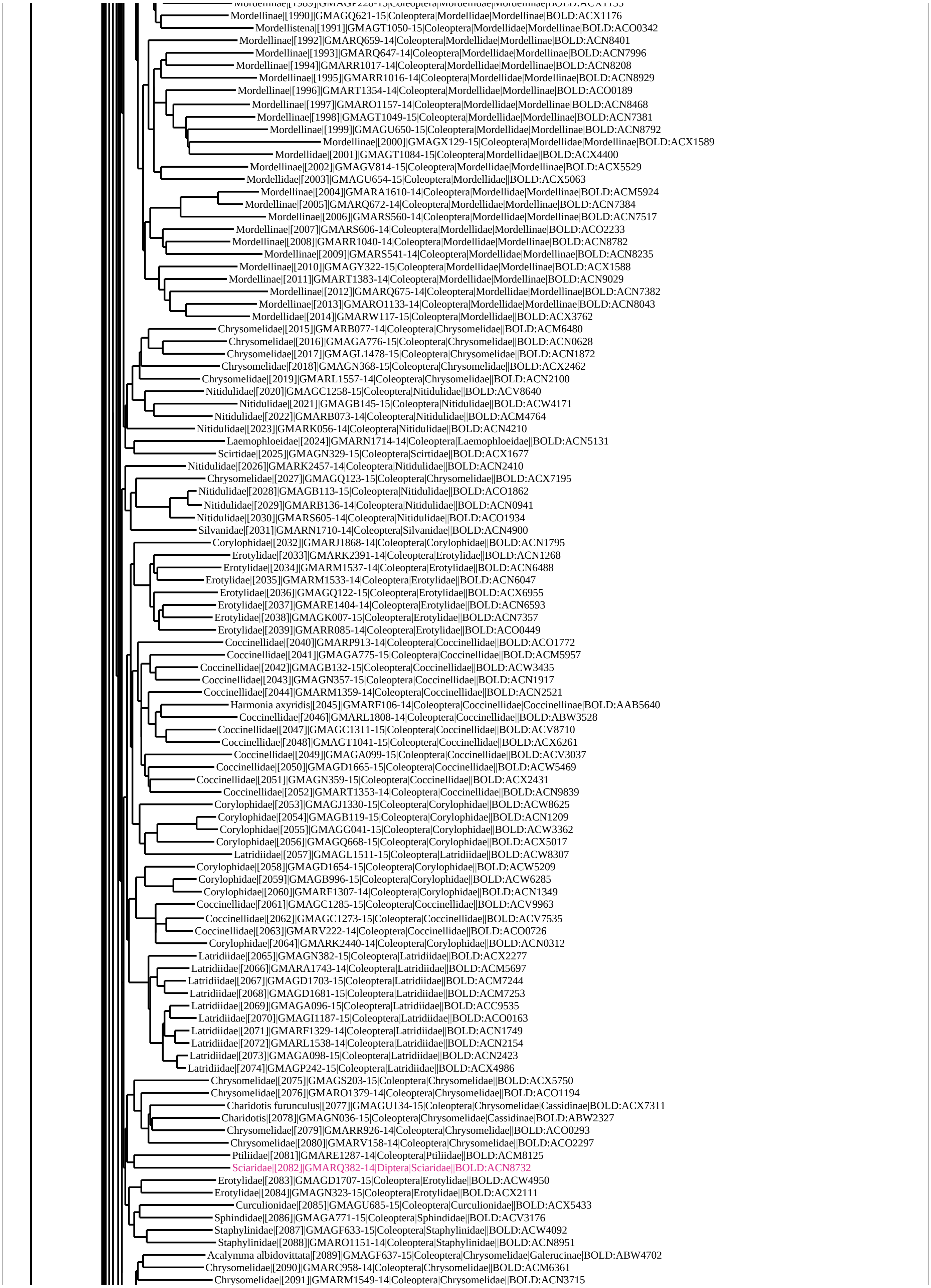
Mordellinae|[1989]|GMAGP228-15|Coleoptera|Mordellidae|Mordellinae|BOLD:ACX1135
Mordellinae|[1990]|GMAGQ621-15|Coleoptera|Mordellidae|Mordellinae|BOLD:ACX1176
Mordellistena|[1991]|GMAGT1050-15|Coleoptera|Mordellidae|Mordellinae|BOLD:ACO0342
Mordellinae|[1992]|GMARQ659-14|Coleoptera|Mordellidae|Mordellinae|BOLD:ACN8401
Mordellinae|[1993]|GMARQ647-14|Coleoptera|Mordellidae|Mordellinae|BOLD:ACN7996
Mordellinae|[1994]|GMARR1017-14|Coleoptera|Mordellidae|Mordellinae|BOLD:ACN8208
Mordellinae|[1995]|GMARR1016-14|Coleoptera|Mordellidae|Mordellinae|BOLD:ACN8929
Mordellinae|[1996]|GMART1354-14|Coleoptera|Mordellidae|Mordellinae|BOLD:ACO0189
Mordellinae|[1997]|GMARO1157-14|Coleoptera|Mordellidae|Mordellinae|BOLD:ACN8468
Mordellinae|[1998]|GMAGT1049-15|Coleoptera|Mordellidae|Mordellinae|BOLD:ACN7381
Mordellinae|[1999]|GMAGU650-15|Coleoptera|Mordellidae|Mordellinae|BOLD:ACN8792
Mordellinae|[2000]|GMAGX129-15|Coleoptera|Mordellidae|Mordellinae|BOLD:ACX1589
Mordellidae|[2001]|GMAGT1084-15|Coleoptera|Mordellidae||BOLD:ACX4400
Mordellinae|[2002]|GMAGV814-15|Coleoptera|Mordellidae|Mordellinae|BOLD:ACX5529
Mordellidae|[2003]|GMAGU654-15|Coleoptera|Mordellidae||BOLD:ACX5063
Mordellinae|[2004]|GMARA1610-14|Coleoptera|Mordellidae|Mordellinae|BOLD:ACM5924
Mordellinae|[2005]|GMARQ672-14|Coleoptera|Mordellidae|Mordellinae|BOLD:ACN7384
Mordellinae|[2006]|GMARS560-14|Coleoptera|Mordellidae|Mordellinae|BOLD:ACN7517
Mordellinae|[2007]|GMARS606-14|Coleoptera|Mordellidae|Mordellinae|BOLD:ACO2233
Mordellinae|[2008]|GMARR1040-14|Coleoptera|Mordellidae|Mordellinae|BOLD:ACN8782
Mordellinae|[2009]|GMARS541-14|Coleoptera|Mordellidae|Mordellinae|BOLD:ACN8235
Mordellinae|[2010]|GMAGY322-15|Coleoptera|Mordellidae|Mordellinae|BOLD:ACX1588
Mordellinae|[2011]|GMART1383-14|Coleoptera|Mordellidae|Mordellinae|BOLD:ACN9029
Mordellinae|[2012]|GMARQ675-14|Coleoptera|Mordellidae|Mordellinae|BOLD:ACN7382
Mordellinae|[2013]|GMARO1133-14|Coleoptera|Mordellidae|Mordellinae|BOLD:ACN8043
Mordellidae|[2014]|GMARW117-15|Coleoptera|Mordellidae||BOLD:ACX3762
Chrysomelidae|[2015]|GMARB077-14|Coleoptera|Chrysomelidae||BOLD:ACM6480
Chrysomelidae|[2016]|GMAGA776-15|Coleoptera|Chrysomelidae||BOLD:ACN0628
Chrysomelidae|[2017]|GMAGL1478-15|Coleoptera|Chrysomelidae||BOLD:ACN1872
Chrysomelidae|[2018]|GMAGN368-15|Coleoptera|Chrysomelidae||BOLD:ACX2462
Chrysomelidae|[2019]|GMARL1557-14|Coleoptera|Chrysomelidae||BOLD:ACN2100
Nitidulidae|[2020]|GMAGC1258-15|Coleoptera|Nitidulidae||BOLD:ACV8640
Nitidulidae|[2021]|GMAGB145-15|Coleoptera|Nitidulidae||BOLD:ACW4171
Nitidulidae|[2022]|GMARB073-14|Coleoptera|Nitidulidae||BOLD:ACM4764
Nitidulidae|[2023]|GMARK056-14|Coleoptera|Nitidulidae||BOLD:ACN4210
Laemophloeidae|[2024]|GMARN1714-14|Coleoptera|Laemophloeidae||BOLD:ACN5131
Scirtidae|[2025]|GMAGN329-15|Coleoptera|Scirtidae||BOLD:ACX1677
Nitidulidae|[2026]|GMARK2457-14|Coleoptera|Nitidulidae||BOLD:ACN2410
Chrysomelidae|[2027]|GMAGQ123-15|Coleoptera|Chrysomelidae||BOLD:ACX7195
Nitidulidae|[2028]|GMAGB113-15|Coleoptera|Nitidulidae||BOLD:ACO1862
Nitidulidae|[2029]|GMARB136-14|Coleoptera|Nitidulidae||BOLD:ACN0941
Nitidulidae|[2030]|GMARS605-14|Coleoptera|Nitidulidae||BOLD:ACO1934
Silvanidae|[2031]|GMARN1710-14|Coleoptera|Silvanidae||BOLD:ACN4900
Corylophidae|[2032]|GMARJ1868-14|Coleoptera|Corylophidae||BOLD:ACN1795
Erotylidae|[2033]|GMARK2391-14|Coleoptera|Erotylidae||BOLD:ACN1268
Erotylidae|[2034]|GMARM1537-14|Coleoptera|Erotylidae||BOLD:ACN6488
Erotylidae|[2035]|GMARM1533-14|Coleoptera|Erotylidae||BOLD:ACN6047
Erotylidae|[2036]|GMAGQ122-15|Coleoptera|Erotylidae||BOLD:ACX6955
Erotylidae|[2037]|GMARE1404-14|Coleoptera|Erotylidae||BOLD:ACN6593
Erotylidae|[2038]|GMAGK007-15|Coleoptera|Erotylidae||BOLD:ACN7357
Erotylidae|[2039]|GMARR085-14|Coleoptera|Erotylidae||BOLD:ACO0449
Coccinellidae|[2040]|GMARP913-14|Coleoptera|Coccinellidae||BOLD:ACO1772
Coccinellidae|[2041]|GMAGA775-15|Coleoptera|Coccinellidae||BOLD:ACM5957
Coccinellidae|[2042]|GMAGB132-15|Coleoptera|Coccinellidae||BOLD:ACW3435
Coccinellidae|[2043]|GMAGN357-15|Coleoptera|Coccinellidae||BOLD:ACN1917
Coccinellidae|[2044]|GMARM1359-14|Coleoptera|Coccinellidae||BOLD:ACN2521
Harmonia axyridis|[2045]|GMARF106-14|Coleoptera|Coccinellidae|Coccinellinae|BOLD:AAB5640
Coccinellidae|[2046]|GMARL1808-14|Coleoptera|Coccinellidae||BOLD:ABW3528
Coccinellidae|[2047]|GMAGC1311-15|Coleoptera|Coccinellidae||BOLD:ACV8710
Coccinellidae|[2048]|GMAGT1041-15|Coleoptera|Coccinellidae||BOLD:ACX6261
Coccinellidae|[2049]|GMAGA099-15|Coleoptera|Coccinellidae||BOLD:ACV3037
Coccinellidae|[2050]|GMAGD1665-15|Coleoptera|Coccinellidae||BOLD:ACW5469
Coccinellidae|[2051]|GMAGN359-15|Coleoptera|Coccinellidae||BOLD:ACX2431
Coccinellidae|[2052]|GMART1353-14|Coleoptera|Coccinellidae||BOLD:ACN9839
Corylophidae|[2053]|GMAGJ1330-15|Coleoptera|Corylophidae||BOLD:ACW8625
Corylophidae|[2054]|GMAGB119-15|Coleoptera|Corylophidae||BOLD:ACN1209
Corylophidae|[2055]|GMAGG041-15|Coleoptera|Corylophidae||BOLD:ACW3362
Corylophidae|[2056]|GMAGQ668-15|Coleoptera|Corylophidae||BOLD:ACX5017
Latridiidae|[2057]|GMAGL1511-15|Coleoptera|Latridiidae||BOLD:ACW8307
Corylophidae|[2058]|GMAGD1654-15|Coleoptera|Corylophidae||BOLD:ACW5209
Corylophidae|[2059]|GMAGB996-15|Coleoptera|Corylophidae||BOLD:ACW6285
Corylophidae|[2060]|GMARF1307-14|Coleoptera|Corylophidae||BOLD:ACN1349
Coccinellidae|[2061]|GMAGC1285-15|Coleoptera|Coccinellidae||BOLD:ACV9963
Coccinellidae|[2062]|GMAGC1273-15|Coleoptera|Coccinellidae||BOLD:ACV7535
Coccinellidae|[2063]|GMARV222-14|Coleoptera|Coccinellidae||BOLD:ACO0726
Corylophidae|[2064]|GMARK2440-14|Coleoptera|Corylophidae||BOLD:ACN0312
Latridiidae|[2065]|GMAGN382-15|Coleoptera|Latridiidae||BOLD:ACX2277
Latridiidae|[2066]|GMARA1743-14|Coleoptera|Latridiidae||BOLD:ACM5697
Latridiidae|[2067]|GMAGD1703-15|Coleoptera|Latridiidae||BOLD:ACM7244
Latridiidae|[2068]|GMAGD1681-15|Coleoptera|Latridiidae||BOLD:ACM7253
Latridiidae|[2069]|GMAGA096-15|Coleoptera|Latridiidae||BOLD:ACC9535
Latridiidae|[2070]|GMAGI1187-15|Coleoptera|Latridiidae||BOLD:ACO0163
Latridiidae|[2071]|GMARF1329-14|Coleoptera|Latridiidae||BOLD:ACN1749
Latridiidae|[2072]|GMARL1538-14|Coleoptera|Latridiidae||BOLD:ACN2154
Latridiidae|[2073]|GMAGA098-15|Coleoptera|Latridiidae||BOLD:ACN2423
Latridiidae|[2074]|GMAGP242-15|Coleoptera|Latridiidae||BOLD:ACX4986
Chrysomelidae|[2075]|GMAGS203-15|Coleoptera|Chrysomelidae||BOLD:ACX5750
Chrysomelidae|[2076]|GMARO1379-14|Coleoptera|Chrysomelidae||BOLD:ACO1194
Charidotis furunculus|[2077]|GMAGU134-15|Coleoptera|Chrysomelidae|Cassidinae|BOLD:ACX7311
Charidotis|[2078]|GMAGN036-15|Coleoptera|Chrysomelidae|Cassidinae|BOLD:ABW2327
Chrysomelidae|[2079]|GMARR926-14|Coleoptera|Chrysomelidae||BOLD:ACO0293
Chrysomelidae|[2080]|GMARV158-14|Coleoptera|Chrysomelidae||BOLD:ACO2297
Ptiliidae|[2081]|GMARE1287-14|Coleoptera|Ptiliidae||BOLD:ACM8125
Sciaridae|[2082]|GMARQ382-14|Diptera|Sciaridae||BOLD:ACN8732
Erotylidae|[2083]|GMAGD1707-15|Coleoptera|Erotylidae||BOLD:ACW4950
Erotylidae|[2084]|GMAGN323-15|Coleoptera|Erotylidae||BOLD:ACX2111
Curculionidae|[2085]|GMAGU685-15|Coleoptera|Curculionidae||BOLD:ACX5433
Sphindidae|[2086]|GMAGA771-15|Coleoptera|Sphindidae||BOLD:ACV3176
Staphylinidae|[2087]|GMAGF633-15|Coleoptera|Staphylinidae||BOLD:ACW4092
Staphylinidae|[2088]|GMARO1151-14|Coleoptera|Staphylinidae||BOLD:ACN8951
Acalymma albidovittata|[2089]|GMAGF637-15|Coleoptera|Chrysomelidae|Galerucinae|BOLD:ABW4702
Chrysomelidae|[2090]|GMARC958-14|Coleoptera|Chrysomelidae||BOLD:ACM6361
Chrysomelidae|[2091]|GMARM1549-14|Coleoptera|Chrysomelidae||BOLD:ACN3715

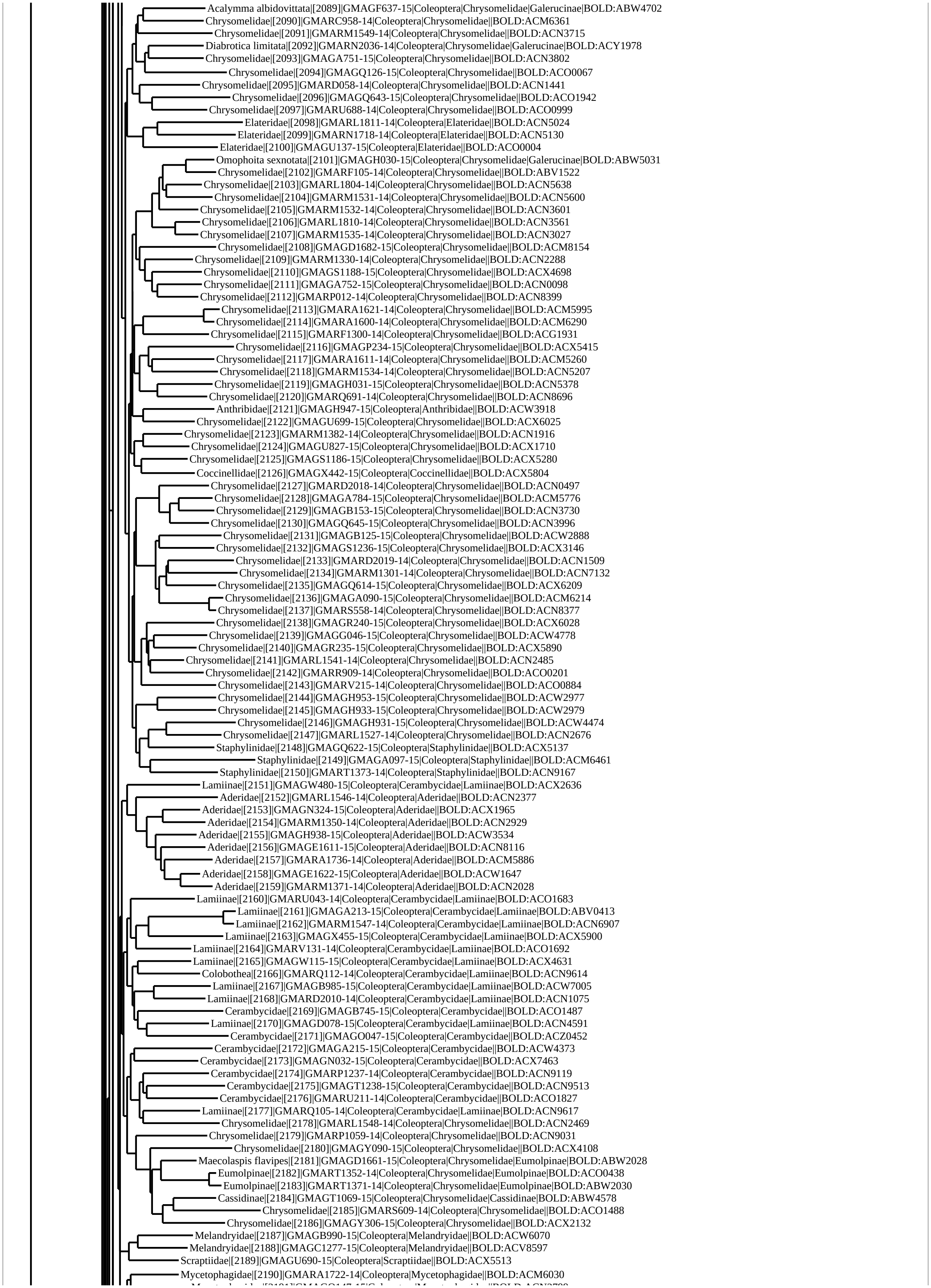
Acalymma albidovittata|[2089]|GMAGF637-15|Coleoptera|Chrysomelidae|Galerucinae|BOLD:ABW4702
Chrysomelidae|[2090]|GMARC958-14|Coleoptera|Chrysomelidae||BOLD:ACM6361
Chrysomelidae|[2091]|GMARM1549-14|Coleoptera|Chrysomelidae||BOLD:ACN3715
Diabrotica limitata|[2092]|GMARN2036-14|Coleoptera|Chrysomelidae|Galerucinae|BOLD:ACY1978
Chrysomelidae|[2093]|GMAGA751-15|Coleoptera|Chrysomelidae||BOLD:ACN3802
Chrysomelidae|[2094]|GMAGQ126-15|Coleoptera|Chrysomelidae||BOLD:ACO0067
Chrysomelidae|[2095]|GMARD058-14|Coleoptera|Chrysomelidae||BOLD:ACN1441
Chrysomelidae|[2096]|GMAGQ643-15|Coleoptera|Chrysomelidae||BOLD:ACO1942
Chrysomelidae|[2097]|GMARU688-14|Coleoptera|Chrysomelidae||BOLD:ACO0999
Elateridae|[2098]|GMARL1811-14|Coleoptera|Elateridae||BOLD:ACN5024
Elateridae|[2099]|GMARN1718-14|Coleoptera|Elateridae||BOLD:ACN5130
Elateridae|[2100]|GMAGU137-15|Coleoptera|Elateridae||BOLD:ACO0004
Omophoita sexnotata|[2101]|GMAGH030-15|Coleoptera|Chrysomelidae|Galerucinae|BOLD:ABW5031
Chrysomelidae|[2102]|GMARF105-14|Coleoptera|Chrysomelidae||BOLD:ABV1522
Chrysomelidae|[2103]|GMARL1804-14|Coleoptera|Chrysomelidae||BOLD:ACN5638
Chrysomelidae|[2104]|GMARM1531-14|Coleoptera|Chrysomelidae||BOLD:ACN5600
Chrysomelidae|[2105]|GMARM1532-14|Coleoptera|Chrysomelidae||BOLD:ACN3601
Chrysomelidae|[2106]|GMARL1810-14|Coleoptera|Chrysomelidae||BOLD:ACN3561
Chrysomelidae|[2107]|GMARM1535-14|Coleoptera|Chrysomelidae||BOLD:ACN3027
Chrysomelidae|[2108]|GMAGD1682-15|Coleoptera|Chrysomelidae||BOLD:ACM8154
Chrysomelidae|[2109]|GMARM1330-14|Coleoptera|Chrysomelidae||BOLD:ACN2288
Chrysomelidae|[2110]|GMAGS1188-15|Coleoptera|Chrysomelidae||BOLD:ACX4698
Chrysomelidae|[2111]|GMAGA752-15|Coleoptera|Chrysomelidae||BOLD:ACN0098
Chrysomelidae|[2112]|GMARP012-14|Coleoptera|Chrysomelidae||BOLD:ACN8399
Chrysomelidae|[2113]|GMARA1621-14|Coleoptera|Chrysomelidae||BOLD:ACM5995
Chrysomelidae|[2114]|GMARA1600-14|Coleoptera|Chrysomelidae||BOLD:ACM6290
Chrysomelidae|[2115]|GMARF1300-14|Coleoptera|Chrysomelidae||BOLD:ACG1931
Chrysomelidae|[2116]|GMAGP234-15|Coleoptera|Chrysomelidae||BOLD:ACX5415
Chrysomelidae|[2117]|GMARA1611-14|Coleoptera|Chrysomelidae||BOLD:ACM5260
Chrysomelidae|[2118]|GMARM1534-14|Coleoptera|Chrysomelidae||BOLD:ACN5207
Chrysomelidae|[2119]|GMAGH031-15|Coleoptera|Chrysomelidae||BOLD:ACN5378
Chrysomelidae|[2120]|GMARQ691-14|Coleoptera|Chrysomelidae||BOLD:ACN8696
Anthribidae|[2121]|GMAGH947-15|Coleoptera|Anthribidae||BOLD:ACW3918
Chrysomelidae|[2122]|GMAGU699-15|Coleoptera|Chrysomelidae||BOLD:ACX6025
Chrysomelidae|[2123]|GMARM1382-14|Coleoptera|Chrysomelidae||BOLD:ACN1916
Chrysomelidae|[2124]|GMAGU827-15|Coleoptera|Chrysomelidae||BOLD:ACX1710
Chrysomelidae|[2125]|GMAGS1186-15|Coleoptera|Chrysomelidae||BOLD:ACX5280
Coccinellidae|[2126]|GMAGX442-15|Coleoptera|Coccinellidae||BOLD:ACX5804
Chrysomelidae|[2127]|GMARD2018-14|Coleoptera|Chrysomelidae||BOLD:ACN0497
Chrysomelidae|[2128]|GMAGA784-15|Coleoptera|Chrysomelidae||BOLD:ACM5776
Chrysomelidae|[2129]|GMAGB153-15|Coleoptera|Chrysomelidae||BOLD:ACN3730
Chrysomelidae|[2130]|GMAGQ645-15|Coleoptera|Chrysomelidae||BOLD:ACN3996
Chrysomelidae|[2131]|GMAGB125-15|Coleoptera|Chrysomelidae||BOLD:ACW2888
Chrysomelidae|[2132]|GMAGS1236-15|Coleoptera|Chrysomelidae||BOLD:ACX3146
Chrysomelidae|[2133]|GMARD2019-14|Coleoptera|Chrysomelidae||BOLD:ACN1509
Chrysomelidae|[2134]|GMARM1301-14|Coleoptera|Chrysomelidae||BOLD:ACN7132
Chrysomelidae|[2135]|GMAGQ614-15|Coleoptera|Chrysomelidae||BOLD:ACX6209
Chrysomelidae|[2136]|GMAGA090-15|Coleoptera|Chrysomelidae||BOLD:ACM6214
Chrysomelidae|[2137]|GMARS558-14|Coleoptera|Chrysomelidae||BOLD:ACN8377
Chrysomelidae|[2138]|GMAGR240-15|Coleoptera|Chrysomelidae||BOLD:ACX6028
Chrysomelidae|[2139]|GMAGG046-15|Coleoptera|Chrysomelidae||BOLD:ACW4778
Chrysomelidae|[2140]|GMAGR235-15|Coleoptera|Chrysomelidae||BOLD:ACX5890
Chrysomelidae|[2141]|GMARL1541-14|Coleoptera|Chrysomelidae||BOLD:ACN2485
Chrysomelidae|[2142]|GMARR909-14|Coleoptera|Chrysomelidae||BOLD:ACO0201
Chrysomelidae|[2143]|GMARV215-14|Coleoptera|Chrysomelidae||BOLD:ACO0884
Chrysomelidae|[2144]|GMAGH953-15|Coleoptera|Chrysomelidae||BOLD:ACW2977
Chrysomelidae|[2145]|GMAGH933-15|Coleoptera|Chrysomelidae||BOLD:ACW2979
Chrysomelidae|[2146]|GMAGH931-15|Coleoptera|Chrysomelidae||BOLD:ACW4474
Chrysomelidae|[2147]|GMARL1527-14|Coleoptera|Chrysomelidae||BOLD:ACN2676
Staphylinidae|[2148]|GMAGQ622-15|Coleoptera|Staphylinidae||BOLD:ACX5137
Staphylinidae|[2149]|GMAGA097-15|Coleoptera|Staphylinidae||BOLD:ACM6461
Staphylinidae|[2150]|GMART1373-14|Coleoptera|Staphylinidae||BOLD:ACN9167
Lamiinae|[2151]|GMAGW480-15|Coleoptera|Cerambycidae|Lamiinae|BOLD:ACX2636
Aderidae|[2152]|GMARL1546-14|Coleoptera|Aderidae||BOLD:ACN2377
Aderidae|[2153]|GMAGN324-15|Coleoptera|Aderidae||BOLD:ACX1965
Aderidae|[2154]|GMARM1350-14|Coleoptera|Aderidae||BOLD:ACN2929
Aderidae|[2155]|GMAGH938-15|Coleoptera|Aderidae||BOLD:ACW3534
Aderidae|[2156]|GMAGE1611-15|Coleoptera|Aderidae||BOLD:ACN8116
Aderidae|[2157]|GMARA1736-14|Coleoptera|Aderidae||BOLD:ACM5886
Aderidae|[2158]|GMAGE1622-15|Coleoptera|Aderidae||BOLD:ACW1647
Aderidae|[2159]|GMARM1371-14|Coleoptera|Aderidae||BOLD:ACN2028
Lamiinae|[2160]|GMARU043-14|Coleoptera|Cerambycidae|Lamiinae|BOLD:ACO1683
Lamiinae|[2161]|GMAGA213-15|Coleoptera|Cerambycidae|Lamiinae|BOLD:ABV0413
Lamiinae|[2162]|GMARM1547-14|Coleoptera|Cerambycidae|Lamiinae|BOLD:ACN6907
Lamiinae|[2163]|GMAGX455-15|Coleoptera|Cerambycidae|Lamiinae|BOLD:ACX5900
Lamiinae|[2164]|GMARV131-14|Coleoptera|Cerambycidae|Lamiinae|BOLD:ACO1692
Lamiinae|[2165]|GMAGW115-15|Coleoptera|Cerambycidae|Lamiinae|BOLD:ACX4631
Colobothea|[2166]|GMARQ112-14|Coleoptera|Cerambycidae|Lamiinae|BOLD:ACN9614
Lamiinae|[2167]|GMAGB985-15|Coleoptera|Cerambycidae|Lamiinae|BOLD:ACW7005
Lamiinae|[2168]|GMARD2010-14|Coleoptera|Cerambycidae|Lamiinae|BOLD:ACN1075
Cerambycidae|[2169]|GMAGB745-15|Coleoptera|Cerambycidae||BOLD:ACO1487
Lamiinae|[2170]|GMAGD078-15|Coleoptera|Cerambycidae|Lamiinae|BOLD:ACN4591
Cerambycidae|[2171]|GMAGO047-15|Coleoptera|Cerambycidae||BOLD:ACZ0452
Cerambycidae|[2172]|GMAGA215-15|Coleoptera|Cerambycidae||BOLD:ACW4373
Cerambycidae|[2173]|GMAGN032-15|Coleoptera|Cerambycidae||BOLD:ACX7463
Cerambycidae|[2174]|GMARP1237-14|Coleoptera|Cerambycidae||BOLD:ACN9119
Cerambycidae|[2175]|GMAGT1238-15|Coleoptera|Cerambycidae||BOLD:ACN9513
Cerambycidae|[2176]|GMARU211-14|Coleoptera|Cerambycidae||BOLD:ACO1827
Lamiinae|[2177]|GMARQ105-14|Coleoptera|Cerambycidae|Lamiinae|BOLD:ACN9617
Chrysomelidae|[2178]|GMARL1548-14|Coleoptera|Chrysomelidae||BOLD:ACN2469
Chrysomelidae|[2179]|GMARP1059-14|Coleoptera|Chrysomelidae||BOLD:ACN9031
Chrysomelidae|[2180]|GMAGY090-15|Coleoptera|Chrysomelidae||BOLD:ACX4108
Maecolaspis flavipes|[2181]|GMAGD1661-15|Coleoptera|Chrysomelidae|Eumolpinae|BOLD:ABW2028
Eumolpinae|[2182]|GMART1352-14|Coleoptera|Chrysomelidae|Eumolpinae|BOLD:ACO0438
Eumolpinae|[2183]|GMART1371-14|Coleoptera|Chrysomelidae|Eumolpinae|BOLD:ABW2030
Cassidinae|[2184]|GMAGT1069-15|Coleoptera|Chrysomelidae|Cassidinae|BOLD:ABW4578
Chrysomelidae|[2185]|GMARS609-14|Coleoptera|Chrysomelidae||BOLD:ACO1488
Chrysomelidae|[2186]|GMAGY306-15|Coleoptera|Chrysomelidae||BOLD:ACX2132
Melandryidae|[2187]|GMAGB990-15|Coleoptera|Melandryidae||BOLD:ACW6070
Melandryidae|[2188]|GMAGC1277-15|Coleoptera|Melandryidae||BOLD:ACV8597
Scraptiidae|[2189]|GMAGU690-15|Coleoptera|Scraptiidae||BOLD:ACX5513
Mycetophagidae|[2190]|GMARA1722-14|Coleoptera|Mycetophagidae||BOLD:ACM6030

[illegible]
Scraptiidae|[2189]|GMAGU690-15|Coleoptera|Scraptiidae||BOLD:ACX5513
Mycetophagidae|[2190]|GMARA1722-14|Coleoptera|Mycetophagidae||BOLD:ACM6030
Mycetophagidae|[2191]|GMAGO147-15|Coleoptera|Mycetophagidae||BOLD:ACN2799
Coleoptera|[2192]|GMAGN350-15|Coleoptera|||BOLD:ACX3149
Coleoptera|[2193]|GMARW115-15|Coleoptera|||BOLD:ACX3921
Leiodidae|[2194]|GMARB059-14|Coleoptera|Leiodidae||BOLD:ACM5269
Leiodidae|[2195]|GMAGJ1356-15|Coleoptera|Leiodidae||BOLD:ACN7848
Leiodidae|[2196]|GMARM1338-14|Coleoptera|Leiodidae||BOLD:ACN2224
Scarabaeidae|[2197]|GMAGY589-15|Coleoptera|Scarabaeidae||BOLD:ACX6062
Leiodidae|[2198]|GMARK2400-14|Coleoptera|Leiodidae||BOLD:ACN0737
Leiodidae|[2199]|GMARN1703-14|Coleoptera|Leiodidae||BOLD:ACN5969
Leiodidae|[2200]|GMAGT1053-15|Coleoptera|Leiodidae||BOLD:ACX5909
Staphylinidae|[2201]|GMARI1338-14|Coleoptera|Staphylinidae||BOLD:ABW8690
Staphylinidae|[2202]|GMAGB1001-15|Coleoptera|Staphylinidae||BOLD:ACW6282
Staphylinidae|[2203]|GMARP1253-14|Coleoptera|Staphylinidae||BOLD:ACN9280
Staphylinidae|[2204]|GMARU797-14|Coleoptera|Staphylinidae||BOLD:ACO1856
Zelus pedestris|[2205]|GMARD1354-14|Hemiptera|Reduviidae|Harpactorinae|BOLD:ABV8946
Buprestidae|[2206]|GMART1701-14|Coleoptera|Buprestidae||BOLD:ACO2083
Eucnemidae|[2207]|GMARB072-14|Coleoptera|Eucnemidae||BOLD:ACM5575
Eucnemidae|[2208]|GMAGP230-15|Coleoptera|Eucnemidae||BOLD:ACX6134
Diptera|[2209]|GMAGG1343-15|Diptera|||BOLD:ACW3791
Staphylinidae|[2210]|GMAGG029-15|Coleoptera|Staphylinidae||BOLD:ACW4767
Staphylinidae|[2211]|GMARQ653-14|Coleoptera|Staphylinidae||BOLD:ACN8400
Staphylinidae|[2212]|GMARA1732-14|Coleoptera|Staphylinidae||BOLD:ACM5319
Staphylinidae|[2213]|GMAGG381-15|Coleoptera|Staphylinidae||BOLD:ACX6203
Staphylinidae|[2214]|GMARG1619-14|Coleoptera|Staphylinidae||BOLD:ACN1508
Staphylinidae|[2215]|GMARO1118-14|Coleoptera|Staphylinidae||BOLD:ACO3699
Staphylinidae|[2216]|GMAGE1630-15|Coleoptera|Staphylinidae||BOLD:ACM5527
Staphylinidae|[2217]|GMARB030-14|Coleoptera|Staphylinidae||BOLD:ACM9937
Staphylinidae|[2218]|GMAGG032-15|Coleoptera|Staphylinidae||BOLD:ACW3160
Staphylinidae|[2219]|GMAGR232-15|Coleoptera|Staphylinidae||BOLD:ACX5952
Staphylinidae|[2220]|GMAGD1660-15|Coleoptera|Staphylinidae||BOLD:ACW5100
Staphylinidae|[2221]|GMARP1183-14|Coleoptera|Staphylinidae||BOLD:ACN8902
Staphylinidae|[2222]|GMARP1232-14|Coleoptera|Staphylinidae||BOLD:ACN9754
Staphylinidae|[2223]|GMARS100-14|Coleoptera|Staphylinidae||BOLD:ACN9482
Staphylinidae|[2224]|GMARV213-14|Coleoptera|Staphylinidae||BOLD:ACO1187
Strangalia|[2225]|GMAGU127-15|Coleoptera|Cerambycidae|Lepturinae|BOLD:ACO1787
Staphylinidae|[2226]|GMAGV802-15|Coleoptera|Staphylinidae||BOLD:ACO0331
Staphylinidae|[2227]|GMAGG380-15|Coleoptera|Staphylinidae||BOLD:ACX4870
Staphylinidae|[2228]|GMAGD1708-15|Coleoptera|Staphylinidae||BOLD:ACW5208
Staphylinidae|[2229]|GMAGP244-15|Coleoptera|Staphylinidae||BOLD:ACX4196
Staphylinidae|[2230]|GMAGI1186-15|Coleoptera|Staphylinidae||BOLD:ACW6289
Staphylinidae|[2231]|GMAGT1033-15|Coleoptera|Staphylinidae||BOLD:ACX4964
Leiodidae|[2232]|GMAGN376-15|Coleoptera|Leiodidae||BOLD:ACB1685
Staphylinidae|[2233]|GMAGE1615-15|Coleoptera|Staphylinidae||BOLD:ACW2269
Staphylinidae|[2234]|GMAGN378-15|Coleoptera|Staphylinidae||BOLD:ACX1941
Staphylinidae|[2235]|GMARQ644-14|Coleoptera|Staphylinidae||BOLD:ACN7929
Staphylinidae|[2236]|GMARD2004-14|Coleoptera|Staphylinidae||BOLD:ACN0216
Staphylinidae|[2237]|GMAGQ654-15|Coleoptera|Staphylinidae||BOLD:ACX6161
Staphylinidae|[2238]|GMAGE1600-15|Coleoptera|Staphylinidae||BOLD:ACW1439
Staphylinidae|[2239]|GMARN1716-14|Coleoptera|Staphylinidae||BOLD:ACN4285
Staphylinidae|[2240]|GMARN1694-14|Coleoptera|Staphylinidae||BOLD:ACN4592
Staphylinidae|[2241]|GMARN1641-14|Coleoptera|Staphylinidae||BOLD:ACO0972
Staphylinidae|[2242]|GMARO1403-14|Coleoptera|Staphylinidae||BOLD:ABA0931
Ptilodactylidae|[2243]|GMARB135-14|Coleoptera|Ptilodactylidae||BOLD:ACN0507
Ptilodactylidae|[2244]|GMAGC1256-15|Coleoptera|Ptilodactylidae||BOLD:ACN9601
Ptilodactylidae|[2245]|GMARA988-14|Coleoptera|Ptilodactylidae||BOLD:ACN1710
Ptilodactylidae|[2246]|GMAGD081-15|Coleoptera|Ptilodactylidae||BOLD:ACN1812
Ptilodactylidae|[2247]|GMAGI016-15|Coleoptera|Ptilodactylidae||BOLD:ACN6580
Ptilodactylidae|[2248]|GMAGS1269-15|Coleoptera|Ptilodactylidae||BOLD:ACN9454
Ptilodactylidae|[2249]|GMARA1745-14|Coleoptera|Ptilodactylidae||BOLD:ACM5823
Ptilodactylidae|[2250]|GMARP1256-14|Coleoptera|Ptilodactylidae||BOLD:ACO0417
Ptilodactylidae|[2251]|GMAGA749-15|Coleoptera|Ptilodactylidae||BOLD:ACM5841
Ptilodactylidae|[2252]|GMARP935-14|Coleoptera|Ptilodactylidae||BOLD:ACO2109
Ptilodactylidae|[2253]|GMARP955-14|Coleoptera|Ptilodactylidae||BOLD:ACN8549
Ptilodactylidae|[2254]|GMAGA755-15|Coleoptera|Ptilodactylidae||BOLD:ACV1985
Ptilodactylidae|[2255]|GMARP031-14|Coleoptera|Ptilodactylidae||BOLD:ACN8422
Ptilodactylidae|[2256]|GMAGD1664-15|Coleoptera|Ptilodactylidae||BOLD:ACW4976
Ptilodactylidae|[2257]|GMARR925-14|Coleoptera|Ptilodactylidae||BOLD:ACN9138
Ptilodactylidae|[2258]|GMARL1813-14|Coleoptera|Ptilodactylidae||BOLD:ACN3755
Ptilodactylidae|[2259]|GMAGQ134-15|Coleoptera|Ptilodactylidae||BOLD:ACN9429
Ptilodactylidae|[2260]|GMAGS196-15|Coleoptera|Ptilodactylidae||BOLD:ACX6884
Staphylinidae|[2261]|GMAGW481-15|Coleoptera|Staphylinidae||BOLD:ACX2040
Hydrophilidae|[2262]|GMAGY308-15|Coleoptera|Hydrophilidae||BOLD:ACX3284
Megalopodidae|[2263]|GMARM1544-14|Coleoptera|Megalopodidae||BOLD:ACN4552
Staphylinidae|[2264]|GMAGH934-15|Coleoptera|Staphylinidae||BOLD:ACW3916
Staphylinidae|[2265]|GMAGQ691-15|Coleoptera|Staphylinidae||BOLD:ACX5783
Staphylinidae|[2266]|GMAGQ010-15|Coleoptera|Staphylinidae||BOLD:ACX6605
Monotomidae|[2267]|GMARL1522-14|Coleoptera|Monotomidae||BOLD:ACN2462
Monotomidae|[2268]|GMAGU653-15|Coleoptera|Monotomidae||BOLD:ACX5961
Nitidulidae|[2269]|GMAGT1067-15|Coleoptera|Nitidulidae||BOLD:ACX4091
Mycetophagidae|[2270]|GMARA1614-14|Coleoptera|Mycetophagidae||BOLD:ACM5321
Staphylinidae|[2271]|GMARM1548-14|Coleoptera|Staphylinidae||BOLD:ACN5407
Cleridae|[2272]|GMAGA750-15|Coleoptera|Cleridae||BOLD:ACN0664
Coleoptera|[2273]|GMARO1121-14|Coleoptera|||BOLD:ACO3819
Coleoptera|[2274]|GMAGT1077-15|Coleoptera|||BOLD:ACX5861
Diptera|[2275]|GMAGP395-15|Diptera|||BOLD:ACX4119
Stigmella schinivora|[2276]|GMARF1159-14|Lepidoptera|Nepticulidae||BOLD:ACN0764
Nepticulidae|[2277]|GMAGA108-15|Lepidoptera|Nepticulidae||BOLD:ACM6469
Stigmella|[2278]|GMAGB978-15|Lepidoptera|Nepticulidae||BOLD:ACM9954
Nepticulidae|[2279]|GMAGA107-15|Lepidoptera|Nepticulidae||BOLD:ACN0952
Nepticulidae|[2280]|GMAGF602-15|Lepidoptera|Nepticulidae||BOLD:ACW3559
Nepticulidae|[2281]|GMAGM987-15|Lepidoptera|Nepticulidae||BOLD:ACY1136
Nepticulidae|[2282]|GMARL1641-14|Lepidoptera|Nepticulidae||BOLD:ACN2400
Nepticulidae|[2283]|GMAGN464-15|Lepidoptera|Nepticulidae||BOLD:ACX4482
Nepticulidae|[2284]|GMARF1145-14|Lepidoptera|Nepticulidae||BOLD:ACN0263
Nepticulidae|[2285]|GMAGO170-15|Lepidoptera|Nepticulidae||BOLD:ACX5285
Nepticulidae|[2286]|GMAGP103-15|Lepidoptera|Nepticulidae||BOLD:ACX1863
Schaefferiana|[2287]|GMAGU008-15|Lepidoptera|Hepialidae||BOLD:ACX4213
Cibyra meridionalis|[2288]|GMAGN012-15|Lepidoptera|Hepialidae||BOLD:ACX6932
Cibyra|[2289]|GMART021-14|Lepidoptera|Hepialidae||BOLD:ACO1910
Opostegidae|[2290]|GMARQ688-14|Lepidoptera|Opostegidae||BOLD:ACN7416

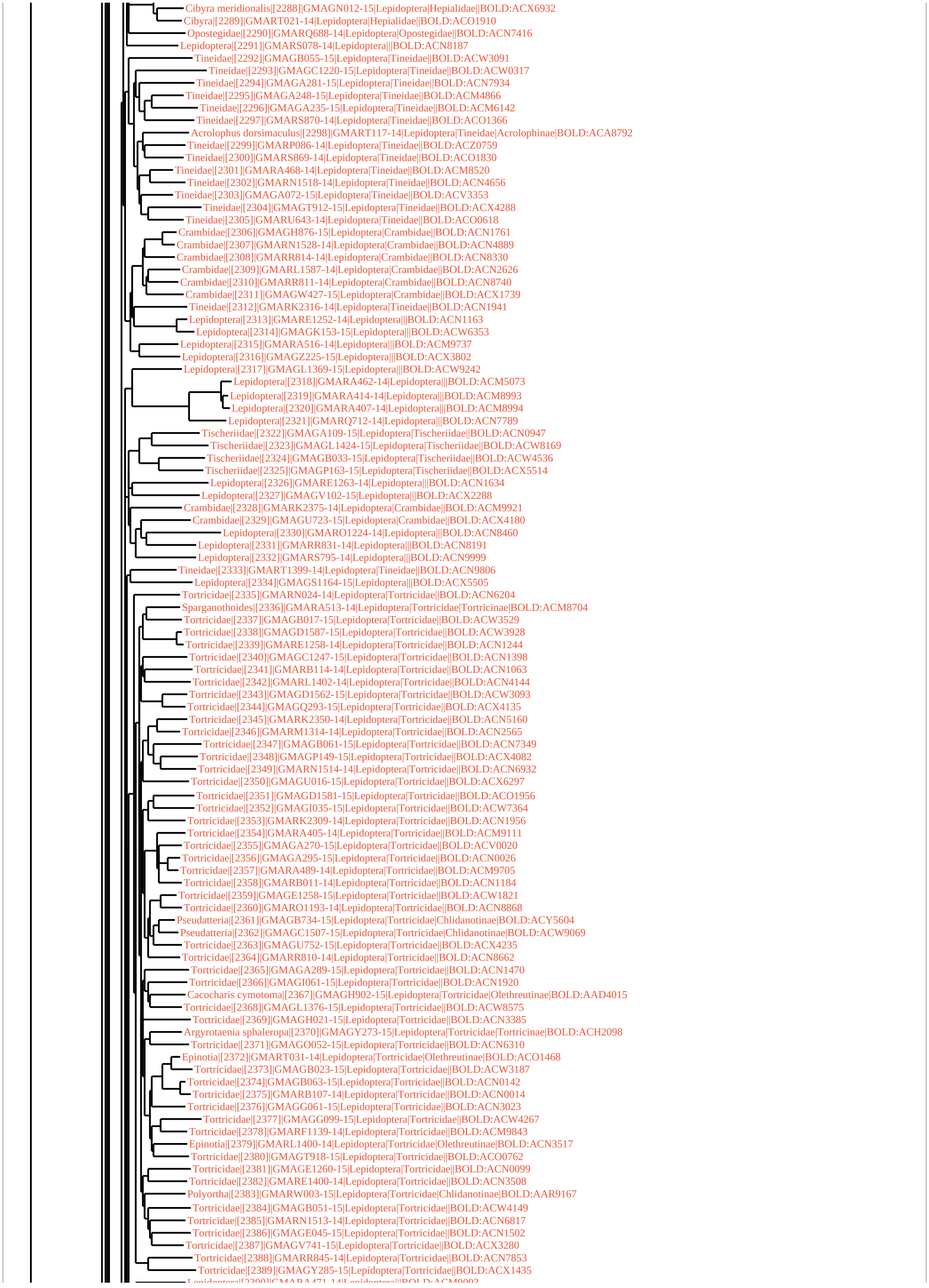
Cibyra meridionalis|[2288]|GMAGN012-15|Lepidoptera|Hepialidae||BOLD:ACX6932
Cibyra|[2289]|GMART021-14|Lepidoptera|Hepialidae||BOLD:ACO1910
Opostegidae|[2290]|GMARQ688-14|Lepidoptera|Opostegidae||BOLD:ACN7416
Lepidoptera|[2291]|GMARS078-14|Lepidoptera|||BOLD:ACN8187
Tineidae|[2292]|GMAGB055-15|Lepidoptera|Tineidae||BOLD:ACW3091
Tineidae|[2293]|GMAGC1220-15|Lepidoptera|Tineidae||BOLD:ACW0317
Tineidae|[2294]|GMAGA281-15|Lepidoptera|Tineidae||BOLD:ACN7934
Tineidae|[2295]|GMAGA248-15|Lepidoptera|Tineidae||BOLD:ACM4866
Tineidae|[2296]|GMAGA235-15|Lepidoptera|Tineidae||BOLD:ACM6142
Tineidae|[2297]|GMARS870-14|Lepidoptera|Tineidae||BOLD:ACO1366
Acrolophus dorsimaculus|[2298]|GMART117-14|Lepidoptera|Tineidae|Acrolophinae|BOLD:ACA8792
Tineidae|[2299]|GMARP086-14|Lepidoptera|Tineidae||BOLD:ACZ0759
Tineidae|[2300]|GMARS869-14|Lepidoptera|Tineidae||BOLD:ACO1830
Tineidae|[2301]|GMARA468-14|Lepidoptera|Tineidae||BOLD:ACM8520
Tineidae|[2302]|GMARN1518-14|Lepidoptera|Tineidae||BOLD:ACN4656
Tineidae|[2303]|GMAGA072-15|Lepidoptera|Tineidae||BOLD:ACV3353
Tineidae|[2304]|GMAGT912-15|Lepidoptera|Tineidae||BOLD:ACX4288
Tineidae|[2305]|GMARU643-14|Lepidoptera|Tineidae||BOLD:ACO0618
Crambidae|[2306]|GMAGH876-15|Lepidoptera|Crambidae||BOLD:ACN1761
Crambidae|[2307]|GMARN1528-14|Lepidoptera|Crambidae||BOLD:ACN4889
Crambidae|[2308]|GMARR814-14|Lepidoptera|Crambidae||BOLD:ACN8330
Crambidae|[2309]|GMARL1587-14|Lepidoptera|Crambidae||BOLD:ACN2626
Crambidae|[2310]|GMARR811-14|Lepidoptera|Crambidae||BOLD:ACN8740
Crambidae|[2311]|GMAGW427-15|Lepidoptera|Crambidae||BOLD:ACX1739
Tineidae|[2312]|GMARK2316-14|Lepidoptera|Tineidae||BOLD:ACN1941
Lepidoptera|[2313]|GMARE1252-14|Lepidoptera|||BOLD:ACN1163
Lepidoptera|[2314]|GMAGK153-15|Lepidoptera|||BOLD:ACW6353
Lepidoptera|[2315]|GMARA516-14|Lepidoptera|||BOLD:ACM9737
Lepidoptera|[2316]|GMAGZ225-15|Lepidoptera|||BOLD:ACX3802
Lepidoptera|[2317]|GMAGL1369-15|Lepidoptera|||BOLD:ACW9242
Lepidoptera|[2318]|GMARA462-14|Lepidoptera|||BOLD:ACM5073
Lepidoptera|[2319]|GMARA414-14|Lepidoptera|||BOLD:ACM8993
Lepidoptera|[2320]|GMARA407-14|Lepidoptera|||BOLD:ACM8994
Lepidoptera|[2321]|GMARQ712-14|Lepidoptera|||BOLD:ACN7789
Tischeriidae|[2322]|GMAGA109-15|Lepidoptera|Tischeriidae||BOLD:ACN0947
Tischeriidae|[2323]|GMAGL1424-15|Lepidoptera|Tischeriidae||BOLD:ACW8169
Tischeriidae|[2324]|GMAGB033-15|Lepidoptera|Tischeriidae||BOLD:ACW4536
Tischeriidae|[2325]|GMAGP163-15|Lepidoptera|Tischeriidae||BOLD:ACX5514
Lepidoptera|[2326]|GMARE1263-14|Lepidoptera|||BOLD:ACN1634
Lepidoptera|[2327]|GMAGV102-15|Lepidoptera|||BOLD:ACX2288
Crambidae|[2328]|GMARK2375-14|Lepidoptera|Crambidae||BOLD:ACM9921
Crambidae|[2329]|GMAGU723-15|Lepidoptera|Crambidae||BOLD:ACX4180
Lepidoptera|[2330]|GMARO1224-14|Lepidoptera|||BOLD:ACN8460
Lepidoptera|[2331]|GMARR831-14|Lepidoptera|||BOLD:ACN8191
Lepidoptera|[2332]|GMARS795-14|Lepidoptera|||BOLD:ACN9999
Tineidae|[2333]|GMART1399-14|Lepidoptera|Tineidae||BOLD:ACN9806
Lepidoptera|[2334]|GMAGS1164-15|Lepidoptera|||BOLD:ACX5505
Tortricidae|[2335]|GMARN024-14|Lepidoptera|Tortricidae||BOLD:ACN6204
Sparganothoides|[2336]|GMARA513-14|Lepidoptera|Tortricidae|Tortricinae|BOLD:ACM8704
Tortricidae|[2337]|GMAGB017-15|Lepidoptera|Tortricidae||BOLD:ACW3529
Tortricidae|[2338]|GMAGD1587-15|Lepidoptera|Tortricidae||BOLD:ACW3928
Tortricidae|[2339]|GMARE1258-14|Lepidoptera|Tortricidae||BOLD:ACN1244
Tortricidae|[2340]|GMAGC1247-15|Lepidoptera|Tortricidae||BOLD:ACN1398
Tortricidae|[2341]|GMARB114-14|Lepidoptera|Tortricidae||BOLD:ACN1063
Tortricidae|[2342]|GMARL1402-14|Lepidoptera|Tortricidae||BOLD:ACN4144
Tortricidae|[2343]|GMAGD1562-15|Lepidoptera|Tortricidae||BOLD:ACW3093
Tortricidae|[2344]|GMAGQ293-15|Lepidoptera|Tortricidae||BOLD:ACX4135
Tortricidae|[2345]|GMARK2350-14|Lepidoptera|Tortricidae||BOLD:ACN5160
Tortricidae|[2346]|GMARM1314-14|Lepidoptera|Tortricidae||BOLD:ACN2565
Tortricidae|[2347]|GMAGB061-15|Lepidoptera|Tortricidae||BOLD:ACN7349
Tortricidae|[2348]|GMAGP149-15|Lepidoptera|Tortricidae||BOLD:ACX4082
Tortricidae|[2349]|GMARN1514-14|Lepidoptera|Tortricidae||BOLD:ACN6932
Tortricidae|[2350]|GMAGU016-15|Lepidoptera|Tortricidae||BOLD:ACX6297
Tortricidae|[2351]|GMAGD1581-15|Lepidoptera|Tortricidae||BOLD:ACO1956
Tortricidae|[2352]|GMAGI035-15|Lepidoptera|Tortricidae||BOLD:ACW7364
Tortricidae|[2353]|GMARK2309-14|Lepidoptera|Tortricidae||BOLD:ACN1956
Tortricidae|[2354]|GMARA405-14|Lepidoptera|Tortricidae||BOLD:ACM9111
Tortricidae|[2355]|GMAGA270-15|Lepidoptera|Tortricidae||BOLD:ACV0020
Tortricidae|[2356]|GMAGA295-15|Lepidoptera|Tortricidae||BOLD:ACN0026
Tortricidae|[2357]|GMARA489-14|Lepidoptera|Tortricidae||BOLD:ACM9705
Tortricidae|[2358]|GMARB011-14|Lepidoptera|Tortricidae||BOLD:ACN1184
Tortricidae|[2359]|GMAGE1258-15|Lepidoptera|Tortricidae||BOLD:ACW1821
Tortricidae|[2360]|GMARO1193-14|Lepidoptera|Tortricidae||BOLD:ACN8868
Pseudatteria|[2361]|GMAGB734-15|Lepidoptera|Tortricidae|Chlidanotinae|BOLD:ACY5604
Pseudatteria|[2362]|GMAGC1507-15|Lepidoptera|Tortricidae|Chlidanotinae|BOLD:ACW9069
Tortricidae|[2363]|GMAGU752-15|Lepidoptera|Tortricidae||BOLD:ACX4235
Tortricidae|[2364]|GMARR810-14|Lepidoptera|Tortricidae||BOLD:ACN8662
Tortricidae|[2365]|GMAGA289-15|Lepidoptera|Tortricidae||BOLD:ACN1470
Tortricidae|[2366]|GMAGI061-15|Lepidoptera|Tortricidae||BOLD:ACN1920
Cacocharis cymotoma|[2367]|GMAGH902-15|Lepidoptera|Tortricidae|Olethreutinae|BOLD:AAD4015
Tortricidae|[2368]|GMAGL1376-15|Lepidoptera|Tortricidae||BOLD:ACW8575
Tortricidae|[2369]|GMAGH021-15|Lepidoptera|Tortricidae||BOLD:ACN3385
Argyrotaenia sphaleropa|[2370]|GMAGY273-15|Lepidoptera|Tortricidae|Tortricinae|BOLD:ACH2098
Tortricidae|[2371]|GMAGO052-15|Lepidoptera|Tortricidae||BOLD:ACN6310
Epinotia|[2372]|GMART031-14|Lepidoptera|Tortricidae|Olethreutinae|BOLD:ACO1468
Tortricidae|[2373]|GMAGB023-15|Lepidoptera|Tortricidae||BOLD:ACW3187
Tortricidae|[2374]|GMAGB063-15|Lepidoptera|Tortricidae||BOLD:ACN0142
Tortricidae|[2375]|GMARB107-14|Lepidoptera|Tortricidae||BOLD:ACN0014
Tortricidae|[2376]|GMAGG061-15|Lepidoptera|Tortricidae||BOLD:ACN3023
Tortricidae|[2377]|GMAGG099-15|Lepidoptera|Tortricidae||BOLD:ACW4267
Tortricidae|[2378]|GMARF1139-14|Lepidoptera|Tortricidae||BOLD:ACM9843
Epinotia|[2379]|GMARL1400-14|Lepidoptera|Tortricidae|Olethreutinae|BOLD:ACN3517
Tortricidae|[2380]|GMAGT918-15|Lepidoptera|Tortricidae||BOLD:ACO0762
Tortricidae|[2381]|GMAGE1260-15|Lepidoptera|Tortricidae||BOLD:ACN0099
Tortricidae|[2382]|GMARE1400-14|Lepidoptera|Tortricidae||BOLD:ACN3508
Polyortha|[2383]|GMARW003-15|Lepidoptera|Tortricidae|Chlidanotinae|BOLD:AAR9167
Tortricidae|[2384]|GMAGB051-15|Lepidoptera|Tortricidae||BOLD:ACW4149
Tortricidae|[2385]|GMARN1513-14|Lepidoptera|Tortricidae||BOLD:ACN6817
Tortricidae|[2386]|GMAGE045-15|Lepidoptera|Tortricidae||BOLD:ACN1502
Tortricidae|[2387]|GMAGV741-15|Lepidoptera|Tortricidae||BOLD:ACX3280
Tortricidae|[2388]|GMARR845-14|Lepidoptera|Tortricidae||BOLD:ACN7853
Tortricidae|[2389]|GMAGY285-15|Lepidoptera|Tortricidae||BOLD:ACX1435
Lepidoptera|[2390]|GMARA471-14|Lepidoptera|||BOLD:ACM[illegible]

[illegible]
Tortricidae|[2388]|GMARR845-14|Lepidoptera|Tortricidae||BOLD:ACN7853
Tortricidae|[2389]|GMAGY285-15|Lepidoptera|Tortricidae||BOLD:ACX1435
Lepidoptera|[2390]|GMARA471-14|Lepidoptera|||BOLD:ACM9093
Noctuidae|[2391]|GMARA417-14|Lepidoptera|Noctuidae||BOLD:ACM9217
Gelechiidae|[2392]|GMAGB053-15|Lepidoptera|Gelechiidae||BOLD:ACY5761
Lepidoptera|[2393]|GMAGQ560-15|Lepidoptera|||BOLD:ACX4927
Atteva pustulella|[2394]|GMAGR019-15|Lepidoptera|Attevidae|Attevinae|BOLD:AAA9259
Phycitinae|[2395]|GMAGP114-15|Lepidoptera|Pyralidae|Phycitinae|BOLD:AAN7208
Crambidae|[2396]|GMAGP164-15|Lepidoptera|Crambidae||BOLD:ACX4486
Crambidae|[2397]|GMARP998-14|Lepidoptera|Crambidae||BOLD:ACO1418
Crambidae|[2398]|GMAGT909-15|Lepidoptera|Crambidae||BOLD:ACX4233
Crambidae|[2399]|GMAGT938-15|Lepidoptera|Crambidae||BOLD:ACX6040
Lepidoptera|[2400]|GMAGI063-15|Lepidoptera|||BOLD:ACN2520
Lepidoptera|[2401]|GMAGT926-15|Lepidoptera|||BOLD:ACX5197
Epermeniidae|[2402]|GMAGD1594-15|Lepidoptera|Epermeniidae||BOLD:ACN1783
Momphidae|[2403]|GMARB381-14|Lepidoptera|Momphidae||BOLD:ACM6144
Momphidae|[2404]|GMAGP157-15|Lepidoptera|Momphidae||BOLD:ACX5624
Lepidoptera|[2405]|GMAGT906-15|Lepidoptera|||BOLD:ACN8972
Lepidoptera|[2406]|GMAGL1386-15|Lepidoptera|||BOLD:ACW9087
Lepidoptera|[2407]|GMAGW445-15|Lepidoptera|||BOLD:ACX2499
Glyphipterigidae|[2408]|GMARA419-14|Lepidoptera|Glyphipterigidae||BOLD:ACX3483
Glyphipterigidae|[2409]|GMAGE1222-15|Lepidoptera|Glyphipterigidae||BOLD:ACN2848
Tineidae|[2410]|GMARA1924-14|Lepidoptera|Tineidae||BOLD:ACM9926
Crambidae|[2411]|GMAGH911-15|Lepidoptera|Crambidae||BOLD:ACW2982
Crambidae|[2412]|GMAGH884-15|Lepidoptera|Crambidae||BOLD:ACN0836
Zygaenidae|[2413]|GMAGV089-15|Lepidoptera|Zygaenidae||BOLD:ACX5962
Lepidoptera|[2414]|GMARM1321-14|Lepidoptera|||BOLD:ACN2016
Glyphipterigidae|[2415]|GMAGB025-15|Lepidoptera|Glyphipterigidae||BOLD:ACM5912
Lepidoptera|[2416]|GMAGD1554-15|Lepidoptera|||BOLD:ACO0777
Gelechiidae|[2417]|GMAGC1254-15|Lepidoptera|Gelechiidae||BOLD:ACV7867
Gelechiidae|[2418]|GMARK2348-14|Lepidoptera|Gelechiidae||BOLD:ACN3707
Lepidoptera|[2419]|GMAGC1238-15|Lepidoptera|||BOLD:ACN0494
Lepidoptera|[2420]|GMARR012-14|Lepidoptera|||BOLD:ACN9207
Bucculatricidae|[2421]|GMAGA106-15|Lepidoptera|Bucculatricidae||BOLD:ACM5911
Bucculatricidae|[2422]|GMAGA850-15|Lepidoptera|Bucculatricidae||BOLD:ACM6084
Bucculatricidae|[2423]|GMAGN440-15|Lepidoptera|Bucculatricidae||BOLD:ACX4487
Bucculatricidae|[2424]|GMAGA101-15|Lepidoptera|Bucculatricidae||BOLD:ACM5074
Bucculatricidae|[2425]|GMAGQ353-15|Lepidoptera|Bucculatricidae||BOLD:ACX2547
Choreutidae|[2426]|GMAGB048-15|Lepidoptera|Choreutidae||BOLD:ACN0599
Tebenna|[2427]|GMARR835-14|Lepidoptera|Choreutidae|Choreutinae|BOLD:ACX2229
Choreutidae|[2428]|GMAGP142-15|Lepidoptera|Choreutidae||BOLD:ACX5515
Episcea extravagans|[2429]|GMARE1393-14|Lepidoptera|Erebidae|Arctiinae|BOLD:AAZ3869
Lepidoptera|[2430]|GMARA450-14|Lepidoptera|||BOLD:ACM8477
Lepidoptera|[2431]|GMARS049-14|Lepidoptera|||BOLD:ACN9585
Heliodinidae|[2432]|GMAGN417-15|Lepidoptera|Heliodinidae||BOLD:ACX4454
Heliodinidae|[2433]|GMAGD1577-15|Lepidoptera|Heliodinidae||BOLD:ACW4735
Heliodinidae|[2434]|GMAGE1264-15|Lepidoptera|Heliodinidae||BOLD:ACM5551
Heliodinidae|[2435]|GMARP997-14|Lepidoptera|Heliodinidae||BOLD:ACO1719
Geometridae|[2436]|GMAGD1570-15|Lepidoptera|Geometridae||BOLD:ACN2646
Scythrididae|[2437]|GMARA446-14|Lepidoptera|Scythrididae||BOLD:ACM5552
Lepidoptera|[2438]|GMAGE1269-15|Lepidoptera|||BOLD:ACM5550
Lepidoptera|[2439]|GMARL1399-14|Lepidoptera|||BOLD:ACN3269
Semaeopus|[2440]|GMAGL1170-15|Lepidoptera|Geometridae|Sterrhinae|BOLD:AAD4215
Geometridae|[2441]|GMAGL1171-15|Lepidoptera|Geometridae||BOLD:ACB9650
Geometridae|[2442]|GMARW148-15|Lepidoptera|Geometridae||BOLD:ACX3398
Semaeopus|[2443]|GMAGA110-15|Lepidoptera|Geometridae|Sterrhinae|BOLD:AAJ7273
Geometridae|[2444]|GMAGC1226-15|Lepidoptera|Geometridae||BOLD:ACV8097
Lepidoptera|[2445]|GMAGA244-15|Lepidoptera|||BOLD:ACM9432
Lepidoptera|[2446]|GMART034-14|Lepidoptera|||BOLD:ACO1798
Gelechiidae|[2447]|GMAGA225-15|Lepidoptera|Gelechiidae||BOLD:ACM6143
Gelechiidae|[2448]|GMAGA300-15|Lepidoptera|Gelechiidae||BOLD:ACM6261
Gelechiidae|[2449]|GMAGE1226-15|Lepidoptera|Gelechiidae||BOLD:ACN6094
Gelechiidae|[2450]|GMAGB058-15|Lepidoptera|Gelechiidae||BOLD:AAM8734
Gelechiidae|[2451]|GMARK2295-14|Lepidoptera|Gelechiidae||BOLD:ACX2807
Gelechiidae|[2452]|GMARL1600-14|Lepidoptera|Gelechiidae||BOLD:ACN2038
Faculta|[2453]|GMAGQ517-15|Lepidoptera|Gelechiidae|Gelechiinae|BOLD:ACR1565
Lepidoptera|[2454]|GMARN014-14|Lepidoptera|||BOLD:ACN6840
Gelechiidae|[2455]|GMAGA221-15|Lepidoptera|Gelechiidae||BOLD:ACV1866
Lepidoptera|[2456]|GMAGT927-15|Lepidoptera|||BOLD:ACX5596
Lepidoptera|[2457]|GMAGY276-15|Lepidoptera|||BOLD:ACX1501
Elachistidae|[2458]|GMAGA102-15|Lepidoptera|Elachistidae||BOLD:ACM6483
Elachistidae|[2459]|GMAGD1601-15|Lepidoptera|Elachistidae||BOLD:ACN0380
Elachistidae|[2460]|GMAGB028-15|Lepidoptera|Elachistidae||BOLD:ACW4515
Elachistidae|[2461]|GMAGP133-15|Lepidoptera|Elachistidae||BOLD:ACX5957
Noctuidae|[2462]|GMARA427-14|Lepidoptera|Noctuidae||BOLD:ACM9156
Bucculatricidae|[2463]|GMARD2032-14|Lepidoptera|Bucculatricidae||BOLD:ACN1134
Lepidoptera|[2464]|GMARV146-14|Lepidoptera|||BOLD:ACO2110
Lepidoptera|[2465]|GMAGK2010-15|Lepidoptera|||BOLD:ACX0074
Gelechiidae|[2466]|GMARK2276-14|Lepidoptera|Gelechiidae||BOLD:ACN2761
Gelechiidae|[2467]|GMARM1318-14|Lepidoptera|Gelechiidae||BOLD:ACN2354
Crambidae|[2468]|GMARA971-14|Lepidoptera|Crambidae||BOLD:ACM9955
Crambidae|[2469]|GMAGX403-15|Lepidoptera|Crambidae||BOLD:ACX4543
Crambidae|[2470]|GMARL1591-14|Lepidoptera|Crambidae||BOLD:ACN2088
Lepidoptera|[2471]|GMARI1558-14|Lepidoptera|||BOLD:ACM9876
Crambidae|[2472]|GMARQ720-14|Lepidoptera|Crambidae||BOLD:ACN7524
Lepidoptera|[2473]|GMARK2353-14|Lepidoptera|||BOLD:ACN6866
Gelechiidae|[2474]|GMAGB059-15|Lepidoptera|Gelechiidae||BOLD:ACW4064
Gelechiidae|[2475]|GMAGG095-15|Lepidoptera|Gelechiidae||BOLD:ACN1665
Gelechiidae|[2476]|GMAGK151-15|Lepidoptera|Gelechiidae||BOLD:ACN8745
Gelechiidae|[2477]|GMAGD1574-15|Lepidoptera|Gelechiidae||BOLD:ACO0702
Gelechiidae|[2478]|GMAGQ518-15|Lepidoptera|Gelechiidae||BOLD:ACX4716
Ephestiodes|[2479]|GMARS046-14|Lepidoptera|Pyralidae|Phycitinae|BOLD:ACO0323
Pyralidae|[2480]|GMAGO064-15|Lepidoptera|Pyralidae||BOLD:ACX4786
Pyralidae|[2481]|GMARN010-14|Lepidoptera|Pyralidae||BOLD:ACN6426
Pyralidae|[2482]|GMARP989-14|Lepidoptera|Pyralidae||BOLD:ACO1760
Crambidae|[2483]|GMAGF580-15|Lepidoptera|Crambidae||BOLD:ACM5224
Lepidoptera|[2484]|GMART1394-14|Lepidoptera|||BOLD:ACO0339
Crambidae|[2485]|GMARS794-14|Lepidoptera|Crambidae||BOLD:ACN9853
Crambidae|[2486]|GMAGX146-15|Lepidoptera|Crambidae||BOLD:ACX2042
Lepidoptera|[2487]|GMAGB038-15|Lepidoptera|||BOLD:ACW3256
Lepidoptera|[2488]|GMARA424-14|Lepidoptera|||BOLD:ACM4864
Lepidoptera|[2489]|GMARB375-14|Lepidoptera|||BOLD:ACM6145

Lepidoptera|[2487]|GMAGB038-15|Lepidoptera|||BOLD:ACW3256
Lepidoptera|[2488]|GMARA424-14|Lepidoptera|||BOLD:ACM4864
Lepidoptera|[2489]|GMARB375-14|Lepidoptera|||BOLD:ACM6145
Crambidae|[2490]|GMARD2033-14|Lepidoptera|Crambidae||BOLD:ACN1505
Lepidoptera|[2491]|GMAGB046-15|Lepidoptera|||BOLD:ACN7649
Lepidoptera|[2492]|GMAGJ1426-15|Lepidoptera|||BOLD:ACW9042
Lepidoptera|[2493]|GMARN1499-14|Lepidoptera|||BOLD:ACN3860
Lepidoptera|[2494]|GMARP1028-14|Lepidoptera|||BOLD:ACO1537
Lepidoptera|[2495]|GMAGA232-15|Lepidoptera|||BOLD:ACV0078
Crambidae|[2496]|GMARA458-14|Lepidoptera|Crambidae||BOLD:ACM9471
Crambidae|[2497]|GMARK2372-14|Lepidoptera|Crambidae||BOLD:ACN8957
Lepidoptera|[2498]|GMARQ716-14|Lepidoptera|||BOLD:ACN8419
Musotiminae|[2499]|GMARA454-14|Lepidoptera|Crambidae|Musotiminae|BOLD:ACM9379
Crambidae|[2500]|GMAGA298-15|Lepidoptera|Crambidae||BOLD:ACN2878
Crambidae|[2501]|GMARL1625-14|Lepidoptera|Crambidae||BOLD:ACN2015
Elachistidae|[2502]|GMAGE1250-15|Lepidoptera|Elachistidae||BOLD:ACN2292
Gelechiidae|[2503]|GMAGB975-15|Lepidoptera|Gelechiidae||BOLD:ACW7607
Gelechiidae|[2504]|GMARR827-14|Lepidoptera|Gelechiidae||BOLD:ACN8382
Gelechiidae|[2505]|GMAGA291-15|Lepidoptera|Gelechiidae||BOLD:ACM8557
Gelechiidae|[2506]|GMAGJ1439-15|Lepidoptera|Gelechiidae||BOLD:ACW9937
Lepidoptera|[2507]|GMAGB044-15|Lepidoptera|||BOLD:ACM4867
Chionodes|[2508]|GMAGD1565-15|Lepidoptera|Gelechiidae|Gelechiinae|BOLD:ACN2621
Gelechiidae|[2509]|GMAGO063-15|Lepidoptera|Gelechiidae||BOLD:ACX4897
Gelechiidae|[2510]|GMAGQ596-15|Lepidoptera|Gelechiidae||BOLD:ACN7699
Gelechiidae|[2511]|GMARN018-14|Lepidoptera|Gelechiidae||BOLD:ACN7011
Gelechiidae|[2512]|GMAGJ1447-15|Lepidoptera|Gelechiidae||BOLD:ACN2749
Gelechiidae|[2513]|GMARS088-14|Lepidoptera|Gelechiidae||BOLD:ACN7769
Gelechiidae|[2514]|GMAGZ010-15|Lepidoptera|Gelechiidae||BOLD:ACX4495
Lepidoptera|[2515]|GMAGP093-15|Lepidoptera|||BOLD:ACN3170
Lepidoptera|[2516]|GMARO005-14|Lepidoptera|||BOLD:ACO1188
Symmetrischema striatella|[2517]|GMAGQ582-15|Lepidoptera|Gelechiidae|Gelechiinae|BOLD:AAD8376
Lepidoptera|[2518]|GMAGO059-15|Lepidoptera|||BOLD:ACX5830
Glauce|[2519]|GMARL1620-14|Lepidoptera|Gelechiidae|Gelechiinae|BOLD:ACN2833
Gelechiidae|[2520]|GMAGG118-15|Lepidoptera|Gelechiidae||BOLD:ACW4855
Gelechiidae|[2521]|GMARU665-14|Lepidoptera|Gelechiidae||BOLD:ACO1078
Lepidoptera|[2522]|GMAGN427-15|Lepidoptera|||BOLD:ACX4083
Lepidoptera|[2523]|GMART1401-14|Lepidoptera|||BOLD:ACN9959
Pyrausta vanalis|[2524]|GMAGD1557-15|Lepidoptera|Crambidae|Pyraustinae|BOLD:AAZ2486
Anania|[2525]|GMAGN030-15|Lepidoptera|Crambidae|Pyraustinae|BOLD:ACX7477
Pyraustinae|[2526]|GMAGL1418-15|Lepidoptera|Crambidae|Pyraustinae|BOLD:ABW7416
Loxostege|[2527]|GMAGA222-15|Lepidoptera|Crambidae|Pyraustinae|BOLD:ACB9353
Diaphania|[2528]|GMAGD094-15|Lepidoptera|Crambidae|Spilomelinae|BOLD:ACW8961
Diaphania|[2529]|GMAGD090-15|Lepidoptera|Crambidae|Spilomelinae|BOLD:ABZ3756
Diaphania|[2530]|GMAGG051-15|Lepidoptera|Crambidae|Spilomelinae|BOLD:AAF8448
Crambidae|[2531]|GMARB116-14|Lepidoptera|Crambidae||BOLD:ACN0411
Crambidae|[2532]|GMAGQ534-15|Lepidoptera|Crambidae||BOLD:ACX4531
Crambidae|[2533]|GMAGT928-15|Lepidoptera|Crambidae||BOLD:ACX5408
Crambidae|[2534]|GMARF1131-14|Lepidoptera|Crambidae||BOLD:ACN0717
Crambidae|[2535]|GMARR791-14|Lepidoptera|Crambidae||BOLD:ACN8307
Samea|[2536]|GMAGE049-15|Lepidoptera|Crambidae|Spilomelinae|BOLD:ACM5071
Crambidae|[2537]|GMARC107-14|Lepidoptera|Crambidae||BOLD:ACN1675
Diasemiodes|[2538]|GMARB112-14|Lepidoptera|Crambidae|Spilomelinae|BOLD:ACN1333
Crambidae|[2539]|GMARC835-14|Lepidoptera|Crambidae||BOLD:ACM6484
Eulepte|[2540]|GMAGB735-15|Lepidoptera|Crambidae|Spilomelinae|BOLD:ACW9593
Salbia|[2541]|GMART033-14|Lepidoptera|Crambidae|Spilomelinae|BOLD:ACO1985
Salbia|[2542]|GMAGH901-15|Lepidoptera|Crambidae|Spilomelinae|BOLD:AAP1982
Spilomelinae|[2543]|GMAGK149-15|Lepidoptera|Crambidae|Spilomelinae|BOLD:AAL8603
Pilemia|[2544]|GMAGQ094-15|Lepidoptera|Crambidae|Pyraustinae|BOLD:ACX6405
Spilomelinae|[2545]|GMART020-14|Lepidoptera|Crambidae|Spilomelinae|BOLD:ACB9166
Syllepis hortalis|[2546]|GMARD044-14|Lepidoptera|Crambidae|Spilomelinae|BOLD:AAA9670
Crambidae|[2547]|GMAGE1263-15|Lepidoptera|Crambidae||BOLD:ACW1743
Crambidae|[2548]|GMAGG062-15|Lepidoptera|Crambidae||BOLD:ACX5597
Herpetogramma phaeopteralis|[2549]|GMARD041-14|Lepidoptera|Crambidae|Spilomelinae|BOLD:ABY7602
Hymenia perspectalis|[2550]|GMAGE048-15|Lepidoptera|Crambidae|Spilomelinae|BOLD:AAA0344
Hymenia perspectalis|[2551]|GMARC104-14|Lepidoptera|Crambidae|Spilomelinae|BOLD:ABY5783
Spilomelinae|[2552]|GMAGG057-15|Lepidoptera|Crambidae|Spilomelinae|BOLD:ACX4731
Spilomelinae|[2553]|GMARE013-14|Lepidoptera|Crambidae|Spilomelinae|BOLD:ACN0621
Spilomelinae|[2554]|GMAGK018-15|Lepidoptera|Crambidae|Spilomelinae|BOLD:ACY6333
Crambidae|[2555]|GMAGG052-15|Lepidoptera|Crambidae||BOLD:ABW7357
Desmia|[2556]|GMAGC1511-15|Lepidoptera|Crambidae|Spilomelinae|BOLD:ACN0324
Desmia|[2557]|GMART027-14|Lepidoptera|Crambidae|Spilomelinae|BOLD:AAM1532
Apogeshna|[2558]|GMARK314-14|Lepidoptera|Crambidae|Spilomelinae|BOLD:AAA0336
Crambidae|[2559]|GMARA520-14|Lepidoptera|Crambidae||BOLD:ACM5842
Spilomelinae|[2560]|GMAGA249-15|Lepidoptera|Crambidae|Spilomelinae|BOLD:ACM8404
Crambidae|[2561]|GMAGO1483-15|Lepidoptera|Crambidae||BOLD:ACX4234
Crambidae|[2562]|GMAGQ553-15|Lepidoptera|Crambidae||BOLD:ACX4255
Udea secticostalis|[2563]|GMARM017-14|Lepidoptera|Crambidae|Spilomelinae|BOLD:AAZ9806
Lepidoptera|[2564]|GMAGR272-15|Lepidoptera|||BOLD:ABA1741
Crambidae|[2565]|GMARK2373-14|Lepidoptera|Crambidae||BOLD:ACN1130
Noctuidae|[2566]|GMARA478-14|Lepidoptera|Noctuidae||BOLD:ACM9008
Lepidoptera|[2567]|GMAGM1524-15|Lepidoptera|||BOLD:ACN2426
Chytonix|[2568]|GMAGY073-15|Lepidoptera|Noctuidae|Noctuinae|BOLD:ACX5302
Erebidae|[2569]|GMAGQ079-15|Lepidoptera|Erebidae||BOLD:ACX7259
Agonopterix|[2570]|GMAGN404-15|Lepidoptera|Depressariidae|Depressariinae|BOLD:ACN9462
Codatractus aminias|[2571]|GMAGY060-15|Lepidoptera|Hesperiidae|Pyrginae|BOLD:AAZ3952
Depressariidae|[2572]|GMAGA267-15|Lepidoptera|Depressariidae||BOLD:ACN1617
Lepidoptera|[2573]|GMAGW432-15|Lepidoptera|||BOLD:ACX3223
Gelechiidae|[2574]|GMAGA227-15|Lepidoptera|Gelechiidae||BOLD:ACM8858
Gelechiidae|[2575]|GMAGA223-15|Lepidoptera|Gelechiidae||BOLD:ACN1892
Gelechiidae|[2576]|GMARA402-14|Lepidoptera|Gelechiidae||BOLD:ACM9656
Lepidoptera|[2577]|GMAGA288-15|Lepidoptera|||BOLD:ACV0211
Lepidoptera|[2578]|GMAGP161-15|Lepidoptera|||BOLD:ACN4069
Argyria centrifugens|[2579]|GMARK2271-14|Lepidoptera|Crambidae|Crambinae|BOLD:AAC0264
Gelechiidae|[2580]|GMART1688-14|Lepidoptera|Gelechiidae||BOLD:ACO1558
Lepidoptera|[2581]|GMAGY266-15|Lepidoptera|||BOLD:ACX1587
Adelpha syma|[2582]|GMAGP074-15|Lepidoptera|Nymphalidae|Limenitidinae|BOLD:AAY7657
Adelpha thessalia|[2583]|GMAGA027-15|Lepidoptera|Nymphalidae|Limenitidinae|BOLD:AAZ5032
Parypthimoides phronius|[2584]|GMARA1877-14|Lepidoptera|Nymphalidae|Satyrinae|BOLD:AAX1665
Parypthimoides poltys|[2585]|GMAGA056-15|Lepidoptera|Nymphalidae|Satyrinae|BOLD:AAX1664
Moneuptychia paeon|[2586]|GMARK004-14|Lepidoptera|Nymphalidae|Satyrinae|BOLD:AAM4884
Moneuptychia griseldis|[2587]|GMAGN020-15|Lepidoptera|Nymphalidae|Satyrinae|BOLD:AAZ1921
Moneuptychia soter|[2588]|GMARA1881-14|Lepidoptera|Nymphalidae|Satyrinae|BOLD:AAM4885
Godartiana muscosa|[2589]|GMARE025-14|Lepidoptera|Nymphalidae|Satyrinae|BOLD:AAY0914

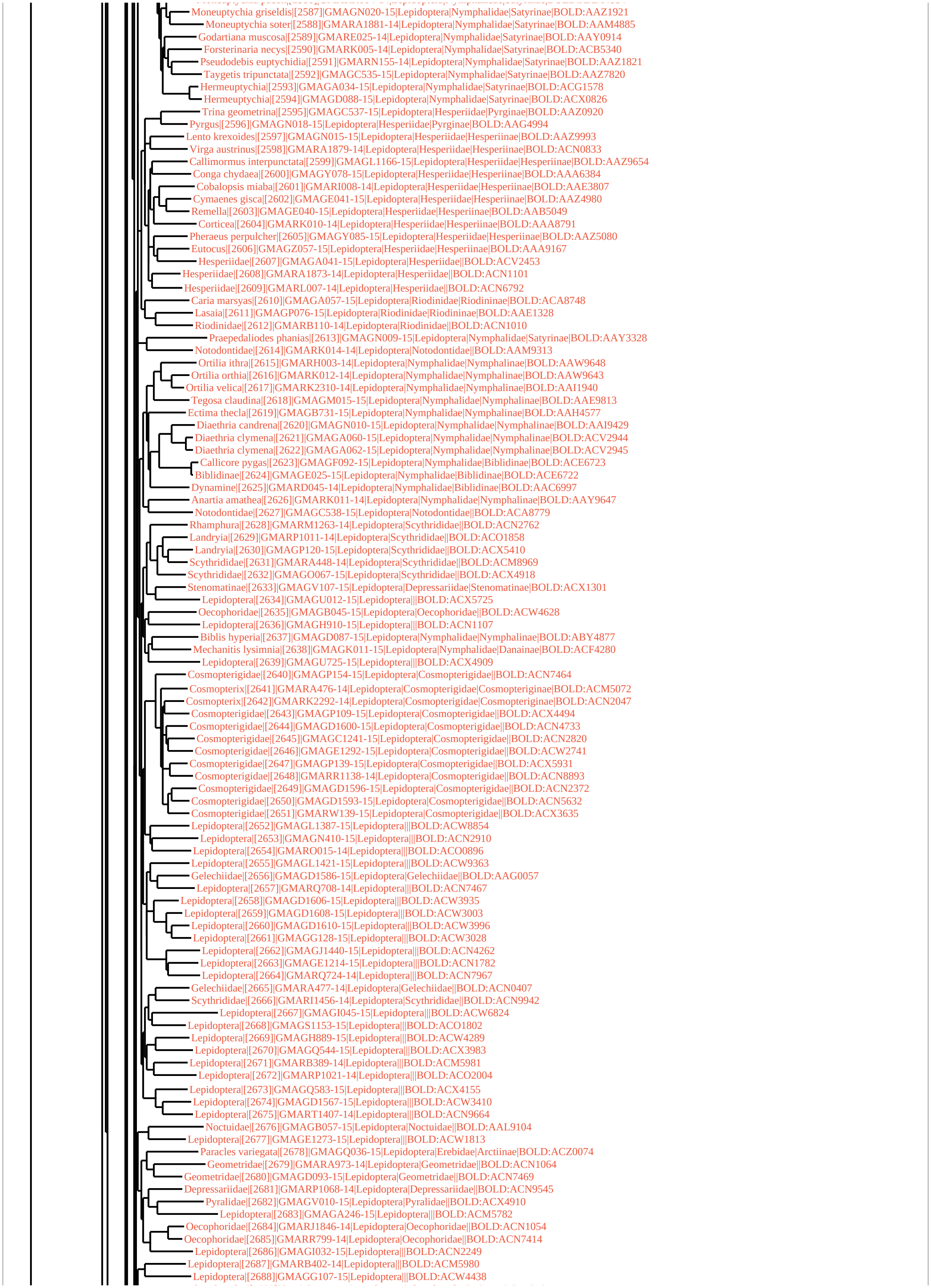
Moneuptychia griseldis|[2587]|GMAGN020-15|Lepidoptera|Nymphalidae|Satyrinae|BOLD:AAZ1921
Moneuptychia soter|[2588]|GMARA1881-14|Lepidoptera|Nymphalidae|Satyrinae|BOLD:AAM4885
Godartiana muscosa|[2589]|GMARE025-14|Lepidoptera|Nymphalidae|Satyrinae|BOLD:AAY0914
Forsterinaria necys|[2590]|GMARK005-14|Lepidoptera|Nymphalidae|Satyrinae|BOLD:ACB5340
Pseudodebis euptychidia|[2591]|GMARN155-14|Lepidoptera|Nymphalidae|Satyrinae|BOLD:AAZ1821
Taygetis tripunctata|[2592]|GMAGC535-15|Lepidoptera|Nymphalidae|Satyrinae|BOLD:AAZ7820
Hermeuptychia|[2593]|GMAGA034-15|Lepidoptera|Nymphalidae|Satyrinae|BOLD:ACG1578
Hermeuptychia|[2594]|GMAGD088-15|Lepidoptera|Nymphalidae|Satyrinae|BOLD:ACX0826
Trina geometrina|[2595]|GMAGC537-15|Lepidoptera|Hesperiidae|Pyrginae|BOLD:AAZ0920
Pyrgus|[2596]|GMAGN018-15|Lepidoptera|Hesperiidae|Pyrginae|BOLD:AAG4994
Lento krexoides|[2597]|GMAGN015-15|Lepidoptera|Hesperiidae|Hesperiinae|BOLD:AAZ9993
Virga austrinus|[2598]|GMARA1879-14|Lepidoptera|Hesperiidae|Hesperiinae|BOLD:ACN0833
Callimormus interpunctata|[2599]|GMAGL1166-15|Lepidoptera|Hesperiidae|Hesperiinae|BOLD:AAZ9654
Conga chydaea|[2600]|GMAGY078-15|Lepidoptera|Hesperiidae|Hesperiinae|BOLD:AAA6384
Cobalopsis miaba|[2601]|GMARI008-14|Lepidoptera|Hesperiidae|Hesperiinae|BOLD:AAE3807
Cymaenes gisca|[2602]|GMAGE041-15|Lepidoptera|Hesperiidae|Hesperiinae|BOLD:AAZ4980
Remella|[2603]|GMAGE040-15|Lepidoptera|Hesperiidae|Hesperiinae|BOLD:AAB5049
Corticea|[2604]|GMARK010-14|Lepidoptera|Hesperiidae|Hesperiinae|BOLD:AAA8791
Pheraeus perpulcher|[2605]|GMAGY085-15|Lepidoptera|Hesperiidae|Hesperiinae|BOLD:AAZ5080
Eutocus|[2606]|GMAGZ057-15|Lepidoptera|Hesperiidae|Hesperiinae|BOLD:AAA9167
Hesperiidae|[2607]|GMAGA041-15|Lepidoptera|Hesperiidae||BOLD:ACV2453
Hesperiidae|[2608]|GMARA1873-14|Lepidoptera|Hesperiidae||BOLD:ACN1101
Hesperiidae|[2609]|GMARL007-14|Lepidoptera|Hesperiidae||BOLD:ACN6792
Caria marsyas|[2610]|GMAGA057-15|Lepidoptera|Riodinidae|Riodininae|BOLD:ACA8748
Lasaia|[2611]|GMAGP076-15|Lepidoptera|Riodinidae|Riodininae|BOLD:AAE1328
Riodinidae|[2612]|GMARB110-14|Lepidoptera|Riodinidae||BOLD:ACN1010
Praepedaliodes phanias|[2613]|GMAGN009-15|Lepidoptera|Nymphalidae|Satyrinae|BOLD:AAY3328
Notodontidae|[2614]|GMARK014-14|Lepidoptera|Notodontidae||BOLD:AAM9313
Ortilia ithra|[2615]|GMARH003-14|Lepidoptera|Nymphalidae|Nymphalinae|BOLD:AAW9648
Ortilia orthia|[2616]|GMARK012-14|Lepidoptera|Nymphalidae|Nymphalinae|BOLD:AAW9643
Ortilia velica|[2617]|GMARK2310-14|Lepidoptera|Nymphalidae|Nymphalinae|BOLD:AAI1940
Tegosa claudina|[2618]|GMAGM015-15|Lepidoptera|Nymphalidae|Nymphalinae|BOLD:AAE9813
Ectima thecla|[2619]|GMAGB731-15|Lepidoptera|Nymphalidae|Nymphalinae|BOLD:AAH4577
Diaethria candrena|[2620]|GMAGN010-15|Lepidoptera|Nymphalidae|Nymphalinae|BOLD:AAI9429
Diaethria clymena|[2621]|GMAGA060-15|Lepidoptera|Nymphalidae|Nymphalinae|BOLD:ACV2944
Diaethria clymena|[2622]|GMAGA062-15|Lepidoptera|Nymphalidae|Nymphalinae|BOLD:ACV2945
Callicore pygas|[2623]|GMAGF092-15|Lepidoptera|Nymphalidae|Biblidinae|BOLD:ACE6723
Biblidinae|[2624]|GMAGE025-15|Lepidoptera|Nymphalidae|Biblidinae|BOLD:ACE6722
Dynamine|[2625]|GMARD045-14|Lepidoptera|Nymphalidae|Biblidinae|BOLD:AAC6997
Anartia amathea|[2626]|GMARK011-14|Lepidoptera|Nymphalidae|Nymphalinae|BOLD:AAY9647
Notodontidae|[2627]|GMAGC538-15|Lepidoptera|Notodontidae||BOLD:ACA8779
Rhamphura|[2628]|GMARM1263-14|Lepidoptera|Scythrididae||BOLD:ACN2762
Landryia|[2629]|GMARP1011-14|Lepidoptera|Scythrididae||BOLD:ACO1858
Landryia|[2630]|GMAGP120-15|Lepidoptera|Scythrididae||BOLD:ACX5410
Scythrididae|[2631]|GMARA448-14|Lepidoptera|Scythrididae||BOLD:ACM8969
Scythrididae|[2632]|GMAGO067-15|Lepidoptera|Scythrididae||BOLD:ACX4918
Stenomatinae|[2633]|GMAGV107-15|Lepidoptera|Depressariidae|Stenomatinae|BOLD:ACX1301
Lepidoptera|[2634]|GMAGU012-15|Lepidoptera|||BOLD:ACX5725
Oecophoridae|[2635]|GMAGB045-15|Lepidoptera|Oecophoridae||BOLD:ACW4628
Lepidoptera|[2636]|GMAGH910-15|Lepidoptera|||BOLD:ACN1107
Biblis hyperia|[2637]|GMAGD087-15|Lepidoptera|Nymphalidae|Nymphalinae|BOLD:ABY4877
Mechanitis lysimnia|[2638]|GMAGK011-15|Lepidoptera|Nymphalidae|Danainae|BOLD:ACF4280
Lepidoptera|[2639]|GMAGU725-15|Lepidoptera|||BOLD:ACX4909
Cosmopterigidae|[2640]|GMAGP154-15|Lepidoptera|Cosmopterigidae||BOLD:ACN7464
Cosmopterix|[2641]|GMARA476-14|Lepidoptera|Cosmopterigidae|Cosmopteriginae|BOLD:ACM5072
Cosmopterix|[2642]|GMARK2292-14|Lepidoptera|Cosmopterigidae|Cosmopteriginae|BOLD:ACN2047
Cosmopterigidae|[2643]|GMAGP109-15|Lepidoptera|Cosmopterigidae||BOLD:ACX4494
Cosmopterigidae|[2644]|GMAGD1600-15|Lepidoptera|Cosmopterigidae||BOLD:ACN4733
Cosmopterigidae|[2645]|GMAGC1241-15|Lepidoptera|Cosmopterigidae||BOLD:ACN2820
Cosmopterigidae|[2646]|GMAGE1292-15|Lepidoptera|Cosmopterigidae||BOLD:ACW2741
Cosmopterigidae|[2647]|GMAGP139-15|Lepidoptera|Cosmopterigidae||BOLD:ACX5931
Cosmopterigidae|[2648]|GMARR1138-14|Lepidoptera|Cosmopterigidae||BOLD:ACN8893
Cosmopterigidae|[2649]|GMAGD1596-15|Lepidoptera|Cosmopterigidae||BOLD:ACN2372
Cosmopterigidae|[2650]|GMAGD1593-15|Lepidoptera|Cosmopterigidae||BOLD:ACN5632
Cosmopterigidae|[2651]|GMARW139-15|Lepidoptera|Cosmopterigidae||BOLD:ACX3635
Lepidoptera|[2652]|GMAGL1387-15|Lepidoptera|||BOLD:ACW8854
Lepidoptera|[2653]|GMAGN410-15|Lepidoptera|||BOLD:ACN2910
Lepidoptera|[2654]|GMARO015-14|Lepidoptera|||BOLD:ACO0896
Lepidoptera|[2655]|GMAGL1421-15|Lepidoptera|||BOLD:ACW9363
Gelechiidae|[2656]|GMAGD1586-15|Lepidoptera|Gelechiidae||BOLD:AAG0057
Lepidoptera|[2657]|GMARQ708-14|Lepidoptera|||BOLD:ACN7467
Lepidoptera|[2658]|GMAGD1606-15|Lepidoptera|||BOLD:ACW3935
Lepidoptera|[2659]|GMAGD1608-15|Lepidoptera|||BOLD:ACW3003
Lepidoptera|[2660]|GMAGD1610-15|Lepidoptera|||BOLD:ACW3996
Lepidoptera|[2661]|GMAGG128-15|Lepidoptera|||BOLD:ACW3028
Lepidoptera|[2662]|GMAGJ1440-15|Lepidoptera|||BOLD:ACN4262
Lepidoptera|[2663]|GMAGE1214-15|Lepidoptera|||BOLD:ACN1782
Lepidoptera|[2664]|GMARQ724-14|Lepidoptera|||BOLD:ACN7967
Gelechiidae|[2665]|GMARA477-14|Lepidoptera|Gelechiidae||BOLD:ACN0407
Scythrididae|[2666]|GMARI1456-14|Lepidoptera|Scythrididae||BOLD:ACN9942
Lepidoptera|[2667]|GMAGI045-15|Lepidoptera|||BOLD:ACW6824
Lepidoptera|[2668]|GMAGS1153-15|Lepidoptera|||BOLD:ACO1802
Lepidoptera|[2669]|GMAGH889-15|Lepidoptera|||BOLD:ACW4289
Lepidoptera|[2670]|GMAGQ544-15|Lepidoptera|||BOLD:ACX3983
Lepidoptera|[2671]|GMARB389-14|Lepidoptera|||BOLD:ACM5981
Lepidoptera|[2672]|GMARP1021-14|Lepidoptera|||BOLD:ACO2004
Lepidoptera|[2673]|GMAGQ583-15|Lepidoptera|||BOLD:ACX4155
Lepidoptera|[2674]|GMAGD1567-15|Lepidoptera|||BOLD:ACW3410
Lepidoptera|[2675]|GMART1407-14|Lepidoptera|||BOLD:ACN9664
Noctuidae|[2676]|GMAGB057-15|Lepidoptera|Noctuidae||BOLD:AAL9104
Lepidoptera|[2677]|GMAGE1273-15|Lepidoptera|||BOLD:ACW1813
Paracles variegata|[2678]|GMAGQ036-15|Lepidoptera|Erebidae|Arctiinae|BOLD:ACZ0074
Geometridae|[2679]|GMARA973-14|Lepidoptera|Geometridae||BOLD:ACN1064
Geometridae|[2680]|GMAGD093-15|Lepidoptera|Geometridae||BOLD:ACN7469
Depressariidae|[2681]|GMARP1068-14|Lepidoptera|Depressariidae||BOLD:ACN9545
Pyralidae|[2682]|GMAGV010-15|Lepidoptera|Pyralidae||BOLD:ACX4910
Lepidoptera|[2683]|GMAGA246-15|Lepidoptera|||BOLD:ACM5782
Oecophoridae|[2684]|GMARJ1846-14|Lepidoptera|Oecophoridae||BOLD:ACN1054
Oecophoridae|[2685]|GMARR799-14|Lepidoptera|Oecophoridae||BOLD:ACN7414
Lepidoptera|[2686]|GMAGI032-15|Lepidoptera|||BOLD:ACN2249
Lepidoptera|[2687]|GMARB402-14|Lepidoptera|||BOLD:ACM5980
Lepidoptera|[2688]|GMAGG107-15|Lepidoptera|||BOLD:ACW4438

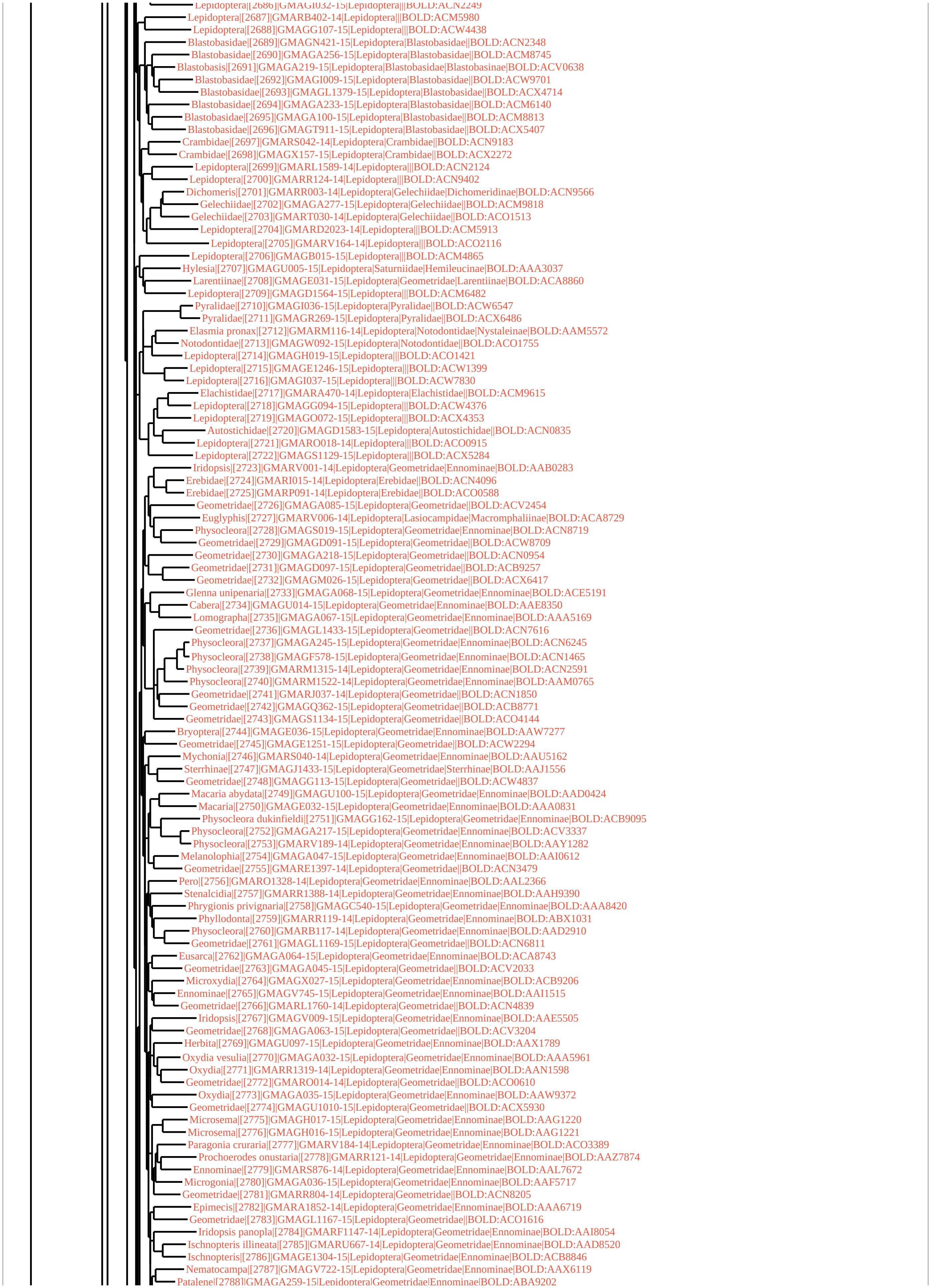
Lepidoptera|[2686]|GMAGI032-15|Lepidoptera|||BOLD:ACN2249
Lepidoptera|[2687]|GMARB402-14|Lepidoptera|||BOLD:ACM5980
Lepidoptera|[2688]|GMAGG107-15|Lepidoptera|||BOLD:ACW4438
Blastobasidae|[2689]|GMAGN421-15|Lepidoptera|Blastobasidae||BOLD:ACN2348
Blastobasidae|[2690]|GMAGA256-15|Lepidoptera|Blastobasidae||BOLD:ACM8745
Blastobasis|[2691]|GMAGA219-15|Lepidoptera|Blastobasidae|Blastobasinae|BOLD:ACV0638
Blastobasidae|[2692]|GMAGI009-15|Lepidoptera|Blastobasidae||BOLD:ACW9701
Blastobasidae|[2693]|GMAGL1379-15|Lepidoptera|Blastobasidae||BOLD:ACX4714
Blastobasidae|[2694]|GMAGA233-15|Lepidoptera|Blastobasidae||BOLD:ACM6140
Blastobasidae|[2695]|GMAGA100-15|Lepidoptera|Blastobasidae||BOLD:ACM8813
Blastobasidae|[2696]|GMAGT911-15|Lepidoptera|Blastobasidae||BOLD:ACX5407
Crambidae|[2697]|GMARS042-14|Lepidoptera|Crambidae||BOLD:ACN9183
Crambidae|[2698]|GMAGX157-15|Lepidoptera|Crambidae||BOLD:ACX2272
Lepidoptera|[2699]|GMARL1589-14|Lepidoptera|||BOLD:ACN2124
Lepidoptera|[2700]|GMARR124-14|Lepidoptera|||BOLD:ACN9402
Dichomeris|[2701]|GMARR003-14|Lepidoptera|Gelechiidae|Dichomeridinae|BOLD:ACN9566
Gelechiidae|[2702]|GMAGA277-15|Lepidoptera|Gelechiidae||BOLD:ACM9818
Gelechiidae|[2703]|GMART030-14|Lepidoptera|Gelechiidae||BOLD:ACO1513
Lepidoptera|[2704]|GMARD2023-14|Lepidoptera|||BOLD:ACM5913
Lepidoptera|[2705]|GMARV164-14|Lepidoptera|||BOLD:ACO2116
Lepidoptera|[2706]|GMAGB015-15|Lepidoptera|||BOLD:ACM4865
Hylesia|[2707]|GMAGU005-15|Lepidoptera|Saturniidae|Hemileucinae|BOLD:AAA3037
Larentiinae|[2708]|GMAGE031-15|Lepidoptera|Geometridae|Larentiinae|BOLD:ACA8860
Lepidoptera|[2709]|GMAGD1564-15|Lepidoptera|||BOLD:ACM6482
Pyralidae|[2710]|GMAGI036-15|Lepidoptera|Pyralidae||BOLD:ACW6547
Pyralidae|[2711]|GMAGR269-15|Lepidoptera|Pyralidae||BOLD:ACX6486
Elasmia pronax|[2712]|GMARM116-14|Lepidoptera|Notodontidae|Nystaleinae|BOLD:AAM5572
Notodontidae|[2713]|GMAGW092-15|Lepidoptera|Notodontidae||BOLD:ACO1755
Lepidoptera|[2714]|GMAGH019-15|Lepidoptera|||BOLD:ACO1421
Lepidoptera|[2715]|GMAGE1246-15|Lepidoptera|||BOLD:ACW1399
Lepidoptera|[2716]|GMAGI037-15|Lepidoptera|||BOLD:ACW7830
Elachistidae|[2717]|GMARA470-14|Lepidoptera|Elachistidae||BOLD:ACM9615
Lepidoptera|[2718]|GMAGG094-15|Lepidoptera|||BOLD:ACW4376
Lepidoptera|[2719]|GMAGO072-15|Lepidoptera|||BOLD:ACX4353
Autostichidae|[2720]|GMAGD1583-15|Lepidoptera|Autostichidae||BOLD:ACN0835
Lepidoptera|[2721]|GMARO018-14|Lepidoptera|||BOLD:ACO0915
Lepidoptera|[2722]|GMAGS1129-15|Lepidoptera|||BOLD:ACX5284
Iridopsis|[2723]|GMARV001-14|Lepidoptera|Geometridae|Ennominae|BOLD:AAB0283
Erebidae|[2724]|GMARI015-14|Lepidoptera|Erebidae||BOLD:ACN4096
Erebidae|[2725]|GMARP091-14|Lepidoptera|Erebidae||BOLD:ACO0588
Geometridae|[2726]|GMAGA085-15|Lepidoptera|Geometridae||BOLD:ACV2454
Euglyphis|[2727]|GMARV006-14|Lepidoptera|Lasiocampidae|Macromphaliinae|BOLD:ACA8729
Physocleora|[2728]|GMAGS019-15|Lepidoptera|Geometridae|Ennominae|BOLD:ACN8719
Geometridae|[2729]|GMAGD091-15|Lepidoptera|Geometridae||BOLD:ACW8709
Geometridae|[2730]|GMAGA218-15|Lepidoptera|Geometridae||BOLD:ACN0954
Geometridae|[2731]|GMAGD097-15|Lepidoptera|Geometridae||BOLD:ACB9257
Geometridae|[2732]|GMAGM026-15|Lepidoptera|Geometridae||BOLD:ACX6417
Glenna unipenaria|[2733]|GMAGA068-15|Lepidoptera|Geometridae|Ennominae|BOLD:ACE5191
Cabera|[2734]|GMAGU014-15|Lepidoptera|Geometridae|Ennominae|BOLD:AAE8350
Lomographa|[2735]|GMAGA067-15|Lepidoptera|Geometridae|Ennominae|BOLD:AAA5169
Geometridae|[2736]|GMAGL1433-15|Lepidoptera|Geometridae||BOLD:ACN7616
Physocleora|[2737]|GMAGA245-15|Lepidoptera|Geometridae|Ennominae|BOLD:ACN6245
Physocleora|[2738]|GMAGF578-15|Lepidoptera|Geometridae|Ennominae|BOLD:ACN1465
Physocleora|[2739]|GMARM1315-14|Lepidoptera|Geometridae|Ennominae|BOLD:ACN2591
Physocleora|[2740]|GMARM1522-14|Lepidoptera|Geometridae|Ennominae|BOLD:AAM0765
Geometridae|[2741]|GMARJ037-14|Lepidoptera|Geometridae||BOLD:ACN1850
Geometridae|[2742]|GMAGQ362-15|Lepidoptera|Geometridae||BOLD:ACB8771
Geometridae|[2743]|GMAGS1134-15|Lepidoptera|Geometridae||BOLD:ACO4144
Bryoptera|[2744]|GMAGE036-15|Lepidoptera|Geometridae|Ennominae|BOLD:AAW7277
Geometridae|[2745]|GMAGE1251-15|Lepidoptera|Geometridae||BOLD:ACW2294
Mychonia|[2746]|GMARS040-14|Lepidoptera|Geometridae|Ennominae|BOLD:AAU5162
Sterrhinae|[2747]|GMAGJ1433-15|Lepidoptera|Geometridae|Sterrhinae|BOLD:AAJ1556
Geometridae|[2748]|GMAGG113-15|Lepidoptera|Geometridae||BOLD:ACW4837
Macaria abydata|[2749]|GMAGU100-15|Lepidoptera|Geometridae|Ennominae|BOLD:AAD0424
Macaria|[2750]|GMAGE032-15|Lepidoptera|Geometridae|Ennominae|BOLD:AAA0831
Physocleora dukinfieldi|[2751]|GMAGG162-15|Lepidoptera|Geometridae|Ennominae|BOLD:ACB9095
Physocleora|[2752]|GMAGA217-15|Lepidoptera|Geometridae|Ennominae|BOLD:ACV3337
Physocleora|[2753]|GMARV189-14|Lepidoptera|Geometridae|Ennominae|BOLD:AAY1282
Melanolophia|[2754]|GMAGA047-15|Lepidoptera|Geometridae|Ennominae|BOLD:AAI0612
Geometridae|[2755]|GMARE1397-14|Lepidoptera|Geometridae||BOLD:ACN3479
Pero|[2756]|GMARO1328-14|Lepidoptera|Geometridae|Ennominae|BOLD:AAL2366
Stenalcidia|[2757]|GMARR1388-14|Lepidoptera|Geometridae|Ennominae|BOLD:AAH9390
Phrygionis privignaria|[2758]|GMAGC540-15|Lepidoptera|Geometridae|Ennominae|BOLD:AAA8420
Phyllodonta|[2759]|GMARR119-14|Lepidoptera|Geometridae|Ennominae|BOLD:ABX1031
Physocleora|[2760]|GMARB117-14|Lepidoptera|Geometridae|Ennominae|BOLD:AAD2910
Geometridae|[2761]|GMAGL1169-15|Lepidoptera|Geometridae||BOLD:ACN6811
Eusarca|[2762]|GMAGA064-15|Lepidoptera|Geometridae|Ennominae|BOLD:ACA8743
Geometridae|[2763]|GMAGA045-15|Lepidoptera|Geometridae||BOLD:ACV2033
Microxydia|[2764]|GMAGX027-15|Lepidoptera|Geometridae|Ennominae|BOLD:ACB9206
Ennominae|[2765]|GMAGV745-15|Lepidoptera|Geometridae|Ennominae|BOLD:AAI1515
Geometridae|[2766]|GMARL1760-14|Lepidoptera|Geometridae||BOLD:ACN4839
Iridopsis|[2767]|GMAGV009-15|Lepidoptera|Geometridae|Ennominae|BOLD:AAE5505
Geometridae|[2768]|GMAGA063-15|Lepidoptera|Geometridae||BOLD:ACV3204
Herbita|[2769]|GMAGU097-15|Lepidoptera|Geometridae|Ennominae|BOLD:AAX1789
Oxydia vesulia|[2770]|GMAGA032-15|Lepidoptera|Geometridae|Ennominae|BOLD:AAA5961
Oxydia|[2771]|GMARR1319-14|Lepidoptera|Geometridae|Ennominae|BOLD:AAN1598
Geometridae|[2772]|GMARO014-14|Lepidoptera|Geometridae||BOLD:ACO0610
Oxydia|[2773]|GMAGA035-15|Lepidoptera|Geometridae|Ennominae|BOLD:AAW9372
Geometridae|[2774]|GMAGU1010-15|Lepidoptera|Geometridae||BOLD:ACX5930
Microsema|[2775]|GMAGH017-15|Lepidoptera|Geometridae|Ennominae|BOLD:AAG1220
Microsema|[2776]|GMAGH016-15|Lepidoptera|Geometridae|Ennominae|BOLD:AAG1221
Paragonia cruraria|[2777]|GMARV184-14|Lepidoptera|Geometridae|Ennominae|BOLD:ACO3389
Prochoerodes onustaria|[2778]|GMARR121-14|Lepidoptera|Geometridae|Ennominae|BOLD:AAZ7874
Ennominae|[2779]|GMARS876-14|Lepidoptera|Geometridae|Ennominae|BOLD:AAL7672
Microgonia|[2780]|GMAGA036-15|Lepidoptera|Geometridae|Ennominae|BOLD:AAF5717
Geometridae|[2781]|GMARR804-14|Lepidoptera|Geometridae||BOLD:ACN8205
Epimecis|[2782]|GMARA1852-14|Lepidoptera|Geometridae|Ennominae|BOLD:AAA6719
Geometridae|[2783]|GMAGL1167-15|Lepidoptera|Geometridae||BOLD:ACO1616
Iridopsis panopla|[2784]|GMARF1147-14|Lepidoptera|Geometridae|Ennominae|BOLD:AAI8054
Ischnopteris illineata|[2785]|GMARU667-14|Lepidoptera|Geometridae|Ennominae|BOLD:AAD8520
Ischnopteris|[2786]|GMAGE1304-15|Lepidoptera|Geometridae|Ennominae|BOLD:ACB8846
Nematocampa|[2787]|GMAGV722-15|Lepidoptera|Geometridae|Ennominae|BOLD:AAX6119
Patalene|[2788]|GMAGA259-15|Lepidoptera|Geometridae|Ennominae|BOLD:ABA9202

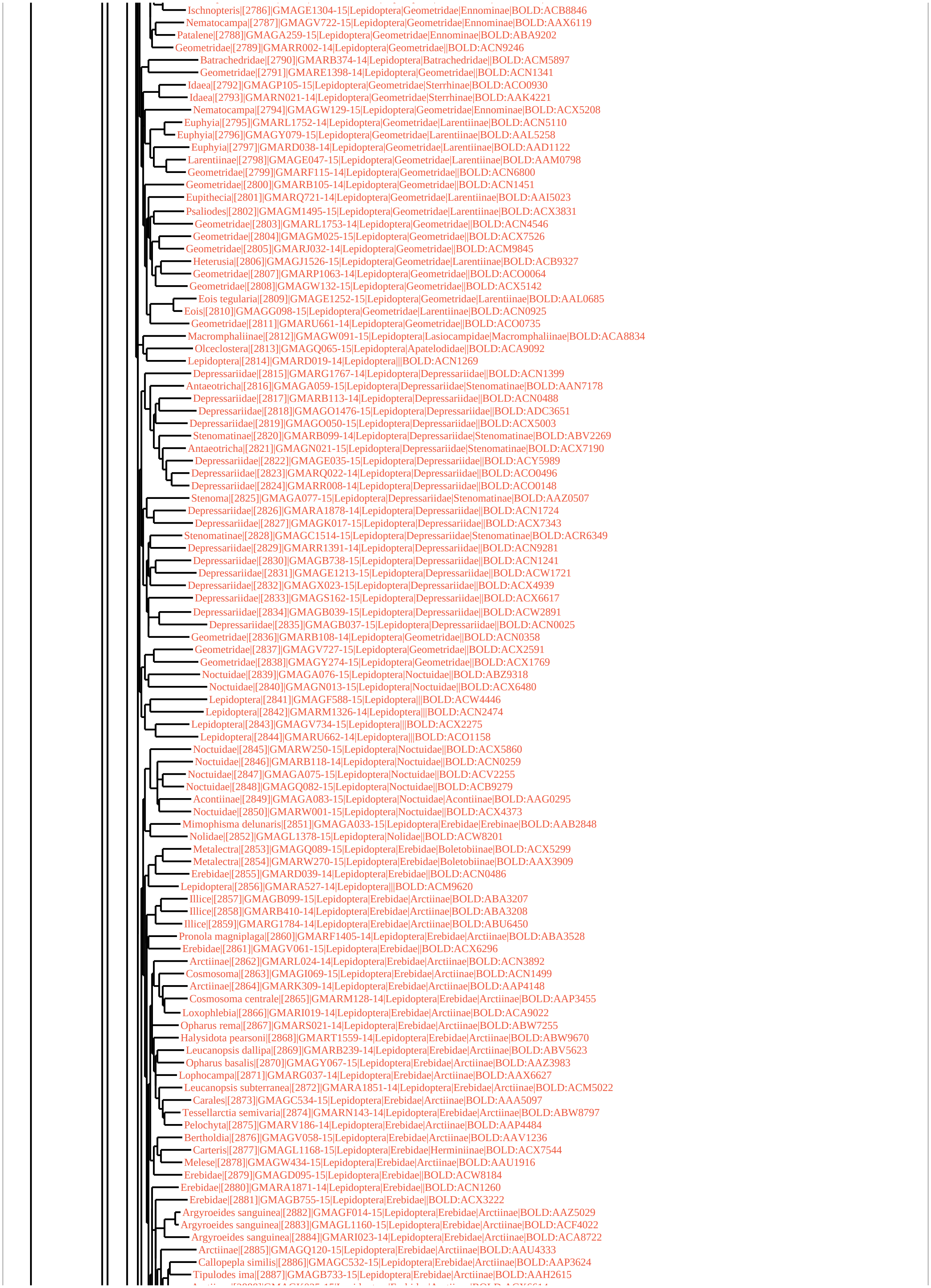
Ischnopteris|[2786]|GMAGE1304-15|Lepidoptera|Geometridae|Ennominae|BOLD:ACB8846
Nematocampa|[2787]|GMAGV722-15|Lepidoptera|Geometridae|Ennominae|BOLD:AAX6119
Patalene|[2788]|GMAGA259-15|Lepidoptera|Geometridae|Ennominae|BOLD:ABA9202
Geometridae|[2789]|GMARR002-14|Lepidoptera|Geometridae||BOLD:ACN9246
Batrachedridae|[2790]|GMARB374-14|Lepidoptera|Batrachedridae||BOLD:ACM5897
Geometridae|[2791]|GMARE1398-14|Lepidoptera|Geometridae||BOLD:ACN1341
Idaea|[2792]|GMAGP105-15|Lepidoptera|Geometridae|Sterrhinae|BOLD:ACO0930
Idaea|[2793]|GMARN021-14|Lepidoptera|Geometridae|Sterrhinae|BOLD:AAK4221
Nematocampa|[2794]|GMAGW129-15|Lepidoptera|Geometridae|Ennominae|BOLD:ACX5208
Euphyia|[2795]|GMARL1752-14|Lepidoptera|Geometridae|Larentiinae|BOLD:ACN5110
Euphyia|[2796]|GMAGY079-15|Lepidoptera|Geometridae|Larentiinae|BOLD:AAL5258
Euphyia|[2797]|GMARD038-14|Lepidoptera|Geometridae|Larentiinae|BOLD:AAD1122
Larentiinae|[2798]|GMAGE047-15|Lepidoptera|Geometridae|Larentiinae|BOLD:AAM0798
Geometridae|[2799]|GMARF115-14|Lepidoptera|Geometridae||BOLD:ACN6800
Geometridae|[2800]|GMARB105-14|Lepidoptera|Geometridae||BOLD:ACN1451
Eupithecia|[2801]|GMARQ721-14|Lepidoptera|Geometridae|Larentiinae|BOLD:AAI5023
Psaliodes|[2802]|GMAGM1495-15|Lepidoptera|Geometridae|Larentiinae|BOLD:ACX3831
Geometridae|[2803]|GMARL1753-14|Lepidoptera|Geometridae||BOLD:ACN4546
Geometridae|[2804]|GMAGM025-15|Lepidoptera|Geometridae||BOLD:ACX7526
Geometridae|[2805]|GMARJ032-14|Lepidoptera|Geometridae||BOLD:ACM9845
Heterusia|[2806]|GMAGJ1526-15|Lepidoptera|Geometridae|Larentiinae|BOLD:ACB9327
Geometridae|[2807]|GMARP1063-14|Lepidoptera|Geometridae||BOLD:ACO0064
Geometridae|[2808]|GMAGW132-15|Lepidoptera|Geometridae||BOLD:ACX5142
Eois tegularia|[2809]|GMAGE1252-15|Lepidoptera|Geometridae|Larentiinae|BOLD:AAL0685
Eois|[2810]|GMAGG098-15|Lepidoptera|Geometridae|Larentiinae|BOLD:ACN0925
Geometridae|[2811]|GMARU661-14|Lepidoptera|Geometridae||BOLD:ACO0735
Macromphaliinae|[2812]|GMAGW091-15|Lepidoptera|Lasiocampidae|Macromphaliinae|BOLD:ACA8834
Olceclostera|[2813]|GMAGQ065-15|Lepidoptera|Apatelodidae||BOLD:ACA9092
Lepidoptera|[2814]|GMARD019-14|Lepidoptera|||BOLD:ACN1269
Depressariidae|[2815]|GMARG1767-14|Lepidoptera|Depressariidae||BOLD:ACN1399
Antaeotricha|[2816]|GMAGA059-15|Lepidoptera|Depressariidae|Stenomatinae|BOLD:AAN7178
Depressariidae|[2817]|GMARB113-14|Lepidoptera|Depressariidae||BOLD:ACN0488
Depressariidae|[2818]|GMAGO1476-15|Lepidoptera|Depressariidae||BOLD:ADC3651
Depressariidae|[2819]|GMAGO050-15|Lepidoptera|Depressariidae||BOLD:ACX5003
Stenomatinae|[2820]|GMARB099-14|Lepidoptera|Depressariidae|Stenomatinae|BOLD:ABV2269
Antaeotricha|[2821]|GMAGN021-15|Lepidoptera|Depressariidae|Stenomatinae|BOLD:ACX7190
Depressariidae|[2822]|GMAGE035-15|Lepidoptera|Depressariidae||BOLD:ACY5989
Depressariidae|[2823]|GMARQ022-14|Lepidoptera|Depressariidae||BOLD:ACO0496
Depressariidae|[2824]|GMARR008-14|Lepidoptera|Depressariidae||BOLD:ACO0148
Stenoma|[2825]|GMAGA077-15|Lepidoptera|Depressariidae|Stenomatinae|BOLD:AAZ0507
Depressariidae|[2826]|GMARA1878-14|Lepidoptera|Depressariidae||BOLD:ACN1724
Depressariidae|[2827]|GMAGK017-15|Lepidoptera|Depressariidae||BOLD:ACX7343
Stenomatinae|[2828]|GMAGC1514-15|Lepidoptera|Depressariidae|Stenomatinae|BOLD:ACR6349
Depressariidae|[2829]|GMARR1391-14|Lepidoptera|Depressariidae||BOLD:ACN9281
Depressariidae|[2830]|GMAGB738-15|Lepidoptera|Depressariidae||BOLD:ACN1241
Depressariidae|[2831]|GMAGE1213-15|Lepidoptera|Depressariidae||BOLD:ACW1721
Depressariidae|[2832]|GMAGX023-15|Lepidoptera|Depressariidae||BOLD:ACX4939
Depressariidae|[2833]|GMAGS162-15|Lepidoptera|Depressariidae||BOLD:ACX6617
Depressariidae|[2834]|GMAGB039-15|Lepidoptera|Depressariidae||BOLD:ACW2891
Depressariidae|[2835]|GMAGB037-15|Lepidoptera|Depressariidae||BOLD:ACN0025
Geometridae|[2836]|GMARB108-14|Lepidoptera|Geometridae||BOLD:ACN0358
Geometridae|[2837]|GMAGV727-15|Lepidoptera|Geometridae||BOLD:ACX2591
Geometridae|[2838]|GMAGY274-15|Lepidoptera|Geometridae||BOLD:ACX1769
Noctuidae|[2839]|GMAGA076-15|Lepidoptera|Noctuidae||BOLD:ABZ9318
Noctuidae|[2840]|GMAGN013-15|Lepidoptera|Noctuidae||BOLD:ACX6480
Lepidoptera|[2841]|GMAGF588-15|Lepidoptera|||BOLD:ACW4446
Lepidoptera|[2842]|GMARM1326-14|Lepidoptera|||BOLD:ACN2474
Lepidoptera|[2843]|GMAGV734-15|Lepidoptera|||BOLD:ACX2275
Lepidoptera|[2844]|GMARU662-14|Lepidoptera|||BOLD:ACO1158
Noctuidae|[2845]|GMARW250-15|Lepidoptera|Noctuidae||BOLD:ACX5860
Noctuidae|[2846]|GMARB118-14|Lepidoptera|Noctuidae||BOLD:ACN0259
Noctuidae|[2847]|GMAGA075-15|Lepidoptera|Noctuidae||BOLD:ACV2255
Noctuidae|[2848]|GMAGQ082-15|Lepidoptera|Noctuidae||BOLD:ACB9279
Acontiinae|[2849]|GMAGA083-15|Lepidoptera|Noctuidae|Acontiinae|BOLD:AAG0295
Noctuidae|[2850]|GMARW001-15|Lepidoptera|Noctuidae||BOLD:ACX4373
Mimophisma delunaris|[2851]|GMAGA033-15|Lepidoptera|Erebidae|Erebinae|BOLD:AAB2848
Nolidae|[2852]|GMAGL1378-15|Lepidoptera|Nolidae||BOLD:ACW8201
Metalectra|[2853]|GMAGQ089-15|Lepidoptera|Erebidae|Boletobiinae|BOLD:ACX5299
Metalectra|[2854]|GMARW270-15|Lepidoptera|Erebidae|Boletobiinae|BOLD:AAX3909
Erebidae|[2855]|GMARD039-14|Lepidoptera|Erebidae||BOLD:ACN0486
Lepidoptera|[2856]|GMARA527-14|Lepidoptera|||BOLD:ACM9620
Illice|[2857]|GMAGB099-15|Lepidoptera|Erebidae|Arctiinae|BOLD:ABA3207
Illice|[2858]|GMARB410-14|Lepidoptera|Erebidae|Arctiinae|BOLD:ABA3208
Illice|[2859]|GMARG1784-14|Lepidoptera|Erebidae|Arctiinae|BOLD:ABU6450
Pronola magniplaga|[2860]|GMARF1405-14|Lepidoptera|Erebidae|Arctiinae|BOLD:ABA3528
Erebidae|[2861]|GMAGV061-15|Lepidoptera|Erebidae||BOLD:ACX6296
Arctiinae|[2862]|GMARL024-14|Lepidoptera|Erebidae|Arctiinae|BOLD:ACN3892
Cosmosoma|[2863]|GMAGI069-15|Lepidoptera|Erebidae|Arctiinae|BOLD:ACN1499
Arctiinae|[2864]|GMARK309-14|Lepidoptera|Erebidae|Arctiinae|BOLD:AAP4148
Cosmosoma centrale|[2865]|GMARM128-14|Lepidoptera|Erebidae|Arctiinae|BOLD:AAP3455
Loxophlebia|[2866]|GMARI019-14|Lepidoptera|Erebidae|Arctiinae|BOLD:ACA9022
Opharus rema|[2867]|GMARS021-14|Lepidoptera|Erebidae|Arctiinae|BOLD:ABW7255
Halysidota pearsoni|[2868]|GMART1559-14|Lepidoptera|Erebidae|Arctiinae|BOLD:ABW9670
Leucanopsis dallipa|[2869]|GMARB239-14|Lepidoptera|Erebidae|Arctiinae|BOLD:ABV5623
Opharus basalis|[2870]|GMAGY067-15|Lepidoptera|Erebidae|Arctiinae|BOLD:AAZ3983
Lophocampa|[2871]|GMARG037-14|Lepidoptera|Erebidae|Arctiinae|BOLD:AAX6627
Leucanopsis subterranea|[2872]|GMARA1851-14|Lepidoptera|Erebidae|Arctiinae|BOLD:ACM5022
Carales|[2873]|GMAGC534-15|Lepidoptera|Erebidae|Arctiinae|BOLD:AAA5097
Tessellarctia semivaria|[2874]|GMARN143-14|Lepidoptera|Erebidae|Arctiinae|BOLD:ABW8797
Pelochyta|[2875]|GMARV186-14|Lepidoptera|Erebidae|Arctiinae|BOLD:AAP4484
Bertholdia|[2876]|GMAGV058-15|Lepidoptera|Erebidae|Arctiinae|BOLD:AAV1236
Carteris|[2877]|GMAGL1168-15|Lepidoptera|Erebidae|Herminiinae|BOLD:ACX7544
Melese|[2878]|GMAGW434-15|Lepidoptera|Erebidae|Arctiinae|BOLD:AAU1916
Erebidae|[2879]|GMAGD095-15|Lepidoptera|Erebidae||BOLD:ACW8184
Erebidae|[2880]|GMARA1871-14|Lepidoptera|Erebidae||BOLD:ACN1260
Erebidae|[2881]|GMAGB755-15|Lepidoptera|Erebidae||BOLD:ACX3222
Argyroeides sanguinea|[2882]|GMAGF014-15|Lepidoptera|Erebidae|Arctiinae|BOLD:AAZ5029
Argyroeides sanguinea|[2883]|GMAGL1160-15|Lepidoptera|Erebidae|Arctiinae|BOLD:ACF4022
Argyroeides sanguinea|[2884]|GMARI023-14|Lepidoptera|Erebidae|Arctiinae|BOLD:ACA8722
Arctiinae|[2885]|GMAGQ120-15|Lepidoptera|Erebidae|Arctiinae|BOLD:AAU4333
Callopepla similis|[2886]|GMAGC532-15|Lepidoptera|Erebidae|Arctiinae|BOLD:AAP3624
Tipulodes ima|[2887]|GMAGB733-15|Lepidoptera|Erebidae|Arctiinae|BOLD:AAH2615

Arctiinae|[2885]|GMAGQ120-15|Lepidoptera|Erebidae|Arctiinae|BOLD:AAU4333
Callopepla similis|[2886]|GMAGC532-15|Lepidoptera|Erebidae|Arctiinae|BOLD:AAP3624
Tipulodes ima|[2887]|GMAGB733-15|Lepidoptera|Erebidae|Arctiinae|BOLD:AAH2615
Arctiinae|[2888]|GMAGK025-15|Lepidoptera|Erebidae|Arctiinae|BOLD:ACX6614
Arctiinae|[2889]|GMARN1988-14|Lepidoptera|Erebidae|Arctiinae|BOLD:ACA8806
Erebidae|[2890]|GMAGC529-15|Lepidoptera|Erebidae||BOLD:ACX1446
Gonodonta|[2891]|GMARH005-14|Lepidoptera|Erebidae|Calpinae|BOLD:AAA4308
Erebidae|[2892]|GMAGY069-15|Lepidoptera|Erebidae||BOLD:AAD1849
Lepidoptera|[2893]|GMAGJ1533-15|Lepidoptera|||BOLD:ACX5919
Renia|[2894]|GMAGU013-15|Lepidoptera|Erebidae|Herminiinae|BOLD:ACZ0598
Herminiinae|[2895]|GMAGY014-15|Lepidoptera|Erebidae|Herminiinae|BOLD:ACO1434
Mastixis|[2896]|GMARL012-14|Lepidoptera|Erebidae|Herminiinae|BOLD:ACB9448
Erebidae|[2897]|GMARK001-14|Lepidoptera|Erebidae||BOLD:ACN5919
Salia|[2898]|GMARL1755-14|Lepidoptera|Erebidae|Herminiinae|BOLD:ACB8890
Herminiinae|[2899]|GMARE1402-14|Lepidoptera|Erebidae|Herminiinae|BOLD:ACN3253
Erebidae|[2900]|GMAGM023-15|Lepidoptera|Erebidae||BOLD:ACO1080
Erebidae|[2901]|GMARM019-14|Lepidoptera|Erebidae||BOLD:ACN4783
Phalaenophana|[2902]|GMARC102-14|Lepidoptera|Erebidae|Herminiinae|BOLD:AAY2639
Erebidae|[2903]|GMARM015-14|Lepidoptera|Erebidae||BOLD:ACN5694
Erebidae|[2904]|GMARM012-14|Lepidoptera|Erebidae||BOLD:ACN5181
Erebidae|[2905]|GMAGB097-15|Lepidoptera|Erebidae||BOLD:ACX5333
Erebidae|[2906]|GMARR018-14|Lepidoptera|Erebidae||BOLD:ACN9289
Herminiinae|[2907]|GMART026-14|Lepidoptera|Erebidae|Herminiinae|BOLD:ACB9302
Herminiinae|[2908]|GMAGB739-15|Lepidoptera|Erebidae|Herminiinae|BOLD:ACB9096
Lascoria|[2909]|GMAGA079-15|Lepidoptera|Erebidae|Herminiinae|BOLD:ACN4883
Lascoria anxa|[2910]|GMARL009-14|Lepidoptera|Erebidae|Herminiinae|BOLD:ACB9002
Lascoria|[2911]|GMARI018-14|Lepidoptera|Erebidae|Herminiinae|BOLD:ACN6895
Lascoria|[2912]|GMARB408-14|Lepidoptera|Erebidae|Herminiinae|BOLD:AAQ3408
Erebidae|[2913]|GMAGH022-15|Lepidoptera|Erebidae||BOLD:ACN6788
Bleptina|[2914]|GMARM130-14|Lepidoptera|Erebidae|Herminiinae|BOLD:ABA8517
Herminiinae|[2915]|GMARA1865-14|Lepidoptera|Erebidae|Herminiinae|BOLD:ACN0262
Herminiinae|[2916]|GMAGQ070-15|Lepidoptera|Erebidae|Herminiinae|BOLD:ACO0916
Erebidae|[2917]|GMARM007-14|Lepidoptera|Erebidae||BOLD:ACN6290
Erebidae|[2918]|GMAGY070-15|Lepidoptera|Erebidae||BOLD:ACX4587
Erebidae|[2919]|GMAGK023-15|Lepidoptera|Erebidae||BOLD:ACN4205
Erebidae|[2920]|GMARG007-14|Lepidoptera|Erebidae||BOLD:ACN6291
Lepidoptera|[2921]|GMAGO1473-15|Lepidoptera|||BOLD:ACN3126
Zale janisca|[2922]|GMAGE026-15|Lepidoptera|Erebidae|Erebinae|BOLD:ACA8701
Zale exhausta|[2923]|GMAGA022-15|Lepidoptera|Erebidae|Erebinae|BOLD:AAC7176
Zale|[2924]|GMAGA028-15|Lepidoptera|Erebidae|Erebinae|BOLD:AAN0254
Elachistidae|[2925]|GMARA461-14|Lepidoptera|Elachistidae||BOLD:ACM9036
Elachistidae|[2926]|GMAGD1569-15|Lepidoptera|Elachistidae||BOLD:ACN0264
Elachistidae|[2927]|GMAGE1276-15|Lepidoptera|Elachistidae||BOLD:ACM4779
Elachistidae|[2928]|GMAGE1297-15|Lepidoptera|Elachistidae||BOLD:ACN0366
Elachistidae|[2929]|GMAGE1281-15|Lepidoptera|Elachistidae||BOLD:ACN1135
Latebraria amphipyroides|[2930]|GMARV137-14|Lepidoptera|Erebidae|Erebinae|BOLD:AAC1011
Elachistidae|[2931]|GMAGJ1425-15|Lepidoptera|Elachistidae||BOLD:ACN0622
Elachistidae|[2932]|GMARA519-14|Lepidoptera|Elachistidae||BOLD:ACM9431
Elachistidae|[2933]|GMAGL1384-15|Lepidoptera|Elachistidae||BOLD:ACN8921
Elachistidae|[2934]|GMARQ696-14|Lepidoptera|Elachistidae||BOLD:ACN8027
Lepidoptera|[2935]|GMAGE1231-15|Lepidoptera|||BOLD:ACW1858
Erebidae|[2936]|GMAGJ1461-15|Lepidoptera|Erebidae||BOLD:ACN9184
Erebidae|[2937]|GMAGW008-15|Lepidoptera|Erebidae||BOLD:AAU4334
Lycomorphodes strigosa|[2938]|GMAGA082-15|Lepidoptera|Erebidae|Arctiinae|BOLD:ABU7217
Erebidae|[2939]|GMARA518-14|Lepidoptera|Erebidae||BOLD:ACM8879
Erebidae|[2940]|GMAGW425-15|Lepidoptera|Erebidae||BOLD:ACX2965
Erebidae|[2941]|GMARP1003-14|Lepidoptera|Erebidae||BOLD:ACB9656
Lepidoptera|[2942]|GMAGT944-15|Lepidoptera|||BOLD:ACX0786
Heterochroma sarepta|[2943]|GMARO172-14|Lepidoptera|Noctuidae|Noctuinae|BOLD:ACX6371
Eriopinae|[2944]|GMAGH020-15|Lepidoptera|Noctuidae|Eriopinae|BOLD:ACB9429
Lacinipolia|[2945]|GMAGQ077-15|Lepidoptera|Noctuidae|Noctuinae|BOLD:ACX7344
Erebidae|[2946]|GMARB111-14|Lepidoptera|Erebidae||BOLD:ACN0532
Elaphria deltoides|[2947]|GMAGQ091-15|Lepidoptera|Noctuidae|Noctuinae|BOLD:ACE4341
Schidax squammularia|[2948]|GMARH001-14|Lepidoptera|Uraniidae|Epipleminae|BOLD:AAY7963
Anticarsia gemmatalis|[2949]|GMAGA025-15|Lepidoptera|Erebidae|Eulepidotinae|BOLD:AAA6923
Catocalinae|[2950]|GMARP093-14|Lepidoptera|Erebidae|Catocalinae|BOLD:ACB9657
Cropia cedica|[2951]|GMAGD086-15|Lepidoptera|Noctuidae|Amphipyrinae|BOLD:ACX0126
Condica|[2952]|GMART1687-14|Lepidoptera|Noctuidae|Condicinae|BOLD:ACB9281
Antiblemma|[2953]|GMARA1867-14|Lepidoptera|Erebidae|Eulepidotinae|BOLD:ABY4140
Erebidae|[2954]|GMARV183-14|Lepidoptera|Erebidae||BOLD:ACO0694
Noctuidae|[2955]|GMARA972-14|Lepidoptera|Noctuidae||BOLD:ACN0009
Noctuidae|[2956]|GMAGE046-15|Lepidoptera|Noctuidae||BOLD:ACN9611
Noctuidae|[2957]|GMAGE043-15|Lepidoptera|Noctuidae||BOLD:ACY6156
Ctenoplusia oxygramma|[2958]|GMARR1390-14|Lepidoptera|Noctuidae|Plusiinae|BOLD:AAA9797
Radosa ordinata|[2959]|GMAGA048-15|Lepidoptera|Erebidae|Calpinae|BOLD:ACB9165
Tiracola grandirena|[2960]|GMAGT022-15|Lepidoptera|Noctuidae|Noctuinae|BOLD:AAB9499
Noctuidae|[2961]|GMARN1994-14|Lepidoptera|Noctuidae||BOLD:ACO1311
Hampsonodes|[2962]|GMAGJ1462-15|Lepidoptera|Noctuidae|Noctuinae|BOLD:AAU5805
Noctuidae|[2963]|GMAGA252-15|Lepidoptera|Noctuidae||BOLD:ACV0807
Gonodes liquida|[2964]|GMAGH018-15|Lepidoptera|Noctuidae|Noctuinae|BOLD:AAA8110
Hampsonodes|[2965]|GMARP1008-14|Lepidoptera|Noctuidae|Noctuinae|BOLD:ACN7661
Noctuidae|[2966]|GMARP1019-14|Lepidoptera|Noctuidae||BOLD:ACN7439
Elaphria|[2967]|GMAGH922-15|Lepidoptera|Noctuidae|Noctuinae|BOLD:AAB2202
Elaphria subobliqua|[2968]|GMAGL1164-15|Lepidoptera|Noctuidae|Noctuinae|BOLD:AAB0341
Elaphria subobliqua|[2969]|GMAGM017-15|Lepidoptera|Noctuidae|Noctuinae|BOLD:AAB0340
Noctuidae|[2970]|GMAGO049-15|Lepidoptera|Noctuidae||BOLD:ACX5504
Bryolymnia|[2971]|GMAGS160-15|Lepidoptera|Noctuidae|Noctuinae|BOLD:AAP3969
Noctuidae|[2972]|GMARQ026-14|Lepidoptera|Noctuidae||BOLD:ACO0118
Leucania|[2973]|GMART119-14|Lepidoptera|Noctuidae|Noctuinae|BOLD:ACB9505
Spodoptera dolichos|[2974]|GMARS867-14|Lepidoptera|Noctuidae|Noctuinae|BOLD:ABY5226
Spodoptera eridania|[2975]|GMAGQ071-15|Lepidoptera|Noctuidae|Noctuinae|BOLD:AAA6521
Condica mobilis|[2976]|GMAGE027-15|Lepidoptera|Noctuidae|Condicinae|BOLD:AAB3357
Condica cupentia|[2977]|GMARH002-14|Lepidoptera|Noctuidae|Condicinae|BOLD:AAA7985
Condica|[2978]|GMAGA040-15|Lepidoptera|Noctuidae|Condicinae|BOLD:ACF3396
Condica|[2979]|GMARG008-14|Lepidoptera|Noctuidae|Condicinae|BOLD:AAC2619
Trachea|[2980]|GMARO173-14|Lepidoptera|Noctuidae|Noctuinae|BOLD:ACN9825
Noctuidae|[2981]|GMAGE029-15|Lepidoptera|Noctuidae||BOLD:ACN5664
Noctuidae|[2982]|GMAGQ078-15|Lepidoptera|Noctuidae||BOLD:ACN4207
Noctuidae|[2983]|GMARQ094-14|Lepidoptera|Noctuidae||BOLD:AAB1969
Chabuata|[2984]|GMAGC536-15|Lepidoptera|Noctuidae|Noctuinae|BOLD:AAN1461
Lacinipolia|[2985]|GMARD040-14|Lepidoptera|Noctuidae|Noctuinae|BOLD:ACN1309
Eriopyga dotata|[2986]|GMARK2339-14|Lepidoptera|Noctuidae|Noctuinae|BOLD:ABU6611
Noctuidae|[2987]|GMARP087-14|Lepidoptera|Noctuidae||BOLD:ACO0145

Lacinipolia|[2985]|GMARD040-14|Lepidoptera|Noctuidae|Noctuinae|BOLD:ACN1309
Eriopyga dotata|[2986]|GMARK2339-14|Lepidoptera|Noctuidae|Noctuinae|BOLD:ABU6611
Noctuidae|[2987]|GMARP087-14|Lepidoptera|Noctuidae||BOLD:ACO0145
Noctuinae|[2988]|GMAGM011-15|Lepidoptera|Noctuidae|Noctuinae|BOLD:ACX7354
Lacinipolia|[2989]|GMAGL1163-15|Lepidoptera|Noctuidae|Noctuinae|BOLD:ACN3851
Noctuidae|[2990]|GMARQ092-14|Lepidoptera|Noctuidae||BOLD:ACN9678
Erebidae|[2991]|GMAGN420-15|Lepidoptera|Erebidae||BOLD:ACX5615
Erebidae|[2992]|GMAGQ552-15|Lepidoptera|Erebidae||BOLD:ACX6005
Radara nealcesalis|[2993]|GMAGC543-15|Lepidoptera|Erebidae|Catocalinae|BOLD:ABA2787
Erebidae|[2994]|GMARU208-14|Lepidoptera|Erebidae||BOLD:ACO1536
Hypena|[2995]|GMAGA071-15|Lepidoptera|Erebidae|Hypeninae|BOLD:ACV2776
Hypena mactatalis|[2996]|GMAGA053-15|Lepidoptera|Erebidae|Hypeninae|BOLD:ACB8793
Hypena|[2997]|GMAGA055-15|Lepidoptera|Erebidae|Hypeninae|BOLD:ACV1934
Phyprosopus ergodan|[2998]|GMAGF021-15|Lepidoptera|Erebidae|Eulepidotinae|BOLD:AAQ4032
Hypena|[2999]|GMAGA074-15|Lepidoptera|Erebidae|Hypeninae|BOLD:AAB3497
Perasia|[3000]|GMAGQ069-15|Lepidoptera|Erebidae|Catocalinae|BOLD:AAG5906
Zale pachystrigata|[3001]|GMAGA030-15|Lepidoptera|Erebidae|Erebinae|BOLD:AAB1417
Zale|[3002]|GMAGA023-15|Lepidoptera|Erebidae|Erebinae|BOLD:AAB1416
Smyra axonia|[3003]|GMAGA051-15|Lepidoptera|Erebidae|Calpinae|BOLD:ACF3452
Peteroma|[3004]|GMARR120-14|Lepidoptera|Erebidae|Catocalinae|BOLD:ACO0260
Pseudyrias|[3005]|GMAGY068-15|Lepidoptera|Erebidae|Calpinae|BOLD:ACY8114
Erebidae|[3006]|GMAGA031-15|Lepidoptera|Erebidae||BOLD:ABY9281
Proroblemma|[3007]|GMAGH923-15|Lepidoptera|Erebidae|Boletobiinae|BOLD:ACN1099
Lepidoptera|[3008]|GMAGQ557-15|Lepidoptera|||BOLD:ACO1309
Catocalinae|[3009]|GMAGD099-15|Lepidoptera|Erebidae|Catocalinae|BOLD:ACB8866
Anomis hemiscopis|[3010]|GMAGQ076-15|Lepidoptera|Erebidae|Scoliopteryginae|BOLD:AAJ2781
Erebidae|[3011]|GMAGD089-15|Lepidoptera|Erebidae||BOLD:ACW8286
Erebidae|[3012]|GMARJ1839-14|Lepidoptera|Erebidae||BOLD:ACM9902
Artace cribraria|[3013]|GMARR016-14|Lepidoptera|Lasiocampidae|Macromphaliinae|BOLD:ACA8943
Erebidae|[3014]|GMARN1991-14|Lepidoptera|Erebidae||BOLD:ACN8991
Coenipeta|[3015]|GMAGA024-15|Lepidoptera|Erebidae|Erebinae|BOLD:ACV3110
Erebidae|[3016]|GMAGQ537-15|Lepidoptera|Erebidae||BOLD:ACX5685
Calledema sura|[3017]|GMAGJ1523-15|Lepidoptera|Notodontidae|Nystaleinae|BOLD:AAN0889
Arctiinae|[3018]|GMARG006-14|Lepidoptera|Erebidae|Arctiinae|BOLD:AAN1616
Erebidae|[3019]|GMARL1614-14|Lepidoptera|Erebidae||BOLD:ACN2748
Erebidae|[3020]|GMARM1264-14|Lepidoptera|Erebidae||BOLD:ACN2247
Lepidoptera|[3021]|GMAGI057-15|Lepidoptera|||BOLD:ACN2222
Lepidoptera|[3022]|GMAGI008-15|Lepidoptera|||BOLD:ACW8607
Lepidoptera|[3023]|GMARV143-14|Lepidoptera|||BOLD:ACO2140
Melese paranensis|[3024]|GMAGB098-15|Lepidoptera|Erebidae|Arctiinae|BOLD:AAG1070
Scopula|[3025]|GMAGI056-15|Lepidoptera|Geometridae|Sterrhinae|BOLD:AAA9026
Aenictes polygrapharia|[3026]|GMAGM020-15|Lepidoptera|Geometridae|Ennominae|BOLD:AAD8832
Arctiinae|[3027]|GMAGA182-15|Lepidoptera|Erebidae|Arctiinae|BOLD:ACE8772
Callionima|[3028]|GMAGA021-15|Lepidoptera|Sphingidae|Macroglossinae|BOLD:AAA6734
Pachylioides resumens|[3029]|GMAGA019-15|Lepidoptera|Sphingidae|Macroglossinae|BOLD:AAA4476
Perigonia|[3030]|GMARW242-15|Lepidoptera|Sphingidae|Macroglossinae|BOLD:ACE8070
Radosa|[3031]|GMAGG049-15|Lepidoptera|Erebidae|Calpinae|BOLD:ACA8835
Euteliidae|[3032]|GMAGE1227-15|Lepidoptera|Euteliidae||BOLD:ACN8627
Isogona|[3033]|GMARM117-14|Lepidoptera|Erebidae|Boletobiinae|BOLD:AAQ1666
Erebidae|[3034]|GMAGY289-15|Lepidoptera|Erebidae||BOLD:ACX1977
Lepidoptera|[3035]|GMAGH872-15|Lepidoptera|||BOLD:ACN8428
Lepidoptera|[3036]|GMARV175-14|Lepidoptera|||BOLD:ACO1114
Leurocephala schinusae|[3037]|GMAGH906-15|Lepidoptera|Gracillariidae|Gracillariinae|BOLD:AAD5995
Gracillariidae|[3038]|GMAGN495-15|Lepidoptera|Gracillariidae||BOLD:ACX4838
Gracillariidae|[3039]|GMAGD1566-15|Lepidoptera|Gracillariidae||BOLD:ACN2041
Gracillariidae|[3040]|GMARA449-14|Lepidoptera|Gracillariidae||BOLD:ACM9712
Gracillariidae|[3041]|GMAGB977-15|Lepidoptera|Gracillariidae||BOLD:ACM5914
Gracillariidae|[3042]|GMAGG135-15|Lepidoptera|Gracillariidae||BOLD:ACW3765
Gracillariidae|[3043]|GMAGM1503-15|Lepidoptera|Gracillariidae||BOLD:ACN8384
Caloptilia|[3044]|GMAGD1592-15|Lepidoptera|Gracillariidae|Gracillariinae|BOLD:ACN2219
Gracillariidae|[3045]|GMAGI034-15|Lepidoptera|Gracillariidae||BOLD:ACW5931
Gracillariidae|[3046]|GMAGE1218-15|Lepidoptera|Gracillariidae||BOLD:ACW1749
Gracillariidae|[3047]|GMARJ1842-14|Lepidoptera|Gracillariidae||BOLD:ACN1875
Gracillariidae|[3048]|GMAGC1255-15|Lepidoptera|Gracillariidae||BOLD:ACN0143
Gracillariidae|[3049]|GMAGP124-15|Lepidoptera|Gracillariidae||BOLD:ACX4865
Gracillariidae|[3050]|GMARA503-14|Lepidoptera|Gracillariidae||BOLD:ACM9032
Gracillariidae|[3051]|GMAGU738-15|Lepidoptera|Gracillariidae||BOLD:ACX5842
Porphyrosela|[3052]|GMAGF616-15|Lepidoptera|Gracillariidae|Lithocolletinae|BOLD:ACM5075
Gracillariidae|[3053]|GMAGB981-15|Lepidoptera|Gracillariidae||BOLD:ACN0010
Gracillariidae|[3054]|GMAGD1716-15|Lepidoptera|Gracillariidae||BOLD:ACW5222
Gracillariidae|[3055]|GMAGE1301-15|Lepidoptera|Gracillariidae||BOLD:ACW2515
Lithocolletinae|[3056]|GMAGL1430-15|Lepidoptera|Gracillariidae|Lithocolletinae|BOLD:ACN1933
Gracillariidae|[3057]|GMAGH905-15|Lepidoptera|Gracillariidae||BOLD:ACN0881
Gracillariidae|[3058]|GMARA521-14|Lepidoptera|Gracillariidae||BOLD:ACM9356
Gracillariidae|[3059]|GMAGO070-15|Lepidoptera|Gracillariidae||BOLD:ACX5002
Gracillariidae|[3060]|GMAGK2011-15|Lepidoptera|Gracillariidae||BOLD:ACW9932
Gracillariidae|[3061]|GMARB401-14|Lepidoptera|Gracillariidae||BOLD:ACM5101
Gracillariidae|[3062]|GMARI1541-14|Lepidoptera|Gracillariidae||BOLD:ACN0722
Gracillariidae|[3063]|GMARR800-14|Lepidoptera|Gracillariidae||BOLD:ACX3941
Gracillariidae|[3064]|GMAGA103-15|Lepidoptera|Gracillariidae||BOLD:ACN0982
Gracillariidae|[3065]|GMAGM1496-15|Lepidoptera|Gracillariidae||BOLD:ACX3548
Gracillariidae|[3066]|GMARB171-14|Lepidoptera|Gracillariidae||BOLD:ACM6315
Phyllocnistis|[3067]|GMAGV941-15|Lepidoptera|Gracillariidae|Phyllocnistinae|BOLD:ACG1885
Gracillariidae|[3068]|GMAGN516-15|Lepidoptera|Gracillariidae||BOLD:ACX6197
Lepidoptera|[3069]|GMAGB976-15|Lepidoptera|||BOLD:ACW6580
Erebidae|[3070]|GMARL1758-14|Lepidoptera|Erebidae||BOLD:ACN7203
Lepidoptera|[3071]|GMAGU1012-15|Lepidoptera|||BOLD:ACX5092
Heliozelidae|[3072]|GMAGD1714-15|Lepidoptera|Heliozelidae||BOLD:ACN1627
Heliozelidae|[3073]|GMAGN498-15|Lepidoptera|Heliozelidae||BOLD:ACN1697
Heliozelidae|[3074]|GMAGQ600-15|Lepidoptera|Heliozelidae||BOLD:ACN7732
Heliozelidae|[3075]|GMARO1172-14|Lepidoptera|Heliozelidae||BOLD:ACN8629
Ceromitia|[3076]|GMARA494-14|Lepidoptera|Adelidae|Nematopogoninae|BOLD:ACM4956
Geometridae|[3077]|GMAGC1234-15|Lepidoptera|Geometridae||BOLD:ACW0443
Geometridae|[3078]|GMARR817-14|Lepidoptera|Geometridae||BOLD:ACX3835
Lepidoptera|[3079]|GMARI1561-14|Lepidoptera|||BOLD:ACN1433
Tineidae|[3080]|GMAGU722-15|Lepidoptera|Tineidae||BOLD:ACN7558
Lepidoptera|[3081]|GMAGE1215-15|Lepidoptera|||BOLD:ACW1383
Tineidae|[3082]|GMARB383-14|Lepidoptera|Tineidae||BOLD:ACM6146
Tineidae|[3083]|GMARR1136-14|Lepidoptera|Tineidae||BOLD:ACN7750
Tineidae|[3084]|GMAGC1229-15|Lepidoptera|Tineidae||BOLD:ACM4863
Tineidae|[3085]|GMAGH877-15|Lepidoptera|Tineidae||BOLD:ACW3400
Tineidae|[3086]|GMARC854-14|Lepidoptera|Tineidae||BOLD:ACM4951
Tineidae|[3087]|GMARS796-14|Lepidoptera|Tineidae||BOLD:ACO0119

[illegible]
Tineidae|[3085]|GMAGH877-15|Lepidoptera|Tineidae||BOLD:ACW3400
Tineidae|[3086]|GMARC854-14|Lepidoptera|Tineidae||BOLD:ACM4951
Tineidae|[3087]|GMARS796-14|Lepidoptera|Tineidae||BOLD:ACO0119
Bedelliidae|[3088]|GMARQ710-14|Lepidoptera|Bedelliidae||BOLD:ACN8395
Lepidoptera|[3089]|GMAGA105-15|Lepidoptera|||BOLD:ACV2322
Erebidae|[3090]|GMAGS1149-15|Lepidoptera|Erebidae||BOLD:ACX4125
Lepidoptera|[3091]|GMAGJ1456-15|Lepidoptera|||BOLD:ACN0093
Lepidoptera|[3092]|GMARA453-14|Lepidoptera|||BOLD:ACM8857
Heliodinidae|[3093]|GMARM1265-14|Lepidoptera|Heliodinidae||BOLD:ACN1936
Lepidoptera|[3094]|GMAGU747-15|Lepidoptera|||BOLD:ACX5532
Tineidae|[3095]|GMAGA275-15|Lepidoptera|Tineidae||BOLD:ACN8113
Tineidae|[3096]|GMARA447-14|Lepidoptera|Tineidae||BOLD:ACM6141
Tineidae|[3097]|GMARA437-14|Lepidoptera|Tineidae||BOLD:ACM9430
Geometridae|[3098]|GMAGF610-15|Lepidoptera|Geometridae||BOLD:ACW3429
Geometridae|[3099]|GMARR809-14|Lepidoptera|Geometridae||BOLD:ACN7956
Geometridae|[3100]|GMARA411-14|Lepidoptera|Geometridae||BOLD:ACM8848
Geometridae|[3101]|GMARD2042-14|Lepidoptera|Geometridae||BOLD:ACN0721
Geometridae|[3102]|GMAGF599-15|Lepidoptera|Geometridae||BOLD:ACW3942
Geometridae|[3103]|GMAGL1382-15|Lepidoptera|Geometridae||BOLD:ACN2962
Geometridae|[3104]|GMARB368-14|Lepidoptera|Geometridae||BOLD:ACX3527
Geometridae|[3105]|GMAGY295-15|Lepidoptera|Geometridae||BOLD:ACX2554
Tineidae|[3106]|GMARD2031-14|Lepidoptera|Tineidae||BOLD:ACN0522
Tineidae|[3107]|GMAGV938-15|Lepidoptera|Tineidae||BOLD:ACX5300
Lepidoptera|[3108]|GMAGE2829-15|Lepidoptera|||BOLD:ACW7470
Lepidoptera|[3109]|GMAGM1493-15|Lepidoptera|||BOLD:ACX1896
Lyonetiidae|[3110]|GMARA500-14|Lepidoptera|Lyonetiidae||BOLD:ACM8860
Lepidoptera|[3111]|GMAGZ208-15|Lepidoptera|||BOLD:ACX3626
Orthoptera|[3112]|GMAGU1063-15|Orthoptera|||BOLD:ACX9790
Orthoptera|[3113]|GMARR1456-14|Orthoptera|||BOLD:ACN9779
Mantidae|[3114]|GMAGO007-15|Mantodea|Mantidae||BOLD:ACX7430
Acanthopidae|[3115]|GMAGA216-15|Mantodea|Acanthopidae||BOLD:ACW4073
Mantidae|[3116]|GMARN2062-14|Mantodea|Mantidae||BOLD:ACO1026
Mantoididae|[3117]|GMARQ765-14|Mantodea|Mantoididae||BOLD:ACN7426
Mantoididae|[3118]|GMARS859-14|Mantodea|Mantoididae||BOLD:ACN9841
Thespidae|[3119]|GMAGP086-15|Mantodea|Thespidae||BOLD:ACN9367
Orthoptera|[3120]|GMAGH1301-15|Orthoptera|||BOLD:ACW6026
Gryllidae|[3121]|GMAGB741-15|Orthoptera|Gryllidae||BOLD:ACN1197
Gryllidae|[3122]|GMAGG170-15|Orthoptera|Gryllidae||BOLD:ACW3868
Gryllidae|[3123]|GMAGL1557-15|Orthoptera|Gryllidae||BOLD:ACW9650
Gryllidae|[3124]|GMAGM1891-15|Orthoptera|Gryllidae||BOLD:ACN8286
Gryllidae|[3125]|GMAGA123-15|Orthoptera|Gryllidae||BOLD:ACN0146
Homeoura|[3126]|GMARJ059-14|Odonata|Coenagrionidae|Ischnurinae|BOLD:AAY5710
Gryllidae|[3127]|GMART1756-14|Orthoptera|Gryllidae||BOLD:ACO2157
Gryllidae|[3128]|GMAGA122-15|Orthoptera|Gryllidae||BOLD:ACN1191
Gryllidae|[3129]|GMARG1929-14|Orthoptera|Gryllidae||BOLD:ACN1031
Gryllidae|[3130]|GMAGA126-15|Orthoptera|Gryllidae||BOLD:ACN0806
Gryllidae|[3131]|GMARB141-14|Orthoptera|Gryllidae||BOLD:ACM5713
Gryllidae|[3132]|GMARE1386-14|Orthoptera|Gryllidae||BOLD:ACN0807
Gryllidae|[3133]|GMARR1457-14|Orthoptera|Gryllidae||BOLD:ACN9512
Gryllidae|[3134]|GMARD2107-14|Orthoptera|Gryllidae||BOLD:ACN9530
Gryllidae|[3135]|GMAGL1138-15|Orthoptera|Gryllidae||BOLD:AAU6107
Gryllidae|[3136]|GMAGL1159-15|Orthoptera|Gryllidae||BOLD:ACX6713
Orthoptera|[3137]|GMAGU998-15|Orthoptera|||BOLD:ACY3089
Blattodea|[3138]|GMARO1422-14|Blattodea|||BOLD:ACO1166
Termitidae|[3139]|GMAGO017-15|Isoptera|Termitidae||BOLD:ACX7010
Blattodea|[3140]|GMAGC531-15|Blattodea|||BOLD:ACN9718
Blattidae|[3141]|GMAGE1672-15|Blattodea|Blattidae||BOLD:ACN0196
Ectobiidae|[3142]|GMARF109-14|Blattodea|Ectobiidae||BOLD:ABX0173
Blattodea|[3143]|GMARQ833-14|Blattodea|||BOLD:ACO0292
Blattodea|[3144]|GMARS618-14|Blattodea|||BOLD:ACO1431
Blattodea|[3145]|GMARU794-14|Blattodea|||BOLD:ACX2863
Ectobiidae|[3146]|GMARA1933-14|Blattodea|Ectobiidae||BOLD:ACW2119
Blattidae|[3147]|GMARD2108-14|Blattodea|Blattidae||BOLD:ACN0792
Ectobiidae|[3148]|GMAGD2320-15|Blattodea|Ectobiidae||BOLD:ACN1098
Ectobiidae|[3149]|GMAGE055-15|Blattodea|Ectobiidae||BOLD:ACN0108
Epilamprinae|[3150]|GMARQ832-14|Blattodea|Blaberidae|Epilamprinae|BOLD:ACN9864
Ectobiidae|[3151]|GMAGI022-15|Blattodea|Ectobiidae||BOLD:ACW9402
Blattidae|[3152]|GMAGA178-15|Blattodea|Blattidae||BOLD:AAU6784
Blattodea|[3153]|GMARR1468-14|Blattodea|||BOLD:ACN9494
Blattodea|[3154]|GMARW274-15|Blattodea|||BOLD:ACX4944
Acrididae|[3155]|GMAGD069-15|Orthoptera|Acrididae||BOLD:ACO2158
Acrididae|[3156]|GMARO1417-14|Orthoptera|Acrididae||BOLD:ACX7181
Acrididae|[3157]|GMAGA155-15|Orthoptera|Acrididae||BOLD:ACN0853
Acrididae|[3158]|GMARP1260-14|Orthoptera|Acrididae||BOLD:AAU6101
Acrididae|[3159]|GMAGQ016-15|Orthoptera|Acrididae||BOLD:ACX6858
Acrididae|[3160]|GMARQ032-14|Orthoptera|Acrididae||BOLD:ACN9911
Ronderosia|[3161]|GMAGA151-15|Orthoptera|Acrididae|Melanoplinae|BOLD:ACN1128
Melanoplinae|[3162]|GMAGB004-15|Orthoptera|Acrididae|Melanoplinae|BOLD:ACW8117
Acrididae|[3163]|GMARA1883-14|Orthoptera|Acrididae||BOLD:ACN0137
Acrididae|[3164]|GMAGV993-15|Orthoptera|Acrididae||BOLD:ACO1678
Tetrigidae|[3165]|GMAGA116-15|Orthoptera|Tetrigidae||BOLD:ACN0623
Tetrigidae|[3166]|GMAGA168-15|Orthoptera|Tetrigidae||BOLD:ACM9976
Orthoptera|[3167]|GMARC124-14|Orthoptera|||BOLD:ACN0796
Pyrrhocoridae|[3168]|GMAGS002-15|Hemiptera|Pyrrhocoridae||BOLD:ACR6617
Miridae|[3169]|GMARA875-14|Hemiptera|Miridae||BOLD:ACM5931
Miridae|[3170]|GMAGE2732-15|Hemiptera|Miridae||BOLD:ACY7813
Miridae|[3171]|GMAGO014-15|Hemiptera|Miridae||BOLD:ABW3710
Miridae|[3172]|GMAGR071-15|Hemiptera|Miridae||BOLD:ACO0699
Pentatomidae|[3173]|GMARH097-14|Hemiptera|Pentatomidae||BOLD:ACN7397
Pentatomidae|[3174]|GMAGD001-15|Hemiptera|Pentatomidae||BOLD:ACN9295
Pentatomidae|[3175]|GMARK2576-14|Hemiptera|Pentatomidae||BOLD:ACY8406
Pentatomidae|[3176]|GMAGQ038-15|Hemiptera|Pentatomidae||BOLD:AAV0120
Pentatomidae|[3177]|GMAGQ039-15|Hemiptera|Pentatomidae||BOLD:ACN9158
Proxys albopunctulatus|[3178]|GMARE001-14|Hemiptera|Pentatomidae|Pentatominae|BOLD:ACB7013
Pentatomidae|[3179]|GMARS001-14|Hemiptera|Pentatomidae||BOLD:ACO2339
Reduviidae|[3180]|GMAGG011-15|Hemiptera|Reduviidae||BOLD:ACW9678
Reduviidae|[3181]|GMAGV181-15|Hemiptera|Reduviidae||BOLD:ACY2275
Reduviidae|[3182]|GMAGY094-15|Hemiptera|Reduviidae||BOLD:AAU4681
Lygaeidae|[3183]|GMAGC1087-15|Hemiptera|Lygaeidae||BOLD:ACO0423
Nysius|[3184]|GMAGP064-15|Hemiptera|Lygaeidae|Orsillinae|BOLD:ABA3751
Nysius raphanus|[3185]|GMAGW351-15|Hemiptera|Lygaeidae|Orsillinae|BOLD:ACE9798
Lygaeidae|[3186]|GMARL135-14|Hemiptera|Lygaeidae||BOLD:ABV3148

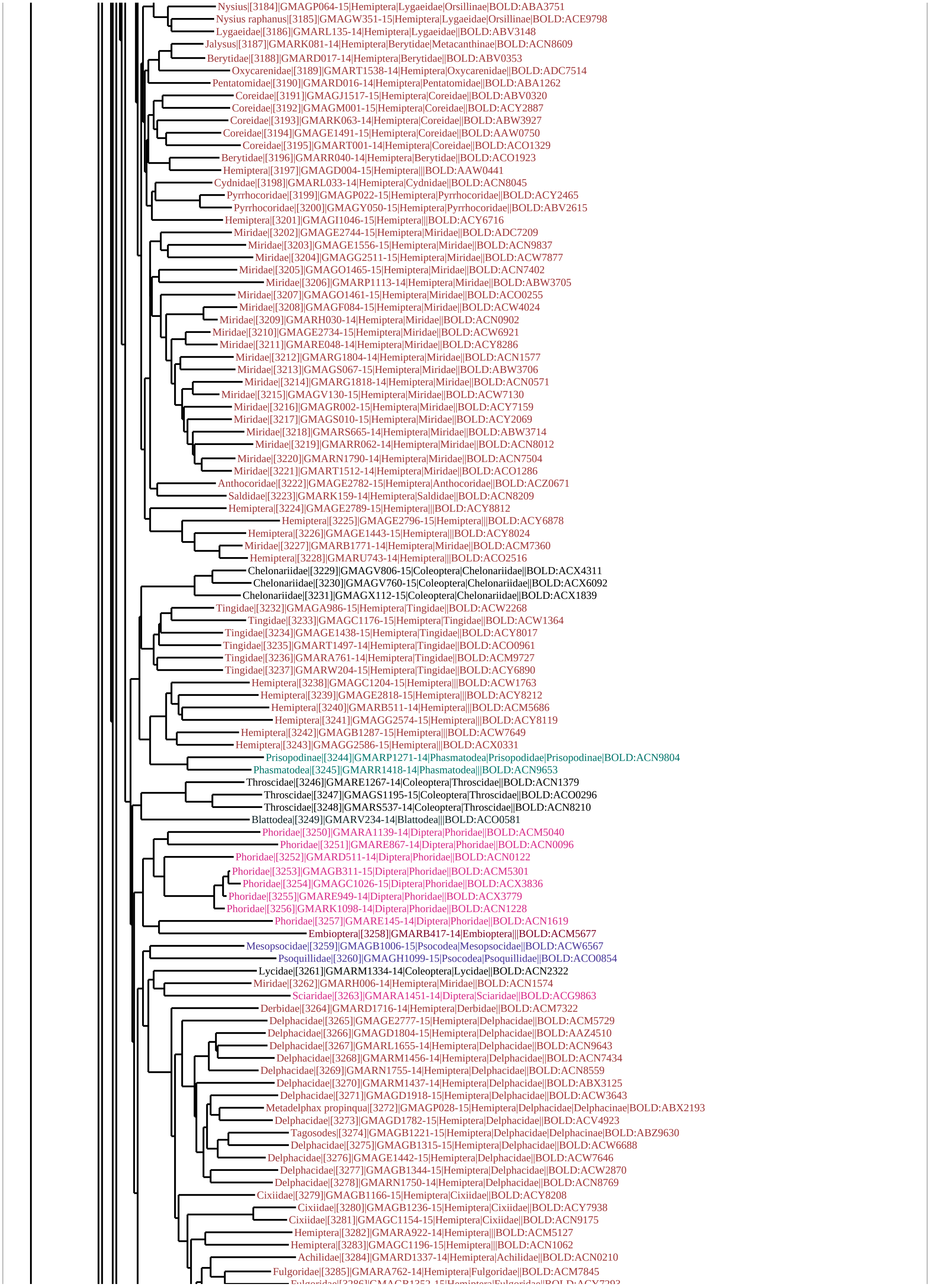
Nysius|[3184]|GMAGP064-15|Hemiptera|Lygaeidae|Orsillinae|BOLD:ABA3751
Nysius raphanus|[3185]|GMAGW351-15|Hemiptera|Lygaeidae|Orsillinae|BOLD:ACE9798
Lygaeidae|[3186]|GMARL135-14|Hemiptera|Lygaeidae||BOLD:ABV3148
Jalysus|[3187]|GMARK081-14|Hemiptera|Berytidae|Metacanthinae|BOLD:ACN8609
Berytidae|[3188]|GMARD017-14|Hemiptera|Berytidae||BOLD:ABV0353
Oxycarenidae|[3189]|GMART1538-14|Hemiptera|Oxycarenidae||BOLD:ADC7514
Pentatomidae|[3190]|GMARD016-14|Hemiptera|Pentatomidae||BOLD:ABA1262
Coreidae|[3191]|GMAGJ1517-15|Hemiptera|Coreidae||BOLD:ABV0320
Coreidae|[3192]|GMAGM001-15|Hemiptera|Coreidae||BOLD:ACY2887
Coreidae|[3193]|GMARK063-14|Hemiptera|Coreidae||BOLD:ABW3927
Coreidae|[3194]|GMAGE1491-15|Hemiptera|Coreidae||BOLD:AAW0750
Coreidae|[3195]|GMART001-14|Hemiptera|Coreidae||BOLD:ACO1329
Berytidae|[3196]|GMARR040-14|Hemiptera|Berytidae||BOLD:ACO1923
Hemiptera|[3197]|GMAGD004-15|Hemiptera|||BOLD:AAW0441
Cydnidae|[3198]|GMARL033-14|Hemiptera|Cydnidae||BOLD:ACN8045
Pyrrhocoridae|[3199]|GMAGP022-15|Hemiptera|Pyrrhocoridae||BOLD:ACY2465
Pyrrhocoridae|[3200]|GMAGY050-15|Hemiptera|Pyrrhocoridae||BOLD:ABV2615
Hemiptera|[3201]|GMAGI1046-15|Hemiptera|||BOLD:ACY6716
Miridae|[3202]|GMAGE2744-15|Hemiptera|Miridae||BOLD:ADC7209
Miridae|[3203]|GMAGE1556-15|Hemiptera|Miridae||BOLD:ACN9837
Miridae|[3204]|GMAGG2511-15|Hemiptera|Miridae||BOLD:ACW7877
Miridae|[3205]|GMAGO1465-15|Hemiptera|Miridae||BOLD:ACN7402
Miridae|[3206]|GMARP1113-14|Hemiptera|Miridae||BOLD:ABW3705
Miridae|[3207]|GMAGO1461-15|Hemiptera|Miridae||BOLD:ACO0255
Miridae|[3208]|GMAGF084-15|Hemiptera|Miridae||BOLD:ACW4024
Miridae|[3209]|GMARH030-14|Hemiptera|Miridae||BOLD:ACN0902
Miridae|[3210]|GMAGE2734-15|Hemiptera|Miridae||BOLD:ACW6921
Miridae|[3211]|GMARE048-14|Hemiptera|Miridae||BOLD:ACY8286
Miridae|[3212]|GMARG1804-14|Hemiptera|Miridae||BOLD:ACN1577
Miridae|[3213]|GMAGS067-15|Hemiptera|Miridae||BOLD:ABW3706
Miridae|[3214]|GMARG1818-14|Hemiptera|Miridae||BOLD:ACN0571
Miridae|[3215]|GMAGV130-15|Hemiptera|Miridae||BOLD:ACW7130
Miridae|[3216]|GMAGR002-15|Hemiptera|Miridae||BOLD:ACY7159
Miridae|[3217]|GMAGS010-15|Hemiptera|Miridae||BOLD:ACY2069
Miridae|[3218]|GMARS665-14|Hemiptera|Miridae||BOLD:ABW3714
Miridae|[3219]|GMARR062-14|Hemiptera|Miridae||BOLD:ACN8012
Miridae|[3220]|GMARN1790-14|Hemiptera|Miridae||BOLD:ACN7504
Miridae|[3221]|GMART1512-14|Hemiptera|Miridae||BOLD:ACO1286
Anthocoridae|[3222]|GMAGE2782-15|Hemiptera|Anthocoridae||BOLD:ACZ0671
Saldidae|[3223]|GMARK159-14|Hemiptera|Saldidae||BOLD:ACN8209
Hemiptera|[3224]|GMAGE2789-15|Hemiptera|||BOLD:ACY8812
Hemiptera|[3225]|GMAGE2796-15|Hemiptera|||BOLD:ACY6878
Hemiptera|[3226]|GMAGE1443-15|Hemiptera|||BOLD:ACY8024
Miridae|[3227]|GMARB1771-14|Hemiptera|Miridae||BOLD:ACM7360
Hemiptera|[3228]|GMARU743-14|Hemiptera|||BOLD:ACO2516
Chelonariidae|[3229]|GMAGV806-15|Coleoptera|Chelonariidae||BOLD:ACX4311
Chelonariidae|[3230]|GMAGV760-15|Coleoptera|Chelonariidae||BOLD:ACX6092
Chelonariidae|[3231]|GMAGX112-15|Coleoptera|Chelonariidae||BOLD:ACX1839
Tingidae|[3232]|GMAGA986-15|Hemiptera|Tingidae||BOLD:ACW2268
Tingidae|[3233]|GMAGC1176-15|Hemiptera|Tingidae||BOLD:ACW1364
Tingidae|[3234]|GMAGE1438-15|Hemiptera|Tingidae||BOLD:ACY8017
Tingidae|[3235]|GMART1497-14|Hemiptera|Tingidae||BOLD:ACO0961
Tingidae|[3236]|GMARA761-14|Hemiptera|Tingidae||BOLD:ACM9727
Tingidae|[3237]|GMARW204-15|Hemiptera|Tingidae||BOLD:ACY6890
Hemiptera|[3238]|GMAGC1204-15|Hemiptera|||BOLD:ACW1763
Hemiptera|[3239]|GMAGE2818-15|Hemiptera|||BOLD:ACY8212
Hemiptera|[3240]|GMARB511-14|Hemiptera|||BOLD:ACM5686
Hemiptera|[3241]|GMAGG2574-15|Hemiptera|||BOLD:ACY8119
Hemiptera|[3242]|GMAGB1287-15|Hemiptera|||BOLD:ACW7649
Hemiptera|[3243]|GMAGG2586-15|Hemiptera|||BOLD:ACX0331
Prisopodinae|[3244]|GMARP1271-14|Phasmatodea|Prisopodidae|Prisopodinae|BOLD:ACN9804
Phasmatodea|[3245]|GMARR1418-14|Phasmatodea|||BOLD:ACN9653
Throscidae|[3246]|GMARE1267-14|Coleoptera|Throscidae||BOLD:ACN1379
Throscidae|[3247]|GMAGS1195-15|Coleoptera|Throscidae||BOLD:ACO0296
Throscidae|[3248]|GMARS537-14|Coleoptera|Throscidae||BOLD:ACN8210
Blattodea|[3249]|GMARV234-14|Blattodea|||BOLD:ACO0581
Phoridae|[3250]|GMARA1139-14|Diptera|Phoridae||BOLD:ACM5040
Phoridae|[3251]|GMARE867-14|Diptera|Phoridae||BOLD:ACN0096
Phoridae|[3252]|GMARD511-14|Diptera|Phoridae||BOLD:ACN0122
Phoridae|[3253]|GMAGB311-15|Diptera|Phoridae||BOLD:ACM5301
Phoridae|[3254]|GMAGC1026-15|Diptera|Phoridae||BOLD:ACX3836
Phoridae|[3255]|GMARE949-14|Diptera|Phoridae||BOLD:ACX3779
Phoridae|[3256]|GMARK1098-14|Diptera|Phoridae||BOLD:ACN1228
Phoridae|[3257]|GMARE145-14|Diptera|Phoridae||BOLD:ACN1619
Embioptera|[3258]|GMARB417-14|Embioptera|||BOLD:ACM5677
Mesopsocidae|[3259]|GMAGB1006-15|Psocodea|Mesopsocidae||BOLD:ACW6567
Psoquillidae|[3260]|GMAGH1099-15|Psocodea|Psoquillidae||BOLD:ACO0854
Lycidae|[3261]|GMARM1334-14|Coleoptera|Lycidae||BOLD:ACN2322
Miridae|[3262]|GMARH006-14|Hemiptera|Miridae||BOLD:ACN1574
Sciaridae|[3263]|GMARA1451-14|Diptera|Sciaridae||BOLD:ACG9863
Derbidae|[3264]|GMARD1716-14|Hemiptera|Derbidae||BOLD:ACM7322
Delphacidae|[3265]|GMAGE2777-15|Hemiptera|Delphacidae||BOLD:ACM5729
Delphacidae|[3266]|GMAGD1804-15|Hemiptera|Delphacidae||BOLD:AAZ4510
Delphacidae|[3267]|GMARL1655-14|Hemiptera|Delphacidae||BOLD:ACN9643
Delphacidae|[3268]|GMARM1456-14|Hemiptera|Delphacidae||BOLD:ACN7434
Delphacidae|[3269]|GMARN1755-14|Hemiptera|Delphacidae||BOLD:ACN8559
Delphacidae|[3270]|GMARM1437-14|Hemiptera|Delphacidae||BOLD:ABX3125
Delphacidae|[3271]|GMAGD1918-15|Hemiptera|Delphacidae||BOLD:ACW3643
Metadelphax propinqua|[3272]|GMAGP028-15|Hemiptera|Delphacidae|Delphacinae|BOLD:ABX2193
Delphacidae|[3273]|GMAGD1782-15|Hemiptera|Delphacidae||BOLD:ACV4923
Tagosodes|[3274]|GMAGB1221-15|Hemiptera|Delphacidae|Delphacinae|BOLD:ABZ9630
Delphacidae|[3275]|GMAGB1315-15|Hemiptera|Delphacidae||BOLD:ACW6688
Delphacidae|[3276]|GMAGE1442-15|Hemiptera|Delphacidae||BOLD:ACW7646
Delphacidae|[3277]|GMAGB1344-15|Hemiptera|Delphacidae||BOLD:ACW2870
Delphacidae|[3278]|GMARN1750-14|Hemiptera|Delphacidae||BOLD:ACN8769
Cixiidae|[3279]|GMAGB1166-15|Hemiptera|Cixiidae||BOLD:ACY8208
Cixiidae|[3280]|GMAGB1236-15|Hemiptera|Cixiidae||BOLD:ACY7938
Cixiidae|[3281]|GMAGC1154-15|Hemiptera|Cixiidae||BOLD:ACN9175
Hemiptera|[3282]|GMARA922-14|Hemiptera|||BOLD:ACM5127
Hemiptera|[3283]|GMAGC1196-15|Hemiptera|||BOLD:ACN1062
Achilidae|[3284]|GMARD1337-14|Hemiptera|Achilidae||BOLD:ACN0210
Fulgoridae|[3285]|GMARA762-14|Hemiptera|Fulgoridae||BOLD:ACM7845

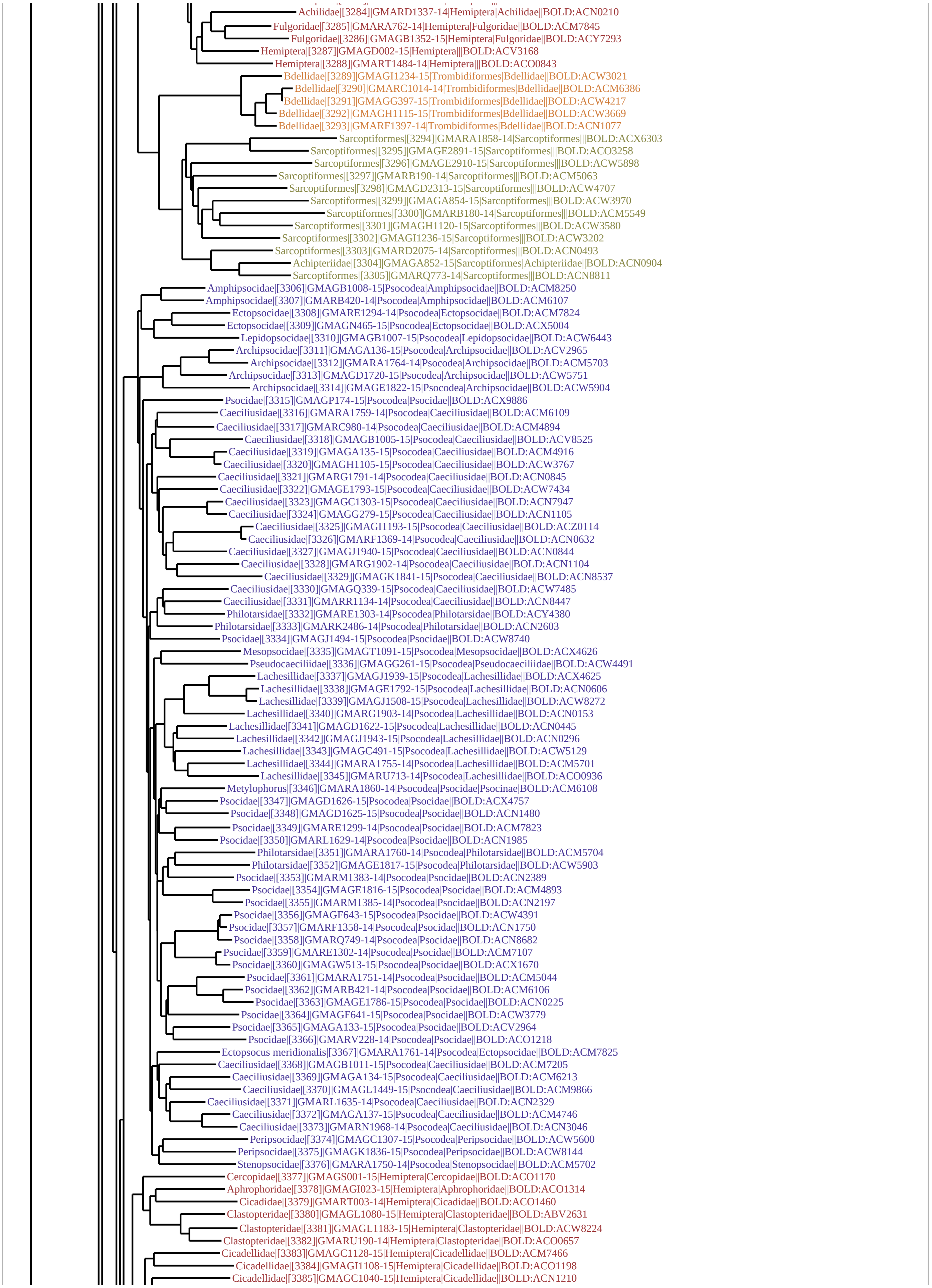
Achilidae|[3284]|GMARD1337-14|Hemiptera|Achilidae||BOLD:ACN0210
Fulgoridae|[3285]|GMARA762-14|Hemiptera|Fulgoridae||BOLD:ACM7845
Fulgoridae|[3286]|GMAGB1352-15|Hemiptera|Fulgoridae||BOLD:ACY7293
Hemiptera|[3287]|GMAGD002-15|Hemiptera|||BOLD:ACV3168
Hemiptera|[3288]|GMART1484-14|Hemiptera|||BOLD:ACO0843
Bdellidae|[3289]|GMAGI1234-15|Trombidiformes|Bdellidae||BOLD:ACW3021
Bdellidae|[3290]|GMARC1014-14|Trombidiformes|Bdellidae||BOLD:ACM6386
Bdellidae|[3291]|GMAGG397-15|Trombidiformes|Bdellidae||BOLD:ACW4217
Bdellidae|[3292]|GMAGH1115-15|Trombidiformes|Bdellidae||BOLD:ACW3669
Bdellidae|[3293]|GMARF1397-14|Trombidiformes|Bdellidae||BOLD:ACN1077
Sarcoptiformes|[3294]|GMARA1858-14|Sarcoptiformes|||BOLD:ACX6303
Sarcoptiformes|[3295]|GMAGE2891-15|Sarcoptiformes|||BOLD:ACO3258
Sarcoptiformes|[3296]|GMAGE2910-15|Sarcoptiformes|||BOLD:ACW5898
Sarcoptiformes|[3297]|GMARB190-14|Sarcoptiformes|||BOLD:ACM5063
Sarcoptiformes|[3298]|GMAGD2313-15|Sarcoptiformes|||BOLD:ACW4707
Sarcoptiformes|[3299]|GMAGA854-15|Sarcoptiformes|||BOLD:ACW3970
Sarcoptiformes|[3300]|GMARB180-14|Sarcoptiformes|||BOLD:ACM5549
Sarcoptiformes|[3301]|GMAGH1120-15|Sarcoptiformes|||BOLD:ACW3580
Sarcoptiformes|[3302]|GMAGI1236-15|Sarcoptiformes|||BOLD:ACW3202
Sarcoptiformes|[3303]|GMARD2075-14|Sarcoptiformes|||BOLD:ACN0493
Achipteriidae|[3304]|GMAGA852-15|Sarcoptiformes|Achipteriidae||BOLD:ACN0904
Sarcoptiformes|[3305]|GMARQ773-14|Sarcoptiformes|||BOLD:ACN8811
Amphipsocidae|[3306]|GMAGB1008-15|Psocodea|Amphipsocidae||BOLD:ACM8250
Amphipsocidae|[3307]|GMARB420-14|Psocodea|Amphipsocidae||BOLD:ACM6107
Ectopsocidae|[3308]|GMARE1294-14|Psocodea|Ectopsocidae||BOLD:ACM7824
Ectopsocidae|[3309]|GMAGN465-15|Psocodea|Ectopsocidae||BOLD:ACX5004
Lepidopsocidae|[3310]|GMAGB1007-15|Psocodea|Lepidopsocidae||BOLD:ACW6443
Archipsocidae|[3311]|GMAGA136-15|Psocodea|Archipsocidae||BOLD:ACV2965
Archipsocidae|[3312]|GMARA1764-14|Psocodea|Archipsocidae||BOLD:ACM5703
Archipsocidae|[3313]|GMAGD1720-15|Psocodea|Archipsocidae||BOLD:ACW5751
Archipsocidae|[3314]|GMAGE1822-15|Psocodea|Archipsocidae||BOLD:ACW5904
Psocidae|[3315]|GMAGP174-15|Psocodea|Psocidae||BOLD:ACX9886
Caeciliusidae|[3316]|GMARA1759-14|Psocodea|Caeciliusidae||BOLD:ACM6109
Caeciliusidae|[3317]|GMARC980-14|Psocodea|Caeciliusidae||BOLD:ACM4894
Caeciliusidae|[3318]|GMAGB1005-15|Psocodea|Caeciliusidae||BOLD:ACV8525
Caeciliusidae|[3319]|GMAGA135-15|Psocodea|Caeciliusidae||BOLD:ACM4916
Caeciliusidae|[3320]|GMAGH1105-15|Psocodea|Caeciliusidae||BOLD:ACW3767
Caeciliusidae|[3321]|GMARG1791-14|Psocodea|Caeciliusidae||BOLD:ACN0845
Caeciliusidae|[3322]|GMAGE1793-15|Psocodea|Caeciliusidae||BOLD:ACW7434
Caeciliusidae|[3323]|GMAGC1303-15|Psocodea|Caeciliusidae||BOLD:ACN7947
Caeciliusidae|[3324]|GMAGG279-15|Psocodea|Caeciliusidae||BOLD:ACN1105
Caeciliusidae|[3325]|GMAGI1193-15|Psocodea|Caeciliusidae||BOLD:ACZ0114
Caeciliusidae|[3326]|GMARF1369-14|Psocodea|Caeciliusidae||BOLD:ACN0632
Caeciliusidae|[3327]|GMAGJ1940-15|Psocodea|Caeciliusidae||BOLD:ACN0844
Caeciliusidae|[3328]|GMARG1902-14|Psocodea|Caeciliusidae||BOLD:ACN1104
Caeciliusidae|[3329]|GMAGK1841-15|Psocodea|Caeciliusidae||BOLD:ACN8537
Caeciliusidae|[3330]|GMAGQ339-15|Psocodea|Caeciliusidae||BOLD:ACW7485
Caeciliusidae|[3331]|GMARR1134-14|Psocodea|Caeciliusidae||BOLD:ACN8447
Philotarsidae|[3332]|GMARE1303-14|Psocodea|Philotarsidae||BOLD:ACY4380
Philotarsidae|[3333]|GMARK2486-14|Psocodea|Philotarsidae||BOLD:ACN2603
Psocidae|[3334]|GMAGJ1494-15|Psocodea|Psocidae||BOLD:ACW8740
Mesopsocidae|[3335]|GMAGT1091-15|Psocodea|Mesopsocidae||BOLD:ACX4626
Pseudocaeciliidae|[3336]|GMAGG261-15|Psocodea|Pseudocaeciliidae||BOLD:ACW4491
Lachesillidae|[3337]|GMAGJ1939-15|Psocodea|Lachesillidae||BOLD:ACX4625
Lachesillidae|[3338]|GMAGE1792-15|Psocodea|Lachesillidae||BOLD:ACN0606
Lachesillidae|[3339]|GMAGJ1508-15|Psocodea|Lachesillidae||BOLD:ACW8272
Lachesillidae|[3340]|GMARG1903-14|Psocodea|Lachesillidae||BOLD:ACN0153
Lachesillidae|[3341]|GMAGD1622-15|Psocodea|Lachesillidae||BOLD:ACN0445
Lachesillidae|[3342]|GMAGJ1943-15|Psocodea|Lachesillidae||BOLD:ACN0296
Lachesillidae|[3343]|GMAGC491-15|Psocodea|Lachesillidae||BOLD:ACW5129
Lachesillidae|[3344]|GMARA1755-14|Psocodea|Lachesillidae||BOLD:ACM5701
Lachesillidae|[3345]|GMARU713-14|Psocodea|Lachesillidae||BOLD:ACO0936
Metylophorus|[3346]|GMARA1860-14|Psocodea|Psocidae|Psocinae|BOLD:ACM6108
Psocidae|[3347]|GMAGD1626-15|Psocodea|Psocidae||BOLD:ACX4757
Psocidae|[3348]|GMAGD1625-15|Psocodea|Psocidae||BOLD:ACN1480
Psocidae|[3349]|GMARE1299-14|Psocodea|Psocidae||BOLD:ACM7823
Psocidae|[3350]|GMARL1629-14|Psocodea|Psocidae||BOLD:ACN1985
Philotarsidae|[3351]|GMARA1760-14|Psocodea|Philotarsidae||BOLD:ACM5704
Philotarsidae|[3352]|GMAGE1817-15|Psocodea|Philotarsidae||BOLD:ACW5903
Psocidae|[3353]|GMARM1383-14|Psocodea|Psocidae||BOLD:ACN2389
Psocidae|[3354]|GMAGE1816-15|Psocodea|Psocidae||BOLD:ACM4893
Psocidae|[3355]|GMARM1385-14|Psocodea|Psocidae||BOLD:ACN2197
Psocidae|[3356]|GMAGF643-15|Psocodea|Psocidae||BOLD:ACW4391
Psocidae|[3357]|GMARF1358-14|Psocodea|Psocidae||BOLD:ACN1750
Psocidae|[3358]|GMARQ749-14|Psocodea|Psocidae||BOLD:ACN8682
Psocidae|[3359]|GMARE1302-14|Psocodea|Psocidae||BOLD:ACM7107
Psocidae|[3360]|GMAGW513-15|Psocodea|Psocidae||BOLD:ACX1670
Psocidae|[3361]|GMARA1751-14|Psocodea|Psocidae||BOLD:ACM5044
Psocidae|[3362]|GMARB421-14|Psocodea|Psocidae||BOLD:ACM6106
Psocidae|[3363]|GMAGE1786-15|Psocodea|Psocidae||BOLD:ACN0225
Psocidae|[3364]|GMAGF641-15|Psocodea|Psocidae||BOLD:ACW3779
Psocidae|[3365]|GMAGA133-15|Psocodea|Psocidae||BOLD:ACV2964
Psocidae|[3366]|GMARV228-14|Psocodea|Psocidae||BOLD:ACO1218
Ectopsocus meridionalis|[3367]|GMARA1761-14|Psocodea|Ectopsocidae||BOLD:ACM7825
Caeciliusidae|[3368]|GMAGB1011-15|Psocodea|Caeciliusidae||BOLD:ACM7205
Caeciliusidae|[3369]|GMAGA134-15|Psocodea|Caeciliusidae||BOLD:ACM6213
Caeciliusidae|[3370]|GMAGL1449-15|Psocodea|Caeciliusidae||BOLD:ACM9866
Caeciliusidae|[3371]|GMARL1635-14|Psocodea|Caeciliusidae||BOLD:ACN2329
Caeciliusidae|[3372]|GMAGA137-15|Psocodea|Caeciliusidae||BOLD:ACM4746
Caeciliusidae|[3373]|GMARN1968-14|Psocodea|Caeciliusidae||BOLD:ACN3046
Peripsocidae|[3374]|GMAGC1307-15|Psocodea|Peripsocidae||BOLD:ACW5600
Peripsocidae|[3375]|GMAGK1836-15|Psocodea|Peripsocidae||BOLD:ACW8144
Stenopsocidae|[3376]|GMARA1750-14|Psocodea|Stenopsocidae||BOLD:ACM5702
Cercopidae|[3377]|GMAGS001-15|Hemiptera|Cercopidae||BOLD:ACO1170
Aphrophoridae|[3378]|GMAGI023-15|Hemiptera|Aphrophoridae||BOLD:ACO1314
Cicadidae|[3379]|GMART003-14|Hemiptera|Cicadidae||BOLD:ACO1460
Clastopteridae|[3380]|GMAGL1080-15|Hemiptera|Clastopteridae||BOLD:ABV2631
Clastopteridae|[3381]|GMAGL1183-15|Hemiptera|Clastopteridae||BOLD:ACW8224
Clastopteridae|[3382]|GMARU190-14|Hemiptera|Clastopteridae||BOLD:ACO0657
Cicadellidae|[3383]|GMAGC1128-15|Hemiptera|Cicadellidae||BOLD:ACM7466
Cicadellidae|[3384]|GMAGI1108-15|Hemiptera|Cicadellidae||BOLD:ACO1198
Cicadellidae|[3385]|GMAGC1040-15|Hemiptera|Cicadellidae||BOLD:ACN1210

Cicadellidae|[3383]|GMAGC1128-15|Hemiptera|Cicadellidae||BOLD:ACM7466
Cicadellidae|[3384]|GMAGI1108-15|Hemiptera|Cicadellidae||BOLD:ACO1198
Cicadellidae|[3385]|GMAGC1040-15|Hemiptera|Cicadellidae||BOLD:ACN1210
Cicadellidae|[3386]|GMARB463-14|Hemiptera|Cicadellidae||BOLD:ACM6262
Cicadellidae|[3387]|GMARA958-14|Hemiptera|Cicadellidae||BOLD:ACM5293
Cicadellidae|[3388]|GMAGD1886-15|Hemiptera|Cicadellidae||BOLD:ACW2871
Cicadellidae|[3389]|GMARA720-14|Hemiptera|Cicadellidae||BOLD:ACM7686
Cicadellidae|[3390]|GMAGB1268-15|Hemiptera|Cicadellidae||BOLD:ACY7576
Cicadellidae|[3391]|GMAGC1167-15|Hemiptera|Cicadellidae||BOLD:ACZ0921
Cicadellidae|[3392]|GMAGA1020-15|Hemiptera|Cicadellidae||BOLD:ACM5128
Cicadellidae|[3393]|GMARA729-14|Hemiptera|Cicadellidae||BOLD:ACM7687
Cicadellidae|[3394]|GMAGO029-15|Hemiptera|Cicadellidae||BOLD:ACX7041
Cicadellidae|[3395]|GMARD1655-14|Hemiptera|Cicadellidae||BOLD:ACM7898
Cicadellidae|[3396]|GMAGA901-15|Hemiptera|Cicadellidae||BOLD:ACM6181
Cicadellidae|[3397]|GMARA779-14|Hemiptera|Cicadellidae||BOLD:ACM5504
Cicadellidae|[3398]|GMAGB1215-15|Hemiptera|Cicadellidae||BOLD:ACN9948
Cicadellidae|[3399]|GMAGD1863-15|Hemiptera|Cicadellidae||BOLD:ACW4833
Cicadellidae|[3400]|GMAGI1094-15|Hemiptera|Cicadellidae||BOLD:ACY7242
Cicadellidae|[3401]|GMARA898-14|Hemiptera|Cicadellidae||BOLD:ACY8391
Cicadellidae|[3402]|GMARN1785-14|Hemiptera|Cicadellidae||BOLD:ACN8728
Cicadellidae|[3403]|GMARA837-14|Hemiptera|Cicadellidae||BOLD:ACY8163
Cicadellidae|[3404]|GMARA1018-14|Hemiptera|Cicadellidae||BOLD:ACM6487
Cicadellidae|[3405]|GMARD1317-14|Hemiptera|Cicadellidae||BOLD:ACN1058
Cicadellidae|[3406]|GMAGC1086-15|Hemiptera|Cicadellidae||BOLD:ACM9992
Cicadellidae|[3407]|GMAGB1305-15|Hemiptera|Cicadellidae||BOLD:ACN0280
Cicadellidae|[3408]|GMAGD1920-15|Hemiptera|Cicadellidae||BOLD:ACN8420
Cicadellidae|[3409]|GMAGV126-15|Hemiptera|Cicadellidae||BOLD:ACY9307
Cicadellidae|[3410]|GMAGA1052-15|Hemiptera|Cicadellidae||BOLD:ACM5687
Cicadellidae|[3411]|GMARR1248-14|Hemiptera|Cicadellidae||BOLD:ADA2705
Cicadellidae|[3412]|GMAGA885-15|Hemiptera|Cicadellidae||BOLD:ACY8509
Cicadellidae|[3413]|GMARA748-14|Hemiptera|Cicadellidae||BOLD:ACM5750
Cicadellidae|[3414]|GMARA815-14|Hemiptera|Cicadellidae||BOLD:ACM6110
Cicadellidae|[3415]|GMARA1016-14|Hemiptera|Cicadellidae||BOLD:ACM5126
Cicadellidae|[3416]|GMAGC1187-15|Hemiptera|Cicadellidae||BOLD:ACM8254
Cicadellidae|[3417]|GMAGA1006-15|Hemiptera|Cicadellidae||BOLD:ACM5341
Cicadellidae|[3418]|GMARA657-14|Hemiptera|Cicadellidae||BOLD:ACM9343
Cicadellidae|[3419]|GMAGA973-15|Hemiptera|Cicadellidae||BOLD:ACM4750
Cicadellidae|[3420]|GMAGA983-15|Hemiptera|Cicadellidae||BOLD:ACM5246
Cicadellidae|[3421]|GMAGA1007-15|Hemiptera|Cicadellidae||BOLD:ACM4852
Cicadellidae|[3422]|GMAGB1205-15|Hemiptera|Cicadellidae||BOLD:ACO1128
Cicadellidae|[3423]|GMAGA897-15|Hemiptera|Cicadellidae||BOLD:ACO1068
Cicadellidae|[3424]|GMARC949-14|Hemiptera|Cicadellidae||BOLD:ACM5749
Cicadellidae|[3425]|GMARA749-14|Hemiptera|Cicadellidae||BOLD:ACY8392
Cicadellidae|[3426]|GMAGH1023-15|Hemiptera|Cicadellidae||BOLD:ACW7984
Cicadellidae|[3427]|GMARS655-14|Hemiptera|Cicadellidae||BOLD:ACN9319
Cicadellidae|[3428]|GMAGA893-15|Hemiptera|Cicadellidae||BOLD:ACM7993
Cicadellidae|[3429]|GMAGI1083-15|Hemiptera|Cicadellidae||BOLD:ACW6687
Cicadellidae|[3430]|GMAGA937-15|Hemiptera|Cicadellidae||BOLD:ACM6180
Cicadellidae|[3431]|GMAGA884-15|Hemiptera|Cicadellidae||BOLD:ACY8164
Cicadellidae|[3432]|GMAGA936-15|Hemiptera|Cicadellidae||BOLD:ACM5137
Cicadellidae|[3433]|GMAGA1026-15|Hemiptera|Cicadellidae||BOLD:ACZ0754
Cicadellidae|[3434]|GMAGB1211-15|Hemiptera|Cicadellidae||BOLD:ACY8239
Cicadellidae|[3435]|GMAGB1224-15|Hemiptera|Cicadellidae||BOLD:ACV5272
Cicadellidae|[3436]|GMAGA1024-15|Hemiptera|Cicadellidae||BOLD:ACM7922
Cicadellidae|[3437]|GMARD1318-14|Hemiptera|Cicadellidae||BOLD:ACM7588
Cicadellidae|[3438]|GMAGA935-15|Hemiptera|Cicadellidae||BOLD:ACM7319
Cicadellidae|[3439]|GMARD1575-14|Hemiptera|Cicadellidae||BOLD:ACM7128
Cicadellidae|[3440]|GMAGF035-15|Hemiptera|Cicadellidae||BOLD:ACW3159
Cicadellidae|[3441]|GMAGF038-15|Hemiptera|Cicadellidae||BOLD:ACW3943
Cicadellidae|[3442]|GMARI1289-14|Hemiptera|Cicadellidae||BOLD:ACN1736
Cicadellidae|[3443]|GMAGA899-15|Hemiptera|Cicadellidae||BOLD:ACM7928
Cicadellidae|[3444]|GMAGI1065-15|Hemiptera|Cicadellidae||BOLD:ACY6683
Cicadellidae|[3445]|GMAGA876-15|Hemiptera|Cicadellidae||BOLD:ACW2243
Cicadellidae|[3446]|GMARG1811-14|Hemiptera|Cicadellidae||BOLD:ACY7359
Cicadellidae|[3447]|GMAGK031-15|Hemiptera|Cicadellidae||BOLD:ADB7453
Cicadellidae|[3448]|GMAGC1117-15|Hemiptera|Cicadellidae||BOLD:ACW1711
Cicadellidae|[3449]|GMAGA878-15|Hemiptera|Cicadellidae||BOLD:ACO1256
Cicadellidae|[3450]|GMARF1193-14|Hemiptera|Cicadellidae||BOLD:ACN0767
Cicadellidae|[3451]|GMARD1335-14|Hemiptera|Cicadellidae||BOLD:ACN1245
Cicadellidae|[3452]|GMARA780-14|Hemiptera|Cicadellidae||BOLD:ACM4772
Cicadellidae|[3453]|GMARB054-14|Hemiptera|Cicadellidae||BOLD:ACM7741
Cicadellidae|[3454]|GMAGC1071-15|Hemiptera|Cicadellidae||BOLD:ACN8457
Cicadellidae|[3455]|GMARA799-14|Hemiptera|Cicadellidae||BOLD:ACW7464
Cicadellidae|[3456]|GMAGT1184-15|Hemiptera|Cicadellidae||BOLD:ACY8116
Cicadellidae|[3457]|GMAGL1071-15|Hemiptera|Cicadellidae||BOLD:ACW9852
Cicadellidae|[3458]|GMARL1704-14|Hemiptera|Cicadellidae||BOLD:ACN9279
Cicadellidae|[3459]|GMARK138-14|Hemiptera|Cicadellidae||BOLD:ACN9123
Cicadellidae|[3460]|GMAGA1030-15|Hemiptera|Cicadellidae||BOLD:ACM6288
Cicadellidae|[3461]|GMARB484-14|Hemiptera|Cicadellidae||BOLD:ACM6179
Cicadellidae|[3462]|GMARN1853-14|Hemiptera|Cicadellidae||BOLD:ACN9477
Cicadellidae|[3463]|GMAGA943-15|Hemiptera|Cicadellidae||BOLD:ACM5276
Cicadellidae|[3464]|GMARA905-14|Hemiptera|Cicadellidae||BOLD:ACM7860
Cicadellidae|[3465]|GMARA873-14|Hemiptera|Cicadellidae||BOLD:ACM8092
Cicadellidae|[3466]|GMAGD1911-15|Hemiptera|Cicadellidae||BOLD:ACY7625
Cicadellidae|[3467]|GMAGA1022-15|Hemiptera|Cicadellidae||BOLD:ACM5522
Cicadellidae|[3468]|GMAGJ1621-15|Hemiptera|Cicadellidae||BOLD:ACW7119
Cicadellidae|[3469]|GMAGD1895-15|Hemiptera|Cicadellidae||BOLD:ACM6417
Cicadellidae|[3470]|GMAGA1031-15|Hemiptera|Cicadellidae||BOLD:ACM4786
Cicadellidae|[3471]|GMARA792-14|Hemiptera|Cicadellidae||BOLD:ACY6936
Cicadellidae|[3472]|GMAGJ1623-15|Hemiptera|Cicadellidae||BOLD:ACW7359
Cicadellidae|[3473]|GMAGA1039-15|Hemiptera|Cicadellidae||BOLD:ACM4785
Cicadellidae|[3474]|GMARA879-14|Hemiptera|Cicadellidae||BOLD:ACM7482
Cicadellidae|[3475]|GMAGA1050-15|Hemiptera|Cicadellidae||BOLD:ACM6353
Cicadellidae|[3476]|GMARK206-14|Hemiptera|Cicadellidae||BOLD:ACN9755
Cicadellidae|[3477]|GMARB506-14|Hemiptera|Cicadellidae||BOLD:ACM6561
Cicadellidae|[3478]|GMAGA895-15|Hemiptera|Cicadellidae||BOLD:ACN7570
Cicadellidae|[3479]|GMARD1639-14|Hemiptera|Cicadellidae||BOLD:ACY6754
Cicadellidae|[3480]|GMARA684-14|Hemiptera|Cicadellidae||BOLD:ACM8178
Cicadellidae|[3481]|GMAGA1038-15|Hemiptera|Cicadellidae||BOLD:ACM8519
Cicadellidae|[3482]|GMAGB1263-15|Hemiptera|Cicadellidae||BOLD:ACM7380
Cicadellidae|[3483]|GMAGB1293-15|Hemiptera|Cicadellidae||BOLD:ACO0448
Cicadellidae|[3484]|GMAGA889-15|Hemiptera|Cicadellidae||BOLD:ACW1925
Cicadellidae|[3485]|GMARO066-14|Hemiptera|Cicadellidae||BOLD:ACN9274

[illegible]
Cicadellidae|[3483]|GMAGB1293-15|Hemiptera|Cicadellidae||BOLD:ACO0448
Cicadellidae|[3484]|GMAGA889-15|Hemiptera|Cicadellidae||BOLD:ACW1925
Cicadellidae|[3485]|GMARQ066-14|Hemiptera|Cicadellidae||BOLD:ACN9274
Cicadellidae|[3486]|GMAGA969-15|Hemiptera|Cicadellidae||BOLD:ACM4787
Cicadellidae|[3487]|GMARF1168-14|Hemiptera|Cicadellidae||BOLD:ACN0793
Cicadellidae|[3488]|GMAGC1158-15|Hemiptera|Cicadellidae||BOLD:ACM6003
Cicadellidae|[3489]|GMAGH059-15|Hemiptera|Cicadellidae||BOLD:ACW3622
Cicadellidae|[3490]|GMARP1091-14|Hemiptera|Cicadellidae||BOLD:ACN9767
Cicadellidae|[3491]|GMAGV132-15|Hemiptera|Cicadellidae||BOLD:ACZ1487
Cicadellidae|[3492]|GMARA842-14|Hemiptera|Cicadellidae||BOLD:ACM9010
Cicadellidae|[3493]|GMAGI1098-15|Hemiptera|Cicadellidae||BOLD:ACY8464
Cicadellidae|[3494]|GMARK2549-14|Hemiptera|Cicadellidae||BOLD:ACO0505
Cicadellidae|[3495]|GMAGC1183-15|Hemiptera|Cicadellidae||BOLD:ACO1038
Cicadellidae|[3496]|GMART1458-14|Hemiptera|Cicadellidae||BOLD:ACO0833
Cicadellidae|[3497]|GMAGA944-15|Hemiptera|Cicadellidae||BOLD:ACM6231
Cicadellidae|[3498]|GMARA846-14|Hemiptera|Cicadellidae||BOLD:AAO8349
Cicadellidae|[3499]|GMAGA938-15|Hemiptera|Cicadellidae||BOLD:ACM6562
Cicadellidae|[3500]|GMARD1615-14|Hemiptera|Cicadellidae||BOLD:ACM8218
Cicadellidae|[3501]|GMART072-14|Hemiptera|Cicadellidae||BOLD:ACO1284
Cicadellidae|[3502]|GMARA649-14|Hemiptera|Cicadellidae||BOLD:ACM9619
Cicadellidae|[3503]|GMAGH058-15|Hemiptera|Cicadellidae||BOLD:ACN0329
Cicadellidae|[3504]|GMARE1022-14|Hemiptera|Cicadellidae||BOLD:ACM7576
Cicadellidae|[3505]|GMARA672-14|Hemiptera|Cicadellidae||BOLD:ACM8958
Cicadellidae|[3506]|GMARA659-14|Hemiptera|Cicadellidae||BOLD:ACM8712
Cicadellidae|[3507]|GMAGA979-15|Hemiptera|Cicadellidae||BOLD:ACW2714
Cicadellidae|[3508]|GMAGC1106-15|Hemiptera|Cicadellidae||BOLD:ACM6182
Cicadellidae|[3509]|GMAGD1858-15|Hemiptera|Cicadellidae||BOLD:ACO0312
Cicadellidae|[3510]|GMAGG2402-15|Hemiptera|Cicadellidae||BOLD:ACN1302
Cicadellidae|[3511]|GMAGA927-15|Hemiptera|Cicadellidae||BOLD:ACM5395
Cicadellidae|[3512]|GMAGB1217-15|Hemiptera|Cicadellidae||BOLD:ACM4804
Cicadellidae|[3513]|GMAGA939-15|Hemiptera|Cicadellidae||BOLD:ACW1391
Cicadellidae|[3514]|GMAGE2776-15|Hemiptera|Cicadellidae||BOLD:ACZ0748
Cicadellidae|[3515]|GMAGX368-15|Hemiptera|Cicadellidae||BOLD:ACY6842
Cicadellidae|[3516]|GMARA595-14|Hemiptera|Cicadellidae||BOLD:ACY5580
Cicadellidae|[3517]|GMARA741-14|Hemiptera|Cicadellidae||BOLD:ACM9324
Cicadellidae|[3518]|GMAGH991-15|Hemiptera|Cicadellidae||BOLD:ACY8721
Cicadellidae|[3519]|GMARB448-14|Hemiptera|Cicadellidae||BOLD:ACM4720
Cicadellidae|[3520]|GMARI1626-14|Hemiptera|Cicadellidae||BOLD:ACN1809
Cicadellidae|[3521]|GMAGY576-15|Hemiptera|Cicadellidae||BOLD:ACY6912
Membracidae|[3522]|GMAGN001-15|Hemiptera|Membracidae||BOLD:ACY3068
Hemiptera|[3523]|GMAGJ1617-15|Hemiptera|||BOLD:ACN0570
Cicadellidae|[3524]|GMARC039-14|Hemiptera|Cicadellidae||BOLD:ACM6139
Cicadellidae|[3525]|GMAGA1045-15|Hemiptera|Cicadellidae||BOLD:ACW1639
Cicadellidae|[3526]|GMARA620-14|Hemiptera|Cicadellidae||BOLD:ACM9200
Cicadellidae|[3527]|GMARA016-14|Hemiptera|Cicadellidae||BOLD:ACN9091
Cicadellidae|[3528]|GMARA012-14|Hemiptera|Cicadellidae||BOLD:ACN9139
Cicadellidae|[3529]|GMARA019-14|Hemiptera|Cicadellidae||BOLD:AAV0142
Cicadellidae|[3530]|GMAGB109-15|Hemiptera|Cicadellidae||BOLD:ACN9353
Cicadellidae|[3531]|GMARR056-14|Hemiptera|Cicadellidae||BOLD:ACO1990
Cicadellidae|[3532]|GMAGA005-15|Hemiptera|Cicadellidae||BOLD:ACN8851
Cicadellidae|[3533]|GMARH096-14|Hemiptera|Cicadellidae||BOLD:ACN8207
Cicadellidae|[3534]|GMAGB090-15|Hemiptera|Cicadellidae||BOLD:ACW3469
Cicadellidae|[3535]|GMARN002-14|Hemiptera|Cicadellidae||BOLD:ACO1206
Cicadellidae|[3536]|GMAGA003-15|Hemiptera|Cicadellidae||BOLD:ACN7826
Cicadellidae|[3537]|GMARA004-14|Hemiptera|Cicadellidae||BOLD:ACN9744
Cicadellidae|[3538]|GMAGE1465-15|Hemiptera|Cicadellidae||BOLD:ACY8015
Cicadellidae|[3539]|GMARI003-14|Hemiptera|Cicadellidae||BOLD:ACO0464
Cicadellidae|[3540]|GMAGD121-15|Hemiptera|Cicadellidae||BOLD:ACO1255
Cicadellidae|[3541]|GMAGR001-15|Hemiptera|Cicadellidae||BOLD:ACY2034
Cicadellidae|[3542]|GMAGI1104-15|Hemiptera|Cicadellidae||BOLD:ACY8266
Cicadellidae|[3543]|GMARA655-14|Hemiptera|Cicadellidae||BOLD:ACM7323
Cicadellidae|[3544]|GMAGS050-15|Hemiptera|Cicadellidae||BOLD:ACY8791
Cicadellidae|[3545]|GMARB003-14|Hemiptera|Cicadellidae||BOLD:ACN8709
Cicadellidae|[3546]|GMAGA924-15|Hemiptera|Cicadellidae||BOLD:ACW2208
Cicadellidae|[3547]|GMAGY104-15|Hemiptera|Cicadellidae||BOLD:ACY3030
Cicadellidae|[3548]|GMAGC1036-15|Hemiptera|Cicadellidae||BOLD:ACW1817
Cicadellidae|[3549]|GMAGA919-15|Hemiptera|Cicadellidae||BOLD:ACW1483
Cicadellidae|[3550]|GMAGK030-15|Hemiptera|Cicadellidae||BOLD:AAZ4509
Cicadellidae|[3551]|GMAGO1467-15|Hemiptera|Cicadellidae||BOLD:ACX7293
Cicadellidae|[3552]|GMARM1421-14|Hemiptera|Cicadellidae||BOLD:ACN8387
Cicadellidae|[3553]|GMARN1793-14|Hemiptera|Cicadellidae||BOLD:ACN8435
Cicadellidae|[3554]|GMAGJ1575-15|Hemiptera|Cicadellidae||BOLD:ACY7501
Cicadellidae|[3555]|GMARF1194-14|Hemiptera|Cicadellidae||BOLD:ACX7517
Cicadellidae|[3556]|GMAGG2416-15|Hemiptera|Cicadellidae||BOLD:ACN0191
Cicadellidae|[3557]|GMARG022-14|Hemiptera|Cicadellidae||BOLD:ACN0720
Cicadellidae|[3558]|GMARB039-14|Hemiptera|Cicadellidae||BOLD:ACM6352
Cicadellidae|[3559]|GMAGE1577-15|Hemiptera|Cicadellidae||BOLD:ACW7339
Cicadellidae|[3560]|GMARR1149-14|Hemiptera|Cicadellidae||BOLD:ACN9308
Cicadellidae|[3561]|GMARL137-14|Hemiptera|Cicadellidae||BOLD:ACN9592
Cicadellidae|[3562]|GMAGI1023-15|Hemiptera|Cicadellidae||BOLD:ACW6691
Cicadellidae|[3563]|GMAGP068-15|Hemiptera|Cicadellidae||BOLD:ACX6739
Cicadellidae|[3564]|GMAGG2367-15|Hemiptera|Cicadellidae||BOLD:ACY8792
Cicadellidae|[3565]|GMAGR063-15|Hemiptera|Cicadellidae||BOLD:ACY1357
Cicadellidae|[3566]|GMARR1221-14|Hemiptera|Cicadellidae||BOLD:ACN9676
Cicadellidae|[3567]|GMAGA977-15|Hemiptera|Cicadellidae||BOLD:ACM5289
Balclutha incisa|[3568]|GMARK151-14|Hemiptera|Cicadellidae|Deltocephalinae|BOLD:AAC9294
Cicadellidae|[3569]|GMARD1766-14|Hemiptera|Cicadellidae||BOLD:ACM7594
Cicadellidae|[3570]|GMAGH073-15|Hemiptera|Cicadellidae||BOLD:ACW3097
Cicadellidae|[3571]|GMARA835-14|Hemiptera|Cicadellidae||BOLD:ACM9523
Cicadellidae|[3572]|GMARA737-14|Hemiptera|Cicadellidae||BOLD:ACM6281
Cicadellidae|[3573]|GMARN1819-14|Hemiptera|Cicadellidae||BOLD:ACN7958
Cicadellidae|[3574]|GMAGK034-15|Hemiptera|Cicadellidae||BOLD:ACM9765
Cicadellidae|[3575]|GMAGA959-15|Hemiptera|Cicadellidae||BOLD:ACZ0641
Cicadellidae|[3576]|GMARA816-14|Hemiptera|Cicadellidae||BOLD:ACY7479
Cicadellidae|[3577]|GMARG1836-14|Hemiptera|Cicadellidae||BOLD:ACN1426
Cicadellidae|[3578]|GMAGA976-15|Hemiptera|Cicadellidae||BOLD:ACW2150
Cicadellidae|[3579]|GMAGQ485-15|Hemiptera|Cicadellidae||BOLD:ACX6863
Cicadellidae|[3580]|GMAGU046-15|Hemiptera|Cicadellidae||BOLD:ACY7517
Cicadellidae|[3581]|GMAGA907-15|Hemiptera|Cicadellidae||BOLD:AAV0140
Cicadellidae|[3582]|GMARA823-14|Hemiptera|Cicadellidae||BOLD:ACM8357
Cicadellidae|[3583]|GMAGJ1606-15|Hemiptera|Cicadellidae||BOLD:ACY8579
Cicadellidae|[3584]|GMAGK047-15|Hemiptera|Cicadellidae||BOLD:ACY6884
[illegible]

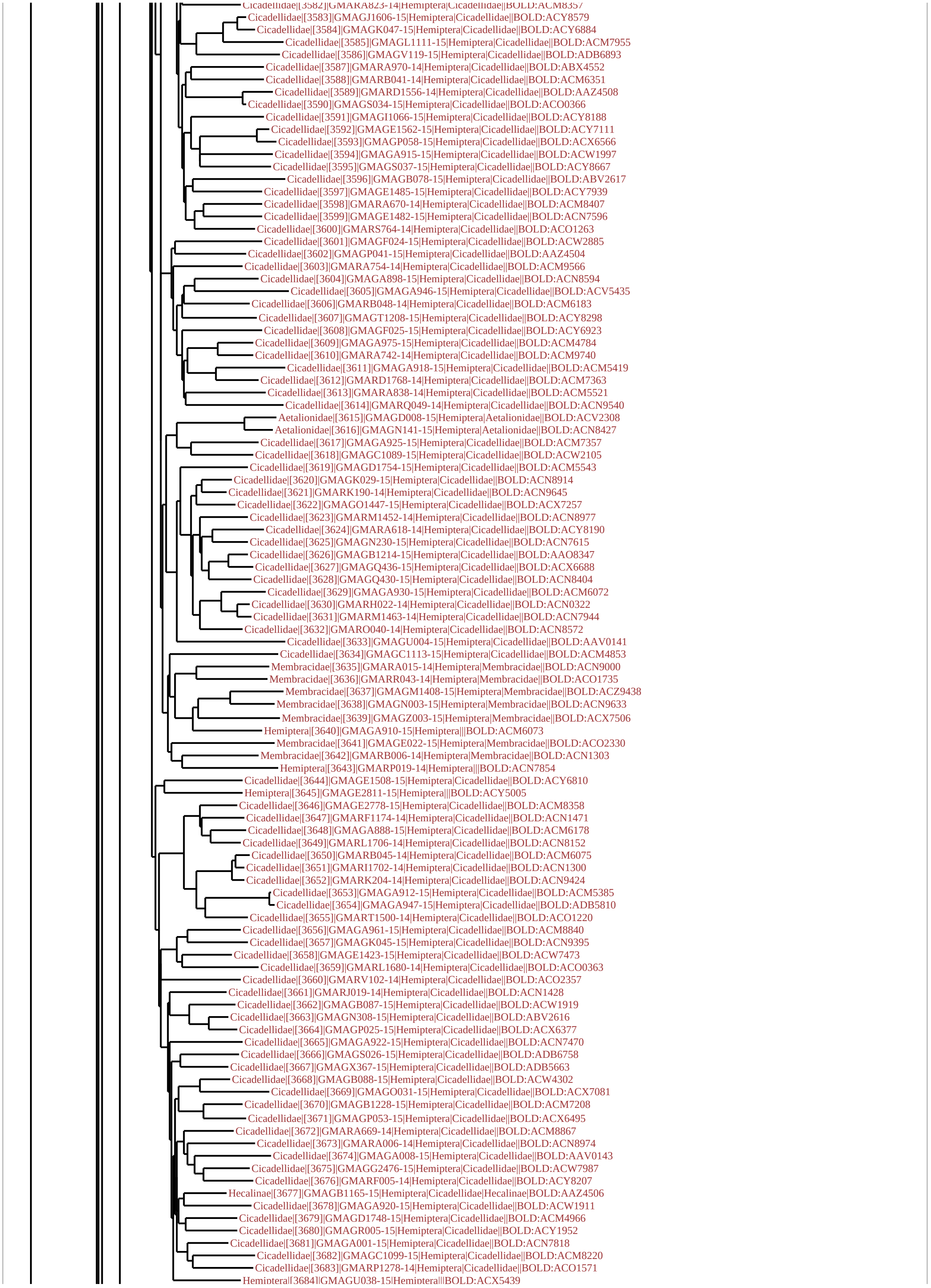
Cicadellidae|[3582]|GMARA823-14|Hemiptera|Cicadellidae||BOLD:ACM8357
Cicadellidae|[3583]|GMAGJ1606-15|Hemiptera|Cicadellidae||BOLD:ACY8579
Cicadellidae|[3584]|GMAGK047-15|Hemiptera|Cicadellidae||BOLD:ACY6884
Cicadellidae|[3585]|GMAGL1111-15|Hemiptera|Cicadellidae||BOLD:ACM7955
Cicadellidae|[3586]|GMAGV119-15|Hemiptera|Cicadellidae||BOLD:ADB6893
Cicadellidae|[3587]|GMARA970-14|Hemiptera|Cicadellidae||BOLD:ABX4552
Cicadellidae|[3588]|GMARB041-14|Hemiptera|Cicadellidae||BOLD:ACM6351
Cicadellidae|[3589]|GMARD1556-14|Hemiptera|Cicadellidae||BOLD:AAZ4508
Cicadellidae|[3590]|GMAGS034-15|Hemiptera|Cicadellidae||BOLD:ACO0366
Cicadellidae|[3591]|GMAGI1066-15|Hemiptera|Cicadellidae||BOLD:ACY8188
Cicadellidae|[3592]|GMAGE1562-15|Hemiptera|Cicadellidae||BOLD:ACY7111
Cicadellidae|[3593]|GMAGP058-15|Hemiptera|Cicadellidae||BOLD:ACX6566
Cicadellidae|[3594]|GMAGA915-15|Hemiptera|Cicadellidae||BOLD:ACW1997
Cicadellidae|[3595]|GMAGS037-15|Hemiptera|Cicadellidae||BOLD:ACY8667
Cicadellidae|[3596]|GMAGB078-15|Hemiptera|Cicadellidae||BOLD:ABV2617
Cicadellidae|[3597]|GMAGE1485-15|Hemiptera|Cicadellidae||BOLD:ACY7939
Cicadellidae|[3598]|GMARA670-14|Hemiptera|Cicadellidae||BOLD:ACM8407
Cicadellidae|[3599]|GMAGE1482-15|Hemiptera|Cicadellidae||BOLD:ACN7596
Cicadellidae|[3600]|GMARS764-14|Hemiptera|Cicadellidae||BOLD:ACO1263
Cicadellidae|[3601]|GMAGF024-15|Hemiptera|Cicadellidae||BOLD:ACW2885
Cicadellidae|[3602]|GMAGP041-15|Hemiptera|Cicadellidae||BOLD:AAZ4504
Cicadellidae|[3603]|GMARA754-14|Hemiptera|Cicadellidae||BOLD:ACM9566
Cicadellidae|[3604]|GMAGA898-15|Hemiptera|Cicadellidae||BOLD:ACN8594
Cicadellidae|[3605]|GMAGA946-15|Hemiptera|Cicadellidae||BOLD:ACV5435
Cicadellidae|[3606]|GMARB048-14|Hemiptera|Cicadellidae||BOLD:ACM6183
Cicadellidae|[3607]|GMAGT1208-15|Hemiptera|Cicadellidae||BOLD:ACY8298
Cicadellidae|[3608]|GMAGF025-15|Hemiptera|Cicadellidae||BOLD:ACY6923
Cicadellidae|[3609]|GMAGA975-15|Hemiptera|Cicadellidae||BOLD:ACM4784
Cicadellidae|[3610]|GMARA742-14|Hemiptera|Cicadellidae||BOLD:ACM9740
Cicadellidae|[3611]|GMAGA918-15|Hemiptera|Cicadellidae||BOLD:ACM5419
Cicadellidae|[3612]|GMARD1768-14|Hemiptera|Cicadellidae||BOLD:ACM7363
Cicadellidae|[3613]|GMARA838-14|Hemiptera|Cicadellidae||BOLD:ACM5521
Cicadellidae|[3614]|GMARQ049-14|Hemiptera|Cicadellidae||BOLD:ACN9540
Aetalionidae|[3615]|GMAGD008-15|Hemiptera|Aetalionidae||BOLD:ACV2308
Aetalionidae|[3616]|GMAGN141-15|Hemiptera|Aetalionidae||BOLD:ACN8427
Cicadellidae|[3617]|GMAGA925-15|Hemiptera|Cicadellidae||BOLD:ACM7357
Cicadellidae|[3618]|GMAGC1089-15|Hemiptera|Cicadellidae||BOLD:ACW2105
Cicadellidae|[3619]|GMAGD1754-15|Hemiptera|Cicadellidae||BOLD:ACM5543
Cicadellidae|[3620]|GMAGK029-15|Hemiptera|Cicadellidae||BOLD:ACN8914
Cicadellidae|[3621]|GMARK190-14|Hemiptera|Cicadellidae||BOLD:ACN9645
Cicadellidae|[3622]|GMAGO1447-15|Hemiptera|Cicadellidae||BOLD:ACX7257
Cicadellidae|[3623]|GMARM1452-14|Hemiptera|Cicadellidae||BOLD:ACN8977
Cicadellidae|[3624]|GMARA618-14|Hemiptera|Cicadellidae||BOLD:ACY8190
Cicadellidae|[3625]|GMAGN230-15|Hemiptera|Cicadellidae||BOLD:ACN7615
Cicadellidae|[3626]|GMAGB1214-15|Hemiptera|Cicadellidae||BOLD:AAO8347
Cicadellidae|[3627]|GMAGQ436-15|Hemiptera|Cicadellidae||BOLD:ACX6688
Cicadellidae|[3628]|GMAGQ430-15|Hemiptera|Cicadellidae||BOLD:ACN8404
Cicadellidae|[3629]|GMAGA930-15|Hemiptera|Cicadellidae||BOLD:ACM6072
Cicadellidae|[3630]|GMARH022-14|Hemiptera|Cicadellidae||BOLD:ACN0322
Cicadellidae|[3631]|GMARM1463-14|Hemiptera|Cicadellidae||BOLD:ACN7944
Cicadellidae|[3632]|GMARO040-14|Hemiptera|Cicadellidae||BOLD:ACN8572
Cicadellidae|[3633]|GMAGU004-15|Hemiptera|Cicadellidae||BOLD:AAV0141
Cicadellidae|[3634]|GMAGC1113-15|Hemiptera|Cicadellidae||BOLD:ACM4853
Membracidae|[3635]|GMARA015-14|Hemiptera|Membracidae||BOLD:ACN9000
Membracidae|[3636]|GMARR043-14|Hemiptera|Membracidae||BOLD:ACO1735
Membracidae|[3637]|GMAGM1408-15|Hemiptera|Membracidae||BOLD:ACZ9438
Membracidae|[3638]|GMAGN003-15|Hemiptera|Membracidae||BOLD:ACN9633
Membracidae|[3639]|GMAGZ003-15|Hemiptera|Membracidae||BOLD:ACX7506
Hemiptera|[3640]|GMAGA910-15|Hemiptera|||BOLD:ACM6073
Membracidae|[3641]|GMAGE022-15|Hemiptera|Membracidae||BOLD:ACO2330
Membracidae|[3642]|GMARB006-14|Hemiptera|Membracidae||BOLD:ACN1303
Hemiptera|[3643]|GMARP019-14|Hemiptera|||BOLD:ACN7854
Cicadellidae|[3644]|GMAGE1508-15|Hemiptera|Cicadellidae||BOLD:ACY6810
Hemiptera|[3645]|GMAGE2811-15|Hemiptera|||BOLD:ACY5005
Cicadellidae|[3646]|GMAGE2778-15|Hemiptera|Cicadellidae||BOLD:ACM8358
Cicadellidae|[3647]|GMARF1174-14|Hemiptera|Cicadellidae||BOLD:ACN1471
Cicadellidae|[3648]|GMAGA888-15|Hemiptera|Cicadellidae||BOLD:ACM6178
Cicadellidae|[3649]|GMARL1706-14|Hemiptera|Cicadellidae||BOLD:ACN8152
Cicadellidae|[3650]|GMARB045-14|Hemiptera|Cicadellidae||BOLD:ACM6075
Cicadellidae|[3651]|GMARI1702-14|Hemiptera|Cicadellidae||BOLD:ACN1300
Cicadellidae|[3652]|GMARK204-14|Hemiptera|Cicadellidae||BOLD:ACN9424
Cicadellidae|[3653]|GMAGA912-15|Hemiptera|Cicadellidae||BOLD:ACM5385
Cicadellidae|[3654]|GMAGA947-15|Hemiptera|Cicadellidae||BOLD:ADB5810
Cicadellidae|[3655]|GMART1500-14|Hemiptera|Cicadellidae||BOLD:ACO1220
Cicadellidae|[3656]|GMAGA961-15|Hemiptera|Cicadellidae||BOLD:ACM8840
Cicadellidae|[3657]|GMAGK045-15|Hemiptera|Cicadellidae||BOLD:ACN9395
Cicadellidae|[3658]|GMAGE1423-15|Hemiptera|Cicadellidae||BOLD:ACW7473
Cicadellidae|[3659]|GMARL1680-14|Hemiptera|Cicadellidae||BOLD:ACO0363
Cicadellidae|[3660]|GMARV102-14|Hemiptera|Cicadellidae||BOLD:ACO2357
Cicadellidae|[3661]|GMARJ019-14|Hemiptera|Cicadellidae||BOLD:ACN1428
Cicadellidae|[3662]|GMAGB087-15|Hemiptera|Cicadellidae||BOLD:ACW1919
Cicadellidae|[3663]|GMAGN308-15|Hemiptera|Cicadellidae||BOLD:ABV2616
Cicadellidae|[3664]|GMAGP025-15|Hemiptera|Cicadellidae||BOLD:ACX6377
Cicadellidae|[3665]|GMAGA922-15|Hemiptera|Cicadellidae||BOLD:ACN7470
Cicadellidae|[3666]|GMAGS026-15|Hemiptera|Cicadellidae||BOLD:ADB6758
Cicadellidae|[3667]|GMAGX367-15|Hemiptera|Cicadellidae||BOLD:ADB5663
Cicadellidae|[3668]|GMAGB088-15|Hemiptera|Cicadellidae||BOLD:ACW4302
Cicadellidae|[3669]|GMAGO031-15|Hemiptera|Cicadellidae||BOLD:ACX7081
Cicadellidae|[3670]|GMAGB1228-15|Hemiptera|Cicadellidae||BOLD:ACM7208
Cicadellidae|[3671]|GMAGP053-15|Hemiptera|Cicadellidae||BOLD:ACX6495
Cicadellidae|[3672]|GMARA669-14|Hemiptera|Cicadellidae||BOLD:ACM8867
Cicadellidae|[3673]|GMARA006-14|Hemiptera|Cicadellidae||BOLD:ACN8974
Cicadellidae|[3674]|GMAGA008-15|Hemiptera|Cicadellidae||BOLD:AAV0143
Cicadellidae|[3675]|GMAGG2476-15|Hemiptera|Cicadellidae||BOLD:ACW7987
Cicadellidae|[3676]|GMARF005-14|Hemiptera|Cicadellidae||BOLD:ACY8207
Hecalinae|[3677]|GMAGB1165-15|Hemiptera|Cicadellidae|Hecalinae|BOLD:AAZ4506
Cicadellidae|[3678]|GMAGA920-15|Hemiptera|Cicadellidae||BOLD:ACW1911
Cicadellidae|[3679]|GMAGD1748-15|Hemiptera|Cicadellidae||BOLD:ACM4966
Cicadellidae|[3680]|GMAGR005-15|Hemiptera|Cicadellidae||BOLD:ACY1952
Cicadellidae|[3681]|GMAGA001-15|Hemiptera|Cicadellidae||BOLD:ACN7818
Cicadellidae|[3682]|GMAGC1099-15|Hemiptera|Cicadellidae||BOLD:ACM8220
Cicadellidae|[3683]|GMARP1278-14|Hemiptera|Cicadellidae||BOLD:ACO1571
Hemiptera|[3684]|GMAGU038-15|Hemiptera|||BOLD:ACX5439

Cicadellidae|[3682]|GMAGC1099-15|Hemiptera|Cicadellidae||BOLD:ACM8220
Cicadellidae|[3683]|GMARP1278-14|Hemiptera|Cicadellidae||BOLD:ACO1571
Hemiptera|[3684]|GMAGU038-15|Hemiptera|||BOLD:ACX5439
Archaeognatha|[3685]|GMAGR435-15|Archaeognatha|||BOLD:ACO2156
Entomobryidae|[3686]|GMAGE1872-15|Entomobryomorpha|Entomobryidae||BOLD:ACN0559
Entomobryidae|[3687]|GMAGK2005-15|Entomobryomorpha|Entomobryidae||BOLD:ACW8629
Entomobryidae|[3688]|GMAGA795-15|Entomobryomorpha|Entomobryidae||BOLD:ACM6024
Entomobryidae|[3689]|GMAGA801-15|Entomobryomorpha|Entomobryidae||BOLD:ACM4890
Entomobryidae|[3690]|GMAGA789-15|Entomobryomorpha|Entomobryidae||BOLD:ACM6207
Entomobryidae|[3691]|GMAGA822-15|Entomobryomorpha|Entomobryidae||BOLD:ACM6126
Entomobryidae|[3692]|GMAGC333-15|Entomobryomorpha|Entomobryidae||BOLD:ACW1445
Entomobryidae|[3693]|GMARB201-14|Entomobryomorpha|Entomobryidae||BOLD:ACM6357
Entomobryidae|[3694]|GMAGC353-15|Entomobryomorpha|Entomobryidae||BOLD:ACN9900
Entomobryidae|[3695]|GMARK2515-14|Entomobryomorpha|Entomobryidae||BOLD:ACN2678
Entomobryidae|[3696]|GMARB216-14|Entomobryomorpha|Entomobryidae||BOLD:ACM6450
Entomobryomorpha|[3697]|GMARO1348-14|Entomobryomorpha|||BOLD:ACN8376
Entomobryomorpha|[3698]|GMARQ737-14|Entomobryomorpha|||BOLD:ACN7803
Entomobryomorpha|[3699]|GMAGD1956-15|Entomobryomorpha|||BOLD:ACW1766
Entomobryomorpha|[3700]|GMAGT1148-15|Entomobryomorpha|||BOLD:ACX2704
Entomobryomorpha|[3701]|GMARQ744-14|Entomobryomorpha|||BOLD:ACN8516
Entomobryomorpha|[3702]|GMAGY481-15|Entomobryomorpha|||BOLD:ACX1693
Entomobryomorpha|[3703]|GMAGW505-15|Entomobryomorpha|||BOLD:ACX2002
Entomobryomorpha|[3704]|GMART1652-14|Entomobryomorpha|||BOLD:ACO0090
Entomobryomorpha|[3705]|GMAGT1162-15|Entomobryomorpha|||BOLD:ACX5839
Entomobryidae|[3706]|GMAGE1842-15|Entomobryomorpha|Entomobryidae||BOLD:ACN1543
Entomobryidae|[3707]|GMAGE1847-15|Entomobryomorpha|Entomobryidae||BOLD:ACN1549
Entomobryidae|[3708]|GMAGK1994-15|Entomobryomorpha|Entomobryidae||BOLD:ACX0206
Entomobryidae|[3709]|GMAGQ379-15|Entomobryomorpha|Entomobryidae||BOLD:ACN8667
Entomobryomorpha|[3710]|GMAGL1588-15|Entomobryomorpha|||BOLD:ACX3800
Entomobryomorpha|[3711]|GMARP1173-14|Entomobryomorpha|||BOLD:ACN8256
Entomobryomorpha|[3712]|GMAGJ1984-15|Entomobryomorpha|||BOLD:ACX3107
Entomobryomorpha|[3713]|GMAGY490-15|Entomobryomorpha|||BOLD:ACX2010
Poduromorpha|[3714]|GMARA1838-14|Poduromorpha|||BOLD:ACM5041
Poduromorpha|[3715]|GMARA1832-14|Poduromorpha|||BOLD:ACM6127
Poduromorpha|[3716]|GMAGU935-15|Poduromorpha|||BOLD:ACX2121
Symphypleona|[3717]|GMARQ727-14|Symphypleona|||BOLD:ACN8454
Symphypleona|[3718]|GMAGH1078-15|Symphypleona|||BOLD:ACW4312
Katiannidae|[3719]|GMAGI1213-15|Symphypleona|Katiannidae||BOLD:ACN1377
Symphypleona|[3720]|GMAGA842-15|Symphypleona|||BOLD:ACM6025
Symphypleona|[3721]|GMAGD1958-15|Symphypleona|||BOLD:ACW1928
Symphypleona|[3722]|GMAGT1147-15|Symphypleona|||BOLD:ACN8814
Symphypleona|[3723]|GMAGY494-15|Symphypleona|||BOLD:ACX2021
Trematuridae|[3724]|GMARE1346-14|Mesostigmata|Trematuridae||BOLD:ACM7760
Mesostigmata|[3725]|GMARM1503-14|Mesostigmata|||BOLD:ACN6275
Araneae|[3726]|GMARA984-14|Araneae|||BOLD:ACN1452
Salticidae|[3727]|GMAGH1112-15|Araneae|Salticidae||BOLD:ACW3336
Salticidae|[3728]|GMARE1380-14|Araneae|Salticidae||BOLD:ACN1288
Salticidae|[3729]|GMARB411-14|Araneae|Salticidae||BOLD:ACM4960
Salticidae|[3730]|GMAGP178-15|Araneae|Salticidae||BOLD:ACN9455
Salticidae|[3731]|GMAGA131-15|Araneae|Salticidae||BOLD:ACM5020
Salticidae|[3732]|GMARV231-14|Araneae|Salticidae||BOLD:ACO1178
Salticidae|[3733]|GMAGJ1931-15|Araneae|Salticidae||BOLD:ACX5509
Selenopidae|[3734]|GMAGH009-15|Araneae|Selenopidae||BOLD:ACX1823
Thomisidae|[3735]|GMAGD1637-15|Araneae|Thomisidae||BOLD:ACW3721
Thomisidae|[3736]|GMAGA128-15|Araneae|Thomisidae||BOLD:ACN0734
Thomisidae|[3737]|GMAGD1631-15|Araneae|Thomisidae||BOLD:ACW2917
Thomisidae|[3738]|GMARC1015-14|Araneae|Thomisidae||BOLD:ACM5879
Thomisidae|[3739]|GMAGD1636-15|Araneae|Thomisidae||BOLD:ACW3079
Thomisidae|[3740]|GMAGG178-15|Araneae|Thomisidae||BOLD:ACW4121
Thomisidae|[3741]|GMAGE1667-15|Araneae|Thomisidae||BOLD:ACW2000
Thomisidae|[3742]|GMARP1084-14|Araneae|Thomisidae||BOLD:ACN9358
Hogna gumia|[3743]|GMAGW152-15|Araneae|Lycosidae||BOLD:AAW0244
Lycosa erythrognatha|[3744]|GMARA985-14|Araneae|Lycosidae||BOLD:ACM9975
Lycosa erythrognatha|[3745]|GMARW160-15|Araneae|Lycosidae||BOLD:ACY7272
Agalenocosa|[3746]|GMAGC467-15|Araneae|Lycosidae||BOLD:ABV1799
Lycosidae|[3747]|GMAGY516-15|Araneae|Lycosidae||BOLD:AAX5852
Pisauridae|[3748]|GMAGN520-15|Araneae|Pisauridae||BOLD:ACN2264
Oxyopidae|[3749]|GMARM1392-14|Araneae|Oxyopidae||BOLD:ACN2177
Lycosidae|[3750]|GMART1666-14|Araneae|Lycosidae||BOLD:ACO0914
Thaumasia velox|[3751]|GMAGL1050-15|Araneae|Pisauridae||BOLD:ABV1505
Araneae|[3752]|GMAGG009-15|Araneae|||BOLD:ACX7150
Styposis selis|[3753]|GMARA1849-14|Araneae|Theridiidae||BOLD:AAW0968
Theridiidae|[3754]|GMAGW527-15|Araneae|Theridiidae||BOLD:ACX2561
Theridiidae|[3755]|GMARD2058-14|Araneae|Theridiidae||BOLD:ACN1722
Theridiidae|[3756]|GMAGA130-15|Araneae|Theridiidae||BOLD:ACV1878
Theridiidae|[3757]|GMARD2055-14|Araneae|Theridiidae||BOLD:ACN8132
Theridiidae|[3758]|GMARD2059-14|Araneae|Theridiidae||BOLD:AAW0974
Theridiidae|[3759]|GMAGH1114-15|Araneae|Theridiidae||BOLD:ACW4816
Theridiidae|[3760]|GMAGG387-15|Araneae|Theridiidae||BOLD:ACX4947
Theridiidae|[3761]|GMAGV926-15|Araneae|Theridiidae||BOLD:ACX4966
Theridiidae|[3762]|GMAGI1223-15|Araneae|Theridiidae||BOLD:ACW4256
Theridiidae|[3763]|GMAGY517-15|Araneae|Theridiidae||BOLD:ACX3279
Araneae|[3764]|GMAGA132-15|Araneae|||BOLD:ACV2377
Trachelidae|[3765]|GMARE024-14|Araneae|Trachelidae||BOLD:ACM9850
Polybetes rapidus|[3766]|GMARA987-14|Araneae|Sparassidae||BOLD:ACN1242
Sparassidae|[3767]|GMARA1850-14|Araneae|Sparassidae||BOLD:ACO1112
Sparassidae|[3768]|GMAGQ037-15|Araneae|Sparassidae||BOLD:ACN9579
Araneae|[3769]|GMAGH1108-15|Araneae|||BOLD:ACW4165
Anyphaenidae|[3770]|GMAGG181-15|Araneae|Anyphaenidae||BOLD:ACW4122
Anyphaenidae|[3771]|GMAGC471-15|Araneae|Anyphaenidae||BOLD:ACM5183
Anyphaenidae|[3772]|GMAGG388-15|Araneae|Anyphaenidae||BOLD:ACN2328
Anyphaenidae|[3773]|GMARA1845-14|Araneae|Anyphaenidae||BOLD:ACM5021
Patrera procera|[3774]|GMAGE1660-15|Araneae|Anyphaenidae||BOLD:ACM5184
Anyphaenidae|[3775]|GMARB414-14|Araneae|Anyphaenidae||BOLD:ACX4236
Arachosia praesignis|[3776]|GMAGQ349-15|Araneae|Anyphaenidae||BOLD:ACG2183
Anyphaenidae|[3777]|GMAGG002-15|Araneae|Anyphaenidae||BOLD:ACX3723
Anyphaenidae|[3778]|GMAGB1012-15|Araneae|Anyphaenidae||BOLD:ACO1167
Anyphaenidae|[3779]|GMAGE006-15|Araneae|Anyphaenidae||BOLD:ACX2158
Anyphaenidae|[3780]|GMARA986-14|Araneae|Anyphaenidae||BOLD:ACN0172
Anyphaenidae|[3781]|GMAGE1659-15|Araneae|Anyphaenidae||BOLD:ACW1433
Anyphaenidae|[3782]|GMARE1376-14|Araneae|Anyphaenidae||BOLD:ACN0931
Anyphaenidae|[3783]|GMAGL1049-15|Araneae|Anyphaenidae||BOLD:ACN9178
[illegible]

Anyphaenidae|[3781]|GMAGE1659-15|Araneae|Anyphaenidae||BOLD:ACW1433
Anyphaenidae|[3782]|GMARE1376-14|Araneae|Anyphaenidae||BOLD:ACN0931
Anyphaenidae|[3783]|GMAGL1049-15|Araneae|Anyphaenidae||BOLD:ACN9178
Anyphaenidae|[3784]|GMAGC474-15|Araneae|Anyphaenidae||BOLD:ACW1840
Anyphaenidae|[3785]|GMAGA851-15|Araneae|Anyphaenidae||BOLD:ACM5774
Anyphaenidae|[3786]|GMAGH1107-15|Araneae|Anyphaenidae||BOLD:ACN9626
Anyphaenidae|[3787]|GMAGQ345-15|Araneae|Anyphaenidae||BOLD:ACX5428
Araneae|[3788]|GMAGV925-15|Araneae|||BOLD:ACX4505
Corinnidae|[3789]|GMAGB012-15|Araneae|Corinnidae||BOLD:ACO2239
Corinnidae|[3790]|GMAGG001-15|Araneae|Corinnidae||BOLD:ACX3873
Araneae|[3791]|GMAGX002-15|Araneae|||BOLD:ACY1953
Araneae|[3792]|GMAGD1640-15|Araneae|||BOLD:ACW5570
Nephila clavipes|[3793]|GMART1665-14|Araneae|Nephilidae||BOLD:AAG7097
Xylethrus superbus|[3794]|GMAGH1109-15|Araneae|Araneidae||BOLD:ACT2295
Araneidae|[3795]|GMAGQ348-15|Araneae|Araneidae||BOLD:ACX4080
Micrathena furva|[3796]|GMAGT1220-15|Araneae|Araneidae||BOLD:ACT2407
Araneidae|[3797]|GMARD2052-14|Araneae|Araneidae||BOLD:ACN0579
Araneidae|[3798]|GMAGW127-15|Araneae|Araneidae||BOLD:ACX5040
Araneidae|[3799]|GMAGV090-15|Araneae|Araneidae||BOLD:ACX6026
Araneidae|[3800]|GMARA983-14|Araneae|Araneidae||BOLD:ACN1213
Araneidae|[3801]|GMAGL1566-15|Araneae|Araneidae||BOLD:ACX6649
Araneidae|[3802]|GMAGT061-15|Araneae|Araneidae||BOLD:ACX7633
Araneidae|[3803]|GMAGX001-15|Araneae|Araneidae||BOLD:ACY2750
Araneidae|[3804]|GMARJ1893-14|Araneae|Araneidae||BOLD:ACN1109
Araneidae|[3805]|GMAGY520-15|Araneae|Araneidae||BOLD:ACX1614
Araneidae|[3806]|GMARF1355-14|Araneae|Araneidae||BOLD:ACN0938
Araneidae|[3807]|GMARV230-14|Araneae|Araneidae||BOLD:ACO0604
Araneidae|[3808]|GMARR1374-14|Araneae|Araneidae||BOLD:ACN8430
Araneidae|[3809]|GMAGX238-15|Araneae|Araneidae||BOLD:ACX2453
Araneae|[3810]|GMAGS1410-15|Araneae|||BOLD:ACX5918
Linyphiidae|[3811]|GMAGI1222-15|Araneae|Linyphiidae||BOLD:ACW3399
Linyphiidae|[3812]|GMARL1638-14|Araneae|Linyphiidae||BOLD:ACN1943
Linyphiidae|[3813]|GMAGU1028-15|Araneae|Linyphiidae||BOLD:ACX3019
Theridiidae|[3814]|GMAGB1014-15|Araneae|Theridiidae||BOLD:ACN1314
Araneae|[3815]|GMARW159-15|Araneae|||BOLD:ACX3990
Pseudoscorpiones|[3816]|GMAGV952-15|Pseudoscorpiones|||BOLD:ACX4337
Pseudoscorpiones|[3817]|GMAGW522-15|Pseudoscorpiones|||BOLD:ACX2931
Mesostigmata|[3818]|GMARQ778-14|Mesostigmata|||BOLD:ACN8896
Mesostigmata|[3819]|GMARB181-14|Mesostigmata|||BOLD:ACM5139
Parasitidae|[3820]|GMARK2524-14|Mesostigmata|Parasitidae||BOLD:ACN2103
Mesostigmata|[3821]|GMARU712-14|Mesostigmata|||BOLD:ACO1154
Mesostigmata|[3822]|GMAGF798-15|Mesostigmata|||BOLD:ACW3862
Mesostigmata|[3823]|GMAGO176-15|Mesostigmata|||BOLD:ACX5840
Mesostigmata|[3824]|GMAGF792-15|Mesostigmata|||BOLD:ACW3302
Arachnida|[3825]|GMARE1348-14||||BOLD:ACY4903
Mesostigmata|[3826]|GMAGE2909-15|Mesostigmata|||BOLD:ACW6850
Mesostigmata|[3827]|GMAGF799-15|Mesostigmata|||BOLD:ACW2890
Blattisociidae|[3828]|GMARK2525-14|Mesostigmata|Blattisociidae||BOLD:ACN2573
Mesostigmata|[3829]|GMAGL1595-15|Mesostigmata|||BOLD:ACW6588
Mesostigmata|[3830]|GMAGC507-15|Mesostigmata|||BOLD:ACW5551
Mesostigmata|[3831]|GMAGC504-15|Mesostigmata|||BOLD:ACW2206
Mesostigmata|[3832]|GMARB189-14|Mesostigmata|||BOLD:ACM4921
Mesostigmata|[3833]|GMAGF796-15|Mesostigmata|||BOLD:ACW3457
Mesostigmata|[3834]|GMAGF807-15|Mesostigmata|||BOLD:ACW2992
Mesostigmata|[3835]|GMAGG392-15|Mesostigmata|||BOLD:ACX5274
Mesostigmata|[3836]|GMAGM1922-15|Mesostigmata|||BOLD:ACN2882
Mesostigmata|[3837]|GMAGO211-15|Mesostigmata|||BOLD:ACX5826
Mesostigmata|[3838]|GMAGE2897-15|Mesostigmata|||BOLD:ACW6676
Mesostigmata|[3839]|GMART1632-14|Mesostigmata|||BOLD:ACN9522
Mesostigmata|[3840]|GMAGG404-15|Mesostigmata|||BOLD:ACX9563
Arachnida|[3841]|GMAGH1116-15||||BOLD:ACW3948
Ameroseiidae|[3842]|GMARB188-14|Mesostigmata|Ameroseiidae||BOLD:ACM5938
Mesostigmata|[3843]|GMAGE2894-15|Mesostigmata|||BOLD:ACW6566
Mesostigmata|[3844]|GMARU706-14|Mesostigmata|||BOLD:ACO1130
Mesostigmata|[3845]|GMAGT1231-15|Mesostigmata|||BOLD:ACX6003
Mesostigmata|[3846]|GMAGL1598-15|Mesostigmata|||BOLD:ACY0534
Mesostigmata|[3847]|GMART1637-14|Mesostigmata|||BOLD:ACN9355
Arachnida|[3848]|GMAGL1596-15||||BOLD:ACX3766
Ceratopogonidae|[3849]|GMARF768-14|Diptera|Ceratopogonidae||BOLD:ACM9537
Ceratopogonidae|[3850]|GMAGG1862-15|Diptera|Ceratopogonidae||BOLD:ACN0842
Ceratopogonidae|[3851]|GMAGL1884-15|Diptera|Ceratopogonidae||BOLD:ACN1807
Ceratopogonidae|[3852]|GMAGM907-15|Diptera|Ceratopogonidae||BOLD:ACX3524
Ceratopogonidae|[3853]|GMAGF327-15|Diptera|Ceratopogonidae||BOLD:ACW3922
Ceratopogonidae|[3854]|GMARL1187-14|Diptera|Ceratopogonidae||BOLD:ACN2035
Ceratopogonidae|[3855]|GMAGP1175-15|Diptera|Ceratopogonidae||BOLD:ACX5128
Ceratopogonidae|[3856]|GMARK1141-14|Diptera|Ceratopogonidae||BOLD:ACM9906
Ceratopogonidae|[3857]|GMAGS480-15|Diptera|Ceratopogonidae||BOLD:ACX4369
Ceratopogonidae|[3858]|GMARA1449-14|Diptera|Ceratopogonidae||BOLD:ACM6052
Ceratopogonidae|[3859]|GMAGN1316-15|Diptera|Ceratopogonidae||BOLD:ACX6111
Ceratopogonidae|[3860]|GMAGE937-15|Diptera|Ceratopogonidae||BOLD:ACN0426
Ceratopogonidae|[3861]|GMARE823-14|Diptera|Ceratopogonidae||BOLD:ACN0711
Ceratopogonidae|[3862]|GMARL098-14|Diptera|Ceratopogonidae||BOLD:ACN2034
Ceratopogonidae|[3863]|GMARD872-14|Diptera|Ceratopogonidae||BOLD:ACM8540
Ceratopogonidae|[3864]|GMAGE1013-15|Diptera|Ceratopogonidae||BOLD:ACW6164
Ceratopogonidae|[3865]|GMAGJ1259-15|Diptera|Ceratopogonidae||BOLD:ACN8076
Ceratopogonidae|[3866]|GMAGJ498-15|Diptera|Ceratopogonidae||BOLD:ACW9384
Ceratopogonidae|[3867]|GMARL374-14|Diptera|Ceratopogonidae||BOLD:ACN4405
Ceratopogonidae|[3868]|GMARP765-14|Diptera|Ceratopogonidae||BOLD:ACO3631
Ceratopogonidae|[3869]|GMART924-14|Diptera|Ceratopogonidae||BOLD:ACO0031
Ceratopogonidae|[3870]|GMARM367-14|Diptera|Ceratopogonidae||BOLD:ACN2499
Ceratopogonidae|[3871]|GMAGL935-15|Diptera|Ceratopogonidae||BOLD:ACN0361
Ceratopogonidae|[3872]|GMAGN2148-15|Diptera|Ceratopogonidae||BOLD:ACX2153
Ceratopogonidae|[3873]|GMARK1835-14|Diptera|Ceratopogonidae||BOLD:ACN1719
Ceratopogonidae|[3874]|GMART096-14|Diptera|Ceratopogonidae||BOLD:ACN9778
Ceratopogonidae|[3875]|GMAGD453-15|Diptera|Ceratopogonidae||BOLD:ACM7187
Ceratopogonidae|[3876]|GMARL1034-14|Diptera|Ceratopogonidae||BOLD:ACN2278
Ceratopogonidae|[3877]|GMARB1047-14|Diptera|Ceratopogonidae||BOLD:ACN0790
Ceratopogonidae|[3878]|GMAGJ1119-15|Diptera|Ceratopogonidae||BOLD:ACW9111
Ceratopogonidae|[3879]|GMARE470-14|Diptera|Ceratopogonidae||BOLD:ACM8119
Ceratopogonidae|[3880]|GMAGN1053-15|Diptera|Ceratopogonidae||BOLD:ACY5450
Ceratopogonidae|[3881]|GMARR674-14|Diptera|Ceratopogonidae||BOLD:ACN8358
Ceratopogonidae|[3882]|GMAGK296-15|Diptera|Ceratopogonidae||BOLD:ACN0723
Ceratopogonidae|[3883]|GMAGL1998-15|Diptera|Ceratopogonidae||BOLD:ACW6749

Ceratopogonidae|[3881]|GMARR674-14|Diptera|Ceratopogonidae||BOLD:ACN8358
Ceratopogonidae|[3882]|GMAGK296-15|Diptera|Ceratopogonidae||BOLD:ACN0723
Ceratopogonidae|[3883]|GMAGL1998-15|Diptera|Ceratopogonidae||BOLD:ACW6749
Ceratopogonidae|[3884]|GMARE904-14|Diptera|Ceratopogonidae||BOLD:ACM9035
Ceratopogonidae|[3885]|GMAGN1499-15|Diptera|Ceratopogonidae||BOLD:ACN7767
Ceratopogonidae|[3886]|GMARK1283-14|Diptera|Ceratopogonidae||BOLD:ACN2391
Ceratopogonidae|[3887]|GMARB1026-14|Diptera|Ceratopogonidae||BOLD:ACM6565
Ceratopogonidae|[3888]|GMAGB264-15|Diptera|Ceratopogonidae||BOLD:ACW2747
Ceratopogonidae|[3889]|GMAGG1174-15|Diptera|Ceratopogonidae||BOLD:ACW3865
Ceratopogonidae|[3890]|GMARI1871-14|Diptera|Ceratopogonidae||BOLD:ACN0774
Ceratopogonidae|[3891]|GMARB583-14|Diptera|Ceratopogonidae||BOLD:ACM6326
Ceratopogonidae|[3892]|GMARI174-14|Diptera|Ceratopogonidae||BOLD:ACN1686
Ceratopogonidae|[3893]|GMARN1370-14|Diptera|Ceratopogonidae||BOLD:ACX1699
Ceratopogonidae|[3894]|GMAGP631-15|Diptera|Ceratopogonidae||BOLD:ACN8495
Ceratopogonidae|[3895]|GMARN619-14|Diptera|Ceratopogonidae||BOLD:ACO0310
Ceratopogonidae|[3896]|GMAGT430-15|Diptera|Ceratopogonidae||BOLD:ACX5355
Ceratopogonidae|[3897]|GMAGT310-15|Diptera|Ceratopogonidae||BOLD:ACX5109
Ceratopogonidae|[3898]|GMARL483-14|Diptera|Ceratopogonidae||BOLD:ACX1110
Ceratopogonidae|[3899]|GMARL869-14|Diptera|Ceratopogonidae||BOLD:ACN2005
Ceratopogonidae|[3900]|GMAGT335-15|Diptera|Ceratopogonidae||BOLD:ACX4406
Ceratopogonidae|[3901]|GMAGM1969-15|Diptera|Ceratopogonidae||BOLD:ACO0656
Ceratopogonidae|[3902]|GMAGG1568-15|Diptera|Ceratopogonidae||BOLD:ACW3048
Ceratopogonidae|[3903]|GMARG758-14|Diptera|Ceratopogonidae||BOLD:ACN0033
Ceratopogonidae|[3904]|GMARS321-14|Diptera|Ceratopogonidae||BOLD:ACN7988
Ceratopogonidae|[3905]|GMAGD703-15|Diptera|Ceratopogonidae||BOLD:ACY8514
Ceratopogonidae|[3906]|GMARH310-14|Diptera|Ceratopogonidae||BOLD:ACN1485
Ceratopogonidae|[3907]|GMAGL2162-15|Diptera|Ceratopogonidae||BOLD:ACN2813
Ceratopogonidae|[3908]|GMARL994-14|Diptera|Ceratopogonidae||BOLD:ACN2463
Ceratopogonidae|[3909]|GMARD1172-14|Diptera|Ceratopogonidae||BOLD:ACM9142
Ceratopogonidae|[3910]|GMARD090-14|Diptera|Ceratopogonidae||BOLD:ACM7507
Ceratopogonidae|[3911]|GMAGA691-15|Diptera|Ceratopogonidae||BOLD:ACM4940
Ceratopogonidae|[3912]|GMAGN2240-15|Diptera|Ceratopogonidae||BOLD:ACX2344
Ceratopogonidae|[3913]|GMARO758-14|Diptera|Ceratopogonidae||BOLD:ACO0108
Ceratopogonidae|[3914]|GMAGI388-15|Diptera|Ceratopogonidae||BOLD:ACW2902
Ceratopogonidae|[3915]|GMARG1293-14|Diptera|Ceratopogonidae||BOLD:ACN0150
Ceratopogonidae|[3916]|GMARN624-14|Diptera|Ceratopogonidae||BOLD:ACN9817
Ceratopogonidae|[3917]|GMARL250-14|Diptera|Ceratopogonidae||BOLD:ACX1980
Ceratopogonidae|[3918]|GMAGT724-15|Diptera|Ceratopogonidae||BOLD:ACX6283
Ceratopogonidae|[3919]|GMAGQ1807-15|Diptera|Ceratopogonidae||BOLD:ACW4243
Ceratopogonidae|[3920]|GMART690-14|Diptera|Ceratopogonidae||BOLD:ACO0284
Ceratopogonidae|[3921]|GMARE862-14|Diptera|Ceratopogonidae||BOLD:ACN1403
Ceratopogonidae|[3922]|GMAGS736-15|Diptera|Ceratopogonidae||BOLD:ACN7389
Ceratopogonidae|[3923]|GMAGE702-15|Diptera|Ceratopogonidae||BOLD:ACN4530
Ceratopogonidae|[3924]|GMAGD649-15|Diptera|Ceratopogonidae||BOLD:ACO3458
Ceratopogonidae|[3925]|GMAGT291-15|Diptera|Ceratopogonidae||BOLD:ACX4158
Ceratopogonidae|[3926]|GMARD2164-14|Diptera|Ceratopogonidae||BOLD:ACN1069
Ceratopogonidae|[3927]|GMAGQ1004-15|Diptera|Ceratopogonidae||BOLD:ACN5591
Ceratopogonidae|[3928]|GMART502-14|Diptera|Ceratopogonidae||BOLD:ACN9109
Ceratopogonidae|[3929]|GMAGH335-15|Diptera|Ceratopogonidae||BOLD:ACN5318
Ceratopogonidae|[3930]|GMARA1395-14|Diptera|Ceratopogonidae||BOLD:ACM9512
Ceratopogonidae|[3931]|GMAGP640-15|Diptera|Ceratopogonidae||BOLD:ACX4254
Ceratopogonidae|[3932]|GMARG1194-14|Diptera|Ceratopogonidae||BOLD:ACN1280
Ceratopogonidae|[3933]|GMAGE2038-15|Diptera|Ceratopogonidae||BOLD:ACN1929
Ceratopogonidae|[3934]|GMARD1536-14|Diptera|Ceratopogonidae||BOLD:ACN0883
Ceratopogonidae|[3935]|GMARK1393-14|Diptera|Ceratopogonidae||BOLD:ACN1028
Ceratopogonidae|[3936]|GMAGN1700-15|Diptera|Ceratopogonidae||BOLD:ACN0304
Ceratopogonidae|[3937]|GMARK1971-14|Diptera|Ceratopogonidae||BOLD:ACN1866
Ceratopogonidae|[3938]|GMAGN1952-15|Diptera|Ceratopogonidae||BOLD:ACX2675
Ceratopogonidae|[3939]|GMAGP1742-15|Diptera|Ceratopogonidae||BOLD:ACX4681
Ceratopogonidae|[3940]|GMARM844-14|Diptera|Ceratopogonidae||BOLD:ACN3660
Ceratopogonidae|[3941]|GMAGK1692-15|Diptera|Ceratopogonidae||BOLD:ACX0164
Cecidomyiidae|[3942]|GMARA312-14|Diptera|Cecidomyiidae||BOLD:ACM5811
Ceratopogonidae|[3943]|GMARD110-14|Diptera|Ceratopogonidae||BOLD:ACN0462
Ceratopogonidae|[3944]|GMAGE839-15|Diptera|Ceratopogonidae||BOLD:ACN1816
Ceratopogonidae|[3945]|GMAGM712-15|Diptera|Ceratopogonidae||BOLD:ACX3380
Ceratopogonidae|[3946]|GMARD2118-14|Diptera|Ceratopogonidae||BOLD:ACN0851
Ceratopogonidae|[3947]|GMAGD1203-15|Diptera|Ceratopogonidae||BOLD:ACW6906
Ceratopogonidae|[3948]|GMAGE2204-15|Diptera|Ceratopogonidae||BOLD:ACM9874
Ceratopogonidae|[3949]|GMAGH1195-15|Diptera|Ceratopogonidae||BOLD:ACM9141
Ceratopogonidae|[3950]|GMARM873-14|Diptera|Ceratopogonidae||BOLD:ACN6370
Ceratopogonidae|[3951]|GMAGJ1276-15|Diptera|Ceratopogonidae||BOLD:ACW8615
Ceratopogonidae|[3952]|GMARD995-14|Diptera|Ceratopogonidae||BOLD:ACM4834
Ceratopogonidae|[3953]|GMARN821-14|Diptera|Ceratopogonidae||BOLD:ACN3700
Ceratopogonidae|[3954]|GMAGA618-15|Diptera|Ceratopogonidae||BOLD:ACM6544
Ceratopogonidae|[3955]|GMARD1286-14|Diptera|Ceratopogonidae||BOLD:ACM8148
Ceratopogonidae|[3956]|GMAGD822-15|Diptera|Ceratopogonidae||BOLD:ACM7154
Ceratopogonidae|[3957]|GMAGB467-15|Diptera|Ceratopogonidae||BOLD:ACM4993
Ceratopogonidae|[3958]|GMAGB249-15|Diptera|Ceratopogonidae||BOLD:ACM5410
Ceratopogonidae|[3959]|GMARD1440-14|Diptera|Ceratopogonidae||BOLD:ACW3506
Ceratopogonidae|[3960]|GMAGF291-15|Diptera|Ceratopogonidae||BOLD:ACW4020
Ceratopogonidae|[3961]|GMAGH412-15|Diptera|Ceratopogonidae||BOLD:ACW1995
Ceratopogonidae|[3962]|GMAGK1687-15|Diptera|Ceratopogonidae||BOLD:ACN1727
Ceratopogonidae|[3963]|GMAGF194-15|Diptera|Ceratopogonidae||BOLD:ACW3913
Ceratopogonidae|[3964]|GMAGH511-15|Diptera|Ceratopogonidae||BOLD:ACW1927
Ceratopogonidae|[3965]|GMAGR868-15|Diptera|Ceratopogonidae||BOLD:ACX5394
Ceratopogonidae|[3966]|GMARH075-14|Diptera|Ceratopogonidae||BOLD:ACN1356
Ceratopogonidae|[3967]|GMAGP883-15|Diptera|Ceratopogonidae||BOLD:ACN7525
Ceratopogonidae|[3968]|GMARR436-14|Diptera|Ceratopogonidae||BOLD:ACN7721
Ceratopogonidae|[3969]|GMAGA437-15|Diptera|Ceratopogonidae||BOLD:ACM6228
Ceratopogonidae|[3970]|GMAGC1025-15|Diptera|Ceratopogonidae||BOLD:ACM4829
Ceratopogonidae|[3971]|GMAGD306-15|Diptera|Ceratopogonidae||BOLD:ACN1255
Ceratopogonidae|[3972]|GMARA316-14|Diptera|Ceratopogonidae||BOLD:ABW7565
Ceratopogonidae|[3973]|GMARD1262-14|Diptera|Ceratopogonidae||BOLD:ADB3920
Ceratopogonidae|[3974]|GMARA254-14|Diptera|Ceratopogonidae||BOLD:ACM6545
Ceratopogonidae|[3975]|GMARA118-14|Diptera|Ceratopogonidae||BOLD:ACM9691
Ceratopogonidae|[3976]|GMAGB230-15|Diptera|Ceratopogonidae||BOLD:ACM8272
Ceratopogonidae|[3977]|GMAGL169-15|Diptera|Ceratopogonidae||BOLD:ACX4775
Ceratopogonidae|[3978]|GMAGA607-15|Diptera|Ceratopogonidae||BOLD:ACM5850
Ceratopogonidae|[3979]|GMAGK346-15|Diptera|Ceratopogonidae||BOLD:ACX5161
Ceratopogonidae|[3980]|GMARA1490-14|Diptera|Ceratopogonidae||BOLD:ACM6056
Ceratopogonidae|[3981]|GMARH174-14|Diptera|Ceratopogonidae||BOLD:ACN0848
Ceratopogonidae|[3982]|GMARD817-14|Diptera|Ceratopogonidae||BOLD:ACM9581
Ceratopogonidae|[3983]|GMAGL938-15|Diptera|Ceratopogonidae||BOLD:ACW7084

Ceratopogonidae|[3980]|GMARA1490-14|Diptera|Ceratopogonidae||BOLD:ACM[illegible]
Ceratopogonidae|[3981]|GMARH174-14|Diptera|Ceratopogonidae||BOLD:ACN0848
Ceratopogonidae|[3982]|GMARD817-14|Diptera|Ceratopogonidae||BOLD:ACM9581
Ceratopogonidae|[3983]|GMAGL938-15|Diptera|Ceratopogonidae||BOLD:ACW7084
Ceratopogonidae|[3984]|GMARI924-14|Diptera|Ceratopogonidae||BOLD:ACM9996
Ceratopogonidae|[3985]|GMARA1107-14|Diptera|Ceratopogonidae||BOLD:ACM8374
Ceratopogonidae|[3986]|GMAGB286-15|Diptera|Ceratopogonidae||BOLD:ACN0591
Ceratopogonidae|[3987]|GMAGM672-15|Diptera|Ceratopogonidae||BOLD:ACN3127
Ceratopogonidae|[3988]|GMAGN1830-15|Diptera|Ceratopogonidae||BOLD:ACN2514
Ceratopogonidae|[3989]|GMAGN1738-15|Diptera|Ceratopogonidae||BOLD:ACX4762
Ceratopogonidae|[3990]|GMARF578-14|Diptera|Ceratopogonidae||BOLD:ACM8388
Ceratopogonidae|[3991]|GMARM1165-14|Diptera|Ceratopogonidae||BOLD:ACX4764
Ceratopogonidae|[3992]|GMARD2149-14|Diptera|Ceratopogonidae||BOLD:ACX5724
Ceratopogonidae|[3993]|GMARN1877-14|Diptera|Ceratopogonidae||BOLD:ACX6030
Ceratopogonidae|[3994]|GMAGT735-15|Diptera|Ceratopogonidae||BOLD:ACX5723
Ceratopogonidae|[3995]|GMAGA580-15|Diptera|Ceratopogonidae||BOLD:ACN8118
Ceratopogonidae|[3996]|GMARR136-14|Diptera|Ceratopogonidae||BOLD:ACN8926
Ceratopogonidae|[3997]|GMARB166-14|Diptera|Ceratopogonidae||BOLD:ACM6034
Ceratopogonidae|[3998]|GMARK1919-14|Diptera|Ceratopogonidae||BOLD:ACN0179
Ceratopogonidae|[3999]|GMARN1094-14|Diptera|Ceratopogonidae||BOLD:ACX7092
Ceratopogonidae|[4000]|GMART1061-14|Diptera|Ceratopogonidae||BOLD:ACO0550
Ceratopogonidae|[4001]|GMARA307-14|Diptera|Ceratopogonidae||BOLD:ACM5172
Ceratopogonidae|[4002]|GMAGF120-15|Diptera|Ceratopogonidae||BOLD:ACW3686
Ceratopogonidae|[4003]|GMARC159-14|Diptera|Ceratopogonidae||BOLD:ACM7289
Ceratopogonidae|[4004]|GMAGG1247-15|Diptera|Ceratopogonidae||BOLD:ACN1089
Ceratopogonidae|[4005]|GMAGG1668-15|Diptera|Ceratopogonidae||BOLD:ACW6235
Ceratopogonidae|[4006]|GMAGE1088-15|Diptera|Ceratopogonidae||BOLD:ACW1747
Ceratopogonidae|[4007]|GMARH1118-14|Diptera|Ceratopogonidae||BOLD:ACN0256
Ceratopogonidae|[4008]|GMAGE175-15|Diptera|Ceratopogonidae||BOLD:ACW3437
Ceratopogonidae|[4009]|GMAGO466-15|Diptera|Ceratopogonidae||BOLD:ACO1837
Ceratopogonidae|[4010]|GMARU111-14|Diptera|Ceratopogonidae||BOLD:ACO0757
Ceratopogonidae|[4011]|GMAGB411-15|Diptera|Ceratopogonidae||BOLD:ACN0095
Ceratopogonidae|[4012]|GMARF1102-14|Diptera|Ceratopogonidae||BOLD:ACM8835
Ceratopogonidae|[4013]|GMARB1053-14|Diptera|Ceratopogonidae||BOLD:ACM5693
Ceratopogonidae|[4014]|GMAGD827-15|Diptera|Ceratopogonidae||BOLD:ACW5353
Ceratopogonidae|[4015]|GMARB1424-14|Diptera|Ceratopogonidae||BOLD:ACM5204
Ceratopogonidae|[4016]|GMAGQ993-15|Diptera|Ceratopogonidae||BOLD:ACO1277
Ceratopogonidae|[4017]|GMARA1480-14|Diptera|Ceratopogonidae||BOLD:ACM5725
Ceratopogonidae|[4018]|GMAGC031-15|Diptera|Ceratopogonidae||BOLD:ACM8169
Ceratopogonidae|[4019]|GMAGE1026-15|Diptera|Ceratopogonidae||BOLD:ACO0503
Ceratopogonidae|[4020]|GMARL459-14|Diptera|Ceratopogonidae||BOLD:ACN4489
Ceratopogonidae|[4021]|GMAGS922-15|Diptera|Ceratopogonidae||BOLD:ACX2284
Ceratopogonidae|[4022]|GMAGH275-15|Diptera|Ceratopogonidae||BOLD:ACW1338
Ceratopogonidae|[4023]|GMAGN2263-15|Diptera|Ceratopogonidae||BOLD:ACX1407
Ceratopogonidae|[4024]|GMAGN1832-15|Diptera|Ceratopogonidae||BOLD:ACN7482
Ceratopogonidae|[4025]|GMARK2051-14|Diptera|Ceratopogonidae||BOLD:ACN2600
Ceratopogonidae|[4026]|GMARM639-14|Diptera|Ceratopogonidae||BOLD:ACN9247
Ceratopogonidae|[4027]|GMARJ1481-14|Diptera|Ceratopogonidae||BOLD:ACN0042
Ceratopogonidae|[4028]|GMAGH670-15|Diptera|Ceratopogonidae||BOLD:ACN0596
Ceratopogonidae|[4029]|GMAGO655-15|Diptera|Ceratopogonidae||BOLD:ACN6351
Ceratopogonidae|[4030]|GMAGJ480-15|Diptera|Ceratopogonidae||BOLD:ACN0104
Ceratopogonidae|[4031]|GMAGF297-15|Diptera|Ceratopogonidae||BOLD:ACW3558
Ceratopogonidae|[4032]|GMAGH564-15|Diptera|Ceratopogonidae||BOLD:ACW2638
Ceratopogonidae|[4033]|GMAGN128-15|Diptera|Ceratopogonidae||BOLD:ACN2773
Ceratopogonidae|[4034]|GMAGJ1188-15|Diptera|Ceratopogonidae||BOLD:ACN0068
Ceratopogonidae|[4035]|GMAGB1042-15|Diptera|Ceratopogonidae||BOLD:ACM7619
Ceratopogonidae|[4036]|GMARI966-14|Diptera|Ceratopogonidae||BOLD:ACN1768
Ceratopogonidae|[4037]|GMAGQ1745-15|Diptera|Ceratopogonidae||BOLD:ACX6154
Ceratopogonidae|[4038]|GMAGN1944-15|Diptera|Ceratopogonidae||BOLD:ACX3031
Ceratopogonidae|[4039]|GMAGN1237-15|Diptera|Ceratopogonidae||BOLD:ACX3734
Ceratopogonidae|[4040]|GMAGJ338-15|Diptera|Ceratopogonidae||BOLD:ACW8185
Ceratopogonidae|[4041]|GMARB800-14|Diptera|Ceratopogonidae||BOLD:ACM4957
Ceratopogonidae|[4042]|GMAGA660-15|Diptera|Ceratopogonidae||BOLD:ACM5233
Ceratopogonidae|[4043]|GMARB1532-14|Diptera|Ceratopogonidae||BOLD:ACM4726
Ceratopogonidae|[4044]|GMAGK399-15|Diptera|Ceratopogonidae||BOLD:ACO0704
Ceratopogonidae|[4045]|GMAGL034-15|Diptera|Ceratopogonidae||BOLD:ACN0520
Ceratopogonidae|[4046]|GMAGC634-15|Diptera|Ceratopogonidae||BOLD:ACM8167
Ceratopogonidae|[4047]|GMAGA614-15|Diptera|Ceratopogonidae||BOLD:ACM9973
Ceratopogonidae|[4048]|GMAGK520-15|Diptera|Ceratopogonidae||BOLD:ACX5996
Ceratopogonidae|[4049]|GMARK1433-14|Diptera|Ceratopogonidae||BOLD:ACN2075
Ceratopogonidae|[4050]|GMARQ361-14|Diptera|Ceratopogonidae||BOLD:ACN8327
Ceratopogonidae|[4051]|GMARL808-14|Diptera|Ceratopogonidae||BOLD:ACN2781
Ceratopogonidae|[4052]|GMAGR755-15|Diptera|Ceratopogonidae||BOLD:ACN8213
Ceratopogonidae|[4053]|GMAGE1699-15|Diptera|Ceratopogonidae||BOLD:ACN2055
Ceratopogonidae|[4054]|GMAGH718-15|Diptera|Ceratopogonidae||BOLD:ACW4844
Ceratopogonidae|[4055]|GMAGG2314-15|Diptera|Ceratopogonidae||BOLD:ACM9312
Ceratopogonidae|[4056]|GMAGJ1320-15|Diptera|Ceratopogonidae||BOLD:ACN0209
Ceratopogonidae|[4057]|GMARO675-14|Diptera|Ceratopogonidae||BOLD:ACN9568
Ceratopogonidae|[4058]|GMAGJ248-15|Diptera|Ceratopogonidae||BOLD:ACN2446
Ceratopogonidae|[4059]|GMARO509-14|Diptera|Ceratopogonidae||BOLD:ACN4586
Ceratopogonidae|[4060]|GMAGS585-15|Diptera|Ceratopogonidae||BOLD:ACX4085
Ceratopogonidae|[4061]|GMARP663-14|Diptera|Ceratopogonidae||BOLD:ACN9104
Ceratopogonidae|[4062]|GMAGG1603-15|Diptera|Ceratopogonidae||BOLD:ACW7698
Ceratopogonidae|[4063]|GMARJ1650-14|Diptera|Ceratopogonidae||BOLD:ACN0611
Ceratopogonidae|[4064]|GMARQ265-14|Diptera|Ceratopogonidae||BOLD:ACN7551
Ceratopogonidae|[4065]|GMAGE304-15|Diptera|Ceratopogonidae||BOLD:ACW8026
Ceratopogonidae|[4066]|GMARJ078-14|Diptera|Ceratopogonidae||BOLD:ACN1290
Ceratopogonidae|[4067]|GMARA1098-14|Diptera|Ceratopogonidae||BOLD:ACM8211
Ceratopogonidae|[4068]|GMARM614-14|Diptera|Ceratopogonidae||BOLD:ACN9748
Ceratopogonidae|[4069]|GMAGE761-15|Diptera|Ceratopogonidae||BOLD:ACM9857
Ceratopogonidae|[4070]|GMARG389-14|Diptera|Ceratopogonidae||BOLD:ACN0046
Ceratopogonidae|[4071]|GMAGC878-15|Diptera|Ceratopogonidae||BOLD:ACW1962
Ceratopogonidae|[4072]|GMARE297-14|Diptera|Ceratopogonidae||BOLD:ACN0898
Ceratopogonidae|[4073]|GMART1215-14|Diptera|Ceratopogonidae||BOLD:ACO1265
Ceratopogonidae|[4074]|GMARC716-14|Diptera|Ceratopogonidae||BOLD:ACX2679
Ceratopogonidae|[4075]|GMAGH578-15|Diptera|Ceratopogonidae||BOLD:ACN7809
Ceratopogonidae|[4076]|GMAGR1003-15|Diptera|Ceratopogonidae||BOLD:ACX4614
Ceratopogonidae|[4077]|GMAGE152-15|Diptera|Ceratopogonidae||BOLD:ACW4730
Ceratopogonidae|[4078]|GMARU480-14|Diptera|Ceratopogonidae||BOLD:ACO1051
Ceratopogonidae|[4079]|GMAGQ1841-15|Diptera|Ceratopogonidae||BOLD:ACN9740
Ceratopogonidae|[4080]|GMAGR679-15|Diptera|Ceratopogonidae||BOLD:ACN8778
Ceratopogonidae|[4081]|GMAGS418-15|Diptera|Ceratopogonidae||BOLD:ACX5319
Ceratopogonidae|[4082]|GMAGG1479-15|Diptera|Ceratopogonidae||BOLD:ACW3393

Ceratopogonidae|[4080]|GMAGR679-15|Diptera|Ceratopogonidae||BOLD:ACN8778
Ceratopogonidae|[4081]|GMAGS418-15|Diptera|Ceratopogonidae||BOLD:ACX5319
Ceratopogonidae|[4082]|GMAGG1479-15|Diptera|Ceratopogonidae||BOLD:ACW3393
Ceratopogonidae|[4083]|GMARN549-14|Diptera|Ceratopogonidae||BOLD:ACO0297
Ceratopogonidae|[4084]|GMARP630-14|Diptera|Ceratopogonidae||BOLD:ACN8154
Ceratopogonidae|[4085]|GMAGQ1911-15|Diptera|Ceratopogonidae||BOLD:ACO0863
Ceratopogonidae|[4086]|GMAGY434-15|Diptera|Ceratopogonidae||BOLD:ACW3734
Ceratopogonidae|[4087]|GMARD804-14|Diptera|Ceratopogonidae||BOLD:ACM9027
Ceratopogonidae|[4088]|GMAGJ521-15|Diptera|Ceratopogonidae||BOLD:ACW8900
Ceratopogonidae|[4089]|GMAGJ1206-15|Diptera|Ceratopogonidae||BOLD:ACN1481
Ceratopogonidae|[4090]|GMARG102-14|Diptera|Ceratopogonidae||BOLD:ACN1883
Ceratopogonidae|[4091]|GMAGZ100-15|Diptera|Ceratopogonidae||BOLD:ACX3381
Ceratopogonidae|[4092]|GMAGM822-15|Diptera|Ceratopogonidae||BOLD:ACN7554
Ceratopogonidae|[4093]|GMARM342-14|Diptera|Ceratopogonidae||BOLD:ACN2751
Ceratopogonidae|[4094]|GMAGH145-15|Diptera|Ceratopogonidae||BOLD:ACN2424
Ceratopogonidae|[4095]|GMAGQ1007-15|Diptera|Ceratopogonidae||BOLD:ACO1235
Ceratopogonidae|[4096]|GMAGL889-15|Diptera|Ceratopogonidae||BOLD:ACW6841
Ceratopogonidae|[4097]|GMAGN1527-15|Diptera|Ceratopogonidae||BOLD:ACW3016
Ceratopogonidae|[4098]|GMAGN1855-15|Diptera|Ceratopogonidae||BOLD:ACX2852
Ceratopogonidae|[4099]|GMAGQ1033-15|Diptera|Ceratopogonidae||BOLD:ACN6219
Ceratopogonidae|[4100]|GMARE948-14|Diptera|Ceratopogonidae||BOLD:ACX7298
Ceratopogonidae|[4101]|GMAGO435-15|Diptera|Ceratopogonidae||BOLD:ACX6009
Ceratopogonidae|[4102]|GMARN369-14|Diptera|Ceratopogonidae||BOLD:ACN6104
Ceratopogonidae|[4103]|GMAGP1528-15|Diptera|Ceratopogonidae||BOLD:ACO0478
Ceratopogonidae|[4104]|GMARM1123-14|Diptera|Ceratopogonidae||BOLD:ACN4438
Ceratopogonidae|[4105]|GMARR621-14|Diptera|Ceratopogonidae||BOLD:ACN7646
Ceratopogonidae|[4106]|GMAGD1727-15|Diptera|Ceratopogonidae||BOLD:ACW5132
Ceratopogonidae|[4107]|GMAGE1688-15|Diptera|Ceratopogonidae||BOLD:ACW2445
Ceratopogonidae|[4108]|GMAGG1259-15|Diptera|Ceratopogonidae||BOLD:ACW4279
Ceratopogonidae|[4109]|GMAGN2002-15|Diptera|Ceratopogonidae||BOLD:ACN1019
Ceratopogonidae|[4110]|GMAGP1478-15|Diptera|Ceratopogonidae||BOLD:ACO0590
Ceratopogonidae|[4111]|GMAGG1237-15|Diptera|Ceratopogonidae||BOLD:ACN1131
Ceratopogonidae|[4112]|GMAGQ1775-15|Diptera|Ceratopogonidae||BOLD:ACX6164
Ceratopogonidae|[4113]|GMARL096-14|Diptera|Ceratopogonidae||BOLD:ACN2553
Ceratopogonidae|[4114]|GMAGE947-15|Diptera|Ceratopogonidae||BOLD:ACW7637
Ceratopogonidae|[4115]|GMART440-14|Diptera|Ceratopogonidae||BOLD:ACO0412
Ceratopogonidae|[4116]|GMAGY158-15|Diptera|Ceratopogonidae||BOLD:ACX2214
Ceratopogonidae|[4117]|GMAGD973-15|Diptera|Ceratopogonidae||BOLD:ACN1354
Ceratopogonidae|[4118]|GMART1122-14|Diptera|Ceratopogonidae||BOLD:ACO0682
Ceratopogonidae|[4119]|GMAGG1910-15|Diptera|Ceratopogonidae||BOLD:ACO1204
Ceratopogonidae|[4120]|GMART532-14|Diptera|Ceratopogonidae||BOLD:ACN9044
Keroplatidae|[4121]|GMAGD200-15|Diptera|Keroplatidae||BOLD:ACN0189
Bibionidae|[4122]|GMAGA205-15|Diptera|Bibionidae||BOLD:ACN0750
Bibionidae|[4123]|GMAGP008-15|Diptera|Bibionidae||BOLD:ACX6982
Bibionidae|[4124]|GMARR1470-14|Diptera|Bibionidae||BOLD:ACO0027
Ceratopogonidae|[4125]|GMARM1156-14|Diptera|Ceratopogonidae||BOLD:ACO0263
Ceratopogonidae|[4126]|GMARE194-14|Diptera|Ceratopogonidae||BOLD:ACN0393
Ceratopogonidae|[4127]|GMARF206-14|Diptera|Ceratopogonidae||BOLD:ACM8943
Ceratopogonidae|[4128]|GMARQ422-14|Diptera|Ceratopogonidae||BOLD:ACN8016
Diptera|[4129]|GMAGH045-15|Diptera|||BOLD:ACN1253
Diptera|[4130]|GMARG764-14|Diptera|||BOLD:ACN0645
Ceratopogonidae|[4131]|GMAGA635-15|Diptera|Ceratopogonidae||BOLD:ACV2287
Diptera|[4132]|GMAGV223-15|Diptera|||BOLD:ACX1876
Psychodidae|[4133]|GMAGG2246-15|Diptera|Psychodidae||BOLD:ACN7519
Psychodidae|[4134]|GMARD145-14|Diptera|Psychodidae||BOLD:ACM9267
Psychodidae|[4135]|GMARB788-14|Diptera|Psychodidae||BOLD:ACM5455
Psychodidae|[4136]|GMAGS345-15|Diptera|Psychodidae||BOLD:ACX4251
Psychodidae|[4137]|GMAGU415-15|Diptera|Psychodidae||BOLD:ACX5852
Psychodidae|[4138]|GMAGK1609-15|Diptera|Psychodidae||BOLD:ACN8836
Psychodidae|[4139]|GMARI1793-14|Diptera|Psychodidae||BOLD:ACN0438
Psychodidae|[4140]|GMARB163-14|Diptera|Psychodidae||BOLD:ACM6465
Psychodidae|[4141]|GMARB344-14|Diptera|Psychodidae||BOLD:ACM5042
Psychodidae|[4142]|GMARB1586-14|Diptera|Psychodidae||BOLD:ACM4907
Psychodidae|[4143]|GMAGG1324-15|Diptera|Psychodidae||BOLD:ACN0235
Cecidomyiidae|[4144]|GMAGI757-15|Diptera|Cecidomyiidae||BOLD:ACY6557
Psychodidae|[4145]|GMAGJ1251-15|Diptera|Psychodidae||BOLD:ACW7173
Psychodidae|[4146]|GMAGL584-15|Diptera|Psychodidae||BOLD:ACN0801
Psychodidae|[4147]|GMARD944-14|Diptera|Psychodidae||BOLD:ACM9116
Psychodidae|[4148]|GMAGE1146-15|Diptera|Psychodidae||BOLD:ACN1858
Psychodidae|[4149]|GMAGI473-15|Diptera|Psychodidae||BOLD:ACW2863
Psychodidae|[4150]|GMAGN1878-15|Diptera|Psychodidae||BOLD:ACX2652
Psychodidae|[4151]|GMAGN1948-15|Diptera|Psychodidae||BOLD:ACN2356
Psychodidae|[4152]|GMAGJ1044-15|Diptera|Psychodidae||BOLD:ACN0607
Psychodidae|[4153]|GMAGN1803-15|Diptera|Psychodidae||BOLD:ACX2203
Psychodidae|[4154]|GMAGJ438-15|Diptera|Psychodidae||BOLD:ACN1483
Psychodidae|[4155]|GMARK806-14|Diptera|Psychodidae||BOLD:ACN0784
Psychodidae|[4156]|GMAGH287-15|Diptera|Psychodidae||BOLD:ACW1811
Psychodidae|[4157]|GMARD210-14|Diptera|Psychodidae||BOLD:ACN1154
Psychodidae|[4158]|GMAGI217-15|Diptera|Psychodidae||BOLD:ACW7000
Psychodidae|[4159]|GMAGH218-15|Diptera|Psychodidae||BOLD:ACN1323
Psychodidae|[4160]|GMARH239-14|Diptera|Psychodidae||BOLD:ACN0689
Psychodidae|[4161]|GMAGM142-15|Diptera|Psychodidae||BOLD:AAF9312
Psychodidae|[4162]|GMARF960-14|Diptera|Psychodidae||BOLD:ACM9681
Psychodidae|[4163]|GMAGN170-15|Diptera|Psychodidae||BOLD:ACX2869
Psychodidae|[4164]|GMAGG1346-15|Diptera|Psychodidae||BOLD:ACW3568
Psychodidae|[4165]|GMARM949-14|Diptera|Psychodidae||BOLD:ACN9697
Psychodidae|[4166]|GMAGA637-15|Diptera|Psychodidae||BOLD:ACV2396
Psychodidae|[4167]|GMAGK1703-15|Diptera|Psychodidae||BOLD:ACW9558
Psychodidae|[4168]|GMARQ469-14|Diptera|Psychodidae||BOLD:ACN8013
Psychodidae|[4169]|GMARR605-14|Diptera|Psychodidae||BOLD:ACN7428
Mycetophilidae|[4170]|GMAGK263-15|Diptera|Mycetophilidae||BOLD:ACX4622
Mycetophilidae|[4171]|GMAGN827-15|Diptera|Mycetophilidae||BOLD:ACX4708
Mycetophilidae|[4172]|GMARF260-14|Diptera|Mycetophilidae||BOLD:ACM8976
Mycetophilidae|[4173]|GMARL1208-14|Diptera|Mycetophilidae||BOLD:ACN2859
Mycetophilidae|[4174]|GMAGP921-15|Diptera|Mycetophilidae||BOLD:ACX4783
Syrphidae|[4175]|GMARL1781-14|Diptera|Syrphidae||BOLD:ACN4587
Mycetophilidae|[4176]|GMAGH308-15|Diptera|Mycetophilidae||BOLD:ACW2599
Mycetophilidae|[4177]|GMARI2179-14|Diptera|Mycetophilidae||BOLD:ACN0690
Mycetophilidae|[4178]|GMAGJ089-15|Diptera|Mycetophilidae||BOLD:ACN0551
Mycetophilidae|[4179]|GMAGK679-15|Diptera|Mycetophilidae||BOLD:ACN1899
Mycetophilidae|[4180]|GMAGD322-15|Diptera|Mycetophilidae||BOLD:ACM5607
Mycetophilidae|[4181]|GMARD290-14|Diptera|Mycetophilidae||BOLD:ACM8151
Mycetophilidae|[4182]|GMAGM258-15|Diptera|Mycetophilidae||BOLD:ACN9456

Mycetophilidae|[4180]|GMAGD322-15|Diptera|Mycetophilidae||BOLD:ACM5607
Mycetophilidae|[4181]|GMARD290-14|Diptera|Mycetophilidae||BOLD:ACM8151
Mycetophilidae|[4182]|GMAGM258-15|Diptera|Mycetophilidae||BOLD:ACN9456
Mycetophilidae|[4183]|GMARD338-14|Diptera|Mycetophilidae||BOLD:ACN0160
Mycetophilidae|[4184]|GMAGK240-15|Diptera|Mycetophilidae||BOLD:ACN2571
Mycetophilidae|[4185]|GMAGN710-15|Diptera|Mycetophilidae||BOLD:ACX4329
Mycetophilidae|[4186]|GMARD1026-14|Diptera|Mycetophilidae||BOLD:ACM8164
Mycetophilidae|[4187]|GMARB1188-14|Diptera|Mycetophilidae||BOLD:ACM5805
Mycetophilidae|[4188]|GMARD392-14|Diptera|Mycetophilidae||BOLD:ACM7418
Mycetophilidae|[4189]|GMAGK744-15|Diptera|Mycetophilidae||BOLD:ACN0449
Mycetophilidae|[4190]|GMAGC769-15|Diptera|Mycetophilidae||BOLD:ACM5678
Mycetophilidae|[4191]|GMARS302-14|Diptera|Mycetophilidae||BOLD:ACN8754
Mycetophilidae|[4192]|GMAGN823-15|Diptera|Mycetophilidae||BOLD:ACX5493
Mycetophilidae|[4193]|GMAGK177-15|Diptera|Mycetophilidae||BOLD:ACN0406
Mycetophilidae|[4194]|GMAGK609-15|Diptera|Mycetophilidae||BOLD:ACX5517
Mycetophilidae|[4195]|GMAGJ427-15|Diptera|Mycetophilidae||BOLD:ACN1475
Mycetophilidae|[4196]|GMAGN1353-15|Diptera|Mycetophilidae||BOLD:ACN1051
Mycetophilidae|[4197]|GMARK1275-14|Diptera|Mycetophilidae||BOLD:ACN2685
Mycetophilidae|[4198]|GMAGI457-15|Diptera|Mycetophilidae||BOLD:ACX3932
Mycetophilidae|[4199]|GMARF216-14|Diptera|Mycetophilidae||BOLD:ACM9203
Mycetophilidae|[4200]|GMARF1012-14|Diptera|Mycetophilidae||BOLD:ACM9919
Mycetophilidae|[4201]|GMARB1536-14|Diptera|Mycetophilidae||BOLD:ACM4727
Mycetophilidae|[4202]|GMAGK327-15|Diptera|Mycetophilidae||BOLD:ACO0626
Mycetophilidae|[4203]|GMARD1112-14|Diptera|Mycetophilidae||BOLD:ACM9826
Mycetophilidae|[4204]|GMARG045-14|Diptera|Mycetophilidae||BOLD:ACN1123
Mycetophilidae|[4205]|GMARP197-14|Diptera|Mycetophilidae||BOLD:ACN7870
Mycetophilidae|[4206]|GMARI370-14|Diptera|Mycetophilidae||BOLD:ACN0609
Mycetophilidae|[4207]|GMAGH175-15|Diptera|Mycetophilidae||BOLD:ACN0271
Mycetophilidae|[4208]|GMAGL150-15|Diptera|Mycetophilidae||BOLD:ACN2589
Mycetophilidae|[4209]|GMAGJ231-15|Diptera|Mycetophilidae||BOLD:ACW8091
Mycetophilidae|[4210]|GMAGN937-15|Diptera|Mycetophilidae||BOLD:ACX4872
Mycetophilidae|[4211]|GMARJ246-14|Diptera|Mycetophilidae||BOLD:ACN3958
Mycetophilidae|[4212]|GMAGJ210-15|Diptera|Mycetophilidae||BOLD:ACN0859
Mycetophilidae|[4213]|GMARH1262-14|Diptera|Mycetophilidae||BOLD:ACN0814
Mycetophilidae|[4214]|GMARD858-14|Diptera|Mycetophilidae||BOLD:ACM8983
Mycetophilidae|[4215]|GMAGN717-15|Diptera|Mycetophilidae||BOLD:ACN9583
Mycetophilidae|[4216]|GMAGG1133-15|Diptera|Mycetophilidae||BOLD:ACM7591
Mycetophilidae|[4217]|GMARG274-14|Diptera|Mycetophilidae||BOLD:ACN0610
Mycetophilidae|[4218]|GMAGI402-15|Diptera|Mycetophilidae||BOLD:ACN0538
Mycetophilidae|[4219]|GMAGL581-15|Diptera|Mycetophilidae||BOLD:ACX3658
Mycetophilidae|[4220]|GMARK738-14|Diptera|Mycetophilidae||BOLD:ACN0995
Mycetophilidae|[4221]|GMAGI151-15|Diptera|Mycetophilidae||BOLD:ACN1654
Mycetophilidae|[4222]|GMARF264-14|Diptera|Mycetophilidae||BOLD:ACM9316
Mycetophilidae|[4223]|GMARB712-14|Diptera|Mycetophilidae||BOLD:ACM4759
Mycetophilidae|[4224]|GMAGD153-15|Diptera|Mycetophilidae||BOLD:ACW5258
Mycetophilidae|[4225]|GMARK540-14|Diptera|Mycetophilidae||BOLD:ACN5082
Mycetophilidae|[4226]|GMART1004-14|Diptera|Mycetophilidae||BOLD:ACN9751
Mycetophilidae|[4227]|GMAGI488-15|Diptera|Mycetophilidae||BOLD:ACW3545
Mycetophilidae|[4228]|GMAGT139-15|Diptera|Mycetophilidae||BOLD:ACX6186
Mycetophilidae|[4229]|GMAGG1114-15|Diptera|Mycetophilidae||BOLD:ACW4371
Mycetophilidae|[4230]|GMAGC555-15|Diptera|Mycetophilidae||BOLD:ACN0479
Mycetophilidae|[4231]|GMARF982-14|Diptera|Mycetophilidae||BOLD:ACN1517
Mycetophilidae|[4232]|GMAGV342-15|Diptera|Mycetophilidae||BOLD:ACX2903
Mycetophilidae|[4233]|GMARB1410-14|Diptera|Mycetophilidae||BOLD:ACM5051
Mycetophilidae|[4234]|GMARC389-14|Diptera|Mycetophilidae||BOLD:ACM8255
Mycetophilidae|[4235]|GMARB829-14|Diptera|Mycetophilidae||BOLD:ACM4945
Mycetophilidae|[4236]|GMAGI373-15|Diptera|Mycetophilidae||BOLD:ACW4739
Mycetophilidae|[4237]|GMARJ1644-14|Diptera|Mycetophilidae||BOLD:ACM9961
Mycetophilidae|[4238]|GMARJ888-14|Diptera|Mycetophilidae||BOLD:ACN0112
Mycetophilidae|[4239]|GMAGG1097-15|Diptera|Mycetophilidae||BOLD:ACW4081
Mycetophilidae|[4240]|GMAGJ299-15|Diptera|Mycetophilidae||BOLD:ACW8196
Mycetophilidae|[4241]|GMAGA645-15|Diptera|Mycetophilidae||BOLD:ACN0782
Mycetophilidae|[4242]|GMAGA371-15|Diptera|Mycetophilidae||BOLD:ACM8354
Mycetophilidae|[4243]|GMARH944-14|Diptera|Mycetophilidae||BOLD:ACN0246
Mycetophilidae|[4244]|GMARI937-14|Diptera|Mycetophilidae||BOLD:ACN0504
Mycetophilidae|[4245]|GMAGC663-15|Diptera|Mycetophilidae||BOLD:ACW1951
Mycetophilidae|[4246]|GMAGE403-15|Diptera|Mycetophilidae||BOLD:ACW7571
Mycetophilidae|[4247]|GMART301-14|Diptera|Mycetophilidae||BOLD:ACO0018
Mycetophilidae|[4248]|GMAGI129-15|Diptera|Mycetophilidae||BOLD:ACW7930
Mycetophilidae|[4249]|GMARE991-14|Diptera|Mycetophilidae||BOLD:ACN1559
Mycetophilidae|[4250]|GMAGI435-15|Diptera|Mycetophilidae||BOLD:ACW3354
Mycetophilidae|[4251]|GMAGB174-15|Diptera|Mycetophilidae||BOLD:ACM5328
Mycetophilidae|[4252]|GMAGE377-15|Diptera|Mycetophilidae||BOLD:ACN1592
Mycetophilidae|[4253]|GMAGN813-15|Diptera|Mycetophilidae||BOLD:ACX4765
Mycetophilidae|[4254]|GMAGC675-15|Diptera|Mycetophilidae||BOLD:ACN0978
Mycetophilidae|[4255]|GMARI1827-14|Diptera|Mycetophilidae||BOLD:ACN0258
Mycetophilidae|[4256]|GMARG663-14|Diptera|Mycetophilidae||BOLD:ACN1257
Mycetophilidae|[4257]|GMAGP1122-15|Diptera|Mycetophilidae||BOLD:ACX5071
Sceptonia|[4258]|GMARJ228-14|Diptera|Mycetophilidae|Mycetophilinae|BOLD:ACX3686
Mycetophilidae|[4259]|GMAGD354-15|Diptera|Mycetophilidae||BOLD:ACR4802
Mycetophilidae|[4260]|GMAGO1234-15|Diptera|Mycetophilidae||BOLD:ACW7427
Mycetophilidae|[4261]|GMAGE359-15|Diptera|Mycetophilidae||BOLD:ABW3762
Mycetophilidae|[4262]|GMAGN1431-15|Diptera|Mycetophilidae||BOLD:ACX1838
Mycetophilidae|[4263]|GMAGI793-15|Diptera|Mycetophilidae||BOLD:ACN0546
Mycetophilidae|[4264]|GMARJ262-14|Diptera|Mycetophilidae||BOLD:ACN6798
Mycetophilidae|[4265]|GMAGB421-15|Diptera|Mycetophilidae||BOLD:ACM7890
Mycetophilidae|[4266]|GMARH555-14|Diptera|Mycetophilidae||BOLD:ACN1038
Mycetophilidae|[4267]|GMAGM336-15|Diptera|Mycetophilidae||BOLD:ACM9958
Mycetophilidae|[4268]|GMARH633-14|Diptera|Mycetophilidae||BOLD:ACN0588
Mycetophilidae|[4269]|GMARE443-14|Diptera|Mycetophilidae||BOLD:ACM7729
Mycetophilidae|[4270]|GMAGJ163-15|Diptera|Mycetophilidae||BOLD:ACN5183
Mycetophilidae|[4271]|GMAGK259-15|Diptera|Mycetophilidae||BOLD:ACN5184
Mycetophilidae|[4272]|GMAGO1362-15|Diptera|Mycetophilidae||BOLD:ACX4504
Mycetophilidae|[4273]|GMAGI236-15|Diptera|Mycetophilidae||BOLD:ACN1437
Mycetophilidae|[4274]|GMARJ081-14|Diptera|Mycetophilidae||BOLD:ACN1681
Mycetophilidae|[4275]|GMARL1312-14|Diptera|Mycetophilidae||BOLD:ACN2911
Mycetophilidae|[4276]|GMAGI413-15|Diptera|Mycetophilidae||BOLD:ACM9234
Mycetophilidae|[4277]|GMAGE547-15|Diptera|Mycetophilidae||BOLD:ACM8980
Mycetophilidae|[4278]|GMARL470-14|Diptera|Mycetophilidae||BOLD:ACN4816
Mycetophilidae|[4279]|GMAGM153-15|Diptera|Mycetophilidae||BOLD:ACN1447
Mycetophilidae|[4280]|GMAGL005-15|Diptera|Mycetophilidae||BOLD:ACX4879
Mycetophilidae|[4281]|GMARN483-14|Diptera|Mycetophilidae||BOLD:ACN3275

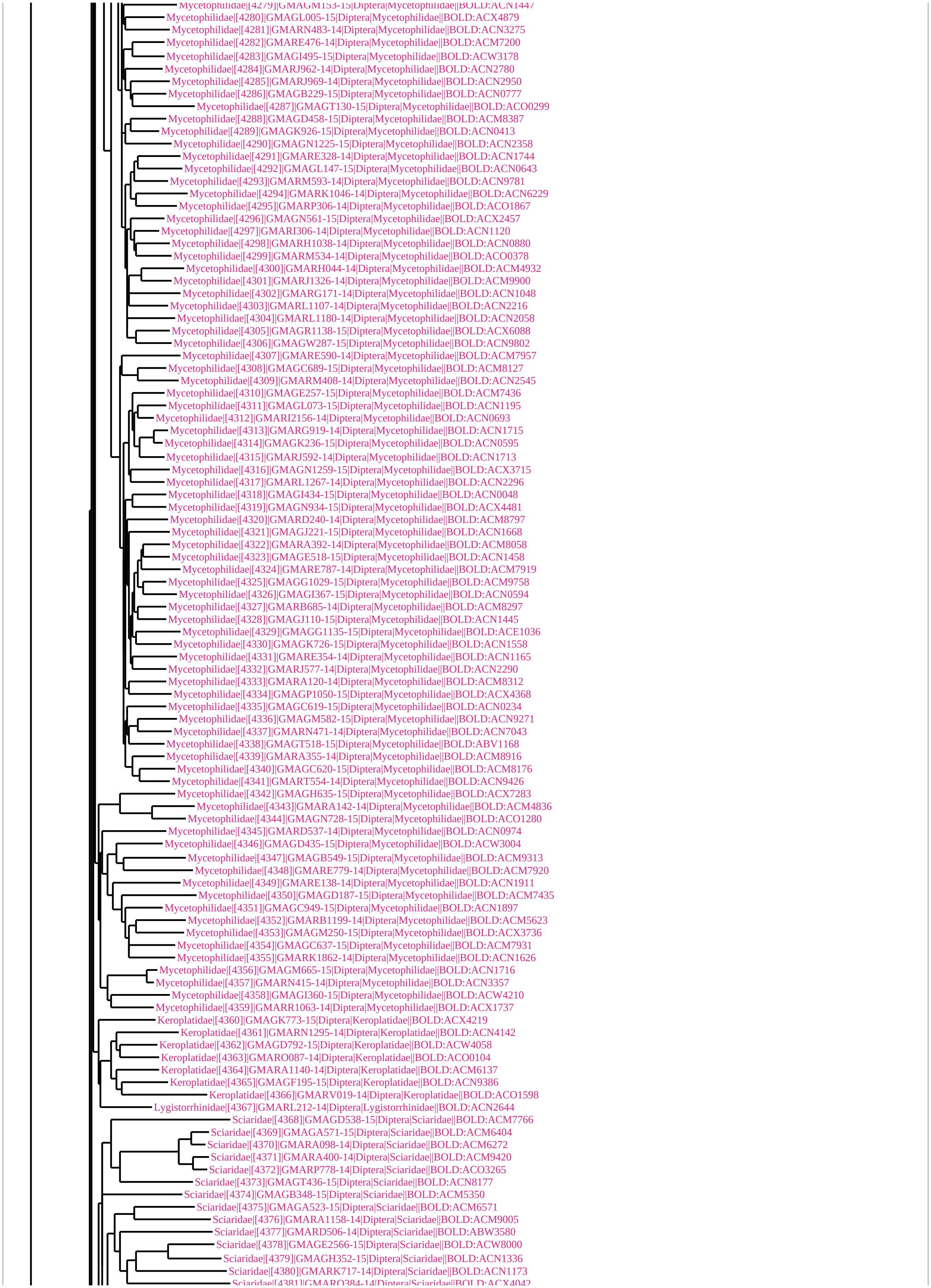
Mycetophilidae|[4279]|GMAGM153-15|Diptera|Mycetophilidae||BOLD:ACN1447
Mycetophilidae|[4280]|GMAGL005-15|Diptera|Mycetophilidae||BOLD:ACX4879
Mycetophilidae|[4281]|GMARN483-14|Diptera|Mycetophilidae||BOLD:ACN3275
Mycetophilidae|[4282]|GMARE476-14|Diptera|Mycetophilidae||BOLD:ACM7200
Mycetophilidae|[4283]|GMAGI495-15|Diptera|Mycetophilidae||BOLD:ACW3178
Mycetophilidae|[4284]|GMARJ962-14|Diptera|Mycetophilidae||BOLD:ACN2780
Mycetophilidae|[4285]|GMARJ969-14|Diptera|Mycetophilidae||BOLD:ACN2950
Mycetophilidae|[4286]|GMAGB229-15|Diptera|Mycetophilidae||BOLD:ACN0777
Mycetophilidae|[4287]|GMAGT130-15|Diptera|Mycetophilidae||BOLD:ACO0299
Mycetophilidae|[4288]|GMAGD458-15|Diptera|Mycetophilidae||BOLD:ACM8387
Mycetophilidae|[4289]|GMAGK926-15|Diptera|Mycetophilidae||BOLD:ACN0413
Mycetophilidae|[4290]|GMAGN1225-15|Diptera|Mycetophilidae||BOLD:ACN2358
Mycetophilidae|[4291]|GMARE328-14|Diptera|Mycetophilidae||BOLD:ACN1744
Mycetophilidae|[4292]|GMAGL147-15|Diptera|Mycetophilidae||BOLD:ACN0643
Mycetophilidae|[4293]|GMARM593-14|Diptera|Mycetophilidae||BOLD:ACN9781
Mycetophilidae|[4294]|GMARK1046-14|Diptera|Mycetophilidae||BOLD:ACN6229
Mycetophilidae|[4295]|GMARP306-14|Diptera|Mycetophilidae||BOLD:ACO1867
Mycetophilidae|[4296]|GMAGN561-15|Diptera|Mycetophilidae||BOLD:ACX2457
Mycetophilidae|[4297]|GMARI306-14|Diptera|Mycetophilidae||BOLD:ACN1120
Mycetophilidae|[4298]|GMARH1038-14|Diptera|Mycetophilidae||BOLD:ACN0880
Mycetophilidae|[4299]|GMARM534-14|Diptera|Mycetophilidae||BOLD:ACO0378
Mycetophilidae|[4300]|GMARH044-14|Diptera|Mycetophilidae||BOLD:ACM4932
Mycetophilidae|[4301]|GMARJ1326-14|Diptera|Mycetophilidae||BOLD:ACM9900
Mycetophilidae|[4302]|GMARG171-14|Diptera|Mycetophilidae||BOLD:ACN1048
Mycetophilidae|[4303]|GMARL1107-14|Diptera|Mycetophilidae||BOLD:ACN2216
Mycetophilidae|[4304]|GMARL1180-14|Diptera|Mycetophilidae||BOLD:ACN2058
Mycetophilidae|[4305]|GMAGR1138-15|Diptera|Mycetophilidae||BOLD:ACX6088
Mycetophilidae|[4306]|GMAGW287-15|Diptera|Mycetophilidae||BOLD:ACN9802
Mycetophilidae|[4307]|GMARE590-14|Diptera|Mycetophilidae||BOLD:ACM7957
Mycetophilidae|[4308]|GMAGC689-15|Diptera|Mycetophilidae||BOLD:ACM8127
Mycetophilidae|[4309]|GMARM408-14|Diptera|Mycetophilidae||BOLD:ACN2545
Mycetophilidae|[4310]|GMAGE257-15|Diptera|Mycetophilidae||BOLD:ACM7436
Mycetophilidae|[4311]|GMAGL073-15|Diptera|Mycetophilidae||BOLD:ACN1195
Mycetophilidae|[4312]|GMARI2156-14|Diptera|Mycetophilidae||BOLD:ACN0693
Mycetophilidae|[4313]|GMARG919-14|Diptera|Mycetophilidae||BOLD:ACN1715
Mycetophilidae|[4314]|GMAGK236-15|Diptera|Mycetophilidae||BOLD:ACN0595
Mycetophilidae|[4315]|GMARJ592-14|Diptera|Mycetophilidae||BOLD:ACN1713
Mycetophilidae|[4316]|GMAGN1259-15|Diptera|Mycetophilidae||BOLD:ACX3715
Mycetophilidae|[4317]|GMARL1267-14|Diptera|Mycetophilidae||BOLD:ACN2296
Mycetophilidae|[4318]|GMAGI434-15|Diptera|Mycetophilidae||BOLD:ACN0048
Mycetophilidae|[4319]|GMAGN934-15|Diptera|Mycetophilidae||BOLD:ACX4481
Mycetophilidae|[4320]|GMARD240-14|Diptera|Mycetophilidae||BOLD:ACM8797
Mycetophilidae|[4321]|GMAGJ221-15|Diptera|Mycetophilidae||BOLD:ACN1668
Mycetophilidae|[4322]|GMARA392-14|Diptera|Mycetophilidae||BOLD:ACM8058
Mycetophilidae|[4323]|GMAGE518-15|Diptera|Mycetophilidae||BOLD:ACN1458
Mycetophilidae|[4324]|GMARE787-14|Diptera|Mycetophilidae||BOLD:ACM7919
Mycetophilidae|[4325]|GMAGG1029-15|Diptera|Mycetophilidae||BOLD:ACM9758
Mycetophilidae|[4326]|GMAGI367-15|Diptera|Mycetophilidae||BOLD:ACN0594
Mycetophilidae|[4327]|GMARB685-14|Diptera|Mycetophilidae||BOLD:ACM8297
Mycetophilidae|[4328]|GMAGJ110-15|Diptera|Mycetophilidae||BOLD:ACN1445
Mycetophilidae|[4329]|GMAGG1135-15|Diptera|Mycetophilidae||BOLD:ACE1036
Mycetophilidae|[4330]|GMAGK726-15|Diptera|Mycetophilidae||BOLD:ACN1558
Mycetophilidae|[4331]|GMARE354-14|Diptera|Mycetophilidae||BOLD:ACN1165
Mycetophilidae|[4332]|GMARJ577-14|Diptera|Mycetophilidae||BOLD:ACN2290
Mycetophilidae|[4333]|GMARA120-14|Diptera|Mycetophilidae||BOLD:ACM8312
Mycetophilidae|[4334]|GMAGP1050-15|Diptera|Mycetophilidae||BOLD:ACX4368
Mycetophilidae|[4335]|GMAGC619-15|Diptera|Mycetophilidae||BOLD:ACN0234
Mycetophilidae|[4336]|GMAGM582-15|Diptera|Mycetophilidae||BOLD:ACN9271
Mycetophilidae|[4337]|GMARN471-14|Diptera|Mycetophilidae||BOLD:ACN7043
Mycetophilidae|[4338]|GMAGT518-15|Diptera|Mycetophilidae||BOLD:ABV1168
Mycetophilidae|[4339]|GMARA355-14|Diptera|Mycetophilidae||BOLD:ACM8916
Mycetophilidae|[4340]|GMAGC620-15|Diptera|Mycetophilidae||BOLD:ACM8176
Mycetophilidae|[4341]|GMART554-14|Diptera|Mycetophilidae||BOLD:ACN9426
Mycetophilidae|[4342]|GMAGH635-15|Diptera|Mycetophilidae||BOLD:ACX7283
Mycetophilidae|[4343]|GMARA142-14|Diptera|Mycetophilidae||BOLD:ACM4836
Mycetophilidae|[4344]|GMAGN728-15|Diptera|Mycetophilidae||BOLD:ACO1280
Mycetophilidae|[4345]|GMARD537-14|Diptera|Mycetophilidae||BOLD:ACN0974
Mycetophilidae|[4346]|GMAGD435-15|Diptera|Mycetophilidae||BOLD:ACW3004
Mycetophilidae|[4347]|GMAGB549-15|Diptera|Mycetophilidae||BOLD:ACM9313
Mycetophilidae|[4348]|GMARE779-14|Diptera|Mycetophilidae||BOLD:ACM7920
Mycetophilidae|[4349]|GMARE138-14|Diptera|Mycetophilidae||BOLD:ACN1911
Mycetophilidae|[4350]|GMAGD187-15|Diptera|Mycetophilidae||BOLD:ACM7435
Mycetophilidae|[4351]|GMAGC949-15|Diptera|Mycetophilidae||BOLD:ACN1897
Mycetophilidae|[4352]|GMARB1199-14|Diptera|Mycetophilidae||BOLD:ACM5623
Mycetophilidae|[4353]|GMAGM250-15|Diptera|Mycetophilidae||BOLD:ACX3736
Mycetophilidae|[4354]|GMAGC637-15|Diptera|Mycetophilidae||BOLD:ACM7931
Mycetophilidae|[4355]|GMARK1862-14|Diptera|Mycetophilidae||BOLD:ACN1626
Mycetophilidae|[4356]|GMAGM665-15|Diptera|Mycetophilidae||BOLD:ACN1716
Mycetophilidae|[4357]|GMARN415-14|Diptera|Mycetophilidae||BOLD:ACN3357
Mycetophilidae|[4358]|GMAGI360-15|Diptera|Mycetophilidae||BOLD:ACW4210
Mycetophilidae|[4359]|GMARR1063-14|Diptera|Mycetophilidae||BOLD:ACX1737
Keroplatidae|[4360]|GMAGK773-15|Diptera|Keroplatidae||BOLD:ACX4219
Keroplatidae|[4361]|GMARN1295-14|Diptera|Keroplatidae||BOLD:ACN4142
Keroplatidae|[4362]|GMAGD792-15|Diptera|Keroplatidae||BOLD:ACW4058
Keroplatidae|[4363]|GMARO087-14|Diptera|Keroplatidae||BOLD:ACO0104
Keroplatidae|[4364]|GMARA1140-14|Diptera|Keroplatidae||BOLD:ACM6137
Keroplatidae|[4365]|GMAGF195-15|Diptera|Keroplatidae||BOLD:ACN9386
Keroplatidae|[4366]|GMARV019-14|Diptera|Keroplatidae||BOLD:ACO1598
Lygistorrhinidae|[4367]|GMARL212-14|Diptera|Lygistorrhinidae||BOLD:ACN2644
Sciaridae|[4368]|GMAGD538-15|Diptera|Sciaridae||BOLD:ACM7766
Sciaridae|[4369]|GMAGA571-15|Diptera|Sciaridae||BOLD:ACM6404
Sciaridae|[4370]|GMARA098-14|Diptera|Sciaridae||BOLD:ACM6272
Sciaridae|[4371]|GMARA400-14|Diptera|Sciaridae||BOLD:ACM9420
Sciaridae|[4372]|GMARP778-14|Diptera|Sciaridae||BOLD:ACO3265
Sciaridae|[4373]|GMAGT436-15|Diptera|Sciaridae||BOLD:ACN8177
Sciaridae|[4374]|GMAGB348-15|Diptera|Sciaridae||BOLD:ACM5350
Sciaridae|[4375]|GMAGA523-15|Diptera|Sciaridae||BOLD:ACM6571
Sciaridae|[4376]|GMARA1158-14|Diptera|Sciaridae||BOLD:ACM9005
Sciaridae|[4377]|GMARD506-14|Diptera|Sciaridae||BOLD:ABW3580
Sciaridae|[4378]|GMAGE2566-15|Diptera|Sciaridae||BOLD:ACW8000
Sciaridae|[4379]|GMAGH352-15|Diptera|Sciaridae||BOLD:ACN1336
Sciaridae|[4380]|GMARK717-14|Diptera|Sciaridae||BOLD:ACN1173
Sciaridae|[4381]|GMARO384-14|Diptera|Sciaridae||BOLD:ACX4042

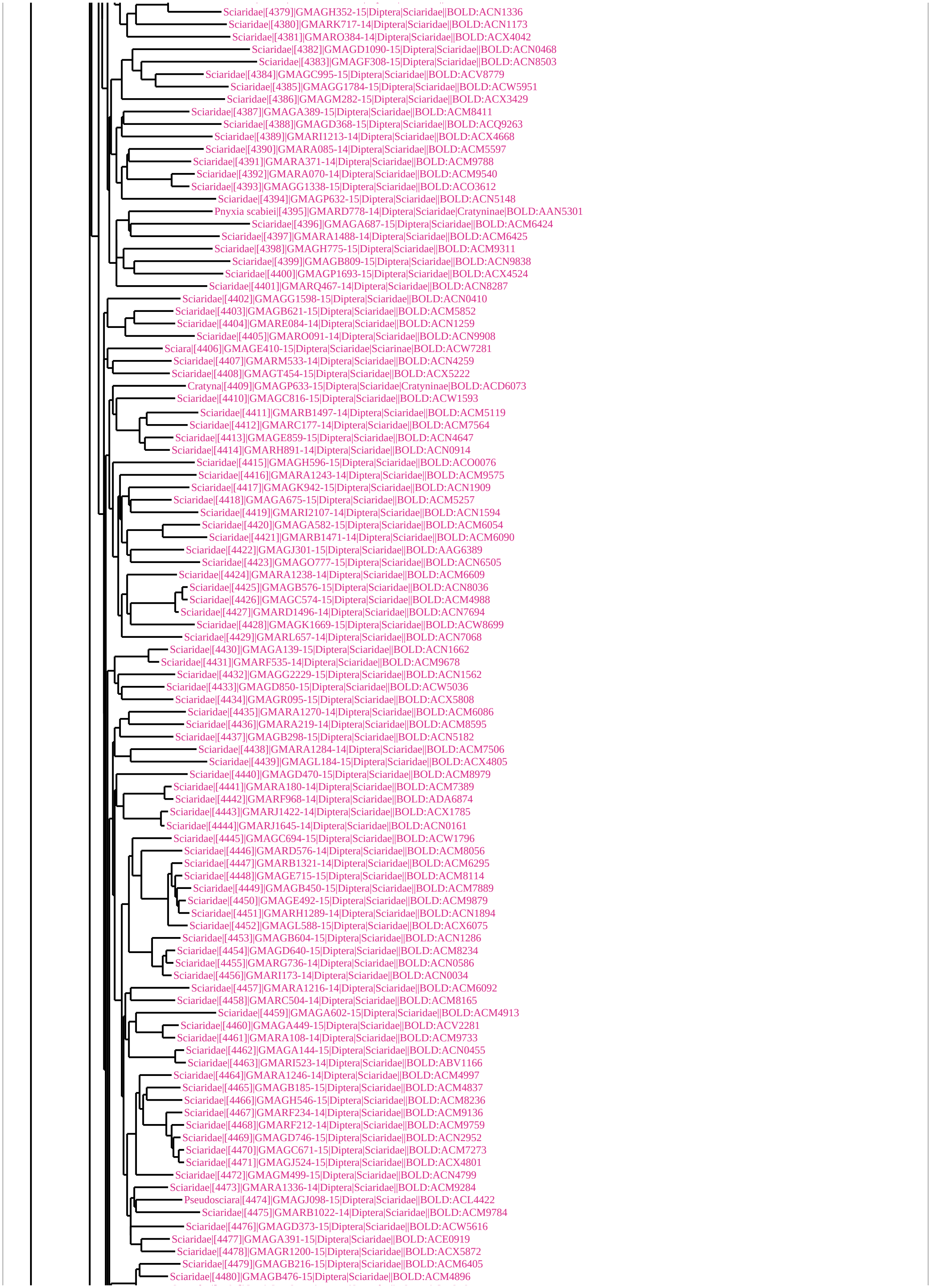
Sciaridae|[4379]|GMAGH352-15|Diptera|Sciaridae||BOLD:ACN1336
Sciaridae|[4380]|GMARK717-14|Diptera|Sciaridae||BOLD:ACN1173
Sciaridae|[4381]|GMARO384-14|Diptera|Sciaridae||BOLD:ACX4042
Sciaridae|[4382]|GMAGD1090-15|Diptera|Sciaridae||BOLD:ACN0468
Sciaridae|[4383]|GMAGF308-15|Diptera|Sciaridae||BOLD:ACN8503
Sciaridae|[4384]|GMAGC995-15|Diptera|Sciaridae||BOLD:ACV8779
Sciaridae|[4385]|GMAGG1784-15|Diptera|Sciaridae||BOLD:ACW5951
Sciaridae|[4386]|GMAGM282-15|Diptera|Sciaridae||BOLD:ACX3429
Sciaridae|[4387]|GMAGA389-15|Diptera|Sciaridae||BOLD:ACM8411
Sciaridae|[4388]|GMAGD368-15|Diptera|Sciaridae||BOLD:ACQ9263
Sciaridae|[4389]|GMARI1213-14|Diptera|Sciaridae||BOLD:ACX4668
Sciaridae|[4390]|GMARA085-14|Diptera|Sciaridae||BOLD:ACM5597
Sciaridae|[4391]|GMARA371-14|Diptera|Sciaridae||BOLD:ACM9788
Sciaridae|[4392]|GMARA070-14|Diptera|Sciaridae||BOLD:ACM9540
Sciaridae|[4393]|GMAGG1338-15|Diptera|Sciaridae||BOLD:ACO3612
Sciaridae|[4394]|GMAGP632-15|Diptera|Sciaridae||BOLD:ACN5148
Pnyxia scabiei|[4395]|GMARD778-14|Diptera|Sciaridae|Cratyninae|BOLD:AAN5301
Sciaridae|[4396]|GMAGA687-15|Diptera|Sciaridae||BOLD:ACM6424
Sciaridae|[4397]|GMARA1488-14|Diptera|Sciaridae||BOLD:ACM6425
Sciaridae|[4398]|GMAGH775-15|Diptera|Sciaridae||BOLD:ACM9311
Sciaridae|[4399]|GMAGB809-15|Diptera|Sciaridae||BOLD:ACN9838
Sciaridae|[4400]|GMAGP1693-15|Diptera|Sciaridae||BOLD:ACX4524
Sciaridae|[4401]|GMARQ467-14|Diptera|Sciaridae||BOLD:ACN8287
Sciaridae|[4402]|GMAGG1598-15|Diptera|Sciaridae||BOLD:ACN0410
Sciaridae|[4403]|GMAGB621-15|Diptera|Sciaridae||BOLD:ACM5852
Sciaridae|[4404]|GMARE084-14|Diptera|Sciaridae||BOLD:ACN1259
Sciaridae|[4405]|GMARO091-14|Diptera|Sciaridae||BOLD:ACN9908
Sciara|[4406]|GMAGE410-15|Diptera|Sciaridae|Sciarinae|BOLD:ACW7281
Sciaridae|[4407]|GMARM533-14|Diptera|Sciaridae||BOLD:ACN4259
Sciaridae|[4408]|GMAGT454-15|Diptera|Sciaridae||BOLD:ACX5222
Cratyna|[4409]|GMAGP633-15|Diptera|Sciaridae|Cratyninae|BOLD:ACD6073
Sciaridae|[4410]|GMAGC816-15|Diptera|Sciaridae||BOLD:ACW1593
Sciaridae|[4411]|GMARB1497-14|Diptera|Sciaridae||BOLD:ACM5119
Sciaridae|[4412]|GMARC177-14|Diptera|Sciaridae||BOLD:ACM7564
Sciaridae|[4413]|GMAGE859-15|Diptera|Sciaridae||BOLD:ACN4647
Sciaridae|[4414]|GMARH891-14|Diptera|Sciaridae||BOLD:ACN0914
Sciaridae|[4415]|GMAGH596-15|Diptera|Sciaridae||BOLD:ACO0076
Sciaridae|[4416]|GMARA1243-14|Diptera|Sciaridae||BOLD:ACM9575
Sciaridae|[4417]|GMAGK942-15|Diptera|Sciaridae||BOLD:ACN1909
Sciaridae|[4418]|GMAGA675-15|Diptera|Sciaridae||BOLD:ACM5257
Sciaridae|[4419]|GMARI2107-14|Diptera|Sciaridae||BOLD:ACN1594
Sciaridae|[4420]|GMAGA582-15|Diptera|Sciaridae||BOLD:ACM6054
Sciaridae|[4421]|GMARB1471-14|Diptera|Sciaridae||BOLD:ACM6090
Sciaridae|[4422]|GMAGJ301-15|Diptera|Sciaridae||BOLD:AAG6389
Sciaridae|[4423]|GMAGO777-15|Diptera|Sciaridae||BOLD:ACN6505
Sciaridae|[4424]|GMARA1238-14|Diptera|Sciaridae||BOLD:ACM6609
Sciaridae|[4425]|GMAGB576-15|Diptera|Sciaridae||BOLD:ACN8036
Sciaridae|[4426]|GMAGC574-15|Diptera|Sciaridae||BOLD:ACM4988
Sciaridae|[4427]|GMARD1496-14|Diptera|Sciaridae||BOLD:ACN7694
Sciaridae|[4428]|GMAGK1669-15|Diptera|Sciaridae||BOLD:ACW8699
Sciaridae|[4429]|GMARL657-14|Diptera|Sciaridae||BOLD:ACN7068
Sciaridae|[4430]|GMAGA139-15|Diptera|Sciaridae||BOLD:ACN1662
Sciaridae|[4431]|GMARF535-14|Diptera|Sciaridae||BOLD:ACM9678
Sciaridae|[4432]|GMAGG2229-15|Diptera|Sciaridae||BOLD:ACN1562
Sciaridae|[4433]|GMAGD850-15|Diptera|Sciaridae||BOLD:ACW5036
Sciaridae|[4434]|GMAGR095-15|Diptera|Sciaridae||BOLD:ACX5808
Sciaridae|[4435]|GMARA1270-14|Diptera|Sciaridae||BOLD:ACM6086
Sciaridae|[4436]|GMARA219-14|Diptera|Sciaridae||BOLD:ACM8595
Sciaridae|[4437]|GMAGB298-15|Diptera|Sciaridae||BOLD:ACN5182
Sciaridae|[4438]|GMARA1284-14|Diptera|Sciaridae||BOLD:ACM7506
Sciaridae|[4439]|GMAGL184-15|Diptera|Sciaridae||BOLD:ACX4805
Sciaridae|[4440]|GMAGD470-15|Diptera|Sciaridae||BOLD:ACM8979
Sciaridae|[4441]|GMARA180-14|Diptera|Sciaridae||BOLD:ACM7389
Sciaridae|[4442]|GMARF968-14|Diptera|Sciaridae||BOLD:ADA6874
Sciaridae|[4443]|GMARJ1422-14|Diptera|Sciaridae||BOLD:ACX1785
Sciaridae|[4444]|GMARJ1645-14|Diptera|Sciaridae||BOLD:ACN0161
Sciaridae|[4445]|GMAGC694-15|Diptera|Sciaridae||BOLD:ACW1796
Sciaridae|[4446]|GMARD576-14|Diptera|Sciaridae||BOLD:ACM8056
Sciaridae|[4447]|GMARB1321-14|Diptera|Sciaridae||BOLD:ACM6295
Sciaridae|[4448]|GMAGE715-15|Diptera|Sciaridae||BOLD:ACM8114
Sciaridae|[4449]|GMAGB450-15|Diptera|Sciaridae||BOLD:ACM7889
Sciaridae|[4450]|GMAGE492-15|Diptera|Sciaridae||BOLD:ACM9879
Sciaridae|[4451]|GMARH1289-14|Diptera|Sciaridae||BOLD:ACN1894
Sciaridae|[4452]|GMAGL588-15|Diptera|Sciaridae||BOLD:ACX6075
Sciaridae|[4453]|GMAGB604-15|Diptera|Sciaridae||BOLD:ACN1286
Sciaridae|[4454]|GMAGD640-15|Diptera|Sciaridae||BOLD:ACM8234
Sciaridae|[4455]|GMARG736-14|Diptera|Sciaridae||BOLD:ACN0586
Sciaridae|[4456]|GMARI173-14|Diptera|Sciaridae||BOLD:ACN0034
Sciaridae|[4457]|GMARA1216-14|Diptera|Sciaridae||BOLD:ACM6092
Sciaridae|[4458]|GMARC504-14|Diptera|Sciaridae||BOLD:ACM8165
Sciaridae|[4459]|GMAGA602-15|Diptera|Sciaridae||BOLD:ACM4913
Sciaridae|[4460]|GMAGA449-15|Diptera|Sciaridae||BOLD:ACV2281
Sciaridae|[4461]|GMARA108-14|Diptera|Sciaridae||BOLD:ACM9733
Sciaridae|[4462]|GMAGA144-15|Diptera|Sciaridae||BOLD:ACN0455
Sciaridae|[4463]|GMARI523-14|Diptera|Sciaridae||BOLD:ABV1166
Sciaridae|[4464]|GMARA1246-14|Diptera|Sciaridae||BOLD:ACM4997
Sciaridae|[4465]|GMAGB185-15|Diptera|Sciaridae||BOLD:ACM4837
Sciaridae|[4466]|GMAGH546-15|Diptera|Sciaridae||BOLD:ACM8236
Sciaridae|[4467]|GMARF234-14|Diptera|Sciaridae||BOLD:ACM9136
Sciaridae|[4468]|GMARF212-14|Diptera|Sciaridae||BOLD:ACM9759
Sciaridae|[4469]|GMAGD746-15|Diptera|Sciaridae||BOLD:ACN2952
Sciaridae|[4470]|GMAGC671-15|Diptera|Sciaridae||BOLD:ACM7273
Sciaridae|[4471]|GMAGJ524-15|Diptera|Sciaridae||BOLD:ACX4801
Sciaridae|[4472]|GMAGM499-15|Diptera|Sciaridae||BOLD:ACN4799
Sciaridae|[4473]|GMARA1336-14|Diptera|Sciaridae||BOLD:ACM9284
Pseudosciara|[4474]|GMAGJ098-15|Diptera|Sciaridae||BOLD:ACL4422
Sciaridae|[4475]|GMARB1022-14|Diptera|Sciaridae||BOLD:ACM9784
Sciaridae|[4476]|GMAGD373-15|Diptera|Sciaridae||BOLD:ACW5616
Sciaridae|[4477]|GMAGA391-15|Diptera|Sciaridae||BOLD:ACE0919
Sciaridae|[4478]|GMAGR1200-15|Diptera|Sciaridae||BOLD:ACX5872
Sciaridae|[4479]|GMAGB216-15|Diptera|Sciaridae||BOLD:ACM6405
Sciaridae|[4480]|GMAGB476-15|Diptera|Sciaridae||BOLD:ACM4896

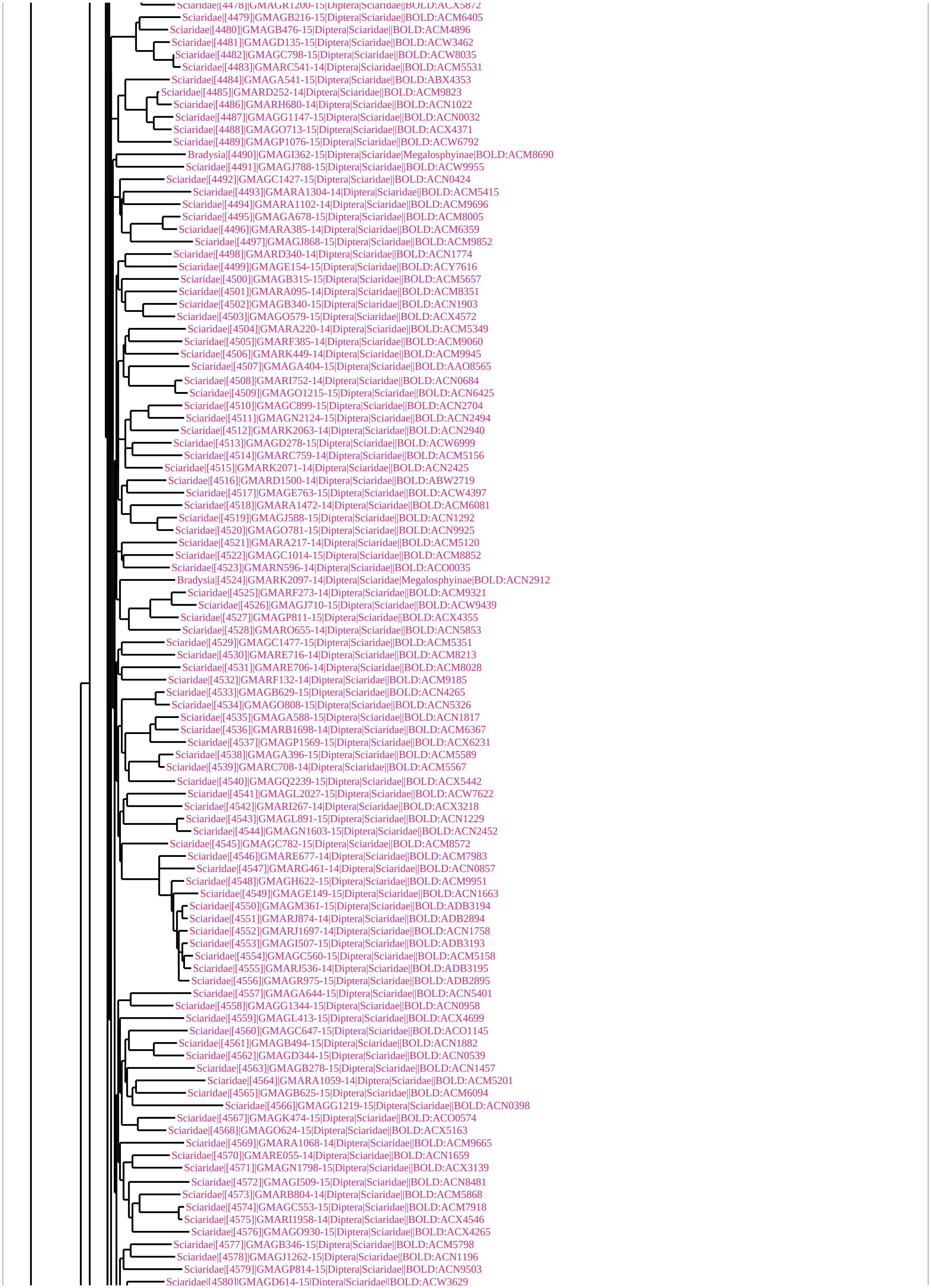
Sciaridae|[4478]|GMAGR1200-15|Diptera|Sciaridae||BOLD:ACX5872
Sciaridae|[4479]|GMAGB216-15|Diptera|Sciaridae||BOLD:ACM6405
Sciaridae|[4480]|GMAGB476-15|Diptera|Sciaridae||BOLD:ACM4896
Sciaridae|[4481]|GMAGD135-15|Diptera|Sciaridae||BOLD:ACW3462
Sciaridae|[4482]|GMAGC798-15|Diptera|Sciaridae||BOLD:ACW8035
Sciaridae|[4483]|GMARC541-14|Diptera|Sciaridae||BOLD:ACM5531
Sciaridae|[4484]|GMAGA541-15|Diptera|Sciaridae||BOLD:ABX4353
Sciaridae|[4485]|GMARD252-14|Diptera|Sciaridae||BOLD:ACM9823
Sciaridae|[4486]|GMARH680-14|Diptera|Sciaridae||BOLD:ACN1022
Sciaridae|[4487]|GMAGG1147-15|Diptera|Sciaridae||BOLD:ACN0032
Sciaridae|[4488]|GMAGO713-15|Diptera|Sciaridae||BOLD:ACX4371
Sciaridae|[4489]|GMAGP1076-15|Diptera|Sciaridae||BOLD:ACW6792
Bradysia|[4490]|GMAGI362-15|Diptera|Sciaridae|Megalosphyinae|BOLD:ACM8690
Sciaridae|[4491]|GMAGJ788-15|Diptera|Sciaridae||BOLD:ACW9955
Sciaridae|[4492]|GMAGC1427-15|Diptera|Sciaridae||BOLD:ACN0424
Sciaridae|[4493]|GMARA1304-14|Diptera|Sciaridae||BOLD:ACM5415
Sciaridae|[4494]|GMARA1102-14|Diptera|Sciaridae||BOLD:ACM9696
Sciaridae|[4495]|GMAGA678-15|Diptera|Sciaridae||BOLD:ACM8005
Sciaridae|[4496]|GMARA385-14|Diptera|Sciaridae||BOLD:ACM6359
Sciaridae|[4497]|GMAGJ868-15|Diptera|Sciaridae||BOLD:ACM9852
Sciaridae|[4498]|GMARD340-14|Diptera|Sciaridae||BOLD:ACN1774
Sciaridae|[4499]|GMAGE154-15|Diptera|Sciaridae||BOLD:ACY7616
Sciaridae|[4500]|GMAGB315-15|Diptera|Sciaridae||BOLD:ACM5657
Sciaridae|[4501]|GMARA095-14|Diptera|Sciaridae||BOLD:ACM8351
Sciaridae|[4502]|GMAGB340-15|Diptera|Sciaridae||BOLD:ACN1903
Sciaridae|[4503]|GMAGO579-15|Diptera|Sciaridae||BOLD:ACX4572
Sciaridae|[4504]|GMARA220-14|Diptera|Sciaridae||BOLD:ACM5349
Sciaridae|[4505]|GMARF385-14|Diptera|Sciaridae||BOLD:ACM9060
Sciaridae|[4506]|GMARK449-14|Diptera|Sciaridae||BOLD:ACM9945
Sciaridae|[4507]|GMAGA404-15|Diptera|Sciaridae||BOLD:AAO8565
Sciaridae|[4508]|GMARI752-14|Diptera|Sciaridae||BOLD:ACN0684
Sciaridae|[4509]|GMAGO1215-15|Diptera|Sciaridae||BOLD:ACN6425
Sciaridae|[4510]|GMAGC899-15|Diptera|Sciaridae||BOLD:ACN2704
Sciaridae|[4511]|GMAGN2124-15|Diptera|Sciaridae||BOLD:ACN2494
Sciaridae|[4512]|GMARK2063-14|Diptera|Sciaridae||BOLD:ACN2940
Sciaridae|[4513]|GMAGD278-15|Diptera|Sciaridae||BOLD:ACW6999
Sciaridae|[4514]|GMARC759-14|Diptera|Sciaridae||BOLD:ACM5156
Sciaridae|[4515]|GMARK2071-14|Diptera|Sciaridae||BOLD:ACN2425
Sciaridae|[4516]|GMARD1500-14|Diptera|Sciaridae||BOLD:ABW2719
Sciaridae|[4517]|GMAGE763-15|Diptera|Sciaridae||BOLD:ACW4397
Sciaridae|[4518]|GMARA1472-14|Diptera|Sciaridae||BOLD:ACM6081
Sciaridae|[4519]|GMAGJ588-15|Diptera|Sciaridae||BOLD:ACN1292
Sciaridae|[4520]|GMAGO781-15|Diptera|Sciaridae||BOLD:ACN9925
Sciaridae|[4521]|GMARA217-14|Diptera|Sciaridae||BOLD:ACM5120
Sciaridae|[4522]|GMAGC1014-15|Diptera|Sciaridae||BOLD:ACM8852
Sciaridae|[4523]|GMARN596-14|Diptera|Sciaridae||BOLD:ACO0035
Bradysia|[4524]|GMARK2097-14|Diptera|Sciaridae|Megalosphyinae|BOLD:ACN2912
Sciaridae|[4525]|GMARF273-14|Diptera|Sciaridae||BOLD:ACM9321
Sciaridae|[4526]|GMAGJ710-15|Diptera|Sciaridae||BOLD:ACW9439
Sciaridae|[4527]|GMAGP811-15|Diptera|Sciaridae||BOLD:ACX4355
Sciaridae|[4528]|GMARO655-14|Diptera|Sciaridae||BOLD:ACN5853
Sciaridae|[4529]|GMAGC1477-15|Diptera|Sciaridae||BOLD:ACM5351
Sciaridae|[4530]|GMARE716-14|Diptera|Sciaridae||BOLD:ACM8213
Sciaridae|[4531]|GMARE706-14|Diptera|Sciaridae||BOLD:ACM8028
Sciaridae|[4532]|GMARF132-14|Diptera|Sciaridae||BOLD:ACM9185
Sciaridae|[4533]|GMAGB629-15|Diptera|Sciaridae||BOLD:ACN4265
Sciaridae|[4534]|GMAGO808-15|Diptera|Sciaridae||BOLD:ACN5326
Sciaridae|[4535]|GMAGA588-15|Diptera|Sciaridae||BOLD:ACN1817
Sciaridae|[4536]|GMARB1698-14|Diptera|Sciaridae||BOLD:ACM6367
Sciaridae|[4537]|GMAGP1569-15|Diptera|Sciaridae||BOLD:ACX6231
Sciaridae|[4538]|GMAGA396-15|Diptera|Sciaridae||BOLD:ACM5589
Sciaridae|[4539]|GMARC708-14|Diptera|Sciaridae||BOLD:ACM5567
Sciaridae|[4540]|GMAGQ2239-15|Diptera|Sciaridae||BOLD:ACX5442
Sciaridae|[4541]|GMAGL2027-15|Diptera|Sciaridae||BOLD:ACW7622
Sciaridae|[4542]|GMARI267-14|Diptera|Sciaridae||BOLD:ACX3218
Sciaridae|[4543]|GMAGL891-15|Diptera|Sciaridae||BOLD:ACN1229
Sciaridae|[4544]|GMAGN1603-15|Diptera|Sciaridae||BOLD:ACN2452
Sciaridae|[4545]|GMAGC782-15|Diptera|Sciaridae||BOLD:ACM8572
Sciaridae|[4546]|GMARE677-14|Diptera|Sciaridae||BOLD:ACM7983
Sciaridae|[4547]|GMARG461-14|Diptera|Sciaridae||BOLD:ACN0857
Sciaridae|[4548]|GMAGH622-15|Diptera|Sciaridae||BOLD:ACM9951
Sciaridae|[4549]|GMAGE149-15|Diptera|Sciaridae||BOLD:ACN1663
Sciaridae|[4550]|GMAGM361-15|Diptera|Sciaridae||BOLD:ADB3194
Sciaridae|[4551]|GMARJ874-14|Diptera|Sciaridae||BOLD:ADB2894
Sciaridae|[4552]|GMARJ1697-14|Diptera|Sciaridae||BOLD:ACN1758
Sciaridae|[4553]|GMAGI507-15|Diptera|Sciaridae||BOLD:ADB3193
Sciaridae|[4554]|GMAGC560-15|Diptera|Sciaridae||BOLD:ACM5158
Sciaridae|[4555]|GMARJ536-14|Diptera|Sciaridae||BOLD:ADB3195
Sciaridae|[4556]|GMAGR975-15|Diptera|Sciaridae||BOLD:ADB2895
Sciaridae|[4557]|GMAGA644-15|Diptera|Sciaridae||BOLD:ACN5401
Sciaridae|[4558]|GMAGG1344-15|Diptera|Sciaridae||BOLD:ACN0958
Sciaridae|[4559]|GMAGL413-15|Diptera|Sciaridae||BOLD:ACX4699
Sciaridae|[4560]|GMAGC647-15|Diptera|Sciaridae||BOLD:ACO1145
Sciaridae|[4561]|GMAGB494-15|Diptera|Sciaridae||BOLD:ACN1882
Sciaridae|[4562]|GMAGD344-15|Diptera|Sciaridae||BOLD:ACN0539
Sciaridae|[4563]|GMAGB278-15|Diptera|Sciaridae||BOLD:ACN1457
Sciaridae|[4564]|GMARA1059-14|Diptera|Sciaridae||BOLD:ACM5201
Sciaridae|[4565]|GMAGB625-15|Diptera|Sciaridae||BOLD:ACM6094
Sciaridae|[4566]|GMAGG1219-15|Diptera|Sciaridae||BOLD:ACN0398
Sciaridae|[4567]|GMAGK474-15|Diptera|Sciaridae||BOLD:ACO0574
Sciaridae|[4568]|GMAGO624-15|Diptera|Sciaridae||BOLD:ACX5163
Sciaridae|[4569]|GMARA1068-14|Diptera|Sciaridae||BOLD:ACM9665
Sciaridae|[4570]|GMARE055-14|Diptera|Sciaridae||BOLD:ACN1659
Sciaridae|[4571]|GMAGN1798-15|Diptera|Sciaridae||BOLD:ACX3139
Sciaridae|[4572]|GMAGI509-15|Diptera|Sciaridae||BOLD:ACN8481
Sciaridae|[4573]|GMARB804-14|Diptera|Sciaridae||BOLD:ACM5868
Sciaridae|[4574]|GMAGC553-15|Diptera|Sciaridae||BOLD:ACM7918
Sciaridae|[4575]|GMARI1958-14|Diptera|Sciaridae||BOLD:ACX4546
Sciaridae|[4576]|GMAGO930-15|Diptera|Sciaridae||BOLD:ACX4265
Sciaridae|[4577]|GMAGB346-15|Diptera|Sciaridae||BOLD:ACM5798
Sciaridae|[4578]|GMAGJ1262-15|Diptera|Sciaridae||BOLD:ACN1196
Sciaridae|[4579]|GMAGP814-15|Diptera|Sciaridae||BOLD:ACN9503
Sciaridae|[4580]|GMAGD614-15|Diptera|Sciaridae||BOLD:ACW3629

Sciaridae|[4578]|GMAGJ1262-15|Diptera|Sciaridae||BOLD:ACN1196
Sciaridae|[4579]|GMAGP814-15|Diptera|Sciaridae||BOLD:ACN9503
Sciaridae|[4580]|GMAGD614-15|Diptera|Sciaridae||BOLD:ACW3629
Sciaridae|[4581]|GMARK1850-14|Diptera|Sciaridae||BOLD:ACN1764
Sciaridae|[4582]|GMAGA531-15|Diptera|Sciaridae||BOLD:ACM4827
Sciaridae|[4583]|GMARK1779-14|Diptera|Sciaridae||BOLD:ACN0351
Sciaridae|[4584]|GMARA1489-14|Diptera|Sciaridae||BOLD:ACM6077
Sciaridae|[4585]|GMAGO397-15|Diptera|Sciaridae||BOLD:ACX4682
Sciaridae|[4586]|GMAGE805-15|Diptera|Sciaridae||BOLD:ACN0816
Sciaridae|[4587]|GMAGD736-15|Diptera|Sciaridae||BOLD:ACN1523
Sciaridae|[4588]|GMARC368-14|Diptera|Sciaridae||BOLD:ACM8069
Sciaridae|[4589]|GMARM268-14|Diptera|Sciaridae||BOLD:ACN2843
Sciaridae|[4590]|GMARG1212-14|Diptera|Sciaridae||BOLD:ACN2567
Sciaridae|[4591]|GMAGK1780-15|Diptera|Sciaridae||BOLD:ACN2798
Sciaridae|[4592]|GMAGL727-15|Diptera|Sciaridae||BOLD:ACN8513
Sciaridae|[4593]|GMAGK1671-15|Diptera|Sciaridae||BOLD:ACW9307
Sciaridae|[4594]|GMAGL2294-15|Diptera|Sciaridae||BOLD:ACN4497
Sciaridae|[4595]|GMAGH619-15|Diptera|Sciaridae||BOLD:ACN0385
Sciaridae|[4596]|GMARD1408-14|Diptera|Sciaridae||BOLD:ACN1896
Sciaridae|[4597]|GMARF1021-14|Diptera|Sciaridae||BOLD:ACO0126
Sciaridae|[4598]|GMARI1773-14|Diptera|Sciaridae||BOLD:ACN0505
Sciaridae|[4599]|GMAGL1912-15|Diptera|Sciaridae||BOLD:ACN7951
Sciaridae|[4600]|GMAGQ1447-15|Diptera|Sciaridae||BOLD:ACX4096
Sciaridae|[4601]|GMAGK1525-15|Diptera|Sciaridae||BOLD:ACN1384
Sciaridae|[4602]|GMARI1171-14|Diptera|Sciaridae||BOLD:ACN0818
Sciaridae|[4603]|GMARK2163-14|Diptera|Sciaridae||BOLD:ACN2069
Sciaridae|[4604]|GMARG594-14|Diptera|Sciaridae||BOLD:ACM9872
Sciaridae|[4605]|GMARI1154-14|Diptera|Sciaridae||BOLD:ACN0912
Sciaridae|[4606]|GMAGA597-15|Diptera|Sciaridae||BOLD:ACV2874
Sciaridae|[4607]|GMARE228-14|Diptera|Sciaridae||BOLD:ACN1401
Sciaridae|[4608]|GMARC714-14|Diptera|Sciaridae||BOLD:ACM4838
Sciaridae|[4609]|GMAGJ1154-15|Diptera|Sciaridae||BOLD:ACN3208
Sciaridae|[4610]|GMARP349-14|Diptera|Sciaridae||BOLD:ACO1414
Sciaridae|[4611]|GMARP646-14|Diptera|Sciaridae||BOLD:ACN9474
Sciaridae|[4612]|GMAGB787-15|Diptera|Sciaridae||BOLD:ACM9210
Sciaridae|[4613]|GMAGE664-15|Diptera|Sciaridae||BOLD:ACN2531
Sciaridae|[4614]|GMAGL2017-15|Diptera|Sciaridae||BOLD:ACO0008
Sciaridae|[4615]|GMAGU303-15|Diptera|Sciaridae||BOLD:ACX2188
Sciaridae|[4616]|GMAGG1746-15|Diptera|Sciaridae||BOLD:ACN0820
Sciaridae|[4617]|GMARI059-14|Diptera|Sciaridae||BOLD:ACN5170
Sciaridae|[4618]|GMAGJ1183-15|Diptera|Sciaridae||BOLD:ACN7006
Sciaridae|[4619]|GMAGA579-15|Diptera|Sciaridae||BOLD:ACM6616
Sciaridae|[4620]|GMAGC052-15|Diptera|Sciaridae||BOLD:ACN0868
Sciaridae|[4621]|GMARR438-14|Diptera|Sciaridae||BOLD:ACN8301
Sciaridae|[4622]|GMARD1153-14|Diptera|Sciaridae||BOLD:ACM9041
Sciaridae|[4623]|GMARK1728-14|Diptera|Sciaridae||BOLD:ACN0680
Sciaridae|[4624]|GMARL957-14|Diptera|Sciaridae||BOLD:ACN2466
Sciaridae|[4625]|GMAGG1651-15|Diptera|Sciaridae||BOLD:ACM7443
Sciaridae|[4626]|GMAGS111-15|Diptera|Sciaridae||BOLD:ACX5583
Sciaridae|[4627]|GMAGN692-15|Diptera|Sciaridae||BOLD:ACN7067
Sciaridae|[4628]|GMAGG1030-15|Diptera|Sciaridae||BOLD:ACW4577
Sciaridae|[4629]|GMAGA546-15|Diptera|Sciaridae||BOLD:ACM5618
Sciaridae|[4630]|GMAGH474-15|Diptera|Sciaridae||BOLD:ACN1037
Sciaridae|[4631]|GMAGJ853-15|Diptera|Sciaridae||BOLD:ACN0405
Sciaridae|[4632]|GMAGK393-15|Diptera|Sciaridae||BOLD:ACN0506
Sciaridae|[4633]|GMARA147-14|Diptera|Sciaridae||BOLD:ACM8896
Sciaridae|[4634]|GMAGA616-15|Diptera|Sciaridae||BOLD:ACM5884
Sciaridae|[4635]|GMARL1280-14|Diptera|Sciaridae||BOLD:ACN2336
Sciaridae|[4636]|GMAGC561-15|Diptera|Sciaridae||BOLD:ACM7441
Sciaridae|[4637]|GMARB1432-14|Diptera|Sciaridae||BOLD:ACM5409
Sciaridae|[4638]|GMAGQ2103-15|Diptera|Sciaridae||BOLD:ACX5821
Sciaridae|[4639]|GMAGN896-15|Diptera|Sciaridae||BOLD:ACN3874
Sciaridae|[4640]|GMAGW279-15|Diptera|Sciaridae||BOLD:ACX3187
Sciaridae|[4641]|GMARI615-14|Diptera|Sciaridae||BOLD:ACN0356
Sciaridae|[4642]|GMAGC644-15|Diptera|Sciaridae||BOLD:ACW1855
Sciaridae|[4643]|GMAGD477-15|Diptera|Sciaridae||BOLD:ACN0198
Sciaridae|[4644]|GMARB854-14|Diptera|Sciaridae||BOLD:ACM5360
Sciaridae|[4645]|GMARD1294-14|Diptera|Sciaridae||BOLD:ACM7847
Sciaridae|[4646]|GMAGM646-15|Diptera|Sciaridae||BOLD:ACX3903
Sciaridae|[4647]|GMARA1299-14|Diptera|Sciaridae||BOLD:ACM4760
Sciaridae|[4648]|GMAGA358-15|Diptera|Sciaridae||BOLD:ACM5857
Sciaridae|[4649]|GMAGB270-15|Diptera|Sciaridae||BOLD:ACM6089
Sciaridae|[4650]|GMAGB179-15|Diptera|Sciaridae||BOLD:ACM5760
Sciaridae|[4651]|GMAGE2918-15|Diptera|Sciaridae||BOLD:ACW7583
Sciaridae|[4652]|GMAGA661-15|Diptera|Sciaridae||BOLD:ACM5069
Sciaridae|[4653]|GMAGH262-15|Diptera|Sciaridae||BOLD:ACW1486
Sciaridae|[4654]|GMAGD689-15|Diptera|Sciaridae||BOLD:ACX3334
Sciaridae|[4655]|GMARG1303-14|Diptera|Sciaridae||BOLD:ACM9860
Sciaridae|[4656]|GMAGA693-15|Diptera|Sciaridae||BOLD:ACM5562
Sciaridae|[4657]|GMAGG2231-15|Diptera|Sciaridae||BOLD:ACN1643
Sciaridae|[4658]|GMAGN1421-15|Diptera|Sciaridae||BOLD:ACX1577
Sciaridae|[4659]|GMARA121-14|Diptera|Sciaridae||BOLD:ACM6087
Sciaridae|[4660]|GMARB941-14|Diptera|Sciaridae||BOLD:ACK6389
Sciaridae|[4661]|GMARB1515-14|Diptera|Sciaridae||BOLD:ACM5235
Sciaridae|[4662]|GMARD578-14|Diptera|Sciaridae||BOLD:ACX7483
Sciaridae|[4663]|GMAGH383-15|Diptera|Sciaridae||BOLD:ACW1370
Sciaridae|[4664]|GMAGA355-15|Diptera|Sciaridae||BOLD:ACM4825
Sciaridae|[4665]|GMARB855-14|Diptera|Sciaridae||BOLD:ACM5361
Sciaridae|[4666]|GMARD1443-14|Diptera|Sciaridae||BOLD:ACM8514
Sciaridae|[4667]|GMAGE708-15|Diptera|Sciaridae||BOLD:ACW4668
Sciaridae|[4668]|GMAGL317-15|Diptera|Sciaridae||BOLD:ACX7497
Sciaridae|[4669]|GMAGA647-15|Diptera|Sciaridae||BOLD:ACN3464
Sciaridae|[4670]|GMARF141-14|Diptera|Sciaridae||BOLD:ACM9139
Sciaridae|[4671]|GMARA133-14|Diptera|Sciaridae||BOLD:ACW3099
Sciaridae|[4672]|GMAGD233-15|Diptera|Sciaridae||BOLD:ACX0906
Sciaridae|[4673]|GMAGE262-15|Diptera|Sciaridae||BOLD:ACM4999
Sciaridae|[4674]|GMARG1600-14|Diptera|Sciaridae||BOLD:ACN1667
Sciaridae|[4675]|GMAGH562-15|Diptera|Sciaridae||BOLD:ACO0091
Sciaridae|[4676]|GMAGN633-15|Diptera|Sciaridae||BOLD:ACN9647
Bradysia peraffinis|[4677]|GMARI720-14|Diptera|Sciaridae|Megalosphyinae|BOLD:ACD7618
Sciaridae|[4678]|GMAGC727-15|Diptera|Sciaridae||BOLD:ACN1879
Sciaridae|[4679]|GMAGC700-15|Diptera|Sciaridae||BOLD:ACN0451
[illegible]

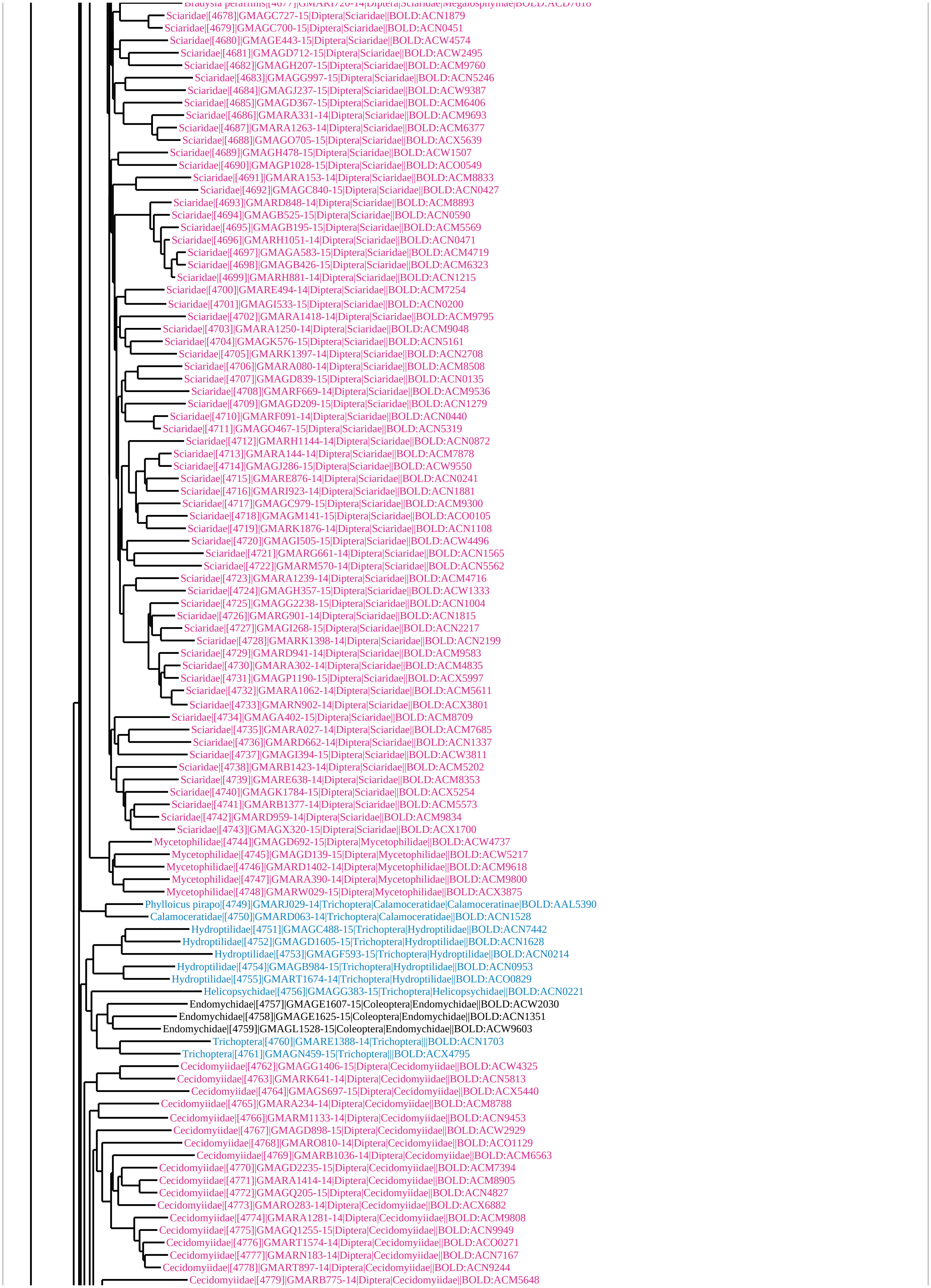
Bradysia peraffinis|[4677]|GMARI720-14|Diptera|Sciaridae|Megalosphyinae|BOLD:ACD7618
Sciaridae|[4678]|GMAGC727-15|Diptera|Sciaridae||BOLD:ACN1879
Sciaridae|[4679]|GMAGC700-15|Diptera|Sciaridae||BOLD:ACN0451
Sciaridae|[4680]|GMAGE443-15|Diptera|Sciaridae||BOLD:ACW4574
Sciaridae|[4681]|GMAGD712-15|Diptera|Sciaridae||BOLD:ACW2495
Sciaridae|[4682]|GMAGH207-15|Diptera|Sciaridae||BOLD:ACM9760
Sciaridae|[4683]|GMAGG997-15|Diptera|Sciaridae||BOLD:ACN5246
Sciaridae|[4684]|GMAGJ237-15|Diptera|Sciaridae||BOLD:ACW9387
Sciaridae|[4685]|GMAGD367-15|Diptera|Sciaridae||BOLD:ACM6406
Sciaridae|[4686]|GMARA331-14|Diptera|Sciaridae||BOLD:ACM9693
Sciaridae|[4687]|GMARA1263-14|Diptera|Sciaridae||BOLD:ACM6377
Sciaridae|[4688]|GMAGO705-15|Diptera|Sciaridae||BOLD:ACX5639
Sciaridae|[4689]|GMAGH478-15|Diptera|Sciaridae||BOLD:ACW1507
Sciaridae|[4690]|GMAGP1028-15|Diptera|Sciaridae||BOLD:ACO0549
Sciaridae|[4691]|GMARA153-14|Diptera|Sciaridae||BOLD:ACM8833
Sciaridae|[4692]|GMAGC840-15|Diptera|Sciaridae||BOLD:ACN0427
Sciaridae|[4693]|GMARD848-14|Diptera|Sciaridae||BOLD:ACM8893
Sciaridae|[4694]|GMAGB525-15|Diptera|Sciaridae||BOLD:ACN0590
Sciaridae|[4695]|GMAGB195-15|Diptera|Sciaridae||BOLD:ACM5569
Sciaridae|[4696]|GMARH1051-14|Diptera|Sciaridae||BOLD:ACN0471
Sciaridae|[4697]|GMAGA583-15|Diptera|Sciaridae||BOLD:ACM4719
Sciaridae|[4698]|GMAGB426-15|Diptera|Sciaridae||BOLD:ACM6323
Sciaridae|[4699]|GMARH881-14|Diptera|Sciaridae||BOLD:ACN1215
Sciaridae|[4700]|GMARE494-14|Diptera|Sciaridae||BOLD:ACM7254
Sciaridae|[4701]|GMAGI533-15|Diptera|Sciaridae||BOLD:ACN0200
Sciaridae|[4702]|GMARA1418-14|Diptera|Sciaridae||BOLD:ACM9795
Sciaridae|[4703]|GMARA1250-14|Diptera|Sciaridae||BOLD:ACM9048
Sciaridae|[4704]|GMAGK576-15|Diptera|Sciaridae||BOLD:ACN5161
Sciaridae|[4705]|GMARK1397-14|Diptera|Sciaridae||BOLD:ACN2708
Sciaridae|[4706]|GMARA080-14|Diptera|Sciaridae||BOLD:ACM8508
Sciaridae|[4707]|GMAGD839-15|Diptera|Sciaridae||BOLD:ACN0135
Sciaridae|[4708]|GMARF669-14|Diptera|Sciaridae||BOLD:ACM9536
Sciaridae|[4709]|GMAGD209-15|Diptera|Sciaridae||BOLD:ACN1279
Sciaridae|[4710]|GMARF091-14|Diptera|Sciaridae||BOLD:ACN0440
Sciaridae|[4711]|GMAGO467-15|Diptera|Sciaridae||BOLD:ACN5319
Sciaridae|[4712]|GMARH1144-14|Diptera|Sciaridae||BOLD:ACN0872
Sciaridae|[4713]|GMARA144-14|Diptera|Sciaridae||BOLD:ACM7878
Sciaridae|[4714]|GMAGJ286-15|Diptera|Sciaridae||BOLD:ACW9550
Sciaridae|[4715]|GMARE876-14|Diptera|Sciaridae||BOLD:ACN0241
Sciaridae|[4716]|GMARI923-14|Diptera|Sciaridae||BOLD:ACN1881
Sciaridae|[4717]|GMAGC979-15|Diptera|Sciaridae||BOLD:ACM9300
Sciaridae|[4718]|GMAGM141-15|Diptera|Sciaridae||BOLD:ACO0105
Sciaridae|[4719]|GMARK1876-14|Diptera|Sciaridae||BOLD:ACN1108
Sciaridae|[4720]|GMAGI505-15|Diptera|Sciaridae||BOLD:ACW4496
Sciaridae|[4721]|GMARG661-14|Diptera|Sciaridae||BOLD:ACN1565
Sciaridae|[4722]|GMARM570-14|Diptera|Sciaridae||BOLD:ACN5562
Sciaridae|[4723]|GMARA1239-14|Diptera|Sciaridae||BOLD:ACM4716
Sciaridae|[4724]|GMAGH357-15|Diptera|Sciaridae||BOLD:ACW1333
Sciaridae|[4725]|GMAGG2238-15|Diptera|Sciaridae||BOLD:ACN1004
Sciaridae|[4726]|GMARG901-14|Diptera|Sciaridae||BOLD:ACN1815
Sciaridae|[4727]|GMAGI268-15|Diptera|Sciaridae||BOLD:ACN2217
Sciaridae|[4728]|GMARK1398-14|Diptera|Sciaridae||BOLD:ACN2199
Sciaridae|[4729]|GMARD941-14|Diptera|Sciaridae||BOLD:ACM9583
Sciaridae|[4730]|GMARA302-14|Diptera|Sciaridae||BOLD:ACM4835
Sciaridae|[4731]|GMAGP1190-15|Diptera|Sciaridae||BOLD:ACX5997
Sciaridae|[4732]|GMARA1062-14|Diptera|Sciaridae||BOLD:ACM5611
Sciaridae|[4733]|GMARN902-14|Diptera|Sciaridae||BOLD:ACX3801
Sciaridae|[4734]|GMAGA402-15|Diptera|Sciaridae||BOLD:ACM8709
Sciaridae|[4735]|GMARA027-14|Diptera|Sciaridae||BOLD:ACM7685
Sciaridae|[4736]|GMARD662-14|Diptera|Sciaridae||BOLD:ACN1337
Sciaridae|[4737]|GMAGI394-15|Diptera|Sciaridae||BOLD:ACW3811
Sciaridae|[4738]|GMARB1423-14|Diptera|Sciaridae||BOLD:ACM5202
Sciaridae|[4739]|GMARE638-14|Diptera|Sciaridae||BOLD:ACM8353
Sciaridae|[4740]|GMAGK1784-15|Diptera|Sciaridae||BOLD:ACX5254
Sciaridae|[4741]|GMARB1377-14|Diptera|Sciaridae||BOLD:ACM5573
Sciaridae|[4742]|GMARD959-14|Diptera|Sciaridae||BOLD:ACM9834
Sciaridae|[4743]|GMAGX320-15|Diptera|Sciaridae||BOLD:ACX1700
Mycetophilidae|[4744]|GMAGD692-15|Diptera|Mycetophilidae||BOLD:ACW4737
Mycetophilidae|[4745]|GMAGD139-15|Diptera|Mycetophilidae||BOLD:ACW5217
Mycetophilidae|[4746]|GMARD1402-14|Diptera|Mycetophilidae||BOLD:ACM9618
Mycetophilidae|[4747]|GMARA390-14|Diptera|Mycetophilidae||BOLD:ACM9800
Mycetophilidae|[4748]|GMARW029-15|Diptera|Mycetophilidae||BOLD:ACX3875
Phylloicus pirapo|[4749]|GMARJ029-14|Trichoptera|Calamoceratidae|Calamoceratinae|BOLD:AAL5390
Calamoceratidae|[4750]|GMARD063-14|Trichoptera|Calamoceratidae||BOLD:ACN1528
Hydroptilidae|[4751]|GMAGC488-15|Trichoptera|Hydroptilidae||BOLD:ACN7442
Hydroptilidae|[4752]|GMAGD1605-15|Trichoptera|Hydroptilidae||BOLD:ACN1628
Hydroptilidae|[4753]|GMAGF593-15|Trichoptera|Hydroptilidae||BOLD:ACN0214
Hydroptilidae|[4754]|GMAGB984-15|Trichoptera|Hydroptilidae||BOLD:ACN0953
Hydroptilidae|[4755]|GMART1674-14|Trichoptera|Hydroptilidae||BOLD:ACO0829
Helicopsychidae|[4756]|GMAGG383-15|Trichoptera|Helicopsychidae||BOLD:ACN0221
Endomychidae|[4757]|GMAGE1607-15|Coleoptera|Endomychidae||BOLD:ACW2030
Endomychidae|[4758]|GMAGE1625-15|Coleoptera|Endomychidae||BOLD:ACN1351
Endomychidae|[4759]|GMAGL1528-15|Coleoptera|Endomychidae||BOLD:ACW9603
Trichoptera|[4760]|GMARE1388-14|Trichoptera|||BOLD:ACN1703
Trichoptera|[4761]|GMAGN459-15|Trichoptera|||BOLD:ACX4795
Cecidomyiidae|[4762]|GMAGG1406-15|Diptera|Cecidomyiidae||BOLD:ACW4325
Cecidomyiidae|[4763]|GMARK641-14|Diptera|Cecidomyiidae||BOLD:ACN5813
Cecidomyiidae|[4764]|GMAGS697-15|Diptera|Cecidomyiidae||BOLD:ACX5440
Cecidomyiidae|[4765]|GMARA234-14|Diptera|Cecidomyiidae||BOLD:ACM8788
Cecidomyiidae|[4766]|GMARM1133-14|Diptera|Cecidomyiidae||BOLD:ACN9453
Cecidomyiidae|[4767]|GMAGD898-15|Diptera|Cecidomyiidae||BOLD:ACW2929
Cecidomyiidae|[4768]|GMARO810-14|Diptera|Cecidomyiidae||BOLD:ACO1129
Cecidomyiidae|[4769]|GMARB1036-14|Diptera|Cecidomyiidae||BOLD:ACM6563
Cecidomyiidae|[4770]|GMAGD2235-15|Diptera|Cecidomyiidae||BOLD:ACM7394
Cecidomyiidae|[4771]|GMARA1414-14|Diptera|Cecidomyiidae||BOLD:ACM8905
Cecidomyiidae|[4772]|GMAGQ205-15|Diptera|Cecidomyiidae||BOLD:ACN4827
Cecidomyiidae|[4773]|GMARO283-14|Diptera|Cecidomyiidae||BOLD:ACX6882
Cecidomyiidae|[4774]|GMARA1281-14|Diptera|Cecidomyiidae||BOLD:ACM9808
Cecidomyiidae|[4775]|GMAGQ1255-15|Diptera|Cecidomyiidae||BOLD:ACN9949
Cecidomyiidae|[4776]|GMART1574-14|Diptera|Cecidomyiidae||BOLD:ACO0271
Cecidomyiidae|[4777]|GMARN183-14|Diptera|Cecidomyiidae||BOLD:ACN7167
Cecidomyiidae|[4778]|GMART897-14|Diptera|Cecidomyiidae||BOLD:ACN9244
Cecidomyiidae|[4779]|GMARB775-14|Diptera|Cecidomyiidae||BOLD:ACM5648

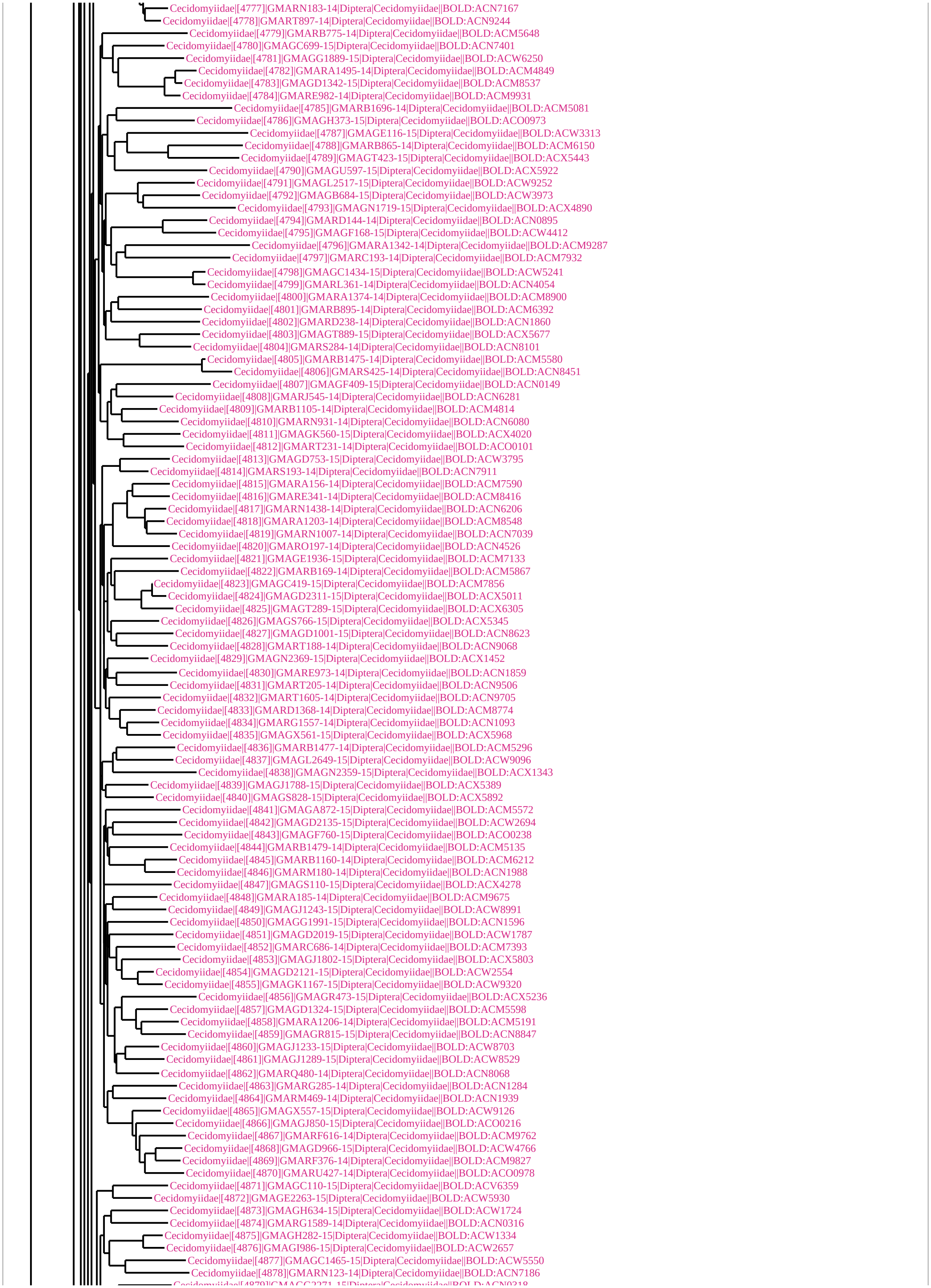
Cecidomyiidae|[4777]|GMARN183-14|Diptera|Cecidomyiidae||BOLD:ACN7167
Cecidomyiidae|[4778]|GMART897-14|Diptera|Cecidomyiidae||BOLD:ACN9244
Cecidomyiidae|[4779]|GMARB775-14|Diptera|Cecidomyiidae||BOLD:ACM5648
Cecidomyiidae|[4780]|GMAGC699-15|Diptera|Cecidomyiidae||BOLD:ACN7401
Cecidomyiidae|[4781]|GMAGG1889-15|Diptera|Cecidomyiidae||BOLD:ACW6250
Cecidomyiidae|[4782]|GMARA1495-14|Diptera|Cecidomyiidae||BOLD:ACM4849
Cecidomyiidae|[4783]|GMAGD1342-15|Diptera|Cecidomyiidae||BOLD:ACM8537
Cecidomyiidae|[4784]|GMARE982-14|Diptera|Cecidomyiidae||BOLD:ACM9931
Cecidomyiidae|[4785]|GMARB1696-14|Diptera|Cecidomyiidae||BOLD:ACM5081
Cecidomyiidae|[4786]|GMAGH373-15|Diptera|Cecidomyiidae||BOLD:ACO0973
Cecidomyiidae|[4787]|GMAGE116-15|Diptera|Cecidomyiidae||BOLD:ACW3313
Cecidomyiidae|[4788]|GMARB865-14|Diptera|Cecidomyiidae||BOLD:ACM6150
Cecidomyiidae|[4789]|GMAGT423-15|Diptera|Cecidomyiidae||BOLD:ACX5443
Cecidomyiidae|[4790]|GMAGU597-15|Diptera|Cecidomyiidae||BOLD:ACX5922
Cecidomyiidae|[4791]|GMAGL2517-15|Diptera|Cecidomyiidae||BOLD:ACW9252
Cecidomyiidae|[4792]|GMAGB684-15|Diptera|Cecidomyiidae||BOLD:ACW3973
Cecidomyiidae|[4793]|GMAGN1719-15|Diptera|Cecidomyiidae||BOLD:ACX4890
Cecidomyiidae|[4794]|GMARD144-14|Diptera|Cecidomyiidae||BOLD:ACN0895
Cecidomyiidae|[4795]|GMAGF168-15|Diptera|Cecidomyiidae||BOLD:ACW4412
Cecidomyiidae|[4796]|GMARA1342-14|Diptera|Cecidomyiidae||BOLD:ACM9287
Cecidomyiidae|[4797]|GMARC193-14|Diptera|Cecidomyiidae||BOLD:ACM7932
Cecidomyiidae|[4798]|GMAGC1434-15|Diptera|Cecidomyiidae||BOLD:ACW5241
Cecidomyiidae|[4799]|GMARL361-14|Diptera|Cecidomyiidae||BOLD:ACN4054
Cecidomyiidae|[4800]|GMARA1374-14|Diptera|Cecidomyiidae||BOLD:ACM8900
Cecidomyiidae|[4801]|GMARB895-14|Diptera|Cecidomyiidae||BOLD:ACM6392
Cecidomyiidae|[4802]|GMARD238-14|Diptera|Cecidomyiidae||BOLD:ACN1860
Cecidomyiidae|[4803]|GMAGT889-15|Diptera|Cecidomyiidae||BOLD:ACX5677
Cecidomyiidae|[4804]|GMARS284-14|Diptera|Cecidomyiidae||BOLD:ACN8101
Cecidomyiidae|[4805]|GMARB1475-14|Diptera|Cecidomyiidae||BOLD:ACM5580
Cecidomyiidae|[4806]|GMARS425-14|Diptera|Cecidomyiidae||BOLD:ACN8451
Cecidomyiidae|[4807]|GMAGF409-15|Diptera|Cecidomyiidae||BOLD:ACN0149
Cecidomyiidae|[4808]|GMARJ545-14|Diptera|Cecidomyiidae||BOLD:ACN6281
Cecidomyiidae|[4809]|GMARB1105-14|Diptera|Cecidomyiidae||BOLD:ACM4814
Cecidomyiidae|[4810]|GMARN931-14|Diptera|Cecidomyiidae||BOLD:ACN6080
Cecidomyiidae|[4811]|GMAGK560-15|Diptera|Cecidomyiidae||BOLD:ACX4020
Cecidomyiidae|[4812]|GMART231-14|Diptera|Cecidomyiidae||BOLD:ACO0101
Cecidomyiidae|[4813]|GMAGD753-15|Diptera|Cecidomyiidae||BOLD:ACW3795
Cecidomyiidae|[4814]|GMARS193-14|Diptera|Cecidomyiidae||BOLD:ACN7911
Cecidomyiidae|[4815]|GMARA156-14|Diptera|Cecidomyiidae||BOLD:ACM7590
Cecidomyiidae|[4816]|GMARE341-14|Diptera|Cecidomyiidae||BOLD:ACM8416
Cecidomyiidae|[4817]|GMARN1438-14|Diptera|Cecidomyiidae||BOLD:ACN6206
Cecidomyiidae|[4818]|GMARA1203-14|Diptera|Cecidomyiidae||BOLD:ACM8548
Cecidomyiidae|[4819]|GMARN1007-14|Diptera|Cecidomyiidae||BOLD:ACN7039
Cecidomyiidae|[4820]|GMARO197-14|Diptera|Cecidomyiidae||BOLD:ACN4526
Cecidomyiidae|[4821]|GMAGE1936-15|Diptera|Cecidomyiidae||BOLD:ACM7133
Cecidomyiidae|[4822]|GMARB169-14|Diptera|Cecidomyiidae||BOLD:ACM5867
Cecidomyiidae|[4823]|GMAGC419-15|Diptera|Cecidomyiidae||BOLD:ACM7856
Cecidomyiidae|[4824]|GMAGD2311-15|Diptera|Cecidomyiidae||BOLD:ACX5011
Cecidomyiidae|[4825]|GMAGT289-15|Diptera|Cecidomyiidae||BOLD:ACX6305
Cecidomyiidae|[4826]|GMAGS766-15|Diptera|Cecidomyiidae||BOLD:ACX5345
Cecidomyiidae|[4827]|GMAGD1001-15|Diptera|Cecidomyiidae||BOLD:ACN8623
Cecidomyiidae|[4828]|GMART188-14|Diptera|Cecidomyiidae||BOLD:ACN9068
Cecidomyiidae|[4829]|GMAGN2369-15|Diptera|Cecidomyiidae||BOLD:ACX1452
Cecidomyiidae|[4830]|GMARE973-14|Diptera|Cecidomyiidae||BOLD:ACN1859
Cecidomyiidae|[4831]|GMART205-14|Diptera|Cecidomyiidae||BOLD:ACN9506
Cecidomyiidae|[4832]|GMART1605-14|Diptera|Cecidomyiidae||BOLD:ACN9705
Cecidomyiidae|[4833]|GMARD1368-14|Diptera|Cecidomyiidae||BOLD:ACM8774
Cecidomyiidae|[4834]|GMARG1557-14|Diptera|Cecidomyiidae||BOLD:ACN1093
Cecidomyiidae|[4835]|GMAGX561-15|Diptera|Cecidomyiidae||BOLD:ACX5968
Cecidomyiidae|[4836]|GMARB1477-14|Diptera|Cecidomyiidae||BOLD:ACM5296
Cecidomyiidae|[4837]|GMAGL2649-15|Diptera|Cecidomyiidae||BOLD:ACW9096
Cecidomyiidae|[4838]|GMAGN2359-15|Diptera|Cecidomyiidae||BOLD:ACX1343
Cecidomyiidae|[4839]|GMAGJ1788-15|Diptera|Cecidomyiidae||BOLD:ACX5389
Cecidomyiidae|[4840]|GMAGS828-15|Diptera|Cecidomyiidae||BOLD:ACX5892
Cecidomyiidae|[4841]|GMAGA872-15|Diptera|Cecidomyiidae||BOLD:ACM5572
Cecidomyiidae|[4842]|GMAGD2135-15|Diptera|Cecidomyiidae||BOLD:ACW2694
Cecidomyiidae|[4843]|GMAGF760-15|Diptera|Cecidomyiidae||BOLD:ACO0238
Cecidomyiidae|[4844]|GMARB1479-14|Diptera|Cecidomyiidae||BOLD:ACM5135
Cecidomyiidae|[4845]|GMARB1160-14|Diptera|Cecidomyiidae||BOLD:ACM6212
Cecidomyiidae|[4846]|GMARM180-14|Diptera|Cecidomyiidae||BOLD:ACN1988
Cecidomyiidae|[4847]|GMAGS110-15|Diptera|Cecidomyiidae||BOLD:ACX4278
Cecidomyiidae|[4848]|GMARA185-14|Diptera|Cecidomyiidae||BOLD:ACM9675
Cecidomyiidae|[4849]|GMAGJ1243-15|Diptera|Cecidomyiidae||BOLD:ACW8991
Cecidomyiidae|[4850]|GMAGG1991-15|Diptera|Cecidomyiidae||BOLD:ACN1596
Cecidomyiidae|[4851]|GMAGD2019-15|Diptera|Cecidomyiidae||BOLD:ACW1787
Cecidomyiidae|[4852]|GMARC686-14|Diptera|Cecidomyiidae||BOLD:ACM7393
Cecidomyiidae|[4853]|GMAGJ1802-15|Diptera|Cecidomyiidae||BOLD:ACX5803
Cecidomyiidae|[4854]|GMAGD2121-15|Diptera|Cecidomyiidae||BOLD:ACW2554
Cecidomyiidae|[4855]|GMAGK1167-15|Diptera|Cecidomyiidae||BOLD:ACW9320
Cecidomyiidae|[4856]|GMAGR473-15|Diptera|Cecidomyiidae||BOLD:ACX5236
Cecidomyiidae|[4857]|GMAGD1324-15|Diptera|Cecidomyiidae||BOLD:ACM5598
Cecidomyiidae|[4858]|GMARA1206-14|Diptera|Cecidomyiidae||BOLD:ACM5191
Cecidomyiidae|[4859]|GMAGR815-15|Diptera|Cecidomyiidae||BOLD:ACN8847
Cecidomyiidae|[4860]|GMAGJ1233-15|Diptera|Cecidomyiidae||BOLD:ACW8703
Cecidomyiidae|[4861]|GMAGJ1289-15|Diptera|Cecidomyiidae||BOLD:ACW8529
Cecidomyiidae|[4862]|GMARQ480-14|Diptera|Cecidomyiidae||BOLD:ACN8068
Cecidomyiidae|[4863]|GMARG285-14|Diptera|Cecidomyiidae||BOLD:ACN1284
Cecidomyiidae|[4864]|GMARM469-14|Diptera|Cecidomyiidae||BOLD:ACN1939
Cecidomyiidae|[4865]|GMAGX557-15|Diptera|Cecidomyiidae||BOLD:ACW9126
Cecidomyiidae|[4866]|GMAGJ850-15|Diptera|Cecidomyiidae||BOLD:ACO0216
Cecidomyiidae|[4867]|GMARF616-14|Diptera|Cecidomyiidae||BOLD:ACM9762
Cecidomyiidae|[4868]|GMAGD966-15|Diptera|Cecidomyiidae||BOLD:ACW4766
Cecidomyiidae|[4869]|GMARF376-14|Diptera|Cecidomyiidae||BOLD:ACM9827
Cecidomyiidae|[4870]|GMARU427-14|Diptera|Cecidomyiidae||BOLD:ACO0978
Cecidomyiidae|[4871]|GMAGC110-15|Diptera|Cecidomyiidae||BOLD:ACV6359
Cecidomyiidae|[4872]|GMAGE2263-15|Diptera|Cecidomyiidae||BOLD:ACW5930
Cecidomyiidae|[4873]|GMAGH634-15|Diptera|Cecidomyiidae||BOLD:ACW1724
Cecidomyiidae|[4874]|GMARG1589-14|Diptera|Cecidomyiidae||BOLD:ACN0316
Cecidomyiidae|[4875]|GMAGH282-15|Diptera|Cecidomyiidae||BOLD:ACW1334
Cecidomyiidae|[4876]|GMAGI986-15|Diptera|Cecidomyiidae||BOLD:ACW2657
Cecidomyiidae|[4877]|GMAGC1465-15|Diptera|Cecidomyiidae||BOLD:ACW5550
Cecidomyiidae|[4878]|GMARN123-14|Diptera|Cecidomyiidae||BOLD:ACN7186

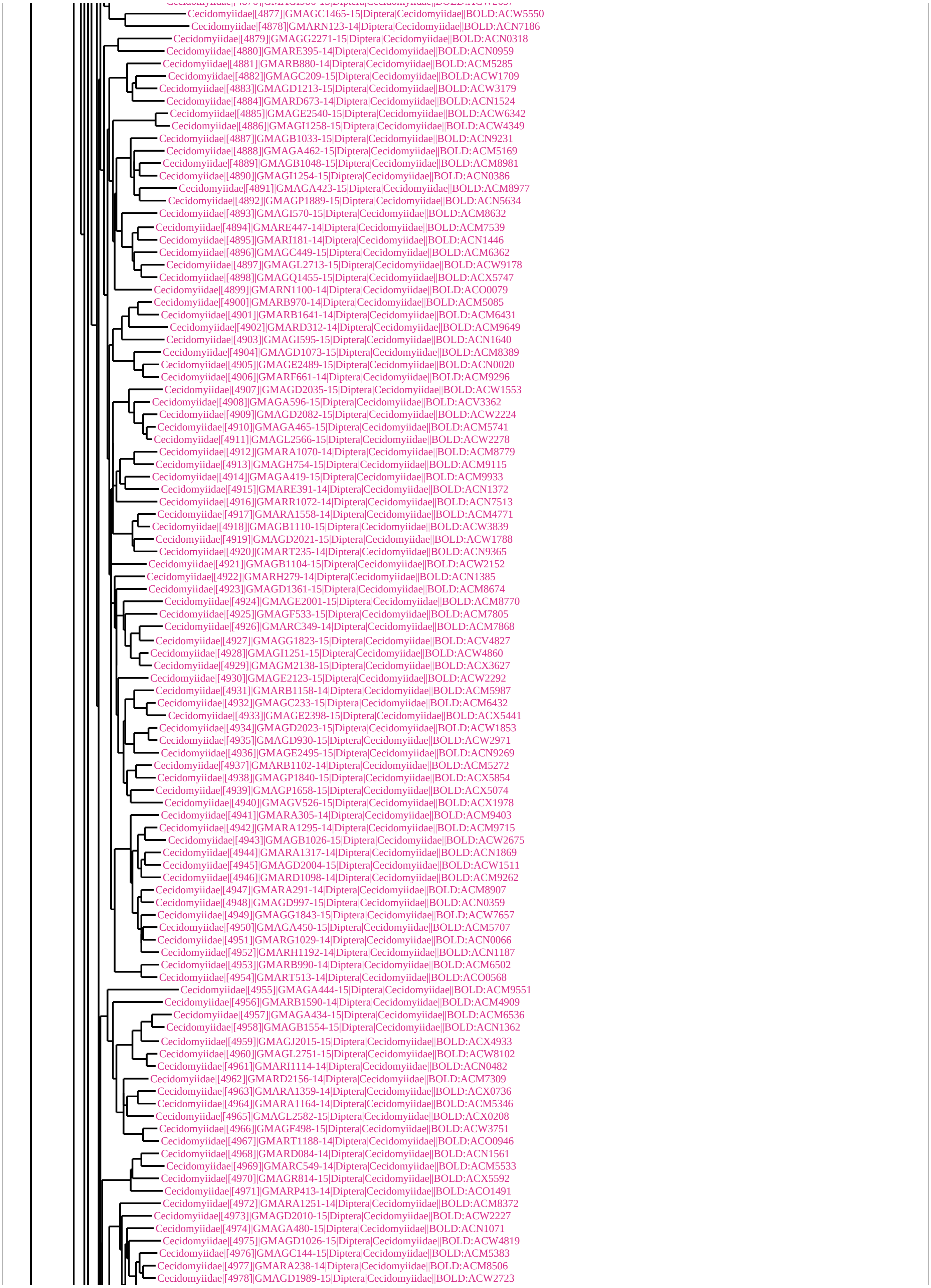
Cecidomyiidae|[4877]|GMAGC1465-15|Diptera|Cecidomyiidae||BOLD:ACW5550
Cecidomyiidae|[4878]|GMARN123-14|Diptera|Cecidomyiidae||BOLD:ACN7186
Cecidomyiidae|[4879]|GMAGG2271-15|Diptera|Cecidomyiidae||BOLD:ACN0318
Cecidomyiidae|[4880]|GMARE395-14|Diptera|Cecidomyiidae||BOLD:ACN0959
Cecidomyiidae|[4881]|GMARB880-14|Diptera|Cecidomyiidae||BOLD:ACM5285
Cecidomyiidae|[4882]|GMAGC209-15|Diptera|Cecidomyiidae||BOLD:ACW1709
Cecidomyiidae|[4883]|GMAGD1213-15|Diptera|Cecidomyiidae||BOLD:ACW3179
Cecidomyiidae|[4884]|GMARD673-14|Diptera|Cecidomyiidae||BOLD:ACN1524
Cecidomyiidae|[4885]|GMAGE2540-15|Diptera|Cecidomyiidae||BOLD:ACW6342
Cecidomyiidae|[4886]|GMAGI1258-15|Diptera|Cecidomyiidae||BOLD:ACW4349
Cecidomyiidae|[4887]|GMAGB1033-15|Diptera|Cecidomyiidae||BOLD:ACN9231
Cecidomyiidae|[4888]|GMAGA462-15|Diptera|Cecidomyiidae||BOLD:ACM5169
Cecidomyiidae|[4889]|GMAGB1048-15|Diptera|Cecidomyiidae||BOLD:ACM8981
Cecidomyiidae|[4890]|GMAGI1254-15|Diptera|Cecidomyiidae||BOLD:ACN0386
Cecidomyiidae|[4891]|GMAGA423-15|Diptera|Cecidomyiidae||BOLD:ACM8977
Cecidomyiidae|[4892]|GMAGP1889-15|Diptera|Cecidomyiidae||BOLD:ACN5634
Cecidomyiidae|[4893]|GMAGI570-15|Diptera|Cecidomyiidae||BOLD:ACM8632
Cecidomyiidae|[4894]|GMARE447-14|Diptera|Cecidomyiidae||BOLD:ACM7539
Cecidomyiidae|[4895]|GMARI181-14|Diptera|Cecidomyiidae||BOLD:ACN1446
Cecidomyiidae|[4896]|GMAGC449-15|Diptera|Cecidomyiidae||BOLD:ACM6362
Cecidomyiidae|[4897]|GMAGL2713-15|Diptera|Cecidomyiidae||BOLD:ACW9178
Cecidomyiidae|[4898]|GMAGQ1455-15|Diptera|Cecidomyiidae||BOLD:ACX5747
Cecidomyiidae|[4899]|GMARN1100-14|Diptera|Cecidomyiidae||BOLD:ACO0079
Cecidomyiidae|[4900]|GMARB970-14|Diptera|Cecidomyiidae||BOLD:ACM5085
Cecidomyiidae|[4901]|GMARB1641-14|Diptera|Cecidomyiidae||BOLD:ACM6431
Cecidomyiidae|[4902]|GMARD312-14|Diptera|Cecidomyiidae||BOLD:ACM9649
Cecidomyiidae|[4903]|GMAGI595-15|Diptera|Cecidomyiidae||BOLD:ACN1640
Cecidomyiidae|[4904]|GMAGD1073-15|Diptera|Cecidomyiidae||BOLD:ACM8389
Cecidomyiidae|[4905]|GMAGE2489-15|Diptera|Cecidomyiidae||BOLD:ACN0020
Cecidomyiidae|[4906]|GMARF661-14|Diptera|Cecidomyiidae||BOLD:ACM9296
Cecidomyiidae|[4907]|GMAGD2035-15|Diptera|Cecidomyiidae||BOLD:ACW1553
Cecidomyiidae|[4908]|GMAGA596-15|Diptera|Cecidomyiidae||BOLD:ACV3362
Cecidomyiidae|[4909]|GMAGD2082-15|Diptera|Cecidomyiidae||BOLD:ACW2224
Cecidomyiidae|[4910]|GMAGA465-15|Diptera|Cecidomyiidae||BOLD:ACM5741
Cecidomyiidae|[4911]|GMAGL2566-15|Diptera|Cecidomyiidae||BOLD:ACW2278
Cecidomyiidae|[4912]|GMARA1070-14|Diptera|Cecidomyiidae||BOLD:ACM8779
Cecidomyiidae|[4913]|GMAGH754-15|Diptera|Cecidomyiidae||BOLD:ACM9115
Cecidomyiidae|[4914]|GMAGA419-15|Diptera|Cecidomyiidae||BOLD:ACM9933
Cecidomyiidae|[4915]|GMARE391-14|Diptera|Cecidomyiidae||BOLD:ACN1372
Cecidomyiidae|[4916]|GMARR1072-14|Diptera|Cecidomyiidae||BOLD:ACN7513
Cecidomyiidae|[4917]|GMARA1558-14|Diptera|Cecidomyiidae||BOLD:ACM4771
Cecidomyiidae|[4918]|GMAGB1110-15|Diptera|Cecidomyiidae||BOLD:ACW3839
Cecidomyiidae|[4919]|GMAGD2021-15|Diptera|Cecidomyiidae||BOLD:ACW1788
Cecidomyiidae|[4920]|GMART235-14|Diptera|Cecidomyiidae||BOLD:ACN9365
Cecidomyiidae|[4921]|GMAGB1104-15|Diptera|Cecidomyiidae||BOLD:ACW2152
Cecidomyiidae|[4922]|GMARH279-14|Diptera|Cecidomyiidae||BOLD:ACN1385
Cecidomyiidae|[4923]|GMAGD1361-15|Diptera|Cecidomyiidae||BOLD:ACM8674
Cecidomyiidae|[4924]|GMAGE2001-15|Diptera|Cecidomyiidae||BOLD:ACM8770
Cecidomyiidae|[4925]|GMAGF533-15|Diptera|Cecidomyiidae||BOLD:ACM7805
Cecidomyiidae|[4926]|GMARC349-14|Diptera|Cecidomyiidae||BOLD:ACM7868
Cecidomyiidae|[4927]|GMAGG1823-15|Diptera|Cecidomyiidae||BOLD:ACV4827
Cecidomyiidae|[4928]|GMAGI1251-15|Diptera|Cecidomyiidae||BOLD:ACW4860
Cecidomyiidae|[4929]|GMAGM2138-15|Diptera|Cecidomyiidae||BOLD:ACX3627
Cecidomyiidae|[4930]|GMAGE2123-15|Diptera|Cecidomyiidae||BOLD:ACW2292
Cecidomyiidae|[4931]|GMARB1158-14|Diptera|Cecidomyiidae||BOLD:ACM5987
Cecidomyiidae|[4932]|GMAGC233-15|Diptera|Cecidomyiidae||BOLD:ACM6432
Cecidomyiidae|[4933]|GMAGE2398-15|Diptera|Cecidomyiidae||BOLD:ACX5441
Cecidomyiidae|[4934]|GMAGD2023-15|Diptera|Cecidomyiidae||BOLD:ACW1853
Cecidomyiidae|[4935]|GMAGD930-15|Diptera|Cecidomyiidae||BOLD:ACW2971
Cecidomyiidae|[4936]|GMAGE2495-15|Diptera|Cecidomyiidae||BOLD:ACN9269
Cecidomyiidae|[4937]|GMARB1102-14|Diptera|Cecidomyiidae||BOLD:ACM5272
Cecidomyiidae|[4938]|GMAGP1840-15|Diptera|Cecidomyiidae||BOLD:ACX5854
Cecidomyiidae|[4939]|GMAGP1658-15|Diptera|Cecidomyiidae||BOLD:ACX5074
Cecidomyiidae|[4940]|GMAGV526-15|Diptera|Cecidomyiidae||BOLD:ACX1978
Cecidomyiidae|[4941]|GMARA305-14|Diptera|Cecidomyiidae||BOLD:ACM9403
Cecidomyiidae|[4942]|GMARA1295-14|Diptera|Cecidomyiidae||BOLD:ACM9715
Cecidomyiidae|[4943]|GMAGB1026-15|Diptera|Cecidomyiidae||BOLD:ACW2675
Cecidomyiidae|[4944]|GMARA1317-14|Diptera|Cecidomyiidae||BOLD:ACN1869
Cecidomyiidae|[4945]|GMAGD2004-15|Diptera|Cecidomyiidae||BOLD:ACW1511
Cecidomyiidae|[4946]|GMARD1098-14|Diptera|Cecidomyiidae||BOLD:ACM9262
Cecidomyiidae|[4947]|GMARA291-14|Diptera|Cecidomyiidae||BOLD:ACM8907
Cecidomyiidae|[4948]|GMAGD997-15|Diptera|Cecidomyiidae||BOLD:ACN0359
Cecidomyiidae|[4949]|GMAGG1843-15|Diptera|Cecidomyiidae||BOLD:ACW7657
Cecidomyiidae|[4950]|GMAGA450-15|Diptera|Cecidomyiidae||BOLD:ACM5707
Cecidomyiidae|[4951]|GMARG1029-14|Diptera|Cecidomyiidae||BOLD:ACN0066
Cecidomyiidae|[4952]|GMARH1192-14|Diptera|Cecidomyiidae||BOLD:ACN1187
Cecidomyiidae|[4953]|GMARB990-14|Diptera|Cecidomyiidae||BOLD:ACM6502
Cecidomyiidae|[4954]|GMART513-14|Diptera|Cecidomyiidae||BOLD:ACO0568
Cecidomyiidae|[4955]|GMAGA444-15|Diptera|Cecidomyiidae||BOLD:ACM9551
Cecidomyiidae|[4956]|GMARB1590-14|Diptera|Cecidomyiidae||BOLD:ACM4909
Cecidomyiidae|[4957]|GMAGA434-15|Diptera|Cecidomyiidae||BOLD:ACM6536
Cecidomyiidae|[4958]|GMAGB1554-15|Diptera|Cecidomyiidae||BOLD:ACN1362
Cecidomyiidae|[4959]|GMAGJ2015-15|Diptera|Cecidomyiidae||BOLD:ACX4933
Cecidomyiidae|[4960]|GMAGL2751-15|Diptera|Cecidomyiidae||BOLD:ACW8102
Cecidomyiidae|[4961]|GMARI1114-14|Diptera|Cecidomyiidae||BOLD:ACN0482
Cecidomyiidae|[4962]|GMARD2156-14|Diptera|Cecidomyiidae||BOLD:ACM7309
Cecidomyiidae|[4963]|GMARA1359-14|Diptera|Cecidomyiidae||BOLD:ACX0736
Cecidomyiidae|[4964]|GMARA1164-14|Diptera|Cecidomyiidae||BOLD:ACM5346
Cecidomyiidae|[4965]|GMAGL2582-15|Diptera|Cecidomyiidae||BOLD:ACX0208
Cecidomyiidae|[4966]|GMAGF498-15|Diptera|Cecidomyiidae||BOLD:ACW3751
Cecidomyiidae|[4967]|GMART1188-14|Diptera|Cecidomyiidae||BOLD:ACO0946
Cecidomyiidae|[4968]|GMARD084-14|Diptera|Cecidomyiidae||BOLD:ACN1561
Cecidomyiidae|[4969]|GMARC549-14|Diptera|Cecidomyiidae||BOLD:ACM5533
Cecidomyiidae|[4970]|GMAGR814-15|Diptera|Cecidomyiidae||BOLD:ACX5592
Cecidomyiidae|[4971]|GMARP413-14|Diptera|Cecidomyiidae||BOLD:ACO1491
Cecidomyiidae|[4972]|GMARA1251-14|Diptera|Cecidomyiidae||BOLD:ACM8372
Cecidomyiidae|[4973]|GMAGD2010-15|Diptera|Cecidomyiidae||BOLD:ACW2227
Cecidomyiidae|[4974]|GMAGA480-15|Diptera|Cecidomyiidae||BOLD:ACN1071
Cecidomyiidae|[4975]|GMAGD1026-15|Diptera|Cecidomyiidae||BOLD:ACW4819
Cecidomyiidae|[4976]|GMAGC144-15|Diptera|Cecidomyiidae||BOLD:ACM5383
Cecidomyiidae|[4977]|GMARA238-14|Diptera|Cecidomyiidae||BOLD:ACM8506
Cecidomyiidae|[4978]|GMAGD1989-15|Diptera|Cecidomyiidae||BOLD:ACW2723

Cecidomyiidae|[4976]|GMAGC144-15|Diptera|Cecidomyiidae||BOLD:ACM5383
Cecidomyiidae|[4977]|GMARA238-14|Diptera|Cecidomyiidae||BOLD:ACM8506
Cecidomyiidae|[4978]|GMAGD1989-15|Diptera|Cecidomyiidae||BOLD:ACW2723
Cecidomyiidae|[4979]|GMAGH254-15|Diptera|Cecidomyiidae||BOLD:ACW2172
Cecidomyiidae|[4980]|GMARD615-14|Diptera|Cecidomyiidae||BOLD:ACN1404
Cecidomyiidae|[4981]|GMARD211-14|Diptera|Cecidomyiidae||BOLD:ACM7988
Cecidomyiidae|[4982]|GMARB1752-14|Diptera|Cecidomyiidae||BOLD:ACM6365
Cecidomyiidae|[4983]|GMAGD1169-15|Diptera|Cecidomyiidae||BOLD:ACW4747
Cecidomyiidae|[4984]|GMAGL1886-15|Diptera|Cecidomyiidae||BOLD:ACW7838
Cecidomyiidae|[4985]|GMAGE181-15|Diptera|Cecidomyiidae||BOLD:ACW3383
Cecidomyiidae|[4986]|GMAGK1302-15|Diptera|Cecidomyiidae||BOLD:ACN9536
Cecidomyiidae|[4987]|GMAGB570-15|Diptera|Cecidomyiidae||BOLD:ACM6407
Cecidomyiidae|[4988]|GMAGC799-15|Diptera|Cecidomyiidae||BOLD:ACM9534
Cecidomyiidae|[4989]|GMAGC181-15|Diptera|Cecidomyiidae||BOLD:ACN1861
Cecidomyiidae|[4990]|GMARD589-14|Diptera|Cecidomyiidae||BOLD:ACM8585
Cecidomyiidae|[4991]|GMART773-14|Diptera|Cecidomyiidae||BOLD:ACN9096
Cecidomyiidae|[4992]|GMAGB800-15|Diptera|Cecidomyiidae||BOLD:ACW7644
Cecidomyiidae|[4993]|GMAGD1064-15|Diptera|Cecidomyiidae||BOLD:ACW3635
Cecidomyiidae|[4994]|GMARB1088-14|Diptera|Cecidomyiidae||BOLD:ACM5176
Cecidomyiidae|[4995]|GMARA1428-14|Diptera|Cecidomyiidae||BOLD:ACM9687
Cecidomyiidae|[4996]|GMAGD627-15|Diptera|Cecidomyiidae||BOLD:ACX2443
Cecidomyiidae|[4997]|GMARD1426-14|Diptera|Cecidomyiidae||BOLD:ACM8706
Cecidomyiidae|[4998]|GMAGD1135-15|Diptera|Cecidomyiidae||BOLD:ACW3156
Cecidomyiidae|[4999]|GMARA178-14|Diptera|Cecidomyiidae||BOLD:ACM4973
Cecidomyiidae|[5000]|GMARA1132-14|Diptera|Cecidomyiidae||BOLD:ACM9336
Cecidomyiidae|[5001]|GMAGB670-15|Diptera|Cecidomyiidae||BOLD:ACW3805
Cecidomyiidae|[5002]|GMAGC106-15|Diptera|Cecidomyiidae||BOLD:ACM4812
Cecidomyiidae|[5003]|GMARD081-14|Diptera|Cecidomyiidae||BOLD:ACN0289
Cecidomyiidae|[5004]|GMAGC288-15|Diptera|Cecidomyiidae||BOLD:ACW2739
Cecidomyiidae|[5005]|GMAGA487-15|Diptera|Cecidomyiidae||BOLD:ACV2022
Cecidomyiidae|[5006]|GMARB648-14|Diptera|Cecidomyiidae||BOLD:ACM6001
Cecidomyiidae|[5007]|GMARB1387-14|Diptera|Cecidomyiidae||BOLD:ACM5570
Cecidomyiidae|[5008]|GMARD570-14|Diptera|Cecidomyiidae||BOLD:ACN1343
Cecidomyiidae|[5009]|GMAGD1088-15|Diptera|Cecidomyiidae||BOLD:ACN8796
Cecidomyiidae|[5010]|GMAGD296-15|Diptera|Cecidomyiidae||BOLD:ACW5237
Cecidomyiidae|[5011]|GMAGJ1643-15|Diptera|Cecidomyiidae||BOLD:ACW8137
Cecidomyiidae|[5012]|GMAGA533-15|Diptera|Cecidomyiidae||BOLD:ACV3038
Cecidomyiidae|[5013]|GMARN587-14|Diptera|Cecidomyiidae||BOLD:ACN9719
Cecidomyiidae|[5014]|GMAGH1130-15|Diptera|Cecidomyiidae||BOLD:ACN1880
Cecidomyiidae|[5015]|GMAGJ1762-15|Diptera|Cecidomyiidae||BOLD:ACN0197
Cecidomyiidae|[5016]|GMAGN2266-15|Diptera|Cecidomyiidae||BOLD:ACX3314
Cecidomyiidae|[5017]|GMAGB654-15|Diptera|Cecidomyiidae||BOLD:ACM5672
Cecidomyiidae|[5018]|GMAGL2493-15|Diptera|Cecidomyiidae||BOLD:ACW9215
Cecidomyiidae|[5019]|GMAGM1649-15|Diptera|Cecidomyiidae||BOLD:ACX4049
Cecidomyiidae|[5020]|GMARL286-14|Diptera|Cecidomyiidae||BOLD:ACN1977
Cecidomyiidae|[5021]|GMAGA591-15|Diptera|Cecidomyiidae||BOLD:ACV2830
Cecidomyiidae|[5022]|GMAGJ1133-15|Diptera|Cecidomyiidae||BOLD:ACW8471
Cecidomyiidae|[5023]|GMAGP1856-15|Diptera|Cecidomyiidae||BOLD:ACX5270
Cecidomyiidae|[5024]|GMAGC251-15|Diptera|Cecidomyiidae||BOLD:ACY4330
Cecidomyiidae|[5025]|GMARR1090-14|Diptera|Cecidomyiidae||BOLD:ACN7619
Cecidomyiidae|[5026]|GMARA1217-14|Diptera|Cecidomyiidae||BOLD:ACM9394
Cecidomyiidae|[5027]|GMARD724-14|Diptera|Cecidomyiidae||BOLD:ACN1150
Cecidomyiidae|[5028]|GMARA1275-14|Diptera|Cecidomyiidae||BOLD:ACM5544
Cecidomyiidae|[5029]|GMARB903-14|Diptera|Cecidomyiidae||BOLD:ACM8553
Cecidomyiidae|[5030]|GMAGE2416-15|Diptera|Cecidomyiidae||BOLD:ACM9602
Cecidomyiidae|[5031]|GMAGA142-15|Diptera|Cecidomyiidae||BOLD:ACM7853
Cecidomyiidae|[5032]|GMARB1480-14|Diptera|Cecidomyiidae||BOLD:ACM5004
Cecidomyiidae|[5033]|GMAGP1777-15|Diptera|Cecidomyiidae||BOLD:ACX5130
Cecidomyiidae|[5034]|GMAGB857-15|Diptera|Cecidomyiidae||BOLD:ACM8711
Cecidomyiidae|[5035]|GMARM1065-14|Diptera|Cecidomyiidae||BOLD:ACN9396
Cecidomyiidae|[5036]|GMAGA557-15|Diptera|Cecidomyiidae||BOLD:ACM6013
Cecidomyiidae|[5037]|GMART733-14|Diptera|Cecidomyiidae||BOLD:ACO0334
Cecidomyiidae|[5038]|GMARA1272-14|Diptera|Cecidomyiidae||BOLD:ACM5745
Cecidomyiidae|[5039]|GMAGC137-15|Diptera|Cecidomyiidae||BOLD:ACN0452
Cecidomyiidae|[5040]|GMAGE2244-15|Diptera|Cecidomyiidae||BOLD:ACW2825
Cecidomyiidae|[5041]|GMAGG1325-15|Diptera|Cecidomyiidae||BOLD:ACW3434
Cecidomyiidae|[5042]|GMARD830-14|Diptera|Cecidomyiidae||BOLD:ACM9112
Cecidomyiidae|[5043]|GMAGN1526-15|Diptera|Cecidomyiidae||BOLD:ACX2669
Cecidomyiidae|[5044]|GMAGD2281-15|Diptera|Cecidomyiidae||BOLD:ACN1963
Cecidomyiidae|[5045]|GMAGE2286-15|Diptera|Cecidomyiidae||BOLD:ACM9436
Cecidomyiidae|[5046]|GMARF515-14|Diptera|Cecidomyiidae||BOLD:ACM9824
Cecidomyiidae|[5047]|GMARA1517-14|Diptera|Cecidomyiidae||BOLD:ACM6518
Cecidomyiidae|[5048]|GMARP202-14|Diptera|Cecidomyiidae||BOLD:ACO0129
Cecidomyiidae|[5049]|GMAGA422-15|Diptera|Cecidomyiidae||BOLD:ACM5864
Cecidomyiidae|[5050]|GMARK1194-14|Diptera|Cecidomyiidae||BOLD:ACN1016
Cecidomyiidae|[5051]|GMARA1167-14|Diptera|Cecidomyiidae||BOLD:ACM9796
Cecidomyiidae|[5052]|GMAGC431-15|Diptera|Cecidomyiidae||BOLD:ACN1975
Cecidomyiidae|[5053]|GMARA034-14|Diptera|Cecidomyiidae||BOLD:ACM8510
Cecidomyiidae|[5054]|GMARL830-14|Diptera|Cecidomyiidae||BOLD:ACN2955
Cecidomyiidae|[5055]|GMART1443-14|Diptera|Cecidomyiidae||BOLD:ACN9638
Cecidomyiidae|[5056]|GMARB959-14|Diptera|Cecidomyiidae||BOLD:ACM5563
Cecidomyiidae|[5057]|GMARA1429-14|Diptera|Cecidomyiidae||BOLD:ACM5869
Cecidomyiidae|[5058]|GMARC662-14|Diptera|Cecidomyiidae||BOLD:ACM7176
Cecidomyiidae|[5059]|GMARK108-14|Diptera|Cecidomyiidae||BOLD:ACN1029
Cecidomyiidae|[5060]|GMAGE2567-15|Diptera|Cecidomyiidae||BOLD:ACY4978
Cecidomyiidae|[5061]|GMARM1021-14|Diptera|Cecidomyiidae||BOLD:ACN8690
Cecidomyiidae|[5062]|GMAGD935-15|Diptera|Cecidomyiidae||BOLD:ACN8653
Cecidomyiidae|[5063]|GMAGD1028-15|Diptera|Cecidomyiidae||BOLD:ACM7911
Cecidomyiidae|[5064]|GMARA076-14|Diptera|Cecidomyiidae||BOLD:ACM8487
Cecidomyiidae|[5065]|GMARE892-14|Diptera|Cecidomyiidae||BOLD:ACN0544
Cecidomyiidae|[5066]|GMAGD1294-15|Diptera|Cecidomyiidae||BOLD:ACM9318
Cecidomyiidae|[5067]|GMARE814-14|Diptera|Cecidomyiidae||BOLD:ACN1067
Cecidomyiidae|[5068]|GMARP199-14|Diptera|Cecidomyiidae||BOLD:ACO0012
Cecidomyiidae|[5069]|GMARB1557-14|Diptera|Cecidomyiidae||BOLD:ACM5858
Cecidomyiidae|[5070]|GMAGB771-15|Diptera|Cecidomyiidae||BOLD:ACW5617
Cecidomyiidae|[5071]|GMARA1180-14|Diptera|Cecidomyiidae||BOLD:ACM8831
Cecidomyiidae|[5072]|GMARW045-15|Diptera|Cecidomyiidae||BOLD:ACX0979
Cecidomyiidae|[5073]|GMAGJ1124-15|Diptera|Cecidomyiidae||BOLD:ACN1047
Cecidomyiidae|[5074]|GMARB908-14|Diptera|Cecidomyiidae||BOLD:ACM5636
Cecidomyiidae|[5075]|GMAGF386-15|Diptera|Cecidomyiidae||BOLD:ACW4197
Cecidomyiidae|[5076]|GMARD900-14|Diptera|Cecidomyiidae||BOLD:ACW3049
Cecidomyiidae|[5077]|GMAGF558-15|Diptera|Cecidomyiidae||BOLD:ACM7809
Cecidomyiidae|[5078]|GMAGD2329-15|Diptera|Cecidomyiidae||BOLD:ACM8872

[illegible]
Cecidomyiidae|[5076]|GMARD900-14|Diptera|Cecidomyiidae||BOLD:ACW3049
Cecidomyiidae|[5077]|GMAGF558-15|Diptera|Cecidomyiidae||BOLD:ACM7809
Cecidomyiidae|[5078]|GMAGD2329-15|Diptera|Cecidomyiidae||BOLD:ACM8872
Cecidomyiidae|[5079]|GMAGM1947-15|Diptera|Cecidomyiidae||BOLD:ACX3452
Cecidomyiidae|[5080]|GMARE931-14|Diptera|Cecidomyiidae||BOLD:ACN1579
Cecidomyiidae|[5081]|GMAGA455-15|Diptera|Cecidomyiidae||BOLD:ACN1578
Cecidomyiidae|[5082]|GMARB937-14|Diptera|Cecidomyiidae||BOLD:ACM5256
Cecidomyiidae|[5083]|GMAGJ1700-15|Diptera|Cecidomyiidae||BOLD:ACN1198
Cecidomyiidae|[5084]|GMAGE2040-15|Diptera|Cecidomyiidae||BOLD:ACM9484
Cecidomyiidae|[5085]|GMAGM2055-15|Diptera|Cecidomyiidae||BOLD:ACN9495
Cecidomyiidae|[5086]|GMAGN2137-15|Diptera|Cecidomyiidae||BOLD:ACN1717
Cecidomyiidae|[5087]|GMARA1565-14|Diptera|Cecidomyiidae||BOLD:ACM5738
Cecidomyiidae|[5088]|GMAGE2058-15|Diptera|Cecidomyiidae||BOLD:ACN0574
Cecidomyiidae|[5089]|GMAGE1732-15|Diptera|Cecidomyiidae||BOLD:ACW1785
Cecidomyiidae|[5090]|GMAGC787-15|Diptera|Cecidomyiidae||BOLD:ACW2204
Cecidomyiidae|[5091]|GMARH071-14|Diptera|Cecidomyiidae||BOLD:ACN0545
Cecidomyiidae|[5092]|GMARB1421-14|Diptera|Cecidomyiidae||BOLD:ACM5200
Cecidomyiidae|[5093]|GMARA1101-14|Diptera|Cecidomyiidae||BOLD:ACM9289
Cecidomyiidae|[5094]|GMAGP1614-15|Diptera|Cecidomyiidae||BOLD:ACX5139
Cecidomyiidae|[5095]|GMAGA482-15|Diptera|Cecidomyiidae||BOLD:ACN1453
Cecidomyiidae|[5096]|GMARA375-14|Diptera|Cecidomyiidae||BOLD:ACM5243
Cecidomyiidae|[5097]|GMARA1079-14|Diptera|Cecidomyiidae||BOLD:ACM8485
Cecidomyiidae|[5098]|GMAGC959-15|Diptera|Cecidomyiidae||BOLD:ACW0077
Cecidomyiidae|[5099]|GMARD636-14|Diptera|Cecidomyiidae||BOLD:ACN1821
Cecidomyiidae|[5100]|GMAGA410-15|Diptera|Cecidomyiidae||BOLD:ACM9215
Cecidomyiidae|[5101]|GMAGB797-15|Diptera|Cecidomyiidae||BOLD:ACW7929
Cecidomyiidae|[5102]|GMAGB459-15|Diptera|Cecidomyiidae||BOLD:ACM9562
Cecidomyiidae|[5103]|GMAGE2003-15|Diptera|Cecidomyiidae||BOLD:ACW6019
Cecidomyiidae|[5104]|GMAGE2264-15|Diptera|Cecidomyiidae||BOLD:ACN2561
Cecidomyiidae|[5105]|GMAGF557-15|Diptera|Cecidomyiidae||BOLD:ACW4836
Cecidomyiidae|[5106]|GMAGA570-15|Diptera|Cecidomyiidae||BOLD:ACM5208
Cecidomyiidae|[5107]|GMAGA547-15|Diptera|Cecidomyiidae||BOLD:ACM5147
Cecidomyiidae|[5108]|GMAGG1451-15|Diptera|Cecidomyiidae||BOLD:ACN2671
Cecidomyiidae|[5109]|GMARD1473-14|Diptera|Cecidomyiidae||BOLD:ACN0040
Cecidomyiidae|[5110]|GMAGE2063-15|Diptera|Cecidomyiidae||BOLD:ACM9962
Cecidomyiidae|[5111]|GMARL800-14|Diptera|Cecidomyiidae||BOLD:ACN9886
Cecidomyiidae|[5112]|GMAGC1000-15|Diptera|Cecidomyiidae||BOLD:ACV6721
Cecidomyiidae|[5113]|GMARD251-14|Diptera|Cecidomyiidae||BOLD:ACN1555
Cecidomyiidae|[5114]|GMAGJ1180-15|Diptera|Cecidomyiidae||BOLD:ACW9298
Cecidomyiidae|[5115]|GMAGI567-15|Diptera|Cecidomyiidae||BOLD:ACW7025
Cecidomyiidae|[5116]|GMAGB665-15|Diptera|Cecidomyiidae||BOLD:ACM4724
Cecidomyiidae|[5117]|GMARD820-14|Diptera|Cecidomyiidae||BOLD:ACM8849
Cecidomyiidae|[5118]|GMAGK1351-15|Diptera|Cecidomyiidae||BOLD:ACW8283
Cecidomyiidae|[5119]|GMARF816-14|Diptera|Cecidomyiidae||BOLD:ACM9301
Cecidomyiidae|[5120]|GMAGM577-15|Diptera|Cecidomyiidae||BOLD:ACX3976
Cecidomyiidae|[5121]|GMAGQ1760-15|Diptera|Cecidomyiidae||BOLD:ACX5956
Cecidomyiidae|[5122]|GMAGH726-15|Diptera|Cecidomyiidae||BOLD:ACN2710
Cecidomyiidae|[5123]|GMARA1574-14|Diptera|Cecidomyiidae||BOLD:ACM5742
Cecidomyiidae|[5124]|GMARC737-14|Diptera|Cecidomyiidae||BOLD:ACM5345
Cecidomyiidae|[5125]|GMAGL1664-15|Diptera|Cecidomyiidae||BOLD:ACN1179
Cecidomyiidae|[5126]|GMAGC229-15|Diptera|Cecidomyiidae||BOLD:ACW2740
Cecidomyiidae|[5127]|GMAGE2110-15|Diptera|Cecidomyiidae||BOLD:ACW7661
Cecidomyiidae|[5128]|GMAGX601-15|Diptera|Cecidomyiidae||BOLD:ACX5398
Cecidomyiidae|[5129]|GMAGL2004-15|Diptera|Cecidomyiidae||BOLD:AAV5754
Cecidomyiidae|[5130]|GMAGA479-15|Diptera|Cecidomyiidae||BOLD:ACN8182
Cecidomyiidae|[5131]|GMAGC228-15|Diptera|Cecidomyiidae||BOLD:ACW2697
Cecidomyiidae|[5132]|GMARD1449-14|Diptera|Cecidomyiidae||BOLD:ACM7210
Cecidomyiidae|[5133]|GMAGJ1674-15|Diptera|Cecidomyiidae||BOLD:ACW9245
Cecidomyiidae|[5134]|GMARA1080-14|Diptera|Cecidomyiidae||BOLD:ACM5669
Cecidomyiidae|[5135]|GMAGE2521-15|Diptera|Cecidomyiidae||BOLD:ACW6173
Cecidomyiidae|[5136]|GMAGD1039-15|Diptera|Cecidomyiidae||BOLD:ACW3119
Cecidomyiidae|[5137]|GMAGG1428-15|Diptera|Cecidomyiidae||BOLD:ACW3365
Cecidomyiidae|[5138]|GMARA043-14|Diptera|Cecidomyiidae||BOLD:ACM7549
Cecidomyiidae|[5139]|GMAGC263-15|Diptera|Cecidomyiidae||BOLD:ACM6158
Cecidomyiidae|[5140]|GMARE967-14|Diptera|Cecidomyiidae||BOLD:ACN0325
Cecidomyiidae|[5141]|GMAGC172-15|Diptera|Cecidomyiidae||BOLD:ACM8129
Cecidomyiidae|[5142]|GMAGD1149-15|Diptera|Cecidomyiidae||BOLD:ACN0891
Cecidomyiidae|[5143]|GMAGK2074-15|Diptera|Cecidomyiidae||BOLD:ACW9788
Cecidomyiidae|[5144]|GMAGG1950-15|Diptera|Cecidomyiidae||BOLD:ACW6212
Cecidomyiidae|[5145]|GMARE994-14|Diptera|Cecidomyiidae||BOLD:ACN0994
Cecidomyiidae|[5146]|GMAGC014-15|Diptera|Cecidomyiidae||BOLD:ACV7854
Cecidomyiidae|[5147]|GMARB1553-14|Diptera|Cecidomyiidae||BOLD:ACM4810
Cecidomyiidae|[5148]|GMARD1175-14|Diptera|Cecidomyiidae||BOLD:ACM9532
Cecidomyiidae|[5149]|GMAGM915-15|Diptera|Cecidomyiidae||BOLD:ACN2810
Cecidomyiidae|[5150]|GMARN1321-14|Diptera|Cecidomyiidae||BOLD:ACO0467
Cecidomyiidae|[5151]|GMAGE1997-15|Diptera|Cecidomyiidae||BOLD:ACW7075
Cecidomyiidae|[5152]|GMARB1177-14|Diptera|Cecidomyiidae||BOLD:ACM7178
Cecidomyiidae|[5153]|GMAGF407-15|Diptera|Cecidomyiidae||BOLD:ACW3570
Cecidomyiidae|[5154]|GMAGR367-15|Diptera|Cecidomyiidae||BOLD:ACX6697
Cecidomyiidae|[5155]|GMARB550-14|Diptera|Cecidomyiidae||BOLD:ACM6535
Cecidomyiidae|[5156]|GMAGD2103-15|Diptera|Cecidomyiidae||BOLD:ACM7242
Cecidomyiidae|[5157]|GMART1561-14|Diptera|Cecidomyiidae||BOLD:ACO0098
Cecidomyiidae|[5158]|GMAGL2710-15|Diptera|Cecidomyiidae||BOLD:ACW8719
Cecidomyiidae|[5159]|GMART1448-14|Diptera|Cecidomyiidae||BOLD:ACO0234
Cecidomyiidae|[5160]|GMAGA598-15|Diptera|Cecidomyiidae||BOLD:ACM5229
Cecidomyiidae|[5161]|GMAGA587-15|Diptera|Cecidomyiidae||BOLD:ACM5585
Cecidomyiidae|[5162]|GMAGB1417-15|Diptera|Cecidomyiidae||BOLD:ACM9029
Cecidomyiidae|[5163]|GMAGA438-15|Diptera|Cecidomyiidae||BOLD:ACM5830
Cecidomyiidae|[5164]|GMARB917-14|Diptera|Cecidomyiidae||BOLD:ACM8699
Cecidomyiidae|[5165]|GMARC091-14|Diptera|Cecidomyiidae||BOLD:ACM8698
Cecidomyiidae|[5166]|GMAGN2336-15|Diptera|Cecidomyiidae||BOLD:ACX1856
Cecidomyiidae|[5167]|GMAGB1447-15|Diptera|Cecidomyiidae||BOLD:ACW5945
Cecidomyiidae|[5168]|GMARB709-14|Diptera|Cecidomyiidae||BOLD:ACM5839
Cecidomyiidae|[5169]|GMARA073-14|Diptera|Cecidomyiidae||BOLD:ACM5817
Cecidomyiidae|[5170]|GMAGA864-15|Diptera|Cecidomyiidae||BOLD:ACM6302
Cecidomyiidae|[5171]|GMARB1608-14|Diptera|Cecidomyiidae||BOLD:ACM6238
Cecidomyiidae|[5172]|GMAGI579-15|Diptera|Cecidomyiidae||BOLD:ACY8480
Cecidomyiidae|[5173]|GMARA1207-14|Diptera|Cecidomyiidae||BOLD:ACM5764
Cecidomyiidae|[5174]|GMAGB721-15|Diptera|Cecidomyiidae||BOLD:ACM8316
Cecidomyiidae|[5175]|GMAGK1155-15|Diptera|Cecidomyiidae||BOLD:ACW9578
Cecidomyiidae|[5176]|GMAGM1641-15|Diptera|Cecidomyiidae||BOLD:ACX3986
Cecidomyiidae|[5177]|GMARF1008-14|Diptera|Cecidomyiidae||BOLD:ACN0753
[illegible]

Cecidomyiidae|[5175]|GMAGK1155-15|Diptera|Cecidomyiidae||BOLD:ACW9578
Cecidomyiidae|[5176]|GMAGM1641-15|Diptera|Cecidomyiidae||BOLD:ACX3986
Cecidomyiidae|[5177]|GMARF1008-14|Diptera|Cecidomyiidae||BOLD:ACN0753
Cecidomyiidae|[5178]|GMAGA412-15|Diptera|Cecidomyiidae||BOLD:ACM5049
Cecidomyiidae|[5179]|GMAGA498-15|Diptera|Cecidomyiidae||BOLD:ACM4713
Cecidomyiidae|[5180]|GMARF1035-14|Diptera|Cecidomyiidae||BOLD:ACN0733
Ceratopogonidae|[5181]|GMAGA416-15|Diptera|Ceratopogonidae||BOLD:ACM5023
Cecidomyiidae|[5182]|GMAGI429-15|Diptera|Cecidomyiidae||BOLD:ACW4616
Cecidomyiidae|[5183]|GMAGL1927-15|Diptera|Cecidomyiidae||BOLD:ACN0387
Cecidomyiidae|[5184]|GMAGV205-15|Diptera|Cecidomyiidae||BOLD:ACX2625
Cecidomyiidae|[5185]|GMARG1408-14|Diptera|Cecidomyiidae||BOLD:ACN0176
Cecidomyiidae|[5186]|GMAGL083-15|Diptera|Cecidomyiidae||BOLD:ACY8457
Cecidomyiidae|[5187]|GMARD920-14|Diptera|Cecidomyiidae||BOLD:ACM9042
Cecidomyiidae|[5188]|GMARA1505-14|Diptera|Cecidomyiidae||BOLD:ACM8105
Cecidomyiidae|[5189]|GMARA1189-14|Diptera|Cecidomyiidae||BOLD:ACM5034
Cecidomyiidae|[5190]|GMAGB204-15|Diptera|Cecidomyiidae||BOLD:ACM6176
Cecidomyiidae|[5191]|GMARB1198-14|Diptera|Cecidomyiidae||BOLD:ACM5624
Cecidomyiidae|[5192]|GMARI1836-14|Diptera|Cecidomyiidae||BOLD:ACN0396
Cecidomyiidae|[5193]|GMAGE125-15|Diptera|Cecidomyiidae||BOLD:ACW3138
Cecidomyiidae|[5194]|GMAGA550-15|Diptera|Cecidomyiidae||BOLD:ACV2616
Cecidomyiidae|[5195]|GMARP412-14|Diptera|Cecidomyiidae||BOLD:ACO2277
Cecidomyiidae|[5196]|GMARD954-14|Diptera|Cecidomyiidae||BOLD:ACM9124
Cecidomyiidae|[5197]|GMARE796-14|Diptera|Cecidomyiidae||BOLD:ACM8223
Cecidomyiidae|[5198]|GMAGG988-15|Diptera|Cecidomyiidae||BOLD:ACN0384
Cecidomyiidae|[5199]|GMAGJ265-15|Diptera|Cecidomyiidae||BOLD:ACW9451
Cecidomyiidae|[5200]|GMAGE1709-15|Diptera|Cecidomyiidae||BOLD:ADB8460
Cecidomyiidae|[5201]|GMARK1442-14|Diptera|Cecidomyiidae||BOLD:ACN2427
Cecidomyiidae|[5202]|GMAGC778-15|Diptera|Cecidomyiidae||BOLD:ACW2017
Cecidomyiidae|[5203]|GMAGB675-15|Diptera|Cecidomyiidae||BOLD:ACW2837
Cecidomyiidae|[5204]|GMARA310-14|Diptera|Cecidomyiidae||BOLD:ACM9757
Cecidomyiidae|[5205]|GMART1044-14|Diptera|Cecidomyiidae||BOLD:ACX8137
Cecidomyiidae|[5206]|GMAGB1479-15|Diptera|Cecidomyiidae||BOLD:ACW3675
Cecidomyiidae|[5207]|GMAGC446-15|Diptera|Cecidomyiidae||BOLD:ACW1789
Cecidomyiidae|[5208]|GMAGC250-15|Diptera|Cecidomyiidae||BOLD:ACW2077
Cecidomyiidae|[5209]|GMAGE2559-15|Diptera|Cecidomyiidae||BOLD:ACW7539
Cecidomyiidae|[5210]|GMAGB424-15|Diptera|Cecidomyiidae||BOLD:ACW6383
Cecidomyiidae|[5211]|GMARH177-14|Diptera|Cecidomyiidae||BOLD:ACN1590
Cecidomyiidae|[5212]|GMAGB1461-15|Diptera|Cecidomyiidae||BOLD:ACW3934
Cecidomyiidae|[5213]|GMARB1021-14|Diptera|Cecidomyiidae||BOLD:ACM6395
Cecidomyiidae|[5214]|GMARD407-14|Diptera|Cecidomyiidae||BOLD:ACN1410
Cecidomyiidae|[5215]|GMAGE2627-15|Diptera|Cecidomyiidae||BOLD:ACW8001
Cecidomyiidae|[5216]|GMAGS336-15|Diptera|Cecidomyiidae||BOLD:ACX5495
Cecidomyiidae|[5217]|GMAGW265-15|Diptera|Cecidomyiidae||BOLD:ACO1000
Cecidomyiidae|[5218]|GMARA056-14|Diptera|Cecidomyiidae||BOLD:ACM9611
Cecidomyiidae|[5219]|GMAGE950-15|Diptera|Cecidomyiidae||BOLD:ACW5939
Cecidomyiidae|[5220]|GMARA243-14|Diptera|Cecidomyiidae||BOLD:ACM9417
Cecidomyiidae|[5221]|GMARA124-14|Diptera|Cecidomyiidae||BOLD:ACM6053
Cecidomyiidae|[5222]|GMAGH550-15|Diptera|Cecidomyiidae||BOLD:ACN0278
Cecidomyiidae|[5223]|GMAGQ864-15|Diptera|Cecidomyiidae||BOLD:ACN8992
Cecidomyiidae|[5224]|GMART374-14|Diptera|Cecidomyiidae||BOLD:ACN9844
Cecidomyiidae|[5225]|GMARA1518-14|Diptera|Cecidomyiidae||BOLD:ACM6517
Cecidomyiidae|[5226]|GMAGE215-15|Diptera|Cecidomyiidae||BOLD:ACW4246
Cecidomyiidae|[5227]|GMAGB294-15|Diptera|Cecidomyiidae||BOLD:ACM6266
Cecidomyiidae|[5228]|GMAGH280-15|Diptera|Cecidomyiidae||BOLD:ACN0233
Cecidomyiidae|[5229]|GMARP615-14|Diptera|Cecidomyiidae||BOLD:ACN9438
Cecidomyiidae|[5230]|GMARR403-14|Diptera|Cecidomyiidae||BOLD:ACN7860
Cecidomyiidae|[5231]|GMARD780-14|Diptera|Cecidomyiidae||BOLD:ACM9384
Cecidomyiidae|[5232]|GMAGA639-15|Diptera|Cecidomyiidae||BOLD:ACV2765
Cecidomyiidae|[5233]|GMARD923-14|Diptera|Cecidomyiidae||BOLD:ACM8669
Cecidomyiidae|[5234]|GMAGU266-15|Diptera|Cecidomyiidae||BOLD:ACN7638
Cecidomyiidae|[5235]|GMAGT420-15|Diptera|Cecidomyiidae||BOLD:ACX5317
Cecidomyiidae|[5236]|GMAGV351-15|Diptera|Cecidomyiidae||BOLD:ACX2036
Cecidomyiidae|[5237]|GMAGN1314-15|Diptera|Cecidomyiidae||BOLD:ACX5645
Cecidomyiidae|[5238]|GMARR299-14|Diptera|Cecidomyiidae||BOLD:ACN8573
Cecidomyiidae|[5239]|GMAGB649-15|Diptera|Cecidomyiidae||BOLD:ACO1278
Cecidomyiidae|[5240]|GMAGC1429-15|Diptera|Cecidomyiidae||BOLD:ACW5280
Cecidomyiidae|[5241]|GMARR593-14|Diptera|Cecidomyiidae||BOLD:ACN7689
Cecidomyiidae|[5242]|GMAGD1057-15|Diptera|Cecidomyiidae||BOLD:ACW3514
Cecidomyiidae|[5243]|GMAGQ1763-15|Diptera|Cecidomyiidae||BOLD:ACX4609
Cecidomyiidae|[5244]|GMART093-14|Diptera|Cecidomyiidae||BOLD:ACO0258
Cecidomyiidae|[5245]|GMAGE231-15|Diptera|Cecidomyiidae||BOLD:ACW4472
Cecidomyiidae|[5246]|GMAGT582-15|Diptera|Cecidomyiidae||BOLD:ACX5582
Cecidomyiidae|[5247]|GMARR286-14|Diptera|Cecidomyiidae||BOLD:ACN8881
Cecidomyiidae|[5248]|GMARN1022-14|Diptera|Cecidomyiidae||BOLD:ACN9556
Cecidomyiidae|[5249]|GMAGV468-15|Diptera|Cecidomyiidae||BOLD:ACO0462
Cecidomyiidae|[5250]|GMARQ235-14|Diptera|Cecidomyiidae||BOLD:ACX3379
Cecidomyiidae|[5251]|GMARB1745-14|Diptera|Cecidomyiidae||BOLD:ACM4983
Cecidomyiidae|[5252]|GMARA277-14|Diptera|Cecidomyiidae||BOLD:ACM9272
Cecidomyiidae|[5253]|GMAGD1177-15|Diptera|Cecidomyiidae||BOLD:ACW4461
Cecidomyiidae|[5254]|GMARQ305-14|Diptera|Cecidomyiidae||BOLD:ACN8880
Cecidomyiidae|[5255]|GMAGE1931-15|Diptera|Cecidomyiidae||BOLD:ACW6876
Cecidomyiidae|[5256]|GMAGD965-15|Diptera|Cecidomyiidae||BOLD:ACW3733
Cecidomyiidae|[5257]|GMAGH1282-15|Diptera|Cecidomyiidae||BOLD:ACW7419
Cecidomyiidae|[5258]|GMAGD945-15|Diptera|Cecidomyiidae||BOLD:ACX4023
Cecidomyiidae|[5259]|GMARC487-14|Diptera|Cecidomyiidae||BOLD:ACM7734
Cecidomyiidae|[5260]|GMAGT780-15|Diptera|Cecidomyiidae||BOLD:ACX4503
Cecidomyiidae|[5261]|GMAGD1317-15|Diptera|Cecidomyiidae||BOLD:ACW4596
Cecidomyiidae|[5262]|GMARR525-14|Diptera|Cecidomyiidae||BOLD:ACN7783
Cecidomyiidae|[5263]|GMAGC762-15|Diptera|Cecidomyiidae||BOLD:ACW2752
Cecidomyiidae|[5264]|GMARA1537-14|Diptera|Cecidomyiidae||BOLD:ACM4924
Cecidomyiidae|[5265]|GMAGH1298-15|Diptera|Cecidomyiidae||BOLD:ACN8834
Cecidomyiidae|[5266]|GMARM595-14|Diptera|Cecidomyiidae||BOLD:ACX1359
Cecidomyiidae|[5267]|GMARA065-14|Diptera|Cecidomyiidae||BOLD:ACM8910
Cecidomyiidae|[5268]|GMARR669-14|Diptera|Cecidomyiidae||BOLD:ACK6521
Cecidomyiidae|[5269]|GMAGV246-15|Diptera|Cecidomyiidae||BOLD:ACX1355
Cecidomyiidae|[5270]|GMARB1543-14|Diptera|Cecidomyiidae||BOLD:ACM4811
Cecidomyiidae|[5271]|GMAGB764-15|Diptera|Cecidomyiidae||BOLD:ACM5344
Cecidomyiidae|[5272]|GMARD221-14|Diptera|Cecidomyiidae||BOLD:ACN1166
Cecidomyiidae|[5273]|GMAGD1103-15|Diptera|Cecidomyiidae||BOLD:ACW4032
Cecidomyiidae|[5274]|GMAGE828-15|Diptera|Cecidomyiidae||BOLD:ACW4852
Cecidomyiidae|[5275]|GMAGD613-15|Diptera|Cecidomyiidae||BOLD:ACW3015
Cecidomyiidae|[5276]|GMAGD562-15|Diptera|Cecidomyiidae||BOLD:ACO1059
Cecidomyiidae|[5277]|GMARP215-14|Diptera|Cecidomyiidae||BOLD:ACN9360

Cecidomyiidae|[5275]|GMAGD613-15|Diptera|Cecidomyiidae||BOLD:ACW3015
Cecidomyiidae|[5276]|GMAGD562-15|Diptera|Cecidomyiidae||BOLD:ACO1059
Cecidomyiidae|[5277]|GMARP215-14|Diptera|Cecidomyiidae||BOLD:ACN9360
Cecidomyiidae|[5278]|GMAGT086-15|Diptera|Cecidomyiidae||BOLD:ACX5692
Cecidomyiidae|[5279]|GMAGD183-15|Diptera|Cecidomyiidae||BOLD:ACW5002
Cecidomyiidae|[5280]|GMARR539-14|Diptera|Cecidomyiidae||BOLD:ACN8626
Cecidomyiidae|[5281]|GMARM370-14|Diptera|Cecidomyiidae||BOLD:ACN2892
Cecidomyiidae|[5282]|GMARR396-14|Diptera|Cecidomyiidae||BOLD:ACN8041
Cecidomyiidae|[5283]|GMAGE2160-15|Diptera|Cecidomyiidae||BOLD:ACX4885
Cecidomyiidae|[5284]|GMARR505-14|Diptera|Cecidomyiidae||BOLD:ACN8727
Cecidomyiidae|[5285]|GMARA232-14|Diptera|Cecidomyiidae||BOLD:ACM5581
Cecidomyiidae|[5286]|GMARU463-14|Diptera|Cecidomyiidae||BOLD:ACO1287
Cecidomyiidae|[5287]|GMAGH1162-15|Diptera|Cecidomyiidae||BOLD:ACN9165
Cecidomyiidae|[5288]|GMARA1175-14|Diptera|Cecidomyiidae||BOLD:ACM9673
Cecidomyiidae|[5289]|GMARQ519-14|Diptera|Cecidomyiidae||BOLD:ACN7657
Cecidomyiidae|[5290]|GMARA1171-14|Diptera|Cecidomyiidae||BOLD:ACM9258
Cecidomyiidae|[5291]|GMARB798-14|Diptera|Cecidomyiidae||BOLD:ACM6044
Cecidomyiidae|[5292]|GMARA058-14|Diptera|Cecidomyiidae||BOLD:ACM8996
Cecidomyiidae|[5293]|GMARA023-14|Diptera|Cecidomyiidae||BOLD:ACM8610
Cecidomyiidae|[5294]|GMAGB559-15|Diptera|Cecidomyiidae||BOLD:ACW4850
Cecidomyiidae|[5295]|GMARF951-14|Diptera|Cecidomyiidae||BOLD:ACM9535
Cecidomyiidae|[5296]|GMAGG1284-15|Diptera|Cecidomyiidae||BOLD:ACW2954
Cecidomyiidae|[5297]|GMARS202-14|Diptera|Cecidomyiidae||BOLD:ACN7888
Cecidomyiidae|[5298]|GMARA025-14|Diptera|Cecidomyiidae||BOLD:ACM9096
Cecidomyiidae|[5299]|GMAGG1437-15|Diptera|Cecidomyiidae||BOLD:ACW4682
Cecidomyiidae|[5300]|GMAGN1520-15|Diptera|Cecidomyiidae||BOLD:ACX1453
Cecidomyiidae|[5301]|GMAGA418-15|Diptera|Cecidomyiidae||BOLD:ACV1940
Cecidomyiidae|[5302]|GMARP132-14|Diptera|Cecidomyiidae||BOLD:ACN7720
Cecidomyiidae|[5303]|GMAGB339-15|Diptera|Cecidomyiidae||BOLD:ACN7498
Cecidomyiidae|[5304]|GMAGC165-15|Diptera|Cecidomyiidae||BOLD:ACN8533
Cecidomyiidae|[5305]|GMAGD661-15|Diptera|Cecidomyiidae||BOLD:ACN8556
Cecidomyiidae|[5306]|GMAGU400-15|Diptera|Cecidomyiidae||BOLD:ACX4963
Cecidomyiidae|[5307]|GMARR176-14|Diptera|Cecidomyiidae||BOLD:ACN7505
Cecidomyiidae|[5308]|GMAGH278-15|Diptera|Cecidomyiidae||BOLD:ACW1580
Cecidomyiidae|[5309]|GMAGP1010-15|Diptera|Cecidomyiidae||BOLD:ACX4159
Cecidomyiidae|[5310]|GMARA229-14|Diptera|Cecidomyiidae||BOLD:ACM9149
Cecidomyiidae|[5311]|GMAGS632-15|Diptera|Cecidomyiidae||BOLD:ACX5535
Cecidomyiidae|[5312]|GMARR1083-14|Diptera|Cecidomyiidae||BOLD:ACN8491
Cecidomyiidae|[5313]|GMARR774-14|Diptera|Cecidomyiidae||BOLD:ACN8276
Cecidomyiidae|[5314]|GMAGS757-15|Diptera|Cecidomyiidae||BOLD:ACX4992
Cecidomyiidae|[5315]|GMAGB301-15|Diptera|Cecidomyiidae||BOLD:ACW1592
Cecidomyiidae|[5316]|GMAGD1293-15|Diptera|Cecidomyiidae||BOLD:ACW3584
Cecidomyiidae|[5317]|GMARR587-14|Diptera|Cecidomyiidae||BOLD:ACN8774
Cecidomyiidae|[5318]|GMAGE2141-15|Diptera|Cecidomyiidae||BOLD:ACW7606
Cecidomyiidae|[5319]|GMARD162-14|Diptera|Cecidomyiidae||BOLD:ACM9972
Cecidomyiidae|[5320]|GMARA1435-14|Diptera|Cecidomyiidae||BOLD:ACM8369
Cecidomyiidae|[5321]|GMARF811-14|Diptera|Cecidomyiidae||BOLD:ACM9671
Cecidomyiidae|[5322]|GMAGU573-15|Diptera|Cecidomyiidae||BOLD:ACX5138
Cecidomyiidae|[5323]|GMAGU251-15|Diptera|Cecidomyiidae||BOLD:ACX5231
Cecidomyiidae|[5324]|GMARS400-14|Diptera|Cecidomyiidae||BOLD:ACN8510
Cecidomyiidae|[5325]|GMAGH158-15|Diptera|Cecidomyiidae||BOLD:ACW3541
Cecidomyiidae|[5326]|GMARS375-14|Diptera|Cecidomyiidae||BOLD:ACN8734
Cecidomyiidae|[5327]|GMARS412-14|Diptera|Cecidomyiidae||BOLD:ACN8193
Cecidomyiidae|[5328]|GMAGB412-15|Diptera|Cecidomyiidae||BOLD:ACM4929
Cecidomyiidae|[5329]|GMARA1141-14|Diptera|Cecidomyiidae||BOLD:ACV3455
Cecidomyiidae|[5330]|GMAGD1095-15|Diptera|Cecidomyiidae||BOLD:ACN7738
Cecidomyiidae|[5331]|GMAGA659-15|Diptera|Cecidomyiidae||BOLD:ACW4185
Cecidomyiidae|[5332]|GMARB760-14|Diptera|Cecidomyiidae||BOLD:ACM6610
Cecidomyiidae|[5333]|GMARA089-14|Diptera|Cecidomyiidae||BOLD:ACM4934
Cecidomyiidae|[5334]|GMARK698-14|Diptera|Cecidomyiidae||BOLD:ACN5204
Cecidomyiidae|[5335]|GMAGA515-15|Diptera|Cecidomyiidae||BOLD:ACM5180
Cecidomyiidae|[5336]|GMARA1497-14|Diptera|Cecidomyiidae||BOLD:ACM4842
Cecidomyiidae|[5337]|GMAGS483-15|Diptera|Cecidomyiidae||BOLD:ACO0690
Cecidomyiidae|[5338]|GMAGD834-15|Diptera|Cecidomyiidae||BOLD:ACM7502
Cecidomyiidae|[5339]|GMARF512-14|Diptera|Cecidomyiidae||BOLD:ACM8679
Cecidomyiidae|[5340]|GMARH1354-14|Diptera|Cecidomyiidae||BOLD:ACN1591
Cecidomyiidae|[5341]|GMART538-14|Diptera|Cecidomyiidae||BOLD:ACO0095
Cecidomyiidae|[5342]|GMARC199-14|Diptera|Cecidomyiidae||BOLD:ACM7906
Cecidomyiidae|[5343]|GMAGE176-15|Diptera|Cecidomyiidae||BOLD:ACW3994
Cecidomyiidae|[5344]|GMAGB1378-15|Diptera|Cecidomyiidae||BOLD:ACW3518
Cecidomyiidae|[5345]|GMAGG1718-15|Diptera|Cecidomyiidae||BOLD:ACN0414
Cecidomyiidae|[5346]|GMARA1483-14|Diptera|Cecidomyiidae||BOLD:ACM4937
Cecidomyiidae|[5347]|GMAGA429-15|Diptera|Cecidomyiidae||BOLD:ACV1967
Cecidomyiidae|[5348]|GMAGG1942-15|Diptera|Cecidomyiidae||BOLD:ACW6755
Cecidomyiidae|[5349]|GMARA1786-14|Diptera|Cecidomyiidae||BOLD:ACM6305
Cecidomyiidae|[5350]|GMARA1341-14|Diptera|Cecidomyiidae||BOLD:ACM8406
Cecidomyiidae|[5351]|GMARG1344-14|Diptera|Cecidomyiidae||BOLD:ACN1460
Cecidomyiidae|[5352]|GMARP802-14|Diptera|Cecidomyiidae||BOLD:ACO3730
Cecidomyiidae|[5353]|GMARF029-14|Diptera|Cecidomyiidae||BOLD:ACN1361
Cecidomyiidae|[5354]|GMAGS106-15|Diptera|Cecidomyiidae||BOLD:ACN8244
Cecidomyiidae|[5355]|GMAGT106-15|Diptera|Cecidomyiidae||BOLD:ACX6190
Cecidomyiidae|[5356]|GMARA069-14|Diptera|Cecidomyiidae||BOLD:ACM6265
Cecidomyiidae|[5357]|GMARA1134-14|Diptera|Cecidomyiidae||BOLD:ACM9513
Cecidomyiidae|[5358]|GMARD1164-14|Diptera|Cecidomyiidae||BOLD:ACM9305
Cecidomyiidae|[5359]|GMARD1125-14|Diptera|Cecidomyiidae||BOLD:ACM8868
Cecidomyiidae|[5360]|GMARB740-14|Diptera|Cecidomyiidae||BOLD:ACM5748
Cecidomyiidae|[5361]|GMAGD558-15|Diptera|Cecidomyiidae||BOLD:ACN0885
Cecidomyiidae|[5362]|GMART448-14|Diptera|Cecidomyiidae||BOLD:ACN9891
Cecidomyiidae|[5363]|GMAGB1508-15|Diptera|Cecidomyiidae||BOLD:ACN0290
Cecidomyiidae|[5364]|GMARA1334-14|Diptera|Cecidomyiidae||BOLD:ACM8430
Cecidomyiidae|[5365]|GMAGE2106-15|Diptera|Cecidomyiidae||BOLD:ACW6421
Cecidomyiidae|[5366]|GMAGH146-15|Diptera|Cecidomyiidae||BOLD:ACW3197
Cecidomyiidae|[5367]|GMART1017-14|Diptera|Cecidomyiidae||BOLD:ACN9985
Cecidomyiidae|[5368]|GMAGE1712-15|Diptera|Cecidomyiidae||BOLD:ACW2795
Cecidomyiidae|[5369]|GMAGG1926-15|Diptera|Cecidomyiidae||BOLD:ACN9582
Cecidomyiidae|[5370]|GMAGD645-15|Diptera|Cecidomyiidae||BOLD:ACW4348
Cecidomyiidae|[5371]|GMAGO360-15|Diptera|Cecidomyiidae||BOLD:ACX5704
Cecidomyiidae|[5372]|GMAGQ390-15|Diptera|Cecidomyiidae||BOLD:ACX7205
Cecidomyiidae|[5373]|GMARD348-14|Diptera|Cecidomyiidae||BOLD:ACN0846
Cecidomyiidae|[5374]|GMARD132-14|Diptera|Cecidomyiidae||BOLD:ACN1536
Cecidomyiidae|[5375]|GMAGN1911-15|Diptera|Cecidomyiidae||BOLD:ACN1100
Cecidomyiidae|[5376]|GMART882-14|Diptera|Cecidomyiidae||BOLD:ACN9947

Cecidomyiidae|[5374]|GMARD152-14|Diptera|Cecidomyiidae||BOLD:ACN1536
Cecidomyiidae|[5375]|GMAGN1911-15|Diptera|Cecidomyiidae||BOLD:ACN1100
Cecidomyiidae|[5376]|GMART882-14|Diptera|Cecidomyiidae||BOLD:ACN9947
Cecidomyiidae|[5377]|GMAGD2251-15|Diptera|Cecidomyiidae||BOLD:ACW6972
Cecidomyiidae|[5378]|GMARL1365-14|Diptera|Cecidomyiidae||BOLD:ACN2022
Cecidomyiidae|[5379]|GMARD561-14|Diptera|Cecidomyiidae||BOLD:ACN0072
Cecidomyiidae|[5380]|GMAGE2101-15|Diptera|Cecidomyiidae||BOLD:ACM5606
Cecidomyiidae|[5381]|GMAGD1165-15|Diptera|Cecidomyiidae||BOLD:ACW4719
Cecidomyiidae|[5382]|GMARC515-14|Diptera|Cecidomyiidae||BOLD:ACM4734
Cecidomyiidae|[5383]|GMAGX591-15|Diptera|Cecidomyiidae||BOLD:ACX5162
Cecidomyiidae|[5384]|GMAGB532-15|Diptera|Cecidomyiidae||BOLD:ACN9140
Cecidomyiidae|[5385]|GMARA1109-14|Diptera|Cecidomyiidae||BOLD:ACM8722
Cecidomyiidae|[5386]|GMART509-14|Diptera|Cecidomyiidae||BOLD:ACO0309
Cecidomyiidae|[5387]|GMART370-14|Diptera|Cecidomyiidae||BOLD:ACN9313
Cecidomyiidae|[5388]|GMARD659-14|Diptera|Cecidomyiidae||BOLD:ACN1535
Cecidomyiidae|[5389]|GMARA1200-14|Diptera|Cecidomyiidae||BOLD:ACM9102
Cecidomyiidae|[5390]|GMAGD2038-15|Diptera|Cecidomyiidae||BOLD:ACW2219
Cecidomyiidae|[5391]|GMAGH1183-15|Diptera|Cecidomyiidae||BOLD:ACW3626
Cecidomyiidae|[5392]|GMARA1469-14|Diptera|Cecidomyiidae||BOLD:ACM5723
Cecidomyiidae|[5393]|GMARR1106-14|Diptera|Cecidomyiidae||BOLD:ACN7480
Cecidomyiidae|[5394]|GMAGB818-15|Diptera|Cecidomyiidae||BOLD:ACM5993
Cecidomyiidae|[5395]|GMAGD2190-15|Diptera|Cecidomyiidae||BOLD:ACW2479
Cecidomyiidae|[5396]|GMAGB1366-15|Diptera|Cecidomyiidae||BOLD:ACW4028
Cecidomyiidae|[5397]|GMAGG1535-15|Diptera|Cecidomyiidae||BOLD:ACN9616
Cecidomyiidae|[5398]|GMARK1401-14|Diptera|Cecidomyiidae||BOLD:ACN2821
Cecidomyiidae|[5399]|GMAGL525-15|Diptera|Cecidomyiidae||BOLD:ACX6078
Cecidomyiidae|[5400]|GMARA343-14|Diptera|Cecidomyiidae||BOLD:ACN8490
Cecidomyiidae|[5401]|GMARN991-14|Diptera|Cecidomyiidae||BOLD:ACN4918
Cecidomyiidae|[5402]|GMARB1041-14|Diptera|Cecidomyiidae||BOLD:ACM5565
Cecidomyiidae|[5403]|GMARM473-14|Diptera|Cecidomyiidae||BOLD:ACN2958
Cecidomyiidae|[5404]|GMARB1438-14|Diptera|Cecidomyiidae||BOLD:ACM6245
Cecidomyiidae|[5405]|GMART1193-14|Diptera|Cecidomyiidae||BOLD:ACO1247
Cecidomyiidae|[5406]|GMAGG2059-15|Diptera|Cecidomyiidae||BOLD:ACO3865
Cecidomyiidae|[5407]|GMARR487-14|Diptera|Cecidomyiidae||BOLD:ACN7632
Cecidomyiidae|[5408]|GMAGD2209-15|Diptera|Cecidomyiidae||BOLD:ACN8809
Cecidomyiidae|[5409]|GMARB717-14|Diptera|Cecidomyiidae||BOLD:ACM4762
Cecidomyiidae|[5410]|GMAGV537-15|Diptera|Cecidomyiidae||BOLD:ACX1560
Cecidomyiidae|[5411]|GMAGD653-15|Diptera|Cecidomyiidae||BOLD:ACN1833
Cecidomyiidae|[5412]|GMAGP1848-15|Diptera|Cecidomyiidae||BOLD:ACX5964
Cecidomyiidae|[5413]|GMAGT375-15|Diptera|Cecidomyiidae||BOLD:ACX5456
Cecidomyiidae|[5414]|GMART217-14|Diptera|Cecidomyiidae||BOLD:ACN9965
Cecidomyiidae|[5415]|GMART1432-14|Diptera|Cecidomyiidae||BOLD:ACN9262
Cecidomyiidae|[5416]|GMARA075-14|Diptera|Cecidomyiidae||BOLD:ACM8830
Cecidomyiidae|[5417]|GMAGB1541-15|Diptera|Cecidomyiidae||BOLD:ACM5213
Cecidomyiidae|[5418]|GMAGB557-15|Diptera|Cecidomyiidae||BOLD:ACN0180
Cecidomyiidae|[5419]|GMARC501-14|Diptera|Cecidomyiidae||BOLD:ACM7565
Cecidomyiidae|[5420]|GMARS198-14|Diptera|Cecidomyiidae||BOLD:ACN8334
Cecidomyiidae|[5421]|GMARA304-14|Diptera|Cecidomyiidae||BOLD:ACM5689
Cecidomyiidae|[5422]|GMAGK800-15|Diptera|Cecidomyiidae||BOLD:ACX6080
Cecidomyiidae|[5423]|GMAGT808-15|Diptera|Cecidomyiidae||BOLD:ACX4316
Cecidomyiidae|[5424]|GMAGV542-15|Diptera|Cecidomyiidae||BOLD:ACX1729
Cecidomyiidae|[5425]|GMARB1440-14|Diptera|Cecidomyiidae||BOLD:ACM5577
Cecidomyiidae|[5426]|GMAGE1097-15|Diptera|Cecidomyiidae||BOLD:ACW1394
Cecidomyiidae|[5427]|GMARH1180-14|Diptera|Cecidomyiidae||BOLD:ACN0283
Cecidomyiidae|[5428]|GMAGE2143-15|Diptera|Cecidomyiidae||BOLD:ACW8039
Cecidomyiidae|[5429]|GMARE097-14|Diptera|Cecidomyiidae||BOLD:ACN1622
Cecidomyiidae|[5430]|GMARA159-14|Diptera|Cecidomyiidae||BOLD:ACM8480
Cecidomyiidae|[5431]|GMAGD1036-15|Diptera|Cecidomyiidae||BOLD:ACN8349
Cecidomyiidae|[5432]|GMARD912-14|Diptera|Cecidomyiidae||BOLD:ACM8643
Cecidomyiidae|[5433]|GMARP561-14|Diptera|Cecidomyiidae||BOLD:ACN9724
Cecidomyiidae|[5434]|GMART542-14|Diptera|Cecidomyiidae||BOLD:ACO0056
Cecidomyiidae|[5435]|GMAGA559-15|Diptera|Cecidomyiidae||BOLD:ACN7023
Cecidomyiidae|[5436]|GMAGC813-15|Diptera|Cecidomyiidae||BOLD:ACN8243
Cecidomyiidae|[5437]|GMAGD429-15|Diptera|Cecidomyiidae||BOLD:ACN7411
Cecidomyiidae|[5438]|GMARI1238-14|Diptera|Cecidomyiidae||BOLD:ACN0450
Cecidomyiidae|[5439]|GMART729-14|Diptera|Cecidomyiidae||BOLD:ACO0276
Cecidomyiidae|[5440]|GMAGH755-15|Diptera|Cecidomyiidae||BOLD:ACW3193
Cecidomyiidae|[5441]|GMAGJ1682-15|Diptera|Cecidomyiidae||BOLD:ACW8538
Cecidomyiidae|[5442]|GMARA1779-14|Diptera|Cecidomyiidae||BOLD:ACM5266
Cecidomyiidae|[5443]|GMAGD1037-15|Diptera|Cecidomyiidae||BOLD:ACW3915
Cecidomyiidae|[5444]|GMAGH491-15|Diptera|Cecidomyiidae||BOLD:ACW2442
Cecidomyiidae|[5445]|GMAGD681-15|Diptera|Cecidomyiidae||BOLD:ACN9294
Cecidomyiidae|[5446]|GMARR392-14|Diptera|Cecidomyiidae||BOLD:ACN7760
Cecidomyiidae|[5447]|GMAGC204-15|Diptera|Cecidomyiidae||BOLD:ACX2592
Cecidomyiidae|[5448]|GMARD539-14|Diptera|Cecidomyiidae||BOLD:ACN1625
Cecidomyiidae|[5449]|GMARR357-14|Diptera|Cecidomyiidae||BOLD:ACN7678
Cecidomyiidae|[5450]|GMAGK1331-15|Diptera|Cecidomyiidae||BOLD:ACO1025
Cecidomyiidae|[5451]|GMARR637-14|Diptera|Cecidomyiidae||BOLD:ACN7667
Cecidomyiidae|[5452]|GMARK2092-14|Diptera|Cecidomyiidae||BOLD:ACN2661
Cecidomyiidae|[5453]|GMAGG2343-15|Diptera|Cecidomyiidae||BOLD:ACW7124
Cecidomyiidae|[5454]|GMARG928-14|Diptera|Cecidomyiidae||BOLD:ACN0876
Cecidomyiidae|[5455]|GMARB1046-14|Diptera|Cecidomyiidae||BOLD:ACM6376
Cecidomyiidae|[5456]|GMAGG1980-15|Diptera|Cecidomyiidae||BOLD:ACO0144
Cecidomyiidae|[5457]|GMARD771-14|Diptera|Cecidomyiidae||BOLD:ACX2510
Cecidomyiidae|[5458]|GMAGB1521-15|Diptera|Cecidomyiidae||BOLD:ACM5279
Cecidomyiidae|[5459]|GMAGD1217-15|Diptera|Cecidomyiidae||BOLD:ACM8851
Cecidomyiidae|[5460]|GMARI797-14|Diptera|Cecidomyiidae||BOLD:ACN1613
Cecidomyiidae|[5461]|GMARJ1320-14|Diptera|Cecidomyiidae||BOLD:ACN1121
Cecidomyiidae|[5462]|GMAGD1327-15|Diptera|Cecidomyiidae||BOLD:ACW3818
Cecidomyiidae|[5463]|GMARS278-14|Diptera|Cecidomyiidae||BOLD:ACN7455
Cecidomyiidae|[5464]|GMAGL2578-15|Diptera|Cecidomyiidae||BOLD:ACN0124
Cecidomyiidae|[5465]|GMARA1166-14|Diptera|Cecidomyiidae||BOLD:ACM9335
Cecidomyiidae|[5466]|GMARI887-14|Diptera|Cecidomyiidae||BOLD:ACN1827
Cecidomyiidae|[5467]|GMAGM2202-15|Diptera|Cecidomyiidae||BOLD:ACX3649
Cecidomyiidae|[5468]|GMARF162-14|Diptera|Cecidomyiidae||BOLD:ACM9367
Cecidomyiidae|[5469]|GMAGD2347-15|Diptera|Cecidomyiidae||BOLD:ACM8539
Cecidomyiidae|[5470]|GMARI1801-14|Diptera|Cecidomyiidae||BOLD:ACN0830
Cecidomyiidae|[5471]|GMAGT697-15|Diptera|Cecidomyiidae||BOLD:ACX4901
Cecidomyiidae|[5472]|GMAGF377-15|Diptera|Cecidomyiidae||BOLD:ACN7746
Cecidomyiidae|[5473]|GMAGM1900-15|Diptera|Cecidomyiidae||BOLD:ACX1893
Cecidomyiidae|[5474]|GMARF1119-14|Diptera|Cecidomyiidae||BOLD:ACN0458
Cecidomyiidae|[5475]|GMAGL1607-15|Diptera|Cecidomyiidae||BOLD:ACX4013
Cecidomyiidae|[5476]|GMAGW027-15|Diptera|Cecidomyiidae||BOLD:ACX3072

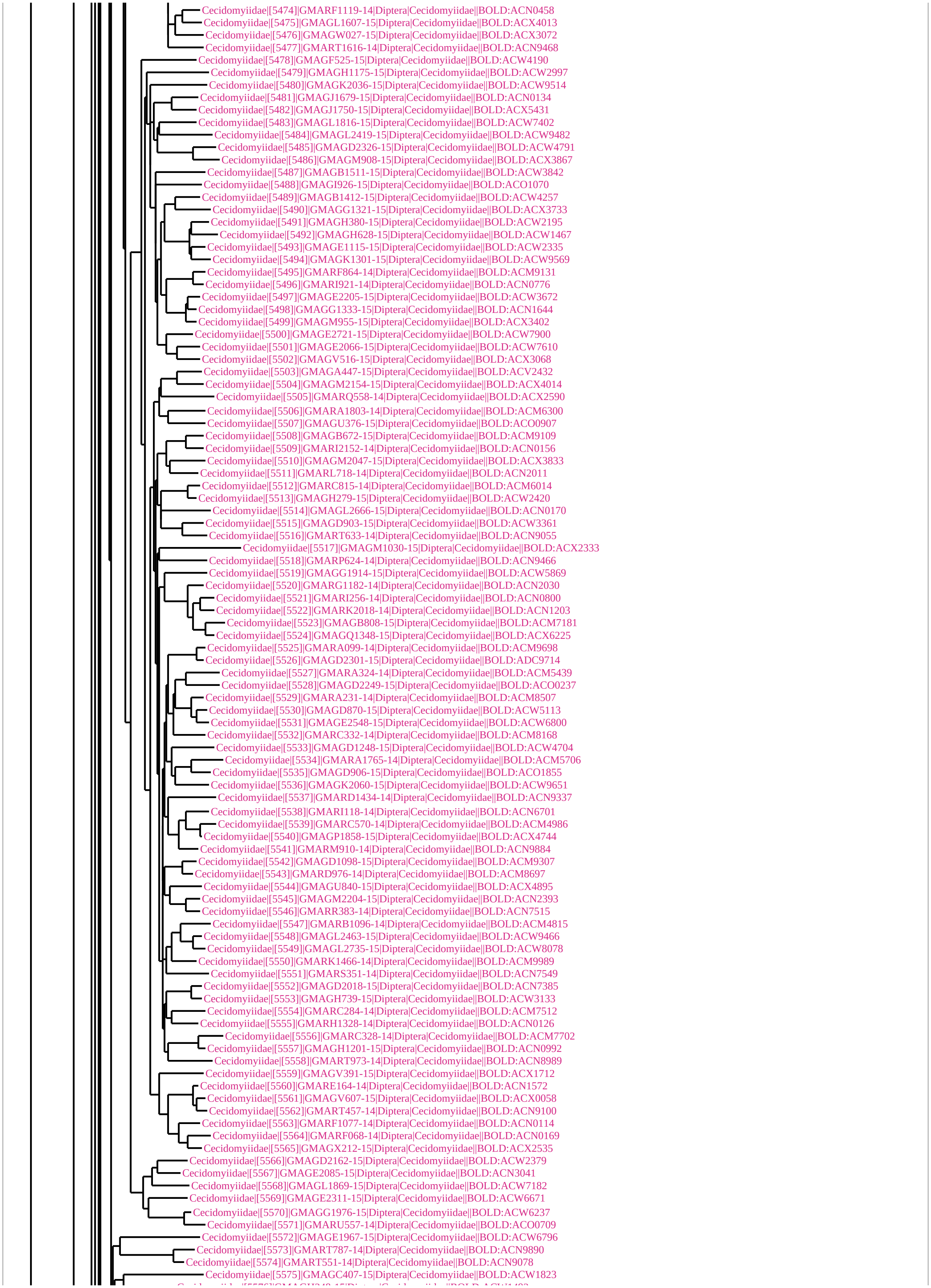
Cecidomyiidae|[5474]|GMARF1119-14|Diptera|Cecidomyiidae||BOLD:ACN0458
Cecidomyiidae|[5475]|GMAGL1607-15|Diptera|Cecidomyiidae||BOLD:ACX4013
Cecidomyiidae|[5476]|GMAGW027-15|Diptera|Cecidomyiidae||BOLD:ACX3072
Cecidomyiidae|[5477]|GMART1616-14|Diptera|Cecidomyiidae||BOLD:ACN9468
Cecidomyiidae|[5478]|GMAGF525-15|Diptera|Cecidomyiidae||BOLD:ACW4190
Cecidomyiidae|[5479]|GMAGH1175-15|Diptera|Cecidomyiidae||BOLD:ACW2997
Cecidomyiidae|[5480]|GMAGK2036-15|Diptera|Cecidomyiidae||BOLD:ACW9514
Cecidomyiidae|[5481]|GMAGJ1679-15|Diptera|Cecidomyiidae||BOLD:ACN0134
Cecidomyiidae|[5482]|GMAGJ1750-15|Diptera|Cecidomyiidae||BOLD:ACX5431
Cecidomyiidae|[5483]|GMAGL1816-15|Diptera|Cecidomyiidae||BOLD:ACW7402
Cecidomyiidae|[5484]|GMAGL2419-15|Diptera|Cecidomyiidae||BOLD:ACW9482
Cecidomyiidae|[5485]|GMAGD2326-15|Diptera|Cecidomyiidae||BOLD:ACW4791
Cecidomyiidae|[5486]|GMAGM908-15|Diptera|Cecidomyiidae||BOLD:ACX3867
Cecidomyiidae|[5487]|GMAGB1511-15|Diptera|Cecidomyiidae||BOLD:ACW3842
Cecidomyiidae|[5488]|GMAGI926-15|Diptera|Cecidomyiidae||BOLD:ACO1070
Cecidomyiidae|[5489]|GMAGB1412-15|Diptera|Cecidomyiidae||BOLD:ACW4257
Cecidomyiidae|[5490]|GMAGG1321-15|Diptera|Cecidomyiidae||BOLD:ACX3733
Cecidomyiidae|[5491]|GMAGH380-15|Diptera|Cecidomyiidae||BOLD:ACW2195
Cecidomyiidae|[5492]|GMAGH628-15|Diptera|Cecidomyiidae||BOLD:ACW1467
Cecidomyiidae|[5493]|GMAGE1115-15|Diptera|Cecidomyiidae||BOLD:ACW2335
Cecidomyiidae|[5494]|GMAGK1301-15|Diptera|Cecidomyiidae||BOLD:ACW9569
Cecidomyiidae|[5495]|GMARF864-14|Diptera|Cecidomyiidae||BOLD:ACM9131
Cecidomyiidae|[5496]|GMARI921-14|Diptera|Cecidomyiidae||BOLD:ACN0776
Cecidomyiidae|[5497]|GMAGE2205-15|Diptera|Cecidomyiidae||BOLD:ACW3672
Cecidomyiidae|[5498]|GMAGG1333-15|Diptera|Cecidomyiidae||BOLD:ACN1644
Cecidomyiidae|[5499]|GMAGM955-15|Diptera|Cecidomyiidae||BOLD:ACX3402
Cecidomyiidae|[5500]|GMAGE2721-15|Diptera|Cecidomyiidae||BOLD:ACW7900
Cecidomyiidae|[5501]|GMAGE2066-15|Diptera|Cecidomyiidae||BOLD:ACW7610
Cecidomyiidae|[5502]|GMAGV516-15|Diptera|Cecidomyiidae||BOLD:ACX3068
Cecidomyiidae|[5503]|GMAGA447-15|Diptera|Cecidomyiidae||BOLD:ACV2432
Cecidomyiidae|[5504]|GMAGM2154-15|Diptera|Cecidomyiidae||BOLD:ACX4014
Cecidomyiidae|[5505]|GMARQ558-14|Diptera|Cecidomyiidae||BOLD:ACX2590
Cecidomyiidae|[5506]|GMARA1803-14|Diptera|Cecidomyiidae||BOLD:ACM6300
Cecidomyiidae|[5507]|GMAGU376-15|Diptera|Cecidomyiidae||BOLD:ACO0907
Cecidomyiidae|[5508]|GMAGB672-15|Diptera|Cecidomyiidae||BOLD:ACM9109
Cecidomyiidae|[5509]|GMARI2152-14|Diptera|Cecidomyiidae||BOLD:ACN0156
Cecidomyiidae|[5510]|GMAGM2047-15|Diptera|Cecidomyiidae||BOLD:ACX3833
Cecidomyiidae|[5511]|GMARL718-14|Diptera|Cecidomyiidae||BOLD:ACN2011
Cecidomyiidae|[5512]|GMARC815-14|Diptera|Cecidomyiidae||BOLD:ACM6014
Cecidomyiidae|[5513]|GMAGH279-15|Diptera|Cecidomyiidae||BOLD:ACW2420
Cecidomyiidae|[5514]|GMAGL2666-15|Diptera|Cecidomyiidae||BOLD:ACN0170
Cecidomyiidae|[5515]|GMAGD903-15|Diptera|Cecidomyiidae||BOLD:ACW3361
Cecidomyiidae|[5516]|GMART633-14|Diptera|Cecidomyiidae||BOLD:ACN9055
Cecidomyiidae|[5517]|GMAGM1030-15|Diptera|Cecidomyiidae||BOLD:ACX2333
Cecidomyiidae|[5518]|GMARP624-14|Diptera|Cecidomyiidae||BOLD:ACN9466
Cecidomyiidae|[5519]|GMAGG1914-15|Diptera|Cecidomyiidae||BOLD:ACW5869
Cecidomyiidae|[5520]|GMARG1182-14|Diptera|Cecidomyiidae||BOLD:ACN2030
Cecidomyiidae|[5521]|GMARI256-14|Diptera|Cecidomyiidae||BOLD:ACN0800
Cecidomyiidae|[5522]|GMARK2018-14|Diptera|Cecidomyiidae||BOLD:ACN1203
Cecidomyiidae|[5523]|GMAGB808-15|Diptera|Cecidomyiidae||BOLD:ACM7181
Cecidomyiidae|[5524]|GMAGQ1348-15|Diptera|Cecidomyiidae||BOLD:ACX6225
Cecidomyiidae|[5525]|GMARA099-14|Diptera|Cecidomyiidae||BOLD:ACM9698
Cecidomyiidae|[5526]|GMAGD2301-15|Diptera|Cecidomyiidae||BOLD:ADC9714
Cecidomyiidae|[5527]|GMARA324-14|Diptera|Cecidomyiidae||BOLD:ACM5439
Cecidomyiidae|[5528]|GMAGD2249-15|Diptera|Cecidomyiidae||BOLD:ACO0237
Cecidomyiidae|[5529]|GMARA231-14|Diptera|Cecidomyiidae||BOLD:ACM8507
Cecidomyiidae|[5530]|GMAGD870-15|Diptera|Cecidomyiidae||BOLD:ACW5113
Cecidomyiidae|[5531]|GMAGE2548-15|Diptera|Cecidomyiidae||BOLD:ACW6800
Cecidomyiidae|[5532]|GMARC332-14|Diptera|Cecidomyiidae||BOLD:ACM8168
Cecidomyiidae|[5533]|GMAGD1248-15|Diptera|Cecidomyiidae||BOLD:ACW4704
Cecidomyiidae|[5534]|GMARA1765-14|Diptera|Cecidomyiidae||BOLD:ACM5706
Cecidomyiidae|[5535]|GMAGD906-15|Diptera|Cecidomyiidae||BOLD:ACO1855
Cecidomyiidae|[5536]|GMAGK2060-15|Diptera|Cecidomyiidae||BOLD:ACW9651
Cecidomyiidae|[5537]|GMARD1434-14|Diptera|Cecidomyiidae||BOLD:ACN9337
Cecidomyiidae|[5538]|GMARI118-14|Diptera|Cecidomyiidae||BOLD:ACN6701
Cecidomyiidae|[5539]|GMARC570-14|Diptera|Cecidomyiidae||BOLD:ACM4986
Cecidomyiidae|[5540]|GMAGP1858-15|Diptera|Cecidomyiidae||BOLD:ACX4744
Cecidomyiidae|[5541]|GMARM910-14|Diptera|Cecidomyiidae||BOLD:ACN9884
Cecidomyiidae|[5542]|GMAGD1098-15|Diptera|Cecidomyiidae||BOLD:ACM9307
Cecidomyiidae|[5543]|GMARD976-14|Diptera|Cecidomyiidae||BOLD:ACM8697
Cecidomyiidae|[5544]|GMAGU840-15|Diptera|Cecidomyiidae||BOLD:ACX4895
Cecidomyiidae|[5545]|GMAGM2204-15|Diptera|Cecidomyiidae||BOLD:ACN2393
Cecidomyiidae|[5546]|GMARR383-14|Diptera|Cecidomyiidae||BOLD:ACN7515
Cecidomyiidae|[5547]|GMARB1096-14|Diptera|Cecidomyiidae||BOLD:ACM4815
Cecidomyiidae|[5548]|GMAGL2463-15|Diptera|Cecidomyiidae||BOLD:ACW9466
Cecidomyiidae|[5549]|GMAGL2735-15|Diptera|Cecidomyiidae||BOLD:ACW8078
Cecidomyiidae|[5550]|GMARK1466-14|Diptera|Cecidomyiidae||BOLD:ACM9989
Cecidomyiidae|[5551]|GMARS351-14|Diptera|Cecidomyiidae||BOLD:ACN7549
Cecidomyiidae|[5552]|GMAGD2018-15|Diptera|Cecidomyiidae||BOLD:ACN7385
Cecidomyiidae|[5553]|GMAGH739-15|Diptera|Cecidomyiidae||BOLD:ACW3133
Cecidomyiidae|[5554]|GMARC284-14|Diptera|Cecidomyiidae||BOLD:ACM7512
Cecidomyiidae|[5555]|GMARH1328-14|Diptera|Cecidomyiidae||BOLD:ACN0126
Cecidomyiidae|[5556]|GMARC328-14|Diptera|Cecidomyiidae||BOLD:ACM7702
Cecidomyiidae|[5557]|GMAGH1201-15|Diptera|Cecidomyiidae||BOLD:ACN0992
Cecidomyiidae|[5558]|GMART973-14|Diptera|Cecidomyiidae||BOLD:ACN8989
Cecidomyiidae|[5559]|GMAGV391-15|Diptera|Cecidomyiidae||BOLD:ACX1712
Cecidomyiidae|[5560]|GMARE164-14|Diptera|Cecidomyiidae||BOLD:ACN1572
Cecidomyiidae|[5561]|GMAGV607-15|Diptera|Cecidomyiidae||BOLD:ACX0058
Cecidomyiidae|[5562]|GMART457-14|Diptera|Cecidomyiidae||BOLD:ACN9100
Cecidomyiidae|[5563]|GMARF1077-14|Diptera|Cecidomyiidae||BOLD:ACN0114
Cecidomyiidae|[5564]|GMARF068-14|Diptera|Cecidomyiidae||BOLD:ACN0169
Cecidomyiidae|[5565]|GMAGX212-15|Diptera|Cecidomyiidae||BOLD:ACX2535
Cecidomyiidae|[5566]|GMAGD2162-15|Diptera|Cecidomyiidae||BOLD:ACW2379
Cecidomyiidae|[5567]|GMAGE2085-15|Diptera|Cecidomyiidae||BOLD:ACN3041
Cecidomyiidae|[5568]|GMAGL1869-15|Diptera|Cecidomyiidae||BOLD:ACW7182
Cecidomyiidae|[5569]|GMAGE2311-15|Diptera|Cecidomyiidae||BOLD:ACW6671
Cecidomyiidae|[5570]|GMAGG1976-15|Diptera|Cecidomyiidae||BOLD:ACW6237
Cecidomyiidae|[5571]|GMARU557-14|Diptera|Cecidomyiidae||BOLD:ACO0709
Cecidomyiidae|[5572]|GMAGE1967-15|Diptera|Cecidomyiidae||BOLD:ACW6796
Cecidomyiidae|[5573]|GMART787-14|Diptera|Cecidomyiidae||BOLD:ACN9890
Cecidomyiidae|[5574]|GMART551-14|Diptera|Cecidomyiidae||BOLD:ACN9078
Cecidomyiidae|[5575]|GMAGC407-15|Diptera|Cecidomyiidae||BOLD:ACW1823

Cecidomyiidae|[5573]|GMART787-14|Diptera|Cecidomyiidae||BOLD:ACN9890
Cecidomyiidae|[5574]|GMART551-14|Diptera|Cecidomyiidae||BOLD:ACN9078
Cecidomyiidae|[5575]|GMAGC407-15|Diptera|Cecidomyiidae||BOLD:ACW1823
Cecidomyiidae|[5576]|GMAGH248-15|Diptera|Cecidomyiidae||BOLD:ACW1492
Cecidomyiidae|[5577]|GMAGK484-15|Diptera|Cecidomyiidae||BOLD:ACX6068
Cecidomyiidae|[5578]|GMARA168-14|Diptera|Cecidomyiidae||BOLD:ACM5746
Cecidomyiidae|[5579]|GMAGD877-15|Diptera|Cecidomyiidae||BOLD:ACX4193
Cecidomyiidae|[5580]|GMAGA532-15|Diptera|Cecidomyiidae||BOLD:ACM4987
Cecidomyiidae|[5581]|GMAGK1243-15|Diptera|Cecidomyiidae||BOLD:ACW9152
Cecidomyiidae|[5582]|GMARF398-14|Diptera|Cecidomyiidae||BOLD:ACM8758
Cecidomyiidae|[5583]|GMARL487-14|Diptera|Cecidomyiidae||BOLD:ACN6347
Cecidomyiidae|[5584]|GMAGK1827-15|Diptera|Cecidomyiidae||BOLD:ACX4256
Cecidomyiidae|[5585]|GMARL154-14|Diptera|Cecidomyiidae||BOLD:ACN1973
Cecidomyiidae|[5586]|GMARN1223-14|Diptera|Cecidomyiidae||BOLD:ACN9569
Cecidomyiidae|[5587]|GMAGI795-15|Diptera|Cecidomyiidae||BOLD:ACN2300
Cecidomyiidae|[5588]|GMARC631-14|Diptera|Cecidomyiidae||BOLD:ACW6743
Cecidomyiidae|[5589]|GMAGC079-15|Diptera|Cecidomyiidae||BOLD:ACY8916
Cecidomyiidae|[5590]|GMARK1587-14|Diptera|Cecidomyiidae||BOLD:ACN0867
Cecidomyiidae|[5591]|GMAGI668-15|Diptera|Cecidomyiidae||BOLD:ACW6063
Cecidomyiidae|[5592]|GMARP655-14|Diptera|Cecidomyiidae||BOLD:ACO0285
Cecidomyiidae|[5593]|GMAGB217-15|Diptera|Cecidomyiidae||BOLD:ACW2259
Cecidomyiidae|[5594]|GMAGQ1014-15|Diptera|Cecidomyiidae||BOLD:ACX4356
Cecidomyiidae|[5595]|GMARB1241-14|Diptera|Cecidomyiidae||BOLD:ACM5440
Cecidomyiidae|[5596]|GMAGA574-15|Diptera|Cecidomyiidae||BOLD:ACM5300
Cecidomyiidae|[5597]|GMARB528-14|Diptera|Cecidomyiidae||BOLD:ACM6539
Cecidomyiidae|[5598]|GMARK1465-14|Diptera|Cecidomyiidae||BOLD:ACN1026
Cecidomyiidae|[5599]|GMARI1255-14|Diptera|Cecidomyiidae||BOLD:ACN0247
Cecidomyiidae|[5600]|GMAGB330-15|Diptera|Cecidomyiidae||BOLD:ACM4723
Cecidomyiidae|[5601]|GMAGE966-15|Diptera|Cecidomyiidae||BOLD:ACM9462
Cecidomyiidae|[5602]|GMAGN1977-15|Diptera|Cecidomyiidae||BOLD:ACN5491
Cecidomyiidae|[5603]|GMARI325-14|Diptera|Cecidomyiidae||BOLD:ACM9873
Cecidomyiidae|[5604]|GMARB1020-14|Diptera|Cecidomyiidae||BOLD:ACM6394
Cecidomyiidae|[5605]|GMAGN1854-15|Diptera|Cecidomyiidae||BOLD:ACX2006
Cecidomyiidae|[5606]|GMAGB379-15|Diptera|Cecidomyiidae||BOLD:ACN0220
Cecidomyiidae|[5607]|GMAGH410-15|Diptera|Cecidomyiidae||BOLD:ACN1651
Cecidomyiidae|[5608]|GMAGE1207-15|Diptera|Cecidomyiidae||BOLD:ACM7348
Cecidomyiidae|[5609]|GMARC428-14|Diptera|Cecidomyiidae||BOLD:ACM7438
Cecidomyiidae|[5610]|GMARF599-14|Diptera|Cecidomyiidae||BOLD:ACM9461
Cecidomyiidae|[5611]|GMAGC054-15|Diptera|Cecidomyiidae||BOLD:ACM8391
Cecidomyiidae|[5612]|GMAGF309-15|Diptera|Cecidomyiidae||BOLD:ACN0047
Cecidomyiidae|[5613]|GMAGB260-15|Diptera|Cecidomyiidae||BOLD:ACM7440
Cecidomyiidae|[5614]|GMARC144-14|Diptera|Cecidomyiidae||BOLD:ACM7583
Cecidomyiidae|[5615]|GMARG345-14|Diptera|Cecidomyiidae||BOLD:ACN0821
Cecidomyiidae|[5616]|GMAGM835-15|Diptera|Cecidomyiidae||BOLD:ACX3557
Cecidomyiidae|[5617]|GMAGN1611-15|Diptera|Cecidomyiidae||BOLD:ACX4743
Cecidomyiidae|[5618]|GMARS225-14|Diptera|Cecidomyiidae||BOLD:ACN8922
Cecidomyiidae|[5619]|GMART320-14|Diptera|Cecidomyiidae||BOLD:ACO0302
Cecidomyiidae|[5620]|GMAGH699-15|Diptera|Cecidomyiidae||BOLD:ACN5117
Cecidomyiidae|[5621]|GMARK1905-14|Diptera|Cecidomyiidae||BOLD:ACO0839
Cecidomyiidae|[5622]|GMARR783-14|Diptera|Cecidomyiidae||BOLD:ACN7917
Cecidomyiidae|[5623]|GMARB562-14|Diptera|Cecidomyiidae||BOLD:ACM6322
Cecidomyiidae|[5624]|GMAGD2267-15|Diptera|Cecidomyiidae||BOLD:ACW3939
Cecidomyiidae|[5625]|GMAGJ1086-15|Diptera|Cecidomyiidae||BOLD:ACW8159
Cecidomyiidae|[5626]|GMAGA870-15|Diptera|Cecidomyiidae||BOLD:ACW2055
Cecidomyiidae|[5627]|GMAGA428-15|Diptera|Cecidomyiidae||BOLD:ACO1065
Cecidomyiidae|[5628]|GMARB1449-14|Diptera|Cecidomyiidae||BOLD:ACM5579
Cecidomyiidae|[5629]|GMARE175-14|Diptera|Cecidomyiidae||BOLD:ACN1740
Cecidomyiidae|[5630]|GMAGN1984-15|Diptera|Cecidomyiidae||BOLD:ACN7604
Cecidomyiidae|[5631]|GMAGC273-15|Diptera|Cecidomyiidae||BOLD:ACM6270
Cecidomyiidae|[5632]|GMAGC188-15|Diptera|Cecidomyiidae||BOLD:ACM7397
Cecidomyiidae|[5633]|GMARK1058-14|Diptera|Cecidomyiidae||BOLD:ACW7264
Cecidomyiidae|[5634]|GMAGC133-15|Diptera|Cecidomyiidae||BOLD:ACM8760
Cecidomyiidae|[5635]|GMARB633-14|Diptera|Cecidomyiidae||BOLD:ACM6163
Cecidomyiidae|[5636]|GMARB1077-14|Diptera|Cecidomyiidae||BOLD:ACM6580
Cecidomyiidae|[5637]|GMAGO409-15|Diptera|Cecidomyiidae||BOLD:ACN3327
Cecidomyiidae|[5638]|GMARL735-14|Diptera|Cecidomyiidae||BOLD:ACN2851
Cecidomyiidae|[5639]|GMAGE2702-15|Diptera|Cecidomyiidae||BOLD:ACW7458
Cecidomyiidae|[5640]|GMARF463-14|Diptera|Cecidomyiidae||BOLD:ACM9319
Cecidomyiidae|[5641]|GMAGU598-15|Diptera|Cecidomyiidae||BOLD:ACX4710
Cecidomyiidae|[5642]|GMAGV429-15|Diptera|Cecidomyiidae||BOLD:ACX3291
Cecidomyiidae|[5643]|GMART1433-14|Diptera|Cecidomyiidae||BOLD:ACO0009
Cecidomyiidae|[5644]|GMARD1196-14|Diptera|Cecidomyiidae||BOLD:ACM9309
Cecidomyiidae|[5645]|GMAGF546-15|Diptera|Cecidomyiidae||BOLD:ACW3287
Cecidomyiidae|[5646]|GMARI283-14|Diptera|Cecidomyiidae||BOLD:ACN0799
Cecidomyiidae|[5647]|GMAGC237-15|Diptera|Cecidomyiidae||BOLD:ACN1015
Cecidomyiidae|[5648]|GMAGH714-15|Diptera|Cecidomyiidae||BOLD:ACW3516
Cecidomyiidae|[5649]|GMAGP1876-15|Diptera|Cecidomyiidae||BOLD:ACX5152
Cecidomyiidae|[5650]|GMARK498-14|Diptera|Cecidomyiidae||BOLD:ACN0323
Cecidomyiidae|[5651]|GMAGO559-15|Diptera|Cecidomyiidae||BOLD:ACX4340
Cecidomyiidae|[5652]|GMAGP1505-15|Diptera|Cecidomyiidae||BOLD:ACX4231
Cecidomyiidae|[5653]|GMARP069-14|Diptera|Cecidomyiidae||BOLD:ACO3877
Cecidomyiidae|[5654]|GMARA1097-14|Diptera|Cecidomyiidae||BOLD:ACM8906
Cecidomyiidae|[5655]|GMAGN1612-15|Diptera|Cecidomyiidae||BOLD:ACO3048
Cecidomyiidae|[5656]|GMARE245-14|Diptera|Cecidomyiidae||BOLD:ACX3639
Cecidomyiidae|[5657]|GMAGT513-15|Diptera|Cecidomyiidae||BOLD:ACO0388
Cecidomyiidae|[5658]|GMAGA862-15|Diptera|Cecidomyiidae||BOLD:ACM4998
Cecidomyiidae|[5659]|GMAGA497-15|Diptera|Cecidomyiidae||BOLD:ACV2919
Cecidomyiidae|[5660]|GMAGG1315-15|Diptera|Cecidomyiidae||BOLD:ACW4187
Cecidomyiidae|[5661]|GMARA1219-14|Diptera|Cecidomyiidae||BOLD:ACX3550
Cecidomyiidae|[5662]|GMARF934-14|Diptera|Cecidomyiidae||BOLD:ACX1007
Cecidomyiidae|[5663]|GMAGH1249-15|Diptera|Cecidomyiidae||BOLD:ACN1725
Cecidomyiidae|[5664]|GMAGJ1767-15|Diptera|Cecidomyiidae||BOLD:ACN1912
Cecidomyiidae|[5665]|GMAGG2094-15|Diptera|Cecidomyiidae||BOLD:ACW7046
Cecidomyiidae|[5666]|GMARK1180-14|Diptera|Cecidomyiidae||BOLD:ACN1804
Cecidomyiidae|[5667]|GMAGV329-15|Diptera|Cecidomyiidae||BOLD:ACX2613
Cecidomyiidae|[5668]|GMARD1438-14|Diptera|Cecidomyiidae||BOLD:ACM8870
Cecidomyiidae|[5669]|GMAGB656-15|Diptera|Cecidomyiidae||BOLD:ACW3242
Cecidomyiidae|[5670]|GMAGI1240-15|Diptera|Cecidomyiidae||BOLD:ACN0467
Cecidomyiidae|[5671]|GMARF313-14|Diptera|Cecidomyiidae||BOLD:ACM9644
Cecidomyiidae|[5672]|GMARN1204-14|Diptera|Cecidomyiidae||BOLD:ACN9051
Cecidomyiidae|[5673]|GMAGG2256-15|Diptera|Cecidomyiidae||BOLD:ACW6559
Cecidomyiidae|[5674]|GMAGJ1098-15|Diptera|Cecidomyiidae||BOLD:ACW9280
Cecidomyiidae|[5675]|GMAGC834-15|Diptera|Cecidomyiidae||BOLD:ACW2386

Cecidomyiidae|[5673]|GMAGG2256-15|Diptera|Cecidomyiidae||BOLD:ACW6559
Cecidomyiidae|[5674]|GMAGJ1098-15|Diptera|Cecidomyiidae||BOLD:ACW9280
Cecidomyiidae|[5675]|GMAGC834-15|Diptera|Cecidomyiidae||BOLD:ACW2386
Cecidomyiidae|[5676]|GMARS342-14|Diptera|Cecidomyiidae||BOLD:ACN7420
Cecidomyiidae|[5677]|GMART557-14|Diptera|Cecidomyiidae||BOLD:ACN9681
Cecidomyiidae|[5678]|GMART248-14|Diptera|Cecidomyiidae||BOLD:ACN9892
Cecidomyiidae|[5679]|GMARA1385-14|Diptera|Cecidomyiidae||BOLD:ACM9021
Cecidomyiidae|[5680]|GMARB1356-14|Diptera|Cecidomyiidae||BOLD:ACM5535
Cecidomyiidae|[5681]|GMARA1420-14|Diptera|Cecidomyiidae||BOLD:ACM9057
Cecidomyiidae|[5682]|GMARE843-14|Diptera|Cecidomyiidae||BOLD:ACN1000
Cecidomyiidae|[5683]|GMARB702-14|Diptera|Cecidomyiidae||BOLD:ACM5994
Cecidomyiidae|[5684]|GMARD1091-14|Diptera|Cecidomyiidae||BOLD:ACM8499
Cecidomyiidae|[5685]|GMARO1283-14|Diptera|Cecidomyiidae||BOLD:ACO3586
Cecidomyiidae|[5686]|GMARA1241-14|Diptera|Cecidomyiidae||BOLD:ACM5908
Cecidomyiidae|[5687]|GMAGC063-15|Diptera|Cecidomyiidae||BOLD:ACV7561
Cecidomyiidae|[5688]|GMARA074-14|Diptera|Cecidomyiidae||BOLD:ACM8479
Cecidomyiidae|[5689]|GMARB1233-14|Diptera|Cecidomyiidae||BOLD:ACM5433
Cecidomyiidae|[5690]|GMAGT707-15|Diptera|Cecidomyiidae||BOLD:ACX4450
Cecidomyiidae|[5691]|GMARB698-14|Diptera|Cecidomyiidae||BOLD:ACM4754
Cecidomyiidae|[5692]|GMARA111-14|Diptera|Cecidomyiidae||BOLD:ACM8612
Cecidomyiidae|[5693]|GMARB1326-14|Diptera|Cecidomyiidae||BOLD:ACM6294
Cecidomyiidae|[5694]|GMAGB1458-15|Diptera|Cecidomyiidae||BOLD:ACN8304
Cecidomyiidae|[5695]|GMAGD690-15|Diptera|Cecidomyiidae||BOLD:ACW3631
Cecidomyiidae|[5696]|GMAGE1896-15|Diptera|Cecidomyiidae||BOLD:ACM8876
Cecidomyiidae|[5697]|GMAGW171-15|Diptera|Cecidomyiidae||BOLD:ACO0700
Cecidomyiidae|[5698]|GMART1437-14|Diptera|Cecidomyiidae||BOLD:ACO0170
Cecidomyiidae|[5699]|GMARA1361-14|Diptera|Cecidomyiidae||BOLD:ACM8986
Cecidomyiidae|[5700]|GMART379-14|Diptera|Cecidomyiidae||BOLD:ACN9199
Cecidomyiidae|[5701]|GMARA1491-14|Diptera|Cecidomyiidae||BOLD:ACM4848
Cecidomyiidae|[5702]|GMARA105-14|Diptera|Cecidomyiidae||BOLD:ACN7889
Cecidomyiidae|[5703]|GMARD613-14|Diptera|Cecidomyiidae||BOLD:ACM9974
Cecidomyiidae|[5704]|GMAGE637-15|Diptera|Cecidomyiidae||BOLD:ACW4126
Cecidomyiidae|[5705]|GMAGF373-15|Diptera|Cecidomyiidae||BOLD:ACW3446
Cecidomyiidae|[5706]|GMAGB1035-15|Diptera|Cecidomyiidae||BOLD:ACN1374
Cecidomyiidae|[5707]|GMARM379-14|Diptera|Cecidomyiidae||BOLD:ACN2366
Cecidomyiidae|[5708]|GMART1071-14|Diptera|Cecidomyiidae||BOLD:ACN9888
Cecidomyiidae|[5709]|GMAGQ1299-15|Diptera|Cecidomyiidae||BOLD:ACX5980
Cecidomyiidae|[5710]|GMARD493-14|Diptera|Cecidomyiidae||BOLD:ACN1371
Cecidomyiidae|[5711]|GMAGD399-15|Diptera|Cecidomyiidae||BOLD:ACO1201
Cecidomyiidae|[5712]|GMAGR568-15|Diptera|Cecidomyiidae||BOLD:ACX5235
Cecidomyiidae|[5713]|GMARD937-14|Diptera|Cecidomyiidae||BOLD:ACM9298
Cecidomyiidae|[5714]|GMART884-14|Diptera|Cecidomyiidae||BOLD:ACN9931
Cecidomyiidae|[5715]|GMARB1618-14|Diptera|Cecidomyiidae||BOLD:ACW7529
Cecidomyiidae|[5716]|GMARH796-14|Diptera|Cecidomyiidae||BOLD:ACN1739
Cecidomyiidae|[5717]|GMARD2137-14|Diptera|Cecidomyiidae||BOLD:ACN1030
Cecidomyiidae|[5718]|GMAGJ1279-15|Diptera|Cecidomyiidae||BOLD:ACX4723
Cecidomyiidae|[5719]|GMARS332-14|Diptera|Cecidomyiidae||BOLD:ACN7954
Cecidomyiidae|[5720]|GMARA1460-14|Diptera|Cecidomyiidae||BOLD:ACM6043
Cecidomyiidae|[5721]|GMAGB812-15|Diptera|Cecidomyiidae||BOLD:ACW7710
Cecidomyiidae|[5722]|GMARI1715-14|Diptera|Cecidomyiidae||BOLD:ACN0589
Cecidomyiidae|[5723]|GMARI125-14|Diptera|Cecidomyiidae||BOLD:ACN7236
Cecidomyiidae|[5724]|GMAGC402-15|Diptera|Cecidomyiidae||BOLD:ACW2534
Cecidomyiidae|[5725]|GMARB1110-14|Diptera|Cecidomyiidae||BOLD:ACM4862
Cecidomyiidae|[5726]|GMARB976-14|Diptera|Cecidomyiidae||BOLD:ACM5160
Cecidomyiidae|[5727]|GMARE384-14|Diptera|Cecidomyiidae||BOLD:ACN0097
Cecidomyiidae|[5728]|GMARS250-14|Diptera|Cecidomyiidae||BOLD:ACN8236
Cecidomyiidae|[5729]|GMARA1136-14|Diptera|Cecidomyiidae||BOLD:ACM9337
Cecidomyiidae|[5730]|GMAGD956-15|Diptera|Cecidomyiidae||BOLD:ACX4435
Cecidomyiidae|[5731]|GMARA226-14|Diptera|Cecidomyiidae||BOLD:ACM9801
Cecidomyiidae|[5732]|GMARA1125-14|Diptera|Cecidomyiidae||BOLD:ACM9429
Cecidomyiidae|[5733]|GMAGD2087-15|Diptera|Cecidomyiidae||BOLD:ACX3217
Cecidomyiidae|[5734]|GMARB1362-14|Diptera|Cecidomyiidae||BOLD:ACM5772
Cecidomyiidae|[5735]|GMAGC454-15|Diptera|Cecidomyiidae||BOLD:ACW1523
Cecidomyiidae|[5736]|GMAGG1361-15|Diptera|Cecidomyiidae||BOLD:ACW2839
Cecidomyiidae|[5737]|GMAGG1845-15|Diptera|Cecidomyiidae||BOLD:ACN9671
Cecidomyiidae|[5738]|GMARB1683-14|Diptera|Cecidomyiidae||BOLD:ACM4994
Cecidomyiidae|[5739]|GMAGK1676-15|Diptera|Cecidomyiidae||BOLD:ACX0057
Cecidomyiidae|[5740]|GMAGC426-15|Diptera|Cecidomyiidae||BOLD:ACN5630
Cecidomyiidae|[5741]|GMAGL1666-15|Diptera|Cecidomyiidae||BOLD:ACW7401
Cecidomyiidae|[5742]|GMARA1111-14|Diptera|Cecidomyiidae||BOLD:ACM5313
Cecidomyiidae|[5743]|GMARL798-14|Diptera|Cecidomyiidae||BOLD:ACN2408
Cecidomyiidae|[5744]|GMAGB1486-15|Diptera|Cecidomyiidae||BOLD:ACN0788
Cecidomyiidae|[5745]|GMARQ171-14|Diptera|Cecidomyiidae||BOLD:ACN7857
Cecidomyiidae|[5746]|GMAGC434-15|Diptera|Cecidomyiidae||BOLD:ACM6192
Cecidomyiidae|[5747]|GMAGG1816-15|Diptera|Cecidomyiidae||BOLD:ACN0519
Cecidomyiidae|[5748]|GMART506-14|Diptera|Cecidomyiidae||BOLD:ACN9501
Cecidomyiidae|[5749]|GMAGG1971-15|Diptera|Cecidomyiidae||BOLD:ACW3789
Cecidomyiidae|[5750]|GMARQ390-14|Diptera|Cecidomyiidae||BOLD:ACN7509
Cecidomyiidae|[5751]|GMAGV226-15|Diptera|Cecidomyiidae||BOLD:ACX2465
Cecidomyiidae|[5752]|GMAGA490-15|Diptera|Cecidomyiidae||BOLD:ACO0196
Cecidomyiidae|[5753]|GMAGK1660-15|Diptera|Cecidomyiidae||BOLD:ACO0161
Cecidomyiidae|[5754]|GMAGJ1840-15|Diptera|Cecidomyiidae||BOLD:ACX4521
Cecidomyiidae|[5755]|GMARI506-14|Diptera|Cecidomyiidae||BOLD:ACN0576
Cecidomyiidae|[5756]|GMARA342-14|Diptera|Cecidomyiidae||BOLD:ACM8371
Cecidomyiidae|[5757]|GMARH705-14|Diptera|Cecidomyiidae||BOLD:ACN0110
Cecidomyiidae|[5758]|GMARU408-14|Diptera|Cecidomyiidae||BOLD:ACO0719
Cecidomyiidae|[5759]|GMARA1362-14|Diptera|Cecidomyiidae||BOLD:ACM8948
Cecidomyiidae|[5760]|GMAGB475-15|Diptera|Cecidomyiidae||BOLD:ACW6524
Cecidomyiidae|[5761]|GMARB1098-14|Diptera|Cecidomyiidae||BOLD:ACM4816
Cecidomyiidae|[5762]|GMARC345-14|Diptera|Cecidomyiidae||BOLD:ACM7930
Cecidomyiidae|[5763]|GMAGR851-15|Diptera|Cecidomyiidae||BOLD:ACX4384
Cecidomyiidae|[5764]|GMARC261-14|Diptera|Cecidomyiidae||BOLD:ACM7290
Cecidomyiidae|[5765]|GMARL899-14|Diptera|Cecidomyiidae||BOLD:ACN1996
Cecidomyiidae|[5766]|GMAGK1328-15|Diptera|Cecidomyiidae||BOLD:ACX3749
Cecidomyiidae|[5767]|GMAGM992-15|Diptera|Cecidomyiidae||BOLD:ACN2783
Cecidomyiidae|[5768]|GMAGS1328-15|Diptera|Cecidomyiidae||BOLD:ACY4455
Cecidomyiidae|[5769]|GMARN433-14|Diptera|Cecidomyiidae||BOLD:ACN4915
Cecidomyiidae|[5770]|GMAGT340-15|Diptera|Cecidomyiidae||BOLD:ACX6226
Cecidomyiidae|[5771]|GMARB752-14|Diptera|Cecidomyiidae||BOLD:ACM5316
Cecidomyiidae|[5772]|GMAGI701-15|Diptera|Cecidomyiidae||BOLD:ACN8928
Cecidomyiidae|[5773]|GMAGF454-15|Diptera|Cecidomyiidae||BOLD:ACN8279
Cecidomyiidae|[5774]|GMARF1082-14|Diptera|Cecidomyiidae||BOLD:ACN0315
Cecidomyiidae|[5775]|GMAGT885-15|Diptera|Cecidomyiidae||BOLD:ACX5906

Cecidomyiidae|[5772]|GMAGI701-15|Diptera|Cecidomyiidae||BOLD:ACN[illegible]
Cecidomyiidae|[5773]|GMAGF454-15|Diptera|Cecidomyiidae||BOLD:ACN8279
Cecidomyiidae|[5774]|GMARF1082-14|Diptera|Cecidomyiidae||BOLD:ACN0315
Cecidomyiidae|[5775]|GMAGT885-15|Diptera|Cecidomyiidae||BOLD:ACX5906
Cecidomyiidae|[5776]|GMAGD1996-15|Diptera|Cecidomyiidae||BOLD:ACN0400
Cecidomyiidae|[5777]|GMARA115-14|Diptera|Cecidomyiidae||BOLD:ACM9012
Cecidomyiidae|[5778]|GMARB1352-14|Diptera|Cecidomyiidae||BOLD:ACM5771
Cecidomyiidae|[5779]|GMAGB850-15|Diptera|Cecidomyiidae||BOLD:ACM6371
Cecidomyiidae|[5780]|GMAGD1035-15|Diptera|Cecidomyiidae||BOLD:ACW2901
Cecidomyiidae|[5781]|GMAGC221-15|Diptera|Cecidomyiidae||BOLD:ACM6430
Cecidomyiidae|[5782]|GMARD2179-14|Diptera|Cecidomyiidae||BOLD:ACN1344
Cecidomyiidae|[5783]|GMAGK1775-15|Diptera|Cecidomyiidae||BOLD:ACW8074
Cecidomyiidae|[5784]|GMAGJ1311-15|Diptera|Cecidomyiidae||BOLD:ACN1140
Cecidomyiidae|[5785]|GMARA1078-14|Diptera|Cecidomyiidae||BOLD:ACM9411
Cecidomyiidae|[5786]|GMAGU188-15|Diptera|Cecidomyiidae||BOLD:ACX6067
Cecidomyiidae|[5787]|GMARB1327-14|Diptera|Cecidomyiidae||BOLD:ACM6400
Cecidomyiidae|[5788]|GMARA1309-14|Diptera|Cecidomyiidae||BOLD:ACM9077
Cecidomyiidae|[5789]|GMARB1227-14|Diptera|Cecidomyiidae||BOLD:ACM5386
Cecidomyiidae|[5790]|GMARB526-14|Diptera|Cecidomyiidae||BOLD:ACM6540
Cecidomyiidae|[5791]|GMAGE2556-15|Diptera|Cecidomyiidae||BOLD:ACW5979
Cecidomyiidae|[5792]|GMAGJ1773-15|Diptera|Cecidomyiidae||BOLD:ACN7935
Cecidomyiidae|[5793]|GMAGA536-15|Diptera|Cecidomyiidae||BOLD:ACN1658
Cecidomyiidae|[5794]|GMARB1216-14|Diptera|Cecidomyiidae||BOLD:ACM5625
Cecidomyiidae|[5795]|GMAGS862-15|Diptera|Cecidomyiidae||BOLD:ACX5053
Cecidomyiidae|[5796]|GMAGC822-15|Diptera|Cecidomyiidae||BOLD:ACO1213
Cecidomyiidae|[5797]|GMARB1048-14|Diptera|Cecidomyiidae||BOLD:ACM6617
Cecidomyiidae|[5798]|GMAGB1127-15|Diptera|Cecidomyiidae||BOLD:ACN1489
Cecidomyiidae|[5799]|GMARB1490-14|Diptera|Cecidomyiidae||BOLD:ACM5056
Cecidomyiidae|[5800]|GMAGD2008-15|Diptera|Cecidomyiidae||BOLD:ACM7233
Cecidomyiidae|[5801]|GMARA1796-14|Diptera|Cecidomyiidae||BOLD:ACM6306
Cecidomyiidae|[5802]|GMARB1636-14|Diptera|Cecidomyiidae||BOLD:ACM5801
Cecidomyiidae|[5803]|GMARD1155-14|Diptera|Cecidomyiidae||BOLD:ACM9167
Cecidomyiidae|[5804]|GMARJ1343-14|Diptera|Cecidomyiidae||BOLD:ACN1090
Cecidomyiidae|[5805]|GMAGD1283-15|Diptera|Cecidomyiidae||BOLD:ACW3126
Cecidomyiidae|[5806]|GMARD1495-14|Diptera|Cecidomyiidae||BOLD:ACN0977
Cecidomyiidae|[5807]|GMAGB1509-15|Diptera|Cecidomyiidae||BOLD:ACW1809
Cecidomyiidae|[5808]|GMAGB1467-15|Diptera|Cecidomyiidae||BOLD:ACW2050
Cecidomyiidae|[5809]|GMAGC223-15|Diptera|Cecidomyiidae||BOLD:ACN8137
Cecidomyiidae|[5810]|GMAGA865-15|Diptera|Cecidomyiidae||BOLD:ACM4897
Cecidomyiidae|[5811]|GMAGC445-15|Diptera|Cecidomyiidae||BOLD:ACM5594
Cecidomyiidae|[5812]|GMARA1782-14|Diptera|Cecidomyiidae||BOLD:ACM6038
Cecidomyiidae|[5813]|GMAGD2256-15|Diptera|Cecidomyiidae||BOLD:ACW2216
Cecidomyiidae|[5814]|GMAGE2518-15|Diptera|Cecidomyiidae||BOLD:ACN8355
Cecidomyiidae|[5815]|GMARA1486-14|Diptera|Cecidomyiidae||BOLD:ACM4846
Cecidomyiidae|[5816]|GMARB989-14|Diptera|Cecidomyiidae||BOLD:ACM6503
Cecidomyiidae|[5817]|GMARC479-14|Diptera|Cecidomyiidae||BOLD:ACM7180
Cecidomyiidae|[5818]|GMAGM1645-15|Diptera|Cecidomyiidae||BOLD:ACO0894
Cecidomyiidae|[5819]|GMARI658-14|Diptera|Cecidomyiidae||BOLD:ACN0260
Cecidomyiidae|[5820]|GMAGE2546-15|Diptera|Cecidomyiidae||BOLD:ACX4431
Cecidomyiidae|[5821]|GMARK881-14|Diptera|Cecidomyiidae||BOLD:ACN0860
Cecidomyiidae|[5822]|GMARD2114-14|Diptera|Cecidomyiidae||BOLD:ACN0718
Cecidomyiidae|[5823]|GMAGF341-15|Diptera|Cecidomyiidae||BOLD:ACW3158
Cecidomyiidae|[5824]|GMAGJ2009-15|Diptera|Cecidomyiidae||BOLD:ACN2017
Cecidomyiidae|[5825]|GMAGB1383-15|Diptera|Cecidomyiidae||BOLD:ACM6458
Cecidomyiidae|[5826]|GMAGD1124-15|Diptera|Cecidomyiidae||BOLD:ACN1775
Cecidomyiidae|[5827]|GMAGD2115-15|Diptera|Cecidomyiidae||BOLD:ACM5393
Cecidomyiidae|[5828]|GMARB797-14|Diptera|Cecidomyiidae||BOLD:ACM6586
Cecidomyiidae|[5829]|GMAGD2328-15|Diptera|Cecidomyiidae||BOLD:ACW9856
Cecidomyiidae|[5830]|GMARB1595-14|Diptera|Cecidomyiidae||BOLD:ACM6129
Cecidomyiidae|[5831]|GMARB1474-14|Diptera|Cecidomyiidae||BOLD:ACM6442
Cecidomyiidae|[5832]|GMAGD2261-15|Diptera|Cecidomyiidae||BOLD:ACW3483
Cecidomyiidae|[5833]|GMAGA869-15|Diptera|Cecidomyiidae||BOLD:ACM5382
Cecidomyiidae|[5834]|GMAGB1404-15|Diptera|Cecidomyiidae||BOLD:ACW4517
Cecidomyiidae|[5835]|GMAGE2710-15|Diptera|Cecidomyiidae||BOLD:ACW7582
Cecidomyiidae|[5836]|GMAGD1981-15|Diptera|Cecidomyiidae||BOLD:ACW1722
Cecidomyiidae|[5837]|GMARA1795-14|Diptera|Cecidomyiidae||BOLD:ACM5762
Cecidomyiidae|[5838]|GMAGD2127-15|Diptera|Cecidomyiidae||BOLD:ACN7654
Cecidomyiidae|[5839]|GMAGG334-15|Diptera|Cecidomyiidae||BOLD:ACW6865
Cecidomyiidae|[5840]|GMAGD1076-15|Diptera|Cecidomyiidae||BOLD:ACW3628
Cecidomyiidae|[5841]|GMAGB1419-15|Diptera|Cecidomyiidae||BOLD:ACM5694
Cecidomyiidae|[5842]|GMARJ884-14|Diptera|Cecidomyiidae||BOLD:ACN0181
Cecidomyiidae|[5843]|GMAGD2321-15|Diptera|Cecidomyiidae||BOLD:ACN1046
Cecidomyiidae|[5844]|GMAGE2654-15|Diptera|Cecidomyiidae||BOLD:ACN7910
Cecidomyiidae|[5845]|GMARF668-14|Diptera|Cecidomyiidae||BOLD:ACM8850
Cecidomyiidae|[5846]|GMARA197-14|Diptera|Cecidomyiidae||BOLD:ACM6620
Cecidomyiidae|[5847]|GMAGN2212-15|Diptera|Cecidomyiidae||BOLD:ACX1656
Cecidomyiidae|[5848]|GMAGS271-15|Diptera|Cecidomyiidae||BOLD:ACX4891
Cecidomyiidae|[5849]|GMAGK1096-15|Diptera|Cecidomyiidae||BOLD:ACX5874
Cecidomyiidae|[5850]|GMARR668-14|Diptera|Cecidomyiidae||BOLD:ACN7876
Cecidomyiidae|[5851]|GMARJ622-14|Diptera|Cecidomyiidae||BOLD:ACN5367
Cecidomyiidae|[5852]|GMAGB698-15|Diptera|Cecidomyiidae||BOLD:ACX4785
Cecidomyiidae|[5853]|GMARC473-14|Diptera|Cecidomyiidae||BOLD:ACM8007
Cecidomyiidae|[5854]|GMAGO214-15|Diptera|Cecidomyiidae||BOLD:ACX5164
Cecidomyiidae|[5855]|GMARA1419-14|Diptera|Cecidomyiidae||BOLD:ACM9273
Cecidomyiidae|[5856]|GMAGA467-15|Diptera|Cecidomyiidae||BOLD:ACM6062
Cecidomyiidae|[5857]|GMARB1500-14|Diptera|Cecidomyiidae||BOLD:ACM5238
Cecidomyiidae|[5858]|GMAGJ1786-15|Diptera|Cecidomyiidae||BOLD:ACX4758
Cecidomyiidae|[5859]|GMAGC136-15|Diptera|Cecidomyiidae||BOLD:ACM5621
Cecidomyiidae|[5860]|GMARH1295-14|Diptera|Cecidomyiidae||BOLD:ACW9228
Cecidomyiidae|[5861]|GMAGA537-15|Diptera|Cecidomyiidae||BOLD:ACM5499
Cecidomyiidae|[5862]|GMAGB1130-15|Diptera|Cecidomyiidae||BOLD:ACM5084
Cecidomyiidae|[5863]|GMAGO450-15|Diptera|Cecidomyiidae||BOLD:ACX5797
Cecidomyiidae|[5864]|GMARG1292-14|Diptera|Cecidomyiidae||BOLD:ACN1296
Cecidomyiidae|[5865]|GMARN1883-14|Diptera|Cecidomyiidae||BOLD:ACY4024
Cecidomyiidae|[5866]|GMAGA426-15|Diptera|Cecidomyiidae||BOLD:ACN1792
Cecidomyiidae|[5867]|GMAGI878-15|Diptera|Cecidomyiidae||BOLD:ACN4548
Cecidomyiidae|[5868]|GMARH1167-14|Diptera|Cecidomyiidae||BOLD:ACN0674
Cecidomyiidae|[5869]|GMARB827-14|Diptera|Cecidomyiidae||BOLD:ACM5297
Cecidomyiidae|[5870]|GMARB536-14|Diptera|Cecidomyiidae||BOLD:ACW4873
Cecidomyiidae|[5871]|GMAGE1924-15|Diptera|Cecidomyiidae||BOLD:ACM9280
Cecidomyiidae|[5872]|GMARQ155-14|Diptera|Cecidomyiidae||BOLD:ACN8119
Cecidomyiidae|[5873]|GMAGB1450-15|Diptera|Cecidomyiidae||BOLD:ACW4280
Cecidomyiidae|[5874]|GMARA1802-14|Diptera|Cecidomyiidae||BOLD:ACM6301

Cecidomyiidae|[5872]|GMARQ155-14|Diptera|Cecidomyiidae||BOLD:ACN8119
Cecidomyiidae|[5873]|GMAGB1450-15|Diptera|Cecidomyiidae||BOLD:ACW4280
Cecidomyiidae|[5874]|GMARA1802-14|Diptera|Cecidomyiidae||BOLD:ACM6301
Cecidomyiidae|[5875]|GMAGE2045-15|Diptera|Cecidomyiidae||BOLD:ACW6108
Cecidomyiidae|[5876]|GMAGB1108-15|Diptera|Cecidomyiidae||BOLD:ACW3069
Cecidomyiidae|[5877]|GMAGO797-15|Diptera|Cecidomyiidae||BOLD:ACO3944
Cecidomyiidae|[5878]|GMARU387-14|Diptera|Cecidomyiidae||BOLD:ACO0939
Cecidomyiidae|[5879]|GMAGA427-15|Diptera|Cecidomyiidae||BOLD:ACM9659
Cecidomyiidae|[5880]|GMAGY421-15|Diptera|Cecidomyiidae||BOLD:ACX3032
Cecidomyiidae|[5881]|GMARC801-14|Diptera|Cecidomyiidae||BOLD:ACM6286
Cecidomyiidae|[5882]|GMARM808-14|Diptera|Cecidomyiidae||BOLD:ACN6092
Cecidomyiidae|[5883]|GMARB877-14|Diptera|Cecidomyiidae||BOLD:ACM6396
Cecidomyiidae|[5884]|GMAGG1396-15|Diptera|Cecidomyiidae||BOLD:ACO0103
Cecidomyiidae|[5885]|GMAGM933-15|Diptera|Cecidomyiidae||BOLD:ACX3655
Cecidomyiidae|[5886]|GMAGD2221-15|Diptera|Cecidomyiidae||BOLD:ACW6463
Cecidomyiidae|[5887]|GMAGS1309-15|Diptera|Cecidomyiidae||BOLD:ACX4296
Cecidomyiidae|[5888]|GMAGM1944-15|Diptera|Cecidomyiidae||BOLD:ACN2691
Cecidomyiidae|[5889]|GMAGP1580-15|Diptera|Cecidomyiidae||BOLD:ACX6229
Cecidomyiidae|[5890]|GMAGP1782-15|Diptera|Cecidomyiidae||BOLD:ACN9578
Cecidomyiidae|[5891]|GMARA1235-14|Diptera|Cecidomyiidae||BOLD:ACM9283
Cecidomyiidae|[5892]|GMAGB1142-15|Diptera|Cecidomyiidae||BOLD:ACW7366
Cecidomyiidae|[5893]|GMARE174-14|Diptera|Cecidomyiidae||BOLD:ACN1142
Cecidomyiidae|[5894]|GMARA1169-14|Diptera|Cecidomyiidae||BOLD:ACM5234
Cecidomyiidae|[5895]|GMAGJ423-15|Diptera|Cecidomyiidae||BOLD:ACW8056
Cecidomyiidae|[5896]|GMARB1279-14|Diptera|Cecidomyiidae||BOLD:ACM5590
Cecidomyiidae|[5897]|GMARM158-14|Diptera|Cecidomyiidae||BOLD:ACN2574
Cecidomyiidae|[5898]|GMARB1731-14|Diptera|Cecidomyiidae||BOLD:ACM6370
Cecidomyiidae|[5899]|GMAGU517-15|Diptera|Cecidomyiidae||BOLD:ACX4247
Cecidomyiidae|[5900]|GMAGB829-15|Diptera|Cecidomyiidae||BOLD:ACN1297
Cecidomyiidae|[5901]|GMARI1826-14|Diptera|Cecidomyiidae||BOLD:ACN0355
Cecidomyiidae|[5902]|GMAGU170-15|Diptera|Cecidomyiidae||BOLD:ACX6113
Cecidomyiidae|[5903]|GMARD2157-14|Diptera|Cecidomyiidae||BOLD:ACN0635
Cecidomyiidae|[5904]|GMARD342-14|Diptera|Cecidomyiidae||BOLD:ACN0692
Cecidomyiidae|[5905]|GMAGK1540-15|Diptera|Cecidomyiidae||BOLD:ACW9619
Cecidomyiidae|[5906]|GMAGE1908-15|Diptera|Cecidomyiidae||BOLD:ACM8757
Cecidomyiidae|[5907]|GMARC694-14|Diptera|Cecidomyiidae||BOLD:ACM7806
Cecidomyiidae|[5908]|GMAGN2253-15|Diptera|Cecidomyiidae||BOLD:ACX2747
Cecidomyiidae|[5909]|GMAGD1050-15|Diptera|Cecidomyiidae||BOLD:ACN0006
Cecidomyiidae|[5910]|GMAGF467-15|Diptera|Cecidomyiidae||BOLD:ACM8288
Cecidomyiidae|[5911]|GMARA1432-14|Diptera|Cecidomyiidae||BOLD:ACM6324
Cecidomyiidae|[5912]|GMARA1536-14|Diptera|Cecidomyiidae||BOLD:ACM4923
Cecidomyiidae|[5913]|GMAGL2668-15|Diptera|Cecidomyiidae||BOLD:ACW9181
Cecidomyiidae|[5914]|GMARB1205-14|Diptera|Cecidomyiidae||BOLD:ACM4715
Cecidomyiidae|[5915]|GMARA1792-14|Diptera|Cecidomyiidae||BOLD:ACM6304
Cecidomyiidae|[5916]|GMARB1028-14|Diptera|Cecidomyiidae||BOLD:ACM6564
Cecidomyiidae|[5917]|GMAGL2729-15|Diptera|Cecidomyiidae||BOLD:ACW8766
Cecidomyiidae|[5918]|GMARA1388-14|Diptera|Cecidomyiidae||BOLD:ACX0565
Cecidomyiidae|[5919]|GMARK762-14|Diptera|Cecidomyiidae||BOLD:ACN0869
Cecidomyiidae|[5920]|GMAGC261-15|Diptera|Cecidomyiidae||BOLD:ACW2183
Cecidomyiidae|[5921]|GMARD676-14|Diptera|Cecidomyiidae||BOLD:ACN1641
Cecidomyiidae|[5922]|GMARE121-14|Diptera|Cecidomyiidae||BOLD:ACN1746
Cecidomyiidae|[5923]|GMAGD2348-15|Diptera|Cecidomyiidae||BOLD:ACN0003
Cecidomyiidae|[5924]|GMAGQ1233-15|Diptera|Cecidomyiidae||BOLD:ACX4339
Cecidomyiidae|[5925]|GMARE938-14|Diptera|Cecidomyiidae||BOLD:ACN1582
Cecidomyiidae|[5926]|GMARB616-14|Diptera|Cecidomyiidae||BOLD:ACM6162
Cecidomyiidae|[5927]|GMAGF429-15|Diptera|Cecidomyiidae||BOLD:ACN1581
Cecidomyiidae|[5928]|GMARE731-14|Diptera|Cecidomyiidae||BOLD:ACN1583
Cecidomyiidae|[5929]|GMAGM2203-15|Diptera|Cecidomyiidae||BOLD:ACX3568
Cecidomyiidae|[5930]|GMAGN2199-15|Diptera|Cecidomyiidae||BOLD:ACX3346
Cecidomyiidae|[5931]|GMAGG2013-15|Diptera|Cecidomyiidae||BOLD:ACN8632
Cecidomyiidae|[5932]|GMAGQ2246-15|Diptera|Cecidomyiidae||BOLD:ACX6223
Cecidomyiidae|[5933]|GMARA1255-14|Diptera|Cecidomyiidae||BOLD:ACM9660
Cecidomyiidae|[5934]|GMAGF185-15|Diptera|Cecidomyiidae||BOLD:ACW3724
Cecidomyiidae|[5935]|GMARG1098-14|Diptera|Cecidomyiidae||BOLD:ACN1550
Cecidomyiidae|[5936]|GMAGB1435-15|Diptera|Cecidomyiidae||BOLD:ACN1664
Cecidomyiidae|[5937]|GMARA1208-14|Diptera|Cecidomyiidae||BOLD:ACM9291
Cecidomyiidae|[5938]|GMAGL2731-15|Diptera|Cecidomyiidae||BOLD:ACN9227
Cecidomyiidae|[5939]|GMAGD900-15|Diptera|Cecidomyiidae||BOLD:ACM8767
Cecidomyiidae|[5940]|GMAGR654-15|Diptera|Cecidomyiidae||BOLD:ACX4934
Cecidomyiidae|[5941]|GMARB1367-14|Diptera|Cecidomyiidae||BOLD:ACM5849
Cecidomyiidae|[5942]|GMAGB1459-15|Diptera|Cecidomyiidae||BOLD:ACM5436
Cecidomyiidae|[5943]|GMAGM1675-15|Diptera|Cecidomyiidae||BOLD:ACX2904
Cecidomyiidae|[5944]|GMARB1132-14|Diptera|Cecidomyiidae||BOLD:ACM6136
Cecidomyiidae|[5945]|GMAGI692-15|Diptera|Cecidomyiidae||BOLD:ACW7742
Cecidomyiidae|[5946]|GMAGO454-15|Diptera|Cecidomyiidae||BOLD:ACX5318
Cecidomyiidae|[5947]|GMAGH1194-15|Diptera|Cecidomyiidae||BOLD:ACY6996
Cecidomyiidae|[5948]|GMAGK1727-15|Diptera|Cecidomyiidae||BOLD:ACN1254
Cecidomyiidae|[5949]|GMARB1309-14|Diptera|Cecidomyiidae||BOLD:ACM5870
Cecidomyiidae|[5950]|GMARA1417-14|Diptera|Cecidomyiidae||BOLD:ACM6131
Cecidomyiidae|[5951]|GMAGE2282-15|Diptera|Cecidomyiidae||BOLD:ACW6801
Cecidomyiidae|[5952]|GMARR296-14|Diptera|Cecidomyiidae||BOLD:ACN8813
Cecidomyiidae|[5953]|GMARA1271-14|Diptera|Cecidomyiidae||BOLD:ACM8465
Cecidomyiidae|[5954]|GMARB1114-14|Diptera|Cecidomyiidae||BOLD:ACM5218
Cecidomyiidae|[5955]|GMARB754-14|Diptera|Cecidomyiidae||BOLD:ACM6614
Cecidomyiidae|[5956]|GMARA1285-14|Diptera|Cecidomyiidae||BOLD:ACM5709
Cecidomyiidae|[5957]|GMARB933-14|Diptera|Cecidomyiidae||BOLD:ACM5761
Cecidomyiidae|[5958]|GMAGB772-15|Diptera|Cecidomyiidae||BOLD:ACW1854
Cecidomyiidae|[5959]|GMARD1274-14|Diptera|Cecidomyiidae||BOLD:ACM8678
Cecidomyiidae|[5960]|GMARE226-14|Diptera|Cecidomyiidae||BOLD:ACN0887
Cecidomyiidae|[5961]|GMARA1289-14|Diptera|Cecidomyiidae||BOLD:ACM8786
Cecidomyiidae|[5962]|GMARK759-14|Diptera|Cecidomyiidae||BOLD:ACN1168
Cecidomyiidae|[5963]|GMART392-14|Diptera|Cecidomyiidae||BOLD:ACN9433
Cecidomyiidae|[5964]|GMAGL2312-15|Diptera|Cecidomyiidae||BOLD:ACX3577
Cecidomyiidae|[5965]|GMARM835-14|Diptera|Cecidomyiidae||BOLD:ACN6574
Cecidomyiidae|[5966]|GMARA1264-14|Diptera|Cecidomyiidae||BOLD:ACM9638
Cecidomyiidae|[5967]|GMARP686-14|Diptera|Cecidomyiidae||BOLD:ACO3252
Cecidomyiidae|[5968]|GMARB164-14|Diptera|Cecidomyiidae||BOLD:ACM5802
Cecidomyiidae|[5969]|GMARA1205-14|Diptera|Cecidomyiidae||BOLD:ACM6156
Cecidomyiidae|[5970]|GMAGN2281-15|Diptera|Cecidomyiidae||BOLD:ACN8976
Cecidomyiidae|[5971]|GMARJ1331-14|Diptera|Cecidomyiidae||BOLD:ACN1652
Cecidomyiidae|[5972]|GMAGE2614-15|Diptera|Cecidomyiidae||BOLD:ACN2176
Cecidomyiidae|[5973]|GMARK1882-14|Diptera|Cecidomyiidae||BOLD:ACN0652
Cecidomyiidae|[5974]|GMAGR344-15|Diptera|Cecidomyiidae||BOLD:ACX6700

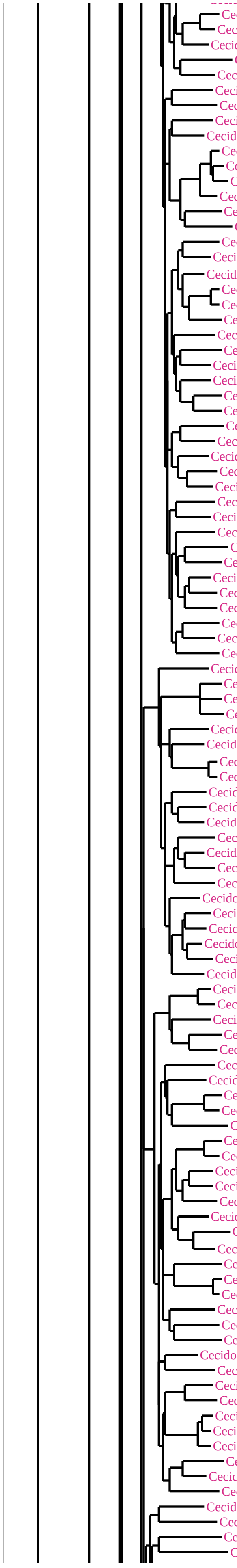

[illegible]
Cecidomyiidae|[5972]|GMAGE2614-15|Diptera|Cecidomyiidae||BOLD:ACN2176
Cecidomyiidae|[5973]|GMARK1882-14|Diptera|Cecidomyiidae||BOLD:ACN0652
Cecidomyiidae|[5974]|GMAGR344-15|Diptera|Cecidomyiidae||BOLD:ACX6700
Cecidomyiidae|[5975]|GMAGE2541-15|Diptera|Cecidomyiidae||BOLD:ACM7867
Cecidomyiidae|[5976]|GMARS476-14|Diptera|Cecidomyiidae||BOLD:ACN7584
Cecidomyiidae|[5977]|GMARA337-14|Diptera|Cecidomyiidae||BOLD:ACM9292
Cecidomyiidae|[5978]|GMAGO359-15|Diptera|Cecidomyiidae||BOLD:ACN2862
Cecidomyiidae|[5979]|GMAGJ1735-15|Diptera|Cecidomyiidae||BOLD:ACN0940
Cecidomyiidae|[5980]|GMARJ217-14|Diptera|Cecidomyiidae||BOLD:ACN4734
Cecidomyiidae|[5981]|GMAGC416-15|Diptera|Cecidomyiidae||BOLD:ACM5498
Cecidomyiidae|[5982]|GMARA1789-14|Diptera|Cecidomyiidae||BOLD:ACM7480
Cecidomyiidae|[5983]|GMARB834-14|Diptera|Cecidomyiidae||BOLD:ACM5358
Cecidomyiidae|[5984]|GMAGM1899-15|Diptera|Cecidomyiidae||BOLD:ACN6005
Cecidomyiidae|[5985]|GMAGM1964-15|Diptera|Cecidomyiidae||BOLD:ACO1681
Cecidomyiidae|[5986]|GMAGS1335-15|Diptera|Cecidomyiidae||BOLD:ACX4527
Cecidomyiidae|[5987]|GMAGE2137-15|Diptera|Cecidomyiidae||BOLD:ACN9334
Cecidomyiidae|[5988]|GMAGE2706-15|Diptera|Cecidomyiidae||BOLD:ACN0729
Cecidomyiidae|[5989]|GMAGB1548-15|Diptera|Cecidomyiidae||BOLD:ACM5831
Cecidomyiidae|[5990]|GMAGA441-15|Diptera|Cecidomyiidae||BOLD:ACM4763
Cecidomyiidae|[5991]|GMARD701-14|Diptera|Cecidomyiidae||BOLD:ACW6183
Cecidomyiidae|[5992]|GMAGF352-15|Diptera|Cecidomyiidae||BOLD:ACM7580
Cecidomyiidae|[5993]|GMAGN2366-15|Diptera|Cecidomyiidae||BOLD:ACX1322
Cecidomyiidae|[5994]|GMARA1146-14|Diptera|Cecidomyiidae||BOLD:ACV4798
Cecidomyiidae|[5995]|GMARC085-14|Diptera|Cecidomyiidae||BOLD:ACX3041
Cecidomyiidae|[5996]|GMAGN2131-15|Diptera|Cecidomyiidae||BOLD:ACX1578
Cecidomyiidae|[5997]|GMARB808-14|Diptera|Cecidomyiidae||BOLD:ACM5457
Cecidomyiidae|[5998]|GMARP080-14|Diptera|Cecidomyiidae||BOLD:ACO3554
Cecidomyiidae|[5999]|GMAGD1992-15|Diptera|Cecidomyiidae||BOLD:ACW2808
Cecidomyiidae|[6000]|GMAGD1281-15|Diptera|Cecidomyiidae||BOLD:ACN0175
Cecidomyiidae|[6001]|GMAGC262-15|Diptera|Cecidomyiidae||BOLD:ACN2026
Cecidomyiidae|[6002]|GMARJ088-14|Diptera|Cecidomyiidae||BOLD:ACN0067
Cecidomyiidae|[6003]|GMARR391-14|Diptera|Cecidomyiidae||BOLD:ACN8347
Cecidomyiidae|[6004]|GMARA1370-14|Diptera|Cecidomyiidae||BOLD:ACM9793
Cecidomyiidae|[6005]|GMARG1254-14|Diptera|Cecidomyiidae||BOLD:ACN0650
Cecidomyiidae|[6006]|GMAGL2217-15|Diptera|Cecidomyiidae||BOLD:ACN7920
Cecidomyiidae|[6007]|GMARA1286-14|Diptera|Cecidomyiidae||BOLD:ACM6398
Cecidomyiidae|[6008]|GMARB1380-14|Diptera|Cecidomyiidae||BOLD:ACM5574
Cecidomyiidae|[6009]|GMARA1135-14|Diptera|Cecidomyiidae||BOLD:ACV3324
Cecidomyiidae|[6010]|GMAGH1221-15|Diptera|Cecidomyiidae||BOLD:ACW2980
Cecidomyiidae|[6011]|GMARI066-14|Diptera|Cecidomyiidae||BOLD:ACN4277
Cecidomyiidae|[6012]|GMARC628-14|Diptera|Cecidomyiidae||BOLD:ACM7258
Cecidomyiidae|[6013]|GMARS130-14|Diptera|Cecidomyiidae||BOLD:ACN8846
Cecidomyiidae|[6014]|GMAGY540-15|Diptera|Cecidomyiidae||BOLD:ACX9887
Cecidomyiidae|[6015]|GMAGD1083-15|Diptera|Cecidomyiidae||BOLD:ACX2078
Cecidomyiidae|[6016]|GMAGI1007-15|Diptera|Cecidomyiidae||BOLD:ACN9062
Cecidomyiidae|[6017]|GMAGG1518-15|Diptera|Cecidomyiidae||BOLD:ACW2263
Cecidomyiidae|[6018]|GMAGJ1657-15|Diptera|Cecidomyiidae||BOLD:ACN1080
Cecidomyiidae|[6019]|GMAGD805-15|Diptera|Cecidomyiidae||BOLD:ACN0276
Cecidomyiidae|[6020]|GMAGH666-15|Diptera|Cecidomyiidae||BOLD:ACO3942
Cecidomyiidae|[6021]|GMAGD1042-15|Diptera|Cecidomyiidae||BOLD:ACW5984
Cecidomyiidae|[6022]|GMAGI294-15|Diptera|Cecidomyiidae||BOLD:ACN1728
Cecidomyiidae|[6023]|GMARD922-14|Diptera|Cecidomyiidae||BOLD:ACM9578
Cecidomyiidae|[6024]|GMAGD1308-15|Diptera|Cecidomyiidae||BOLD:ACW4871
Cecidomyiidae|[6025]|GMAGU624-15|Diptera|Cecidomyiidae||BOLD:ACX4779
Cecidomyiidae|[6026]|GMAGD1154-15|Diptera|Cecidomyiidae||BOLD:ACN4553
Cecidomyiidae|[6027]|GMAGD773-15|Diptera|Cecidomyiidae||BOLD:ACN1289
Cecidomyiidae|[6028]|GMAGH342-15|Diptera|Cecidomyiidae||BOLD:ACN3664
Cecidomyiidae|[6029]|GMAGJ421-15|Diptera|Cecidomyiidae||BOLD:ACN0036
Cecidomyiidae|[6030]|GMAGP1556-15|Diptera|Cecidomyiidae||BOLD:ACX5650
Cecidomyiidae|[6031]|GMARE682-14|Diptera|Cecidomyiidae||BOLD:ACN2631
Cecidomyiidae|[6032]|GMAGP1468-15|Diptera|Cecidomyiidae||BOLD:ACO0485
Cecidomyiidae|[6033]|GMAGQ911-15|Diptera|Cecidomyiidae||BOLD:ACX5853
Cecidomyiidae|[6034]|GMAGS1038-15|Diptera|Cecidomyiidae||BOLD:ACX5951
Cecidomyiidae|[6035]|GMAGU165-15|Diptera|Cecidomyiidae||BOLD:ACX2336
Cecidomyiidae|[6036]|GMARA1204-14|Diptera|Cecidomyiidae||BOLD:ACM6277
Cecidomyiidae|[6037]|GMAGC284-15|Diptera|Cecidomyiidae||BOLD:ACW6407
Cecidomyiidae|[6038]|GMARA272-14|Diptera|Cecidomyiidae||BOLD:ACM9546
Cecidomyiidae|[6039]|GMAGE1719-15|Diptera|Cecidomyiidae||BOLD:ACW2393
Cecidomyiidae|[6040]|GMAGL2418-15|Diptera|Cecidomyiidae||BOLD:ACN1132
Cecidomyiidae|[6041]|GMARS415-14|Diptera|Cecidomyiidae||BOLD:ACN7804
Cecidomyiidae|[6042]|GMART357-14|Diptera|Cecidomyiidae||BOLD:ACN9177
Cecidomyiidae|[6043]|GMARF209-14|Diptera|Cecidomyiidae||BOLD:ACM8384
Cecidomyiidae|[6044]|GMAGO558-15|Diptera|Cecidomyiidae||BOLD:ACX4473
Cecidomyiidae|[6045]|GMART350-14|Diptera|Cecidomyiidae||BOLD:ACN9249
Cecidomyiidae|[6046]|GMARA1237-14|Diptera|Cecidomyiidae||BOLD:ACM9695
Cecidomyiidae|[6047]|GMAGH435-15|Diptera|Cecidomyiidae||BOLD:ACW2059
Cecidomyiidae|[6048]|GMAGA425-15|Diptera|Cecidomyiidae||BOLD:ACM6434
Cecidomyiidae|[6049]|GMAGD1134-15|Diptera|Cecidomyiidae||BOLD:ACN0642
Cecidomyiidae|[6050]|GMARI840-14|Diptera|Cecidomyiidae||BOLD:ACN0707
Cecidomyiidae|[6051]|GMARJ142-14|Diptera|Cecidomyiidae||BOLD:ACM9901
Cecidomyiidae|[6052]|GMAGB679-15|Diptera|Cecidomyiidae||BOLD:ACM9591
Cecidomyiidae|[6053]|GMAGO316-15|Diptera|Cecidomyiidae||BOLD:ACX4894
Cecidomyiidae|[6054]|GMAGC603-15|Diptera|Cecidomyiidae||BOLD:ACN9683
Cecidomyiidae|[6055]|GMARD1002-14|Diptera|Cecidomyiidae||BOLD:ACM9773
Cecidomyiidae|[6056]|GMAGE599-15|Diptera|Cecidomyiidae||BOLD:ACY4645
Cecidomyiidae|[6057]|GMARG911-14|Diptera|Cecidomyiidae||BOLD:ACN0817
Cecidomyiidae|[6058]|GMARR385-14|Diptera|Cecidomyiidae||BOLD:ACN7997
Cecidomyiidae|[6059]|GMART1077-14|Diptera|Cecidomyiidae||BOLD:ACO0791
Cecidomyiidae|[6060]|GMARK2073-14|Diptera|Cecidomyiidae||BOLD:ACN2395
Cecidomyiidae|[6061]|GMAGP1263-15|Diptera|Cecidomyiidae||BOLD:ACX5802
Cecidomyiidae|[6062]|GMARA1192-14|Diptera|Cecidomyiidae||BOLD:ACM9271
Cecidomyiidae|[6063]|GMARD1509-14|Diptera|Cecidomyiidae||BOLD:ACN0660
Cecidomyiidae|[6064]|GMAGB529-15|Diptera|Cecidomyiidae||BOLD:ACN2951
Cecidomyiidae|[6065]|GMARB1229-14|Diptera|Cecidomyiidae||BOLD:ACM4718
Cecidomyiidae|[6066]|GMART1244-14|Diptera|Cecidomyiidae||BOLD:ACO0804
Cecidomyiidae|[6067]|GMARA264-14|Diptera|Cecidomyiidae||BOLD:ACM9399
Cecidomyiidae|[6068]|GMAGB461-15|Diptera|Cecidomyiidae||BOLD:ACM4981
Cecidomyiidae|[6069]|GMARU337-14|Diptera|Cecidomyiidae||BOLD:ACO0820
Cecidomyiidae|[6070]|GMARA1294-14|Diptera|Cecidomyiidae||BOLD:ACM8753
Cecidomyiidae|[6071]|GMAGB1535-15|Diptera|Cecidomyiidae||BOLD:ACO1599
Cecidomyiidae|[6072]|GMAGD1016-15|Diptera|Cecidomyiidae||BOLD:ACN9654
Cecidomyiidae|[6073]|GMAGK1185-15|Diptera|Cecidomyiidae||BOLD:ACW8681
[illegible]

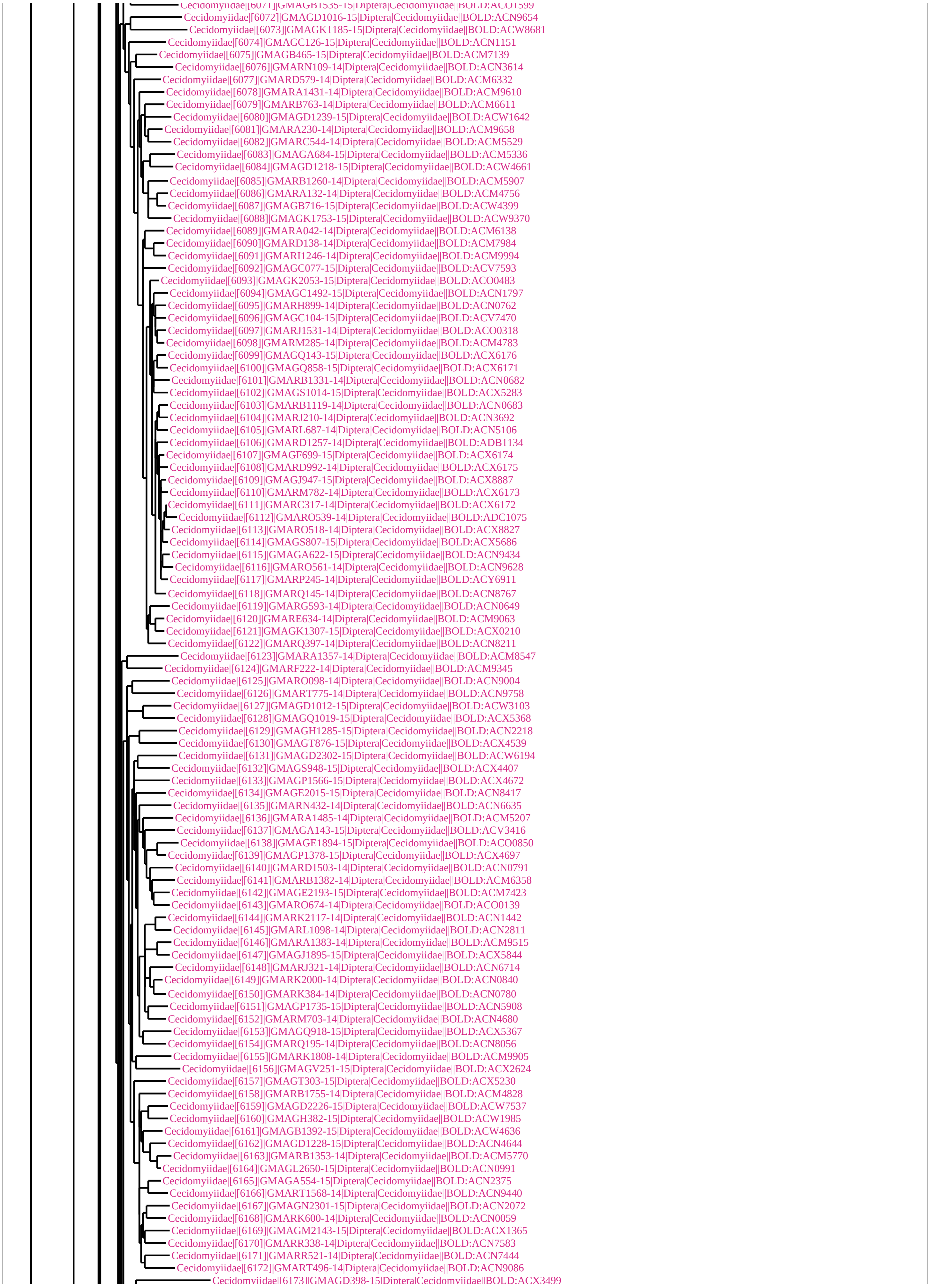
Cecidomyiidae|[6071]|GMAGB1535-15|Diptera|Cecidomyiidae||BOLD:ACO1599
Cecidomyiidae|[6072]|GMAGD1016-15|Diptera|Cecidomyiidae||BOLD:ACN9654
Cecidomyiidae|[6073]|GMAGK1185-15|Diptera|Cecidomyiidae||BOLD:ACW8681
Cecidomyiidae|[6074]|GMAGC126-15|Diptera|Cecidomyiidae||BOLD:ACN1151
Cecidomyiidae|[6075]|GMAGB465-15|Diptera|Cecidomyiidae||BOLD:ACM7139
Cecidomyiidae|[6076]|GMARN109-14|Diptera|Cecidomyiidae||BOLD:ACN3614
Cecidomyiidae|[6077]|GMARD579-14|Diptera|Cecidomyiidae||BOLD:ACM6332
Cecidomyiidae|[6078]|GMARA1431-14|Diptera|Cecidomyiidae||BOLD:ACM9610
Cecidomyiidae|[6079]|GMARB763-14|Diptera|Cecidomyiidae||BOLD:ACM6611
Cecidomyiidae|[6080]|GMAGD1239-15|Diptera|Cecidomyiidae||BOLD:ACW1642
Cecidomyiidae|[6081]|GMARA230-14|Diptera|Cecidomyiidae||BOLD:ACM9658
Cecidomyiidae|[6082]|GMARC544-14|Diptera|Cecidomyiidae||BOLD:ACM5529
Cecidomyiidae|[6083]|GMAGA684-15|Diptera|Cecidomyiidae||BOLD:ACM5336
Cecidomyiidae|[6084]|GMAGD1218-15|Diptera|Cecidomyiidae||BOLD:ACW4661
Cecidomyiidae|[6085]|GMARB1260-14|Diptera|Cecidomyiidae||BOLD:ACM5907
Cecidomyiidae|[6086]|GMARA132-14|Diptera|Cecidomyiidae||BOLD:ACM4756
Cecidomyiidae|[6087]|GMAGB716-15|Diptera|Cecidomyiidae||BOLD:ACW4399
Cecidomyiidae|[6088]|GMAGK1753-15|Diptera|Cecidomyiidae||BOLD:ACW9370
Cecidomyiidae|[6089]|GMARA042-14|Diptera|Cecidomyiidae||BOLD:ACM6138
Cecidomyiidae|[6090]|GMARD138-14|Diptera|Cecidomyiidae||BOLD:ACM7984
Cecidomyiidae|[6091]|GMARI1246-14|Diptera|Cecidomyiidae||BOLD:ACM9994
Cecidomyiidae|[6092]|GMAGC077-15|Diptera|Cecidomyiidae||BOLD:ACV7593
Cecidomyiidae|[6093]|GMAGK2053-15|Diptera|Cecidomyiidae||BOLD:ACO0483
Cecidomyiidae|[6094]|GMAGC1492-15|Diptera|Cecidomyiidae||BOLD:ACN1797
Cecidomyiidae|[6095]|GMARH899-14|Diptera|Cecidomyiidae||BOLD:ACN0762
Cecidomyiidae|[6096]|GMAGC104-15|Diptera|Cecidomyiidae||BOLD:ACV7470
Cecidomyiidae|[6097]|GMARJ1531-14|Diptera|Cecidomyiidae||BOLD:ACO0318
Cecidomyiidae|[6098]|GMARM285-14|Diptera|Cecidomyiidae||BOLD:ACM4783
Cecidomyiidae|[6099]|GMAGQ143-15|Diptera|Cecidomyiidae||BOLD:ACX6176
Cecidomyiidae|[6100]|GMAGQ858-15|Diptera|Cecidomyiidae||BOLD:ACX6171
Cecidomyiidae|[6101]|GMARB1331-14|Diptera|Cecidomyiidae||BOLD:ACN0682
Cecidomyiidae|[6102]|GMAGS1014-15|Diptera|Cecidomyiidae||BOLD:ACX5283
Cecidomyiidae|[6103]|GMARB1119-14|Diptera|Cecidomyiidae||BOLD:ACN0683
Cecidomyiidae|[6104]|GMARJ210-14|Diptera|Cecidomyiidae||BOLD:ACN3692
Cecidomyiidae|[6105]|GMARL687-14|Diptera|Cecidomyiidae||BOLD:ACN5106
Cecidomyiidae|[6106]|GMARD1257-14|Diptera|Cecidomyiidae||BOLD:ADB1134
Cecidomyiidae|[6107]|GMAGF699-15|Diptera|Cecidomyiidae||BOLD:ACX6174
Cecidomyiidae|[6108]|GMARD992-14|Diptera|Cecidomyiidae||BOLD:ACX6175
Cecidomyiidae|[6109]|GMAGJ947-15|Diptera|Cecidomyiidae||BOLD:ACX8887
Cecidomyiidae|[6110]|GMARM782-14|Diptera|Cecidomyiidae||BOLD:ACX6173
Cecidomyiidae|[6111]|GMARC317-14|Diptera|Cecidomyiidae||BOLD:ACX6172
Cecidomyiidae|[6112]|GMARO539-14|Diptera|Cecidomyiidae||BOLD:ADC1075
Cecidomyiidae|[6113]|GMARO518-14|Diptera|Cecidomyiidae||BOLD:ACX8827
Cecidomyiidae|[6114]|GMAGS807-15|Diptera|Cecidomyiidae||BOLD:ACX5686
Cecidomyiidae|[6115]|GMAGA622-15|Diptera|Cecidomyiidae||BOLD:ACN9434
Cecidomyiidae|[6116]|GMARO561-14|Diptera|Cecidomyiidae||BOLD:ACN9628
Cecidomyiidae|[6117]|GMARP245-14|Diptera|Cecidomyiidae||BOLD:ACY6911
Cecidomyiidae|[6118]|GMARQ145-14|Diptera|Cecidomyiidae||BOLD:ACN8767
Cecidomyiidae|[6119]|GMARG593-14|Diptera|Cecidomyiidae||BOLD:ACN0649
Cecidomyiidae|[6120]|GMARE634-14|Diptera|Cecidomyiidae||BOLD:ACM9063
Cecidomyiidae|[6121]|GMAGK1307-15|Diptera|Cecidomyiidae||BOLD:ACX0210
Cecidomyiidae|[6122]|GMARQ397-14|Diptera|Cecidomyiidae||BOLD:ACN8211
Cecidomyiidae|[6123]|GMARA1357-14|Diptera|Cecidomyiidae||BOLD:ACM8547
Cecidomyiidae|[6124]|GMARF222-14|Diptera|Cecidomyiidae||BOLD:ACM9345
Cecidomyiidae|[6125]|GMARO098-14|Diptera|Cecidomyiidae||BOLD:ACN9004
Cecidomyiidae|[6126]|GMART775-14|Diptera|Cecidomyiidae||BOLD:ACN9758
Cecidomyiidae|[6127]|GMAGD1012-15|Diptera|Cecidomyiidae||BOLD:ACW3103
Cecidomyiidae|[6128]|GMAGQ1019-15|Diptera|Cecidomyiidae||BOLD:ACX5368
Cecidomyiidae|[6129]|GMAGH1285-15|Diptera|Cecidomyiidae||BOLD:ACN2218
Cecidomyiidae|[6130]|GMAGT876-15|Diptera|Cecidomyiidae||BOLD:ACX4539
Cecidomyiidae|[6131]|GMAGD2302-15|Diptera|Cecidomyiidae||BOLD:ACW6194
Cecidomyiidae|[6132]|GMAGS948-15|Diptera|Cecidomyiidae||BOLD:ACX4407
Cecidomyiidae|[6133]|GMAGP1566-15|Diptera|Cecidomyiidae||BOLD:ACX4672
Cecidomyiidae|[6134]|GMAGE2015-15|Diptera|Cecidomyiidae||BOLD:ACN8417
Cecidomyiidae|[6135]|GMARN432-14|Diptera|Cecidomyiidae||BOLD:ACN6635
Cecidomyiidae|[6136]|GMARA1485-14|Diptera|Cecidomyiidae||BOLD:ACM5207
Cecidomyiidae|[6137]|GMAGA143-15|Diptera|Cecidomyiidae||BOLD:ACV3416
Cecidomyiidae|[6138]|GMAGE1894-15|Diptera|Cecidomyiidae||BOLD:ACO0850
Cecidomyiidae|[6139]|GMAGP1378-15|Diptera|Cecidomyiidae||BOLD:ACX4697
Cecidomyiidae|[6140]|GMARD1503-14|Diptera|Cecidomyiidae||BOLD:ACN0791
Cecidomyiidae|[6141]|GMARB1382-14|Diptera|Cecidomyiidae||BOLD:ACM6358
Cecidomyiidae|[6142]|GMAGE2193-15|Diptera|Cecidomyiidae||BOLD:ACM7423
Cecidomyiidae|[6143]|GMARO674-14|Diptera|Cecidomyiidae||BOLD:ACO0139
Cecidomyiidae|[6144]|GMARK2117-14|Diptera|Cecidomyiidae||BOLD:ACN1442
Cecidomyiidae|[6145]|GMARL1098-14|Diptera|Cecidomyiidae||BOLD:ACN2811
Cecidomyiidae|[6146]|GMARA1383-14|Diptera|Cecidomyiidae||BOLD:ACM9515
Cecidomyiidae|[6147]|GMAGJ1895-15|Diptera|Cecidomyiidae||BOLD:ACX5844
Cecidomyiidae|[6148]|GMARJ321-14|Diptera|Cecidomyiidae||BOLD:ACN6714
Cecidomyiidae|[6149]|GMARK2000-14|Diptera|Cecidomyiidae||BOLD:ACN0840
Cecidomyiidae|[6150]|GMARK384-14|Diptera|Cecidomyiidae||BOLD:ACN0780
Cecidomyiidae|[6151]|GMAGP1735-15|Diptera|Cecidomyiidae||BOLD:ACN5908
Cecidomyiidae|[6152]|GMARM703-14|Diptera|Cecidomyiidae||BOLD:ACN4680
Cecidomyiidae|[6153]|GMAGQ918-15|Diptera|Cecidomyiidae||BOLD:ACX5367
Cecidomyiidae|[6154]|GMARQ195-14|Diptera|Cecidomyiidae||BOLD:ACN8056
Cecidomyiidae|[6155]|GMARK1808-14|Diptera|Cecidomyiidae||BOLD:ACM9905
Cecidomyiidae|[6156]|GMAGV251-15|Diptera|Cecidomyiidae||BOLD:ACX2624
Cecidomyiidae|[6157]|GMAGT303-15|Diptera|Cecidomyiidae||BOLD:ACX5230
Cecidomyiidae|[6158]|GMARB1755-14|Diptera|Cecidomyiidae||BOLD:ACM4828
Cecidomyiidae|[6159]|GMAGD2226-15|Diptera|Cecidomyiidae||BOLD:ACW7537
Cecidomyiidae|[6160]|GMAGH382-15|Diptera|Cecidomyiidae||BOLD:ACW1985
Cecidomyiidae|[6161]|GMAGB1392-15|Diptera|Cecidomyiidae||BOLD:ACW4636
Cecidomyiidae|[6162]|GMAGD1228-15|Diptera|Cecidomyiidae||BOLD:ACN4644
Cecidomyiidae|[6163]|GMARB1353-14|Diptera|Cecidomyiidae||BOLD:ACM5770
Cecidomyiidae|[6164]|GMAGL2650-15|Diptera|Cecidomyiidae||BOLD:ACN0991
Cecidomyiidae|[6165]|GMAGA554-15|Diptera|Cecidomyiidae||BOLD:ACN2375
Cecidomyiidae|[6166]|GMART1568-14|Diptera|Cecidomyiidae||BOLD:ACN9440
Cecidomyiidae|[6167]|GMAGN2301-15|Diptera|Cecidomyiidae||BOLD:ACN2072
Cecidomyiidae|[6168]|GMARK600-14|Diptera|Cecidomyiidae||BOLD:ACN0059
Cecidomyiidae|[6169]|GMAGM2143-15|Diptera|Cecidomyiidae||BOLD:ACX1365
Cecidomyiidae|[6170]|GMARR338-14|Diptera|Cecidomyiidae||BOLD:ACN7583
Cecidomyiidae|[6171]|GMARR521-14|Diptera|Cecidomyiidae||BOLD:ACN7444
Cecidomyiidae|[6172]|GMART496-14|Diptera|Cecidomyiidae||BOLD:ACN9086
Cecidomyiidae|[6173]|GMAGD398-15|Diptera|Cecidomyiidae||BOLD:ACX3499

Cecidomyiidae|[6171]|GMARR521-14|Diptera|Cecidomyiidae||BOLD:ACN7444
Cecidomyiidae|[6172]|GMART496-14|Diptera|Cecidomyiidae||BOLD:ACN9086
Cecidomyiidae|[6173]|GMAGD398-15|Diptera|Cecidomyiidae||BOLD:ACX3499
Cecidomyiidae|[6174]|GMAGM1115-15|Diptera|Cecidomyiidae||BOLD:ACX3881
Cecidomyiidae|[6175]|GMARM817-14|Diptera|Cecidomyiidae||BOLD:ACN2383
Cecidomyiidae|[6176]|GMAGQ1137-15|Diptera|Cecidomyiidae||BOLD:ACX5499
Cecidomyiidae|[6177]|GMAGP1193-15|Diptera|Cecidomyiidae||BOLD:ACN7587
Cecidomyiidae|[6178]|GMARA360-14|Diptera|Cecidomyiidae||BOLD:ACM8925
Cecidomyiidae|[6179]|GMARN947-14|Diptera|Cecidomyiidae||BOLD:ACX2389
Cecidomyiidae|[6180]|GMAGD1003-15|Diptera|Cecidomyiidae||BOLD:ACW2960
Cecidomyiidae|[6181]|GMARL1643-14|Diptera|Cecidomyiidae||BOLD:ACN2654
Cecidomyiidae|[6182]|GMARL222-14|Diptera|Cecidomyiidae||BOLD:ACN2624
Cecidomyiidae|[6183]|GMAGQ1073-15|Diptera|Cecidomyiidae||BOLD:ACX4228
Cecidomyiidae|[6184]|GMARA1276-14|Diptera|Cecidomyiidae||BOLD:ACM5154
Cecidomyiidae|[6185]|GMARA317-14|Diptera|Cecidomyiidae||BOLD:ACM8224
Cecidomyiidae|[6186]|GMARN1897-14|Diptera|Cecidomyiidae||BOLD:ACN6590
Cecidomyiidae|[6187]|GMAGB420-15|Diptera|Cecidomyiidae||BOLD:ACW4225
Cecidomyiidae|[6188]|GMAGH605-15|Diptera|Cecidomyiidae||BOLD:ACM9506
Cecidomyiidae|[6189]|GMARM073-14|Diptera|Cecidomyiidae||BOLD:ACN2351
Cecidomyiidae|[6190]|GMAGQ848-15|Diptera|Cecidomyiidae||BOLD:ACN4843
Cecidomyiidae|[6191]|GMAGQ1128-15|Diptera|Cecidomyiidae||BOLD:ACX4192
Cecidomyiidae|[6192]|GMARA267-14|Diptera|Cecidomyiidae||BOLD:ACM8505
Cecidomyiidae|[6193]|GMAGR685-15|Diptera|Cecidomyiidae||BOLD:ACX4935
Cecidomyiidae|[6194]|GMAGB318-15|Diptera|Cecidomyiidae||BOLD:ACW1479
Cecidomyiidae|[6195]|GMAGE1952-15|Diptera|Cecidomyiidae||BOLD:ACW7609
Cecidomyiidae|[6196]|GMAGQ1932-15|Diptera|Cecidomyiidae||BOLD:ACX5888
Cecidomyiidae|[6197]|GMARM658-14|Diptera|Cecidomyiidae||BOLD:ACN9957
Cecidomyiidae|[6198]|GMAGQ1758-15|Diptera|Cecidomyiidae||BOLD:ACX5094
Cecidomyiidae|[6199]|GMAGS1050-15|Diptera|Cecidomyiidae||BOLD:ACX6259
Cecidomyiidae|[6200]|GMAGC143-15|Diptera|Cecidomyiidae||BOLD:ACV6434
Cecidomyiidae|[6201]|GMAGD1311-15|Diptera|Cecidomyiidae||BOLD:ACW4331
Cecidomyiidae|[6202]|GMARA1402-14|Diptera|Cecidomyiidae||BOLD:ACM9073
Cecidomyiidae|[6203]|GMAGD1194-15|Diptera|Cecidomyiidae||BOLD:ACW4259
Cecidomyiidae|[6204]|GMARB1684-14|Diptera|Cecidomyiidae||BOLD:ACM5215
Cecidomyiidae|[6205]|GMAGP826-15|Diptera|Cecidomyiidae||BOLD:ACX6187
Cecidomyiidae|[6206]|GMAGU514-15|Diptera|Cecidomyiidae||BOLD:ACX5043
Cecidomyiidae|[6207]|GMARA1222-14|Diptera|Cecidomyiidae||BOLD:ACM8915
Cecidomyiidae|[6208]|GMART368-14|Diptera|Cecidomyiidae||BOLD:ACO0146
Cecidomyiidae|[6209]|GMARD1150-14|Diptera|Cecidomyiidae||BOLD:ACM8928
Cecidomyiidae|[6210]|GMARF1066-14|Diptera|Cecidomyiidae||BOLD:ACN1435
Cecidomyiidae|[6211]|GMAGG1416-15|Diptera|Cecidomyiidae||BOLD:ACW3790
Cecidomyiidae|[6212]|GMAGG1733-15|Diptera|Cecidomyiidae||BOLD:ACW7055
Cecidomyiidae|[6213]|GMAGJ2049-15|Diptera|Cecidomyiidae||BOLD:ACX4816
Cecidomyiidae|[6214]|GMARE400-14|Diptera|Cecidomyiidae||BOLD:ACN0927
Cecidomyiidae|[6215]|GMARK1268-14|Diptera|Cecidomyiidae||BOLD:ACN0309
Cecidomyiidae|[6216]|GMAGE1911-15|Diptera|Cecidomyiidae||BOLD:ACW7060
Cecidomyiidae|[6217]|GMAGN1915-15|Diptera|Cecidomyiidae||BOLD:ACX3026
Cecidomyiidae|[6218]|GMAGE2583-15|Diptera|Cecidomyiidae||BOLD:ACM8390
Cecidomyiidae|[6219]|GMAGH426-15|Diptera|Cecidomyiidae||BOLD:ACW2082
Cecidomyiidae|[6220]|GMAGI708-15|Diptera|Cecidomyiidae||BOLD:ACW7615
Cecidomyiidae|[6221]|GMARI959-14|Diptera|Cecidomyiidae||BOLD:ACN0633
Cecidomyiidae|[6222]|GMAGA593-15|Diptera|Cecidomyiidae||BOLD:ACM5456
Cecidomyiidae|[6223]|GMAGF325-15|Diptera|Cecidomyiidae||BOLD:ACW4057
Cecidomyiidae|[6224]|GMAGG2328-15|Diptera|Cecidomyiidae||BOLD:ACN0362
Cecidomyiidae|[6225]|GMAGD1133-15|Diptera|Cecidomyiidae||BOLD:ACN0815
Cecidomyiidae|[6226]|GMAGO329-15|Diptera|Cecidomyiidae||BOLD:ACX6228
Cecidomyiidae|[6227]|GMAGD992-15|Diptera|Cecidomyiidae||BOLD:ACW4533
Cecidomyiidae|[6228]|GMAGH737-15|Diptera|Cecidomyiidae||BOLD:ACW4470
Cecidomyiidae|[6229]|GMAGN1717-15|Diptera|Cecidomyiidae||BOLD:ACX5669
Cecidomyiidae|[6230]|GMAGG1762-15|Diptera|Cecidomyiidae||BOLD:ACW6640
Cecidomyiidae|[6231]|GMARI426-14|Diptera|Cecidomyiidae||BOLD:ACN0244
Cecidomyiidae|[6232]|GMAGQ388-15|Diptera|Cecidomyiidae||BOLD:ACX5077
Cecidomyiidae|[6233]|GMAGI881-15|Diptera|Cecidomyiidae||BOLD:ACW7454
Cecidomyiidae|[6234]|GMAGL2697-15|Diptera|Cecidomyiidae||BOLD:ACY8741
Cecidomyiidae|[6235]|GMAGC218-15|Diptera|Cecidomyiidae||BOLD:ACM5596
Cecidomyiidae|[6236]|GMARO496-14|Diptera|Cecidomyiidae||BOLD:ACO0474
Cecidomyiidae|[6237]|GMARR339-14|Diptera|Cecidomyiidae||BOLD:ACN8053
Cecidomyiidae|[6238]|GMAGH648-15|Diptera|Cecidomyiidae||BOLD:ACN4353
Cecidomyiidae|[6239]|GMARI479-14|Diptera|Cecidomyiidae||BOLD:ACN0004
Cecidomyiidae|[6240]|GMAGH672-15|Diptera|Cecidomyiidae||BOLD:ACW2792
Cecidomyiidae|[6241]|GMAGH1182-15|Diptera|Cecidomyiidae||BOLD:ACW3207
Cecidomyiidae|[6242]|GMARC746-14|Diptera|Cecidomyiidae||BOLD:ACM4782
Cecidomyiidae|[6243]|GMARJ905-14|Diptera|Cecidomyiidae||BOLD:ACN1765
Cecidomyiidae|[6244]|GMAGE2146-15|Diptera|Cecidomyiidae||BOLD:ACN7683
Cecidomyiidae|[6245]|GMAGK2038-15|Diptera|Cecidomyiidae||BOLD:ACN7900
Cecidomyiidae|[6246]|GMART1598-14|Diptera|Cecidomyiidae||BOLD:ACN9255
Cecidomyiidae|[6247]|GMARM323-14|Diptera|Cecidomyiidae||BOLD:ACN2770
Cecidomyiidae|[6248]|GMARL814-14|Diptera|Cecidomyiidae||BOLD:ACN2345
Cecidomyiidae|[6249]|GMAGQ1673-15|Diptera|Cecidomyiidae||BOLD:ACX4086
Cecidomyiidae|[6250]|GMARA314-14|Diptera|Cecidomyiidae||BOLD:ACM8481
Cecidomyiidae|[6251]|GMARM210-14|Diptera|Cecidomyiidae||BOLD:ACN2476
Cecidomyiidae|[6252]|GMAGE1977-15|Diptera|Cecidomyiidae||BOLD:ACW5963
Cecidomyiidae|[6253]|GMAGE1095-15|Diptera|Cecidomyiidae||BOLD:ACW2272
Cecidomyiidae|[6254]|GMARA1396-14|Diptera|Cecidomyiidae||BOLD:ACM8825
Cecidomyiidae|[6255]|GMAGG1930-15|Diptera|Cecidomyiidae||BOLD:ACN1360
Cecidomyiidae|[6256]|GMAGB598-15|Diptera|Cecidomyiidae||BOLD:ACW4212
Cecidomyiidae|[6257]|GMARM484-14|Diptera|Cecidomyiidae||BOLD:ACN2771
Cecidomyiidae|[6258]|GMARM1160-14|Diptera|Cecidomyiidae||BOLD:ACO0305
Cecidomyiidae|[6259]|GMART588-14|Diptera|Cecidomyiidae||BOLD:ACN9854
Cecidomyiidae|[6260]|GMARL1002-14|Diptera|Cecidomyiidae||BOLD:ACN2324
Cecidomyiidae|[6261]|GMART1243-14|Diptera|Cecidomyiidae||BOLD:ACO0673
Cecidomyiidae|[6262]|GMARB1223-14|Diptera|Cecidomyiidae||BOLD:ACM5437
Cecidomyiidae|[6263]|GMARD219-14|Diptera|Cecidomyiidae||BOLD:ACN1113
Cecidomyiidae|[6264]|GMAGD474-15|Diptera|Cecidomyiidae||BOLD:ACX2413
Cecidomyiidae|[6265]|GMARL728-14|Diptera|Cecidomyiidae||BOLD:ACN2658
Cecidomyiidae|[6266]|GMARL1174-14|Diptera|Cecidomyiidae||BOLD:ACN2674
Cecidomyiidae|[6267]|GMARK1754-14|Diptera|Cecidomyiidae||BOLD:ACN0528
Cecidomyiidae|[6268]|GMARL945-14|Diptera|Cecidomyiidae||BOLD:ACN2551
Cecidomyiidae|[6269]|GMAGI377-15|Diptera|Cecidomyiidae||BOLD:ACW4226
Cecidomyiidae|[6270]|GMARK1145-14|Diptera|Cecidomyiidae||BOLD:ACN1267
Asteromyia|[6271]|GMAGP1069-15|Diptera|Cecidomyiidae|Cecidomyiinae|BOLD:ACX6118
Cecidomyiidae|[6272]|GMARB1344-14|Diptera|Cecidomyiidae||BOLD:ACM6209

Cecidomyiidae|[6270]|GMARK1143-14|Diptera|Cecidomyiidae||BOLD:ACN1267
Asteromyia|[6271]|GMAGP1069-15|Diptera|Cecidomyiidae|Cecidomyiinae|BOLD:ACX6118
Cecidomyiidae|[6272]|GMARB1344-14|Diptera|Cecidomyiidae||BOLD:ACM6209
Cecidomyiidae|[6273]|GMAGB517-15|Diptera|Cecidomyiidae||BOLD:ACM5540
Cecidomyiidae|[6274]|GMARB1116-14|Diptera|Cecidomyiidae||BOLD:ACM6413
Cecidomyiidae|[6275]|GMAGP845-15|Diptera|Cecidomyiidae||BOLD:ACX4893
Cecidomyiidae|[6276]|GMAGG2130-15|Diptera|Cecidomyiidae||BOLD:ACW5959
Cecidomyiidae|[6277]|GMARD189-14|Diptera|Cecidomyiidae||BOLD:ACN1657
Cecidomyiidae|[6278]|GMAGQ1784-15|Diptera|Cecidomyiidae||BOLD:ACN9110
Cecidomyiidae|[6279]|GMARB1111-14|Diptera|Cecidomyiidae||BOLD:ACM5217
Cecidomyiidae|[6280]|GMAGA636-15|Diptera|Cecidomyiidae||BOLD:ACX6571
Cecidomyiidae|[6281]|GMAGE137-15|Diptera|Cecidomyiidae||BOLD:ACN0926
Cecidomyiidae|[6282]|GMARD1167-14|Diptera|Cecidomyiidae||BOLD:ACM9297
Cecidomyiidae|[6283]|GMART1110-14|Diptera|Cecidomyiidae||BOLD:ACO0621
Cecidomyiidae|[6284]|GMARU541-14|Diptera|Cecidomyiidae||BOLD:ACO0684
Cecidomyiidae|[6285]|GMARA1248-14|Diptera|Cecidomyiidae||BOLD:ACM6303
Cecidomyiidae|[6286]|GMARB1295-14|Diptera|Cecidomyiidae||BOLD:ACM5591
Cecidomyiidae|[6287]|GMARN096-14|Diptera|Cecidomyiidae||BOLD:ACN3440
Cecidomyiidae|[6288]|GMAGD1109-15|Diptera|Cecidomyiidae||BOLD:ACW3391
Cecidomyiidae|[6289]|GMARD1481-14|Diptera|Cecidomyiidae||BOLD:ACN1375
Cecidomyiidae|[6290]|GMAGE1890-15|Diptera|Cecidomyiidae||BOLD:ACW7285
Cecidomyiidae|[6291]|GMARP173-14|Diptera|Cecidomyiidae||BOLD:ACN9148
Cecidomyiidae|[6292]|GMARA1266-14|Diptera|Cecidomyiidae||BOLD:ACM8926
Cecidomyiidae|[6293]|GMAGQ831-15|Diptera|Cecidomyiidae||BOLD:ACX4221
Cecidomyiidae|[6294]|GMARJ1898-14|Diptera|Cecidomyiidae||BOLD:ACN0811
Cecidomyiidae|[6295]|GMARK1981-14|Diptera|Cecidomyiidae||BOLD:ACM9944
Cecidomyiidae|[6296]|GMARQ316-14|Diptera|Cecidomyiidae||BOLD:ACX3942
Cecidomyiidae|[6297]|GMAGF497-15|Diptera|Cecidomyiidae||BOLD:ACW2816
Cecidomyiidae|[6298]|GMARA1571-14|Diptera|Cecidomyiidae||BOLD:ACM5740
Cecidomyiidae|[6299]|GMARL1077-14|Diptera|Cecidomyiidae||BOLD:ACN2282
Cecidomyiidae|[6300]|GMARP293-14|Diptera|Cecidomyiidae||BOLD:ACO0536
Cecidomyiidae|[6301]|GMARR653-14|Diptera|Cecidomyiidae||BOLD:ACN7433
Cecidomyiidae|[6302]|GMAGS872-15|Diptera|Cecidomyiidae||BOLD:ACX5671
Cecidomyiidae|[6303]|GMAGD1320-15|Diptera|Cecidomyiidae||BOLD:ACM8718
Cecidomyiidae|[6304]|GMARS266-14|Diptera|Cecidomyiidae||BOLD:ACN7923
Cecidomyiidae|[6305]|GMART977-14|Diptera|Cecidomyiidae||BOLD:ACN9420
Cecidomyiidae|[6306]|GMARL359-14|Diptera|Cecidomyiidae||BOLD:ACN6467
Cecidomyiidae|[6307]|GMAGE2486-15|Diptera|Cecidomyiidae||BOLD:ACM7498
Cecidomyiidae|[6308]|GMARA262-14|Diptera|Cecidomyiidae||BOLD:ACM8370
Cecidomyiidae|[6309]|GMAGG2038-15|Diptera|Cecidomyiidae||BOLD:ACM7873
Cecidomyiidae|[6310]|GMARH1352-14|Diptera|Cecidomyiidae||BOLD:ACN0011
Cecidomyiidae|[6311]|GMAGD2215-15|Diptera|Cecidomyiidae||BOLD:ACM8676
Cecidomyiidae|[6312]|GMAGS739-15|Diptera|Cecidomyiidae||BOLD:ACX4179
Cecidomyiidae|[6313]|GMARD924-14|Diptera|Cecidomyiidae||BOLD:ACM9346
Cecidomyiidae|[6314]|GMARR1053-14|Diptera|Cecidomyiidae||BOLD:ACN8687
Cecidomyiidae|[6315]|GMARA1815-14|Diptera|Cecidomyiidae||BOLD:ACM5473
Cecidomyiidae|[6316]|GMARG500-14|Diptera|Cecidomyiidae||BOLD:ACN0512
Cecidomyiidae|[6317]|GMARA286-14|Diptera|Cecidomyiidae||BOLD:ACM7513
Cecidomyiidae|[6318]|GMAGI900-15|Diptera|Cecidomyiidae||BOLD:ACN9989
Cecidomyiidae|[6319]|GMARC577-14|Diptera|Cecidomyiidae||BOLD:ACM5863
Cecidomyiidae|[6320]|GMARF573-14|Diptera|Cecidomyiidae||BOLD:ACM8646
Cecidomyiidae|[6321]|GMAGV969-15|Diptera|Cecidomyiidae||BOLD:ADB9434
Cecidomyiidae|[6322]|GMAGB1134-15|Diptera|Cecidomyiidae||BOLD:ACW4869
Cecidomyiidae|[6323]|GMARB813-14|Diptera|Cecidomyiidae||BOLD:ACM5508
Cecidomyiidae|[6324]|GMARC258-14|Diptera|Cecidomyiidae||BOLD:ADC9288
Cecidomyiidae|[6325]|GMART499-14|Diptera|Cecidomyiidae||BOLD:ACO0022
Cecidomyiidae|[6326]|GMART258-14|Diptera|Cecidomyiidae||BOLD:ACN9037
Cecidomyiidae|[6327]|GMARP849-14|Diptera|Cecidomyiidae||BOLD:ACO3515
Cecidomyiidae|[6328]|GMAGA476-15|Diptera|Cecidomyiidae||BOLD:ACV2529
Cecidomyiidae|[6329]|GMARI2038-14|Diptera|Cecidomyiidae||BOLD:ACX3793
Cecidomyiidae|[6330]|GMAGM1948-15|Diptera|Cecidomyiidae||BOLD:ACN0802
Cecidomyiidae|[6331]|GMART818-14|Diptera|Cecidomyiidae||BOLD:ACN9730
Cecidomyiidae|[6332]|GMARD1539-14|Diptera|Cecidomyiidae||BOLD:ACN1586
Cecidomyiinae|[6333]|GMAGB1100-15|Diptera|Cecidomyiidae|Cecidomyiinae|BOLD:ACW0494
Cecidomyiinae|[6334]|GMAGD1732-15|Diptera|Cecidomyiidae|Cecidomyiinae|BOLD:ACW5764
Cecidomyiidae|[6335]|GMAGA646-15|Diptera|Cecidomyiidae||BOLD:ACM5660
Cecidomyiidae|[6336]|GMAGB435-15|Diptera|Cecidomyiidae||BOLD:ACV2516
Cecidomyiidae|[6337]|GMAGA456-15|Diptera|Cecidomyiidae||BOLD:ACV2517
Cecidomyiidae|[6338]|GMARA1356-14|Diptera|Cecidomyiidae||BOLD:ACU3526
Cecidomyiidae|[6339]|GMAGB606-15|Diptera|Cecidomyiidae||BOLD:ACW3297
Cecidomyiidae|[6340]|GMARB858-14|Diptera|Cecidomyiidae||BOLD:ACM6151
Cecidomyiidae|[6341]|GMARD961-14|Diptera|Cecidomyiidae||BOLD:ACM9132
Cecidomyiidae|[6342]|GMARA1313-14|Diptera|Cecidomyiidae||BOLD:ACM4741
Cecidomyiidae|[6343]|GMARO880-14|Diptera|Cecidomyiidae||BOLD:ACN7665
Cecidomyiidae|[6344]|GMARN1074-14|Diptera|Cecidomyiidae||BOLD:ACN9469
Cecidomyiidae|[6345]|GMARR434-14|Diptera|Cecidomyiidae||BOLD:ACN7680
Cecidomyiidae|[6346]|GMAGB846-15|Diptera|Cecidomyiidae||BOLD:ACN9712
Cecidomyiinae|[6347]|GMAGV963-15|Diptera|Cecidomyiidae|Cecidomyiinae|BOLD:ACX4098
Cecidomyiidae|[6348]|GMARA1770-14|Diptera|Cecidomyiidae||BOLD:ACM5891
Cecidomyiidae|[6349]|GMARB918-14|Diptera|Cecidomyiidae||BOLD:ACM5546
Cecidomyiidae|[6350]|GMAGE2120-15|Diptera|Cecidomyiidae||BOLD:ACN7709
Cecidomyiidae|[6351]|GMARA1533-14|Diptera|Cecidomyiidae||BOLD:ACM4869
Cecidomyiidae|[6352]|GMAGB1439-15|Diptera|Cecidomyiidae||BOLD:ACM5652
Cecidomyiidae|[6353]|GMAGJ949-15|Diptera|Cecidomyiidae||BOLD:ACW8989
Cecidomyiidae|[6354]|GMAGB1398-15|Diptera|Cecidomyiidae||BOLD:ACW3894
Cecidomyiidae|[6355]|GMARB1624-14|Diptera|Cecidomyiidae||BOLD:ACM6234
Cecidomyiidae|[6356]|GMAGP1077-15|Diptera|Cecidomyiidae||BOLD:ACX5362
Cecidomyiidae|[6357]|GMAGJ1746-15|Diptera|Cecidomyiidae||BOLD:ACX5241
Cecidomyiidae|[6358]|GMART1256-14|Diptera|Cecidomyiidae||BOLD:ACO0600
Cecidomyiidae|[6359]|GMARA1121-14|Diptera|Cecidomyiidae||BOLD:ACM9789
Cecidomyiidae|[6360]|GMAGD2337-15|Diptera|Cecidomyiidae||BOLD:ACW3509
Cecidomyiidae|[6361]|GMARN1023-14|Diptera|Cecidomyiidae||BOLD:ACX6829
Cecidomyiidae|[6362]|GMAGD2163-15|Diptera|Cecidomyiidae||BOLD:ACW2314
Cecidomyiidae|[6363]|GMARM601-14|Diptera|Cecidomyiidae||BOLD:ACN9423
Cecidomyiidae|[6364]|GMAGB1504-15|Diptera|Cecidomyiidae||BOLD:ACK1800
Cecidomyiidae|[6365]|GMARF229-14|Diptera|Cecidomyiidae||BOLD:ACM9569
Cecidomyiidae|[6366]|GMAGT775-15|Diptera|Cecidomyiidae||BOLD:ACX4820
Cecidomyiidae|[6367]|GMAGN2013-15|Diptera|Cecidomyiidae||BOLD:ACX1647
Cecidomyiidae|[6368]|GMAGN2151-15|Diptera|Cecidomyiidae||BOLD:ACX1620
Cecidomyiidae|[6369]|GMAGE1991-15|Diptera|Cecidomyiidae||BOLD:ACW6968
Cecidomyiidae|[6370]|GMARC207-14|Diptera|Cecidomyiidae||BOLD:ACM7293
Cecidomyiidae|[6371]|GMARE607-14|Diptera|Cecidomyiidae||BOLD:ACM8161
Cecidomyiidae|[6372]|GMAGN1727-15|Diptera|Cecidomyiidae||BOLD:ACX4132

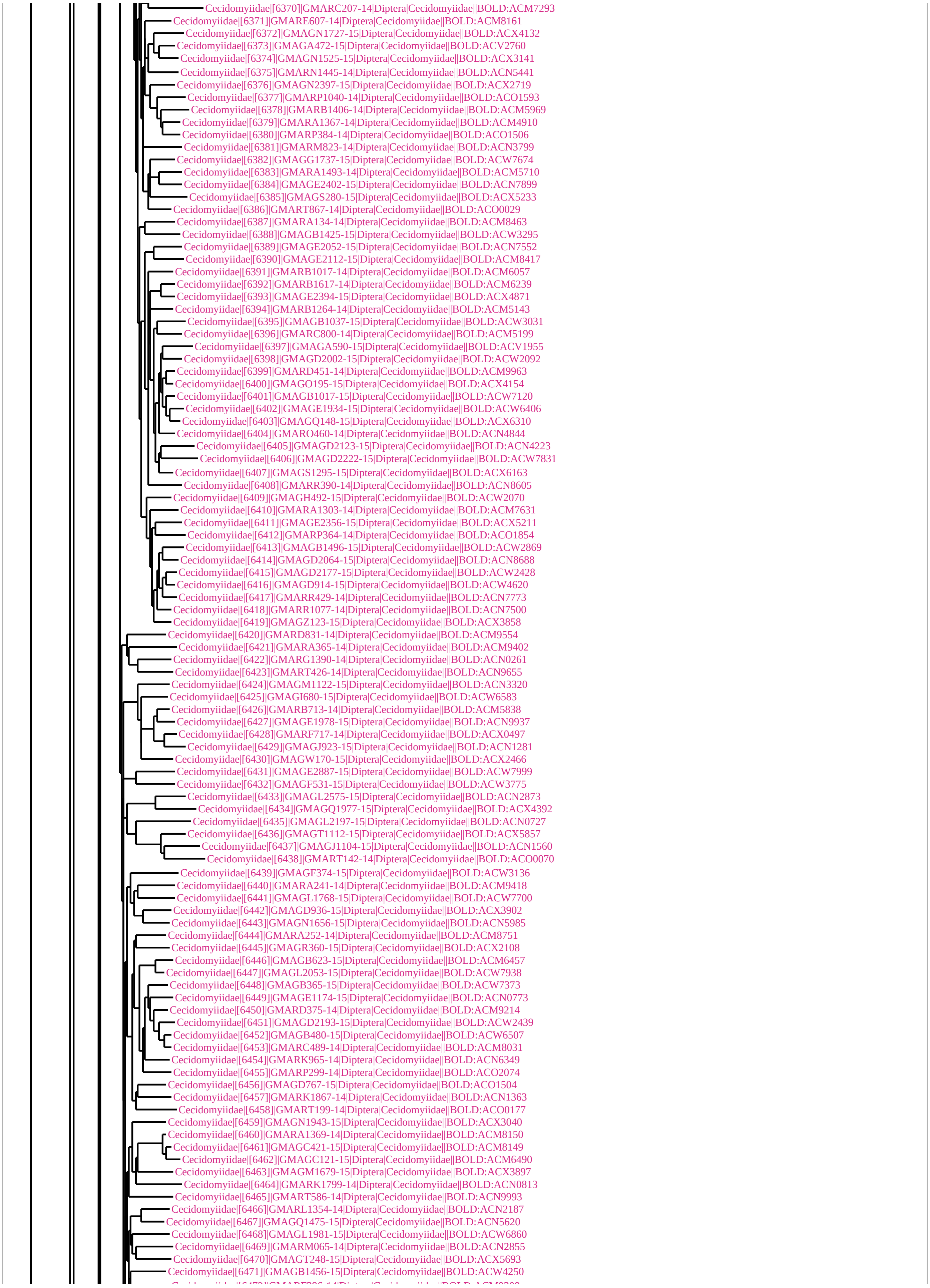
Cecidomyiidae|[6370]|GMARC207-14|Diptera|Cecidomyiidae||BOLD:ACM7293
Cecidomyiidae|[6371]|GMARE607-14|Diptera|Cecidomyiidae||BOLD:ACM8161
Cecidomyiidae|[6372]|GMAGN1727-15|Diptera|Cecidomyiidae||BOLD:ACX4132
Cecidomyiidae|[6373]|GMAGA472-15|Diptera|Cecidomyiidae||BOLD:ACV2760
Cecidomyiidae|[6374]|GMAGN1525-15|Diptera|Cecidomyiidae||BOLD:ACX3141
Cecidomyiidae|[6375]|GMARN1445-14|Diptera|Cecidomyiidae||BOLD:ACN5441
Cecidomyiidae|[6376]|GMAGN2397-15|Diptera|Cecidomyiidae||BOLD:ACX2719
Cecidomyiidae|[6377]|GMARP1040-14|Diptera|Cecidomyiidae||BOLD:ACO1593
Cecidomyiidae|[6378]|GMARB1406-14|Diptera|Cecidomyiidae||BOLD:ACM5969
Cecidomyiidae|[6379]|GMARA1367-14|Diptera|Cecidomyiidae||BOLD:ACM4910
Cecidomyiidae|[6380]|GMARP384-14|Diptera|Cecidomyiidae||BOLD:ACO1506
Cecidomyiidae|[6381]|GMARM823-14|Diptera|Cecidomyiidae||BOLD:ACN3799
Cecidomyiidae|[6382]|GMAGG1737-15|Diptera|Cecidomyiidae||BOLD:ACW7674
Cecidomyiidae|[6383]|GMARA1493-14|Diptera|Cecidomyiidae||BOLD:ACM5710
Cecidomyiidae|[6384]|GMAGE2402-15|Diptera|Cecidomyiidae||BOLD:ACN7899
Cecidomyiidae|[6385]|GMAGS280-15|Diptera|Cecidomyiidae||BOLD:ACX5233
Cecidomyiidae|[6386]|GMART867-14|Diptera|Cecidomyiidae||BOLD:ACO0029
Cecidomyiidae|[6387]|GMARA134-14|Diptera|Cecidomyiidae||BOLD:ACM8463
Cecidomyiidae|[6388]|GMAGB1425-15|Diptera|Cecidomyiidae||BOLD:ACW3295
Cecidomyiidae|[6389]|GMAGE2052-15|Diptera|Cecidomyiidae||BOLD:ACN7552
Cecidomyiidae|[6390]|GMAGE2112-15|Diptera|Cecidomyiidae||BOLD:ACM8417
Cecidomyiidae|[6391]|GMARB1017-14|Diptera|Cecidomyiidae||BOLD:ACM6057
Cecidomyiidae|[6392]|GMARB1617-14|Diptera|Cecidomyiidae||BOLD:ACM6239
Cecidomyiidae|[6393]|GMAGE2394-15|Diptera|Cecidomyiidae||BOLD:ACX4871
Cecidomyiidae|[6394]|GMARB1264-14|Diptera|Cecidomyiidae||BOLD:ACM5143
Cecidomyiidae|[6395]|GMAGB1037-15|Diptera|Cecidomyiidae||BOLD:ACW3031
Cecidomyiidae|[6396]|GMARC800-14|Diptera|Cecidomyiidae||BOLD:ACM5199
Cecidomyiidae|[6397]|GMAGA590-15|Diptera|Cecidomyiidae||BOLD:ACV1955
Cecidomyiidae|[6398]|GMAGD2002-15|Diptera|Cecidomyiidae||BOLD:ACW2092
Cecidomyiidae|[6399]|GMARD451-14|Diptera|Cecidomyiidae||BOLD:ACM9963
Cecidomyiidae|[6400]|GMAGO195-15|Diptera|Cecidomyiidae||BOLD:ACX4154
Cecidomyiidae|[6401]|GMAGB1017-15|Diptera|Cecidomyiidae||BOLD:ACW7120
Cecidomyiidae|[6402]|GMAGE1934-15|Diptera|Cecidomyiidae||BOLD:ACW6406
Cecidomyiidae|[6403]|GMAGQ148-15|Diptera|Cecidomyiidae||BOLD:ACX6310
Cecidomyiidae|[6404]|GMARO460-14|Diptera|Cecidomyiidae||BOLD:ACN4844
Cecidomyiidae|[6405]|GMAGD2123-15|Diptera|Cecidomyiidae||BOLD:ACN4223
Cecidomyiidae|[6406]|GMAGD2222-15|Diptera|Cecidomyiidae||BOLD:ACW7831
Cecidomyiidae|[6407]|GMAGS1295-15|Diptera|Cecidomyiidae||BOLD:ACX6163
Cecidomyiidae|[6408]|GMARR390-14|Diptera|Cecidomyiidae||BOLD:ACN8605
Cecidomyiidae|[6409]|GMAGH492-15|Diptera|Cecidomyiidae||BOLD:ACW2070
Cecidomyiidae|[6410]|GMARA1303-14|Diptera|Cecidomyiidae||BOLD:ACM7631
Cecidomyiidae|[6411]|GMAGE2356-15|Diptera|Cecidomyiidae||BOLD:ACX5211
Cecidomyiidae|[6412]|GMARP364-14|Diptera|Cecidomyiidae||BOLD:ACO1854
Cecidomyiidae|[6413]|GMAGB1496-15|Diptera|Cecidomyiidae||BOLD:ACW2869
Cecidomyiidae|[6414]|GMAGD2064-15|Diptera|Cecidomyiidae||BOLD:ACN8688
Cecidomyiidae|[6415]|GMAGD2177-15|Diptera|Cecidomyiidae||BOLD:ACW2428
Cecidomyiidae|[6416]|GMAGD914-15|Diptera|Cecidomyiidae||BOLD:ACW4620
Cecidomyiidae|[6417]|GMARR429-14|Diptera|Cecidomyiidae||BOLD:ACN7773
Cecidomyiidae|[6418]|GMARR1077-14|Diptera|Cecidomyiidae||BOLD:ACN7500
Cecidomyiidae|[6419]|GMAGZ123-15|Diptera|Cecidomyiidae||BOLD:ACX3858
Cecidomyiidae|[6420]|GMARD831-14|Diptera|Cecidomyiidae||BOLD:ACM9554
Cecidomyiidae|[6421]|GMARA365-14|Diptera|Cecidomyiidae||BOLD:ACM9402
Cecidomyiidae|[6422]|GMARG1390-14|Diptera|Cecidomyiidae||BOLD:ACN0261
Cecidomyiidae|[6423]|GMART426-14|Diptera|Cecidomyiidae||BOLD:ACN9655
Cecidomyiidae|[6424]|GMAGM1122-15|Diptera|Cecidomyiidae||BOLD:ACN3320
Cecidomyiidae|[6425]|GMAGI680-15|Diptera|Cecidomyiidae||BOLD:ACW6583
Cecidomyiidae|[6426]|GMARB713-14|Diptera|Cecidomyiidae||BOLD:ACM5838
Cecidomyiidae|[6427]|GMAGE1978-15|Diptera|Cecidomyiidae||BOLD:ACN9937
Cecidomyiidae|[6428]|GMARF717-14|Diptera|Cecidomyiidae||BOLD:ACX0497
Cecidomyiidae|[6429]|GMAGJ923-15|Diptera|Cecidomyiidae||BOLD:ACN1281
Cecidomyiidae|[6430]|GMAGW170-15|Diptera|Cecidomyiidae||BOLD:ACX2466
Cecidomyiidae|[6431]|GMAGE2887-15|Diptera|Cecidomyiidae||BOLD:ACW7999
Cecidomyiidae|[6432]|GMAGF531-15|Diptera|Cecidomyiidae||BOLD:ACW3775
Cecidomyiidae|[6433]|GMAGL2575-15|Diptera|Cecidomyiidae||BOLD:ACN2873
Cecidomyiidae|[6434]|GMAGQ1977-15|Diptera|Cecidomyiidae||BOLD:ACX4392
Cecidomyiidae|[6435]|GMAGL2197-15|Diptera|Cecidomyiidae||BOLD:ACN0727
Cecidomyiidae|[6436]|GMAGT1112-15|Diptera|Cecidomyiidae||BOLD:ACX5857
Cecidomyiidae|[6437]|GMAGJ1104-15|Diptera|Cecidomyiidae||BOLD:ACN1560
Cecidomyiidae|[6438]|GMART142-14|Diptera|Cecidomyiidae||BOLD:ACO0070
Cecidomyiidae|[6439]|GMAGF374-15|Diptera|Cecidomyiidae||BOLD:ACW3136
Cecidomyiidae|[6440]|GMARA241-14|Diptera|Cecidomyiidae||BOLD:ACM9418
Cecidomyiidae|[6441]|GMAGL1768-15|Diptera|Cecidomyiidae||BOLD:ACW7700
Cecidomyiidae|[6442]|GMAGD936-15|Diptera|Cecidomyiidae||BOLD:ACX3902
Cecidomyiidae|[6443]|GMAGN1656-15|Diptera|Cecidomyiidae||BOLD:ACN5985
Cecidomyiidae|[6444]|GMARA252-14|Diptera|Cecidomyiidae||BOLD:ACM8751
Cecidomyiidae|[6445]|GMAGR360-15|Diptera|Cecidomyiidae||BOLD:ACX2108
Cecidomyiidae|[6446]|GMAGB623-15|Diptera|Cecidomyiidae||BOLD:ACM6457
Cecidomyiidae|[6447]|GMAGL2053-15|Diptera|Cecidomyiidae||BOLD:ACW7938
Cecidomyiidae|[6448]|GMAGB365-15|Diptera|Cecidomyiidae||BOLD:ACW7373
Cecidomyiidae|[6449]|GMAGE1174-15|Diptera|Cecidomyiidae||BOLD:ACN0773
Cecidomyiidae|[6450]|GMARD375-14|Diptera|Cecidomyiidae||BOLD:ACM9214
Cecidomyiidae|[6451]|GMAGD2193-15|Diptera|Cecidomyiidae||BOLD:ACW2439
Cecidomyiidae|[6452]|GMAGB480-15|Diptera|Cecidomyiidae||BOLD:ACW6507
Cecidomyiidae|[6453]|GMARC489-14|Diptera|Cecidomyiidae||BOLD:ACM8031
Cecidomyiidae|[6454]|GMARK965-14|Diptera|Cecidomyiidae||BOLD:ACN6349
Cecidomyiidae|[6455]|GMARP299-14|Diptera|Cecidomyiidae||BOLD:ACO2074
Cecidomyiidae|[6456]|GMAGD767-15|Diptera|Cecidomyiidae||BOLD:ACO1504
Cecidomyiidae|[6457]|GMARK1867-14|Diptera|Cecidomyiidae||BOLD:ACN1363
Cecidomyiidae|[6458]|GMART199-14|Diptera|Cecidomyiidae||BOLD:ACO0177
Cecidomyiidae|[6459]|GMAGN1943-15|Diptera|Cecidomyiidae||BOLD:ACX3040
Cecidomyiidae|[6460]|GMARA1369-14|Diptera|Cecidomyiidae||BOLD:ACM8150
Cecidomyiidae|[6461]|GMAGC421-15|Diptera|Cecidomyiidae||BOLD:ACM8149
Cecidomyiidae|[6462]|GMAGC121-15|Diptera|Cecidomyiidae||BOLD:ACM6490
Cecidomyiidae|[6463]|GMAGM1679-15|Diptera|Cecidomyiidae||BOLD:ACX3897
Cecidomyiidae|[6464]|GMARK1799-14|Diptera|Cecidomyiidae||BOLD:ACN0813
Cecidomyiidae|[6465]|GMART586-14|Diptera|Cecidomyiidae||BOLD:ACN9993
Cecidomyiidae|[6466]|GMARL1354-14|Diptera|Cecidomyiidae||BOLD:ACN2187
Cecidomyiidae|[6467]|GMAGQ1475-15|Diptera|Cecidomyiidae||BOLD:ACN5620
Cecidomyiidae|[6468]|GMAGL1981-15|Diptera|Cecidomyiidae||BOLD:ACW6860
Cecidomyiidae|[6469]|GMARM065-14|Diptera|Cecidomyiidae||BOLD:ACN2855
Cecidomyiidae|[6470]|GMAGT248-15|Diptera|Cecidomyiidae||BOLD:ACX5693
Cecidomyiidae|[6471]|GMAGB1456-15|Diptera|Cecidomyiidae||BOLD:ACW4250

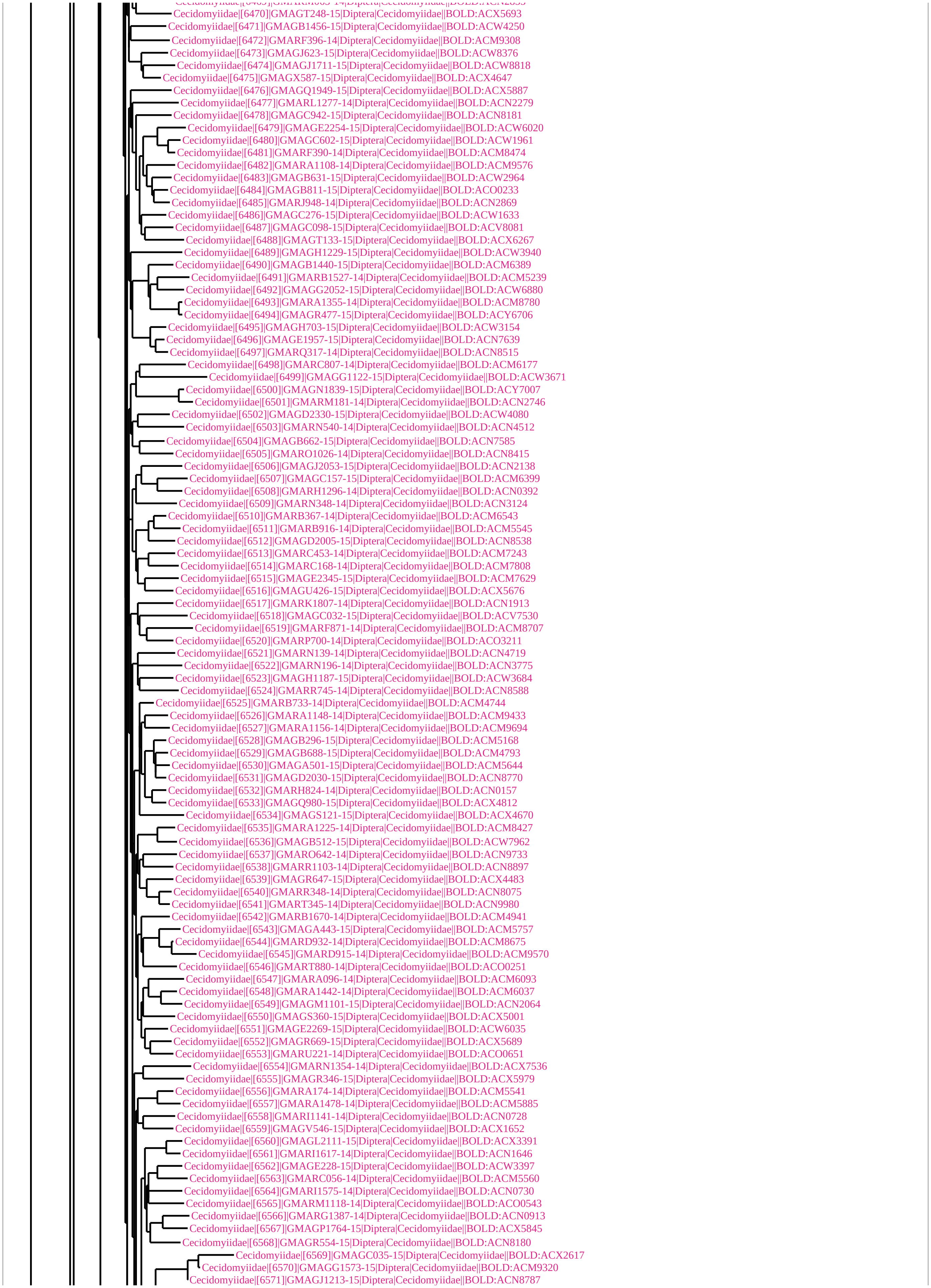
[illegible]
Cecidomyiidae|[6470]|GMAGT248-15|Diptera|Cecidomyiidae||BOLD:ACX5693
Cecidomyiidae|[6471]|GMAGB1456-15|Diptera|Cecidomyiidae||BOLD:ACW4250
Cecidomyiidae|[6472]|GMARF396-14|Diptera|Cecidomyiidae||BOLD:ACM9308
Cecidomyiidae|[6473]|GMAGJ623-15|Diptera|Cecidomyiidae||BOLD:ACW8376
Cecidomyiidae|[6474]|GMAGJ1711-15|Diptera|Cecidomyiidae||BOLD:ACW8818
Cecidomyiidae|[6475]|GMAGX587-15|Diptera|Cecidomyiidae||BOLD:ACX4647
Cecidomyiidae|[6476]|GMAGQ1949-15|Diptera|Cecidomyiidae||BOLD:ACX5887
Cecidomyiidae|[6477]|GMARL1277-14|Diptera|Cecidomyiidae||BOLD:ACN2279
Cecidomyiidae|[6478]|GMAGC942-15|Diptera|Cecidomyiidae||BOLD:ACN8181
Cecidomyiidae|[6479]|GMAGE2254-15|Diptera|Cecidomyiidae||BOLD:ACW6020
Cecidomyiidae|[6480]|GMAGC602-15|Diptera|Cecidomyiidae||BOLD:ACW1961
Cecidomyiidae|[6481]|GMARF390-14|Diptera|Cecidomyiidae||BOLD:ACM8474
Cecidomyiidae|[6482]|GMARA1108-14|Diptera|Cecidomyiidae||BOLD:ACM9576
Cecidomyiidae|[6483]|GMAGB631-15|Diptera|Cecidomyiidae||BOLD:ACW2964
Cecidomyiidae|[6484]|GMAGB811-15|Diptera|Cecidomyiidae||BOLD:ACO0233
Cecidomyiidae|[6485]|GMARJ948-14|Diptera|Cecidomyiidae||BOLD:ACN2869
Cecidomyiidae|[6486]|GMAGC276-15|Diptera|Cecidomyiidae||BOLD:ACW1633
Cecidomyiidae|[6487]|GMAGC098-15|Diptera|Cecidomyiidae||BOLD:ACV8081
Cecidomyiidae|[6488]|GMAGT133-15|Diptera|Cecidomyiidae||BOLD:ACX6267
Cecidomyiidae|[6489]|GMAGH1229-15|Diptera|Cecidomyiidae||BOLD:ACW3940
Cecidomyiidae|[6490]|GMAGB1440-15|Diptera|Cecidomyiidae||BOLD:ACM6389
Cecidomyiidae|[6491]|GMARB1527-14|Diptera|Cecidomyiidae||BOLD:ACM5239
Cecidomyiidae|[6492]|GMAGG2052-15|Diptera|Cecidomyiidae||BOLD:ACW6880
Cecidomyiidae|[6493]|GMARA1355-14|Diptera|Cecidomyiidae||BOLD:ACM8780
Cecidomyiidae|[6494]|GMAGR477-15|Diptera|Cecidomyiidae||BOLD:ACY6706
Cecidomyiidae|[6495]|GMAGH703-15|Diptera|Cecidomyiidae||BOLD:ACW3154
Cecidomyiidae|[6496]|GMAGE1957-15|Diptera|Cecidomyiidae||BOLD:ACN7639
Cecidomyiidae|[6497]|GMARQ317-14|Diptera|Cecidomyiidae||BOLD:ACN8515
Cecidomyiidae|[6498]|GMARC807-14|Diptera|Cecidomyiidae||BOLD:ACM6177
Cecidomyiidae|[6499]|GMAGG1122-15|Diptera|Cecidomyiidae||BOLD:ACW3671
Cecidomyiidae|[6500]|GMAGN1839-15|Diptera|Cecidomyiidae||BOLD:ACY7007
Cecidomyiidae|[6501]|GMARM181-14|Diptera|Cecidomyiidae||BOLD:ACN2746
Cecidomyiidae|[6502]|GMAGD2330-15|Diptera|Cecidomyiidae||BOLD:ACW4080
Cecidomyiidae|[6503]|GMARN540-14|Diptera|Cecidomyiidae||BOLD:ACN4512
Cecidomyiidae|[6504]|GMAGB662-15|Diptera|Cecidomyiidae||BOLD:ACN7585
Cecidomyiidae|[6505]|GMARO1026-14|Diptera|Cecidomyiidae||BOLD:ACN8415
Cecidomyiidae|[6506]|GMAGJ2053-15|Diptera|Cecidomyiidae||BOLD:ACN2138
Cecidomyiidae|[6507]|GMAGC157-15|Diptera|Cecidomyiidae||BOLD:ACM6399
Cecidomyiidae|[6508]|GMARH1296-14|Diptera|Cecidomyiidae||BOLD:ACN0392
Cecidomyiidae|[6509]|GMARN348-14|Diptera|Cecidomyiidae||BOLD:ACN3124
Cecidomyiidae|[6510]|GMARB367-14|Diptera|Cecidomyiidae||BOLD:ACM6543
Cecidomyiidae|[6511]|GMARB916-14|Diptera|Cecidomyiidae||BOLD:ACM5545
Cecidomyiidae|[6512]|GMAGD2005-15|Diptera|Cecidomyiidae||BOLD:ACN8538
Cecidomyiidae|[6513]|GMARC453-14|Diptera|Cecidomyiidae||BOLD:ACM7243
Cecidomyiidae|[6514]|GMARC168-14|Diptera|Cecidomyiidae||BOLD:ACM7808
Cecidomyiidae|[6515]|GMAGE2345-15|Diptera|Cecidomyiidae||BOLD:ACM7629
Cecidomyiidae|[6516]|GMAGU426-15|Diptera|Cecidomyiidae||BOLD:ACX5676
Cecidomyiidae|[6517]|GMARK1807-14|Diptera|Cecidomyiidae||BOLD:ACN1913
Cecidomyiidae|[6518]|GMAGC032-15|Diptera|Cecidomyiidae||BOLD:ACV7530
Cecidomyiidae|[6519]|GMARF871-14|Diptera|Cecidomyiidae||BOLD:ACM8707
Cecidomyiidae|[6520]|GMARP700-14|Diptera|Cecidomyiidae||BOLD:ACO3211
Cecidomyiidae|[6521]|GMARN139-14|Diptera|Cecidomyiidae||BOLD:ACN4719
Cecidomyiidae|[6522]|GMARN196-14|Diptera|Cecidomyiidae||BOLD:ACN3775
Cecidomyiidae|[6523]|GMAGH1187-15|Diptera|Cecidomyiidae||BOLD:ACW3684
Cecidomyiidae|[6524]|GMARR745-14|Diptera|Cecidomyiidae||BOLD:ACN8588
Cecidomyiidae|[6525]|GMARB733-14|Diptera|Cecidomyiidae||BOLD:ACM4744
Cecidomyiidae|[6526]|GMARA1148-14|Diptera|Cecidomyiidae||BOLD:ACM9433
Cecidomyiidae|[6527]|GMARA1156-14|Diptera|Cecidomyiidae||BOLD:ACM9694
Cecidomyiidae|[6528]|GMAGB296-15|Diptera|Cecidomyiidae||BOLD:ACM5168
Cecidomyiidae|[6529]|GMAGB688-15|Diptera|Cecidomyiidae||BOLD:ACM4793
Cecidomyiidae|[6530]|GMAGA501-15|Diptera|Cecidomyiidae||BOLD:ACM5644
Cecidomyiidae|[6531]|GMAGD2030-15|Diptera|Cecidomyiidae||BOLD:ACN8770
Cecidomyiidae|[6532]|GMARH824-14|Diptera|Cecidomyiidae||BOLD:ACN0157
Cecidomyiidae|[6533]|GMAGQ980-15|Diptera|Cecidomyiidae||BOLD:ACX4812
Cecidomyiidae|[6534]|GMAGS121-15|Diptera|Cecidomyiidae||BOLD:ACX4670
Cecidomyiidae|[6535]|GMARA1225-14|Diptera|Cecidomyiidae||BOLD:ACM8427
Cecidomyiidae|[6536]|GMAGB512-15|Diptera|Cecidomyiidae||BOLD:ACW7962
Cecidomyiidae|[6537]|GMARO642-14|Diptera|Cecidomyiidae||BOLD:ACN9733
Cecidomyiidae|[6538]|GMARR1103-14|Diptera|Cecidomyiidae||BOLD:ACN8897
Cecidomyiidae|[6539]|GMAGR647-15|Diptera|Cecidomyiidae||BOLD:ACX4483
Cecidomyiidae|[6540]|GMARR348-14|Diptera|Cecidomyiidae||BOLD:ACN8075
Cecidomyiidae|[6541]|GMART345-14|Diptera|Cecidomyiidae||BOLD:ACN9980
Cecidomyiidae|[6542]|GMARB1670-14|Diptera|Cecidomyiidae||BOLD:ACM4941
Cecidomyiidae|[6543]|GMAGA443-15|Diptera|Cecidomyiidae||BOLD:ACM5757
Cecidomyiidae|[6544]|GMARD932-14|Diptera|Cecidomyiidae||BOLD:ACM8675
Cecidomyiidae|[6545]|GMARD915-14|Diptera|Cecidomyiidae||BOLD:ACM9570
Cecidomyiidae|[6546]|GMART880-14|Diptera|Cecidomyiidae||BOLD:ACO0251
Cecidomyiidae|[6547]|GMARA096-14|Diptera|Cecidomyiidae||BOLD:ACM6093
Cecidomyiidae|[6548]|GMARA1442-14|Diptera|Cecidomyiidae||BOLD:ACM6037
Cecidomyiidae|[6549]|GMAGM1101-15|Diptera|Cecidomyiidae||BOLD:ACN2064
Cecidomyiidae|[6550]|GMAGS360-15|Diptera|Cecidomyiidae||BOLD:ACX5001
Cecidomyiidae|[6551]|GMAGE2269-15|Diptera|Cecidomyiidae||BOLD:ACW6035
Cecidomyiidae|[6552]|GMAGR669-15|Diptera|Cecidomyiidae||BOLD:ACX5689
Cecidomyiidae|[6553]|GMARU221-14|Diptera|Cecidomyiidae||BOLD:ACO0651
Cecidomyiidae|[6554]|GMARN1354-14|Diptera|Cecidomyiidae||BOLD:ACX7536
Cecidomyiidae|[6555]|GMAGR346-15|Diptera|Cecidomyiidae||BOLD:ACX5979
Cecidomyiidae|[6556]|GMARA174-14|Diptera|Cecidomyiidae||BOLD:ACM5541
Cecidomyiidae|[6557]|GMARA1478-14|Diptera|Cecidomyiidae||BOLD:ACM5885
Cecidomyiidae|[6558]|GMARI1141-14|Diptera|Cecidomyiidae||BOLD:ACN0728
Cecidomyiidae|[6559]|GMAGV546-15|Diptera|Cecidomyiidae||BOLD:ACX1652
Cecidomyiidae|[6560]|GMAGL2111-15|Diptera|Cecidomyiidae||BOLD:ACX3391
Cecidomyiidae|[6561]|GMARI1617-14|Diptera|Cecidomyiidae||BOLD:ACN1646
Cecidomyiidae|[6562]|GMAGE228-15|Diptera|Cecidomyiidae||BOLD:ACW3397
Cecidomyiidae|[6563]|GMARC056-14|Diptera|Cecidomyiidae||BOLD:ACM5560
Cecidomyiidae|[6564]|GMARI1575-14|Diptera|Cecidomyiidae||BOLD:ACN0730
Cecidomyiidae|[6565]|GMARM1118-14|Diptera|Cecidomyiidae||BOLD:ACO0543
Cecidomyiidae|[6566]|GMARG1387-14|Diptera|Cecidomyiidae||BOLD:ACN0913
Cecidomyiidae|[6567]|GMAGP1764-15|Diptera|Cecidomyiidae||BOLD:ACX5845
Cecidomyiidae|[6568]|GMAGR554-15|Diptera|Cecidomyiidae||BOLD:ACN8180
Cecidomyiidae|[6569]|GMAGC035-15|Diptera|Cecidomyiidae||BOLD:ACX2617
Cecidomyiidae|[6570]|GMAGG1573-15|Diptera|Cecidomyiidae||BOLD:ACM9320
Cecidomyiidae|[6571]|GMAGJ1213-15|Diptera|Cecidomyiidae||BOLD:ACN8787

Cecidomyiidae|[6569]|GMAGC035-15|Diptera|Cecidomyiidae||BOLD:ACX2617
Cecidomyiidae|[6570]|GMAGG1573-15|Diptera|Cecidomyiidae||BOLD:ACM9320
Cecidomyiidae|[6571]|GMAGJ1213-15|Diptera|Cecidomyiidae||BOLD:ACN8787
Cecidomyiidae|[6572]|GMARB632-14|Diptera|Cecidomyiidae||BOLD:ACM6164
Cecidomyiidae|[6573]|GMAGD194-15|Diptera|Cecidomyiidae||BOLD:ACW5038
Cecidomyiidae|[6574]|GMARA297-14|Diptera|Cecidomyiidae||BOLD:ACM5240
Cecidomyiidae|[6575]|GMAGA673-15|Diptera|Cecidomyiidae||BOLD:ACW4378
Cecidomyiidae|[6576]|GMAGF255-15|Diptera|Cecidomyiidae||BOLD:ACW4145
Cecidomyiidae|[6577]|GMARA1298-14|Diptera|Cecidomyiidae||BOLD:ACM5812
Cecidomyiidae|[6578]|GMARG1393-14|Diptera|Cecidomyiidae||BOLD:ACN1474
Cecidomyiidae|[6579]|GMARK1934-14|Diptera|Cecidomyiidae||BOLD:ACN0218
Cecidomyiidae|[6580]|GMAGO204-15|Diptera|Cecidomyiidae||BOLD:ACX5923
Cecidomyiidae|[6581]|GMAGE1917-15|Diptera|Cecidomyiidae||BOLD:ACW2874
Cecidomyiidae|[6582]|GMAGD1118-15|Diptera|Cecidomyiidae||BOLD:ACW4619
Cecidomyiidae|[6583]|GMARG1568-14|Diptera|Cecidomyiidae||BOLD:ACN0428
Cecidomyiidae|[6584]|GMAGA569-15|Diptera|Cecidomyiidae||BOLD:ACM5039
Cecidomyiidae|[6585]|GMAGN1768-15|Diptera|Cecidomyiidae||BOLD:ACN0305
Cecidomyiidae|[6586]|GMARK925-14|Diptera|Cecidomyiidae||BOLD:ACN2575
Cecidomyiidae|[6587]|GMAGP1703-15|Diptera|Cecidomyiidae||BOLD:ACN6803
Cecidomyiidae|[6588]|GMARO996-14|Diptera|Cecidomyiidae||BOLD:ACN8579
Cecidomyiidae|[6589]|GMARM1052-14|Diptera|Cecidomyiidae||BOLD:ACN9721
Cecidomyiidae|[6590]|GMARN421-14|Diptera|Cecidomyiidae||BOLD:ACN4681
Cecidomyiidae|[6591]|GMART835-14|Diptera|Cecidomyiidae||BOLD:ACN9490
Cecidomyiidae|[6592]|GMARU515-14|Diptera|Cecidomyiidae||BOLD:ACX6597
Cecidomyiidae|[6593]|GMAGB582-15|Diptera|Cecidomyiidae||BOLD:ACW2296
Cecidomyiidae|[6594]|GMARB1011-14|Diptera|Cecidomyiidae||BOLD:ACM6403
Cecidomyiidae|[6595]|GMAGD979-15|Diptera|Cecidomyiidae||BOLD:ACW2953
Cecidomyiidae|[6596]|GMAGD2286-15|Diptera|Cecidomyiidae||BOLD:ACW6617
Cecidomyiidae|[6597]|GMAGJ1831-15|Diptera|Cecidomyiidae||BOLD:ACN0484
Cecidomyiidae|[6598]|GMARE349-14|Diptera|Cecidomyiidae||BOLD:ACN0760
Cecidomyiidae|[6599]|GMAGI744-15|Diptera|Cecidomyiidae||BOLD:ADB1867
Cecidomyiidae|[6600]|GMAGM1308-15|Diptera|Cecidomyiidae||BOLD:ACX3389
Cecidomyiidae|[6601]|GMARB886-14|Diptera|Cecidomyiidae||BOLD:ACM5284
Cecidomyiidae|[6602]|GMAGC1486-15|Diptera|Cecidomyiidae||BOLD:ACM7350
Cecidomyiidae|[6603]|GMARO627-14|Diptera|Cecidomyiidae||BOLD:ACO0275
Cecidomyiidae|[6604]|GMAGB1464-15|Diptera|Cecidomyiidae||BOLD:ACW3331
Cecidomyiidae|[6605]|GMAGC094-15|Diptera|Cecidomyiidae||BOLD:ACV6288
Cecidomyiidae|[6606]|GMARF077-14|Diptera|Cecidomyiidae||BOLD:ACN1249
Cecidomyiidae|[6607]|GMARF538-14|Diptera|Cecidomyiidae||BOLD:ACM8855
Cecidomyiidae|[6608]|GMAGF455-15|Diptera|Cecidomyiidae||BOLD:ACW3214
Cecidomyiidae|[6609]|GMARH052-14|Diptera|Cecidomyiidae||BOLD:ACN1825
Cecidomyiidae|[6610]|GMAGD1362-15|Diptera|Cecidomyiidae||BOLD:ACM5627
Cecidomyiidae|[6611]|GMARD483-14|Diptera|Cecidomyiidae||BOLD:ACM4839
Cecidomyiidae|[6612]|GMAGJ969-15|Diptera|Cecidomyiidae||BOLD:ACX0165
Cecidomyiidae|[6613]|GMARP689-14|Diptera|Cecidomyiidae||BOLD:ACO3680
Cecidomyiidae|[6614]|GMARA1234-14|Diptera|Cecidomyiidae||BOLD:ACM9514
Cecidomyiidae|[6615]|GMAGG1715-15|Diptera|Cecidomyiidae||BOLD:ACN0242
Cecidomyiidae|[6616]|GMAGB1476-15|Diptera|Cecidomyiidae||BOLD:ACW3673
Cecidomyiidae|[6617]|GMAGB1133-15|Diptera|Cecidomyiidae||BOLD:ACM4944
Cecidomyiidae|[6618]|GMARB681-14|Diptera|Cecidomyiidae||BOLD:ACM4918
Cecidomyiidae|[6619]|GMARB1695-14|Diptera|Cecidomyiidae||BOLD:ACM5416
Cecidomyiidae|[6620]|GMARR441-14|Diptera|Cecidomyiidae||BOLD:ACN8923
Cecidomyiidae|[6621]|GMARB1267-14|Diptera|Cecidomyiidae||BOLD:ACM5976
Cecidomyiidae|[6622]|GMARA1202-14|Diptera|Cecidomyiidae||BOLD:ACM6411
Cecidomyiidae|[6623]|GMARB1592-14|Diptera|Cecidomyiidae||BOLD:ACM5670
Cecidomyiidae|[6624]|GMARD1529-14|Diptera|Cecidomyiidae||BOLD:ACN1405
Cecidomyiidae|[6625]|GMAGB1497-15|Diptera|Cecidomyiidae||BOLD:ACW4342
Cecidomyiidae|[6626]|GMAGB1377-15|Diptera|Cecidomyiidae||BOLD:ACN0065
Cecidomyiidae|[6627]|GMAGH1246-15|Diptera|Cecidomyiidae||BOLD:ACN0279
Cecidomyiidae|[6628]|GMAGI909-15|Diptera|Cecidomyiidae||BOLD:ACW7723
Cecidomyiidae|[6629]|GMAGG378-15|Diptera|Cecidomyiidae||BOLD:ACW3412
Cecidomyiidae|[6630]|GMAGK2044-15|Diptera|Cecidomyiidae||BOLD:ACW9709
Cecidomyiidae|[6631]|GMARR787-14|Diptera|Cecidomyiidae||BOLD:ACN7828
Cecidomyiidae|[6632]|GMAGA415-15|Diptera|Cecidomyiidae||BOLD:ACM9306
Cecidomyiidae|[6633]|GMARA1245-14|Diptera|Cecidomyiidae||BOLD:ACM6331
Cecidomyiidae|[6634]|GMAGD2142-15|Diptera|Cecidomyiidae||BOLD:ACW2266
Cecidomyiidae|[6635]|GMAGB814-15|Diptera|Cecidomyiidae||BOLD:ACM7370
Cecidomyiidae|[6636]|GMARD408-14|Diptera|Cecidomyiidae||BOLD:ACY5901
Cecidomyiidae|[6637]|GMARH1096-14|Diptera|Cecidomyiidae||BOLD:ACN0461
Cecidomyiidae|[6638]|GMARA1268-14|Diptera|Cecidomyiidae||BOLD:ACM7767
Cecidomyiidae|[6639]|GMARB686-14|Diptera|Cecidomyiidae||BOLD:ACM6325
Cecidomyiidae|[6640]|GMARB1376-14|Diptera|Cecidomyiidae||BOLD:ACM5851
Cecidomyiidae|[6641]|GMARF126-14|Diptera|Cecidomyiidae||BOLD:ACM8793
Cecidomyiidae|[6642]|GMARH1154-14|Diptera|Cecidomyiidae||BOLD:ACN0865
Cecidomyiidae|[6643]|GMAGY132-15|Diptera|Cecidomyiidae||BOLD:ACX2294
Cecidomyiidae|[6644]|GMARG498-14|Diptera|Cecidomyiidae||BOLD:ACN0515
Cecidomyiidae|[6645]|GMAGF535-15|Diptera|Cecidomyiidae||BOLD:ACN1223
Cecidomyiidae|[6646]|GMARH906-14|Diptera|Cecidomyiidae||BOLD:ACN1484
Cecidomyiidae|[6647]|GMARG168-14|Diptera|Cecidomyiidae||BOLD:ACN1593
Cecidomyiidae|[6648]|GMAGF475-15|Diptera|Cecidomyiidae||BOLD:ACM9643
Cecidomyiidae|[6649]|GMARH1205-14|Diptera|Cecidomyiidae||BOLD:ACN1906
Cecidomyiidae|[6650]|GMARI783-14|Diptera|Cecidomyiidae||BOLD:ACX5316
Cecidomyiidae|[6651]|GMARR766-14|Diptera|Cecidomyiidae||BOLD:ACN7918
Cecidomyiidae|[6652]|GMARD554-14|Diptera|Cecidomyiidae||BOLD:ACN0320
Cecidomyiidae|[6653]|GMAGJ2023-15|Diptera|Cecidomyiidae||BOLD:ACN1661
Cecidomyiidae|[6654]|GMAGB786-15|Diptera|Cecidomyiidae||BOLD:ACM8247
Cecidomyiidae|[6655]|GMAGB1457-15|Diptera|Cecidomyiidae||BOLD:ACN1338
Cecidomyiidae|[6656]|GMAGC114-15|Diptera|Cecidomyiidae||BOLD:ACM5387
Cecidomyiidae|[6657]|GMAGI678-15|Diptera|Cecidomyiidae||BOLD:ACM9838
Cecidomyiidae|[6658]|GMARB1448-14|Diptera|Cecidomyiidae||BOLD:ACM5427
Cecidomyiidae|[6659]|GMARF269-14|Diptera|Cecidomyiidae||BOLD:ACN2473
Cecidomyiidae|[6660]|GMARL939-14|Diptera|Cecidomyiidae||BOLD:ACN2231
Cecidomyiidae|[6661]|GMAGI999-15|Diptera|Cecidomyiidae||BOLD:ACW6137
Cecidomyiidae|[6662]|GMARL935-14|Diptera|Cecidomyiidae||BOLD:ACN0439
Cecidomyiidae|[6663]|GMAGI689-15|Diptera|Cecidomyiidae||BOLD:ACN9038
Cecidomyiidae|[6664]|GMAGD2218-15|Diptera|Cecidomyiidae||BOLD:ACM7349
Cecidomyiidae|[6665]|GMARN1876-14|Diptera|Cecidomyiidae||BOLD:ACN6990
Cecidomyiidae|[6666]|GMAGG1721-15|Diptera|Cecidomyiidae||BOLD:ACN1258
Cecidomyiidae|[6667]|GMARF965-14|Diptera|Cecidomyiidae||BOLD:ACX1008
Cecidomyiidae|[6668]|GMAGD1351-15|Diptera|Cecidomyiidae||BOLD:ACN0514
Cecidomyiidae|[6669]|GMARA1413-14|Diptera|Cecidomyiidae||BOLD:ACM9274
Cecidomyiidae|[6670]|GMAGF513-15|Diptera|Cecidomyiidae||BOLD:ACW3482
[illegible]

Cecidomyiidae|[6668]|GMAGD[illegible]-15|Diptera|Cecidomyiidae||BOLD:ACN[illegible]
Cecidomyiidae|[6669]|GMARA1413-14|Diptera|Cecidomyiidae||BOLD:ACM9274
Cecidomyiidae|[6670]|GMAGF513-15|Diptera|Cecidomyiidae||BOLD:ACW3482
Cecidomyiidae|[6671]|GMART1197-14|Diptera|Cecidomyiidae||BOLD:ACO1211
Cecidomyiidae|[6672]|GMARA1325-14|Diptera|Cecidomyiidae||BOLD:ACM5005
Cecidomyiidae|[6673]|GMARR646-14|Diptera|Cecidomyiidae||BOLD:ACX2960
Cecidomyiidae|[6674]|GMAGB1515-15|Diptera|Cecidomyiidae||BOLD:ACW3261
Cecidomyiidae|[6675]|GMAGS1354-15|Diptera|Cecidomyiidae||BOLD:ACN8133
Cecidomyiidae|[6676]|GMARA139-14|Diptera|Cecidomyiidae||BOLD:ACM9006
Cecidomyiidae|[6677]|GMAGD2026-15|Diptera|Cecidomyiidae||BOLD:ACN8832
Cecidomyiidae|[6678]|GMARB996-14|Diptera|Cecidomyiidae||BOLD:ACM5327
Cecidomyiidae|[6679]|GMAGD2184-15|Diptera|Cecidomyiidae||BOLD:ACW7651
Cecidomyiidae|[6680]|GMAGG2265-15|Diptera|Cecidomyiidae||BOLD:ACX4286
Cecidomyiidae|[6681]|GMAGG2055-15|Diptera|Cecidomyiidae||BOLD:ACW7564
Cecidomyiidae|[6682]|GMAGV968-15|Diptera|Cecidomyiidae||BOLD:ACX5369
Cecidomyiidae|[6683]|GMART1599-14|Diptera|Cecidomyiidae||BOLD:ACN9505
Cecidomyiidae|[6684]|GMAGI743-15|Diptera|Cecidomyiidae||BOLD:ACN2606
Cecidomyiidae|[6685]|GMARF358-14|Diptera|Cecidomyiidae||BOLD:ACM9563
Cecidomyiidae|[6686]|GMARA1292-14|Diptera|Cecidomyiidae||BOLD:ACM6275
Cecidomyiidae|[6687]|GMAGG1267-15|Diptera|Cecidomyiidae||BOLD:ACN1278
Cecidomyiidae|[6688]|GMAGE2243-15|Diptera|Cecidomyiidae||BOLD:ACN7797
Cecidomyiidae|[6689]|GMAGC025-15|Diptera|Cecidomyiidae||BOLD:ACV8590
Cecidomyiidae|[6690]|GMAGF705-15|Diptera|Cecidomyiidae||BOLD:ACW3238
Cecidomyiidae|[6691]|GMARC725-14|Diptera|Cecidomyiidae||BOLD:ACM4833
Cecidomyiidae|[6692]|GMAGR415-15|Diptera|Cecidomyiidae||BOLD:ACX6462
Cecidomyiidae|[6693]|GMARE299-14|Diptera|Cecidomyiidae||BOLD:ACN1835
Cecidomyiidae|[6694]|GMARO378-14|Diptera|Cecidomyiidae||BOLD:ACN3055
Cecidomyiidae|[6695]|GMAGC400-15|Diptera|Cecidomyiidae||BOLD:ACW1800
Cecidomyiidae|[6696]|GMARI1878-14|Diptera|Cecidomyiidae||BOLD:ACN1826
Cecidomyiidae|[6697]|GMARA1152-14|Diptera|Cecidomyiidae||BOLD:ACM7342
Cecidomyiidae|[6698]|GMAGG2295-15|Diptera|Cecidomyiidae||BOLD:ACN0709
Cecidomyiidae|[6699]|GMARE086-14|Diptera|Cecidomyiidae||BOLD:ACN1692
Cecidomyiidae|[6700]|GMARL627-14|Diptera|Cecidomyiidae||BOLD:ACN5933
Cecidomyiidae|[6701]|GMAGE2162-15|Diptera|Cecidomyiidae||BOLD:ACW5923
Cecidomyiidae|[6702]|GMARG1372-14|Diptera|Cecidomyiidae||BOLD:ACN0303
Cecidomyiidae|[6703]|GMAGA526-15|Diptera|Cecidomyiidae||BOLD:ACN9151
Cecidomyiidae|[6704]|GMAGD2015-15|Diptera|Cecidomyiidae||BOLD:ACN1832
Cecidomyiidae|[6705]|GMARC295-14|Diptera|Cecidomyiidae||BOLD:ACM7566
Cecidomyiidae|[6706]|GMAGJ1014-15|Diptera|Cecidomyiidae||BOLD:ACN2289
Cecidomyiidae|[6707]|GMAGM1934-15|Diptera|Cecidomyiidae||BOLD:ACN6607
Cecidomyiidae|[6708]|GMARK1170-14|Diptera|Cecidomyiidae||BOLD:ACN0184
Cecidomyiidae|[6709]|GMARM632-14|Diptera|Cecidomyiidae||BOLD:ACX2414
Cecidomyiidae|[6710]|GMAGE1096-15|Diptera|Cecidomyiidae||BOLD:ACM8500
Cecidomyiidae|[6711]|GMAGG1883-15|Diptera|Cecidomyiidae||BOLD:ACW6258
Cecidomyiidae|[6712]|GMARD1124-14|Diptera|Cecidomyiidae||BOLD:ACM8927
Cecidomyiidae|[6713]|GMARF236-14|Diptera|Cecidomyiidae||BOLD:ACM9251
Cecidomyiidae|[6714]|GMARB849-14|Diptera|Cecidomyiidae||BOLD:ACM5359
Cecidomyiidae|[6715]|GMARD2138-14|Diptera|Cecidomyiidae||BOLD:ACN0087
Cecidomyiidae|[6716]|GMARA1242-14|Diptera|Cecidomyiidae||BOLD:ACM5294
Cecidomyiidae|[6717]|GMARR564-14|Diptera|Cecidomyiidae||BOLD:ACN8037
Cecidomyiidae|[6718]|GMAGW270-15|Diptera|Cecidomyiidae||BOLD:ACX3271
Cecidomyiidae|[6719]|GMAGC120-15|Diptera|Cecidomyiidae||BOLD:ACV6720
Cecidomyiidae|[6720]|GMAGC1485-15|Diptera|Cecidomyiidae||BOLD:ACM8883
Cecidomyiidae|[6721]|GMARA1127-14|Diptera|Cecidomyiidae||BOLD:ADD0857
Cecidomyiidae|[6722]|GMARA1529-14|Diptera|Cecidomyiidae||BOLD:ACM4969
Cecidomyiidae|[6723]|GMARA1578-14|Diptera|Cecidomyiidae||BOLD:ADD0859
Cecidomyiidae|[6724]|GMARB807-14|Diptera|Cecidomyiidae||BOLD:ADD0860
Cecidomyiidae|[6725]|GMAGJ647-15|Diptera|Cecidomyiidae||BOLD:ADD0858
Cecidomyiidae|[6726]|GMAGE1887-15|Diptera|Cecidomyiidae||BOLD:ACW7709
Cecidomyiidae|[6727]|GMARE508-14|Diptera|Cecidomyiidae||BOLD:ACM8226
Cecidomyiidae|[6728]|GMARA308-14|Diptera|Cecidomyiidae||BOLD:ACM9837
Cecidomyiidae|[6729]|GMARB1751-14|Diptera|Cecidomyiidae||BOLD:ACM6366
Cecidomyiidae|[6730]|GMAGJ1754-15|Diptera|Cecidomyiidae||BOLD:ACX5120
Cecidomyiidae|[6731]|GMAGS560-15|Diptera|Cecidomyiidae||BOLD:ACX5728
Cecidomyiidae|[6732]|GMAGA503-15|Diptera|Cecidomyiidae||BOLD:ACM6236
Cecidomyiidae|[6733]|GMARB1675-14|Diptera|Cecidomyiidae||BOLD:ACM5804
Cecidomyiidae|[6734]|GMARG116-14|Diptera|Cecidomyiidae||BOLD:ACN0071
Cecidomyiidae|[6735]|GMARD1191-14|Diptera|Cecidomyiidae||BOLD:ACM9347
Cecidomyiidae|[6736]|GMAGM2013-15|Diptera|Cecidomyiidae||BOLD:ACX3913
Cecidomyiidae|[6737]|GMAGY426-15|Diptera|Cecidomyiidae||BOLD:ACX2498
Cecidomyiidae|[6738]|GMARR141-14|Diptera|Cecidomyiidae||BOLD:ACN8323
Cecidomyiidae|[6739]|GMARB1135-14|Diptera|Cecidomyiidae||BOLD:ACM5391
Cecidomyiidae|[6740]|GMAGE2465-15|Diptera|Cecidomyiidae||BOLD:ACW6313
Cecidomyiidae|[6741]|GMARA125-14|Diptera|Cecidomyiidae||BOLD:ACM8834
Cecidomyiidae|[6742]|GMAGD993-15|Diptera|Cecidomyiidae||BOLD:ACW4690
Cecidomyiidae|[6743]|GMARA031-14|Diptera|Cecidomyiidae||BOLD:ACM5458
Cecidomyiidae|[6744]|GMARA366-14|Diptera|Cecidomyiidae||BOLD:ACM8778
Cecidomyiidae|[6745]|GMARA1224-14|Diptera|Cecidomyiidae||BOLD:ACM6273
Cecidomyiidae|[6746]|GMAGA556-15|Diptera|Cecidomyiidae||BOLD:ACM4755
Cecidomyiidae|[6747]|GMARD1023-14|Diptera|Cecidomyiidae||BOLD:ACO0668
Cecidomyiidae|[6748]|GMAGE2432-15|Diptera|Cecidomyiidae||BOLD:ACX3936
Cecidomyiidae|[6749]|GMARG263-14|Diptera|Cecidomyiidae||BOLD:ACN1017
Cecidomyiidae|[6750]|GMAGA578-15|Diptera|Cecidomyiidae||BOLD:ACM5796
Cecidomyiidae|[6751]|GMARA1232-14|Diptera|Cecidomyiidae||BOLD:ACM8924
Cecidomyiidae|[6752]|GMARB718-14|Diptera|Cecidomyiidae||BOLD:ACM5837
Cecidomyiidae|[6753]|GMARS264-14|Diptera|Cecidomyiidae||BOLD:ACN8725
Cecidomyiidae|[6754]|GMAGR788-15|Diptera|Cecidomyiidae||BOLD:ACX7671
Cecidomyiidae|[6755]|GMAGU799-15|Diptera|Cecidomyiidae||BOLD:ACX4809
Cecidomyiidae|[6756]|GMARG1514-14|Diptera|Cecidomyiidae||BOLD:ACN1630
Cecidomyiidae|[6757]|GMART232-14|Diptera|Cecidomyiidae||BOLD:ACN9915
Cecidomyiidae|[6758]|GMARU292-14|Diptera|Cecidomyiidae||BOLD:ACO1176
Cecidomyiidae|[6759]|GMAGD258-15|Diptera|Cecidomyiidae||BOLD:ACW5630
Cecidomyiidae|[6760]|GMAGE2379-15|Diptera|Cecidomyiidae||BOLD:ACN8830
Cecidomyiidae|[6761]|GMARR517-14|Diptera|Cecidomyiidae||BOLD:ACN8233
Cecidomyiidae|[6762]|GMARV037-14|Diptera|Cecidomyiidae||BOLD:ACO1925
Cecidomyiidae|[6763]|GMAGK2046-15|Diptera|Cecidomyiidae||BOLD:ACW9322
Cecidomyiidae|[6764]|GMARD1019-14|Diptera|Cecidomyiidae||BOLD:ACM9651
Cecidomyiidae|[6765]|GMARO1287-14|Diptera|Cecidomyiidae||BOLD:ACO3832
Cecidomyiidae|[6766]|GMARS273-14|Diptera|Cecidomyiidae||BOLD:ACN8260
Cecidomyiidae|[6767]|GMAGC225-15|Diptera|Cecidomyiidae||BOLD:ACW1778
Cecidomyiidae|[6768]|GMARB1484-14|Diptera|Cecidomyiidae||BOLD:ACM6079
Cecidomyiidae|[6769]|GMAGD2167-15|Diptera|Cecidomyiidae||BOLD:ACW2349
Cecidomyiidae|[6770]|GMARC447-14|Diptera|Cecidomyiidae||BOLD:ACM7241

Cecidomyiidae|[6768]|GMARB1484-14|Diptera|Cecidomyiidae||BOLD:ACM6079
Cecidomyiidae|[6769]|GMAGD2167-15|Diptera|Cecidomyiidae||BOLD:ACW2349
Cecidomyiidae|[6770]|GMARC447-14|Diptera|Cecidomyiidae||BOLD:ACM7241
Cecidomyiidae|[6771]|GMAGG1945-15|Diptera|Cecidomyiidae||BOLD:ACW6128
Cecidomyiidae|[6772]|GMAGC109-15|Diptera|Cecidomyiidae||BOLD:ACV8778
Cecidomyiidae|[6773]|GMAGG1989-15|Diptera|Cecidomyiidae||BOLD:ACW6390
Cecidomyiidae|[6774]|GMAGA502-15|Diptera|Cecidomyiidae||BOLD:ACV1992
Cecidomyiidae|[6775]|GMAGI700-15|Diptera|Cecidomyiidae||BOLD:ACN0648
Cecidomyiidae|[6776]|GMART1158-14|Diptera|Cecidomyiidae||BOLD:ACO1169
Cecidomyiidae|[6777]|GMAGE2503-15|Diptera|Cecidomyiidae||BOLD:ACW7681
Cecidomyiidae|[6778]|GMAGI961-15|Diptera|Cecidomyiidae||BOLD:ACN4519
Cecidomyiidae|[6779]|GMARC323-14|Diptera|Cecidomyiidae||BOLD:ACM7190
Cecidomyiidae|[6780]|GMAGB1372-15|Diptera|Cecidomyiidae||BOLD:ACW3321
Cecidomyiidae|[6781]|GMARL971-14|Diptera|Cecidomyiidae||BOLD:ACN2675
Cecidomyiidae|[6782]|GMARB1450-14|Diptera|Cecidomyiidae||BOLD:ACM5578
Cecidomyiidae|[6783]|GMAGL2135-15|Diptera|Cecidomyiidae||BOLD:ACW9012
Cecidomyiidae|[6784]|GMARP083-14|Diptera|Cecidomyiidae||BOLD:ACO2894
Cecidomyiidae|[6785]|GMAGC610-15|Diptera|Cecidomyiidae||BOLD:ACW1707
Cecidomyiidae|[6786]|GMAGA146-15|Diptera|Cecidomyiidae||BOLD:ACM4885
Cecidomyiidae|[6787]|GMAGC576-15|Diptera|Cecidomyiidae||BOLD:ACW2042
Cecidomyiidae|[6788]|GMAGD1319-15|Diptera|Cecidomyiidae||BOLD:ACX3531
Cecidomyiidae|[6789]|GMAGI841-15|Diptera|Cecidomyiidae||BOLD:ACW6241
Cecidomyiidae|[6790]|GMAGJ882-15|Diptera|Cecidomyiidae||BOLD:ACW9216
Cecidomyiidae|[6791]|GMARA054-14|Diptera|Cecidomyiidae||BOLD:ACM8748
Cecidomyiidae|[6792]|GMARB659-14|Diptera|Cecidomyiidae||BOLD:ACM6429
Cecidomyiidae|[6793]|GMARB644-14|Diptera|Cecidomyiidae||BOLD:ACM4931
Cecidomyiidae|[6794]|GMAGF469-15|Diptera|Cecidomyiidae||BOLD:ACW3066
Cecidomyiidae|[6795]|GMARB586-14|Diptera|Cecidomyiidae||BOLD:ACM6328
Cecidomyiidae|[6796]|GMARA1556-14|Diptera|Cecidomyiidae||BOLD:ACM5923
Cecidomyiidae|[6797]|GMART1006-14|Diptera|Cecidomyiidae||BOLD:ACN9340
Cecidomyiidae|[6798]|GMARA271-14|Diptera|Cecidomyiidae||BOLD:ACM9193
Cecidomyiidae|[6799]|GMAGC138-15|Diptera|Cecidomyiidae||BOLD:ACV6360
Cecidomyiidae|[6800]|GMARB678-14|Diptera|Cecidomyiidae||BOLD:ACM5990
Cecidomyiidae|[6801]|GMARE118-14|Diptera|Cecidomyiidae||BOLD:ACN0694
Cecidomyiidae|[6802]|GMARA1559-14|Diptera|Cecidomyiidae||BOLD:ACM5242
Cecidomyiidae|[6803]|GMARF040-14|Diptera|Cecidomyiidae||BOLD:ACN1841
Cecidomyiidae|[6804]|GMARH478-14|Diptera|Cecidomyiidae||BOLD:ACN1868
Cecidomyiidae|[6805]|GMARO232-14|Diptera|Cecidomyiidae||BOLD:ACN3285
Cecidomyiidae|[6806]|GMARD654-14|Diptera|Cecidomyiidae||BOLD:ACN0086
Cecidomyiidae|[6807]|GMAGC1027-15|Diptera|Cecidomyiidae||BOLD:ACV7127
Cecidomyiidae|[6808]|GMARU062-14|Diptera|Cecidomyiidae||BOLD:ACO0954
Cecidomyiidae|[6809]|GMAGE2911-15|Diptera|Cecidomyiidae||BOLD:ACW7689
Cecidomyiidae|[6810]|GMAGJ2054-15|Diptera|Cecidomyiidae||BOLD:ACX2635
Cecidomyiidae|[6811]|GMAGM1490-15|Diptera|Cecidomyiidae||BOLD:ACN0906
Cecidomyiidae|[6812]|GMAGB1540-15|Diptera|Cecidomyiidae||BOLD:ACO0861
Cecidomyiidae|[6813]|GMAGA421-15|Diptera|Cecidomyiidae||BOLD:ACV3035
Cecidomyiidae|[6814]|GMAGA871-15|Diptera|Cecidomyiidae||BOLD:ACW3627
Cecidomyiidae|[6815]|GMARD909-14|Diptera|Cecidomyiidae||BOLD:ACM7604
Cecidomyiidae|[6816]|GMAGN2296-15|Diptera|Cecidomyiidae||BOLD:ACX1787
Cecidomyiidae|[6817]|GMAGR139-15|Diptera|Cecidomyiidae||BOLD:ACX6423
Cecidomyiidae|[6818]|GMAGO256-15|Diptera|Cecidomyiidae||BOLD:ACX5481
Cecidomyiidae|[6819]|GMAGD2276-15|Diptera|Cecidomyiidae||BOLD:ACN9570
Cecidomyiidae|[6820]|GMAGK1796-15|Diptera|Cecidomyiidae||BOLD:ACX3975
Cecidomyiidae|[6821]|GMARC461-14|Diptera|Cecidomyiidae||BOLD:ACM7504
Cecidomyiidae|[6822]|GMARI281-14|Diptera|Cecidomyiidae||BOLD:ACN0064
Cecidomyiidae|[6823]|GMAGE1687-15|Diptera|Cecidomyiidae||BOLD:ACM9062
Cecidomyiidae|[6824]|GMARI502-14|Diptera|Cecidomyiidae||BOLD:ACN0230
Cecidomyiidae|[6825]|GMAGP1631-15|Diptera|Cecidomyiidae||BOLD:ACX6114
Cecidomyiidae|[6826]|GMARM415-14|Diptera|Cecidomyiidae||BOLD:ACN2542
Cecidomyiidae|[6827]|GMARA066-14|Diptera|Cecidomyiidae||BOLD:ACM8014
Cecidomyiidae|[6828]|GMARQ211-14|Diptera|Cecidomyiidae||BOLD:ACX8280
Cecidomyiidae|[6829]|GMARA191-14|Diptera|Cecidomyiidae||BOLD:ACM6422
Cecidomyiidae|[6830]|GMARB1058-14|Diptera|Cecidomyiidae||BOLD:ACN9826
Cecidomyiidae|[6831]|GMAGD622-15|Diptera|Cecidomyiidae||BOLD:ACW2957
Cecidomyiidae|[6832]|GMAGV393-15|Diptera|Cecidomyiidae||BOLD:ACX2937
Cecidomyiidae|[6833]|GMAGY404-15|Diptera|Cecidomyiidae||BOLD:ACX2812
Cecidomyiidae|[6834]|GMARA1515-14|Diptera|Cecidomyiidae||BOLD:ACM6516
Cecidomyiidae|[6835]|GMARA1508-14|Diptera|Cecidomyiidae||BOLD:ACM6023
Cecidomyiidae|[6836]|GMARA1523-14|Diptera|Cecidomyiidae||BOLD:ACM6308
Cecidomyiidae|[6837]|GMAGC428-15|Diptera|Cecidomyiidae||BOLD:ACW2002
Cecidomyiidae|[6838]|GMAGB1098-15|Diptera|Cecidomyiidae||BOLD:ACW2437
Cecidomyiidae|[6839]|GMAGB1022-15|Diptera|Cecidomyiidae||BOLD:ACM5668
Cecidomyiidae|[6840]|GMAGH1248-15|Diptera|Cecidomyiidae||BOLD:ACW3390
Cecidomyiidae|[6841]|GMAGD2332-15|Diptera|Cecidomyiidae||BOLD:ACM7460
Cecidomyiidae|[6842]|GMAGH1274-15|Diptera|Cecidomyiidae||BOLD:ACW5997
Cecidomyiidae|[6843]|GMARE1318-14|Diptera|Cecidomyiidae||BOLD:ACY4515
Cecidomyiidae|[6844]|GMARB1284-14|Diptera|Cecidomyiidae||BOLD:ACM5815
Cecidomyiidae|[6845]|GMAGQ839-15|Diptera|Cecidomyiidae||BOLD:ACX5559
Cecidomyiidae|[6846]|GMAGE2187-15|Diptera|Cecidomyiidae||BOLD:ACM5532
Cecidomyiidae|[6847]|GMARD277-14|Diptera|Cecidomyiidae||BOLD:ACM7732
Cecidomyiidae|[6848]|GMAGH235-15|Diptera|Cecidomyiidae||BOLD:ACN8876
Cecidomyiidae|[6849]|GMAGU606-15|Diptera|Cecidomyiidae||BOLD:ACX5674
Cecidomyiidae|[6850]|GMAGB677-15|Diptera|Cecidomyiidae||BOLD:ACM7505
Cecidomyiidae|[6851]|GMARP180-14|Diptera|Cecidomyiidae||BOLD:ACN9317
Cecidomyiidae|[6852]|GMAGE2440-15|Diptera|Cecidomyiidae||BOLD:ACM5348
Cecidomyiidae|[6853]|GMAGE121-15|Diptera|Cecidomyiidae||BOLD:ACM8677
Cecidomyiidae|[6854]|GMARK1355-14|Diptera|Cecidomyiidae||BOLD:ACN2629
Cecidomyiidae|[6855]|GMAGU506-15|Diptera|Cecidomyiidae||BOLD:ACY6341
Cecidomyiidae|[6856]|GMAGA509-15|Diptera|Cecidomyiidae||BOLD:ACM5985
Cecidomyiidae|[6857]|GMARA245-14|Diptera|Cecidomyiidae||BOLD:ACM9414
Cecidomyiidae|[6858]|GMARA037-14|Diptera|Cecidomyiidae||BOLD:ACM8723
Cecidomyiidae|[6859]|GMARB1314-14|Diptera|Cecidomyiidae||BOLD:ACM4977
Cecidomyiidae|[6860]|GMARA1514-14|Diptera|Cecidomyiidae||BOLD:ACM9763
Cecidomyiidae|[6861]|GMARG368-14|Diptera|Cecidomyiidae||BOLD:ACN1611
Cecidomyiidae|[6862]|GMAGY256-15|Diptera|Cecidomyiidae||BOLD:ACX1521
Cecidomyiidae|[6863]|GMAGD263-15|Diptera|Cecidomyiidae||BOLD:ACW5759
Cecidomyiidae|[6864]|GMAGZ085-15|Diptera|Cecidomyiidae||BOLD:ACX3549
Cecidomyiidae|[6865]|GMARB1009-14|Diptera|Cecidomyiidae||BOLD:ACM6402
Cecidomyiidae|[6866]|GMARA1323-14|Diptera|Cecidomyiidae||BOLD:ACM9269
Cecidomyiidae|[6867]|GMAGC238-15|Diptera|Cecidomyiidae||BOLD:ACX2964
Cecidomyiidae|[6868]|GMAGS1011-15|Diptera|Cecidomyiidae||BOLD:ACX5950
Cecidomyiidae|[6869]|GMAGB1524-15|Diptera|Cecidomyiidae||BOLD:ACW3384
Cecidomyiidae|[6870]|GMARQ381-14|Diptera|Cecidomyiidae||BOLD:ACN8701

Cecidomyiidae|[6868]|GMAGS1011-15|Diptera|Cecidomyiidae||BOLD:ACX5950
Cecidomyiidae|[6869]|GMAGB1524-15|Diptera|Cecidomyiidae||BOLD:ACW3384
Cecidomyiidae|[6870]|GMARQ381-14|Diptera|Cecidomyiidae||BOLD:ACN8701
Cecidomyiidae|[6871]|GMAGA461-15|Diptera|Cecidomyiidae||BOLD:ACM9930
Cecidomyiidae|[6872]|GMAGE1940-15|Diptera|Cecidomyiidae||BOLD:ACM7855
Cecidomyiidae|[6873]|GMARE630-14|Diptera|Cecidomyiidae||BOLD:ACM8296
Cecidomyiidae|[6874]|GMAGB586-15|Diptera|Cecidomyiidae||BOLD:ACW3405
Cecidomyiidae|[6875]|GMAGE1082-15|Diptera|Cecidomyiidae||BOLD:ACW1466
Cecidomyiidae|[6876]|GMARL532-14|Diptera|Cecidomyiidae||BOLD:ACN1981
Cecidomyiidae|[6877]|GMAGQ958-15|Diptera|Cecidomyiidae||BOLD:ACX5668
Cecidomyiidae|[6878]|GMAGB792-15|Diptera|Cecidomyiidae||BOLD:ACM9829
Cecidomyiidae|[6879]|GMAGB1512-15|Diptera|Cecidomyiidae||BOLD:ACW4271
Cecidomyiidae|[6880]|GMAGD1157-15|Diptera|Cecidomyiidae||BOLD:ACW4642
Cecidomyiidae|[6881]|GMAGB1490-15|Diptera|Cecidomyiidae||BOLD:ACM9145
Cecidomyiidae|[6882]|GMAGB303-15|Diptera|Cecidomyiidae||BOLD:ACW2351
Cecidomyiidae|[6883]|GMAGB560-15|Diptera|Cecidomyiidae||BOLD:ACM8903
Cecidomyiidae|[6884]|GMAGT234-15|Diptera|Cecidomyiidae||BOLD:ACX4669
Cecidomyiidae|[6885]|GMAGP1522-15|Diptera|Cecidomyiidae||BOLD:ACX6183
Cecidomyiidae|[6886]|GMARC750-14|Diptera|Cecidomyiidae||BOLD:ACM6408
Cecidomyiidae|[6887]|GMAGQ1354-15|Diptera|Cecidomyiidae||BOLD:ACX4760
Cecidomyiidae|[6888]|GMARA1548-14|Diptera|Cecidomyiidae||BOLD:ACM5988
Cecidomyiidae|[6889]|GMAGB501-15|Diptera|Cecidomyiidae||BOLD:ACM9464
Cecidomyiidae|[6890]|GMARG378-14|Diptera|Cecidomyiidae||BOLD:ACN1387
Cecidomyiidae|[6891]|GMARL159-14|Diptera|Cecidomyiidae||BOLD:ACN2059
Cecidomyiidae|[6892]|GMARM407-14|Diptera|Cecidomyiidae||BOLD:ACN2475
Cecidomyiidae|[6893]|GMARM771-14|Diptera|Cecidomyiidae||BOLD:ACO0371
Cecidomyiidae|[6894]|GMARJ1578-14|Diptera|Cecidomyiidae||BOLD:ACX5901
Cecidomyiidae|[6895]|GMARM1110-14|Diptera|Cecidomyiidae||BOLD:ACM9986
Cecidomyiidae|[6896]|GMARD898-14|Diptera|Cecidomyiidae||BOLD:ACM8497
Cecidomyiidae|[6897]|GMARD086-14|Diptera|Cecidomyiidae||BOLD:ACM7581
Cecidomyiidae|[6898]|GMAGB838-15|Diptera|Cecidomyiidae||BOLD:ACW6705
Cecidomyiidae|[6899]|GMAGV610-15|Diptera|Cecidomyiidae||BOLD:ACX2808
Cecidomyiidae|[6900]|GMAGL1916-15|Diptera|Cecidomyiidae||BOLD:ACN2273
Cecidomyiidae|[6901]|GMARI585-14|Diptera|Cecidomyiidae||BOLD:ACN0327
Cecidomyiidae|[6902]|GMARR501-14|Diptera|Cecidomyiidae||BOLD:ACN7754
Cecidomyiidae|[6903]|GMARB1171-14|Diptera|Cecidomyiidae||BOLD:ACM5926
Cecidomyiidae|[6904]|GMAGD1333-15|Diptera|Cecidomyiidae||BOLD:ACW3561
Cecidomyiidae|[6905]|GMARC093-14|Diptera|Cecidomyiidae||BOLD:ACM5609
Cecidomyiidae|[6906]|GMAGB1519-15|Diptera|Cecidomyiidae||BOLD:ACW3499
Cecidomyiidae|[6907]|GMAGB343-15|Diptera|Cecidomyiidae||BOLD:ACW1852
Cecidomyiidae|[6908]|GMAGC410-15|Diptera|Cecidomyiidae||BOLD:ACW2621
Cecidomyiidae|[6909]|GMARA1085-14|Diptera|Cecidomyiidae||BOLD:ACM5671
Cecidomyiidae|[6910]|GMAGE2372-15|Diptera|Cecidomyiidae||BOLD:ACX4661
Cecidomyiidae|[6911]|GMARK1252-14|Diptera|Cecidomyiidae||BOLD:ACN1027
Cecidomyiidae|[6912]|GMAGD1321-15|Diptera|Cecidomyiidae||BOLD:ACN1345
Cecidomyiidae|[6913]|GMAGL2275-15|Diptera|Cecidomyiidae||BOLD:ACN9052
Cecidomyiidae|[6914]|GMART724-14|Diptera|Cecidomyiidae||BOLD:ACN9093
Cecidomyiidae|[6915]|GMARM753-14|Diptera|Cecidomyiidae||BOLD:ACX3113
Cecidomyiidae|[6916]|GMARL1272-14|Diptera|Cecidomyiidae||BOLD:ACN2381
Cecidomyiidae|[6917]|GMAGQ1813-15|Diptera|Cecidomyiidae||BOLD:ACX5949
Cecidomyiidae|[6918]|GMARA1344-14|Diptera|Cecidomyiidae||BOLD:ACM9690
Cecidomyiidae|[6919]|GMARB907-14|Diptera|Cecidomyiidae||BOLD:ACM5542
Cecidomyiidae|[6920]|GMART614-14|Diptera|Cecidomyiidae||BOLD:ACN9726
Cecidomyiidae|[6921]|GMAGA672-15|Diptera|Cecidomyiidae||BOLD:ACM4889
Cecidomyiidae|[6922]|GMAGD471-15|Diptera|Cecidomyiidae||BOLD:ACN0456
Cecidomyiidae|[6923]|GMARB1597-14|Diptera|Cecidomyiidae||BOLD:ACM5853
Cecidomyiidae|[6924]|GMAGD710-15|Diptera|Cecidomyiidae||BOLD:ACW3582
Cecidomyiidae|[6925]|GMARB936-14|Diptera|Cecidomyiidae||BOLD:ACM5558
Cecidomyiidae|[6926]|GMARA323-14|Diptera|Cecidomyiidae||BOLD:ACM8832
Cecidomyiidae|[6927]|GMARO1023-14|Diptera|Cecidomyiidae||BOLD:ACN8363
Cecidomyiidae|[6928]|GMAGJ1161-15|Diptera|Cecidomyiidae||BOLD:ACN4723
Cecidomyiidae|[6929]|GMARA1188-14|Diptera|Cecidomyiidae||BOLD:ACM9699
Cecidomyiidae|[6930]|GMARL438-14|Diptera|Cecidomyiidae||BOLD:ACN4180
Cecidomyiidae|[6931]|GMAGP1063-15|Diptera|Cecidomyiidae||BOLD:ACX5787
Cecidomyiidae|[6932]|GMAGQ1362-15|Diptera|Cecidomyiidae||BOLD:ACX4472
Cecidomyiidae|[6933]|GMAGI863-15|Diptera|Cecidomyiidae||BOLD:ACN5056
Cecidomyiidae|[6934]|GMAGK1161-15|Diptera|Cecidomyiidae||BOLD:ACN0016
Cecidomyiidae|[6935]|GMARK1482-14|Diptera|Cecidomyiidae||BOLD:ACN0113
Cecidomyiidae|[6936]|GMART517-14|Diptera|Cecidomyiidae||BOLD:ACO0444
Cecidomyiidae|[6937]|GMARA1329-14|Diptera|Cecidomyiidae||BOLD:ACM8545
Cecidomyiidae|[6938]|GMAGE2041-15|Diptera|Cecidomyiidae||BOLD:ACW6809
Cecidomyiidae|[6939]|GMARH970-14|Diptera|Cecidomyiidae||BOLD:ACN0531
Cecidomyiidae|[6940]|GMARS423-14|Diptera|Cecidomyiidae||BOLD:ACN8057
Cecidomyiidae|[6941]|GMAGB1381-15|Diptera|Cecidomyiidae||BOLD:ACN7398
Cecidomyiidae|[6942]|GMAGB704-15|Diptera|Cecidomyiidae||BOLD:ACN9034
Cecidomyiidae|[6943]|GMARA130-14|Diptera|Cecidomyiidae||BOLD:ACM8562
Cecidomyiidae|[6944]|GMAGY424-15|Diptera|Cecidomyiidae||BOLD:ACX2084
Cecidomyiidae|[6945]|GMAGB795-15|Diptera|Cecidomyiidae||BOLD:ACM5298
Cecidomyiidae|[6946]|GMARB585-14|Diptera|Cecidomyiidae||BOLD:ACM6327
Cecidomyiidae|[6947]|GMAGC159-15|Diptera|Cecidomyiidae||BOLD:ACN8969
Cecidomyiidae|[6948]|GMAGD407-15|Diptera|Cecidomyiidae||BOLD:ACW5182
Cecidomyiidae|[6949]|GMARA341-14|Diptera|Cecidomyiidae||BOLD:ACM8482
Cecidomyiidae|[6950]|GMAGB513-15|Diptera|Cecidomyiidae||BOLD:ACW6991
Cecidomyiidae|[6951]|GMAGD872-15|Diptera|Cecidomyiidae||BOLD:ACW5583
Cecidomyiidae|[6952]|GMAGE2420-15|Diptera|Cecidomyiidae||BOLD:ACX5113
Cecidomyiidae|[6953]|GMAGK2055-15|Diptera|Cecidomyiidae||BOLD:ACN1672
Cecidomyiidae|[6954]|GMARK1413-14|Diptera|Cecidomyiidae||BOLD:ACN2330
Cecidomyiidae|[6955]|GMAGC226-15|Diptera|Cecidomyiidae||BOLD:ACW1576
Cecidomyiidae|[6956]|GMAGE1881-15|Diptera|Cecidomyiidae||BOLD:ACW6808
Cecidomyiidae|[6957]|GMARB1059-14|Diptera|Cecidomyiidae||BOLD:ACM5866
Cecidomyiidae|[6958]|GMARA1547-14|Diptera|Cecidomyiidae||BOLD:ACM5803
Cecidomyiidae|[6959]|GMARB1512-14|Diptera|Cecidomyiidae||BOLD:ACM5236
Cecidomyiidae|[6960]|GMARC462-14|Diptera|Cecidomyiidae||BOLD:ACM7387
Cecidomyiidae|[6961]|GMARN1920-14|Diptera|Cecidomyiidae||BOLD:ACN4612
Cecidomyiidae|[6962]|GMAGB395-15|Diptera|Cecidomyiidae||BOLD:ACM6246
Cecidomyiidae|[6963]|GMARB801-14|Diptera|Cecidomyiidae||BOLD:ACM4758
Cecidomyiidae|[6964]|GMAGU887-15|Diptera|Cecidomyiidae||BOLD:ACX6008
Cecidomyiidae|[6965]|GMARA1375-14|Diptera|Cecidomyiidae||BOLD:ACM9677
Cecidomyiidae|[6966]|GMARC129-14|Diptera|Cecidomyiidae||BOLD:ACM7414
Cecidomyiidae|[6967]|GMAGS311-15|Diptera|Cecidomyiidae||BOLD:ACX4737
Cecidomyiidae|[6968]|GMARB1262-14|Diptera|Cecidomyiidae||BOLD:ACM5145
Cecidomyiidae|[6969]|GMAGD1025-15|Diptera|Cecidomyiidae||BOLD:ACN3353

Cecidomyiidae|[6967]|GMAGS311-15|Diptera|Cecidomyiidae||BOLD:ACX4737
Cecidomyiidae|[6968]|GMARB1262-14|Diptera|Cecidomyiidae||BOLD:ACM5145
Cecidomyiidae|[6969]|GMAGD1025-15|Diptera|Cecidomyiidae||BOLD:ACN3353
Cecidomyiidae|[6970]|GMAGB1386-15|Diptera|Cecidomyiidae||BOLD:ACW2876
Cecidomyiidae|[6971]|GMAGB1418-15|Diptera|Cecidomyiidae||BOLD:ABV1359
Cecidomyiidae|[6972]|GMAGH764-15|Diptera|Cecidomyiidae||BOLD:ACY4378
Cecidomyiidae|[6973]|GMAGE2360-15|Diptera|Cecidomyiidae||BOLD:ACX4201
Cecidomyiidae|[6974]|GMAGB834-15|Diptera|Cecidomyiidae||BOLD:ACW6615
Cecidomyiidae|[6975]|GMAGQ1232-15|Diptera|Cecidomyiidae||BOLD:ACX4276
Cecidomyiidae|[6976]|GMAGE2368-15|Diptera|Cecidomyiidae||BOLD:ACX5025
Cecidomyiidae|[6977]|GMAGA491-15|Diptera|Cecidomyiidae||BOLD:ACM6267
Cecidomyiidae|[6978]|GMAGT765-15|Diptera|Cecidomyiidae||BOLD:ACX5129
Cecidomyiidae|[6979]|GMARB816-14|Diptera|Cecidomyiidae||BOLD:ACM5295
Cecidomyiidae|[6980]|GMART1414-14|Diptera|Cecidomyiidae||BOLD:ACN9032
Cecidomyiidae|[6981]|GMAGQ189-15|Diptera|Cecidomyiidae||BOLD:ACX5243
Cecidomyiidae|[6982]|GMAGB770-15|Diptera|Cecidomyiidae||BOLD:ACO0756
Cecidomyiidae|[6983]|GMAGD743-15|Diptera|Cecidomyiidae||BOLD:ACN7780
Cecidomyiidae|[6984]|GMAGB526-15|Diptera|Cecidomyiidae||BOLD:ACM5626
Cecidomyiidae|[6985]|GMARB1473-14|Diptera|Cecidomyiidae||BOLD:ACM5537
Cecidomyiidae|[6986]|GMARB1178-14|Diptera|Cecidomyiidae||BOLD:ACM5171
Cecidomyiidae|[6987]|GMART1582-14|Diptera|Cecidomyiidae||BOLD:ACN9415
Cecidomyiidae|[6988]|GMARE896-14|Diptera|Cecidomyiidae||BOLD:ACN1459
Cecidomyiidae|[6989]|GMARU443-14|Diptera|Cecidomyiidae||BOLD:ACO0846
Cecidomyiidae|[6990]|GMARO959-14|Diptera|Cecidomyiidae||BOLD:ACN4971
Cecidomyiidae|[6991]|GMAGE2554-15|Diptera|Cecidomyiidae||BOLD:ACX6271
Cecidomyiidae|[6992]|GMARS246-14|Diptera|Cecidomyiidae||BOLD:ACX5718
Cecidomyiidae|[6993]|GMAGB558-15|Diptera|Cecidomyiidae||BOLD:ACO1635
Cecidomyiidae|[6994]|GMAGB251-15|Diptera|Cecidomyiidae||BOLD:ACW1350
Cecidomyiidae|[6995]|GMARB1107-14|Diptera|Cecidomyiidae||BOLD:ACM5274
Cecidomyiidae|[6996]|GMARB1115-14|Diptera|Cecidomyiidae||BOLD:ACM5901
Cecidomyiidae|[6997]|GMARB1486-14|Diptera|Cecidomyiidae||BOLD:ACM5055
Cecidomyiidae|[6998]|GMARA1768-14|Diptera|Cecidomyiidae||BOLD:ACM5711
Cecidomyiidae|[6999]|GMAGH745-15|Diptera|Cecidomyiidae||BOLD:ACW3690
Cecidomyiidae|[7000]|GMAGA499-15|Diptera|Cecidomyiidae||BOLD:ACM9331
Cecidomyiidae|[7001]|GMAGB1040-15|Diptera|Cecidomyiidae||BOLD:ACW4031
Cecidomyiidae|[7002]|GMAGB1525-15|Diptera|Cecidomyiidae||BOLD:ACW4709
Cecidomyiidae|[7003]|GMAGN2374-15|Diptera|Cecidomyiidae||BOLD:ACN4352
Cecidomyiidae|[7004]|GMAGS265-15|Diptera|Cecidomyiidae||BOLD:ACX5153
Cecidomyiidae|[7005]|GMART706-14|Diptera|Cecidomyiidae||BOLD:ACN9544
Cecidomyiidae|[7006]|GMARL231-14|Diptera|Cecidomyiidae||BOLD:ACN2190
Cecidomyiidae|[7007]|GMARO587-14|Diptera|Cecidomyiidae||BOLD:ACN9597
Cecidomyiidae|[7008]|GMAGC044-15|Diptera|Cecidomyiidae||BOLD:ACV7560
Cecidomyiidae|[7009]|GMAGR316-15|Diptera|Cecidomyiidae||BOLD:ACN5608
Cecidomyiidae|[7010]|GMAGD592-15|Diptera|Cecidomyiidae||BOLD:ACN9221
Cecidomyiidae|[7011]|GMAGY144-15|Diptera|Cecidomyiidae||BOLD:ACX1984
Cecidomyiidae|[7012]|GMAGD2268-15|Diptera|Cecidomyiidae||BOLD:ACW7993
Cecidomyiidae|[7013]|GMARQ568-14|Diptera|Cecidomyiidae||BOLD:ACN8870
Cecidomyiidae|[7014]|GMAGE2285-15|Diptera|Cecidomyiidae||BOLD:ACN3734
Cecidomyiidae|[7015]|GMAGE1081-15|Diptera|Cecidomyiidae||BOLD:ACW2593
Cecidomyiidae|[7016]|GMAGY220-15|Diptera|Cecidomyiidae||BOLD:ACX3341
Cecidomyiidae|[7017]|GMARA1404-14|Diptera|Cecidomyiidae||BOLD:ACM5840
Cecidomyiidae|[7018]|GMARA367-14|Diptera|Cecidomyiidae||BOLD:ACM9701
Cecidomyiidae|[7019]|GMARB650-14|Diptera|Cecidomyiidae||BOLD:ACM5992
Cecidomyiidae|[7020]|GMAGE2121-15|Diptera|Cecidomyiidae||BOLD:ACW6179
Cecidomyiidae|[7021]|GMARA175-14|Diptera|Cecidomyiidae||BOLD:ACM9286
Cecidomyiidae|[7022]|GMARB902-14|Diptera|Cecidomyiidae||BOLD:ACM5637
Cecidomyiidae|[7023]|GMARB656-14|Diptera|Cecidomyiidae||BOLD:ACM5118
Cecidomyiidae|[7024]|GMAGD1259-15|Diptera|Cecidomyiidae||BOLD:ACW3953
Cecidomyiidae|[7025]|GMAGB252-15|Diptera|Cecidomyiidae||BOLD:ACM6329
Cecidomyiidae|[7026]|GMARL920-14|Diptera|Cecidomyiidae||BOLD:ACN2544
Cecidomyiidae|[7027]|GMARC464-14|Diptera|Cecidomyiidae||BOLD:ACM7869
Cecidomyiidae|[7028]|GMAGB1542-15|Diptera|Cecidomyiidae||BOLD:ACW3820
Cecidomyiidae|[7029]|GMARB1623-14|Diptera|Cecidomyiidae||BOLD:ACM6233
Cecidomyiidae|[7030]|GMARB1359-14|Diptera|Cecidomyiidae||BOLD:ACM5773
Cecidomyiidae|[7031]|GMARP454-14|Diptera|Cecidomyiidae||BOLD:ACO1881
Cecidomyiidae|[7032]|GMARB853-14|Diptera|Cecidomyiidae||BOLD:ACM6032
Cecidomyiidae|[7033]|GMAGG1776-15|Diptera|Cecidomyiidae||BOLD:ACN0045
Cecidomyiidae|[7034]|GMAGC252-15|Diptera|Cecidomyiidae||BOLD:ACW1926
Cecidomyiidae|[7035]|GMARU509-14|Diptera|Cecidomyiidae||BOLD:ACO1117
Cecidomyiidae|[7036]|GMARA146-14|Diptera|Cecidomyiidae||BOLD:ACM8752
Cecidomyiidae|[7037]|GMAGB1507-15|Diptera|Cecidomyiidae||BOLD:ACW1357
Cecidomyiidae|[7038]|GMARD2148-14|Diptera|Cecidomyiidae||BOLD:ACN1373
Cecidomyiidae|[7039]|GMAGG1491-15|Diptera|Cecidomyiidae||BOLD:ACW3234
Cecidomyiidae|[7040]|GMAGP1695-15|Diptera|Cecidomyiidae||BOLD:ACN8984
Cecidomyiidae|[7041]|GMAGB1118-15|Diptera|Cecidomyiidae||BOLD:ACN0395
Cecidomyiidae|[7042]|GMARR743-14|Diptera|Cecidomyiidae||BOLD:ACN8794
Cecidomyiidae|[7043]|GMAGD1244-15|Diptera|Cecidomyiidae||BOLD:ACN8882
Cecidomyiidae|[7044]|GMAGY131-15|Diptera|Cecidomyiidae||BOLD:ACX2139
Cecidomyiidae|[7045]|GMARA1384-14|Diptera|Cecidomyiidae||BOLD:ACM8814
Cecidomyiidae|[7046]|GMARC647-14|Diptera|Cecidomyiidae||BOLD:ACM7704
Cecidomyiidae|[7047]|GMART338-14|Diptera|Cecidomyiidae||BOLD:ACN9376
Cecidomyiidae|[7048]|GMAGY438-15|Diptera|Cecidomyiidae||BOLD:ACX3071
Cecidomyiidae|[7049]|GMARA213-14|Diptera|Cecidomyiidae||BOLD:ACM8367
Cecidomyiidae|[7050]|GMAGB655-15|Diptera|Cecidomyiidae||BOLD:ACW4631
Cecidomyiidae|[7051]|GMARB1245-14|Diptera|Cecidomyiidae||BOLD:ACM5148
Cecidomyiidae|[7052]|GMAGC197-15|Diptera|Cecidomyiidae||BOLD:ACV6531
Cecidomyiidae|[7053]|GMARA1563-14|Diptera|Cecidomyiidae||BOLD:ACM5737
Cecidomyiidae|[7054]|GMARA1274-14|Diptera|Cecidomyiidae||BOLD:ACX0807
Cecidomyiidae|[7055]|GMAGC280-15|Diptera|Cecidomyiidae||BOLD:ACR8209
Cecidomyiidae|[7056]|GMARB1271-14|Diptera|Cecidomyiidae||BOLD:ACM5813
Cecidomyiidae|[7057]|GMAGG2340-15|Diptera|Cecidomyiidae||BOLD:ACW7870
Cecidomyiidae|[7058]|GMARB1637-14|Diptera|Cecidomyiidae||BOLD:ACM5512
Cecidomyiidae|[7059]|GMAGT488-15|Diptera|Cecidomyiidae||BOLD:ACX5427
Cecidomyiidae|[7060]|GMARC761-14|Diptera|Cecidomyiidae||BOLD:ACM6409
Cecidomyiidae|[7061]|GMARB608-14|Diptera|Cecidomyiidae||BOLD:ACM5949
Cecidomyiidae|[7062]|GMAGB408-15|Diptera|Cecidomyiidae||BOLD:ACN1653
Cecidomyiidae|[7063]|GMAGC969-15|Diptera|Cecidomyiidae||BOLD:ACV7980
Cecidomyiidae|[7064]|GMART1108-14|Diptera|Cecidomyiidae||BOLD:ACO0776
Cecidomyiidae|[7065]|GMAGJ839-15|Diptera|Cecidomyiidae||BOLD:ACN9101
Cecidomyiidae|[7066]|GMARA1113-14|Diptera|Cecidomyiidae||BOLD:ACM9290
Cecidomyiidae|[7067]|GMARN1005-14|Diptera|Cecidomyiidae||BOLD:ACN4422
Cecidomyiidae|[7068]|GMAGR707-15|Diptera|Cecidomyiidae||BOLD:ACO2088
Cecidomyiidae|[7069]|GMARO692-14|Diptera|Cecidomyiidae||BOLD:ACN9277

Cecidomyiidae|[7067]|GMARN1005-14|Diptera|Cecidomyiidae||BOLD:ACN4422
Cecidomyiidae|[7068]|GMAGR707-15|Diptera|Cecidomyiidae||BOLD:ACO2088
Cecidomyiidae|[7069]|GMARO692-14|Diptera|Cecidomyiidae||BOLD:ACN9277
Cecidomyiidae|[7070]|GMART567-14|Diptera|Cecidomyiidae||BOLD:ACN9441
Cecidomyiidae|[7071]|GMARA1065-14|Diptera|Cecidomyiidae||BOLD:ACM5799
Cecidomyiidae|[7072]|GMAGB568-15|Diptera|Cecidomyiidae||BOLD:ACN5389
Cecidomyiidae|[7073]|GMAGD779-15|Diptera|Cecidomyiidae||BOLD:ACM8869
Cecidomyiidae|[7074]|GMARC251-14|Diptera|Cecidomyiidae||BOLD:ACM7239
Cecidomyiidae|[7075]|GMARF1025-14|Diptera|Cecidomyiidae||BOLD:ACN1461
Cecidomyiidae|[7076]|GMAGD829-15|Diptera|Cecidomyiidae||BOLD:ACW4941
Cecidomyiidae|[7077]|GMARC314-14|Diptera|Cecidomyiidae||BOLD:ACM7415
Cecidomyiidae|[7078]|GMARN220-14|Diptera|Cecidomyiidae||BOLD:ACN3111
Cecidomyiidae|[7079]|GMAGD884-15|Diptera|Cecidomyiidae||BOLD:ACW3325
Cecidomyiidae|[7080]|GMAGD624-15|Diptera|Cecidomyiidae||BOLD:ACW4553
Cecidomyiidae|[7081]|GMAGK2024-15|Diptera|Cecidomyiidae||BOLD:ACW8753
Cecidomyiidae|[7082]|GMART1046-14|Diptera|Cecidomyiidae||BOLD:ACO0322
Cecidomyiidae|[7083]|GMARA273-14|Diptera|Cecidomyiidae||BOLD:ACM7974
Cecidomyiidae|[7084]|GMAGB646-15|Diptera|Cecidomyiidae||BOLD:ACM9463
Cecidomyiidae|[7085]|GMARJ441-14|Diptera|Cecidomyiidae||BOLD:ACN0207
Cecidomyiidae|[7086]|GMAGC567-15|Diptera|Cecidomyiidae||BOLD:ACW2649
Cecidomyiidae|[7087]|GMARB1544-14|Diptera|Cecidomyiidae||BOLD:ACM4725
Cecidomyiidae|[7088]|GMARR358-14|Diptera|Cecidomyiidae||BOLD:ACN8899
Cecidomyiidae|[7089]|GMAGB711-15|Diptera|Cecidomyiidae||BOLD:ACM6235
Cecidomyiidae|[7090]|GMAGY420-15|Diptera|Cecidomyiidae||BOLD:ACX2682
Cecidomyiidae|[7091]|GMAGA500-15|Diptera|Cecidomyiidae||BOLD:ACN0123
Cecidomyiidae|[7092]|GMARC533-14|Diptera|Cecidomyiidae||BOLD:ACM4732
Cecidomyiidae|[7093]|GMARJ1467-14|Diptera|Cecidomyiidae||BOLD:ACN1277
Cecidomyiidae|[7094]|GMAGZ092-15|Diptera|Cecidomyiidae||BOLD:ACX3876
Cecidomyiidae|[7095]|GMAGS594-15|Diptera|Cecidomyiidae||BOLD:ACX5745
Cecidomyiidae|[7096]|GMAGT560-15|Diptera|Cecidomyiidae||BOLD:ACX6189
Cecidomyiidae|[7097]|GMAGC208-15|Diptera|Cecidomyiidae||BOLD:ACW2054
Cecidomyiidae|[7098]|GMARB1329-14|Diptera|Cecidomyiidae||BOLD:ACM5832
Cecidomyiidae|[7099]|GMARE207-14|Diptera|Cecidomyiidae||BOLD:ACN1615
Cecidomyiidae|[7100]|GMART1257-14|Diptera|Cecidomyiidae||BOLD:ACN9738
Cecidomyiidae|[7101]|GMAGB1390-15|Diptera|Cecidomyiidae||BOLD:ACW3737
Cecidomyiidae|[7102]|GMAGF722-15|Diptera|Cecidomyiidae||BOLD:ACW3299
Cecidomyiidae|[7103]|GMAGY200-15|Diptera|Cecidomyiidae||BOLD:ACX1803
Cecidomyiidae|[7104]|GMAGB523-15|Diptera|Cecidomyiidae||BOLD:ACN0838
Cecidomyiidae|[7105]|GMAGB1431-15|Diptera|Cecidomyiidae||BOLD:ACM4959
Cecidomyiidae|[7106]|GMAGC161-15|Diptera|Cecidomyiidae||BOLD:ACM6168
Cecidomyiidae|[7107]|GMAGB676-15|Diptera|Cecidomyiidae||BOLD:ACW3654
Cecidomyiidae|[7108]|GMAGB1409-15|Diptera|Cecidomyiidae||BOLD:ACO0976
Cecidomyiidae|[7109]|GMARB1483-14|Diptera|Cecidomyiidae||BOLD:ACM5053
Cecidomyiidae|[7110]|GMARL908-14|Diptera|Cecidomyiidae||BOLD:ACN2875
Cecidomyiidae|[7111]|GMAGB778-15|Diptera|Cecidomyiidae||BOLD:ACW7292
Cecidomyiidae|[7112]|GMARK1582-14|Diptera|Cecidomyiidae||BOLD:ACN0716
Cecidomyiidae|[7113]|GMARA1477-14|Diptera|Cecidomyiidae||BOLD:ACM5882
Cecidomyiidae|[7114]|GMAGB1400-15|Diptera|Cecidomyiidae||BOLD:ACW3798
Cecidomyiidae|[7115]|GMARJ772-14|Diptera|Cecidomyiidae||BOLD:ACM9858
Cecidomyiidae|[7116]|GMART1170-14|Diptera|Cecidomyiidae||BOLD:ACO0495
Cecidomyiidae|[7117]|GMAGA424-15|Diptera|Cecidomyiidae||BOLD:ACM5610
Cecidomyiidae|[7118]|GMARC587-14|Diptera|Cecidomyiidae||BOLD:ACM6091
Cecidomyiidae|[7119]|GMAGZ140-15|Diptera|Cecidomyiidae||BOLD:ACX3782
Cecidomyiidae|[7120]|GMARK2012-14|Diptera|Cecidomyiidae||BOLD:ACN0882
Cecidomyiidae|[7121]|GMARP287-14|Diptera|Cecidomyiidae||BOLD:ACN9588
Cecidomyiidae|[7122]|GMAGD897-15|Diptera|Cecidomyiidae||BOLD:ACW4677
Cecidomyiidae|[7123]|GMAGB669-15|Diptera|Cecidomyiidae||BOLD:ACW4213
Cecidomyiidae|[7124]|GMARB1238-14|Diptera|Cecidomyiidae||BOLD:ACM5434
Cecidomyiidae|[7125]|GMAGB1369-15|Diptera|Cecidomyiidae||BOLD:ACN1745
Cecidomyiidae|[7126]|GMAGC151-15|Diptera|Cecidomyiidae||BOLD:ACO1224
Cecidomyiidae|[7127]|GMAGD931-15|Diptera|Cecidomyiidae||BOLD:ACM7528
Cecidomyiidae|[7128]|GMAGE2255-15|Diptera|Cecidomyiidae||BOLD:ACW1687
Cecidomyiidae|[7129]|GMAGE2021-15|Diptera|Cecidomyiidae||BOLD:ACX5240
Cecidomyiidae|[7130]|GMAGC433-15|Diptera|Cecidomyiidae||BOLD:ACW1695
Cecidomyiidae|[7131]|GMAGW074-15|Diptera|Cecidomyiidae||BOLD:ACX1393
Cecidomyiidae|[7132]|GMARA1230-14|Diptera|Cecidomyiidae||BOLD:ACW2249
Cecidomyiidae|[7133]|GMAGC214-15|Diptera|Cecidomyiidae||BOLD:ACW1764
Cecidomyiidae|[7134]|GMAGG1364-15|Diptera|Cecidomyiidae||BOLD:ACW3578
Cecidomyiidae|[7135]|GMAGH675-15|Diptera|Cecidomyiidae||BOLD:ADB3011
Cecidomyiidae|[7136]|GMARB1583-14|Diptera|Cecidomyiidae||BOLD:ACM5800
Cecidomyiidae|[7137]|GMAGI674-15|Diptera|Cecidomyiidae||BOLD:ACW7905
Cecidomyiidae|[7138]|GMAGB448-15|Diptera|Cecidomyiidae||BOLD:ACM5619
Cecidomyiidae|[7139]|GMAGE1042-15|Diptera|Cecidomyiidae||BOLD:ACW1871
Cecidomyiidae|[7140]|GMARB1660-14|Diptera|Cecidomyiidae||BOLD:ACM5595
Cecidomyiidae|[7141]|GMARB1313-14|Diptera|Cecidomyiidae||BOLD:ACM5986
Cecidomyiidae|[7142]|GMAGM1337-15|Diptera|Cecidomyiidae||BOLD:ACX3493
Cecidomyiidae|[7143]|GMARB594-14|Diptera|Cecidomyiidae||BOLD:ACM4933
Cecidomyiidae|[7144]|GMARU110-14|Diptera|Cecidomyiidae||BOLD:ACO0813
Cecidomyiidae|[7145]|GMARD625-14|Diptera|Cecidomyiidae||BOLD:ACN0839
Cecidomyiidae|[7146]|GMAGS337-15|Diptera|Cecidomyiidae||BOLD:ACX6308
Cecidomyiidae|[7147]|GMAGW082-15|Diptera|Cecidomyiidae||BOLD:ACX3081
Cecidomyiidae|[7148]|GMAGU249-15|Diptera|Cecidomyiidae||BOLD:ACX4679
Cecidomyiidae|[7149]|GMAGE1950-15|Diptera|Cecidomyiidae||BOLD:ACO0436
Cecidomyiidae|[7150]|GMAGB1487-15|Diptera|Cecidomyiidae||BOLD:ACN0291
Cecidomyiidae|[7151]|GMART1135-14|Diptera|Cecidomyiidae||BOLD:ACO0612
Cecidomyiidae|[7152]|GMART154-14|Diptera|Cecidomyiidae||BOLD:ACN9586
Cecidomyiidae|[7153]|GMAGI758-15|Diptera|Cecidomyiidae||BOLD:ADB6823
Cecidomyiidae|[7154]|GMARG976-14|Diptera|Cecidomyiidae||BOLD:ACM9959
Cecidomyiidae|[7155]|GMAGK1338-15|Diptera|Cecidomyiidae||BOLD:ACN2772
Cecidomyiidae|[7156]|GMARI583-14|Diptera|Cecidomyiidae||BOLD:ACN0517
Cecidomyiidae|[7157]|GMAGR684-15|Diptera|Cecidomyiidae||BOLD:ACX6237
Cecidomyiidae|[7158]|GMART477-14|Diptera|Cecidomyiidae||BOLD:ACN9385
Cecidomyiidae|[7159]|GMARD480-14|Diptera|Cecidomyiidae||BOLD:ACX6334
Cecidomyiidae|[7160]|GMARB909-14|Diptera|Cecidomyiidae||BOLD:ACM5759
Cecidomyiidae|[7161]|GMARE727-14|Diptera|Cecidomyiidae||BOLD:ACM7140
Cecidomyiidae|[7162]|GMAGB246-15|Diptera|Cecidomyiidae||BOLD:ACM6237
Cecidomyiidae|[7163]|GMAGB583-15|Diptera|Cecidomyiidae||BOLD:ACN8165
Cecidomyiidae|[7164]|GMAGB615-15|Diptera|Cecidomyiidae||BOLD:ACW3598
Cecidomyiidae|[7165]|GMAGC092-15|Diptera|Cecidomyiidae||BOLD:ACV8863
Cecidomyiidae|[7166]|GMAGH215-15|Diptera|Cecidomyiidae||BOLD:ACW1691
Cecidomyiidae|[7167]|GMAGX208-15|Diptera|Cecidomyiidae||BOLD:ACX1544
Cecidomyiidae|[7168]|GMAGG2324-15|Diptera|Cecidomyiidae||BOLD:ACW7671
[illegible]

Cecidomyiidae|[7166]|GMAGH213-15|Diptera|Cecidomyiidae||BOLD:ACW1691
Cecidomyiidae|[7167]|GMAGX208-15|Diptera|Cecidomyiidae||BOLD:ACX1544
Cecidomyiidae|[7168]|GMAGG2324-15|Diptera|Cecidomyiidae||BOLD:ACW7671
Cecidomyiidae|[7169]|GMAGJ2047-15|Diptera|Cecidomyiidae||BOLD:ACX5843
Cecidomyiidae|[7170]|GMARA1542-14|Diptera|Cecidomyiidae||BOLD:ACM6278
Cecidomyiidae|[7171]|GMARB1263-14|Diptera|Cecidomyiidae||BOLD:ACM5144
Cecidomyiidae|[7172]|GMARF030-14|Diptera|Cecidomyiidae||BOLD:ACN1585
Cecidomyiidae|[7173]|GMAGH652-15|Diptera|Cecidomyiidae||BOLD:ACX5329
Cecidomyiidae|[7174]|GMARC498-14|Diptera|Cecidomyiidae||BOLD:ACM7976
Cecidomyiidae|[7175]|GMAGJ1193-15|Diptera|Cecidomyiidae||BOLD:ACX5328
Cecidomyiidae|[7176]|GMAGN2419-15|Diptera|Cecidomyiidae||BOLD:ACX2500
Cecidomyiidae|[7177]|GMART616-14|Diptera|Cecidomyiidae||BOLD:ACN9486
Cecidomyiidae|[7178]|GMARB904-14|Diptera|Cecidomyiidae||BOLD:ACM5758
Cecidomyiidae|[7179]|GMAGD654-15|Diptera|Cecidomyiidae||BOLD:ACN1620
Cecidomyiidae|[7180]|GMAGB822-15|Diptera|Cecidomyiidae||BOLD:ACM7130
Cecidomyiidae|[7181]|GMARA1090-14|Diptera|Cecidomyiidae||BOLD:ACM8121
Cecidomyiidae|[7182]|GMARB1397-14|Diptera|Cecidomyiidae||BOLD:ACM9835
Cecidomyiidae|[7183]|GMAGN1834-15|Diptera|Cecidomyiidae||BOLD:ACX2154
Cecidomyiidae|[7184]|GMAGX494-15|Diptera|Cecidomyiidae||BOLD:ACX4202
Cecidomyiidae|[7185]|GMARA398-14|Diptera|Cecidomyiidae||BOLD:ACM5155
Cecidomyiidae|[7186]|GMARR728-14|Diptera|Cecidomyiidae||BOLD:ACN8938
Cecidomyiidae|[7187]|GMAGY397-15|Diptera|Cecidomyiidae||BOLD:ACX2182
Cecidomyiidae|[7188]|GMAGY367-15|Diptera|Cecidomyiidae||BOLD:ACX2195
Cecidomyiidae|[7189]|GMAGH528-15|Diptera|Cecidomyiidae||BOLD:ACN1122
Cecidomyiidae|[7190]|GMAGL1713-15|Diptera|Cecidomyiidae||BOLD:ACW6336
Cecidomyiidae|[7191]|GMAGB785-15|Diptera|Cecidomyiidae||BOLD:ACW7258
Cecidomyiidae|[7192]|GMAGC210-15|Diptera|Cecidomyiidae||BOLD:ACM5438
Cecidomyiidae|[7193]|GMARJ201-14|Diptera|Cecidomyiidae||BOLD:ACN3566
Cecidomyiidae|[7194]|GMARK1581-14|Diptera|Cecidomyiidae||BOLD:ACN1172
Cecidomyiidae|[7195]|GMARA1421-14|Diptera|Cecidomyiidae||BOLD:ACM6241
Cecidomyiidae|[7196]|GMARO100-14|Diptera|Cecidomyiidae||BOLD:ACN9080
Cecidomyiidae|[7197]|GMARB998-14|Diptera|Cecidomyiidae||BOLD:ACM4792
Cecidomyiidae|[7198]|GMAGB366-15|Diptera|Cecidomyiidae||BOLD:ACW4743
Cecidomyiidae|[7199]|GMAGD1233-15|Diptera|Cecidomyiidae||BOLD:ACW4258
Cecidomyiidae|[7200]|GMAGB674-15|Diptera|Cecidomyiidae||BOLD:ACM9487
Cecidomyiidae|[7201]|GMAGE2212-15|Diptera|Cecidomyiidae||BOLD:ACW7743
Cecidomyiidae|[7202]|GMAGL2472-15|Diptera|Cecidomyiidae||BOLD:ACN3188
Cecidomyiidae|[7203]|GMARC133-14|Diptera|Cecidomyiidae||BOLD:ACM8066
Cecidomyiidae|[7204]|GMAGK1192-15|Diptera|Cecidomyiidae||BOLD:ACW9244
Cecidomyiidae|[7205]|GMAGB652-15|Diptera|Cecidomyiidae||BOLD:ACM7364
Cecidomyiidae|[7206]|GMAGD2003-15|Diptera|Cecidomyiidae||BOLD:ACM9161
Cecidomyiidae|[7207]|GMAGO212-15|Diptera|Cecidomyiidae||BOLD:ACX4501
Cecidomyiidae|[7208]|GMAGB1516-15|Diptera|Cecidomyiidae||BOLD:ACW4464
Cecidomyiidae|[7209]|GMAGH1293-15|Diptera|Cecidomyiidae||BOLD:ACN3843
Cecidomyiidae|[7210]|GMAGL2044-15|Diptera|Cecidomyiidae||BOLD:ACN8601
Cecidomyiidae|[7211]|GMARO941-14|Diptera|Cecidomyiidae||BOLD:ACN8587
Cecidomyiidae|[7212]|GMAGG1756-15|Diptera|Cecidomyiidae||BOLD:ACW6577
Cecidomyiidae|[7213]|GMARB830-14|Diptera|Cecidomyiidae||BOLD:ACM4947
Cecidomyiidae|[7214]|GMAGG1275-15|Diptera|Cecidomyiidae||BOLD:ACW2835
Cecidomyiidae|[7215]|GMAGB287-15|Diptera|Cecidomyiidae||BOLD:ACM5232
Cecidomyiidae|[7216]|GMAGS959-15|Diptera|Cecidomyiidae||BOLD:ACX5335
Cecidomyiidae|[7217]|GMAGC282-15|Diptera|Cecidomyiidae||BOLD:ACW2370
Cecidomyiidae|[7218]|GMARB1358-14|Diptera|Cecidomyiidae||BOLD:ACM5768
Cecidomyiidae|[7219]|GMAGA594-15|Diptera|Cecidomyiidae||BOLD:ACM6397
Cecidomyiidae|[7220]|GMAGG2316-15|Diptera|Cecidomyiidae||BOLD:ACM7876
Cecidomyiidae|[7221]|GMARA1407-14|Diptera|Cecidomyiidae||BOLD:ACM5765
Cecidomyiidae|[7222]|GMARB1485-14|Diptera|Cecidomyiidae||BOLD:ACM5054
Cecidomyiidae|[7223]|GMARV058-14|Diptera|Cecidomyiidae||BOLD:ACO0899
Cecidomyiidae|[7224]|GMAGD2009-15|Diptera|Cecidomyiidae||BOLD:ACM9016
Cecidomyiidae|[7225]|GMAGD2192-15|Diptera|Cecidomyiidae||BOLD:ACW2328
Cecidomyiidae|[7226]|GMARH610-14|Diptera|Cecidomyiidae||BOLD:ACN1444
Cecidomyiidae|[7227]|GMARD1031-14|Diptera|Cecidomyiidae||BOLD:ACM8541
Cecidomyiidae|[7228]|GMAGB637-15|Diptera|Cecidomyiidae||BOLD:ACW3370
Cecidomyiidae|[7229]|GMARL1366-14|Diptera|Cecidomyiidae||BOLD:ACN2481
Cecidomyiidae|[7230]|GMART596-14|Diptera|Cecidomyiidae||BOLD:ACN9077
Cecidomyiidae|[7231]|GMARB820-14|Diptera|Cecidomyiidae||BOLD:ACM4942
Cecidomyiidae|[7232]|GMARN819-14|Diptera|Cecidomyiidae||BOLD:ACN7424
Cecidomyiidae|[7233]|GMARD1178-14|Diptera|Cecidomyiidae||BOLD:ACM9140
Cecidomyiidae|[7234]|GMARN1494-14|Diptera|Cecidomyiidae||BOLD:ACN8381
Cecidomyiidae|[7235]|GMARB938-14|Diptera|Cecidomyiidae||BOLD:ACM4740
Cecidomyiidae|[7236]|GMAGL1702-15|Diptera|Cecidomyiidae||BOLD:ACN6458
Cecidomyiidae|[7237]|GMARB787-14|Diptera|Cecidomyiidae||BOLD:ACM5622
Cecidomyiidae|[7238]|GMAGR368-15|Diptera|Cecidomyiidae||BOLD:ACX6523
Cecidomyiidae|[7239]|GMARB1312-14|Diptera|Cecidomyiidae||BOLD:ACM4975
Cecidomyiidae|[7240]|GMART903-14|Diptera|Cecidomyiidae||BOLD:ACN9701
Cecidomyiidae|[7241]|GMAGX549-15|Diptera|Cecidomyiidae||BOLD:ACX4160
Cecidomyiidae|[7242]|GMAGD2100-15|Diptera|Cecidomyiidae||BOLD:ACW2427
Cecidomyiidae|[7243]|GMAGN2258-15|Diptera|Cecidomyiidae||BOLD:ACX2189
Cecidomyiidae|[7244]|GMARL1123-14|Diptera|Cecidomyiidae||BOLD:ACN2766
Cecidomyiidae|[7245]|GMAGY159-15|Diptera|Cecidomyiidae||BOLD:ACX3005
Cecidomyiidae|[7246]|GMARW044-15|Diptera|Cecidomyiidae||BOLD:ACX3653
Cecidomyiidae|[7247]|GMARA1458-14|Diptera|Cecidomyiidae||BOLD:ACM5181
Cecidomyiidae|[7248]|GMARA1525-14|Diptera|Cecidomyiidae||BOLD:ACM6522
Cecidomyiidae|[7249]|GMAGE2565-15|Diptera|Cecidomyiidae||BOLD:ACW7475
Cecidomyiidae|[7250]|GMAGE2584-15|Diptera|Cecidomyiidae||BOLD:ACW8013
Cecidomyiidae|[7251]|GMARR355-14|Diptera|Cecidomyiidae||BOLD:ACN8120
Cecidomyiidae|[7252]|GMAGB830-15|Diptera|Cecidomyiidae||BOLD:ACW6837
Cecidomyiidae|[7253]|GMAGB806-15|Diptera|Cecidomyiidae||BOLD:ACW6735
Cecidomyiidae|[7254]|GMAGE2108-15|Diptera|Cecidomyiidae||BOLD:ACW6503
Cecidomyiidae|[7255]|GMAGF416-15|Diptera|Cecidomyiidae||BOLD:ACW3293
Cecidomyiidae|[7256]|GMARF258-14|Diptera|Cecidomyiidae||BOLD:ACM9650
Cecidomyiidae|[7257]|GMARA1433-14|Diptera|Cecidomyiidae||BOLD:ACM9383
Cecidomyiidae|[7258]|GMAGO229-15|Diptera|Cecidomyiidae||BOLD:ACX5502
Cecidomyiidae|[7259]|GMAGD2179-15|Diptera|Cecidomyiidae||BOLD:ACW2032
Cecidomyiidae|[7260]|GMAGB725-15|Diptera|Cecidomyiidae||BOLD:ACN8722
Cecidomyiidae|[7261]|GMAGA471-15|Diptera|Cecidomyiidae||BOLD:ACM6215
Cecidomyiidae|[7262]|GMAGE2473-15|Diptera|Cecidomyiidae||BOLD:ACN8405
Cecidomyiidae|[7263]|GMAGW272-15|Diptera|Cecidomyiidae||BOLD:ACX2973
Cecidomyiidae|[7264]|GMAGB585-15|Diptera|Cecidomyiidae||BOLD:ACW3990
Cecidomyiidae|[7265]|GMARF844-14|Diptera|Cecidomyiidae||BOLD:ACM9668
Cecidomyiidae|[7266]|GMAGK1417-15|Diptera|Cecidomyiidae||BOLD:ACN7748
Cecidomyiidae|[7267]|GMARU436-14|Diptera|Cecidomyiidae||BOLD:ACO0790
Cecidomyiidae|[7268]|GMAGA454-15|Diptera|Cecidomyiidae||BOLD:ACN9848

Cecidomyiidae|[7266]|GMAGK1417-15|Diptera|Cecidomyiidae||BOLD:ACN7748
Cecidomyiidae|[7267]|GMARU436-14|Diptera|Cecidomyiidae||BOLD:ACO0790
Cecidomyiidae|[7268]|GMAGA454-15|Diptera|Cecidomyiidae||BOLD:ACN9848
Cecidomyiidae|[7269]|GMAGB1483-15|Diptera|Cecidomyiidae||BOLD:ACN9687
Cecidomyiidae|[7270]|GMARB742-14|Diptera|Cecidomyiidae||BOLD:ACM5481
Cecidomyiidae|[7271]|GMARA394-14|Diptera|Cecidomyiidae||BOLD:ACM8462
Cecidomyiidae|[7272]|GMAGB279-15|Diptera|Cecidomyiidae||BOLD:ACW2446
Cecidomyiidae|[7273]|GMAGT591-15|Diptera|Cecidomyiidae||BOLD:ACN7491
Cecidomyiidae|[7274]|GMAGE2026-15|Diptera|Cecidomyiidae||BOLD:ACN9331
Cecidomyiidae|[7275]|GMAGE2262-15|Diptera|Cecidomyiidae||BOLD:ACN0459
Cecidomyiidae|[7276]|GMARC812-14|Diptera|Cecidomyiidae||BOLD:ACO0362
Cecidomyiidae|[7277]|GMAGI1243-15|Diptera|Cecidomyiidae||BOLD:ACW3625
Cecidomyiidae|[7278]|GMAGB1131-15|Diptera|Cecidomyiidae||BOLD:ACM7417
Cecidomyiidae|[7279]|GMAGI974-15|Diptera|Cecidomyiidae||BOLD:ACN7576
Cecidomyiidae|[7280]|GMAGQ1531-15|Diptera|Cecidomyiidae||BOLD:ACX5176
Cecidomyiidae|[7281]|GMAGD2283-15|Diptera|Cecidomyiidae||BOLD:ACN8364
Cecidomyiidae|[7282]|GMAGN1627-15|Diptera|Cecidomyiidae||BOLD:ACN2012
Cecidomyiidae|[7283]|GMAGB1550-15|Diptera|Cecidomyiidae||BOLD:ACX4029
Cecidomyiidae|[7284]|GMAGD1071-15|Diptera|Cecidomyiidae||BOLD:ACM9168
Cecidomyiidae|[7285]|GMARF436-14|Diptera|Cecidomyiidae||BOLD:ACM8854
Cecidomyiidae|[7286]|GMAGC216-15|Diptera|Cecidomyiidae||BOLD:ACN0691
Cecidomyiidae|[7287]|GMAGD994-15|Diptera|Cecidomyiidae||BOLD:ACW9644
Cecidomyiidae|[7288]|GMAGG1358-15|Diptera|Cecidomyiidae||BOLD:ACN7640
Cecidomyiidae|[7289]|GMAGB1385-15|Diptera|Cecidomyiidae||BOLD:ACN9220
Cecidomyiidae|[7290]|GMAGG2303-15|Diptera|Cecidomyiidae||BOLD:ACW6611
Cecidomyiidae|[7291]|GMARI2161-14|Diptera|Cecidomyiidae||BOLD:ACN0548
Cecidomyiidae|[7292]|GMAGJ1192-15|Diptera|Cecidomyiidae||BOLD:ACN1434
Cecidomyiidae|[7293]|GMAGP283-15|Diptera|Cecidomyiidae||BOLD:ACN7508
Cecidomyiidae|[7294]|GMARA1349-14|Diptera|Cecidomyiidae||BOLD:ACM8461
Cecidomyiidae|[7295]|GMAGB793-15|Diptera|Cecidomyiidae||BOLD:ACM7439
Cecidomyiidae|[7296]|GMARB1736-14|Diptera|Cecidomyiidae||BOLD:ACM4982
Cecidomyiidae|[7297]|GMARC139-14|Diptera|Cecidomyiidae||BOLD:ACM7630
Cecidomyiidae|[7298]|GMAGH697-15|Diptera|Cecidomyiidae||BOLD:ACW4104
Cecidomyiidae|[7299]|GMARF422-14|Diptera|Cecidomyiidae||BOLD:ACM9574
Cecidomyiidae|[7300]|GMAGG2264-15|Diptera|Cecidomyiidae||BOLD:ACN9089
Cecidomyiidae|[7301]|GMARN1416-14|Diptera|Cecidomyiidae||BOLD:ACN4629
Cecidomyiidae|[7302]|GMAGU625-15|Diptera|Cecidomyiidae||BOLD:ACX4229
Cecidomyiidae|[7303]|GMART989-14|Diptera|Cecidomyiidae||BOLD:ACN9659
Cecidomyiidae|[7304]|GMART202-14|Diptera|Cecidomyiidae||BOLD:ACN9166
Cecidomyiidae|[7305]|GMARD1185-14|Diptera|Cecidomyiidae||BOLD:ACM8837
Cecidomyiidae|[7306]|GMART756-14|Diptera|Cecidomyiidae||BOLD:ACO0380
Cecidomyiidae|[7307]|GMAGB1106-15|Diptera|Cecidomyiidae||BOLD:ACN8416
Cecidomyiidae|[7308]|GMAGB1436-15|Diptera|Cecidomyiidae||BOLD:ACN0311
Cecidomyiidae|[7309]|GMART741-14|Diptera|Cecidomyiidae||BOLD:ACN9033
Cecidomyiidae|[7310]|GMAGD1348-15|Diptera|Cecidomyiidae||BOLD:ACM7121
Cecidomyiidae|[7311]|GMARD1514-14|Diptera|Cecidomyiidae||BOLD:ACN1789
Cecidomyiidae|[7312]|GMAGR483-15|Diptera|Cecidomyiidae||BOLD:ACN8994
Cecidomyiidae|[7313]|GMARA1106-14|Diptera|Cecidomyiidae||BOLD:ACM9747
Cecidomyiidae|[7314]|GMARB826-14|Diptera|Cecidomyiidae||BOLD:ACM6363
Cecidomyiidae|[7315]|GMAGP1425-15|Diptera|Cecidomyiidae||BOLD:ACN9549
Cecidomyiidae|[7316]|GMAGT868-15|Diptera|Cecidomyiidae||BOLD:ACX1408
Cecidomyiidae|[7317]|GMARQ384-14|Diptera|Cecidomyiidae||BOLD:ACN8239
Cecidomyiidae|[7318]|GMARB780-14|Diptera|Cecidomyiidae||BOLD:ACM5649
Cecidomyiidae|[7319]|GMAGC390-15|Diptera|Cecidomyiidae||BOLD:ACO0592
Cecidomyiidae|[7320]|GMAGN1760-15|Diptera|Cecidomyiidae||BOLD:ACN8831
Cecidomyiidae|[7321]|GMAGA586-15|Diptera|Cecidomyiidae||BOLD:ACN1563
Cecidomyiidae|[7322]|GMAGH1163-15|Diptera|Cecidomyiidae||BOLD:ACW3969
Cecidomyiidae|[7323]|GMARM260-14|Diptera|Cecidomyiidae||BOLD:ACN2039
Cecidomyiidae|[7324]|GMAGA435-15|Diptera|Cecidomyiidae||BOLD:ACN7672
Cecidomyiidae|[7325]|GMAGD2253-15|Diptera|Cecidomyiidae||BOLD:ACW6126
Cecidomyiidae|[7326]|GMARD1272-14|Diptera|Cecidomyiidae||BOLD:ACM9764
Cecidomyiidae|[7327]|GMAGD2144-15|Diptera|Cecidomyiidae||BOLD:ACN8014
Cecidomyiidae|[7328]|GMARC658-14|Diptera|Cecidomyiidae||BOLD:ACM7501
Cecidomyiidae|[7329]|GMARP394-14|Diptera|Cecidomyiidae||BOLD:ACO2245
Cecidomyiidae|[7330]|GMAGG2047-15|Diptera|Cecidomyiidae||BOLD:ACW7824
Cecidomyiidae|[7331]|GMART1596-14|Diptera|Cecidomyiidae||BOLD:ACO0185
Cecidomyiidae|[7332]|GMAGD2156-15|Diptera|Cecidomyiidae||BOLD:ACN2162
Cecidomyiidae|[7333]|GMAGD1108-15|Diptera|Cecidomyiidae||BOLD:ACN8081
Cecidomyiidae|[7334]|GMARG433-14|Diptera|Cecidomyiidae||BOLD:ACN0824
Cecidomyiidae|[7335]|GMAGD2104-15|Diptera|Cecidomyiidae||BOLD:ACN8192
Cecidomyiidae|[7336]|GMART1583-14|Diptera|Cecidomyiidae||BOLD:ACN9641
Cecidomyiidae|[7337]|GMARA1468-14|Diptera|Cecidomyiidae||BOLD:ACM5179
Cecidomyiidae|[7338]|GMARQ514-14|Diptera|Cecidomyiidae||BOLD:ACN8555
Cecidomyiidae|[7339]|GMAGD1309-15|Diptera|Cecidomyiidae||BOLD:ACW3656
Cecidomyiidae|[7340]|GMAGD1006-15|Diptera|Cecidomyiidae||BOLD:ACW4521
Cecidomyiidae|[7341]|GMARD1292-14|Diptera|Cecidomyiidae||BOLD:ACM9623
Cecidomyiidae|[7342]|GMARQ165-14|Diptera|Cecidomyiidae||BOLD:ACN8148
Cecidomyiidae|[7343]|GMARG1545-14|Diptera|Cecidomyiidae||BOLD:ACN1803
Cecidomyiidae|[7344]|GMARG1489-14|Diptera|Cecidomyiidae||BOLD:ACN1544
Cecidomyiidae|[7345]|GMAGK1795-15|Diptera|Cecidomyiidae||BOLD:ACW8093
Cecidomyiidae|[7346]|GMARL1029-14|Diptera|Cecidomyiidae||BOLD:ACN1971
Cecidomyiidae|[7347]|GMARP127-14|Diptera|Cecidomyiidae||BOLD:ACN9731
Cecidomyiidae|[7348]|GMARB1015-14|Diptera|Cecidomyiidae||BOLD:ACM9825
Cecidomyiidae|[7349]|GMAGH1185-15|Diptera|Cecidomyiidae||BOLD:ACW4307
Cecidomyiidae|[7350]|GMARP1042-14|Diptera|Cecidomyiidae||BOLD:ACO1633
Cecidomyiidae|[7351]|GMAGA493-15|Diptera|Cecidomyiidae||BOLD:ACM9648
Cecidomyiidae|[7352]|GMARB843-14|Diptera|Cecidomyiidae||BOLD:ACM4818
Cecidomyiidae|[7353]|GMAGH1284-15|Diptera|Cecidomyiidae||BOLD:ACX0021
Cecidomyiidae|[7354]|GMAGE2514-15|Diptera|Cecidomyiidae||BOLD:ACO1583
Cecidomyiidae|[7355]|GMARD553-14|Diptera|Cecidomyiidae||BOLD:ACN1554
Cecidomyiidae|[7356]|GMARA1767-14|Diptera|Cecidomyiidae||BOLD:ACM5708
Cecidomyiidae|[7357]|GMAGH1170-15|Diptera|Cecidomyiidae||BOLD:ACW4039
Cecidomyiidae|[7358]|GMARO980-14|Diptera|Cecidomyiidae||BOLD:ACN8853
Cecidomyiidae|[7359]|GMARB1401-14|Diptera|Cecidomyiidae||BOLD:ACM6360
Cecidomyiidae|[7360]|GMAGD2055-15|Diptera|Cecidomyiidae||BOLD:ACN9846
Cecidomyiidae|[7361]|GMAGE2186-15|Diptera|Cecidomyiidae||BOLD:ACW7932
Cecidomyiidae|[7362]|GMAGA483-15|Diptera|Cecidomyiidae||BOLD:ACM7416
Cecidomyiidae|[7363]|GMARR614-14|Diptera|Cecidomyiidae||BOLD:ACN9722
Cecidomyiidae|[7364]|GMAGV571-15|Diptera|Cecidomyiidae||BOLD:ACX2235
Cecidomyiidae|[7365]|GMAGC993-15|Diptera|Cecidomyiidae||BOLD:ACW0076
Cecidomyiidae|[7366]|GMAGB1443-15|Diptera|Cecidomyiidae||BOLD:ACO3367
Cecidomyiidae|[7367]|GMAGG1904-15|Diptera|Cecidomyiidae||BOLD:ACW7819
Cecidomyiidae|[7368]|GMARU543-14|Diptera|Cecidomyiidae||BOLD:ACO1273

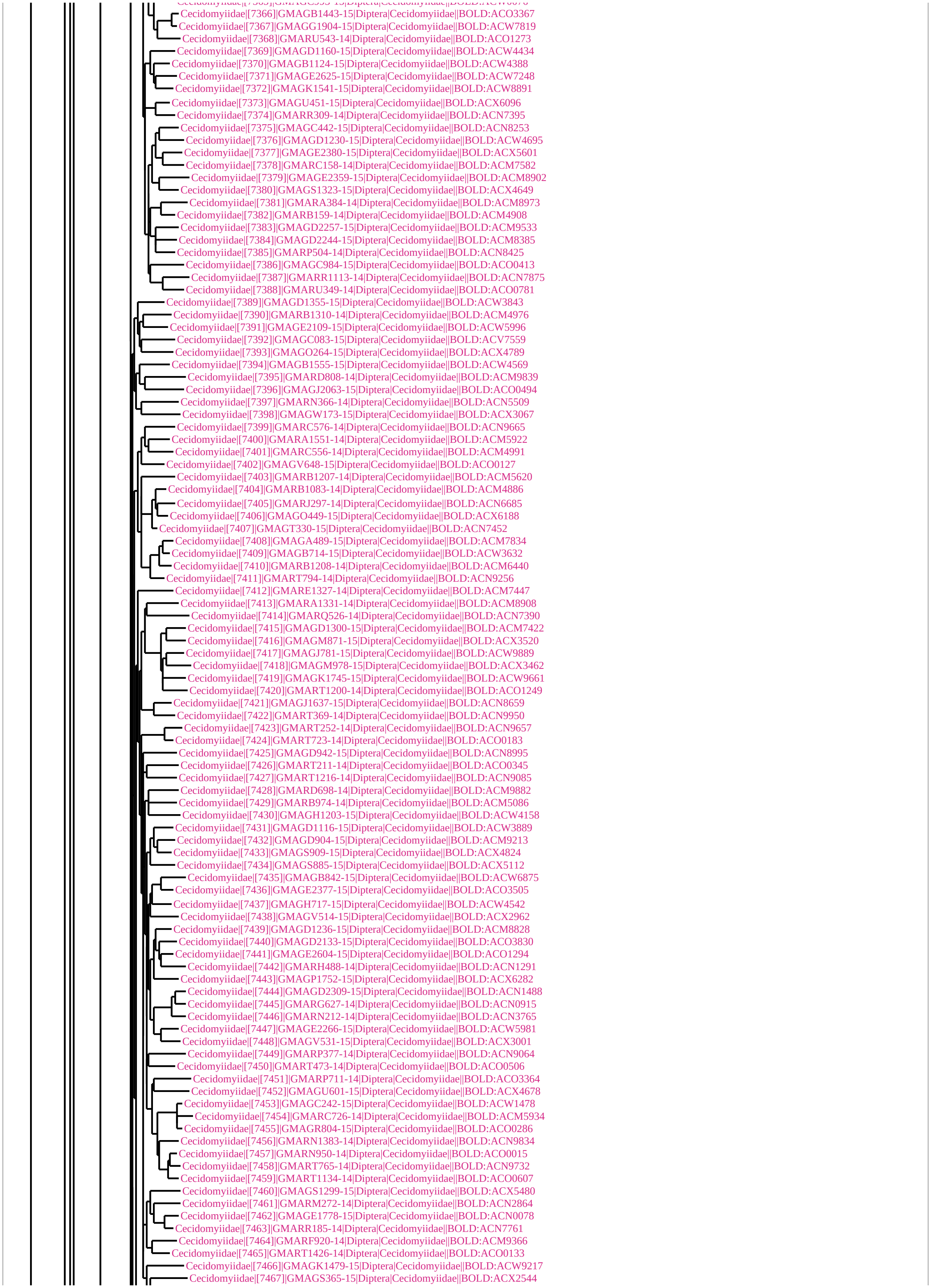

Cecidomyiidae|[illegible]|Diptera|Cecidomyiidae||BOLD:[illegible]
Cecidomyiidae|[7366]|GMAGB1443-15|Diptera|Cecidomyiidae||BOLD:ACO3367
Cecidomyiidae|[7367]|GMAGG1904-15|Diptera|Cecidomyiidae||BOLD:ACW7819
Cecidomyiidae|[7368]|GMARU543-14|Diptera|Cecidomyiidae||BOLD:ACO1273
Cecidomyiidae|[7369]|GMAGD1160-15|Diptera|Cecidomyiidae||BOLD:ACW4434
Cecidomyiidae|[7370]|GMAGB1124-15|Diptera|Cecidomyiidae||BOLD:ACW4388
Cecidomyiidae|[7371]|GMAGE2625-15|Diptera|Cecidomyiidae||BOLD:ACW7248
Cecidomyiidae|[7372]|GMAGK1541-15|Diptera|Cecidomyiidae||BOLD:ACW8891
Cecidomyiidae|[7373]|GMAGU451-15|Diptera|Cecidomyiidae||BOLD:ACX6096
Cecidomyiidae|[7374]|GMARR309-14|Diptera|Cecidomyiidae||BOLD:ACN7395
Cecidomyiidae|[7375]|GMAGC442-15|Diptera|Cecidomyiidae||BOLD:ACN8253
Cecidomyiidae|[7376]|GMAGD1230-15|Diptera|Cecidomyiidae||BOLD:ACW4695
Cecidomyiidae|[7377]|GMAGE2380-15|Diptera|Cecidomyiidae||BOLD:ACX5601
Cecidomyiidae|[7378]|GMARC158-14|Diptera|Cecidomyiidae||BOLD:ACM7582
Cecidomyiidae|[7379]|GMAGE2359-15|Diptera|Cecidomyiidae||BOLD:ACM8902
Cecidomyiidae|[7380]|GMAGS1323-15|Diptera|Cecidomyiidae||BOLD:ACX4649
Cecidomyiidae|[7381]|GMARA384-14|Diptera|Cecidomyiidae||BOLD:ACM8973
Cecidomyiidae|[7382]|GMARB159-14|Diptera|Cecidomyiidae||BOLD:ACM4908
Cecidomyiidae|[7383]|GMAGD2257-15|Diptera|Cecidomyiidae||BOLD:ACM9533
Cecidomyiidae|[7384]|GMAGD2244-15|Diptera|Cecidomyiidae||BOLD:ACM8385
Cecidomyiidae|[7385]|GMARP504-14|Diptera|Cecidomyiidae||BOLD:ACN8425
Cecidomyiidae|[7386]|GMAGC984-15|Diptera|Cecidomyiidae||BOLD:ACO0413
Cecidomyiidae|[7387]|GMARR1113-14|Diptera|Cecidomyiidae||BOLD:ACN7875
Cecidomyiidae|[7388]|GMARU349-14|Diptera|Cecidomyiidae||BOLD:ACO0781
Cecidomyiidae|[7389]|GMAGD1355-15|Diptera|Cecidomyiidae||BOLD:ACW3843
Cecidomyiidae|[7390]|GMARB1310-14|Diptera|Cecidomyiidae||BOLD:ACM4976
Cecidomyiidae|[7391]|GMAGE2109-15|Diptera|Cecidomyiidae||BOLD:ACW5996
Cecidomyiidae|[7392]|GMAGC083-15|Diptera|Cecidomyiidae||BOLD:ACV7559
Cecidomyiidae|[7393]|GMAGO264-15|Diptera|Cecidomyiidae||BOLD:ACX4789
Cecidomyiidae|[7394]|GMAGB1555-15|Diptera|Cecidomyiidae||BOLD:ACW4569
Cecidomyiidae|[7395]|GMARD808-14|Diptera|Cecidomyiidae||BOLD:ACM9839
Cecidomyiidae|[7396]|GMAGJ2063-15|Diptera|Cecidomyiidae||BOLD:ACO0494
Cecidomyiidae|[7397]|GMARN366-14|Diptera|Cecidomyiidae||BOLD:ACN5509
Cecidomyiidae|[7398]|GMAGW173-15|Diptera|Cecidomyiidae||BOLD:ACX3067
Cecidomyiidae|[7399]|GMARC576-14|Diptera|Cecidomyiidae||BOLD:ACN9665
Cecidomyiidae|[7400]|GMARA1551-14|Diptera|Cecidomyiidae||BOLD:ACM5922
Cecidomyiidae|[7401]|GMARC556-14|Diptera|Cecidomyiidae||BOLD:ACM4991
Cecidomyiidae|[7402]|GMAGV648-15|Diptera|Cecidomyiidae||BOLD:ACO0127
Cecidomyiidae|[7403]|GMARB1207-14|Diptera|Cecidomyiidae||BOLD:ACM5620
Cecidomyiidae|[7404]|GMARB1083-14|Diptera|Cecidomyiidae||BOLD:ACM4886
Cecidomyiidae|[7405]|GMARJ297-14|Diptera|Cecidomyiidae||BOLD:ACN6685
Cecidomyiidae|[7406]|GMAGO449-15|Diptera|Cecidomyiidae||BOLD:ACX6188
Cecidomyiidae|[7407]|GMAGT330-15|Diptera|Cecidomyiidae||BOLD:ACN7452
Cecidomyiidae|[7408]|GMAGA489-15|Diptera|Cecidomyiidae||BOLD:ACM7834
Cecidomyiidae|[7409]|GMAGB714-15|Diptera|Cecidomyiidae||BOLD:ACW3632
Cecidomyiidae|[7410]|GMARB1208-14|Diptera|Cecidomyiidae||BOLD:ACM6440
Cecidomyiidae|[7411]|GMART794-14|Diptera|Cecidomyiidae||BOLD:ACN9256
Cecidomyiidae|[7412]|GMARE1327-14|Diptera|Cecidomyiidae||BOLD:ACM7447
Cecidomyiidae|[7413]|GMARA1331-14|Diptera|Cecidomyiidae||BOLD:ACM8908
Cecidomyiidae|[7414]|GMARQ526-14|Diptera|Cecidomyiidae||BOLD:ACN7390
Cecidomyiidae|[7415]|GMAGD1300-15|Diptera|Cecidomyiidae||BOLD:ACM7422
Cecidomyiidae|[7416]|GMAGM871-15|Diptera|Cecidomyiidae||BOLD:ACX3520
Cecidomyiidae|[7417]|GMAGJ781-15|Diptera|Cecidomyiidae||BOLD:ACW9889
Cecidomyiidae|[7418]|GMAGM978-15|Diptera|Cecidomyiidae||BOLD:ACX3462
Cecidomyiidae|[7419]|GMAGK1745-15|Diptera|Cecidomyiidae||BOLD:ACW9661
Cecidomyiidae|[7420]|GMART1200-14|Diptera|Cecidomyiidae||BOLD:ACO1249
Cecidomyiidae|[7421]|GMAGJ1637-15|Diptera|Cecidomyiidae||BOLD:ACN8659
Cecidomyiidae|[7422]|GMART369-14|Diptera|Cecidomyiidae||BOLD:ACN9950
Cecidomyiidae|[7423]|GMART252-14|Diptera|Cecidomyiidae||BOLD:ACN9657
Cecidomyiidae|[7424]|GMART723-14|Diptera|Cecidomyiidae||BOLD:ACO0183
Cecidomyiidae|[7425]|GMAGD942-15|Diptera|Cecidomyiidae||BOLD:ACN8995
Cecidomyiidae|[7426]|GMART211-14|Diptera|Cecidomyiidae||BOLD:ACO0345
Cecidomyiidae|[7427]|GMART1216-14|Diptera|Cecidomyiidae||BOLD:ACN9085
Cecidomyiidae|[7428]|GMARD698-14|Diptera|Cecidomyiidae||BOLD:ACM9882
Cecidomyiidae|[7429]|GMARB974-14|Diptera|Cecidomyiidae||BOLD:ACM5086
Cecidomyiidae|[7430]|GMAGH1203-15|Diptera|Cecidomyiidae||BOLD:ACW4158
Cecidomyiidae|[7431]|GMAGD1116-15|Diptera|Cecidomyiidae||BOLD:ACW3889
Cecidomyiidae|[7432]|GMAGD904-15|Diptera|Cecidomyiidae||BOLD:ACM9213
Cecidomyiidae|[7433]|GMAGS909-15|Diptera|Cecidomyiidae||BOLD:ACX4824
Cecidomyiidae|[7434]|GMAGS885-15|Diptera|Cecidomyiidae||BOLD:ACX5112
Cecidomyiidae|[7435]|GMAGB842-15|Diptera|Cecidomyiidae||BOLD:ACW6875
Cecidomyiidae|[7436]|GMAGE2377-15|Diptera|Cecidomyiidae||BOLD:ACO3505
Cecidomyiidae|[7437]|GMAGH717-15|Diptera|Cecidomyiidae||BOLD:ACW4542
Cecidomyiidae|[7438]|GMAGV514-15|Diptera|Cecidomyiidae||BOLD:ACX2962
Cecidomyiidae|[7439]|GMAGD1236-15|Diptera|Cecidomyiidae||BOLD:ACM8828
Cecidomyiidae|[7440]|GMAGD2133-15|Diptera|Cecidomyiidae||BOLD:ACO3830
Cecidomyiidae|[7441]|GMAGE2604-15|Diptera|Cecidomyiidae||BOLD:ACO1294
Cecidomyiidae|[7442]|GMARH488-14|Diptera|Cecidomyiidae||BOLD:ACN1291
Cecidomyiidae|[7443]|GMAGP1752-15|Diptera|Cecidomyiidae||BOLD:ACX6282
Cecidomyiidae|[7444]|GMAGD2309-15|Diptera|Cecidomyiidae||BOLD:ACN1488
Cecidomyiidae|[7445]|GMARG627-14|Diptera|Cecidomyiidae||BOLD:ACN0915
Cecidomyiidae|[7446]|GMARN212-14|Diptera|Cecidomyiidae||BOLD:ACN3765
Cecidomyiidae|[7447]|GMAGE2266-15|Diptera|Cecidomyiidae||BOLD:ACW5981
Cecidomyiidae|[7448]|GMAGV531-15|Diptera|Cecidomyiidae||BOLD:ACX3001
Cecidomyiidae|[7449]|GMARP377-14|Diptera|Cecidomyiidae||BOLD:ACN9064
Cecidomyiidae|[7450]|GMART473-14|Diptera|Cecidomyiidae||BOLD:ACO0506
Cecidomyiidae|[7451]|GMARP711-14|Diptera|Cecidomyiidae||BOLD:ACO3364
Cecidomyiidae|[7452]|GMAGU601-15|Diptera|Cecidomyiidae||BOLD:ACX4678
Cecidomyiidae|[7453]|GMAGC242-15|Diptera|Cecidomyiidae||BOLD:ACW1478
Cecidomyiidae|[7454]|GMARC726-14|Diptera|Cecidomyiidae||BOLD:ACM5934
Cecidomyiidae|[7455]|GMAGR804-15|Diptera|Cecidomyiidae||BOLD:ACO0286
Cecidomyiidae|[7456]|GMARN1383-14|Diptera|Cecidomyiidae||BOLD:ACN9834
Cecidomyiidae|[7457]|GMARN950-14|Diptera|Cecidomyiidae||BOLD:ACO0015
Cecidomyiidae|[7458]|GMART765-14|Diptera|Cecidomyiidae||BOLD:ACN9732
Cecidomyiidae|[7459]|GMART1134-14|Diptera|Cecidomyiidae||BOLD:ACO0607
Cecidomyiidae|[7460]|GMAGS1299-15|Diptera|Cecidomyiidae||BOLD:ACX5480
Cecidomyiidae|[7461]|GMARM272-14|Diptera|Cecidomyiidae||BOLD:ACN2864
Cecidomyiidae|[7462]|GMAGE1778-15|Diptera|Cecidomyiidae||BOLD:ACN0078
Cecidomyiidae|[7463]|GMARR185-14|Diptera|Cecidomyiidae||BOLD:ACN7761
Cecidomyiidae|[7464]|GMARF920-14|Diptera|Cecidomyiidae||BOLD:ACM9366
Cecidomyiidae|[7465]|GMART1426-14|Diptera|Cecidomyiidae||BOLD:ACO0133
Cecidomyiidae|[7466]|GMAGK1479-15|Diptera|Cecidomyiidae||BOLD:ACW9217
Cecidomyiidae|[7467]|GMAGS365-15|Diptera|Cecidomyiidae||BOLD:ACX2544

Cecidomyiidae|[7465]|GMART1426-14|Diptera|Cecidomyiidae||BOLD:ACO0133
Cecidomyiidae|[7466]|GMAGK1479-15|Diptera|Cecidomyiidae||BOLD:ACW9217
Cecidomyiidae|[7467]|GMAGS365-15|Diptera|Cecidomyiidae||BOLD:ACX2544
Cecidomyiidae|[7468]|GMAGL2707-15|Diptera|Cecidomyiidae||BOLD:ACO1146
Cecidomyiidae|[7469]|GMAGS537-15|Diptera|Cecidomyiidae||BOLD:ACX4432
Cecidomyiidae|[7470]|GMAGX480-15|Diptera|Cecidomyiidae||BOLD:ACX5907
Cecidomyiidae|[7471]|GMARA1532-14|Diptera|Cecidomyiidae||BOLD:ACM4971
Cecidomyiidae|[7472]|GMAGD2099-15|Diptera|Cecidomyiidae||BOLD:ACN0899
Cecidomyiidae|[7473]|GMAGD651-15|Diptera|Cecidomyiidae||BOLD:ACN1998
Cecidomyiidae|[7474]|GMARJ1778-14|Diptera|Cecidomyiidae||BOLD:ACN1511
Cecidomyiidae|[7475]|GMAGR114-15|Diptera|Cecidomyiidae||BOLD:ACX7676
Cecidomyiidae|[7476]|GMAGE2184-15|Diptera|Cecidomyiidae||BOLD:ACW6388
Cecidomyiidae|[7477]|GMARA259-14|Diptera|Cecidomyiidae||BOLD:ACM9511
Cecidomyiidae|[7478]|GMARR259-14|Diptera|Cecidomyiidae||BOLD:ACO0908
Cecidomyiidae|[7479]|GMAGE2022-15|Diptera|Cecidomyiidae||BOLD:ACW7576
Cecidomyiidae|[7480]|GMARI1158-14|Diptera|Cecidomyiidae||BOLD:ACN1870
Cecidomyiidae|[7481]|GMAGD937-15|Diptera|Cecidomyiidae||BOLD:ACW3603
Cecidomyiidae|[7482]|GMARL1125-14|Diptera|Cecidomyiidae||BOLD:ACN2827
Cecidomyiidae|[7483]|GMARC305-14|Diptera|Cecidomyiidae||BOLD:ACM7632
Cecidomyiidae|[7484]|GMAGU870-15|Diptera|Cecidomyiidae||BOLD:ACX5361
Cecidomyiidae|[7485]|GMAGG1884-15|Diptera|Cecidomyiidae||BOLD:ACO0878
Cecidomyiidae|[7486]|GMAGU793-15|Diptera|Cecidomyiidae||BOLD:ACX4574
Cecidomyiidae|[7487]|GMAGD899-15|Diptera|Cecidomyiidae||BOLD:ACN8875
Cecidomyiidae|[7488]|GMAGD1112-15|Diptera|Cecidomyiidae||BOLD:ACW4680
Cecidomyiidae|[7489]|GMARD076-14|Diptera|Cecidomyiidae||BOLD:ACN1153
Cecidomyiidae|[7490]|GMAGD2131-15|Diptera|Cecidomyiidae||BOLD:ACW1904
Cecidomyiidae|[7491]|GMAGE2091-15|Diptera|Cecidomyiidae||BOLD:ACN7560
Cecidomyiidae|[7492]|GMAGQ1871-15|Diptera|Cecidomyiidae||BOLD:ACN9323
Cecidomyiidae|[7493]|GMAGD1122-15|Diptera|Cecidomyiidae||BOLD:ACN8946
Cecidomyiidae|[7494]|GMAGD2042-15|Diptera|Cecidomyiidae||BOLD:ACW2706
Cecidomyiidae|[7495]|GMAGD1066-15|Diptera|Cecidomyiidae||BOLD:ACN8452
Cecidomyiidae|[7496]|GMARS468-14|Diptera|Cecidomyiidae||BOLD:ACN7704
Cecidomyiidae|[7497]|GMARA1262-14|Diptera|Cecidomyiidae||BOLD:ACM9604
Cecidomyiidae|[7498]|GMARR192-14|Diptera|Cecidomyiidae||BOLD:ACN8939
Cecidomyiidae|[7499]|GMAGA469-15|Diptera|Cecidomyiidae||BOLD:ACM9288
Cecidomyiidae|[7500]|GMAGU469-15|Diptera|Cecidomyiidae||BOLD:ACX3073
Cecidomyiidae|[7501]|GMARV061-14|Diptera|Cecidomyiidae||BOLD:ACO0723
Cecidomyiidae|[7502]|GMAGA458-15|Diptera|Cecidomyiidae||BOLD:ACV2562
Cecidomyiidae|[7503]|GMARC650-14|Diptera|Cecidomyiidae||BOLD:ACM6444
Cecidomyiidae|[7504]|GMAGB726-15|Diptera|Cecidomyiidae||BOLD:ACW3123
Cecidomyiidae|[7505]|GMARF603-14|Diptera|Cecidomyiidae||BOLD:ACM9044
Cecidomyiidae|[7506]|GMARA1160-14|Diptera|Cecidomyiidae||BOLD:ACM5347
Cecidomyiidae|[7507]|GMARF551-14|Diptera|Cecidomyiidae||BOLD:ACM9830
Cecidomyiidae|[7508]|GMAGN2150-15|Diptera|Cecidomyiidae||BOLD:ACX2183
Cecidomyiidae|[7509]|GMARO900-14|Diptera|Cecidomyiidae||BOLD:ACN8661
Cecidomyiidae|[7510]|GMARB661-14|Diptera|Cecidomyiidae||BOLD:ACM6368
Cecidomyiidae|[7511]|GMARB741-14|Diptera|Cecidomyiidae||BOLD:ACM5747
Cecidomyiidae|[7512]|GMAGD961-15|Diptera|Cecidomyiidae||BOLD:ACN0971
Cecidomyiidae|[7513]|GMAGG346-15|Diptera|Cecidomyiidae||BOLD:ACW7748
Cecidomyiidae|[7514]|GMAGD2020-15|Diptera|Cecidomyiidae||BOLD:ACM5530
Cecidomyiidae|[7515]|GMARA1172-14|Diptera|Cecidomyiidae||BOLD:ACM5428
Cecidomyiidae|[7516]|GMAGD2206-15|Diptera|Cecidomyiidae||BOLD:ACM9988
Cecidomyiidae|[7517]|GMAGE2651-15|Diptera|Cecidomyiidae||BOLD:ACO0875
Cecidomyiidae|[7518]|GMAGZ161-15|Diptera|Cecidomyiidae||BOLD:ACX3795
Cecidomyiidae|[7519]|GMAGA492-15|Diptera|Cecidomyiidae||BOLD:ACM4992
Cecidomyiidae|[7520]|GMARB832-14|Diptera|Cecidomyiidae||BOLD:ACM5357
Cecidomyiidae|[7521]|GMARB1071-14|Diptera|Cecidomyiidae||BOLD:ACM5695
Cecidomyiidae|[7522]|GMARD2151-14|Diptera|Cecidomyiidae||BOLD:ACN0888
Cecidomyiidae|[7523]|GMARB810-14|Diptera|Cecidomyiidae||BOLD:ACM5506
Cecidomyiidae|[7524]|GMARB623-14|Diptera|Cecidomyiidae||BOLD:ACM6157
Cecidomyiidae|[7525]|GMAGE2520-15|Diptera|Cecidomyiidae||BOLD:ACW6954
Cecidomyiidae|[7526]|GMAGC279-15|Diptera|Cecidomyiidae||BOLD:ACW1566
Cecidomyiidae|[7527]|GMARB1482-14|Diptera|Cecidomyiidae||BOLD:ACM5052
Cecidomyiidae|[7528]|GMAGA477-15|Diptera|Cecidomyiidae||BOLD:ACM4714
Cecidomyiidae|[7529]|GMARA1278-14|Diptera|Cecidomyiidae||BOLD:ACM9836
Cecidomyiidae|[7530]|GMARB1109-14|Diptera|Cecidomyiidae||BOLD:ACM6080
Cecidomyiidae|[7531]|GMAGB713-15|Diptera|Cecidomyiidae||BOLD:ACW3714
Cecidomyiidae|[7532]|GMARC260-14|Diptera|Cecidomyiidae||BOLD:ACM7240
Cecidomyiidae|[7533]|GMARB1288-14|Diptera|Cecidomyiidae||BOLD:ACM5816
Cecidomyiidae|[7534]|GMARA1360-14|Diptera|Cecidomyiidae||BOLD:ACM5763
Cecidomyiidae|[7535]|GMARA1487-14|Diptera|Cecidomyiidae||BOLD:ACM4847
Cecidomyiidae|[7536]|GMAGE2523-15|Diptera|Cecidomyiidae||BOLD:ACW5856
Cecidomyiidae|[7537]|GMAGD1728-15|Diptera|Cecidomyiidae||BOLD:ACN0043
Cecidomyiidae|[7538]|GMAGA478-15|Diptera|Cecidomyiidae||BOLD:ACM6078
Cecidomyiidae|[7539]|GMAGS698-15|Diptera|Cecidomyiidae||BOLD:ACX4323
Cecidomyiidae|[7540]|GMAGD1080-15|Diptera|Cecidomyiidae||BOLD:ACW4660
Cecidomyiidae|[7541]|GMAGD2185-15|Diptera|Cecidomyiidae||BOLD:ACM9616
Asteromyia|[7542]|GMARA356-14|Diptera|Cecidomyiidae|Cecidomyiinae|BOLD:ACX2062
Cecidomyiidae|[7543]|GMARB618-14|Diptera|Cecidomyiidae||BOLD:ACM6161
Cecidomyiidae|[7544]|GMARE612-14|Diptera|Cecidomyiidae||BOLD:ACM8004
Cecidomyiidae|[7545]|GMARP737-14|Diptera|Cecidomyiidae||BOLD:ACO3105
Cecidomyiidae|[7546]|GMARB968-14|Diptera|Cecidomyiidae||BOLD:ACM5083
Cecidomyiidae|[7547]|GMARL418-14|Diptera|Cecidomyiidae||BOLD:ACN6625
Cecidomyiidae|[7548]|GMART421-14|Diptera|Cecidomyiidae||BOLD:ACO0154
Cecidomyiidae|[7549]|GMAGA466-15|Diptera|Cecidomyiidae||BOLD:ACV2416
Cecidomyiidae|[7550]|GMAGB1047-15|Diptera|Cecidomyiidae||BOLD:ACN7618
Cecidomyiidae|[7551]|GMAGQ1494-15|Diptera|Cecidomyiidae||BOLD:ACX5572
Cecidomyiidae|[7552]|GMARB1231-14|Diptera|Cecidomyiidae||BOLD:ACM5435
Cecidomyiidae|[7553]|GMAGD2258-15|Diptera|Cecidomyiidae||BOLD:ACO0268
Cecidomyiidae|[7554]|GMAGW161-15|Diptera|Cecidomyiidae||BOLD:ACX2210
Cecidomyiidae|[7555]|GMAGD1146-15|Diptera|Cecidomyiidae||BOLD:ACN8648
Cecidomyiidae|[7556]|GMAGA485-15|Diptera|Cecidomyiidae||BOLD:ACM6541
Cecidomyiidae|[7557]|GMARA294-14|Diptera|Cecidomyiidae||BOLD:ACM6216
Cecidomyiidae|[7558]|GMAGC461-15|Diptera|Cecidomyiidae||BOLD:ACW1704
Cecidomyiidae|[7559]|GMAGU611-15|Diptera|Cecidomyiidae||BOLD:ACO1060
Cecidomyiidae|[7560]|GMAGB666-15|Diptera|Cecidomyiidae||BOLD:ACW3551
Cecidomyiidae|[7561]|GMARR332-14|Diptera|Cecidomyiidae||BOLD:ACN8838
Cecidomyiidae|[7562]|GMAGA442-15|Diptera|Cecidomyiidae||BOLD:ACM5559
Cecidomyiidae|[7563]|GMART1570-14|Diptera|Cecidomyiidae||BOLD:ACN9213
Cecidomyiidae|[7564]|GMAGD2308-15|Diptera|Cecidomyiidae||BOLD:ACW6797
Cecidomyiidae|[7565]|GMARG790-14|Diptera|Cecidomyiidae||BOLD:ACN1682
Cecidomyiidae|[7566]|GMAGN2188-15|Diptera|Cecidomyiidae||BOLD:ACN2692
Cecidomyiidae|[7567]|GMARB1172-14|Diptera|Cecidomyiidae||BOLD:ACM4717

Cecidomyiidae|[7565]|GMARG790-14|Diptera|Cecidomyiidae||BOLD:ACN1682
Cecidomyiidae|[7566]|GMAGN2188-15|Diptera|Cecidomyiidae||BOLD:ACN2692
Cecidomyiidae|[7567]|GMARB1172-14|Diptera|Cecidomyiidae||BOLD:ACM4717
Cecidomyiidae|[7568]|GMAGB816-15|Diptera|Cecidomyiidae||BOLD:ACW6465
Cecidomyiidae|[7569]|GMAGB1031-15|Diptera|Cecidomyiidae||BOLD:ACW6411
Cecidomyiidae|[7570]|GMARA1193-14|Diptera|Cecidomyiidae||BOLD:ACM9148
Cecidomyiidae|[7571]|GMARB912-14|Diptera|Cecidomyiidae||BOLD:ACM5638
Cecidomyiidae|[7572]|GMAGB1402-15|Diptera|Cecidomyiidae||BOLD:ACX1366
Cecidomyiidae|[7573]|GMAGD1167-15|Diptera|Cecidomyiidae||BOLD:ACW4848
Cecidomyiidae|[7574]|GMAGD999-15|Diptera|Cecidomyiidae||BOLD:ACW4765
Cecidomyiidae|[7575]|GMARA1226-14|Diptera|Cecidomyiidae||BOLD:ACM8410
Cecidomyiidae|[7576]|GMAGB823-15|Diptera|Cecidomyiidae||BOLD:ACW3826
Cecidomyiidae|[7577]|GMARB1665-14|Diptera|Cecidomyiidae||BOLD:ACM6036
Cecidomyiidae|[7578]|GMAGE2310-15|Diptera|Cecidomyiidae||BOLD:ACW6435
Cecidomyiidae|[7579]|GMAGE2456-15|Diptera|Cecidomyiidae||BOLD:ACN9238
Cecidomyiidae|[7580]|GMAGD1280-15|Diptera|Cecidomyiidae||BOLD:ACW3819
Cecidomyiidae|[7581]|GMAGH1168-15|Diptera|Cecidomyiidae||BOLD:ACW4662
Cecidomyiidae|[7582]|GMAGB1379-15|Diptera|Cecidomyiidae||BOLD:ACM5384
Cecidomyiidae|[7583]|GMARA1122-14|Diptera|Cecidomyiidae||BOLD:ACM9664
Cecidomyiidae|[7584]|GMAGD1128-15|Diptera|Cecidomyiidae||BOLD:ACM9527
Cecidomyiidae|[7585]|GMARB1130-14|Diptera|Cecidomyiidae||BOLD:ACM6415
Cecidomyiidae|[7586]|GMAGA494-15|Diptera|Cecidomyiidae||BOLD:ACM9334
Cecidomyiidae|[7587]|GMAGR085-15|Diptera|Cecidomyiidae||BOLD:ACX7145
Cecidomyiidae|[7588]|GMAGS1288-15|Diptera|Cecidomyiidae||BOLD:ACX4277
Cecidomyiidae|[7589]|GMARA1568-14|Diptera|Cecidomyiidae||BOLD:ACM5739
Cecidomyiidae|[7590]|GMARD2165-14|Diptera|Cecidomyiidae||BOLD:ACM5157
Cecidomyiidae|[7591]|GMAGU378-15|Diptera|Cecidomyiidae||BOLD:ACN7924
Cecidomyiidae|[7592]|GMAGD2240-15|Diptera|Cecidomyiidae||BOLD:ACW6286
Cecidomyiidae|[7593]|GMART1610-14|Diptera|Cecidomyiidae||BOLD:ACN9534
Cecidomyiidae|[7594]|GMAGA484-15|Diptera|Cecidomyiidae||BOLD:ACM6373
Cecidomyiidae|[7595]|GMARD1405-14|Diptera|Cecidomyiidae||BOLD:ACM8495
Cecidomyiidae|[7596]|GMARA1386-14|Diptera|Cecidomyiidae||BOLD:ACM4813
Cecidomyiidae|[7597]|GMARV081-14|Diptera|Cecidomyiidae||BOLD:ACO0688
Cecidomyiidae|[7598]|GMAGB1020-15|Diptera|Cecidomyiidae||BOLD:ACW6582
Cecidomyiidae|[7599]|GMAGC102-15|Diptera|Cecidomyiidae||BOLD:ACM6393
Cecidomyiidae|[7600]|GMARA1400-14|Diptera|Cecidomyiidae||BOLD:ACW2878
Cecidomyiidae|[7601]|GMARJ1550-14|Diptera|Cecidomyiidae||BOLD:ACN0037
Cecidomyiidae|[7602]|GMAGA527-15|Diptera|Cecidomyiidae||BOLD:ACM6015
Cecidomyiidae|[7603]|GMARB1018-14|Diptera|Cecidomyiidae||BOLD:ACX1340
Cecidomyiidae|[7604]|GMARM1059-14|Diptera|Cecidomyiidae||BOLD:ACN9263
Cecidomyiidae|[7605]|GMAGQ1577-15|Diptera|Cecidomyiidae||BOLD:ACO0401
Cecidomyiidae|[7606]|GMAGS385-15|Diptera|Cecidomyiidae||BOLD:ACX5801
Cecidomyiidae|[7607]|GMAGB1538-15|Diptera|Cecidomyiidae||BOLD:ACN0267
Cecidomyiidae|[7608]|GMARB554-14|Diptera|Cecidomyiidae||BOLD:ACM5413
Cecidomyiidae|[7609]|GMAGE2293-15|Diptera|Cecidomyiidae||BOLD:ACO1185
Cecidomyiidae|[7610]|GMAGA475-15|Diptera|Cecidomyiidae||BOLD:ACM9009
Cecidomyiidae|[7611]|GMAGD2119-15|Diptera|Cecidomyiidae||BOLD:ACW2626
Cecidomyiidae|[7612]|GMARB1619-14|Diptera|Cecidomyiidae||BOLD:ACM6240
Cecidomyiidae|[7613]|GMARR360-14|Diptera|Cecidomyiidae||BOLD:ACN7391
Cecidomyiidae|[7614]|GMARB1161-14|Diptera|Cecidomyiidae||BOLD:ACN1112
Cecidomyiidae|[7615]|GMARW070-15|Diptera|Cecidomyiidae||BOLD:ACX3850
Cecidomyiidae|[7616]|GMAGC1491-15|Diptera|Cecidomyiidae||BOLD:ACX6891
Cecidomyiidae|[7617]|GMAGC040-15|Diptera|Cecidomyiidae||BOLD:ACN1116
Staphylinidae|[7618]|GMAGC492-15|Coleoptera|Staphylinidae||BOLD:ACW4886
Staphylinidae|[7619]|GMARD2022-14|Coleoptera|Staphylinidae||BOLD:ACQ8840
Cecidomyiidae|[7620]|GMAGB1506-15|Diptera|Cecidomyiidae||BOLD:ACW3630
Cecidomyiidae|[7621]|GMAGC146-15|Diptera|Cecidomyiidae||BOLD:ACV6221
Cecidomyiidae|[7622]|GMARB815-14|Diptera|Cecidomyiidae||BOLD:ACM5507
Cecidomyiidae|[7623]|GMARA1776-14|Diptera|Cecidomyiidae||BOLD:ACM5268
Cecidomyiidae|[7624]|GMAGC236-15|Diptera|Cecidomyiidae||BOLD:ACM6160
Cecidomyiidae|[7625]|GMAGG1786-15|Diptera|Cecidomyiidae||BOLD:ACW5962
Cecidomyiidae|[7626]|GMARE288-14|Diptera|Cecidomyiidae||BOLD:ACN0543
Cecidomyiidae|[7627]|GMARP714-14|Diptera|Cecidomyiidae||BOLD:ACO3782
Cecidomyiidae|[7628]|GMARR648-14|Diptera|Cecidomyiidae||BOLD:ACN8693
Cecidomyiidae|[7629]|GMARA126-14|Diptera|Cecidomyiidae||BOLD:ACM8999
Cecidomyiidae|[7630]|GMAGC191-15|Diptera|Cecidomyiidae||BOLD:ACV7843
Cecidomyiidae|[7631]|GMAGQ1325-15|Diptera|Cecidomyiidae||BOLD:ACX5903
Cecidomyiidae|[7632]|GMAGC1028-15|Diptera|Cecidomyiidae||BOLD:ACM9580
Cecidomyiidae|[7633]|GMARR312-14|Diptera|Cecidomyiidae||BOLD:ACN8861
Cecidomyiidae|[7634]|GMARA1528-14|Diptera|Cecidomyiidae||BOLD:ACM4970
Cecidomyiidae|[7635]|GMARA388-14|Diptera|Cecidomyiidae||BOLD:ACM8425
Cecidomyiidae|[7636]|GMAGC791-15|Diptera|Cecidomyiidae||BOLD:ADB2581
Cecidomyiidae|[7637]|GMARC264-14|Diptera|Cecidomyiidae||BOLD:ACM8319
Cecidomyiidae|[7638]|GMARB679-14|Diptera|Cecidomyiidae||BOLD:ACM4917
Cecidomyiidae|[7639]|GMARD309-14|Diptera|Cecidomyiidae||BOLD:ACN1409
Cecidomyiidae|[7640]|GMAGK1536-15|Diptera|Cecidomyiidae||BOLD:ACW8107
Cecidomyiidae|[7641]|GMAGG1747-15|Diptera|Cecidomyiidae||BOLD:ACW5850
Cecidomyiidae|[7642]|GMARI2074-14|Diptera|Cecidomyiidae||BOLD:ACN0732
Cecidomyiidae|[7643]|GMARM188-14|Diptera|Cecidomyiidae||BOLD:ACN2200
Cecidomyiidae|[7644]|GMART479-14|Diptera|Cecidomyiidae||BOLD:ACN9117
Aphididae|[7645]|GMARC952-14|Hemiptera|Aphididae||BOLD:ACM5189
Eulachnus rileyi|[7646]|GMAGA1062-15|Hemiptera|Aphididae|Lachninae|BOLD:AAC6347
Schizaphis graminum|[7647]|GMAGA1037-15|Hemiptera|Aphididae|Aphidinae|BOLD:ABV9069
Aphididae|[7648]|GMAGG2584-15|Hemiptera|Aphididae||BOLD:ACX1020
Rhopalosiphoninus latysiphon|[7649]|GMARO1258-14|Hemiptera|Aphididae|Aphidinae|BOLD:AAX9061
Aphis nerii|[7650]|GMARS622-14|Hemiptera|Aphididae|Aphidinae|BOLD:AAC1372
Rhopalosiphum maidis|[7651]|GMAGI1109-15|Hemiptera|Aphididae|Aphidinae|BOLD:AAD4538
Rhopalosiphum rufiabdominale|[7652]|GMARE039-14|Hemiptera|Aphididae|Aphidinae|BOLD:AAI0406
Rhopalosiphum nymphaeae|[7653]|GMARK2571-14|Hemiptera|Aphididae|Aphidinae|BOLD:AAE3554
Aphis spiraecola|[7654]|GMAGA1010-15|Hemiptera|Aphididae|Aphidinae|BOLD:AAA4183
Aphis|[7655]|GMARB1765-14|Hemiptera|Aphididae|Aphidinae|BOLD:AAA3070
Aphis coreopsidis|[7656]|GMARK2579-14|Hemiptera|Aphididae|Aphidinae|BOLD:AAJ1228
Aphis|[7657]|GMARS670-14|Hemiptera|Aphididae|Aphidinae|BOLD:AAF7506
Aphidinae|[7658]|GMAGA880-15|Hemiptera|Aphididae|Aphidinae|BOLD:ACY2210
Aphidinae|[7659]|GMAGS1455-15|Hemiptera|Aphididae|Aphidinae|BOLD:AAD7187
Aulacorthum solani|[7660]|GMARM1484-14|Hemiptera|Aphididae|Aphidinae|BOLD:AAB2572
Brachycaudus helichrysi|[7661]|GMARM1428-14|Hemiptera|Aphididae|Aphidinae|BOLD:ACE9092
Myzus persicae|[7662]|GMAGM1762-15|Hemiptera|Aphididae|Aphidinae|BOLD:AAA7683
Uroleucon|[7663]|GMAGP044-15|Hemiptera|Aphididae|Aphidinae|BOLD:AAC1285
Aphididae|[7664]|GMARH019-14|Hemiptera|Aphididae||BOLD:ACN0670
Hymenoptera|[7665]|GMAGB918-15|Hymenoptera|||BOLD:ACW7094
Hymenoptera|[7666]|GMAGN2466-15|Hymenoptera|||BOLD:ACX2029

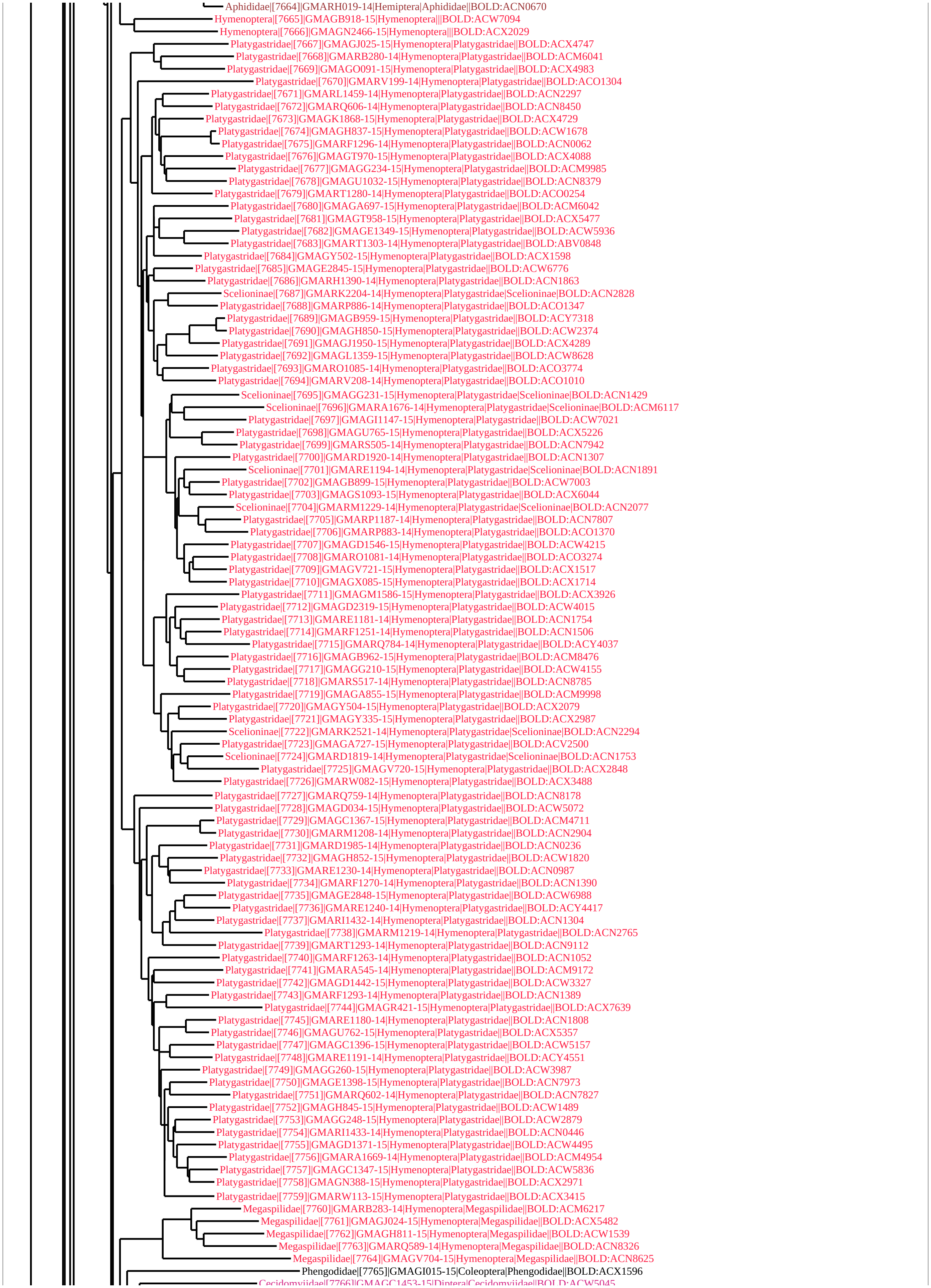
Aphididae|[7664]|GMARH019-14|Hemiptera|Aphididae||BOLD:ACN0670
Hymenoptera|[7665]|GMAGB918-15|Hymenoptera|||BOLD:ACW7094
Hymenoptera|[7666]|GMAGN2466-15|Hymenoptera|||BOLD:ACX2029
Platygastridae|[7667]|GMAGJ025-15|Hymenoptera|Platygastridae||BOLD:ACX4747
Platygastridae|[7668]|GMARB280-14|Hymenoptera|Platygastridae||BOLD:ACM6041
Platygastridae|[7669]|GMAGO091-15|Hymenoptera|Platygastridae||BOLD:ACX4983
Platygastridae|[7670]|GMARV199-14|Hymenoptera|Platygastridae||BOLD:ACO1304
Platygastridae|[7671]|GMARL1459-14|Hymenoptera|Platygastridae||BOLD:ACN2297
Platygastridae|[7672]|GMARQ606-14|Hymenoptera|Platygastridae||BOLD:ACN8450
Platygastridae|[7673]|GMAGK1868-15|Hymenoptera|Platygastridae||BOLD:ACX4729
Platygastridae|[7674]|GMAGH837-15|Hymenoptera|Platygastridae||BOLD:ACW1678
Platygastridae|[7675]|GMARF1296-14|Hymenoptera|Platygastridae||BOLD:ACN0062
Platygastridae|[7676]|GMAGT970-15|Hymenoptera|Platygastridae||BOLD:ACX4088
Platygastridae|[7677]|GMAGG234-15|Hymenoptera|Platygastridae||BOLD:ACM9985
Platygastridae|[7678]|GMAGU1032-15|Hymenoptera|Platygastridae||BOLD:ACN8379
Platygastridae|[7679]|GMART1280-14|Hymenoptera|Platygastridae||BOLD:ACO0254
Platygastridae|[7680]|GMAGA697-15|Hymenoptera|Platygastridae||BOLD:ACM6042
Platygastridae|[7681]|GMAGT958-15|Hymenoptera|Platygastridae||BOLD:ACX5477
Platygastridae|[7682]|GMAGE1349-15|Hymenoptera|Platygastridae||BOLD:ACW5936
Platygastridae|[7683]|GMART1303-14|Hymenoptera|Platygastridae||BOLD:ABV0848
Platygastridae|[7684]|GMAGY502-15|Hymenoptera|Platygastridae||BOLD:ACX1598
Platygastridae|[7685]|GMAGE2845-15|Hymenoptera|Platygastridae||BOLD:ACW6776
Platygastridae|[7686]|GMARH1390-14|Hymenoptera|Platygastridae||BOLD:ACN1863
Scelioninae|[7687]|GMARK2204-14|Hymenoptera|Platygastridae|Scelioninae|BOLD:ACN2828
Platygastridae|[7688]|GMARP886-14|Hymenoptera|Platygastridae||BOLD:ACO1347
Platygastridae|[7689]|GMAGB959-15|Hymenoptera|Platygastridae||BOLD:ACY7318
Platygastridae|[7690]|GMAGH850-15|Hymenoptera|Platygastridae||BOLD:ACW2374
Platygastridae|[7691]|GMAGJ1950-15|Hymenoptera|Platygastridae||BOLD:ACX4289
Platygastridae|[7692]|GMAGL1359-15|Hymenoptera|Platygastridae||BOLD:ACW8628
Platygastridae|[7693]|GMARO1085-14|Hymenoptera|Platygastridae||BOLD:ACO3774
Platygastridae|[7694]|GMARV208-14|Hymenoptera|Platygastridae||BOLD:ACO1010
Scelioninae|[7695]|GMAGG231-15|Hymenoptera|Platygastridae|Scelioninae|BOLD:ACN1429
Scelioninae|[7696]|GMARA1676-14|Hymenoptera|Platygastridae|Scelioninae|BOLD:ACM6117
Platygastridae|[7697]|GMAGI1147-15|Hymenoptera|Platygastridae||BOLD:ACW7021
Platygastridae|[7698]|GMAGU765-15|Hymenoptera|Platygastridae||BOLD:ACX5226
Platygastridae|[7699]|GMARS505-14|Hymenoptera|Platygastridae||BOLD:ACN7942
Platygastridae|[7700]|GMARD1920-14|Hymenoptera|Platygastridae||BOLD:ACN1307
Scelioninae|[7701]|GMARE1194-14|Hymenoptera|Platygastridae|Scelioninae|BOLD:ACN1891
Platygastridae|[7702]|GMAGB899-15|Hymenoptera|Platygastridae||BOLD:ACW7003
Platygastridae|[7703]|GMAGS1093-15|Hymenoptera|Platygastridae||BOLD:ACX6044
Scelioninae|[7704]|GMARM1229-14|Hymenoptera|Platygastridae|Scelioninae|BOLD:ACN2077
Platygastridae|[7705]|GMARP1187-14|Hymenoptera|Platygastridae||BOLD:ACN7807
Platygastridae|[7706]|GMARP883-14|Hymenoptera|Platygastridae||BOLD:ACO1370
Platygastridae|[7707]|GMAGD1546-15|Hymenoptera|Platygastridae||BOLD:ACW4215
Platygastridae|[7708]|GMARO1081-14|Hymenoptera|Platygastridae||BOLD:ACO3274
Platygastridae|[7709]|GMAGV721-15|Hymenoptera|Platygastridae||BOLD:ACX1517
Platygastridae|[7710]|GMAGX085-15|Hymenoptera|Platygastridae||BOLD:ACX1714
Platygastridae|[7711]|GMAGM1586-15|Hymenoptera|Platygastridae||BOLD:ACX3926
Platygastridae|[7712]|GMAGD2319-15|Hymenoptera|Platygastridae||BOLD:ACW4015
Platygastridae|[7713]|GMARE1181-14|Hymenoptera|Platygastridae||BOLD:ACN1754
Platygastridae|[7714]|GMARF1251-14|Hymenoptera|Platygastridae||BOLD:ACN1506
Platygastridae|[7715]|GMARQ784-14|Hymenoptera|Platygastridae||BOLD:ACY4037
Platygastridae|[7716]|GMAGB962-15|Hymenoptera|Platygastridae||BOLD:ACM8476
Platygastridae|[7717]|GMAGG210-15|Hymenoptera|Platygastridae||BOLD:ACW4155
Platygastridae|[7718]|GMARS517-14|Hymenoptera|Platygastridae||BOLD:ACN8785
Platygastridae|[7719]|GMAGA855-15|Hymenoptera|Platygastridae||BOLD:ACM9998
Platygastridae|[7720]|GMAGY504-15|Hymenoptera|Platygastridae||BOLD:ACX2079
Platygastridae|[7721]|GMAGY335-15|Hymenoptera|Platygastridae||BOLD:ACX2987
Scelioninae|[7722]|GMARK2521-14|Hymenoptera|Platygastridae|Scelioninae|BOLD:ACN2294
Platygastridae|[7723]|GMAGA727-15|Hymenoptera|Platygastridae||BOLD:ACV2500
Scelioninae|[7724]|GMARD1819-14|Hymenoptera|Platygastridae|Scelioninae|BOLD:ACN1753
Platygastridae|[7725]|GMAGV720-15|Hymenoptera|Platygastridae||BOLD:ACX2848
Platygastridae|[7726]|GMARW082-15|Hymenoptera|Platygastridae||BOLD:ACX3488
Platygastridae|[7727]|GMARQ759-14|Hymenoptera|Platygastridae||BOLD:ACN8178
Platygastridae|[7728]|GMAGD034-15|Hymenoptera|Platygastridae||BOLD:ACW5072
Platygastridae|[7729]|GMAGC1367-15|Hymenoptera|Platygastridae||BOLD:ACM4711
Platygastridae|[7730]|GMARM1208-14|Hymenoptera|Platygastridae||BOLD:ACN2904
Platygastridae|[7731]|GMARD1985-14|Hymenoptera|Platygastridae||BOLD:ACN0236
Platygastridae|[7732]|GMAGH852-15|Hymenoptera|Platygastridae||BOLD:ACW1820
Platygastridae|[7733]|GMARE1230-14|Hymenoptera|Platygastridae||BOLD:ACN0987
Platygastridae|[7734]|GMARF1270-14|Hymenoptera|Platygastridae||BOLD:ACN1390
Platygastridae|[7735]|GMAGE2848-15|Hymenoptera|Platygastridae||BOLD:ACW6988
Platygastridae|[7736]|GMARE1240-14|Hymenoptera|Platygastridae||BOLD:ACY4417
Platygastridae|[7737]|GMARI1432-14|Hymenoptera|Platygastridae||BOLD:ACN1304
Platygastridae|[7738]|GMARM1219-14|Hymenoptera|Platygastridae||BOLD:ACN2765
Platygastridae|[7739]|GMART1293-14|Hymenoptera|Platygastridae||BOLD:ACN9112
Platygastridae|[7740]|GMARF1263-14|Hymenoptera|Platygastridae||BOLD:ACN1052
Platygastridae|[7741]|GMARA545-14|Hymenoptera|Platygastridae||BOLD:ACM9172
Platygastridae|[7742]|GMAGD1442-15|Hymenoptera|Platygastridae||BOLD:ACW3327
Platygastridae|[7743]|GMARF1293-14|Hymenoptera|Platygastridae||BOLD:ACN1389
Platygastridae|[7744]|GMAGR421-15|Hymenoptera|Platygastridae||BOLD:ACX7639
Platygastridae|[7745]|GMARE1180-14|Hymenoptera|Platygastridae||BOLD:ACN1808
Platygastridae|[7746]|GMAGU762-15|Hymenoptera|Platygastridae||BOLD:ACX5357
Platygastridae|[7747]|GMAGC1396-15|Hymenoptera|Platygastridae||BOLD:ACW5157
Platygastridae|[7748]|GMARE1191-14|Hymenoptera|Platygastridae||BOLD:ACY4551
Platygastridae|[7749]|GMAGG260-15|Hymenoptera|Platygastridae||BOLD:ACW3987
Platygastridae|[7750]|GMAGE1398-15|Hymenoptera|Platygastridae||BOLD:ACN7973
Platygastridae|[7751]|GMARQ602-14|Hymenoptera|Platygastridae||BOLD:ACN7827
Platygastridae|[7752]|GMAGH845-15|Hymenoptera|Platygastridae||BOLD:ACW1489
Platygastridae|[7753]|GMAGG248-15|Hymenoptera|Platygastridae||BOLD:ACW2879
Platygastridae|[7754]|GMARI1433-14|Hymenoptera|Platygastridae||BOLD:ACN0446
Platygastridae|[7755]|GMAGD1371-15|Hymenoptera|Platygastridae||BOLD:ACW4495
Platygastridae|[7756]|GMARA1669-14|Hymenoptera|Platygastridae||BOLD:ACM4954
Platygastridae|[7757]|GMAGC1347-15|Hymenoptera|Platygastridae||BOLD:ACW5836
Platygastridae|[7758]|GMAGN388-15|Hymenoptera|Platygastridae||BOLD:ACX2971
Platygastridae|[7759]|GMARW113-15|Hymenoptera|Platygastridae||BOLD:ACX3415
Megaspilidae|[7760]|GMARB283-14|Hymenoptera|Megaspilidae||BOLD:ACM6217
Megaspilidae|[7761]|GMAGJ024-15|Hymenoptera|Megaspilidae||BOLD:ACX5482
Megaspilidae|[7762]|GMAGH811-15|Hymenoptera|Megaspilidae||BOLD:ACW1539
Megaspilidae|[7763]|GMARQ589-14|Hymenoptera|Megaspilidae||BOLD:ACN8326
Megaspilidae|[7764]|GMAGV704-15|Hymenoptera|Megaspilidae||BOLD:ACN8625
Phengodidae|[7765]|GMAGI015-15|Coleoptera|Phengodidae||BOLD:ACX1596
Cecidomyiidae|[7766]|GMAGC1453-15|Diptera|Cecidomyiidae||BOLD:ACW5045

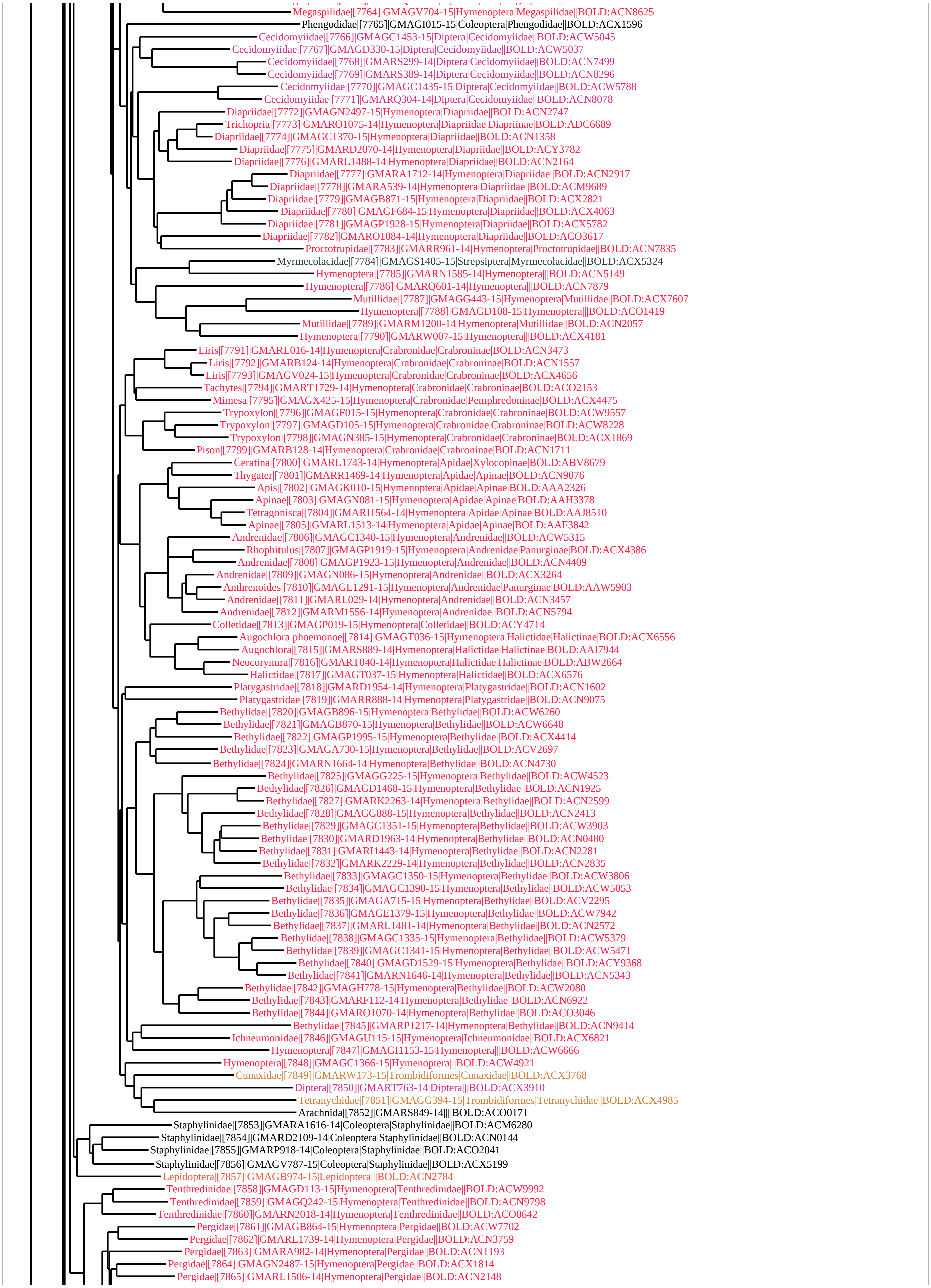
Megaspilidae|[7764]|GMAGV704-15|Hymenoptera|Megaspilidae||BOLD:ACN8625
Phengodidae|[7765]|GMAGI015-15|Coleoptera|Phengodidae||BOLD:ACX1596
Cecidomyiidae|[7766]|GMAGC1453-15|Diptera|Cecidomyiidae||BOLD:ACW5045
Cecidomyiidae|[7767]|GMAGD330-15|Diptera|Cecidomyiidae||BOLD:ACW5037
Cecidomyiidae|[7768]|GMARS299-14|Diptera|Cecidomyiidae||BOLD:ACN7499
Cecidomyiidae|[7769]|GMARS389-14|Diptera|Cecidomyiidae||BOLD:ACN8296
Cecidomyiidae|[7770]|GMAGC1435-15|Diptera|Cecidomyiidae||BOLD:ACW5788
Cecidomyiidae|[7771]|GMARQ304-14|Diptera|Cecidomyiidae||BOLD:ACN8078
Diapriidae|[7772]|GMAGN2497-15|Hymenoptera|Diapriidae||BOLD:ACN2747
Trichopria|[7773]|GMARO1075-14|Hymenoptera|Diapriidae|Diapriinae|BOLD:ADC6689
Diapriidae|[7774]|GMAGC1370-15|Hymenoptera|Diapriidae||BOLD:ACN1358
Diapriidae|[7775]|GMARD2070-14|Hymenoptera|Diapriidae||BOLD:ACY3782
Diapriidae|[7776]|GMARL1488-14|Hymenoptera|Diapriidae||BOLD:ACN2164
Diapriidae|[7777]|GMARA1712-14|Hymenoptera|Diapriidae||BOLD:ACN2917
Diapriidae|[7778]|GMARA539-14|Hymenoptera|Diapriidae||BOLD:ACM9689
Diapriidae|[7779]|GMAGB871-15|Hymenoptera|Diapriidae||BOLD:ACX2821
Diapriidae|[7780]|GMAGF684-15|Hymenoptera|Diapriidae||BOLD:ACX4063
Diapriidae|[7781]|GMAGP1928-15|Hymenoptera|Diapriidae||BOLD:ACX5782
Diapriidae|[7782]|GMARO1084-14|Hymenoptera|Diapriidae||BOLD:ACO3617
Proctotrupidae|[7783]|GMARR961-14|Hymenoptera|Proctotrupidae||BOLD:ACN7835
Myrmecolacidae|[7784]|GMAGS1405-15|Strepsiptera|Myrmecolacidae||BOLD:ACX5324
Hymenoptera|[7785]|GMARN1585-14|Hymenoptera|||BOLD:ACN5149
Hymenoptera|[7786]|GMARQ601-14|Hymenoptera|||BOLD:ACN7879
Mutillidae|[7787]|GMAGG443-15|Hymenoptera|Mutillidae||BOLD:ACX7607
Hymenoptera|[7788]|GMAGD108-15|Hymenoptera|||BOLD:ACO1419
Mutillidae|[7789]|GMARM1200-14|Hymenoptera|Mutillidae||BOLD:ACN2057
Hymenoptera|[7790]|GMARW007-15|Hymenoptera|||BOLD:ACX4181
Liris|[7791]|GMARL016-14|Hymenoptera|Crabronidae|Crabroninae|BOLD:ACN3473
Liris|[7792]|GMARB124-14|Hymenoptera|Crabronidae|Crabroninae|BOLD:ACN1557
Liris|[7793]|GMAGV024-15|Hymenoptera|Crabronidae|Crabroninae|BOLD:ACX4656
Tachytes|[7794]|GMART1729-14|Hymenoptera|Crabronidae|Crabroninae|BOLD:ACO2153
Mimesa|[7795]|GMAGX425-15|Hymenoptera|Crabronidae|Pemphredoninae|BOLD:ACX4475
Trypoxylon|[7796]|GMAGF015-15|Hymenoptera|Crabronidae|Crabroninae|BOLD:ACW9557
Trypoxylon|[7797]|GMAGD105-15|Hymenoptera|Crabronidae|Crabroninae|BOLD:ACW8228
Trypoxylon|[7798]|GMAGN385-15|Hymenoptera|Crabronidae|Crabroninae|BOLD:ACX1869
Pison|[7799]|GMARB128-14|Hymenoptera|Crabronidae|Crabroninae|BOLD:ACN1711
Ceratina|[7800]|GMARL1743-14|Hymenoptera|Apidae|Xylocopinae|BOLD:ABV8679
Thygater|[7801]|GMARR1469-14|Hymenoptera|Apidae|Apinae|BOLD:ACN9076
Apis|[7802]|GMAGK010-15|Hymenoptera|Apidae|Apinae|BOLD:AAA2326
Apinae|[7803]|GMAGN081-15|Hymenoptera|Apidae|Apinae|BOLD:AAH3378
Tetragonisca|[7804]|GMARI1564-14|Hymenoptera|Apidae|Apinae|BOLD:AAJ8510
Apinae|[7805]|GMARL1513-14|Hymenoptera|Apidae|Apinae|BOLD:AAF3842
Andrenidae|[7806]|GMAGC1340-15|Hymenoptera|Andrenidae||BOLD:ACW5315
Rhophitulus|[7807]|GMAGP1919-15|Hymenoptera|Andrenidae|Panurginae|BOLD:ACX4386
Andrenidae|[7808]|GMAGP1923-15|Hymenoptera|Andrenidae||BOLD:ACN4409
Andrenidae|[7809]|GMAGN086-15|Hymenoptera|Andrenidae||BOLD:ACX3264
Anthrenoides|[7810]|GMAGL1291-15|Hymenoptera|Andrenidae|Panurginae|BOLD:AAW5903
Andrenidae|[7811]|GMARL029-14|Hymenoptera|Andrenidae||BOLD:ACN3457
Andrenidae|[7812]|GMARM1556-14|Hymenoptera|Andrenidae||BOLD:ACN5794
Colletidae|[7813]|GMAGP019-15|Hymenoptera|Colletidae||BOLD:ACY4714
Augochlora phoemonoe|[7814]|GMAGT036-15|Hymenoptera|Halictidae|Halictinae|BOLD:ACX6556
Augochlora|[7815]|GMARS889-14|Hymenoptera|Halictidae|Halictinae|BOLD:AAI7944
Neocorynura|[7816]|GMART040-14|Hymenoptera|Halictidae|Halictinae|BOLD:ABW2664
Halictidae|[7817]|GMAGT037-15|Hymenoptera|Halictidae||BOLD:ACX6576
Platygastridae|[7818]|GMARD1954-14|Hymenoptera|Platygastridae||BOLD:ACN1602
Platygastridae|[7819]|GMARR888-14|Hymenoptera|Platygastridae||BOLD:ACN9075
Bethylidae|[7820]|GMAGB896-15|Hymenoptera|Bethylidae||BOLD:ACW6260
Bethylidae|[7821]|GMAGB870-15|Hymenoptera|Bethylidae||BOLD:ACW6648
Bethylidae|[7822]|GMAGP1995-15|Hymenoptera|Bethylidae||BOLD:ACX4414
Bethylidae|[7823]|GMAGA730-15|Hymenoptera|Bethylidae||BOLD:ACV2697
Bethylidae|[7824]|GMARN1664-14|Hymenoptera|Bethylidae||BOLD:ACN4730
Bethylidae|[7825]|GMAGG225-15|Hymenoptera|Bethylidae||BOLD:ACW4523
Bethylidae|[7826]|GMAGD1468-15|Hymenoptera|Bethylidae||BOLD:ACN1925
Bethylidae|[7827]|GMARK2263-14|Hymenoptera|Bethylidae||BOLD:ACN2599
Bethylidae|[7828]|GMAGG888-15|Hymenoptera|Bethylidae||BOLD:ACN2413
Bethylidae|[7829]|GMAGC1351-15|Hymenoptera|Bethylidae||BOLD:ACW3903
Bethylidae|[7830]|GMARD1963-14|Hymenoptera|Bethylidae||BOLD:ACN0480
Bethylidae|[7831]|GMARI1443-14|Hymenoptera|Bethylidae||BOLD:ACN2281
Bethylidae|[7832]|GMARK2229-14|Hymenoptera|Bethylidae||BOLD:ACN2835
Bethylidae|[7833]|GMAGC1350-15|Hymenoptera|Bethylidae||BOLD:ACW3806
Bethylidae|[7834]|GMAGC1390-15|Hymenoptera|Bethylidae||BOLD:ACW5053
Bethylidae|[7835]|GMAGA715-15|Hymenoptera|Bethylidae||BOLD:ACV2295
Bethylidae|[7836]|GMAGE1379-15|Hymenoptera|Bethylidae||BOLD:ACW7942
Bethylidae|[7837]|GMARL1481-14|Hymenoptera|Bethylidae||BOLD:ACN2572
Bethylidae|[7838]|GMAGC1335-15|Hymenoptera|Bethylidae||BOLD:ACW5379
Bethylidae|[7839]|GMAGC1341-15|Hymenoptera|Bethylidae||BOLD:ACW5471
Bethylidae|[7840]|GMAGD1529-15|Hymenoptera|Bethylidae||BOLD:ACY9368
Bethylidae|[7841]|GMARN1646-14|Hymenoptera|Bethylidae||BOLD:ACN5343
Bethylidae|[7842]|GMAGH778-15|Hymenoptera|Bethylidae||BOLD:ACW2080
Bethylidae|[7843]|GMARF112-14|Hymenoptera|Bethylidae||BOLD:ACN6922
Bethylidae|[7844]|GMARO1070-14|Hymenoptera|Bethylidae||BOLD:ACO3046
Bethylidae|[7845]|GMARP1217-14|Hymenoptera|Bethylidae||BOLD:ACN9414
Ichneumonidae|[7846]|GMAGU115-15|Hymenoptera|Ichneumonidae||BOLD:ACX6821
Hymenoptera|[7847]|GMAGI1153-15|Hymenoptera|||BOLD:ACW6666
Hymenoptera|[7848]|GMAGC1366-15|Hymenoptera|||BOLD:ACW4921
Cunaxidae|[7849]|GMARW173-15|Trombidiformes|Cunaxidae||BOLD:ACX3768
Diptera|[7850]|GMART763-14|Diptera|||BOLD:ACX3910
Tetranychidae|[7851]|GMAGG394-15|Trombidiformes|Tetranychidae||BOLD:ACX4985
Arachnida|[7852]|GMARS849-14|||||BOLD:ACO0171
Staphylinidae|[7853]|GMARA1616-14|Coleoptera|Staphylinidae||BOLD:ACM6280
Staphylinidae|[7854]|GMARD2109-14|Coleoptera|Staphylinidae||BOLD:ACN0144
Staphylinidae|[7855]|GMARP918-14|Coleoptera|Staphylinidae||BOLD:ACO2041
Staphylinidae|[7856]|GMAGV787-15|Coleoptera|Staphylinidae||BOLD:ACX5199
Lepidoptera|[7857]|GMAGB974-15|Lepidoptera|||BOLD:ACN2784
Tenthredinidae|[7858]|GMAGD113-15|Hymenoptera|Tenthredinidae||BOLD:ACW9992
Tenthredinidae|[7859]|GMAGQ242-15|Hymenoptera|Tenthredinidae||BOLD:ACN9798
Tenthredinidae|[7860]|GMARN2018-14|Hymenoptera|Tenthredinidae||BOLD:ACO0642
Pergidae|[7861]|GMAGB864-15|Hymenoptera|Pergidae||BOLD:ACW7702
Pergidae|[7862]|GMARL1739-14|Hymenoptera|Pergidae||BOLD:ACN3759
Pergidae|[7863]|GMARA982-14|Hymenoptera|Pergidae||BOLD:ACN1193
Pergidae|[7864]|GMAGN2487-15|Hymenoptera|Pergidae||BOLD:ACX1814
Pergidae|[7865]|GMARL1506-14|Hymenoptera|Pergidae||BOLD:ACN2148

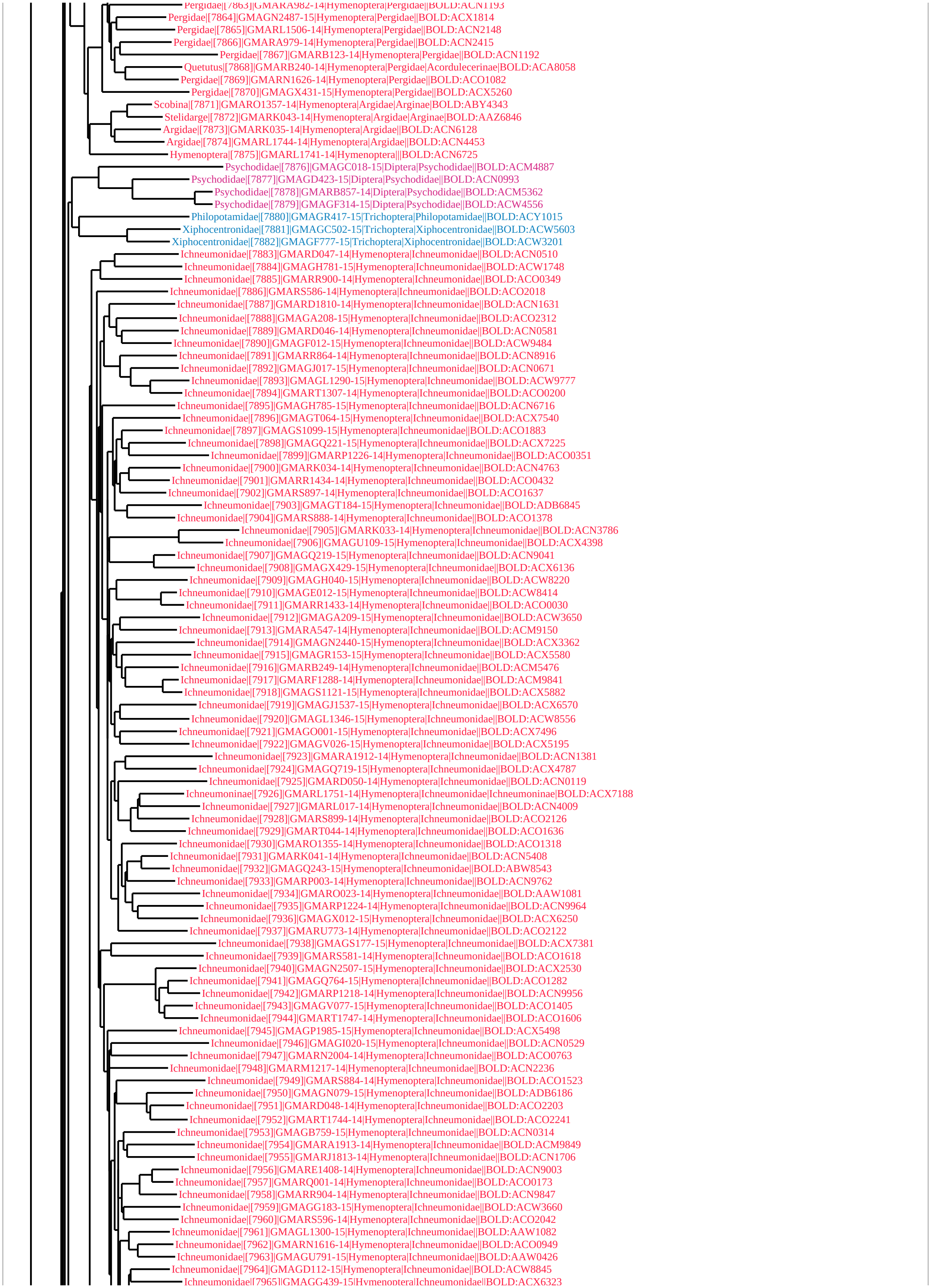
Pergidae|[7863]|GMARA982-14|Hymenoptera|Pergidae||BOLD:ACN1193
Pergidae|[7864]|GMAGN2487-15|Hymenoptera|Pergidae||BOLD:ACX1814
Pergidae|[7865]|GMARL1506-14|Hymenoptera|Pergidae||BOLD:ACN2148
Pergidae|[7866]|GMARA979-14|Hymenoptera|Pergidae||BOLD:ACN2415
Pergidae|[7867]|GMARB123-14|Hymenoptera|Pergidae||BOLD:ACN1192
Quetutus|[7868]|GMARB240-14|Hymenoptera|Pergidae|Acordulecerinae|BOLD:ACA8058
Pergidae|[7869]|GMARN1626-14|Hymenoptera|Pergidae||BOLD:ACO1082
Pergidae|[7870]|GMAGX431-15|Hymenoptera|Pergidae||BOLD:ACX5260
Scobina|[7871]|GMARO1357-14|Hymenoptera|Argidae|Arginae|BOLD:ABY4343
Stelidarge|[7872]|GMARK043-14|Hymenoptera|Argidae|Arginae|BOLD:AAZ6846
Argidae|[7873]|GMARK035-14|Hymenoptera|Argidae||BOLD:ACN6128
Argidae|[7874]|GMARL1744-14|Hymenoptera|Argidae||BOLD:ACN4453
Hymenoptera|[7875]|GMARL1741-14|Hymenoptera|||BOLD:ACN6725
Psychodidae|[7876]|GMAGC018-15|Diptera|Psychodidae||BOLD:ACM4887
Psychodidae|[7877]|GMAGD423-15|Diptera|Psychodidae||BOLD:ACN0993
Psychodidae|[7878]|GMARB857-14|Diptera|Psychodidae||BOLD:ACM5362
Psychodidae|[7879]|GMAGF314-15|Diptera|Psychodidae||BOLD:ACW4556
Philopotamidae|[7880]|GMAGR417-15|Trichoptera|Philopotamidae||BOLD:ACY1015
Xiphocentronidae|[7881]|GMAGC502-15|Trichoptera|Xiphocentronidae||BOLD:ACW5603
Xiphocentronidae|[7882]|GMAGF777-15|Trichoptera|Xiphocentronidae||BOLD:ACW3201
Ichneumonidae|[7883]|GMARD047-14|Hymenoptera|Ichneumonidae||BOLD:ACN0510
Ichneumonidae|[7884]|GMAGH781-15|Hymenoptera|Ichneumonidae||BOLD:ACW1748
Ichneumonidae|[7885]|GMARR900-14|Hymenoptera|Ichneumonidae||BOLD:ACO0349
Ichneumonidae|[7886]|GMARS586-14|Hymenoptera|Ichneumonidae||BOLD:ACO2018
Ichneumonidae|[7887]|GMARD1810-14|Hymenoptera|Ichneumonidae||BOLD:ACN1631
Ichneumonidae|[7888]|GMAGA208-15|Hymenoptera|Ichneumonidae||BOLD:ACO2312
Ichneumonidae|[7889]|GMARD046-14|Hymenoptera|Ichneumonidae||BOLD:ACN0581
Ichneumonidae|[7890]|GMAGF012-15|Hymenoptera|Ichneumonidae||BOLD:ACW9484
Ichneumonidae|[7891]|GMARR864-14|Hymenoptera|Ichneumonidae||BOLD:ACN8916
Ichneumonidae|[7892]|GMAGJ017-15|Hymenoptera|Ichneumonidae||BOLD:ACN0671
Ichneumonidae|[7893]|GMAGL1290-15|Hymenoptera|Ichneumonidae||BOLD:ACW9777
Ichneumonidae|[7894]|GMART1307-14|Hymenoptera|Ichneumonidae||BOLD:ACO0200
Ichneumonidae|[7895]|GMAGH785-15|Hymenoptera|Ichneumonidae||BOLD:ACN6716
Ichneumonidae|[7896]|GMAGT064-15|Hymenoptera|Ichneumonidae||BOLD:ACX7540
Ichneumonidae|[7897]|GMAGS1099-15|Hymenoptera|Ichneumonidae||BOLD:ACO1883
Ichneumonidae|[7898]|GMAGQ221-15|Hymenoptera|Ichneumonidae||BOLD:ACX7225
Ichneumonidae|[7899]|GMARP1226-14|Hymenoptera|Ichneumonidae||BOLD:ACO0351
Ichneumonidae|[7900]|GMARK034-14|Hymenoptera|Ichneumonidae||BOLD:ACN4763
Ichneumonidae|[7901]|GMARR1434-14|Hymenoptera|Ichneumonidae||BOLD:ACO0432
Ichneumonidae|[7902]|GMARS897-14|Hymenoptera|Ichneumonidae||BOLD:ACO1637
Ichneumonidae|[7903]|GMAGT184-15|Hymenoptera|Ichneumonidae||BOLD:ADB6845
Ichneumonidae|[7904]|GMARS888-14|Hymenoptera|Ichneumonidae||BOLD:ACO1378
Ichneumonidae|[7905]|GMARK033-14|Hymenoptera|Ichneumonidae||BOLD:ACN3786
Ichneumonidae|[7906]|GMAGU109-15|Hymenoptera|Ichneumonidae||BOLD:ACX4398
Ichneumonidae|[7907]|GMAGQ219-15|Hymenoptera|Ichneumonidae||BOLD:ACN9041
Ichneumonidae|[7908]|GMAGX429-15|Hymenoptera|Ichneumonidae||BOLD:ACX6136
Ichneumonidae|[7909]|GMAGH040-15|Hymenoptera|Ichneumonidae||BOLD:ACW8220
Ichneumonidae|[7910]|GMAGE012-15|Hymenoptera|Ichneumonidae||BOLD:ACW8414
Ichneumonidae|[7911]|GMARR1433-14|Hymenoptera|Ichneumonidae||BOLD:ACO0030
Ichneumonidae|[7912]|GMAGA209-15|Hymenoptera|Ichneumonidae||BOLD:ACW3650
Ichneumonidae|[7913]|GMARA547-14|Hymenoptera|Ichneumonidae||BOLD:ACM9150
Ichneumonidae|[7914]|GMAGN2440-15|Hymenoptera|Ichneumonidae||BOLD:ACX3362
Ichneumonidae|[7915]|GMAGR153-15|Hymenoptera|Ichneumonidae||BOLD:ACX5580
Ichneumonidae|[7916]|GMARB249-14|Hymenoptera|Ichneumonidae||BOLD:ACM5476
Ichneumonidae|[7917]|GMARF1288-14|Hymenoptera|Ichneumonidae||BOLD:ACM9841
Ichneumonidae|[7918]|GMAGS1121-15|Hymenoptera|Ichneumonidae||BOLD:ACX5882
Ichneumonidae|[7919]|GMAGJ1537-15|Hymenoptera|Ichneumonidae||BOLD:ACX6570
Ichneumonidae|[7920]|GMAGL1346-15|Hymenoptera|Ichneumonidae||BOLD:ACW8556
Ichneumonidae|[7921]|GMAGO001-15|Hymenoptera|Ichneumonidae||BOLD:ACX7496
Ichneumonidae|[7922]|GMAGV026-15|Hymenoptera|Ichneumonidae||BOLD:ACX5195
Ichneumonidae|[7923]|GMARA1912-14|Hymenoptera|Ichneumonidae||BOLD:ACN1381
Ichneumonidae|[7924]|GMAGQ719-15|Hymenoptera|Ichneumonidae||BOLD:ACX4787
Ichneumonidae|[7925]|GMARD050-14|Hymenoptera|Ichneumonidae||BOLD:ACN0119
Ichneumoninae|[7926]|GMARL1751-14|Hymenoptera|Ichneumonidae|Ichneumoninae|BOLD:ACX7188
Ichneumonidae|[7927]|GMARL017-14|Hymenoptera|Ichneumonidae||BOLD:ACN4009
Ichneumonidae|[7928]|GMARS899-14|Hymenoptera|Ichneumonidae||BOLD:ACO2126
Ichneumonidae|[7929]|GMART044-14|Hymenoptera|Ichneumonidae||BOLD:ACO1636
Ichneumonidae|[7930]|GMARO1355-14|Hymenoptera|Ichneumonidae||BOLD:ACO1318
Ichneumonidae|[7931]|GMARK041-14|Hymenoptera|Ichneumonidae||BOLD:ACN5408
Ichneumonidae|[7932]|GMAGQ243-15|Hymenoptera|Ichneumonidae||BOLD:ABW8543
Ichneumonidae|[7933]|GMARP003-14|Hymenoptera|Ichneumonidae||BOLD:ACN9762
Ichneumonidae|[7934]|GMARO023-14|Hymenoptera|Ichneumonidae||BOLD:AAW1081
Ichneumonidae|[7935]|GMARP1224-14|Hymenoptera|Ichneumonidae||BOLD:ACN9964
Ichneumonidae|[7936]|GMAGX012-15|Hymenoptera|Ichneumonidae||BOLD:ACX6250
Ichneumonidae|[7937]|GMARU773-14|Hymenoptera|Ichneumonidae||BOLD:ACO2122
Ichneumonidae|[7938]|GMAGS177-15|Hymenoptera|Ichneumonidae||BOLD:ACX7381
Ichneumonidae|[7939]|GMARS581-14|Hymenoptera|Ichneumonidae||BOLD:ACO1618
Ichneumonidae|[7940]|GMAGN2507-15|Hymenoptera|Ichneumonidae||BOLD:ACX2530
Ichneumonidae|[7941]|GMAGQ764-15|Hymenoptera|Ichneumonidae||BOLD:ACO1282
Ichneumonidae|[7942]|GMARP1218-14|Hymenoptera|Ichneumonidae||BOLD:ACN9956
Ichneumonidae|[7943]|GMAGV077-15|Hymenoptera|Ichneumonidae||BOLD:ACO1405
Ichneumonidae|[7944]|GMART1747-14|Hymenoptera|Ichneumonidae||BOLD:ACO1606
Ichneumonidae|[7945]|GMAGP1985-15|Hymenoptera|Ichneumonidae||BOLD:ACX5498
Ichneumonidae|[7946]|GMAGI020-15|Hymenoptera|Ichneumonidae||BOLD:ACN0529
Ichneumonidae|[7947]|GMARN2004-14|Hymenoptera|Ichneumonidae||BOLD:ACO0763
Ichneumonidae|[7948]|GMARM1217-14|Hymenoptera|Ichneumonidae||BOLD:ACN2236
Ichneumonidae|[7949]|GMARS884-14|Hymenoptera|Ichneumonidae||BOLD:ACO1523
Ichneumonidae|[7950]|GMAGN079-15|Hymenoptera|Ichneumonidae||BOLD:ADB6186
Ichneumonidae|[7951]|GMARD048-14|Hymenoptera|Ichneumonidae||BOLD:ACO2203
Ichneumonidae|[7952]|GMART1744-14|Hymenoptera|Ichneumonidae||BOLD:ACO2241
Ichneumonidae|[7953]|GMAGB759-15|Hymenoptera|Ichneumonidae||BOLD:ACN0314
Ichneumonidae|[7954]|GMARA1913-14|Hymenoptera|Ichneumonidae||BOLD:ACM9849
Ichneumonidae|[7955]|GMARJ1813-14|Hymenoptera|Ichneumonidae||BOLD:ACN1706
Ichneumonidae|[7956]|GMARE1408-14|Hymenoptera|Ichneumonidae||BOLD:ACN9003
Ichneumonidae|[7957]|GMARQ001-14|Hymenoptera|Ichneumonidae||BOLD:ACO0173
Ichneumonidae|[7958]|GMARR904-14|Hymenoptera|Ichneumonidae||BOLD:ACN9847
Ichneumonidae|[7959]|GMAGG183-15|Hymenoptera|Ichneumonidae||BOLD:ACW3660
Ichneumonidae|[7960]|GMARS596-14|Hymenoptera|Ichneumonidae||BOLD:ACO2042
Ichneumonidae|[7961]|GMAGL1300-15|Hymenoptera|Ichneumonidae||BOLD:AAW1082
Ichneumonidae|[7962]|GMARN1616-14|Hymenoptera|Ichneumonidae||BOLD:ACO0949
Ichneumonidae|[7963]|GMAGU791-15|Hymenoptera|Ichneumonidae||BOLD:AAW0426
Ichneumonidae|[7964]|GMAGD112-15|Hymenoptera|Ichneumonidae||BOLD:ACW8845
Ichneumonidae|[7965]|GMAGG439-15|Hymenoptera|Ichneumonidae||BOLD:ACX6323

Ichneumonidae|[7963]|GMAGU791-15|Hymenoptera|Ichneumonidae||BOLD:AAW0426
Ichneumonidae|[7964]|GMAGD112-15|Hymenoptera|Ichneumonidae||BOLD:ACW8845
Ichneumonidae|[7965]|GMAGG439-15|Hymenoptera|Ichneumonidae||BOLD:ACX6323
Ichneumonidae|[7966]|GMAGD1463-15|Hymenoptera|Ichneumonidae||BOLD:ACM6485
Ichneumonidae|[7967]|GMARJ046-14|Hymenoptera|Ichneumonidae||BOLD:ACM9894
Ichneumonidae|[7968]|GMAGT974-15|Hymenoptera|Ichneumonidae||BOLD:ACX5454
Ichneumonidae|[7969]|GMAGP1939-15|Hymenoptera|Ichneumonidae||BOLD:ACX5912
Ichneumonidae|[7970]|GMARQ007-14|Hymenoptera|Ichneumonidae||BOLD:ACN9449
Ichneumonidae|[7971]|GMAGP005-15|Hymenoptera|Ichneumonidae||BOLD:ACX6521
Ichneumonidae|[7972]|GMARU767-14|Hymenoptera|Ichneumonidae||BOLD:AAV4421
Ichneumonidae|[7973]|GMAGE024-15|Hymenoptera|Ichneumonidae||BOLD:ACO0577
Ichneumonidae|[7974]|GMARU028-14|Hymenoptera|Ichneumonidae||BOLD:ACO1402
Ichneumonidae|[7975]|GMARD1893-14|Hymenoptera|Ichneumonidae||BOLD:ACN0961
Ichneumonidae|[7976]|GMAGR162-15|Hymenoptera|Ichneumonidae||BOLD:ACN7396
Mesochorus|[7977]|GMAGJ1920-15|Hymenoptera|Ichneumonidae|Mesochorinae|BOLD:ACX6194
Mesochorus|[7978]|GMAGN2491-15|Hymenoptera|Ichneumonidae|Mesochorinae|BOLD:ACX2650
Ichneumonidae|[7979]|GMARI1526-14|Hymenoptera|Ichneumonidae||BOLD:ACN1521
Ichneumonidae|[7980]|GMAGI1139-15|Hymenoptera|Ichneumonidae||BOLD:ACW6579
Ichneumonidae|[7981]|GMAGN2476-15|Hymenoptera|Ichneumonidae||BOLD:ACX2446
Mesochorus|[7982]|GMARJ1820-14|Hymenoptera|Ichneumonidae|Mesochorinae|BOLD:ACN0332
Ichneumonidae|[7983]|GMAGP1960-15|Hymenoptera|Ichneumonidae||BOLD:ACX5239
Ichneumonidae|[7984]|GMAGJ041-15|Hymenoptera|Ichneumonidae||BOLD:ACX5814
Ichneumonidae|[7985]|GMAGY341-15|Hymenoptera|Ichneumonidae||BOLD:ACX2089
Leurus davidwahli|[7986]|GMAGK064-15|Hymenoptera|Ichneumonidae|Metopiinae|BOLD:AAJ9262
Leurus marjorietownesae|[7987]|GMAGW144-15|Hymenoptera|Ichneumonidae|Metopiinae|BOLD:AAD6183
Colpotrochia|[7988]|GMARP1220-14|Hymenoptera|Ichneumonidae|Metopiinae|BOLD:ACO0545
Ichneumonidae|[7989]|GMARP005-14|Hymenoptera|Ichneumonidae||BOLD:ACO0614
Ichneumonidae|[7990]|GMAGD1464-15|Hymenoptera|Ichneumonidae||BOLD:ACK2330
Ichneumonidae|[7991]|GMARP891-14|Hymenoptera|Ichneumonidae||BOLD:ACO1715
Trieces|[7992]|GMAGV680-15|Hymenoptera|Ichneumonidae|Metopiinae|BOLD:ACX2045
Ichneumonidae|[7993]|GMAGA694-15|Hymenoptera|Ichneumonidae||BOLD:ACV1973
Ichneumonidae|[7994]|GMAGM1385-15|Hymenoptera|Ichneumonidae||BOLD:ACX3682
Meniscomorpha|[7995]|GMAGF011-15|Hymenoptera|Ichneumonidae|Banchinae|BOLD:ACW9079
Ichneumonidae|[7996]|GMAGO004-15|Hymenoptera|Ichneumonidae||BOLD:ACX7228
Ichneumonidae|[7997]|GMAGA210-15|Hymenoptera|Ichneumonidae||BOLD:ABW8538
Ichneumonidae|[7998]|GMAGL1161-15|Hymenoptera|Ichneumonidae||BOLD:ACX7331
Ichneumonidae|[7999]|GMAGU113-15|Hymenoptera|Ichneumonidae||BOLD:ACX7093
Ichneumonidae|[8000]|GMARJ044-14|Hymenoptera|Ichneumonidae||BOLD:ACN1510
Ichneumonidae|[8001]|GMARE1410-14|Hymenoptera|Ichneumonidae||BOLD:ACN6264
Ichneumonidae|[8002]|GMAGQ240-15|Hymenoptera|Ichneumonidae||BOLD:ACX6875
Ichneumonidae|[8003]|GMARS902-14|Hymenoptera|Ichneumonidae||BOLD:ACO1330
Ichneumonidae|[8004]|GMARS593-14|Hymenoptera|Ichneumonidae||BOLD:ACY5984
Ichneumonidae|[8005]|GMAGF017-15|Hymenoptera|Ichneumonidae||BOLD:ACM9977
Ichneumonidae|[8006]|GMAGU020-15|Hymenoptera|Ichneumonidae||BOLD:ACO1410
Ichneumonidae|[8007]|GMAGT173-15|Hymenoptera|Ichneumonidae||BOLD:ADB7070
Ichneumonidae|[8008]|GMAGX421-15|Hymenoptera|Ichneumonidae||BOLD:ACO1493
Netelia|[8009]|GMARM1557-14|Hymenoptera|Ichneumonidae|Tryphoninae|BOLD:ACN4098
Ichneumonidae|[8010]|GMAGE007-15|Hymenoptera|Ichneumonidae||BOLD:ACO0922
Ichneumonidae|[8011]|GMARN1659-14|Hymenoptera|Ichneumonidae||BOLD:ACN5819
Ichneumonidae|[8012]|GMARB019-14|Hymenoptera|Ichneumonidae||BOLD:ACN0306
Ichneumonidae|[8013]|GMARN1667-14|Hymenoptera|Ichneumonidae||BOLD:ACN4930
Anomaloninae|[8014]|GMAGT066-15|Hymenoptera|Ichneumonidae|Anomaloninae|BOLD:AAV3534
Ichneumonidae|[8015]|GMAGB754-15|Hymenoptera|Ichneumonidae||BOLD:ACN3426
Ichneumonidae|[8016]|GMAGW107-15|Hymenoptera|Ichneumonidae||BOLD:ACX6057
Ichneumonidae|[8017]|GMAGY035-15|Hymenoptera|Ichneumonidae||BOLD:ACX4657
Ichneumonidae|[8018]|GMARA569-14|Hymenoptera|Ichneumonidae||BOLD:ACM9276
Ichneumonidae|[8019]|GMARR868-14|Hymenoptera|Ichneumonidae||BOLD:ACN7535
Campoletis flavicincta|[8020]|GMAGP1942-15|Hymenoptera|Ichneumonidae|Campopleginae|BOLD:AAN7740
Ichneumonidae|[8021]|GMAGJ001-15|Hymenoptera|Ichneumonidae||BOLD:ACN1707
Casinaria|[8022]|GMAGS1116-15|Hymenoptera|Ichneumonidae|Campopleginae|BOLD:ACX5603
Casinaria|[8023]|GMAGF010-15|Hymenoptera|Ichneumonidae|Campopleginae|BOLD:ACW9395
Casinaria|[8024]|GMART1734-14|Hymenoptera|Ichneumonidae|Campopleginae|BOLD:ACO1895
Ichneumonidae|[8025]|GMAGQ245-15|Hymenoptera|Ichneumonidae||BOLD:ACX6498
Ichneumonidae|[8026]|GMARH1374-14|Hymenoptera|Ichneumonidae||BOLD:ACN1527
Campoplex|[8027]|GMARU770-14|Hymenoptera|Ichneumonidae|Campopleginae|BOLD:AAD2343
Campoplex|[8028]|GMARL1408-14|Hymenoptera|Ichneumonidae|Campopleginae|BOLD:ABX5473
Ichneumonidae|[8029]|GMAGP1983-15|Hymenoptera|Ichneumonidae||BOLD:ACX5913
Ichneumonidae|[8030]|GMAGS1102-15|Hymenoptera|Ichneumonidae||BOLD:ACX5414
Ichneumonidae|[8031]|GMARP872-14|Hymenoptera|Ichneumonidae||BOLD:ACO1716
Ichneumonidae|[8032]|GMARP007-14|Hymenoptera|Ichneumonidae||BOLD:ACO0877
Ichneumonidae|[8033]|GMARM1565-14|Hymenoptera|Ichneumonidae||BOLD:ACN7215
Ichneumonidae|[8034]|GMARR1421-14|Hymenoptera|Ichneumonidae||BOLD:ACN9727
Ichneumonidae|[8035]|GMAGV022-15|Hymenoptera|Ichneumonidae||BOLD:ACX6234
Campopleginae|[8036]|GMAGG436-15|Hymenoptera|Ichneumonidae|Campopleginae|BOLD:ACN9200
Dusona|[8037]|GMAGA696-15|Hymenoptera|Ichneumonidae|Campopleginae|BOLD:ACM5451
Dusona|[8038]|GMAGE014-15|Hymenoptera|Ichneumonidae|Campopleginae|BOLD:ACN9763
Dusona|[8039]|GMARS585-14|Hymenoptera|Ichneumonidae|Campopleginae|BOLD:ACO1927
Dusona|[8040]|GMARU769-14|Hymenoptera|Ichneumonidae|Campopleginae|BOLD:ACO1520
Ichneumonidae|[8041]|GMARS577-14|Hymenoptera|Ichneumonidae||BOLD:ACO1581
Ichneumonidae|[8042]|GMARS582-14|Hymenoptera|Ichneumonidae||BOLD:ACO2208
Ichneumonidae|[8043]|GMARP010-14|Hymenoptera|Ichneumonidae||BOLD:ACN9954
Ichneumonidae|[8044]|GMART1736-14|Hymenoptera|Ichneumonidae||BOLD:ACO1986
Hyposoter|[8045]|GMAGD1470-15|Hymenoptera|Ichneumonidae|Campopleginae|BOLD:ACW4128
Ichneumonidae|[8046]|GMARN1620-14|Hymenoptera|Ichneumonidae||BOLD:ACO1046
Microcharops|[8047]|GMARK2334-14|Hymenoptera|Ichneumonidae|Campopleginae|BOLD:ACN5334
Microcharops|[8048]|GMARB025-14|Hymenoptera|Ichneumonidae|Campopleginae|BOLD:ABA0304
Microcharops|[8049]|GMARL1409-14|Hymenoptera|Ichneumonidae|Campopleginae|BOLD:ACN3760
Microcharops|[8050]|GMARR903-14|Hymenoptera|Ichneumonidae|Campopleginae|BOLD:AAI1575
Hyposoter|[8051]|GMARA1646-14|Hymenoptera|Ichneumonidae|Campopleginae|BOLD:ACM5105
Ichneumonidae|[8052]|GMAGD111-15|Hymenoptera|Ichneumonidae||BOLD:ACW9656
Ichneumonidae|[8053]|GMARL1449-14|Hymenoptera|Ichneumonidae||BOLD:ACN2538
Ichneumonidae|[8054]|GMAGE1343-15|Hymenoptera|Ichneumonidae||BOLD:ACW6345
Ichneumonidae|[8055]|GMAGU117-15|Hymenoptera|Ichneumonidae||BOLD:ACX6930
Ichneumonidae|[8056]|GMARK2331-14|Hymenoptera|Ichneumonidae||BOLD:ACN5146
Ichneumonidae|[8057]|GMARR026-14|Hymenoptera|Ichneumonidae||BOLD:ACO0514
Ichneumonidae|[8058]|GMAGD1467-15|Hymenoptera|Ichneumonidae||BOLD:ACW2828
Ichneumonidae|[8059]|GMART047-14|Hymenoptera|Ichneumonidae||BOLD:ACO1982
Xiphosomella|[8060]|GMARG1753-14|Hymenoptera|Ichneumonidae|Cremastinae|BOLD:AAA7062
Xiphosomella|[8061]|GMARA1922-14|Hymenoptera|Ichneumonidae|Cremastinae|BOLD:ACN0430
Ichneumonidae|[8062]|GMARF1346-14|Hymenoptera|Ichneumonidae||BOLD:ACN0313
Ichneumonidae|[8063]|GMAGI1121-15|Hymenoptera|Ichneumonidae||BOLD:ACW6298
Ichneumonidae|[8064]|GMAGU771-15|Hymenoptera|Ichneumonidae||BOLD:ACX4659
[illegible]

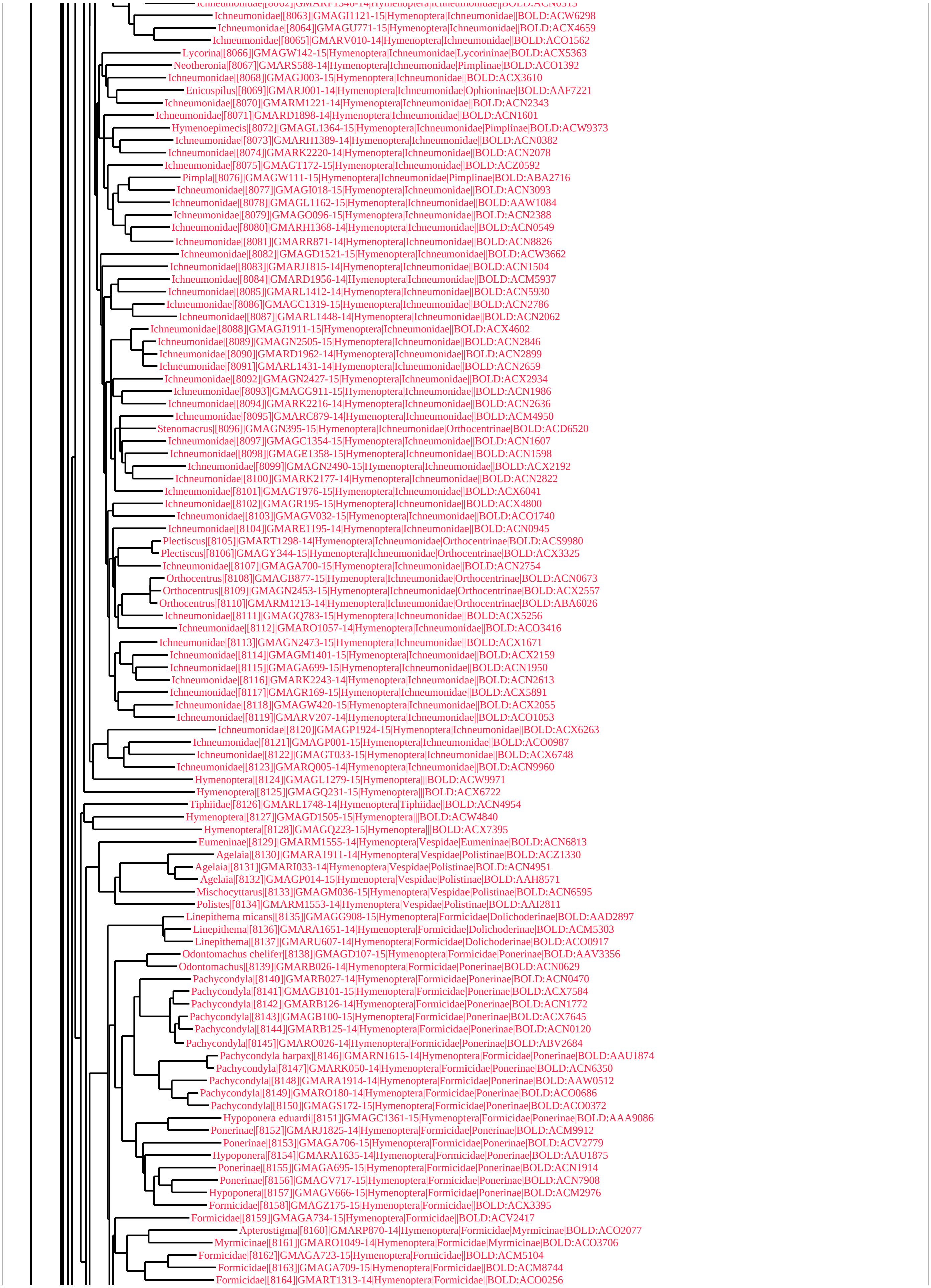
Ichneumonidae|[8062]|GMARF1346-14|Hymenoptera|Ichneumonidae||BOLD:ACN0313
Ichneumonidae|[8063]|GMAGI1121-15|Hymenoptera|Ichneumonidae||BOLD:ACW6298
Ichneumonidae|[8064]|GMAGU771-15|Hymenoptera|Ichneumonidae||BOLD:ACX4659
Ichneumonidae|[8065]|GMARV010-14|Hymenoptera|Ichneumonidae||BOLD:ACO1562
Lycorina|[8066]|GMAGW142-15|Hymenoptera|Ichneumonidae|Lycorininae|BOLD:ACX5363
Neotheronia|[8067]|GMARS588-14|Hymenoptera|Ichneumonidae|Pimplinae|BOLD:ACO1392
Ichneumonidae|[8068]|GMAGJ003-15|Hymenoptera|Ichneumonidae||BOLD:ACX3610
Enicospilus|[8069]|GMARJ001-14|Hymenoptera|Ichneumonidae|Ophioninae|BOLD:AAF7221
Ichneumonidae|[8070]|GMARM1221-14|Hymenoptera|Ichneumonidae||BOLD:ACN2343
Ichneumonidae|[8071]|GMARD1898-14|Hymenoptera|Ichneumonidae||BOLD:ACN1601
Hymenoepimecis|[8072]|GMAGL1364-15|Hymenoptera|Ichneumonidae|Pimplinae|BOLD:ACW9373
Ichneumonidae|[8073]|GMARH1389-14|Hymenoptera|Ichneumonidae||BOLD:ACN0382
Ichneumonidae|[8074]|GMARK2220-14|Hymenoptera|Ichneumonidae||BOLD:ACN2078
Ichneumonidae|[8075]|GMAGT172-15|Hymenoptera|Ichneumonidae||BOLD:ACZ0592
Pimpla|[8076]|GMAGW111-15|Hymenoptera|Ichneumonidae|Pimplinae|BOLD:ABA2716
Ichneumonidae|[8077]|GMAGI018-15|Hymenoptera|Ichneumonidae||BOLD:ACN3093
Ichneumonidae|[8078]|GMAGL1162-15|Hymenoptera|Ichneumonidae||BOLD:AAW1084
Ichneumonidae|[8079]|GMAGO096-15|Hymenoptera|Ichneumonidae||BOLD:ACN2388
Ichneumonidae|[8080]|GMARH1368-14|Hymenoptera|Ichneumonidae||BOLD:ACN0549
Ichneumonidae|[8081]|GMARR871-14|Hymenoptera|Ichneumonidae||BOLD:ACN8826
Ichneumonidae|[8082]|GMAGD1521-15|Hymenoptera|Ichneumonidae||BOLD:ACW3662
Ichneumonidae|[8083]|GMARJ1815-14|Hymenoptera|Ichneumonidae||BOLD:ACN1504
Ichneumonidae|[8084]|GMARD1956-14|Hymenoptera|Ichneumonidae||BOLD:ACM5937
Ichneumonidae|[8085]|GMARL1412-14|Hymenoptera|Ichneumonidae||BOLD:ACN5930
Ichneumonidae|[8086]|GMAGC1319-15|Hymenoptera|Ichneumonidae||BOLD:ACN2786
Ichneumonidae|[8087]|GMARL1448-14|Hymenoptera|Ichneumonidae||BOLD:ACN2062
Ichneumonidae|[8088]|GMAGJ1911-15|Hymenoptera|Ichneumonidae||BOLD:ACX4602
Ichneumonidae|[8089]|GMAGN2505-15|Hymenoptera|Ichneumonidae||BOLD:ACN2846
Ichneumonidae|[8090]|GMARD1962-14|Hymenoptera|Ichneumonidae||BOLD:ACN2899
Ichneumonidae|[8091]|GMARL1431-14|Hymenoptera|Ichneumonidae||BOLD:ACN2659
Ichneumonidae|[8092]|GMAGN2427-15|Hymenoptera|Ichneumonidae||BOLD:ACX2934
Ichneumonidae|[8093]|GMAGG911-15|Hymenoptera|Ichneumonidae||BOLD:ACN1986
Ichneumonidae|[8094]|GMARK2216-14|Hymenoptera|Ichneumonidae||BOLD:ACN2636
Ichneumonidae|[8095]|GMARC879-14|Hymenoptera|Ichneumonidae||BOLD:ACM4950
Stenomacrus|[8096]|GMAGN395-15|Hymenoptera|Ichneumonidae|Orthocentrinae|BOLD:ACD6520
Ichneumonidae|[8097]|GMAGC1354-15|Hymenoptera|Ichneumonidae||BOLD:ACN1607
Ichneumonidae|[8098]|GMAGE1358-15|Hymenoptera|Ichneumonidae||BOLD:ACN1598
Ichneumonidae|[8099]|GMAGN2490-15|Hymenoptera|Ichneumonidae||BOLD:ACX2192
Ichneumonidae|[8100]|GMARK2177-14|Hymenoptera|Ichneumonidae||BOLD:ACN2822
Ichneumonidae|[8101]|GMAGT976-15|Hymenoptera|Ichneumonidae||BOLD:ACX6041
Ichneumonidae|[8102]|GMAGR195-15|Hymenoptera|Ichneumonidae||BOLD:ACX4800
Ichneumonidae|[8103]|GMAGV032-15|Hymenoptera|Ichneumonidae||BOLD:ACO1740
Ichneumonidae|[8104]|GMARE1195-14|Hymenoptera|Ichneumonidae||BOLD:ACN0945
Plectiscus|[8105]|GMART1298-14|Hymenoptera|Ichneumonidae|Orthocentrinae|BOLD:ACS9980
Plectiscus|[8106]|GMAGY344-15|Hymenoptera|Ichneumonidae|Orthocentrinae|BOLD:ACX3325
Ichneumonidae|[8107]|GMAGA700-15|Hymenoptera|Ichneumonidae||BOLD:ACN2754
Orthocentrus|[8108]|GMAGB877-15|Hymenoptera|Ichneumonidae|Orthocentrinae|BOLD:ACN0673
Orthocentrus|[8109]|GMAGN2453-15|Hymenoptera|Ichneumonidae|Orthocentrinae|BOLD:ACX2557
Orthocentrus|[8110]|GMARM1213-14|Hymenoptera|Ichneumonidae|Orthocentrinae|BOLD:ABA6026
Ichneumonidae|[8111]|GMAGQ783-15|Hymenoptera|Ichneumonidae||BOLD:ACX5256
Ichneumonidae|[8112]|GMARO1057-14|Hymenoptera|Ichneumonidae||BOLD:ACO3416
Ichneumonidae|[8113]|GMAGN2473-15|Hymenoptera|Ichneumonidae||BOLD:ACX1671
Ichneumonidae|[8114]|GMAGM1401-15|Hymenoptera|Ichneumonidae||BOLD:ACX2159
Ichneumonidae|[8115]|GMAGA699-15|Hymenoptera|Ichneumonidae||BOLD:ACN1950
Ichneumonidae|[8116]|GMARK2243-14|Hymenoptera|Ichneumonidae||BOLD:ACN2613
Ichneumonidae|[8117]|GMAGR169-15|Hymenoptera|Ichneumonidae||BOLD:ACX5891
Ichneumonidae|[8118]|GMAGW420-15|Hymenoptera|Ichneumonidae||BOLD:ACX2055
Ichneumonidae|[8119]|GMARV207-14|Hymenoptera|Ichneumonidae||BOLD:ACO1053
Ichneumonidae|[8120]|GMAGP1924-15|Hymenoptera|Ichneumonidae||BOLD:ACX6263
Ichneumonidae|[8121]|GMAGP001-15|Hymenoptera|Ichneumonidae||BOLD:ACO0987
Ichneumonidae|[8122]|GMAGT033-15|Hymenoptera|Ichneumonidae||BOLD:ACX6748
Ichneumonidae|[8123]|GMARQ005-14|Hymenoptera|Ichneumonidae||BOLD:ACN9960
Hymenoptera|[8124]|GMAGL1279-15|Hymenoptera|||BOLD:ACW9971
Hymenoptera|[8125]|GMAGQ231-15|Hymenoptera|||BOLD:ACX6722
Tiphiidae|[8126]|GMARL1748-14|Hymenoptera|Tiphiidae||BOLD:ACN4954
Hymenoptera|[8127]|GMAGD1505-15|Hymenoptera|||BOLD:ACW4840
Hymenoptera|[8128]|GMAGQ223-15|Hymenoptera|||BOLD:ACX7395
Eumeninae|[8129]|GMARM1555-14|Hymenoptera|Vespidae|Eumeninae|BOLD:ACN6813
Agelaia|[8130]|GMARA1911-14|Hymenoptera|Vespidae|Polistinae|BOLD:ACZ1330
Agelaia|[8131]|GMARI033-14|Hymenoptera|Vespidae|Polistinae|BOLD:ACN4951
Agelaia|[8132]|GMAGP014-15|Hymenoptera|Vespidae|Polistinae|BOLD:AAH8571
Mischocyttarus|[8133]|GMAGM036-15|Hymenoptera|Vespidae|Polistinae|BOLD:ACN6595
Polistes|[8134]|GMARM1553-14|Hymenoptera|Vespidae|Polistinae|BOLD:AAI2811
Linepithema micans|[8135]|GMAGG908-15|Hymenoptera|Formicidae|Dolichoderinae|BOLD:AAD2897
Linepithema|[8136]|GMARA1651-14|Hymenoptera|Formicidae|Dolichoderinae|BOLD:ACM5303
Linepithema|[8137]|GMARU607-14|Hymenoptera|Formicidae|Dolichoderinae|BOLD:ACO0917
Odontomachus chelifer|[8138]|GMAGD107-15|Hymenoptera|Formicidae|Ponerinae|BOLD:AAV3356
Odontomachus|[8139]|GMARB026-14|Hymenoptera|Formicidae|Ponerinae|BOLD:ACN0629
Pachycondyla|[8140]|GMARB027-14|Hymenoptera|Formicidae|Ponerinae|BOLD:ACN0470
Pachycondyla|[8141]|GMAGB101-15|Hymenoptera|Formicidae|Ponerinae|BOLD:ACX7584
Pachycondyla|[8142]|GMARB126-14|Hymenoptera|Formicidae|Ponerinae|BOLD:ACN1772
Pachycondyla|[8143]|GMAGB100-15|Hymenoptera|Formicidae|Ponerinae|BOLD:ACX7645
Pachycondyla|[8144]|GMARB125-14|Hymenoptera|Formicidae|Ponerinae|BOLD:ACN0120
Pachycondyla|[8145]|GMARO026-14|Hymenoptera|Formicidae|Ponerinae|BOLD:ABV2684
Pachycondyla harpax|[8146]|GMARN1615-14|Hymenoptera|Formicidae|Ponerinae|BOLD:AAU1874
Pachycondyla|[8147]|GMARK050-14|Hymenoptera|Formicidae|Ponerinae|BOLD:ACN6350
Pachycondyla|[8148]|GMARA1914-14|Hymenoptera|Formicidae|Ponerinae|BOLD:AAW0512
Pachycondyla|[8149]|GMARO180-14|Hymenoptera|Formicidae|Ponerinae|BOLD:ACO0686
Pachycondyla|[8150]|GMAGS172-15|Hymenoptera|Formicidae|Ponerinae|BOLD:ACO0372
Hypoponera eduardi|[8151]|GMAGC1361-15|Hymenoptera|Formicidae|Ponerinae|BOLD:AAA9086
Ponerinae|[8152]|GMARJ1825-14|Hymenoptera|Formicidae|Ponerinae|BOLD:ACM9912
Ponerinae|[8153]|GMAGA706-15|Hymenoptera|Formicidae|Ponerinae|BOLD:ACV2779
Hypoponera|[8154]|GMARA1635-14|Hymenoptera|Formicidae|Ponerinae|BOLD:AAU1875
Ponerinae|[8155]|GMAGA695-15|Hymenoptera|Formicidae|Ponerinae|BOLD:ACN1914
Ponerinae|[8156]|GMAGV717-15|Hymenoptera|Formicidae|Ponerinae|BOLD:ACN7908
Hypoponera|[8157]|GMAGV666-15|Hymenoptera|Formicidae|Ponerinae|BOLD:ACM2976
Formicidae|[8158]|GMAGZ175-15|Hymenoptera|Formicidae||BOLD:ACX3395
Formicidae|[8159]|GMAGA734-15|Hymenoptera|Formicidae||BOLD:ACV2417
Apterostigma|[8160]|GMARP870-14|Hymenoptera|Formicidae|Myrmicinae|BOLD:ACO2077
Myrmicinae|[8161]|GMARO1049-14|Hymenoptera|Formicidae|Myrmicinae|BOLD:ACO3706
Formicidae|[8162]|GMAGA723-15|Hymenoptera|Formicidae||BOLD:ACM5104
Formicidae|[8163]|GMAGA709-15|Hymenoptera|Formicidae||BOLD:ACM8744
Formicidae|[8164]|GMART1313-14|Hymenoptera|Formicidae||BOLD:ACO0256

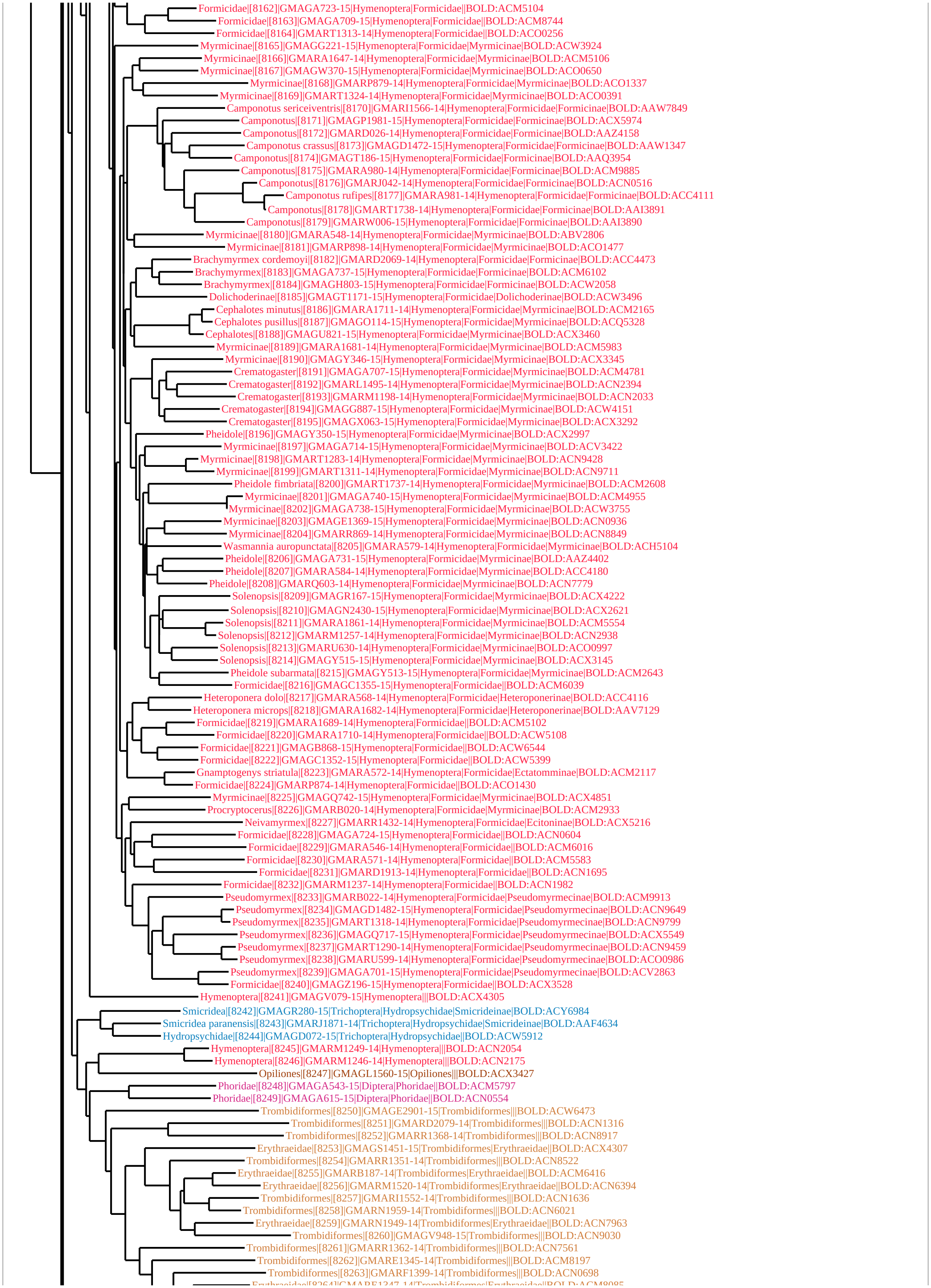
Formicidae|[8162]|GMAGA723-15|Hymenoptera|Formicidae||BOLD:ACM5104
Formicidae|[8163]|GMAGA709-15|Hymenoptera|Formicidae||BOLD:ACM8744
Formicidae|[8164]|GMART1313-14|Hymenoptera|Formicidae||BOLD:ACO0256
Myrmicinae|[8165]|GMAGG221-15|Hymenoptera|Formicidae|Myrmicinae|BOLD:ACW3924
Myrmicinae|[8166]|GMARA1647-14|Hymenoptera|Formicidae|Myrmicinae|BOLD:ACM5106
Myrmicinae|[8167]|GMAGW370-15|Hymenoptera|Formicidae|Myrmicinae|BOLD:ACO0650
Myrmicinae|[8168]|GMARP879-14|Hymenoptera|Formicidae|Myrmicinae|BOLD:ACO1337
Myrmicinae|[8169]|GMART1324-14|Hymenoptera|Formicidae|Myrmicinae|BOLD:ACO0391
Camponotus sericeiventris|[8170]|GMARI1566-14|Hymenoptera|Formicidae|Formicinae|BOLD:AAW7849
Camponotus|[8171]|GMAGP1981-15|Hymenoptera|Formicidae|Formicinae|BOLD:ACX5974
Camponotus|[8172]|GMARD026-14|Hymenoptera|Formicidae|Formicinae|BOLD:AAZ4158
Camponotus crassus|[8173]|GMAGD1472-15|Hymenoptera|Formicidae|Formicinae|BOLD:AAW1347
Camponotus|[8174]|GMAGT186-15|Hymenoptera|Formicidae|Formicinae|BOLD:AAQ3954
Camponotus|[8175]|GMARA980-14|Hymenoptera|Formicidae|Formicinae|BOLD:ACM9885
Camponotus|[8176]|GMARJ042-14|Hymenoptera|Formicidae|Formicinae|BOLD:ACN0516
Camponotus rufipes|[8177]|GMARA981-14|Hymenoptera|Formicidae|Formicinae|BOLD:ACC4111
Camponotus|[8178]|GMART1738-14|Hymenoptera|Formicidae|Formicinae|BOLD:AAI3891
Camponotus|[8179]|GMARW006-15|Hymenoptera|Formicidae|Formicinae|BOLD:AAI3890
Myrmicinae|[8180]|GMARA548-14|Hymenoptera|Formicidae|Myrmicinae|BOLD:ABV2806
Myrmicinae|[8181]|GMARP898-14|Hymenoptera|Formicidae|Myrmicinae|BOLD:ACO1477
Brachymyrmex cordemoyi|[8182]|GMARD2069-14|Hymenoptera|Formicidae|Formicinae|BOLD:ACC4473
Brachymyrmex|[8183]|GMAGA737-15|Hymenoptera|Formicidae|Formicinae|BOLD:ACM6102
Brachymyrmex|[8184]|GMAGH803-15|Hymenoptera|Formicidae|Formicinae|BOLD:ACW2058
Dolichoderinae|[8185]|GMAGT1171-15|Hymenoptera|Formicidae|Dolichoderinae|BOLD:ACW3496
Cephalotes minutus|[8186]|GMARA1711-14|Hymenoptera|Formicidae|Myrmicinae|BOLD:ACM2165
Cephalotes pusillus|[8187]|GMAGO114-15|Hymenoptera|Formicidae|Myrmicinae|BOLD:ACQ5328
Cephalotes|[8188]|GMAGU821-15|Hymenoptera|Formicidae|Myrmicinae|BOLD:ACX3460
Myrmicinae|[8189]|GMARA1681-14|Hymenoptera|Formicidae|Myrmicinae|BOLD:ACM5983
Myrmicinae|[8190]|GMAGY346-15|Hymenoptera|Formicidae|Myrmicinae|BOLD:ACX3345
Crematogaster|[8191]|GMAGA707-15|Hymenoptera|Formicidae|Myrmicinae|BOLD:ACM4781
Crematogaster|[8192]|GMARL1495-14|Hymenoptera|Formicidae|Myrmicinae|BOLD:ACN2394
Crematogaster|[8193]|GMARM1198-14|Hymenoptera|Formicidae|Myrmicinae|BOLD:ACN2033
Crematogaster|[8194]|GMAGG887-15|Hymenoptera|Formicidae|Myrmicinae|BOLD:ACW4151
Crematogaster|[8195]|GMAGX063-15|Hymenoptera|Formicidae|Myrmicinae|BOLD:ACX3292
Pheidole|[8196]|GMAGY350-15|Hymenoptera|Formicidae|Myrmicinae|BOLD:ACX2997
Myrmicinae|[8197]|GMAGA714-15|Hymenoptera|Formicidae|Myrmicinae|BOLD:ACV3422
Myrmicinae|[8198]|GMART1283-14|Hymenoptera|Formicidae|Myrmicinae|BOLD:ACN9428
Myrmicinae|[8199]|GMART1311-14|Hymenoptera|Formicidae|Myrmicinae|BOLD:ACN9711
Pheidole fimbriata|[8200]|GMART1737-14|Hymenoptera|Formicidae|Myrmicinae|BOLD:ACM2608
Myrmicinae|[8201]|GMAGA740-15|Hymenoptera|Formicidae|Myrmicinae|BOLD:ACM4955
Myrmicinae|[8202]|GMAGA738-15|Hymenoptera|Formicidae|Myrmicinae|BOLD:ACW3755
Myrmicinae|[8203]|GMAGE1369-15|Hymenoptera|Formicidae|Myrmicinae|BOLD:ACN0936
Myrmicinae|[8204]|GMARR869-14|Hymenoptera|Formicidae|Myrmicinae|BOLD:ACN8849
Wasmannia auropunctata|[8205]|GMARA579-14|Hymenoptera|Formicidae|Myrmicinae|BOLD:ACH5104
Pheidole|[8206]|GMAGA731-15|Hymenoptera|Formicidae|Myrmicinae|BOLD:AAZ4402
Pheidole|[8207]|GMARA584-14|Hymenoptera|Formicidae|Myrmicinae|BOLD:ACC4180
Pheidole|[8208]|GMARQ603-14|Hymenoptera|Formicidae|Myrmicinae|BOLD:ACN7779
Solenopsis|[8209]|GMAGR167-15|Hymenoptera|Formicidae|Myrmicinae|BOLD:ACX4222
Solenopsis|[8210]|GMAGN2430-15|Hymenoptera|Formicidae|Myrmicinae|BOLD:ACX2621
Solenopsis|[8211]|GMARA1861-14|Hymenoptera|Formicidae|Myrmicinae|BOLD:ACM5554
Solenopsis|[8212]|GMARM1257-14|Hymenoptera|Formicidae|Myrmicinae|BOLD:ACN2938
Solenopsis|[8213]|GMARU630-14|Hymenoptera|Formicidae|Myrmicinae|BOLD:ACO0997
Solenopsis|[8214]|GMAGY515-15|Hymenoptera|Formicidae|Myrmicinae|BOLD:ACX3145
Pheidole subarmata|[8215]|GMAGY513-15|Hymenoptera|Formicidae|Myrmicinae|BOLD:ACM2643
Formicidae|[8216]|GMAGC1355-15|Hymenoptera|Formicidae||BOLD:ACM6039
Heteroponera dolo|[8217]|GMARA568-14|Hymenoptera|Formicidae|Heteroponerinae|BOLD:ACC4116
Heteroponera microps|[8218]|GMARA1682-14|Hymenoptera|Formicidae|Heteroponerinae|BOLD:AAV7129
Formicidae|[8219]|GMARA1689-14|Hymenoptera|Formicidae||BOLD:ACM5102
Formicidae|[8220]|GMARA1710-14|Hymenoptera|Formicidae||BOLD:ACW5108
Formicidae|[8221]|GMAGB868-15|Hymenoptera|Formicidae||BOLD:ACW6544
Formicidae|[8222]|GMAGC1352-15|Hymenoptera|Formicidae||BOLD:ACW5399
Gnamptogenys striatula|[8223]|GMARA572-14|Hymenoptera|Formicidae|Ectatomminae|BOLD:ACM2117
Formicidae|[8224]|GMARP874-14|Hymenoptera|Formicidae||BOLD:ACO1430
Myrmicinae|[8225]|GMAGQ742-15|Hymenoptera|Formicidae|Myrmicinae|BOLD:ACX4851
Procryptocerus|[8226]|GMARB020-14|Hymenoptera|Formicidae|Myrmicinae|BOLD:ACM2933
Neivamyrmex|[8227]|GMARR1432-14|Hymenoptera|Formicidae|Ecitoninae|BOLD:ACX5216
Formicidae|[8228]|GMAGA724-15|Hymenoptera|Formicidae||BOLD:ACN0604
Formicidae|[8229]|GMARA546-14|Hymenoptera|Formicidae||BOLD:ACM6016
Formicidae|[8230]|GMARA571-14|Hymenoptera|Formicidae||BOLD:ACM5583
Formicidae|[8231]|GMARD1913-14|Hymenoptera|Formicidae||BOLD:ACN1695
Formicidae|[8232]|GMARM1237-14|Hymenoptera|Formicidae||BOLD:ACN1982
Pseudomyrmex|[8233]|GMARB022-14|Hymenoptera|Formicidae|Pseudomyrmecinae|BOLD:ACM9913
Pseudomyrmex|[8234]|GMAGD1482-15|Hymenoptera|Formicidae|Pseudomyrmecinae|BOLD:ACN9649
Pseudomyrmex|[8235]|GMART1318-14|Hymenoptera|Formicidae|Pseudomyrmecinae|BOLD:ACN9799
Pseudomyrmex|[8236]|GMAGQ717-15|Hymenoptera|Formicidae|Pseudomyrmecinae|BOLD:ACX5549
Pseudomyrmex|[8237]|GMART1290-14|Hymenoptera|Formicidae|Pseudomyrmecinae|BOLD:ACN9459
Pseudomyrmex|[8238]|GMARU599-14|Hymenoptera|Formicidae|Pseudomyrmecinae|BOLD:ACO0986
Pseudomyrmex|[8239]|GMAGA701-15|Hymenoptera|Formicidae|Pseudomyrmecinae|BOLD:ACV2863
Formicidae|[8240]|GMAGZ196-15|Hymenoptera|Formicidae||BOLD:ACX3528
Hymenoptera|[8241]|GMAGV079-15|Hymenoptera|||BOLD:ACX4305
Smicridea|[8242]|GMAGR280-15|Trichoptera|Hydropsychidae|Smicrideinae|BOLD:ACY6984
Smicridea paranensis|[8243]|GMARJ1871-14|Trichoptera|Hydropsychidae|Smicrideinae|BOLD:AAF4634
Hydropsychidae|[8244]|GMAGD072-15|Trichoptera|Hydropsychidae||BOLD:ACW5912
Hymenoptera|[8245]|GMARM1249-14|Hymenoptera|||BOLD:ACN2054
Hymenoptera|[8246]|GMARM1246-14|Hymenoptera|||BOLD:ACN2175
Opiliones|[8247]|GMAGL1560-15|Opiliones|||BOLD:ACX3427
Phoridae|[8248]|GMAGA543-15|Diptera|Phoridae||BOLD:ACM5797
Phoridae|[8249]|GMAGA615-15|Diptera|Phoridae||BOLD:ACN0554
Trombidiformes|[8250]|GMAGE2901-15|Trombidiformes|||BOLD:ACW6473
Trombidiformes|[8251]|GMARD2079-14|Trombidiformes|||BOLD:ACN1316
Trombidiformes|[8252]|GMARR1368-14|Trombidiformes|||BOLD:ACN8917
Erythraeidae|[8253]|GMAGS1451-15|Trombidiformes|Erythraeidae||BOLD:ACX4307
Trombidiformes|[8254]|GMARR1351-14|Trombidiformes|||BOLD:ACN8522
Erythraeidae|[8255]|GMARB187-14|Trombidiformes|Erythraeidae||BOLD:ACM6416
Erythraeidae|[8256]|GMARM1520-14|Trombidiformes|Erythraeidae||BOLD:ACN6394
Trombidiformes|[8257]|GMARI1552-14|Trombidiformes|||BOLD:ACN1636
Trombidiformes|[8258]|GMARN1959-14|Trombidiformes|||BOLD:ACN6021
Erythraeidae|[8259]|GMARN1949-14|Trombidiformes|Erythraeidae||BOLD:ACN7963
Trombidiformes|[8260]|GMAGV948-15|Trombidiformes|||BOLD:ACN9030
Trombidiformes|[8261]|GMARR1362-14|Trombidiformes|||BOLD:ACN7561
Trombidiformes|[8262]|GMARE1345-14|Trombidiformes|||BOLD:ACM8197
Trombidiformes|[8263]|GMARF1399-14|Trombidiformes|||BOLD:ACN0698

Trombidiformes|[8261]|GMARR1302-14|Trombidiformes|||BOLD:[illegible]
Trombidiformes|[8262]|GMARE1345-14|Trombidiformes|||BOLD:ACM8197
Trombidiformes|[8263]|GMARF1399-14|Trombidiformes|||BOLD:ACN0698
Erythraeidae|[8264]|GMARE1347-14|Trombidiformes|Erythraeidae||BOLD:ACM8085
Trombidiformes|[8265]|GMARM1506-14|Trombidiformes|||BOLD:ACN7251
Trombidiformes|[8266]|GMAGW531-15|Trombidiformes|||BOLD:ACX3313
Psyllidae|[8267]|GMAGA929-15|Hemiptera|Psyllidae||BOLD:ACM5342
Psyllidae|[8268]|GMAGO043-15|Hemiptera|Psyllidae||BOLD:ACX7050
Microgastrinae|[8269]|GMAGY330-15|Hymenoptera|Braconidae|Microgastrinae|BOLD:ACX1575
Microgastrinae|[8270]|GMAGE1352-15|Hymenoptera|Braconidae|Microgastrinae|BOLD:ACN1396
Glyptapanteles|[8271]|GMARS491-14|Hymenoptera|Braconidae|Microgastrinae|BOLD:AAA4564
Microgastrinae|[8272]|GMAGE1348-15|Hymenoptera|Braconidae|Microgastrinae|BOLD:ACM9970
Microgastrinae|[8273]|GMAGO082-15|Hymenoptera|Braconidae|Microgastrinae|BOLD:ACN2284
Alphomelon|[8274]|GMAGX091-15|Hymenoptera|Braconidae|Microgastrinae|BOLD:AAB8584
Microgastrinae|[8275]|GMAGP1987-15|Hymenoptera|Braconidae|Microgastrinae|BOLD:ACX4915
Microgastrinae|[8276]|GMAGN2445-15|Hymenoptera|Braconidae|Microgastrinae|BOLD:ACX3261
Apanteles|[8277]|GMARC884-14|Hymenoptera|Braconidae|Microgastrinae|BOLD:ACM5674
Apanteles|[8278]|GMAGP1914-15|Hymenoptera|Braconidae|Microgastrinae|BOLD:ACN2113
Microgastrinae|[8279]|GMAGP1990-15|Hymenoptera|Braconidae|Microgastrinae|BOLD:ACX4510
Microgastrinae|[8280]|GMARB241-14|Hymenoptera|Braconidae|Microgastrinae|BOLD:ACM6065
Apanteles javiercontrerasi|[8281]|GMARF1244-14|Hymenoptera|Braconidae|Microgastrinae|BOLD:ACF1627
Apanteles|[8282]|GMARM1235-14|Hymenoptera|Braconidae|Microgastrinae|BOLD:ACN2508
Microgastrinae|[8283]|GMARE1201-14|Hymenoptera|Braconidae|Microgastrinae|BOLD:ACN1507
Microgastrinae|[8284]|GMARA1650-14|Hymenoptera|Braconidae|Microgastrinae|BOLD:ACM5107
Microgastrinae|[8285]|GMAGO087-15|Hymenoptera|Braconidae|Microgastrinae|BOLD:ACN2711
Apanteles|[8286]|GMARC875-14|Hymenoptera|Braconidae|Microgastrinae|BOLD:ABW5846
Microgastrinae|[8287]|GMAGT972-15|Hymenoptera|Braconidae|Microgastrinae|BOLD:ACX1985
Parapanteles|[8288]|GMAGI1133-15|Hymenoptera|Braconidae|Microgastrinae|BOLD:AAJ1367
Microgastrinae|[8289]|GMARJ1829-14|Hymenoptera|Braconidae|Microgastrinae|BOLD:ACM9914
Braconidae|[8290]|GMAGM1384-15|Hymenoptera|Braconidae||BOLD:ACX3912
Microgastrinae|[8291]|GMAGB860-15|Hymenoptera|Braconidae|Microgastrinae|BOLD:ACW6631
Microgastrinae|[8292]|GMARH1373-14|Hymenoptera|Braconidae|Microgastrinae|BOLD:ACN0620
Microgastrinae|[8293]|GMAGL1286-15|Hymenoptera|Braconidae|Microgastrinae|BOLD:ACX5418
Diolcogaster|[8294]|GMAGQ726-15|Hymenoptera|Braconidae|Microgastrinae|BOLD:ACX4182
Microgastrinae|[8295]|GMARL1430-14|Hymenoptera|Braconidae|Microgastrinae|BOLD:ACN2459
Microgastrinae|[8296]|GMAGP1967-15|Hymenoptera|Braconidae|Microgastrinae|BOLD:ACX5934
Hypomicrogaster|[8297]|GMAGN2442-15|Hymenoptera|Braconidae|Microgastrinae|BOLD:ACX3194
Hypomicrogaster|[8298]|GMAGL1284-15|Hymenoptera|Braconidae|Microgastrinae|BOLD:ACX8209
Hypomicrogaster|[8299]|GMAGN2463-15|Hymenoptera|Braconidae|Microgastrinae|BOLD:ACX1765
Glyptapanteles|[8300]|GMAGH836-15|Hymenoptera|Braconidae|Microgastrinae|BOLD:AAD1799
Glyptapanteles|[8301]|GMARA1684-14|Hymenoptera|Braconidae|Microgastrinae|BOLD:ACM4968
Glyptapanteles|[8302]|GMARA1688-14|Hymenoptera|Braconidae|Microgastrinae|BOLD:ACM5453
Glyptapanteles|[8303]|GMAGF693-15|Hymenoptera|Braconidae|Microgastrinae|BOLD:ACW4460
Glyptapanteles|[8304]|GMAGM1383-15|Hymenoptera|Braconidae|Microgastrinae|BOLD:AAL3667
Glyptapanteles|[8305]|GMAGN2510-15|Hymenoptera|Braconidae|Microgastrinae|BOLD:ACX2623
Glyptapanteles|[8306]|GMARE1208-14|Hymenoptera|Braconidae|Microgastrinae|BOLD:ACN1492
Glyptapanteles|[8307]|GMARA544-14|Hymenoptera|Braconidae|Microgastrinae|BOLD:ACM9409
Glyptapanteles|[8308]|GMARK2253-14|Hymenoptera|Braconidae|Microgastrinae|BOLD:ACN2246
Glyptapanteles|[8309]|GMAGE1360-15|Hymenoptera|Braconidae|Microgastrinae|BOLD:ACW3827
Glyptapanteles|[8310]|GMAGN2428-15|Hymenoptera|Braconidae|Microgastrinae|BOLD:ACX1474
Glyptapanteles|[8311]|GMARK2202-14|Hymenoptera|Braconidae|Microgastrinae|BOLD:ACN2205
Glyptapanteles|[8312]|GMARL1458-14|Hymenoptera|Braconidae|Microgastrinae|BOLD:AAM1082
Glyptapanteles nealweberi|[8313]|GMART1304-14|Hymenoptera|Braconidae|Microgastrinae|BOLD:ACE4132
Glyptapanteles|[8314]|GMAGI1123-15|Hymenoptera|Braconidae|Microgastrinae|BOLD:ACW6737
Glyptapanteles|[8315]|GMAGP1949-15|Hymenoptera|Braconidae|Microgastrinae|BOLD:ACX6235
Glyptapanteles|[8316]|GMAGE1322-15|Hymenoptera|Braconidae|Microgastrinae|BOLD:ACN2367
Glyptapanteles|[8317]|GMAGH825-15|Hymenoptera|Braconidae|Microgastrinae|BOLD:ACW1966
Glyptapanteles|[8318]|GMART1331-14|Hymenoptera|Braconidae|Microgastrinae|BOLD:ACN9918
Microgastrinae|[8319]|GMAGF675-15|Hymenoptera|Braconidae|Microgastrinae|BOLD:ACN0272
Microgastrinae|[8320]|GMARK2199-14|Hymenoptera|Braconidae|Microgastrinae|BOLD:ACN2960
Microgastrinae|[8321]|GMARI1412-14|Hymenoptera|Braconidae|Microgastrinae|BOLD:ACN1181
Microgastrinae|[8322]|GMAGQ745-15|Hymenoptera|Braconidae|Microgastrinae|BOLD:ACN3557
Microgastrinae|[8323]|GMARR955-14|Hymenoptera|Braconidae|Microgastrinae|BOLD:ACN8530
Cotesia|[8324]|GMAGN2448-15|Hymenoptera|Braconidae|Microgastrinae|BOLD:ACN2430
Cotesia|[8325]|GMARK2178-14|Hymenoptera|Braconidae|Microgastrinae|BOLD:ACN2021
Cotesia|[8326]|GMARR866-14|Hymenoptera|Braconidae|Microgastrinae|BOLD:ABY6803
Cotesia|[8327]|GMAGO108-15|Hymenoptera|Braconidae|Microgastrinae|BOLD:ACX5238
Parapanteles scotti|[8328]|GMAGN2469-15|Hymenoptera|Braconidae|Microgastrinae|BOLD:AAB9141
Cotesia|[8329]|GMARJ1821-14|Hymenoptera|Braconidae|Microgastrinae|BOLD:ACN1700
Cotesia|[8330]|GMAGD1515-15|Hymenoptera|Braconidae|Microgastrinae|BOLD:ACM5743
Cotesia|[8331]|GMAGP1959-15|Hymenoptera|Braconidae|Microgastrinae|BOLD:ACX5579
Cotesia|[8332]|GMARR859-14|Hymenoptera|Braconidae|Microgastrinae|BOLD:ACN7733
Cotesia|[8333]|GMARS494-14|Hymenoptera|Braconidae|Microgastrinae|BOLD:ACN8967
Braconidae|[8334]|GMAGJ015-15|Hymenoptera|Braconidae||BOLD:ACX5570
Microgastrinae|[8335]|GMAGP1964-15|Hymenoptera|Braconidae|Microgastrinae|BOLD:ACX6313
Microgastrinae|[8336]|GMARI1423-14|Hymenoptera|Braconidae|Microgastrinae|BOLD:ACN0855
Microgastrinae|[8337]|GMARO1068-14|Hymenoptera|Braconidae|Microgastrinae|BOLD:ACO3453
Diolcogaster|[8338]|GMARR862-14|Hymenoptera|Braconidae|Microgastrinae|BOLD:ACN8418
Microgastrinae|[8339]|GMARH1369-14|Hymenoptera|Braconidae|Microgastrinae|BOLD:ACN0061
Diolcogaster|[8340]|GMAGF689-15|Hymenoptera|Braconidae|Microgastrinae|BOLD:ACX2459
Diolcogaster|[8341]|GMAGN2488-15|Hymenoptera|Braconidae|Microgastrinae|BOLD:ACN2957
Microgastrinae|[8342]|GMAGQ758-15|Hymenoptera|Braconidae|Microgastrinae|BOLD:ACX5507
Microgastrinae|[8343]|GMAGV691-15|Hymenoptera|Braconidae|Microgastrinae|BOLD:ACN7502
Microgastrinae|[8344]|GMAGJ014-15|Hymenoptera|Braconidae|Microgastrinae|BOLD:ACN1603
Braconidae|[8345]|GMAGU789-15|Hymenoptera|Braconidae||BOLD:ACX4223
Braconidae|[8346]|GMARJ1827-14|Hymenoptera|Braconidae||BOLD:ACN0223
Braconidae|[8347]|GMARK2208-14|Hymenoptera|Braconidae||BOLD:ACN2082
Braconidae|[8348]|GMAGV696-15|Hymenoptera|Braconidae||BOLD:ACX2463
Braconidae|[8349]|GMAGZ176-15|Hymenoptera|Braconidae||BOLD:ACX3502
Braconidae|[8350]|GMARE1215-14|Hymenoptera|Braconidae||BOLD:ACQ9649
Braconidae|[8351]|GMARL1445-14|Hymenoptera|Braconidae||BOLD:ACN2437
Braconidae|[8352]|GMAGN2461-15|Hymenoptera|Braconidae||BOLD:ACN2714
Braconidae|[8353]|GMAGQ720-15|Hymenoptera|Braconidae||BOLD:ACX5567
Braconidae|[8354]|GMARB250-14|Hymenoptera|Braconidae||BOLD:ACM6067
Braconidae|[8355]|GMAGL1358-15|Hymenoptera|Braconidae||BOLD:ACN2335
Braconidae|[8356]|GMAGO111-15|Hymenoptera|Braconidae||BOLD:ACX4413
Braconidae|[8357]|GMARB301-14|Hymenoptera|Braconidae||BOLD:ACM6219
Braconidae|[8358]|GMAGJ040-15|Hymenoptera|Braconidae||BOLD:ACN2320
Braconidae|[8359]|GMARL1487-14|Hymenoptera|Braconidae||BOLD:ACN2087
Braconidae|[8360]|GMAGT979-15|Hymenoptera|Braconidae||BOLD:ACX5833
Braconidae|[8361]|GMAGO100-15|Hymenoptera|Braconidae||BOLD:ACX5932
Braconidae|[8362]|GMAGI1131-15|Hymenoptera|Braconidae||BOLD:ACW7392
Braconidae|[8363]|GMAGP1926-15|Hymenoptera|Braconidae||BOLD:ACX6264

Braconidae|[8361]|GMAGO100-15|Hymenoptera|Braconidae||BOLD:ACX5932
Braconidae|[8362]|GMAGI1131-15|Hymenoptera|Braconidae||BOLD:ACW7392
Braconidae|[8363]|GMAGP1926-15|Hymenoptera|Braconidae||BOLD:ACX6264
Braconidae|[8364]|GMAGP1943-15|Hymenoptera|Braconidae||BOLD:ACX5910
Braconidae|[8365]|GMARN1590-14|Hymenoptera|Braconidae||BOLD:ACN4528
Meteorus|[8366]|GMARF1347-14|Hymenoptera|Braconidae|Euphorinae|BOLD:AAD6012
Braconidae|[8367]|GMAGY333-15|Hymenoptera|Braconidae||BOLD:ACX1565
Braconidae|[8368]|GMAGA702-15|Hymenoptera|Braconidae||BOLD:ACV3432
Braconidae|[8369]|GMAGN2451-15|Hymenoptera|Braconidae||BOLD:ACX2542
Braconidae|[8370]|GMARN1550-14|Hymenoptera|Braconidae||BOLD:ACN7147
Aphidius|[8371]|GMARO1080-14|Hymenoptera|Braconidae|Aphidiinae|BOLD:AAJ1250
Aphidiinae|[8372]|GMAGL1288-15|Hymenoptera|Braconidae|Aphidiinae|BOLD:ACM9915
Braconidae|[8373]|GMARA1699-14|Hymenoptera|Braconidae||BOLD:ACM5452
Braconidae|[8374]|GMARF1259-14|Hymenoptera|Braconidae||BOLD:ACN0171
Braconidae|[8375]|GMAGU781-15|Hymenoptera|Braconidae||BOLD:ACX4303
Braconidae|[8376]|GMAGC1384-15|Hymenoptera|Braconidae||BOLD:ACW4944
Braconidae|[8377]|GMAGB942-15|Hymenoptera|Braconidae||BOLD:ACN0894
Braconidae|[8378]|GMAGI1149-15|Hymenoptera|Braconidae||BOLD:ACN1759
Braconidae|[8379]|GMAGC1328-15|Hymenoptera|Braconidae||BOLD:ACW5031
Braconidae|[8380]|GMAGL1317-15|Hymenoptera|Braconidae||BOLD:ACW9081
Braconidae|[8381]|GMARM1244-14|Hymenoptera|Braconidae||BOLD:ACN2841
Braconidae|[8382]|GMARF1247-14|Hymenoptera|Braconidae||BOLD:ACN1272
Braconidae|[8383]|GMARC862-14|Hymenoptera|Braconidae||BOLD:ACM4712
Mirax|[8384]|GMAGG905-15|Hymenoptera|Braconidae|Miracinae|BOLD:ACK2796
Braconidae|[8385]|GMAGO088-15|Hymenoptera|Braconidae||BOLD:ACX4655
Braconidae|[8386]|GMAGW406-15|Hymenoptera|Braconidae||BOLD:ACX1324
Venanides|[8387]|GMAGO083-15|Hymenoptera|Braconidae|Microgastrinae|BOLD:ACX5835
Venanides|[8388]|GMAGX092-15|Hymenoptera|Braconidae|Microgastrinae|BOLD:AAW7325
Ichneutinae|[8389]|GMARL1491-14|Hymenoptera|Braconidae|Ichneutinae|BOLD:ACN2398
Braconidae|[8390]|GMAGB878-15|Hymenoptera|Braconidae||BOLD:ACW3165
Braconidae|[8391]|GMARL1514-14|Hymenoptera|Braconidae||BOLD:ACN2840
Braconidae|[8392]|GMARN1549-14|Hymenoptera|Braconidae||BOLD:ACN3933
Braconidae|[8393]|GMAGH832-15|Hymenoptera|Braconidae||BOLD:ACW1740
Braconidae|[8394]|GMAGQ746-15|Hymenoptera|Braconidae||BOLD:ACO3672
Braconidae|[8395]|GMART1261-14|Hymenoptera|Braconidae||BOLD:ACN9555
Braconidae|[8396]|GMARI035-14|Hymenoptera|Braconidae||BOLD:ACN7289
Heteropteron|[8397]|GMARB130-14|Hymenoptera|Braconidae|Cardiochilinae|BOLD:ACN0431
Macrocentrus|[8398]|GMAGK062-15|Hymenoptera|Braconidae|Macrocentrinae|BOLD:ACK7466
Braconidae|[8399]|GMARB024-14|Hymenoptera|Braconidae||BOLD:ACN1347
Braconidae|[8400]|GMAGI1119-15|Hymenoptera|Braconidae||BOLD:ACW7618
Braconidae|[8401]|GMAGE1315-15|Hymenoptera|Braconidae||BOLD:ACW7492
Braconidae|[8402]|GMAGS181-15|Hymenoptera|Braconidae||BOLD:ACO1697
Braconidae|[8403]|GMAGE1323-15|Hymenoptera|Braconidae||BOLD:ACM5925
Braconidae|[8404]|GMAGE1332-15|Hymenoptera|Braconidae||BOLD:ACW2696
Braconidae|[8405]|GMARF1253-14|Hymenoptera|Braconidae||BOLD:ACN1829
Braconidae|[8406]|GMARR873-14|Hymenoptera|Braconidae||BOLD:ACN8576
Braconidae|[8407]|GMAGX059-15|Hymenoptera|Braconidae||BOLD:ACO1135
Aphidiinae|[8408]|GMAGP1950-15|Hymenoptera|Braconidae|Aphidiinae|BOLD:ACX5497
Braconidae|[8409]|GMARN1555-14|Hymenoptera|Braconidae||BOLD:ACN6284
Braconidae|[8410]|GMARN1669-14|Hymenoptera|Braconidae||BOLD:ACN3369
Braconidae|[8411]|GMAGW345-15|Hymenoptera|Braconidae||BOLD:ACX3322
Braconidae|[8412]|GMARE1187-14|Hymenoptera|Braconidae||BOLD:ACN1847
Braconidae|[8413]|GMAGI1162-15|Hymenoptera|Braconidae||BOLD:ACW6471
Braconidae|[8414]|GMAGB865-15|Hymenoptera|Braconidae||BOLD:ACO1045
Braconidae|[8415]|GMAGD1475-15|Hymenoptera|Braconidae||BOLD:ACW3369
Braconidae|[8416]|GMAGF093-15|Hymenoptera|Braconidae||BOLD:ACN1311
Braconidae|[8417]|GMARK2330-14|Hymenoptera|Braconidae||BOLD:ACN4541
Braconidae|[8418]|GMAGG192-15|Hymenoptera|Braconidae||BOLD:ACW4202
Mesostoinae|[8419]|GMARB290-14|Hymenoptera|Braconidae|Mesostoinae|BOLD:ACM6218
Mesostoinae|[8420]|GMAGG902-15|Hymenoptera|Braconidae|Mesostoinae|BOLD:ACW3998
Mesostoinae|[8421]|GMAGN2484-15|Hymenoptera|Braconidae|Mesostoinae|BOLD:ACX3133
Mesostoinae|[8422]|GMAGP1975-15|Hymenoptera|Braconidae|Mesostoinae|BOLD:ACO3462
Mesostoinae|[8423]|GMAGM1593-15|Hymenoptera|Braconidae|Mesostoinae|BOLD:ACN0582
Braconidae|[8424]|GMAGR190-15|Hymenoptera|Braconidae||BOLD:ACX5976
Braconidae|[8425]|GMARU034-14|Hymenoptera|Braconidae||BOLD:ACO1788
Braconidae|[8426]|GMARM1567-14|Hymenoptera|Braconidae||BOLD:ACN6259
Braconidae|[8427]|GMAGZ077-15|Hymenoptera|Braconidae||BOLD:ACX6043
Braconidae|[8428]|GMARB023-14|Hymenoptera|Braconidae||BOLD:ACM9893
Aleiodes|[8429]|GMARE1185-14|Hymenoptera|Braconidae|Rogadinae|BOLD:ACN1837
Aleiodes|[8430]|GMAGP1961-15|Hymenoptera|Braconidae|Rogadinae|BOLD:ACN2401
Aleiodes|[8431]|GMAGO116-15|Hymenoptera|Braconidae|Rogadinae|BOLD:ACX5933
Aleiodes|[8432]|GMAGS1126-15|Hymenoptera|Braconidae|Rogadinae|BOLD:AAV6240
Aleiodes|[8433]|GMAGE1356-15|Hymenoptera|Braconidae|Rogadinae|BOLD:ACN8970
Braconidae|[8434]|GMART1301-14|Hymenoptera|Braconidae||BOLD:ACO0525
Braconidae|[8435]|GMAGL1362-15|Hymenoptera|Braconidae||BOLD:ACW9551
Braconidae|[8436]|GMAGB964-15|Hymenoptera|Braconidae||BOLD:ACW7792
Braconidae|[8437]|GMART1287-14|Hymenoptera|Braconidae||BOLD:ACO0249
Braconidae|[8438]|GMART1270-14|Hymenoptera|Braconidae||BOLD:ACN9144
Braconidae|[8439]|GMARA1642-14|Hymenoptera|Braconidae||BOLD:ACM5103
Braconidae|[8440]|GMARI1410-14|Hymenoptera|Braconidae||BOLD:ACN0447
Heterospilus|[8441]|GMAGY340-15|Hymenoptera|Braconidae|Doryctinae|BOLD:ACX3246
Braconidae|[8442]|GMARG1913-14|Hymenoptera|Braconidae||BOLD:ACN0501
Braconidae|[8443]|GMARR875-14|Hymenoptera|Braconidae||BOLD:ACN8029
Braconidae|[8444]|GMAGJ046-15|Hymenoptera|Braconidae||BOLD:ACX5657
Braconidae|[8445]|GMARM1188-14|Hymenoptera|Braconidae||BOLD:ACN2109
Braconidae|[8446]|GMAGD1517-15|Hymenoptera|Braconidae||BOLD:ACW2987
Braconidae|[8447]|GMAGI1154-15|Hymenoptera|Braconidae||BOLD:ACW6044
Braconidae|[8448]|GMAGX107-15|Hymenoptera|Braconidae||BOLD:ACX2362
Doryctinae|[8449]|GMARL1438-14|Hymenoptera|Braconidae|Doryctinae|BOLD:ACN2414
Braconidae|[8450]|GMAGB910-15|Hymenoptera|Braconidae||BOLD:ACW6766
Braconidae|[8451]|GMAGO086-15|Hymenoptera|Braconidae||BOLD:ACX5141
Braconidae|[8452]|GMART1297-14|Hymenoptera|Braconidae||BOLD:ACO0176
Braconidae|[8453]|GMARG1755-14|Hymenoptera|Braconidae||BOLD:ACN1497
Braconidae|[8454]|GMAGI1135-15|Hymenoptera|Braconidae||BOLD:ACW8046
Braconidae|[8455]|GMAGK1849-15|Hymenoptera|Braconidae||BOLD:ACX5945
Braconidae|[8456]|GMARN1567-14|Hymenoptera|Braconidae||BOLD:ACN3930
Braconidae|[8457]|GMAGL1303-15|Hymenoptera|Braconidae||BOLD:ACW9086
Braconidae|[8458]|GMARH1371-14|Hymenoptera|Braconidae||BOLD:ACN0307
Braconidae|[8459]|GMARK2519-14|Hymenoptera|Braconidae||BOLD:ACN2721
Braconidae|[8460]|GMAGE1341-15|Hymenoptera|Braconidae||BOLD:ACW7471
Braconidae|[8461]|GMARF1286-14|Hymenoptera|Braconidae||BOLD:ACN1864
Braconidae|[8462]|GMAGL1335-15|Hymenoptera|Braconidae||BOLD:ACW8833
Braconidae|[8463]|GMAGD1508-15|Hymenoptera|Braconidae||BOLD:ACW3771

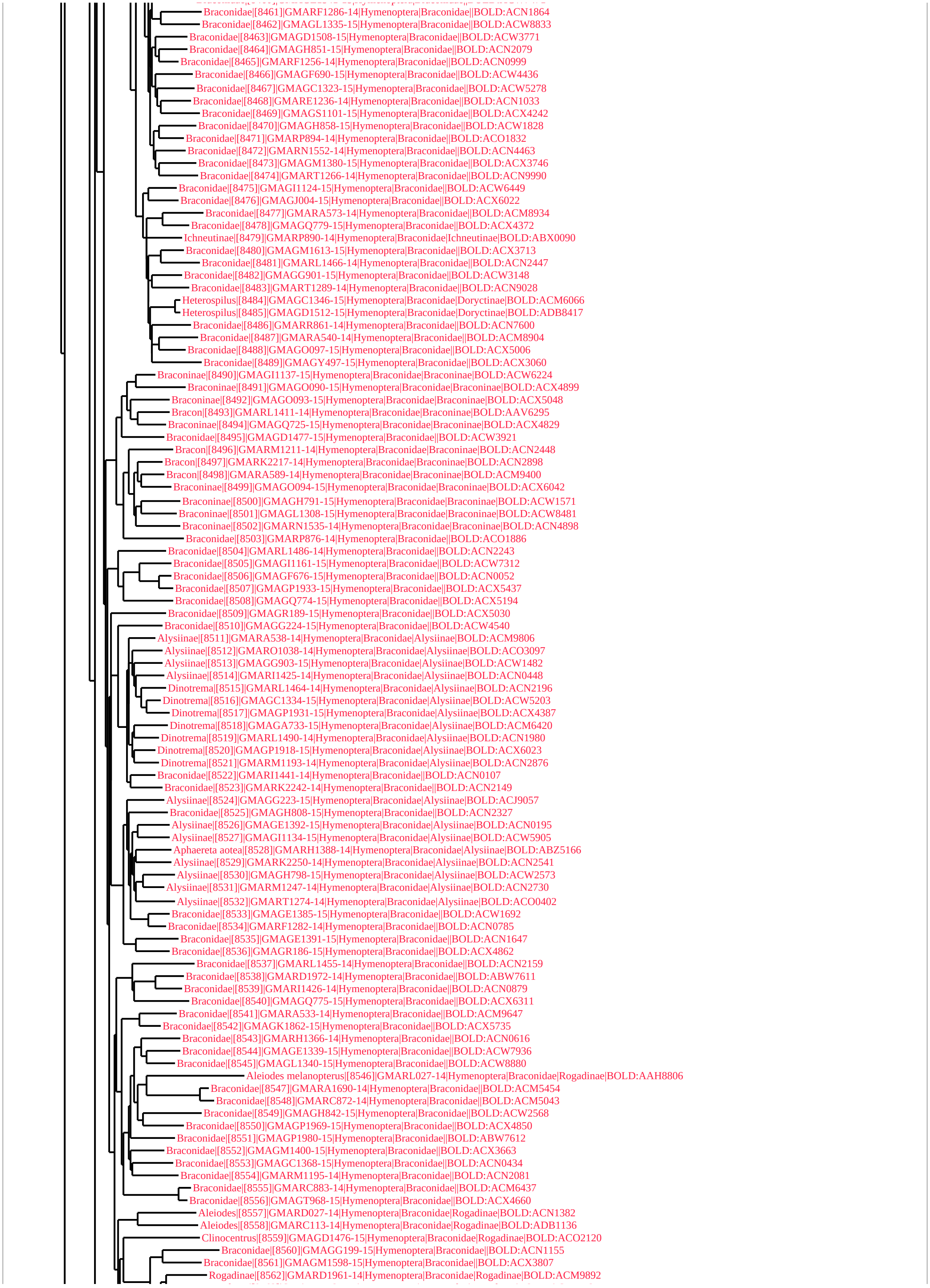
Braconidae|[8461]|GMARF1286-14|Hymenoptera|Braconidae||BOLD:ACN1864
Braconidae|[8462]|GMAGL1335-15|Hymenoptera|Braconidae||BOLD:ACW8833
Braconidae|[8463]|GMAGD1508-15|Hymenoptera|Braconidae||BOLD:ACW3771
Braconidae|[8464]|GMAGH851-15|Hymenoptera|Braconidae||BOLD:ACN2079
Braconidae|[8465]|GMARF1256-14|Hymenoptera|Braconidae||BOLD:ACN0999
Braconidae|[8466]|GMAGF690-15|Hymenoptera|Braconidae||BOLD:ACW4436
Braconidae|[8467]|GMAGC1323-15|Hymenoptera|Braconidae||BOLD:ACW5278
Braconidae|[8468]|GMARE1236-14|Hymenoptera|Braconidae||BOLD:ACN1033
Braconidae|[8469]|GMAGS1101-15|Hymenoptera|Braconidae||BOLD:ACX4242
Braconidae|[8470]|GMAGH858-15|Hymenoptera|Braconidae||BOLD:ACW1828
Braconidae|[8471]|GMARP894-14|Hymenoptera|Braconidae||BOLD:ACO1832
Braconidae|[8472]|GMARN1552-14|Hymenoptera|Braconidae||BOLD:ACN4463
Braconidae|[8473]|GMAGM1380-15|Hymenoptera|Braconidae||BOLD:ACX3746
Braconidae|[8474]|GMART1266-14|Hymenoptera|Braconidae||BOLD:ACN9990
Braconidae|[8475]|GMAGI1124-15|Hymenoptera|Braconidae||BOLD:ACW6449
Braconidae|[8476]|GMAGJ004-15|Hymenoptera|Braconidae||BOLD:ACX6022
Braconidae|[8477]|GMARA573-14|Hymenoptera|Braconidae||BOLD:ACM8934
Braconidae|[8478]|GMAGQ779-15|Hymenoptera|Braconidae||BOLD:ACX4372
Ichneutinae|[8479]|GMARP890-14|Hymenoptera|Braconidae|Ichneutinae|BOLD:ABX0090
Braconidae|[8480]|GMAGM1613-15|Hymenoptera|Braconidae||BOLD:ACX3713
Braconidae|[8481]|GMARL1466-14|Hymenoptera|Braconidae||BOLD:ACN2447
Braconidae|[8482]|GMAGG901-15|Hymenoptera|Braconidae||BOLD:ACW3148
Braconidae|[8483]|GMART1289-14|Hymenoptera|Braconidae||BOLD:ACN9028
Heterospilus|[8484]|GMAGC1346-15|Hymenoptera|Braconidae|Doryctinae|BOLD:ACM6066
Heterospilus|[8485]|GMAGD1512-15|Hymenoptera|Braconidae|Doryctinae|BOLD:ADB8417
Braconidae|[8486]|GMARR861-14|Hymenoptera|Braconidae||BOLD:ACN7600
Braconidae|[8487]|GMARA540-14|Hymenoptera|Braconidae||BOLD:ACM8904
Braconidae|[8488]|GMAGO097-15|Hymenoptera|Braconidae||BOLD:ACX5006
Braconidae|[8489]|GMAGY497-15|Hymenoptera|Braconidae||BOLD:ACX3060
Braconinae|[8490]|GMAGI1137-15|Hymenoptera|Braconidae|Braconinae|BOLD:ACW6224
Braconinae|[8491]|GMAGO090-15|Hymenoptera|Braconidae|Braconinae|BOLD:ACX4899
Braconinae|[8492]|GMAGO093-15|Hymenoptera|Braconidae|Braconinae|BOLD:ACX5048
Bracon|[8493]|GMARL1411-14|Hymenoptera|Braconidae|Braconinae|BOLD:AAV6295
Braconinae|[8494]|GMAGQ725-15|Hymenoptera|Braconidae|Braconinae|BOLD:ACX4829
Braconidae|[8495]|GMAGD1477-15|Hymenoptera|Braconidae||BOLD:ACW3921
Bracon|[8496]|GMARM1211-14|Hymenoptera|Braconidae|Braconinae|BOLD:ACN2448
Bracon|[8497]|GMARK2217-14|Hymenoptera|Braconidae|Braconinae|BOLD:ACN2898
Bracon|[8498]|GMARA589-14|Hymenoptera|Braconidae|Braconinae|BOLD:ACM9400
Braconinae|[8499]|GMAGO094-15|Hymenoptera|Braconidae|Braconinae|BOLD:ACX6042
Braconinae|[8500]|GMAGH791-15|Hymenoptera|Braconidae|Braconinae|BOLD:ACW1571
Braconinae|[8501]|GMAGL1308-15|Hymenoptera|Braconidae|Braconinae|BOLD:ACW8481
Braconinae|[8502]|GMARN1535-14|Hymenoptera|Braconidae|Braconinae|BOLD:ACN4898
Braconidae|[8503]|GMARP876-14|Hymenoptera|Braconidae||BOLD:ACO1886
Braconidae|[8504]|GMARL1486-14|Hymenoptera|Braconidae||BOLD:ACN2243
Braconidae|[8505]|GMAGI1161-15|Hymenoptera|Braconidae||BOLD:ACW7312
Braconidae|[8506]|GMAGF676-15|Hymenoptera|Braconidae||BOLD:ACN0052
Braconidae|[8507]|GMAGP1933-15|Hymenoptera|Braconidae||BOLD:ACX5437
Braconidae|[8508]|GMAGQ774-15|Hymenoptera|Braconidae||BOLD:ACX5194
Braconidae|[8509]|GMAGR189-15|Hymenoptera|Braconidae||BOLD:ACX5030
Braconidae|[8510]|GMAGG224-15|Hymenoptera|Braconidae||BOLD:ACW4540
Alysiinae|[8511]|GMARA538-14|Hymenoptera|Braconidae|Alysiinae|BOLD:ACM9806
Alysiinae|[8512]|GMARO1038-14|Hymenoptera|Braconidae|Alysiinae|BOLD:ACO3097
Alysiinae|[8513]|GMAGG903-15|Hymenoptera|Braconidae|Alysiinae|BOLD:ACW1482
Alysiinae|[8514]|GMARI1425-14|Hymenoptera|Braconidae|Alysiinae|BOLD:ACN0448
Dinotrema|[8515]|GMARL1464-14|Hymenoptera|Braconidae|Alysiinae|BOLD:ACN2196
Dinotrema|[8516]|GMAGC1334-15|Hymenoptera|Braconidae|Alysiinae|BOLD:ACW5203
Dinotrema|[8517]|GMAGP1931-15|Hymenoptera|Braconidae|Alysiinae|BOLD:ACX4387
Dinotrema|[8518]|GMAGA733-15|Hymenoptera|Braconidae|Alysiinae|BOLD:ACM6420
Dinotrema|[8519]|GMARL1490-14|Hymenoptera|Braconidae|Alysiinae|BOLD:ACN1980
Dinotrema|[8520]|GMAGP1918-15|Hymenoptera|Braconidae|Alysiinae|BOLD:ACX6023
Dinotrema|[8521]|GMARM1193-14|Hymenoptera|Braconidae|Alysiinae|BOLD:ACN2876
Braconidae|[8522]|GMARI1441-14|Hymenoptera|Braconidae||BOLD:ACN0107
Braconidae|[8523]|GMARK2242-14|Hymenoptera|Braconidae||BOLD:ACN2149
Alysiinae|[8524]|GMAGG223-15|Hymenoptera|Braconidae|Alysiinae|BOLD:ACJ9057
Braconidae|[8525]|GMAGH808-15|Hymenoptera|Braconidae||BOLD:ACN2327
Alysiinae|[8526]|GMAGE1392-15|Hymenoptera|Braconidae|Alysiinae|BOLD:ACN0195
Alysiinae|[8527]|GMAGI1134-15|Hymenoptera|Braconidae|Alysiinae|BOLD:ACW5905
Aphaereta aotea|[8528]|GMARH1388-14|Hymenoptera|Braconidae|Alysiinae|BOLD:ABZ5166
Alysiinae|[8529]|GMARK2250-14|Hymenoptera|Braconidae|Alysiinae|BOLD:ACN2541
Alysiinae|[8530]|GMAGH798-15|Hymenoptera|Braconidae|Alysiinae|BOLD:ACW2573
Alysiinae|[8531]|GMARM1247-14|Hymenoptera|Braconidae|Alysiinae|BOLD:ACN2730
Alysiinae|[8532]|GMART1274-14|Hymenoptera|Braconidae|Alysiinae|BOLD:ACO0402
Braconidae|[8533]|GMAGE1385-15|Hymenoptera|Braconidae||BOLD:ACW1692
Braconidae|[8534]|GMARF1282-14|Hymenoptera|Braconidae||BOLD:ACN0785
Braconidae|[8535]|GMAGE1391-15|Hymenoptera|Braconidae||BOLD:ACN1647
Braconidae|[8536]|GMAGR186-15|Hymenoptera|Braconidae||BOLD:ACX4862
Braconidae|[8537]|GMARL1455-14|Hymenoptera|Braconidae||BOLD:ACN2159
Braconidae|[8538]|GMARD1972-14|Hymenoptera|Braconidae||BOLD:ABW7611
Braconidae|[8539]|GMARI1426-14|Hymenoptera|Braconidae||BOLD:ACN0879
Braconidae|[8540]|GMAGQ775-15|Hymenoptera|Braconidae||BOLD:ACX6311
Braconidae|[8541]|GMARA533-14|Hymenoptera|Braconidae||BOLD:ACM9647
Braconidae|[8542]|GMAGK1862-15|Hymenoptera|Braconidae||BOLD:ACX5735
Braconidae|[8543]|GMARH1366-14|Hymenoptera|Braconidae||BOLD:ACN0616
Braconidae|[8544]|GMAGE1339-15|Hymenoptera|Braconidae||BOLD:ACW7936
Braconidae|[8545]|GMAGL1340-15|Hymenoptera|Braconidae||BOLD:ACW8880
Aleiodes melanopterus|[8546]|GMARL027-14|Hymenoptera|Braconidae|Rogadinae|BOLD:AAH8806
Braconidae|[8547]|GMARA1690-14|Hymenoptera|Braconidae||BOLD:ACM5454
Braconidae|[8548]|GMARC872-14|Hymenoptera|Braconidae||BOLD:ACM5043
Braconidae|[8549]|GMAGH842-15|Hymenoptera|Braconidae||BOLD:ACW2568
Braconidae|[8550]|GMAGP1969-15|Hymenoptera|Braconidae||BOLD:ACX4850
Braconidae|[8551]|GMAGP1980-15|Hymenoptera|Braconidae||BOLD:ABW7612
Braconidae|[8552]|GMAGM1400-15|Hymenoptera|Braconidae||BOLD:ACX3663
Braconidae|[8553]|GMAGC1368-15|Hymenoptera|Braconidae||BOLD:ACN0434
Braconidae|[8554]|GMARM1195-14|Hymenoptera|Braconidae||BOLD:ACN2081
Braconidae|[8555]|GMARC883-14|Hymenoptera|Braconidae||BOLD:ACM6437
Braconidae|[8556]|GMAGT968-15|Hymenoptera|Braconidae||BOLD:ACX4660
Aleiodes|[8557]|GMARD027-14|Hymenoptera|Braconidae|Rogadinae|BOLD:ACN1382
Aleiodes|[8558]|GMARC113-14|Hymenoptera|Braconidae|Rogadinae|BOLD:ADB1136
Clinocentrus|[8559]|GMAGD1476-15|Hymenoptera|Braconidae|Rogadinae|BOLD:ACO2120
Braconidae|[8560]|GMAGG199-15|Hymenoptera|Braconidae||BOLD:ACN1155
Braconidae|[8561]|GMAGM1598-15|Hymenoptera|Braconidae||BOLD:ACX3807
Rogadinae|[8562]|GMARD1961-14|Hymenoptera|Braconidae|Rogadinae|BOLD:ACM9892

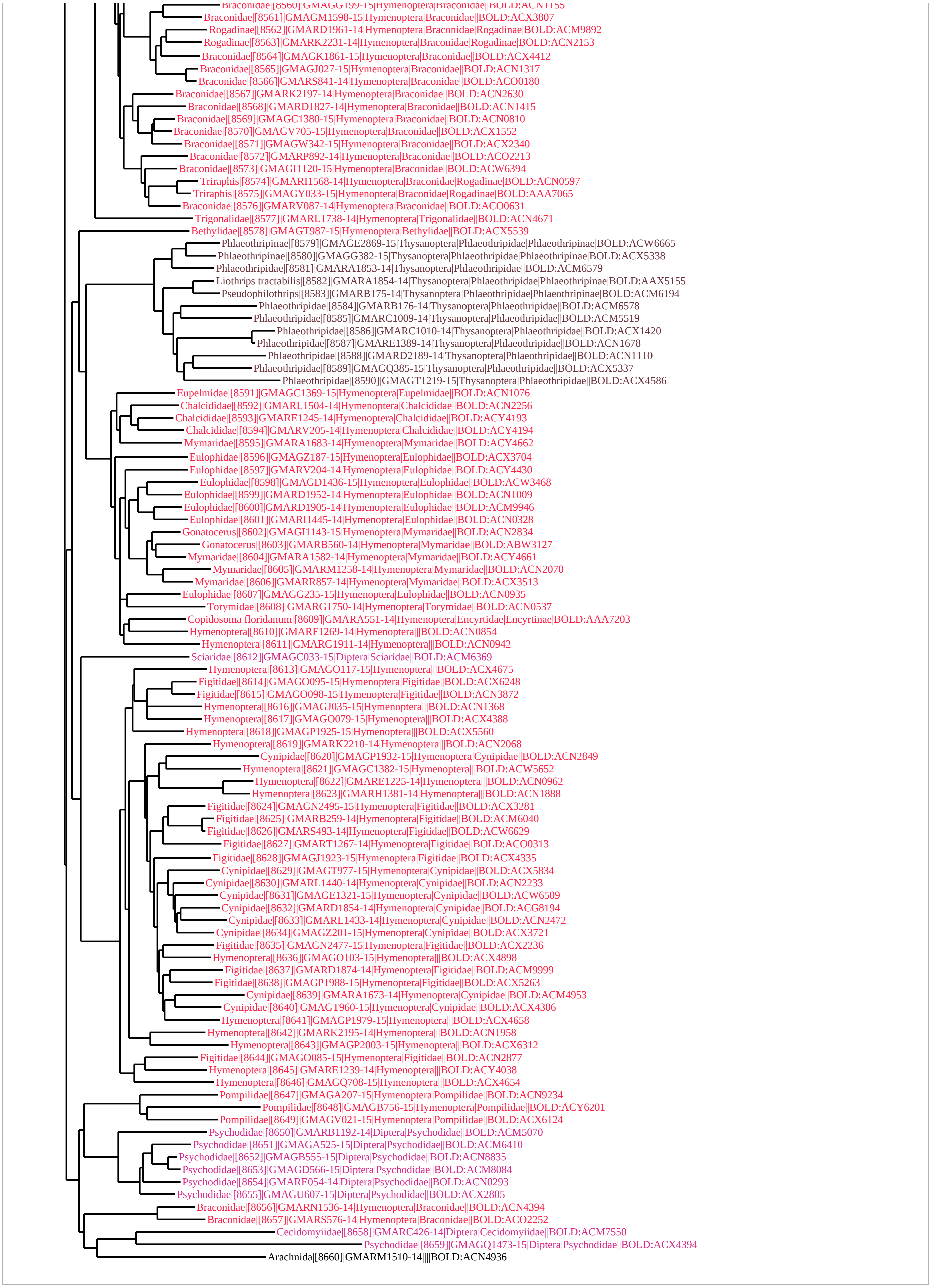

Braconidae|[8560]|GMAGG199-15|Hymenoptera|Braconidae||BOLD:ACN1155
Braconidae|[8561]|GMAGM1598-15|Hymenoptera|Braconidae||BOLD:ACX3807
Rogadinae|[8562]|GMARD1961-14|Hymenoptera|Braconidae|Rogadinae|BOLD:ACM9892
Rogadinae|[8563]|GMARK2231-14|Hymenoptera|Braconidae|Rogadinae|BOLD:ACN2153
Braconidae|[8564]|GMAGK1861-15|Hymenoptera|Braconidae||BOLD:ACX4412
Braconidae|[8565]|GMAGJ027-15|Hymenoptera|Braconidae||BOLD:ACN1317
Braconidae|[8566]|GMARS841-14|Hymenoptera|Braconidae||BOLD:ACO0180
Braconidae|[8567]|GMARK2197-14|Hymenoptera|Braconidae||BOLD:ACN2630
Braconidae|[8568]|GMARD1827-14|Hymenoptera|Braconidae||BOLD:ACN1415
Braconidae|[8569]|GMAGC1380-15|Hymenoptera|Braconidae||BOLD:ACN0810
Braconidae|[8570]|GMAGV705-15|Hymenoptera|Braconidae||BOLD:ACX1552
Braconidae|[8571]|GMAGW342-15|Hymenoptera|Braconidae||BOLD:ACX2340
Braconidae|[8572]|GMARP892-14|Hymenoptera|Braconidae||BOLD:ACO2213
Braconidae|[8573]|GMAGI1120-15|Hymenoptera|Braconidae||BOLD:ACW6394
Triraphis|[8574]|GMARI1568-14|Hymenoptera|Braconidae|Rogadinae|BOLD:ACN0597
Triraphis|[8575]|GMAGY033-15|Hymenoptera|Braconidae|Rogadinae|BOLD:AAA7065
Braconidae|[8576]|GMARV087-14|Hymenoptera|Braconidae||BOLD:ACO0631
Trigonalidae|[8577]|GMARL1738-14|Hymenoptera|Trigonalidae||BOLD:ACN4671
Bethylidae|[8578]|GMAGT987-15|Hymenoptera|Bethylidae||BOLD:ACX5539
Phlaeothripinae|[8579]|GMAGE2869-15|Thysanoptera|Phlaeothripidae|Phlaeothripinae|BOLD:ACW6665
Phlaeothripinae|[8580]|GMAGG382-15|Thysanoptera|Phlaeothripidae|Phlaeothripinae|BOLD:ACX5338
Phlaeothripidae|[8581]|GMARA1853-14|Thysanoptera|Phlaeothripidae||BOLD:ACM6579
Liothrips tractabilis|[8582]|GMARA1854-14|Thysanoptera|Phlaeothripidae|Phlaeothripinae|BOLD:AAX5155
Pseudophilothrips|[8583]|GMARB175-14|Thysanoptera|Phlaeothripidae|Phlaeothripinae|BOLD:ACM6194
Phlaeothripidae|[8584]|GMARB176-14|Thysanoptera|Phlaeothripidae||BOLD:ACM6578
Phlaeothripidae|[8585]|GMARC1009-14|Thysanoptera|Phlaeothripidae||BOLD:ACM5519
Phlaeothripidae|[8586]|GMARC1010-14|Thysanoptera|Phlaeothripidae||BOLD:ACX1420
Phlaeothripidae|[8587]|GMARE1389-14|Thysanoptera|Phlaeothripidae||BOLD:ACN1678
Phlaeothripidae|[8588]|GMARD2189-14|Thysanoptera|Phlaeothripidae||BOLD:ACN1110
Phlaeothripidae|[8589]|GMAGQ385-15|Thysanoptera|Phlaeothripidae||BOLD:ACX5337
Phlaeothripidae|[8590]|GMAGT1219-15|Thysanoptera|Phlaeothripidae||BOLD:ACX4586
Eupelmidae|[8591]|GMAGC1369-15|Hymenoptera|Eupelmidae||BOLD:ACN1076
Chalcididae|[8592]|GMARL1504-14|Hymenoptera|Chalcididae||BOLD:ACN2256
Chalcididae|[8593]|GMARE1245-14|Hymenoptera|Chalcididae||BOLD:ACY4193
Chalcididae|[8594]|GMARV205-14|Hymenoptera|Chalcididae||BOLD:ACY4194
Mymaridae|[8595]|GMARA1683-14|Hymenoptera|Mymaridae||BOLD:ACY4662
Eulophidae|[8596]|GMAGZ187-15|Hymenoptera|Eulophidae||BOLD:ACX3704
Eulophidae|[8597]|GMARV204-14|Hymenoptera|Eulophidae||BOLD:ACY4430
Eulophidae|[8598]|GMAGD1436-15|Hymenoptera|Eulophidae||BOLD:ACW3468
Eulophidae|[8599]|GMARD1952-14|Hymenoptera|Eulophidae||BOLD:ACN1009
Eulophidae|[8600]|GMARD1905-14|Hymenoptera|Eulophidae||BOLD:ACM9946
Eulophidae|[8601]|GMARI1445-14|Hymenoptera|Eulophidae||BOLD:ACN0328
Gonatocerus|[8602]|GMAGI1143-15|Hymenoptera|Mymaridae||BOLD:ACN2834
Gonatocerus|[8603]|GMARB560-14|Hymenoptera|Mymaridae||BOLD:ABW3127
Mymaridae|[8604]|GMARA1582-14|Hymenoptera|Mymaridae||BOLD:ACY4661
Mymaridae|[8605]|GMARM1258-14|Hymenoptera|Mymaridae||BOLD:ACN2070
Mymaridae|[8606]|GMARR857-14|Hymenoptera|Mymaridae||BOLD:ACX3513
Eulophidae|[8607]|GMAGG235-15|Hymenoptera|Eulophidae||BOLD:ACN0935
Torymidae|[8608]|GMARG1750-14|Hymenoptera|Torymidae||BOLD:ACN0537
Copidosoma floridanum|[8609]|GMARA551-14|Hymenoptera|Encyrtidae|Encyrtinae|BOLD:AAA7203
Hymenoptera|[8610]|GMARF1269-14|Hymenoptera|||BOLD:ACN0854
Hymenoptera|[8611]|GMARG1911-14|Hymenoptera|||BOLD:ACN0942
Sciaridae|[8612]|GMAGC033-15|Diptera|Sciaridae||BOLD:ACM6369
Hymenoptera|[8613]|GMAGO117-15|Hymenoptera|||BOLD:ACX4675
Figitidae|[8614]|GMAGO095-15|Hymenoptera|Figitidae||BOLD:ACX6248
Figitidae|[8615]|GMAGO098-15|Hymenoptera|Figitidae||BOLD:ACN3872
Hymenoptera|[8616]|GMAGJ035-15|Hymenoptera|||BOLD:ACN1368
Hymenoptera|[8617]|GMAGO079-15|Hymenoptera|||BOLD:ACX4388
Hymenoptera|[8618]|GMAGP1925-15|Hymenoptera|||BOLD:ACX5560
Hymenoptera|[8619]|GMARK2210-14|Hymenoptera|||BOLD:ACN2068
Cynipidae|[8620]|GMAGP1932-15|Hymenoptera|Cynipidae||BOLD:ACN2849
Hymenoptera|[8621]|GMAGC1382-15|Hymenoptera|||BOLD:ACW5652
Hymenoptera|[8622]|GMARE1225-14|Hymenoptera|||BOLD:ACN0962
Hymenoptera|[8623]|GMARH1381-14|Hymenoptera|||BOLD:ACN1888
Figitidae|[8624]|GMAGN2495-15|Hymenoptera|Figitidae||BOLD:ACX3281
Figitidae|[8625]|GMARB259-14|Hymenoptera|Figitidae||BOLD:ACM6040
Figitidae|[8626]|GMARS493-14|Hymenoptera|Figitidae||BOLD:ACW6629
Figitidae|[8627]|GMART1267-14|Hymenoptera|Figitidae||BOLD:ACO0313
Figitidae|[8628]|GMAGJ1923-15|Hymenoptera|Figitidae||BOLD:ACX4335
Cynipidae|[8629]|GMAGT977-15|Hymenoptera|Cynipidae||BOLD:ACX5834
Cynipidae|[8630]|GMARL1440-14|Hymenoptera|Cynipidae||BOLD:ACN2233
Cynipidae|[8631]|GMAGE1321-15|Hymenoptera|Cynipidae||BOLD:ACW6509
Cynipidae|[8632]|GMARD1854-14|Hymenoptera|Cynipidae||BOLD:ACG8194
Cynipidae|[8633]|GMARL1433-14|Hymenoptera|Cynipidae||BOLD:ACN2472
Cynipidae|[8634]|GMAGZ201-15|Hymenoptera|Cynipidae||BOLD:ACX3721
Figitidae|[8635]|GMAGN2477-15|Hymenoptera|Figitidae||BOLD:ACX2236
Hymenoptera|[8636]|GMAGO103-15|Hymenoptera|||BOLD:ACX4898
Figitidae|[8637]|GMARD1874-14|Hymenoptera|Figitidae||BOLD:ACM9999
Figitidae|[8638]|GMAGP1988-15|Hymenoptera|Figitidae||BOLD:ACX5263
Cynipidae|[8639]|GMARA1673-14|Hymenoptera|Cynipidae||BOLD:ACM4953
Cynipidae|[8640]|GMAGT960-15|Hymenoptera|Cynipidae||BOLD:ACX4306
Hymenoptera|[8641]|GMAGP1979-15|Hymenoptera|||BOLD:ACX4658
Hymenoptera|[8642]|GMARK2195-14|Hymenoptera|||BOLD:ACN1958
Hymenoptera|[8643]|GMAGP2003-15|Hymenoptera|||BOLD:ACX6312
Figitidae|[8644]|GMAGO085-15|Hymenoptera|Figitidae||BOLD:ACN2877
Hymenoptera|[8645]|GMARE1239-14|Hymenoptera|||BOLD:ACY4038
Hymenoptera|[8646]|GMAGQ708-15|Hymenoptera|||BOLD:ACX4654
Pompilidae|[8647]|GMAGA207-15|Hymenoptera|Pompilidae||BOLD:ACN9234
Pompilidae|[8648]|GMAGB756-15|Hymenoptera|Pompilidae||BOLD:ACY6201
Pompilidae|[8649]|GMAGV021-15|Hymenoptera|Pompilidae||BOLD:ACX6124
Psychodidae|[8650]|GMARB1192-14|Diptera|Psychodidae||BOLD:ACM5070
Psychodidae|[8651]|GMAGA525-15|Diptera|Psychodidae||BOLD:ACM6410
Psychodidae|[8652]|GMAGB555-15|Diptera|Psychodidae||BOLD:ACN8835
Psychodidae|[8653]|GMAGD566-15|Diptera|Psychodidae||BOLD:ACM8084
Psychodidae|[8654]|GMARE054-14|Diptera|Psychodidae||BOLD:ACN0293
Psychodidae|[8655]|GMAGU607-15|Diptera|Psychodidae||BOLD:ACX2805
Braconidae|[8656]|GMARN1536-14|Hymenoptera|Braconidae||BOLD:ACN4394
Braconidae|[8657]|GMARS576-14|Hymenoptera|Braconidae||BOLD:ACO2252
Cecidomyiidae|[8658]|GMARC426-14|Diptera|Cecidomyiidae||BOLD:ACM7550
Psychodidae|[8659]|GMAGQ1473-15|Diptera|Psychodidae||BOLD:ACX4394
Arachnida|[8660]|GMARM1510-14||||BOLD:ACN4936

S2 Fig.
