# Supplementary material for: DNA barcodes reveal striking arthropod diversity and unveil seasonal patterns of variation in the southern Atlantic Forest
Source: PLoS One. 2022 Apr 28;17(4):e0267390. doi: 10.1371/journal.pone.0267390 (PMC9049551; doi:10.1371/journal.pone.0267390)
Supplement: S1 Table — The pair order compared, the difference of the proportions of the orders compared, the critical value, the Chi-square statistic, and the p-value are informed. (DOCX) [file pone.0267390.s008.docx]

**S1 Table.**

| Order | Difference of proportions | Critical value | X^2^ | *p*-value |
| --- | --- | --- | --- | --- |
| Coleptera-Diptera | 0.172 | 0.073 | 62.16 | *p* < 0.05 |
| Coleoptera-Hemiptera | 0.259 | 0.098 | 77.33 | *p* < 0.05 |
| Coleoptera-Hymenoptera | -0.012 | 0.087 | 0.22 | 0.998 |
| Coleoptera-Lepidoptera | 0.139 | 0.090 | 26.50 | *p* < 0.05 |
| Coleoptera-Psocoptera | 0.160 | 0.206 | 6.70 | 0.243 |
| Diptera-Hemiptera | 0.086 | 0.073 | 15.55 | *p* < 0.05 |
| Diptera-Hymenoptera | -0.184 | 0.058 | 113.62 | *p* < 0.05 |
| Diptera-Lepidoptera | -0.032 | 0.062 | 3.06 | 0.689 |
| Diptera-Psocoptera | -0.012 | 0.195 | 0.04 | 0.999 |
| Hemiptera-Hymenoptera | -0.271 | 0.087 | 106.78 | *p* < 0.05 |
| Hemiptera-Lepidoptera | -0.119 | 0.090 | 19.25 | *p* < 0.05 |
| Hemiptera-Psocoptera | -0.098 | 0.206 | 2.51 | 0.774 |
| Hymenoptera-Lepidoptera | 0.152 | 0.078 | 41.38 | *p* < 0.05 |
| Hymenoptera-Psocoptera | 0.173 | 0.201 | 8.14 | 0.148 |
| Lepidoptera-Psocoptera | 0.021 | 0.203 | 0.11 | 0.999 |
