# Supplementary material for: DNA barcodes reveal striking arthropod diversity and unveil seasonal patterns of variation in the southern Atlantic Forest
Source: PLoS One. 2022 Apr 28;17(4):e0267390. doi: 10.1371/journal.pone.0267390 (PMC9049551; doi:10.1371/journal.pone.0267390)
Supplement: S2 Table — Their taxonomic details, the total number of individuals of each BIN, and the number of weeks in which it was collected are reported. (DOCX) [file pone.0267390.s009.docx]

**S2 Table.**

| BIN | Order | Family | Species | Abundance | Week count | |
| --- | --- | --- | --- | --- | --- | --- |
| BOLD:ACM5798 | Diptera | Sciaridae | Unknown | 1572 | 43 | |
| BOLD:ACM5158 | Diptera | Sciaridae | Unknown | 1241 | 36 |  |
| BOLD:ACM6616 | Diptera | Sciaridae | Unknown | 793 | 46 |  |
| BOLD:ACM5562 | Diptera | Sciaridae | Unknown | 743 | 43 |  |
| BOLD:ACM5153 | Diptera | Chironomidae | Unknown | 704 | 39 |  |
| BOLD:ACM7619 | Diptera | Ceratopogonidae | Unknown | 637 | 38 |  |
| BOLD:ACM4716 | Diptera | Sciaridae | Unknown | 616 | 30 |  |
| BOLD:ACN1102 | Coleoptera | Curculionidae | Unknown | 610 | 27 |  |
| BOLD:ACL4422 | Diptera | Sciaridae | Unknown | 546 | 23 |  |
| BOLD:ACM6089 | Diptera | Sciaridae | Unknown | 533 | 28 |  |
| BOLD:ACM5257 | Diptera | Sciaridae | Unknown | 527 | 42 |  |
| BOLD:ACM7503 | Diptera | Chironomidae | Unknown | 519 | 23 |  |
| BOLD:ACM5611 | Diptera | Sciaridae | Unknown | 454 | 44 |  |
| BOLD:ACK9746 | Diptera | Phoridae | Unknown | 433 | 31 |  |
| BOLD:ACM7389 | Diptera | Sciaridae | Unknown | 429 | 38 |  |
| BOLD:ACM4760 | Diptera | Sciaridae | Unknown | 412 | 34 |  |
| BOLD:ACM8351 | Diptera | Sciaridae | Unknown | 403 | 40 |  |
| BOLD:ACM6072 | Hemiptera | Cicadellidae | Unknown | 395 | 37 |  |
| BOLD:ACM5034 | Diptera | Cecidomyiidae | Unknown | 390 | 36 |  |
| BOLD:ABX4353 | Diptera | Sciaridae | Unknown | 367 | 38 |  |
| BOLD:ACN9434 | Diptera | Cecidomyiidae | Unknown | 338 | 39 |  |
| BOLD:ACM5415 | Diptera | Sciaridae | Unknown | 334 | 44 |  |
| BOLD:ACM5023 | Diptera | Ceratopogonidae | Unknown | 310 | 32 |  |
| BOLD:ACM5750 | Hemiptera | Cicadellidae | Unknown | 289 | 35 |  |
| BOLD:ACN1455 | Diptera | Chironomidae | Unknown | 288 | 17 |  |
| BOLD:ACM6087 | Diptera | Sciaridae | Unknown | 286 | 23 |  |
| BOLD:ACM5276 | Hemiptera | Cicadellidae | Unknown | 245 | 34 |  |
| BOLD:ACM8565 | Diptera | Chironomidae | Unknown | 234 | 11 |  |
| BOLD:ACM4746 | Psocoptera | Caeciliusidae | Unknown | 214 | 41 |  |
| BOLD:ACN0745 | Diptera | Tachinidae | Unknown | 203 | 15 |  |
| BOLD:ACM6084 | Lepidoptera | Bucculatricidae | Unknown | 139 | 36 |  |
| BOLD:ACM4866 | Lepidoptera | Tineidae | Unknown | 135 | 27 |  |
| BOLD:AAJ7273 | Lepidoptera | Geometridae | Unknown | 134 | 36 |  |
| BOLD:ACC4180 | Hymenoptera | Formicidae | *Pheidole sp.* | 110 | 21 |  |
| BOLD:ACY5761 | Lepidoptera | Gelechiidae | Unknown | 105 | 22 |  |
| BOLD:ACM5303 | Hymenoptera | Formicidae | Unknown | 96 | 13 |  |
| BOLD:ACM5911 | Lepidoptera | Bucculatricidae | Unknown | 94 | 27 |  |
| BOLD:AAZ4402 | Hymenoptera | Formicidae | *Pheidole sp.* | 75 | 13 |  |
